# Molecular architecture of GLUT4 trafficking and exocytosis in adipocytes and skeletal muscle: A systematic review and integrated conceptual framework

**DOI:** 10.3389/fendo.2026.1932455

**Published:** 2026-10-19

**Authors:** Hieu Si Nguyen, Kien Trung Tran, Manh Sy Ho, Linh Mai Nguyen, Tri Min Tran, Phuong Ha Vu, Loc Tien Nguyen, Lam The Pham, Ha Ngoc Pham, Dung Ti My Phan, Manh Duc Pham, Ky Dinh Nguyen, Hanh Thi Minh Bui, Toshio Hosaka, Nam Hoang Tran, Hiep Hoang Phan, Chung Thi Kim Le

**Affiliations:** 1 Hanoi Medical University, Hanoi, Vietnam; 2 Department of Food and Nutritional Sciences, University of Shizuoka, Shizuoka, Japan; 3 Tokushima University, Tokushima, Japan; 4 National Hospital of Endocrinology, Hanoi, Vietnam

**Keywords:** adipocytes, exocytosis, glucose transporter type 4, insulin signaling, molecular trafficking, skeletal muscle

## Abstract

**Introduction:**

GLUT4 exocytosis links insulin signaling to cellular glucose disposal in adipocytes and skeletal muscle. Evidence for its molecular regulation is dispersed across experimental models, trafficking stages, and signaling pathways. We systematically mapped the molecular evidence underlying GLUT4 vesicle mobilization, tethering, docking, and membrane fusion and compared adipocyte and skeletal-muscle mechanisms.

**Methods:**

We searched PubMed (MEDLINE), Scopus, and Embase, supplemented by ProQuest Dissertations & Theses and preprint repositories. Primary experimental studies examining molecular regulators of GLUT4 exocytosis using in vitro and in vivo models were eligible. Two reviewers independently performed study selection, data extraction, and quality appraisal in accordance with PRISMA 2020. The protocol was prospectively registered in PROSPERO. Findings were synthesized narratively and mapped using the prespecified evidence-tier framework.

**Results:**

Of 15,835 identified records, 350 primary experimental studies met the eligibility criteria. The evidence map identified 206 distinct molecular entries in adipocyte studies and 109 in skeletal-muscle studies, representing the breadth of reported associations rather than uniformly validated regulators. Both tissues showed partially overlapping late trafficking machinery involving SNARE- and exocyst-related components. Adipocyte evidence most consistently supported AKT2 -TBC1D4 (AS160) -RAB10 signaling together with TC10- and RAC1-associated cortical modules. Skeletal-muscle pathways integrated insulin-responsive signaling with contraction-, AMPK-, and calcium-associated inputs involving TBC1D1/TBC1D4, RAC1, and RAB8A/RAB13. Direct imaging and perturbation experiments provided the strongest evidence for step-specific roles, but much of the evidence derived from differentiated cell lines and rodent models.

**Discussion:**

GLUT4 trafficking in both tissues follows a shared broad logic of signal-dependent vesicle release, cortical targeting, and membrane insertion, while individual regulatory nodes differ in their contribution and evidence base. These findings support signal-to-trafficking coupling as a relevant mechanistic layer of insulin resistance and identify pathways requiring validation in physiologically relevant human models before therapeutic translation.

**Systematic Review registration:**

https://www.crd.york.ac.uk/PROSPERO/, identifier CRD420251105528.

## 1 Introduction

Glucose homeostasis depends on the coordinated regulation of hormonal signaling and cellular glucose transport. GLUT4 is the principal insulin-responsive facilitative glucose transporter expressed in adipocytes and skeletal muscle and plays a major role in postprandial glucose disposal. In insulin resistance, defects in GLUT4 trafficking can limit transporter delivery to the plasma membrane and thereby impair cellular glucose uptake. These trafficking abnormalities should be viewed as one component of a multifactorial pathophysiology, acting alongside impaired receptor-proximal insulin signaling, lipotoxicity and ceramide accumulation, chronic tissue inflammation, mitochondrial stress, and reduced total GLUT4 expression (1–4). Accordingly, defining the molecular mechanisms that regulate GLUT4 mobilization to the plasma membrane may reveal a complementary layer of insulin-resistance biology and help prioritize targets for future therapeutic investigation.

Unlike constitutively expressed transporters, GLUT4 resides predominantly within specialized intracellular storage vesicles under basal conditions. Following insulin stimulation, these vesicles undergo a tightly regulated sequence of trafficking events that includes release from retention compartments, cytoskeletal transport, tethering, docking, membrane fusion, and surface retention. This process is orchestrated through integrated signaling networks involving the insulin receptor–IRS–PI3K–AKT axis, Rab GTPases, actin-remodeling proteins, molecular motors, and SNARE-associated fusion machinery (3–7). Disruption at any of these checkpoints may reduce glucose uptake despite apparently preserved upstream receptor signaling, highlighting that insulin action is fundamentally a trafficking-dependent biological phenomenon rather than a purely signaling-dependent one.

Over the past three decades, mechanistic understanding of GLUT4 biology has evolved considerably. Early biochemical fractionation studies established the principle of insulin-stimulated transporter redistribution, whereas later genetic and imaging approaches identified specific regulators such as TBC1D4 (AS160), TBC1D1, RAB10, the exocyst complex, STX4 (syntaxin-4), and MUNC18C (STXBP3). More recently, live-cell imaging, total internal reflection fluorescence microscopy, endogenous tagging, and single-vesicle analyses have refined the field from a static translocation model toward a dynamic view of regulated exocytosis with step-specific control nodes (7–10). These advances have also revealed substantial tissue specificity in the organization of GLUT4 trafficking.

Although adipose tissue and skeletal muscle share core downstream fusion machinery, their upstream regulatory logic differs markedly. Adipocytes are optimized for rapid nutrient storage and rely predominantly on insulin-dependent signaling. In contrast, skeletal muscle integrates insulin signaling with contraction-, calcium-, and AMP-activated pathways, allowing glucose uptake to remain responsive during exercise even when systemic insulin sensitivity is impaired (6, 11–13). This biological divergence has important translational implications, as it suggests that distinct trafficking nodes may represent therapeutic targets in different metabolic tissues.

Despite extensive experimental literature, the molecular regulation of GLUT4 trafficking has not yet been systematically synthesized. Existing reviews have provided important narrative accounts of insulin signaling, GLUT4 translocation, and selected trafficking mechanisms, but no systematic review has comprehensively mapped the experimental evidence on GLUT4 trafficking across models, tissues, and molecular stages. This gap is particularly evident for the exocytotic phase of GLUT4 trafficking, in which vesicle mobilization, tethering, docking, and membrane fusion convert upstream signaling into functional transporter insertion at the plasma membrane. Because these steps are distributed across different experimental systems, imaging approaches, and molecular subfields, the available evidence remains difficult to compare and interpret within a unified framework. To our knowledge, no previous systematic review has cataloged the molecular regulators of GLUT4 exocytosis and directly compared conserved versus tissue-specific mechanisms between adipocytes and skeletal muscle.

Therefore, the present systematic review aimed to comprehensively evaluate the molecular components and regulatory mechanisms governing GLUT4 exocytosis in experimental models. Specifically, we sought to: (i) identify key proteins and signaling pathways involved in vesicle mobilization, tethering, docking, and fusion; (ii) compare conserved versus tissue-specific mechanisms between adipocytes and skeletal muscle; and (iii) synthesize potential therapeutic applications. By integrating evidence from 350 studies, this review provides an updated conceptual framework for understanding how insulin signaling is converted into effective cellular glucose transport.

## 2 Methods

This study is a systematic review of the molecular components and regulatory mechanisms involved in GLUT4 exocytosis in experimental models. The review protocol was developed *a priori* and registered in PROSPERO (CRD420251105528) to ensure methodological transparency and to minimize selection bias.

The conduct and reporting of this review adhere to the PRISMA 2020 statement for systematic reviews (14) and the PRISMA-S guidelines for reporting literature search strategies (15).

### 2.1 Search method

A comprehensive literature search strategy was developed to identify experimental studies investigating the exocytotic phase of GLUT4 trafficking. The final database search was conducted on 10 August 2025. We searched PubMed/MEDLINE, Scopus, and Embase from database inception to 10 August 2025, with no restriction on publication year. To maximize coverage, supplementary searches were performed by searching Google Scholar and reviewing ProQuest Dissertations & Theses and biological preprint servers, including bioRxiv. The search focused on primary experimental studies related to GLUT4 exocytosis, including signaling, mobilization, tethering, docking, and membrane fusion. Only articles published in English were considered eligible. The search strategy was structured using a PICO-based framework: the population included *in vitro* adipocyte and skeletal muscle cell models, as well as ex vivo and *in vivo* animal models expressing endogenous or recombinant GLUT4; the intervention included experimental manipulations designed to activate, inhibit, or modulate GLUT4 exocytosis, such as insulin stimulation, contraction or AICAR treatment, pharmacological inhibition, cellular stress, knockout, knockdown, or overexpression; no specific comparator was required; and outcomes included molecular components, functional roles, and regulatory mechanisms involved in the exocytotic trafficking of GLUT4. The detailed search strategy is described in **Supplementary File 1** (search strategy).

### 2.2 Eligibility criteria

Eligible studies were included if they involved any *in vitro* cell models (primary adipocytes or primary skeletal muscle cells, and differentiated immortalized precursor cell lines, such as C2C12 and 3T3-L1) or *in vivo* animal models (skeletal muscle, or adipose tissue) with endogenous GLUT4 expression participating in glucose transport. Eligible interventions included insulin, contraction or exercise-related metabolic stimuli, pharmacological modulation, cellular stress, and genetic manipulation such as knockout, knockdown, mutation, or overexpression. Eligible outcomes included GLUT4 vesicle mobilization, cytoskeletal transport, tethering, docking, membrane fusion, cell-surface insertion, or related molecular mechanisms.

Studies were excluded if they were reviews, meta-analyses, editorials, protocols, conference abstracts without sufficient experimental data, purely computational or mathematical studies without experimental validation, human-only studies, or studies not directly assessing GLUT4 trafficking/exocytosis. Studies using artificial GLUT4 expression in unrelated heterologous systems were excluded unless they were used for mechanistic validation directly relevant to GLUT4 exocytosis.

### 2.3 Data extraction

All retrieved references were imported into EndNote (16) and then uploaded to Rayyan (17) for automated duplicate detection. The screening process was conducted independently by two reviewers in two stages (1): title and abstract screening and (2) full-text assessment based on the predefined eligibility criteria. Rayyan was used to facilitate a blinded screening workflow. Any disagreements between the two reviewers were resolved through discussion or, when necessary, consultation with a third reviewer. The extracted information included: DOI, first author, publication year, country, study aim, study population, intervention/exposure, outcome measurement techniques, timing of outcome assessment, comparator group, key findings, primary molecule(s) investigated, direction of effect on exocytosis, newly identified mechanisms, and the corresponding stage within the GLUT4 exocytosis process.

### 2.4 Evidence-tier classification and model provenance

The prespecified tier system was applied at the level of a study-molecule-model claim. It was not a measure of study quality, a proxy for biological importance, or a permanent property of a molecule. A molecule could have Tier 1 evidence in one model and Tier 3 or Tier 4 evidence in another. Direct GLUT4 trafficking endpoints were required for Tier 1–3 assignment; glucose uptake, protein expression, phosphorylation, or colocalization alone were classified as Tier 4 unless paired with an appropriate trafficking endpoint. Model provenance was coded separately as immortalized/differentiated cell line, primary or ex vivo rodent tissue, *in vivo* rodent genetic model, or human-derived material. The full operational definitions are shown in **Table 1**.

**Table 1:** Prespecified evidence-tier framework for study-molecule-model claims in the systematic review.

Tier	Minimum evidentiary requirement	Interpretation in the synthesis
Tier 1 Convergent mechanistic evidence	A perturbation/rescue experiment plus interaction, localization, or temporal-order evidence and a direct GLUT4 trafficking endpoint in the same biological context.	Supports a mechanistic model in the stated model system.
Tier 2 Functional mechanistic evidence	Genetic perturbation with a direct trafficking endpoint, or interaction/localization evidence paired with a trafficking endpoint; rescue and full mechanistic convergence are incomplete.	Supports a functional contribution in the stated model system.
Tier 3 Pharmacological functional evidence	Pharmacological perturbation with a direct trafficking endpoint, but target specificity or causal assignment remains limited.	Signals a candidate functional pathway that needs genetic validation.
Tier 4 Descriptive or correlative evidence	Expression, phosphorylation, colocalization, glucose uptake alone, disease association, or insufficiently specified trafficking evidence.	Documents association or presence, not a trafficking mechanism.

### 2.5 Quality appraisal

The methodological quality of the included studies was assessed independently by two reviewers; any disagreements were resolved through discussion or consultation with a third reviewer.

For *in vivo* animal studies, we used the SYRCLE risk-of-bias tool (18) to assess risks of bias related to selection, performance, detection, attrition, and reporting. For *in vitro* cell-based studies, because no universally accepted risk-of-bias tool exists for mechanistic cell-culture studies, we applied an *a priori* customized methodological reporting checklist informed by life-science reporting standards and cell-line authentication recommendations (19) (20),. The checklist assessed six domains: cell line source/authentication or traceability, specification of biological replicates, use of appropriate internal controls, randomization or unbiased allocation of culture plates/wells, blinding of outcome assessment where feasible, and appropriateness of statistical analysis. This checklist was used to evaluate methodological rigor and reporting adequacy rather than to exclude studies from the synthesis.

The results of the risk-of-bias assessment were visualized using “traffic light plots” generated with the robvis (21) tool. Studies assessed as having a high risk of bias were not excluded; however, sensitivity analyses were performed when necessary to examine the robustness of the synthesized findings.

### 2.6 Data synthesis

Due to substantial heterogeneity in experimental models, types of interventions, and outcome measurement methods, a quantitative meta-analysis was not performed. Instead, the data were synthesized using a structured narrative synthesis approach.

The synthesis focused exclusively on the exocytosis stage of GLUT4, with findings organized according to the key mechanistic steps: vesicle tethering, vesicle docking, and membrane fusion. Regulatory mechanisms were compared between skeletal muscle and adipose tissue to identify both shared and tissue-specific factors.

### 2.7 Ethics statement

This study did not require ethical approval because it involved only the use and analysis of previously published, publicly available data.

## 3 Results

### 3.1 Study selection

From an initial 15,835 records, 350 studies satisfying the eligibility criteria were included in this systematic review following a thorough screening process. The detailed selection procedure is illustrated in the PRISMA flow diagram (**Figure 1**).

**Figure 1:**
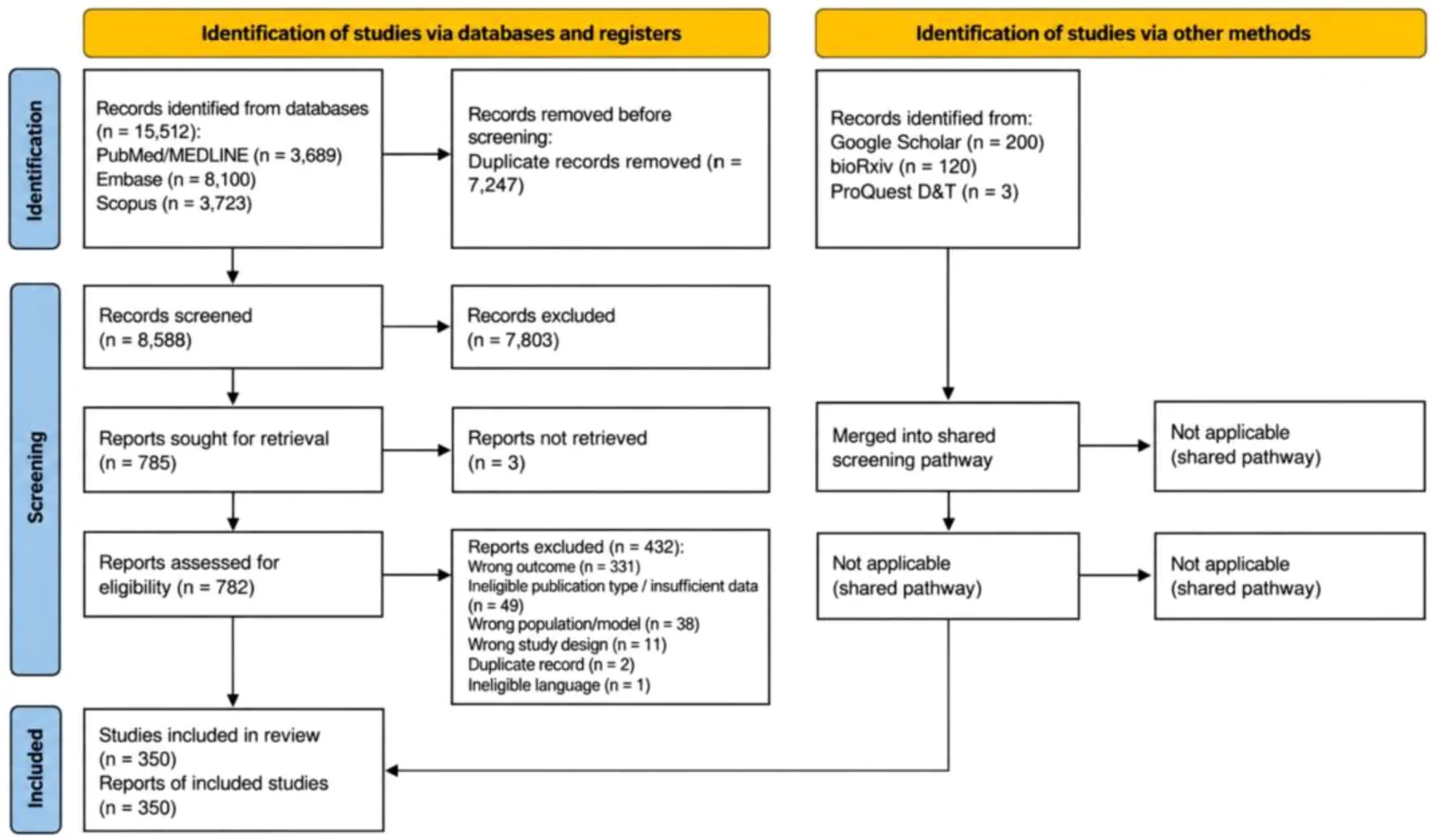
PRISMA flow diagram. The diagram illustrates the flow of information through the different phases of the systematic review, including identification, screening, and inclusion of studies.

### 3.2 Study characteristics

The 350 included studies encompassed a diverse range of experimental models and interventions relevant to GLUT4 trafficking. Consistent with the eligibility criteria, the experimental models were categorized into *in vitro* systems, predominantly utilizing 3T3-L1 adipocytes and L6 myoblasts, and ex vivo or *in vivo* models involving isolated skeletal muscle (e.g., soleus, epitrochlearis) and adipose tissue from rodent models.

Interventions examined in these studies included physiological stimulation via insulin and contraction/exercise-related metabolic stimuli (e.g., AICAR), as well as pharmacological inhibition (e.g., wortmannin) and genetic manipulation (knockdown or overexpression of specific proteins). The primary outcomes assessed were GLUT4 translocation dynamics, glucose uptake, and the phosphorylation status of signaling intermediates, measured using techniques such as Western blotting, immunofluorescence microscopy, and glucose uptake assays. A summary of the characteristics of all included studies is provided in **Supplementary Table S1**.

### 3.3 Risk of Bias assessment

Methodological quality was assessed separately for 60 *in vivo* animal-study reports using the SYRCLE risk-of-bias tool (**Figure 2A**) and for 338 *in vitro* cell-based reports using a predefined six-item methodological-rigor checklist (**Figure 2B**). Because some reports included both model types, the denominators for the two panels are not mutually exclusive.

**Figure 2:**
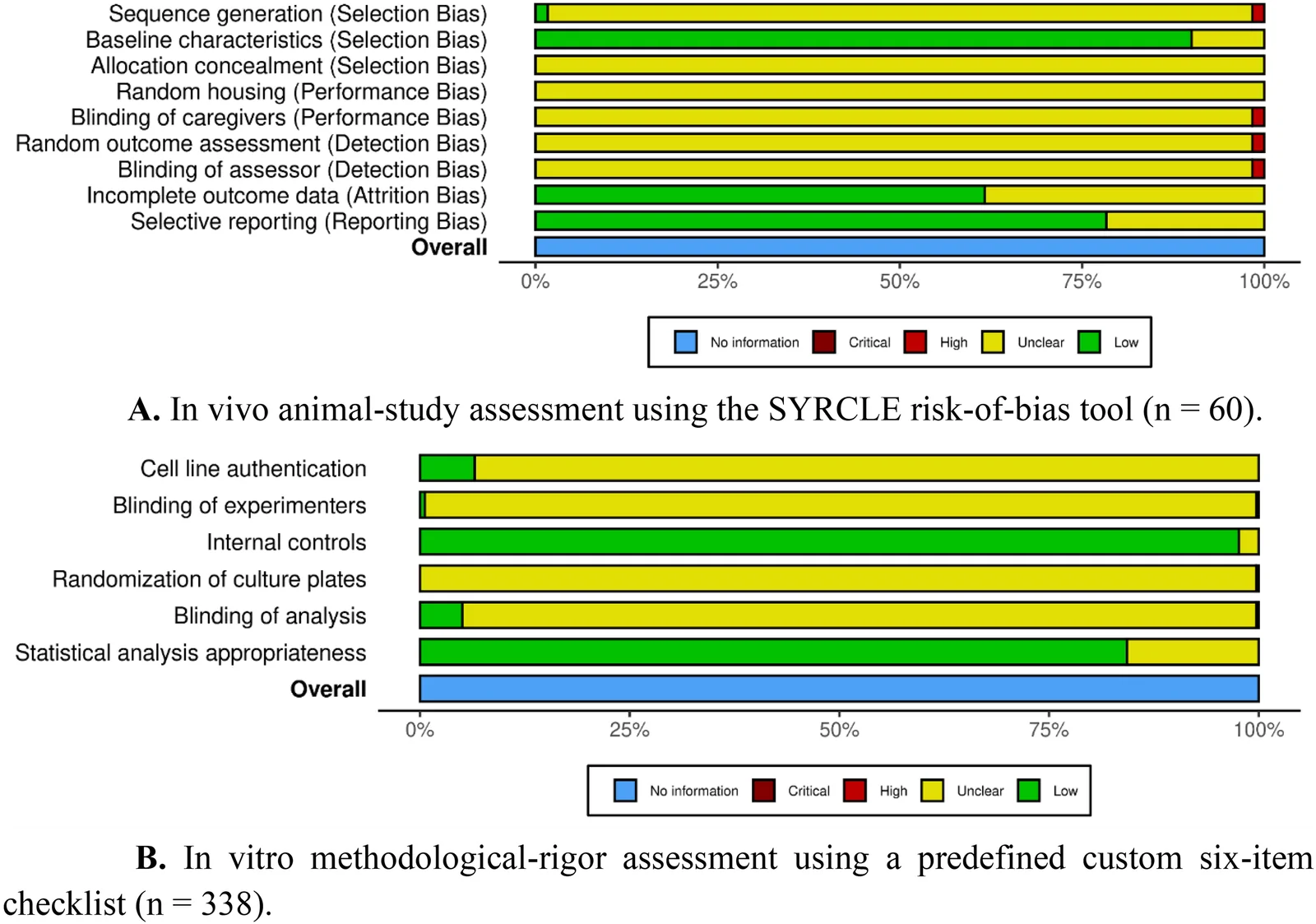
**(A)** *In vivo* animal-study assessment using the SYRCLE risk-of-bias tool (n = 60). **(B)** *In vitro* methodological-rigor assessment using a predefined custom six-item checklist (n = 338). Summary of risk-of-bias and methodological-rigor assessments. Reports containing both model types contributed to both relevant panels; therefore, the denominators are not mutually exclusive.

In the animal-study assessment, incomplete reporting was the predominant concern. Sequence generation was rated as unclear in 58/60 reports (96.7%), while allocation concealment and random housing were unclear in all reports. Caregiver blinding, random outcome assessment, and assessor blinding were also unclear in 59/60 reports (98.3% each). In contrast, baseline characteristics were rated as low risk in 54/60 reports (90.0%), incomplete outcome data in 37/60 (61.7%), and selective reporting in 47/60 (78.3%). Explicit high-risk judgments were uncommon.

For *in vitro* studies, appropriate internal controls and statistical analysis were commonly reported and were rated as low risk in 330/338 (97.6%) and 284/338 (84.0%) reports, respectively. In contrast, cell-line authentication, blinding of experimenters, randomization of culture plates, and blinding of analysis were rarely reported and were predominantly rated as unclear. Thus, across both assessments, the principal limitation was insufficient reporting of safeguards against bias rather than a high frequency of explicitly high-risk methods.

### 3.4 Molecular components involved in GLUT4 trafficking

**Supplementary Tables S2** and **S3** provide a study-level molecular evidence map for GLUT4 trafficking in adipocytes and skeletal muscle, respectively. Each entry assigns a primary molecule to the exocytic phase in which it was examined, including signaling, vesicle translocation, tethering, docking, and membrane fusion, and links the finding to its original reference. For the present tabulation, explicitly reported isoforms, phosphorylation states, and named variants were retained as separate molecular entries; only labels that were identical across different phases were counted once in the overall total. Therefore, phase-specific counts are not mutually exclusive and should not be summed without accounting for repeated molecules across stages.

In adipocytes (**Supplementary Table S2**), 83 molecular entries were identified in signaling, 60 in translocation, 14 in tethering, 13 in docking, and 53 in fusion. These 223 phase-specific entries corresponded to 206 distinct molecular entries after removal of 17 repeated labels occurring in more than one phase. In skeletal muscle (**Supplementary Table S3**), 52 entries were identified in signaling, 37 in translocation, 5 in tethering, 5 in docking, and 21 in fusion. These 120 phase-specific entries corresponded to 109 distinct molecular entries after removal of 11 repeated labels across phases. Thus, signaling represented the largest reported category in both tissues, whereas the terminal tethering, docking, and fusion machinery comprised fewer molecular entries. The larger inventory in adipocytes should be interpreted as the breadth of reported molecular associations rather than as evidence that every listed molecule has an equivalent or universally established role.

**Figures 3**, **4** complement **Supplementary Tables S2** and **S3** by presenting integrated regulatory models of GLUT4 trafficking in adipocytes and skeletal muscle, respectively. In these figures, node colors encode the evidence category represented for each molecular claim: red denotes Tier 1 evidence; yellow denotes Tier 2 evidence based on physical interaction/localization study; dark blue denotes Tier 2 evidence based on genetic perturbation; green denotes Tier 2 evidence that is complementary, combining findings from distinct studies—such as a genetic perturbation study alongside a separate physical interaction/localization study—each meeting Tier 2 criteria; black denotes Tier 3 evidence; and gray denotes Tier 4 evidence. This color scheme reflects the nature and strength of the supporting evidence, rather than the biological importance, direction of effect, or magnitude of the role of a molecule. The figures should therefore be interpreted as visual syntheses of the study-level evidence maps, not as single linear pathways established uniformly in every model system.

**Figure 3:**
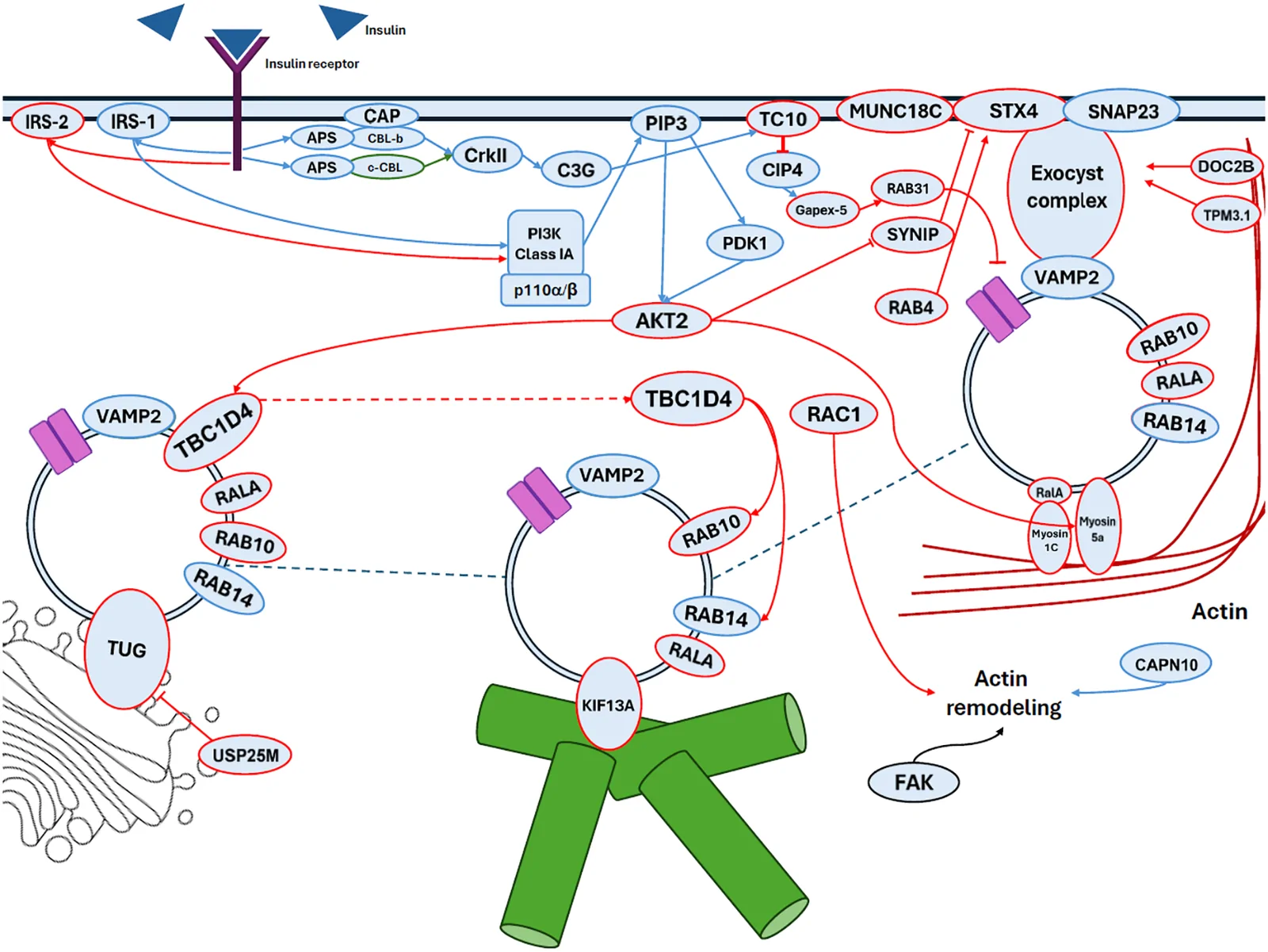
Integrated molecular model of insulin-stimulated GLUT4 trafficking in adipocytes.

**Figure 4:**
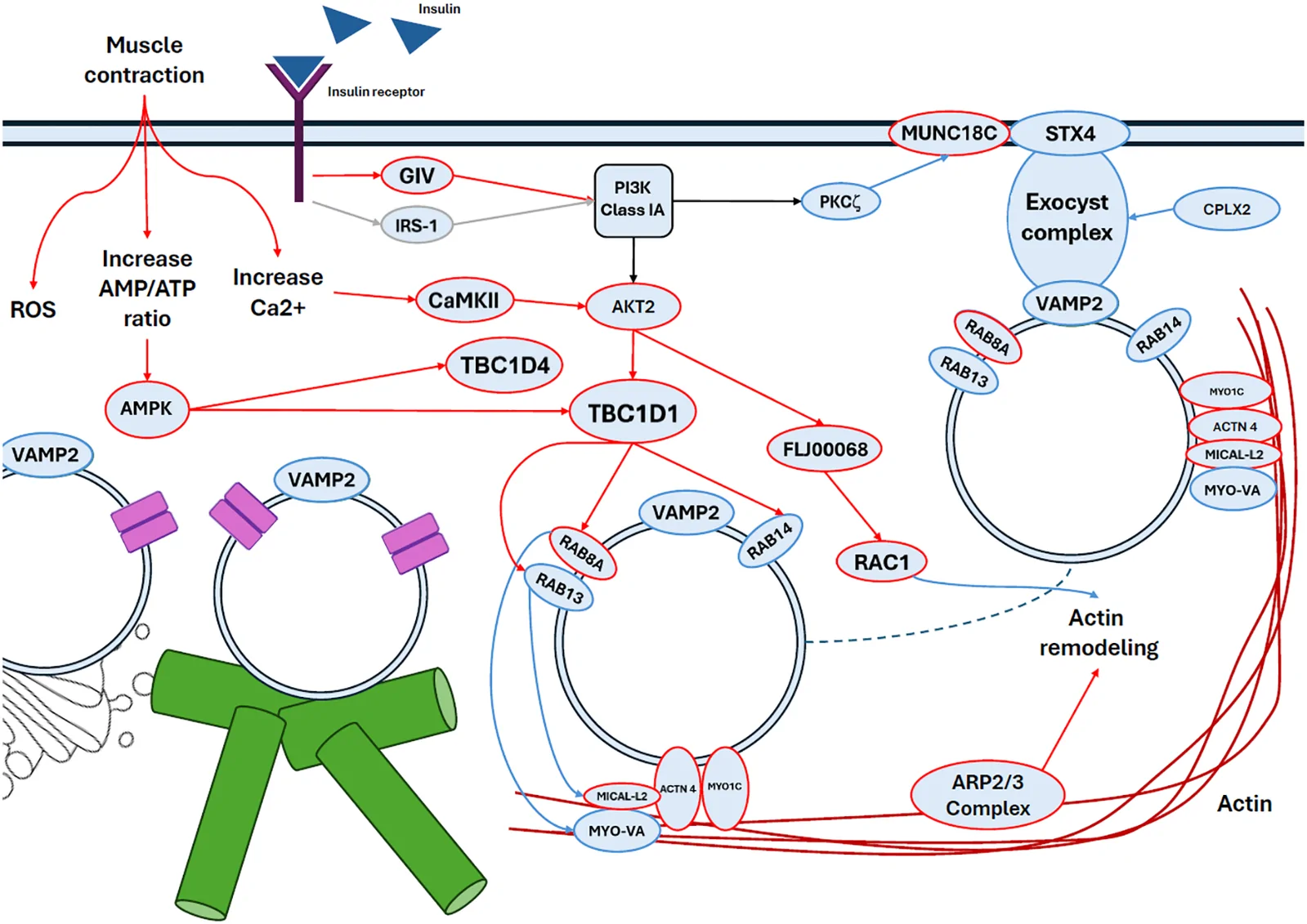
Integrated model of insulin- and contraction-regulated GLUT4 trafficking in skeletal muscle.

To make the provenance and strength of each molecular claim transparent, **Supplementary Tables S2** and **S3** also report the primary model system, intervention class, and intervention tier. The primary model system field records the biological context in which each claim was obtained. Much of the mechanistic evidence derives from differentiated cell lines, particularly 3T3-L1 adipocytes and L6/C2C12 myotubes, whereas primary rodent cells, ex vivo adipose or muscle preparations, and *in vivo* genetic models strengthen selected nodes. Human-derived material remains limited, and heterologous systems such as CHO or COS cells are used chiefly for mechanistic validation. Accordingly, each regulator is interpreted only within the model system in which the relevant evidence was generated.

The intervention class field specifies how the association was tested, including genetic perturbation or rescue, pharmacological perturbation, physiological stimulation, disease or stress modeling, and combinations of these approaches. Recording this information is essential because a change in glucose uptake, translocation, or downstream signaling alone cannot establish a direct or selective trafficking mechanism.

### 3.5 Integrated regulatory model of GLUT4 trafficking

#### 3.5.1 Regulatory networks of GLUT4 trafficking in adipocytes

The adipocyte model in **Figure 3** is a map of convergent experimental modules rather than a single pathway established in every adipocyte preparation. Most causal studies were performed in 3T3-L1 adipocytes; primary rodent, adipocyte-specific knockout, and human systems selectively strengthen particular nodes.

##### 3.5.1.1 Insulin signaling, AKT2–TBC1D4, and rho-family modules

In differentiated 3T3-L1 adipocytes, insulin-stimulated AKT2 signaling has Tier 1 evidence at the SYNIP (STX4IP)/STX4 checkpoint, where AKT2-dependent SYNIP phosphorylation facilitates GLUT4 vesicle docking and fusion (22). Independent functional data from primary mouse adipocytes and *Akt2*-deficient mice support a preferential requirement for AKT2 over AKT1 for insulin-stimulated GLUT4 translocation (23). Thus, AKT2 is described here as a major insulin-responsive node in the studied rodent adipocyte systems, rather than as an unqualified universal determinant of human adipocyte physiology.

TBC1D4 provides a well-supported downstream brake on GLUT4 exocytosis in 3T3-L1 cells: genetic depletion, rescue, GAP-domain mutation, and live trafficking measurements support its role in basal retention and insulin-responsive mobilization (24). These experiments also show that TBC1D4-dependent and TBC1D4-independent routes coexist, so TBC1D4 should not be presented as the sole gatekeeper of vesicle release.

The TC10 (RHOQ) and RAC1 branches should likewise be separated rather than ranked as mutually exclusive tissue-defining pathways. CAP/APS/CBL/CRK/C3G–TC10 signaling and the TC10–CIP4–Gapex-5–RAB31 module were mechanistically characterized mainly in 3T3-L1 adipocytes, often with CHO or COS cells for interaction assays (25–27). RAC1, however, is also required for insulin-stimulated GLUT4 trafficking in 3T3-L1 reporter cells and in adipocyte-specific *Rac1* knockout mice with primary mature white adipocytes (28, 29). Accordingly, **Figure 3** depicts TC10 and RAC1 as context-dependent, potentially complementary regulators of cortical organization and vesicle targeting; it does not designate TC10 as the principal regulator of all adipocytes.

##### 3.5.1.2 GLUT4 storage, vesicle mobilization, and cortical delivery

TUG-dependent processing is supported as one insulin-responsive retention-release mechanism, but its scope must be qualified. In differentiated 3T3-L1 adipocytes, cleavage-resistant TUG impairs insulin-responsive GLUT4 translocation, consistent with release from a Golgi-associated or insulin-responsive intracellular GLUT4 retention compartment (30). USP25M-dependent processing has been demonstrated in 3T3-L1 and primary adipocytes (31). These data do not establish that all GLUT4 storage vesicles arise from the trans-Golgi network, nor that TUG cleavage strictly controls every release event. CAPN10 (calpain-10) evidence in 3T3-L1 cells instead supports a contribution to cortical actin remodeling and GLUT4 translocation (32), not a general claim that it is the protease executing TUG cleavage.

RAB10 is the best-supported adipocyte Rab effector, but its contribution is substantial rather than exclusive. Imaging and perturbation studies in 3T3-L1 adipocytes link RAB10-GTP to exocyst recruitment (33), whereas adipose-specific *Rab10* deletion reduces, but does not abolish, insulin-stimulated GLUT4 translocation in primary adipocytes (34). RAB14, RAB4, and RAB31 have more model-restricted roles in sorting, recycling, or inhibitory retention (27, 35, 36). For motor- and actin-associated nodes, KIF13A has convergent data in 3T3-L1 and human SGBS adipocytes (37), while FAK has pharmacological support in primary mouse and human adipocytes (38). These latter findings support involvement in the corresponding systems but should not be interpreted as definitive causal maps in all human adipose depots.

##### 3.5.1.3 Tethering, docking, and fusion

The exocyst is supported as a tethering module in 3T3-L1 adipocytes (39), with complementary live-cell evidence for EXOC7 (Exo70)-dependent tethering in primary rat adipocytes (40). At the terminal membrane interface, functional 3T3-L1 experiments distinguish the roles of the t-SNAREs STX4 and SNAP23 from that of VAMP2: depletion of STX4 or SNAP23 blocks tethering and fusion, whereas VAMP2 depletion preferentially blocks the subsequent fusion step (41, 42). A direct RAB4–STX4 interaction has also been shown in primary rat adipocytes (35), and mouse/MEF models support a regulatory role of MUNC18C at the STX4 checkpoint (43).

Therefore, SNAP23—not SNAP25—is the functionally supported SNAP isoform in the adipocyte fusion evidence summarized here. SNAP25 has only descriptive expression/localization evidence in human insulin-sensitive tissues and 3T3-L1 cells (44). DOC2B and TPM3.1 provide additional, respectively 3T3-L1- and mouse-supported, modulators of the terminal actin/fusion environment (45, 46). Together, these studies support an overlapping terminal module in experimental adipocyte models; they do not demonstrate a fully conserved human fusion apparatus.

#### 3.5.2 Regulatory networks of GLUT4 trafficking in skeletal muscle

**Figure 4** integrates insulin-responsive and contraction-responsive modules in skeletal muscle as distinct inputs with convergence and potential cross-talk. The strength and provenance of evidence vary from cell-line imaging (principally L6 and C2C12) to isolated and *in vivo* rodent muscle; available human material is comparatively limited and is not used to infer causality.

##### 3.5.2.1 Insulin signaling and contraction-related inputs

For insulin signaling, the consistent formulation is an AKT2-dependent FLJ00068–RAC1 relay, not direct activation of RAC1 by AKT2. In L6 cultures and adult mouse skeletal muscle, FLJ00068 is required downstream of AKT2 for RAC1 activation and GLUT4 translocation (47, 48). Muscle-specific *Rac1* knockout and isolated mouse muscle studies independently support RAC1 in insulin-stimulated surface delivery (49, 50). This supports a rodent muscle mechanism in which AKT2 signals through the FLJ00068 guanine-nucleotide exchange factor to RAC1 and downstream actin remodeling.

Contraction changes cellular energy and Ca2+-related signals and can engage AMPK, CaMKII, and related pathways. AICAR is therefore described here as a pharmacological AMPK-activating agent, or an exercise-related metabolic stimulus, rather than as a direct contraction mimetic. In a mouse imaging model, AICAR- and contraction-stimulated GLUT4 translocation differed in their dependence on AMPKα2 (51). Moreover, electrical-pulse stimulation in C2C12 myotubes identified a CaMKII–Kalirin–RAC1–AKT link (52), and prior AMPK activation can enhance a subsequent insulin response through TBC1D4 in mouse muscle (53, 54). Thus, insulin and contraction supply distinguishable inputs that converge on shared trafficking nodes and can exhibit cross-talk.

##### 3.5.2.2 TBC1D4, TBC1D1, and Rab effectors

TBC1D4 is supported as a convergence node for insulin and exercise-related sensitization in skeletal muscle, including muscle-specific *Tbc1d4* knockout experiments (54). TBC1D1 is more accurately described as a muscle-enriched Rab-GAP and a component of energy-responsive signaling pathways, rather than as a direct cellular energy sensor. Mouse knock-in studies indicate that its Ser231 phosphorylation contributes in a context-dependent manner to AMPK-regulated glucose uptake (55), and *Tbc1d1* knockout rats show impaired contraction-stimulated GLUT4 translocation with preserved total GLUT4 abundance (56). Biochemical and L6 data further show phosphorylation of TBC1D1 by AMPK-activating and insulin-related stimuli (57, 58).

Downstream RAB8A, RAB13, and RAB14 are chiefly supported by L6 or C2C12 perturbation and imaging experiments. RAB8A and RAB13 act at distinct late trafficking steps in L6 cells (59), RAB8A has rescue-supported exocytic evidence in C2C12 cells (60), and RAB14 has functional support in C2C12/L6 systems (61). These findings are retained as mechanistic cell-model evidence; they are not extrapolated as a complete *in vivo* or human skeletal-muscle Rab hierarchy.

##### 3.5.2.3 Cytoskeletal transport, tethering, and membrane fusion

Cortical actin remodeling is supported by perturbation studies in rodent muscle models. ARP2/3–cofilin cycling has Tier 1 support in L6 cells (62), while MYO1C-dependent vesicle immobilization/tethering has been observed in L6 cells and isolated rat flexor digitorum brevis fibers (63). RAB8A–MYO5A (myosin Va) and RAB13–MICAL-L2–ACTN4 modules are likewise defined primarily in L6/C2C12 culture systems (64, 65). These modules should therefore be described as experimentally supported routes within rodent muscle models, not as universally established components of adult human myofibers.

At the terminal step, muscle-specific *Exoc5* deletion provides *in vivo* support for exocyst-dependent GLUT4 exocytosis (13). VAMP2 has functional fusion evidence in L6-GLUT4myc cells (66), and STX4 has transgenic-mouse support (67). The observation that increased MUNC18C selectively suppressed T-tubular, but not sarcolemmal, GLUT4-EGFP translocation was made in one HSA-GLUT4-EGFP transgenic-mouse imaging model (68); it is presented as a model-specific finding, not as a universal mechanism of skeletal muscle. Finally, SNAP25 should not be presented as an established functional component of the skeletal-muscle GLUT4 fusion machinery: its cited evidence is descriptive expression/localization (44), whereas functional SNAP23 data in this review arise from 3T3-L1 adipocytes (41, 42).

### 3.6 Comparison of GLUT4 trafficking mechanisms between skeletal muscle and adipocytes

#### 3.6.1 Shared components, unequal evidence bases

Both tissues contain evidence for an insulin-responsive INSR/PI3K/AKT2-to-Rab-GAP axis and for plasma-membrane tethering/fusion components. However, the apparent overlap is not evidence of identical physiology. In adipocytes, much of the direct pathway architecture derives from 3T3-L1 cells, with selective extension to primary rodent adipocytes, adipocyte-specific knockout models, human SGBS cells, or primary human adipocytes (23, 29, 34, 37, 38). In skeletal muscle, high-resolution evidence is concentrated in L6/C2C12 cultures but is complemented for selected nodes by *in vivo* rodent-muscle genetics (13, 49, 54–56). Consequently, the shared model is best described as an overlapping experimental framework rather than as a uniform mechanism in all adipose and muscle tissues, particularly in humans.

#### 3.6.2 Upstream signal integration and cross-talk

Adipocyte studies emphasize insulin-regulated AKT2/TBC1D4 signaling together with TC10- and RAC1-associated cortical modules. The TC10 circuit is particularly well defined in 3T3-L1 cells (25–27), whereas RAC1 is supported in both 3T3-L1 and adipocyte-specific *Rac1* knockout/primary adipocyte systems (25–29). Skeletal muscle adds contraction- and energy-responsive inputs that converge at TBC1D4, TBC1D1, RAC1, and cytoskeletal trafficking; experimental models identify cross-talk through CaMKII–RAC1–AKT and AMPK-dependent enhancement of subsequent insulin action (52–54).

#### 3.6.3 Rab-GAP, Rab, and actin specialization

TBC1D4 is a shared signaling-to-trafficking regulator, whereas TBC1D1 is more highly represented in skeletal-muscle experiments and participates in context-dependent AMPK/contraction-responsive regulation (54–56). This is tissue enrichment and functional specialization, not proof that TBC1D1 directly senses cellular energy. At the Rab/cytoskeletal level, the strongest adipocyte evidence favors a major but non-exclusive RAB10 contribution, including primary adipocyte/adipose knockout data (33, 34). Skeletal-muscle experiments more often identify RAB8A/RAB13 and the RAC1–actin system, largely in L6/C2C12 or rodent muscle preparations (49, 59, 60, 62). The comparison is therefore evidence-weighted rather than an exclusive assignment of Rab or Rho-family GTPases to one tissue.

#### 3.6.4 Terminal tethering and fusion

The terminal apparatus is partially overlapping but not equally established in the two tissues. In adipocytes, functional 3T3-L1 studies directly support STX4, SNAP23, and VAMP2 with distinguishable tethering and fusion roles (41, 42), with additional primary rodent support for RAB4–STX4 interaction (35). In muscle, exocyst function is supported in muscle-specific *Exoc5* knockout mice (13), while VAMP2, RAB8A/RAB13, and associated actin modules are principally defined in rodent cell models (13, 60, 64, 66, 69). The MUNC18C T-tubule result remains tied to the transgenic-mouse imaging model in which it was observed (68). SNAP25 remains expression-level evidence only (44); it should not be used to claim a functionally established, conserved skeletal-muscle SNARE complex.

#### 3.6.5 Comparative conclusion

Overall, the data support a common logic—signal-dependent release and mobilization of GLUT4 vesicles, cortical targeting, and SNARE/exocyst-associated surface delivery—while the strength, order, and model provenance of individual nodes differ materially between adipocyte and muscle studies. Adipocyte-specific conclusions are strongest for the 3T3-L1/rodent systems in which TC10, RAC1, RAB10, TUG, and SNAP23 were tested; skeletal-muscle conclusions are strongest for the rodent L6/C2C12 and *in vivo* muscle systems in which AKT2–FLJ00068–RAC1, TBC1D1/TBC1D4, RAB8A/RAB13, and EXOC5 were tested. Human translation should therefore be framed as a priority for validation rather than as an already established physiological equivalence.

## 4 Discussion

This systematic review synthesizes current evidence supporting GLUT4 exocytosis as a major effector arm of insulin action through which endocrine signaling is translated into cellular glucose disposal. Across the included literature, insulin-responsive glucose transport was consistently linked not only to proximal receptor signaling, but also to coordinated trafficking events involving vesicle release, mobilization, targeting, docking, membrane fusion, and post-fusion retention (3, 70). Direct imaging and mechanistic studies further demonstrated that insulin acutely promotes vesicle tethering, docking, and fusion at the plasma membrane, while exocyst-dependent targeting is required for efficient transporter insertion (7–9). Collectively, these findings support a model in which the metabolic efficacy of insulin depends on the functional integrity of an integrated signaling–trafficking system rather than on isolated signaling nodes alone.

Importantly, the extracted evidence indicates that GLUT4 trafficking proteins should not be interpreted as functioning within strictly isolated sequential steps. Several regulatory molecules appeared repeatedly across multiple trafficking checkpoints, suggesting that the exocytotic system operates as a highly interconnected and dynamically coordinated network rather than a rigid linear cascade. For example, TBC1D4 participates not only in vesicle release but also indirectly influences vesicle targeting through Rab GTPase regulation, whereas proteins such as MUNC18C, exocyst components, RAC1, and myosin isoforms contribute to overlapping processes including cytoskeletal remodeling, tethering, docking, and fusion. This functional overlap likely increases trafficking efficiency, permits rapid signal amplification, and provides mechanistic flexibility for integrating diverse metabolic stimuli across different cellular contexts.

A central concept emerging from this synthesis is that GLUT4 regulation is best viewed as a modular signaling–trafficking architecture. Canonical insulin signaling through the insulin receptor, insulin receptor substrates, phosphoinositide 3-kinase (PI3K), and AKT establishes a permissive intracellular state for transporter mobilization and surface delivery (2–4, 70, 71). One of the most extensively characterized downstream events is AKT-mediated phosphorylation of TBC1D4, which relieves Rab GTPase suppression and promotes release of GLUT4 storage vesicles from intracellular sequestration (72–74). Experimental studies further indicate that RAB10, together with MYO5A, contributes to directional vesicle translocation toward the cell cortex (75). At the terminal stage, efficient plasma membrane insertion depends on exocyst-mediated targeting (9), MUNC18C-regulated docking control (76), SNAP23 participation in fusion processes (77), SYNIP-dependent syntaxin regulation (78), and broader SNARE network integrity, with increased STX4 expression improving metabolic control in preclinical models (10). This multi-step organization provides a biologically plausible explanation for why impaired glucose uptake may occur despite partial preservation of upstream insulin signaling.

The evolution of experimental methodology has been instrumental in refining this mechanistic framework. The progressive transition from endpoint biochemical assays to real-time visualization platforms is summarized in **Supplementary Table S4**, which highlights how methodological innovation has expanded the resolution of GLUT4 trafficking from bulk redistribution to single-vesicle dynamics. Early biochemical studies using subcellular fractionation, immunoblotting, and radiotracer uptake assays established the principle that insulin redistributes GLUT4 from intracellular stores to the plasma membrane (2, 3, 71). However, these approaches were inherently limited in their ability to distinguish specific exocytic checkpoints. Subsequent use of molecular perturbation systems, fluorescence microscopy, and genetically modified models enabled assignment of discrete proteins to defined trafficking steps (70). More recently, total internal reflection fluorescence microscopy (TIRF), live-cell imaging, and single-vesicle analyses directly visualized vesicle tethering, docking, and fusion in real time (7, 8). These advances shifted the field from a static translocation model toward dynamic regulation of membrane traffic.

These methodological advances also provide important context for interpreting the current molecular landscape of GLUT4 trafficking. The larger number of reported regulators identified in adipocytes compared with skeletal muscle does not necessarily indicate intrinsically greater biological complexity. Instead, this discrepancy may partly reflect differences in experimental accessibility, model availability, and historical research emphasis across the field. Likewise, the predominance of signaling-related molecules in the literature likely reflects the earlier reliance on biochemical, phosphorylation-based, and fractionation approaches, which were more readily suited to investigating upstream signaling cascades than dynamic membrane trafficking events. Only with the later emergence of live-cell imaging, total internal reflection fluorescence microscopy, endogenous protein tagging, and single-vesicle tracking has it become possible to resolve tethering, docking, and fusion with greater spatial and temporal precision. At the same time, it remains plausible that upstream signaling layers are genuinely more molecularly diverse than the relatively conserved downstream fusion machinery. Therefore, the currently available molecular inventory likely reflects both true biological organization and uneven investigational coverage across trafficking stages and metabolic tissues.

Our synthesis also indicates that tissue-specific insulin responses likely arise from differential upstream signal integration acting on partially shared trafficking machinery. In adipocytes, insulin-dependent PI3K–AKT2–TBC1D4 signaling appears tightly coupled to rapid GLUT4 recruitment during the postprandial state (2, 3, 70, 73). Additional pathways involving TC10, cortical actin remodeling, and exocyst-mediated targeting provide spatial control of transporter insertion (9, 79). In skeletal muscle, by contrast, GLUT4 trafficking is regulated within a broader physiological network in which insulin signaling coexists with contraction-sensitive and energy-sensing pathways, particularly AMP-activated protein kinase (AMPK)-dependent mechanisms (6, 11, 80, 81). This dual control is consistent with the observation that exercise-stimulated glucose uptake can remain relatively preserved in some insulin-resistant states. At the organismal level, such tissue-selective regulation may contribute to metabolic flexibility and whole-body glycemic homeostasis.

Taken together, these findings suggest that adipocytes and skeletal muscle employ complementary but physiologically distinct strategies for metabolic regulation. Adipocytes appear optimized for rapid insulin-dependent nutrient storage during the postprandial state, whereas skeletal muscle integrates insulin signaling with contraction- and energy-sensitive pathways to preserve glucose disposal during fluctuating energetic demand. Importantly, both tissues ultimately converge on partially shared Rab- and SNARE-dependent trafficking machinery despite marked divergence in upstream regulatory architecture. This organizational logic may help explain why exercise-mediated glucose uptake can remain relatively preserved in insulin-resistant states and supports the concept that tissue-specific upstream signaling nodes represent potential therapeutic targets distinct from the conserved downstream fusion machinery.

An important translational implication is that defects in signal-to-trafficking coupling represent a complementary mechanistic layer in the pathogenesis of insulin resistance. Rather than serving as an exclusive alternative to receptor-proximal signaling failure, these trafficking defects operate alongside established classical paradigms, including lipotoxicity and ceramide accumulation, chronic tissue inflammation, mitochondrial stress, and reduced overall GLUT4 expression. Within this integrated framework, trafficking defects have been reported at multiple levels, including impaired TBC1D4/Rab regulation (73, 74), altered vesicle traffic and intracellular retention (3, 70), and broader remodeling of insulin signaling networks in insulin-resistant states (4).

As an external translational corroboration to our primary experimental dataset, the mechanistic framework derived from our systematic review finds direct support in independent clinical studies conducted in human metabolic tissues. While human-only studies were excluded from the systematic review process to maintain experimental model consistency, in skeletal-muscle biopsies obtained from individuals with type 2 diabetes during a euglycemic–hyperinsulinemic clamp, Karlsson and colleagues demonstrated that insulin-stimulated phosphorylation of TBC1D4 at Thr642 was reduced by approximately 39% compared with lean control subjects, despite comparable TBC1D4 protein content and only modestly impaired proximal AKT activity — a dissociation consistent with a post-AKT defect in signal-to-trafficking coupling rather than simple upstream signaling failure (82). Extending this observation to the trafficking machinery itself, proteomics of human skeletal muscle from obese individuals with type 2 diabetes revealed coordinated reductions in key endosomal sorting proteins — including SORT1 (−44%), STX16 (−34%), and SNX27 (−60%) — alongside a 30% decrease in total GLUT4 content, suggesting that intracellular compartmental organization of the transporter is broadly disrupted in human disease (83). In adipose tissue, *SLC2A4* mRNA expression in subcutaneous biopsies from individuals with type 2 diabetes is substantially reduced relative to healthy controls and correlates inversely with HOMA-IR, indicating that transporter availability at the source pool is compromised independently of downstream trafficking events (84). Taken together, these human biopsy data provide clinical corroboration that the trafficking checkpoints identified across the included experimental literature — spanning Rab-GAP signaling, vesicle sorting, and transporter pool regulation — are not artifacts of model systems but reflect pathophysiological processes operative in insulin-resistant human metabolic tissues.

The therapeutic implications emerging from GLUT4 trafficking biology extend beyond conventional enhancement of receptor-proximal insulin signaling. Multiple intervention strategies identified across the included literature target distinct trafficking checkpoints capable of improving cellular glucose disposal through partially insulin-independent mechanisms. Exercise and AMPK-activating interventions, for example, stimulate GLUT4 translocation despite impaired canonical insulin signaling, highlighting the therapeutic potential of contraction-responsive pathways in insulin-resistant states (6, 11, 80, 81). Other approaches aim to increase the bioavailable intracellular pool of GLUT4 vesicles through modulation of TUG-dependent retention–release systems (30), whereas enhancement of terminal fusion competence—such as increased STX4 expression—has improved glycemic control in preclinical models (10). Collectively, these findings support the concept that individual components of the trafficking network may serve as mechanistically distinct therapeutic targets. Rather than focusing exclusively on upstream insulin sensitization, emerging strategies may be classified according to the dominant trafficking node they modulate, including vesicle mobilization, cytoskeletal remodeling, tethering, docking, or membrane fusion. Representative pharmacological, exercise-related metabolic stimuli, and vesicle-regulatory approaches derived from GLUT4 trafficking biology are summarized in **Supplementary Table S5**. **Supplementary Table S5** is a hypothesis-generating overview of preclinical observations and does not identify validated, selective GLUT4-trafficking targets or clinical therapeutic candidates.

Several broader principles relevant to molecular endocrinology emerge from the available evidence. First, GLUT4 vesicles should not be regarded as passive carriers, but as specialized endocrine-responsive organelles whose behavior is actively regulated by nutrient and hormonal cues (3, 70). Second, the plasma membrane functions as a regulatory interface rather than merely a destination, integrating tethering complexes, cortical cytoskeletal remodeling, and SNARE activity to determine fusion competence (7–10, 76–78). Third, tissue specificity in insulin action appears to derive primarily from context-dependent coupling between extracellular stimuli and conserved trafficking modules, rather than from entirely distinct terminal machinery in each metabolic tissue (6, 11, 70).

Several limitations should be considered when interpreting this review. First, a substantial proportion of mechanistic evidence derives from immortalized adipocyte cell lines or acute rodent models, which may not fully recapitulate chronic human metabolic disease. Second, insulin concentrations, stimulation duration, and outcome definitions varied considerably across studies, limiting direct cross-study comparability. Third, quantitative assessment of surface GLUT4 was methodologically heterogeneous, ranging from biochemical fractionation to microscopy-based surrogates. Fourth, reporting of randomization, blinding, and cell authentication was frequently incomplete. Finally, publication bias toward positive mechanistic findings may have reduced visibility of neutral or contradictory observations.

Future work should increasingly prioritize physiologically relevant human systems and integrative multi-omic approaches. Combining endogenous protein tagging, genome editing, spatial proteomics, phosphoproteomics, lipidomics, and high-resolution live-cell imaging in primary human adipocytes, skeletal muscle tissue, or *in vivo* models may provide more precise mapping of trafficking checkpoints disrupted by obesity, aging, inflammation, circadian misalignment, and physical inactivity. From a therapeutic perspective, classification of candidate interventions according to their dominant trafficking node of action—vesicle release, mobilization, targeting, docking, or fusion—may facilitate more rational combination strategies for restoring insulin responsiveness.

## 5 Conclusion

This systematic review demonstrates that GLUT4 exocytosis is regulated through an integrated multi-step signaling–trafficking network involving Rab-GAP signaling, cytoskeletal remodeling, vesicle tethering, docking, and SNARE-mediated membrane fusion. Although adipocytes and skeletal muscle share a conserved downstream fusion machinery, tissue-specific upstream signaling architectures generate distinct physiological responses to insulin and energetic demand. The findings further support the concept that the pathogenesis of insulin resistance is highly integrated; it arises not only from impaired receptor signaling, lipotoxicity, and reduced transporter expression, but also incorporates defects in signal-to-trafficking coupling as a critical, complementary mechanistic layer. Collectively, these insights identify GLUT4 trafficking pathways as potential therapeutic targets and provide a mechanistic framework for developing trafficking-directed strategies to improve glucose homeostasis in metabolic disease.

## Data Availability

The original contributions presented in the study are included in the article/**Supplementary Material**. Further inquiries can be directed to the corresponding author.

## References

[1] DeFronzo RA . From the triumvirate to the ominous octet: A new paradigm for the treatment of type 2 diabetes mellitus. Diabetes. (2009) 58:773–95. doi: 10.2337/db09-9028 19336687 PMC2661582

[2] Huang S Czech MP . The Glut4 glucose transporter. Cell Metab. (2007) 5:237–52. doi: 10.1016/j.cmet.2007.03.006 17403369

[3] Bogan J . Regulation of glucose transporter translocation in health and diabetes. Annu Rev Biochem. (2012) 81:507–32. doi: 10.1146/annurev-biochem-060109-094246 22482906

[4] James DE Stöckli J Birnbaum MJ . The aetiology and molecular landscape of insulin resistance. Nat Rev Mol Cell Biol. (2021) 22:751–71. doi: 10.1038/s41580-021-00390-6 34285405

[5] Lu X Xie Q Pan X Zhang R Zhang X Peng G . Type 2 diabetes mellitus in adults: Pathogenesis, prevention and therapy. Signal Transduction Targeted Ther. (2024) 9:262. doi: 10.1038/s41392-024-01951-9 39353925 PMC11445387

[6] Richter EA Hargreaves M . Exercise, Glut4, and skeletal muscle glucose uptake. Physiol Rev. (2013) 93:993–1017. doi: 10.1152/physrev.00038.2012 23899560

[7] Lizunov VA Matsumoto H Zimmerberg J Cushman SW Frolov VA . Insulin stimulates the halting, tethering, and fusion of mobile Glut4 vesicles in rat adipose cells. J Cell Biol. (2005) 169:481–9. doi: 10.1083/jcb.200412069 15866888 PMC2171949

[8] van Gerwen J Shun-Shion AS Fazakerley DJ . Insulin signalling and Glut4 trafficking in insulin resistance. Biochem Soc Trans. (2023) 51:1057–69. doi: 10.1042/bst20221066 37248992 PMC10317183

[9] Inoue M Chang L Hwang J Chiang S-H Saltiel AR . The exocyst complex is required for targeting of Glut4 to the plasma membrane by insulin. Nature. (2003) 422:629–33. doi: 10.1038/nature01533 12687004

[10] Oh E Miller RA Thurmond DC . Syntaxin 4 overexpression ameliorates effects of aging and high-fat diet on glucose control and extends lifespan. Cell Metab. (2015) 22:499–507. doi: 10.1016/j.cmet.2015.07.023 26331606 PMC4560841

[11] Sylow L Kleinert M Richter EA Jensen TE . Exercise-stimulated glucose uptake — regulation and implications for glycaemic control. Nat Rev Endocrinol. (2017) 13:133–48. doi: 10.1038/nrendo.2016.162 27739515

[12] Kjøbsted R Hingst JR Fentz J Foretz M Sanz MN Pehmøller C . Ampk in skeletal muscle function and metabolism. FASEB J. (2018) 32:1741–77. doi: 10.1096/fj.201700442R 29242278 PMC5945561

[13] Fujimoto BA Young M Nakamura N Ha H Carter L Pitts MW . Disrupted glucose homeostasis and skeletal-muscle-specific glucose uptake in an exocyst knockout mouse model. J Biol Chem. (2021) 296:100482. doi: 10.1016/j.jbc.2021.100482 33647317 PMC8027262

[14] Page MJ McKenzie JE Bossuyt PM Boutron I Hoffmann TC Mulrow CD . The Prisma 2020 statement: An updated guideline for reporting systematic reviews. BMJ. (2021) 372:n71. doi: 10.1136/bmj.n71 33782057 PMC8005924

[15] Rethlefsen ML Kirtley S Waffenschmidt S Ayala AP Moher D Page MJ . Prisma-S: An extension to the Prisma statement for reporting literature searches in systematic reviews. Systematic Rev. (2021) 10:39. doi: 10.1186/s13643-020-01542-z 34285662 PMC8270366

[16] The EndNote Team . EndNote. Version 21. Philadelphia, PA: Clarivate (2023).

[17] Ouzzani M Hammady H Fedorowicz Z Elmagarmid A . Rayyan—a web and mobile app for systematic reviews. (2016) 5:210. doi: 10.1186/s13643-016-0384-4 PMC5139140 27919275

[18] Hooijmans CR Rovers MM de Vries RBM Leenaars M Ritskes-Hoitinga M Langendam MW . Syrcle's risk of bias tool for animal studies. BMC Med Res Methodol. (2014) 14:43. doi: 10.1186/1471-2288-14-43 24667063 PMC4230647

[19] Nature Portfolio . Reporting Standards and Availability of Data, Materials, Code and Protocols. Available online at: https://www.nature.com/nature-portfolio/editorial-policies/reporting-standards-and-availability-of-data-materials-code-and-protocols (Accessed April 6, 2026).

[20] OECD . Guidance Document on Good In Vitro Method Practices (GIVIMP). OECD Series on Testing and Assessment, No. 286. (2018). Paris: OECD Publishing. 206 p. doi: 10.1787/9789264304796-en

[21] McGuinness LA Higgins JPT . Risk‐of‐bias visualization (Robvis): An R package and shiny web app for visualizing risk‐of‐bias assessments. Res Synth Methods. (2020) 12:55–61. doi: 10.1002/jrsm.1411 32336025

[22] Yamada E Okada S Saito T Ohshima K Sato M Tsuchiya T . AKT2 phosphorylates Synip to regulate docking and fusion of Glut4-containing vesicles. J Cell Biol. (2005) 168:921–8. doi: 10.1083/jcb.200408182 15753124 PMC2171785

[23] Bae SS Cho H Mu J Birnbaum MJ . Isoform-specific regulation of insulin-dependent glucose uptake by AKT/protein kinase B. J Biol Chem. (2003) 278:49530–6. doi: 10.1074/jbc.M306782200 14522993

[24] Eguez L Lee A Chavez JA Miinea CP Kane S Lienhard GE . Full intracellular retention of GLUT4 requires AS160 Rab GTPase-activating protein. Cell Metab. (2005) 2:263–72. doi: 10.1016/j.cmet.2005.09.005 16213228

[25] Liu J Kimura A Baumann CA Saltiel AR . Aps facilitates C-Cbl tyrosine phosphorylation and Glut4 translocation in response to insulin in 3t3-L1 adipocytes. Mol Cell Biol. (2002) 22:3599–609. doi: 10.1128/mcb.22.11.3599-3609.2002 11997497 PMC133825

[26] Okada S Yamada E Saito T Ohshima K Hashimoto K Yamada M . Cdk5-dependent phosphorylation of the Rho family Gtpase Tc10α regulates insulin-stimulated Glut4 translocation *. J Biol Chem. (2008) 283:35455–63. doi: 10.1074/jbc.M806531200 18948252

[27] Lodhi IJ Chiang SH Chang L Vollenweider D Watson RT Inoue M . Gapex-5, a Rab31 guanine nucleotide exchange factor that regulates Glut4 trafficking in adipocytes. Cell Metab. (2007) 5:59–72. doi: 10.1016/j.cmet.2006.12.006 17189207 PMC1779820

[28] Takenaka N Nakao M Matsui S Satoh T . A crucial role for the small Gtpase Rac1 downstream of the protein kinase AKT2 in insulin signaling that regulates glucose uptake in mouse adipocytes. Int J Mol Sci. (2019) 20:5443. doi: 10.3390/ijms20215443 31683681 PMC6861951

[29] Hasegawa K Takenaka N Tanida K Chan MP Sakata M Aiba A . Atrophy of white adipose tissue accompanied with decreased insulin-stimulated glucose uptake in mice lacking the small Gtpase Rac1 specifically in adipocytes. Int J Mol Sci. (2021) 22:10753. doi: 10.3390/ijms221910753 34639094 PMC8509237

[30] Bogan JS Rubin BR Yu C Löffler MG Orme CM Belman JP . Endoproteolytic cleavage of Tug protein regulates Glut4 glucose transporter translocation. J Biol Chem. (2012) 287:23932–47. doi: 10.1074/jbc.M112.339457 22610098 PMC3390669

[31] Habtemichael EN Li DT Alcázar-Román A Westergaard XO Li M Petersen MC . USP25M protease regulates ubiquitin-like processing of Tug proteins to control Glut4 glucose transporter translocation in adipocytes. J Biol Chem. (2018) 293:10466–86. doi: 10.1074/jbc.RA118.003021 29773651 PMC6036200

[32] Paul DS Harmon AW Winston CP Patel YM . Calpain facilitates Glut4 vesicle translocation during insulin-stimulated glucose uptake in adipocytes. Biochem J. (2003) 376:625–32. doi: 10.1042/bj20030681 12974673 PMC1223814

[33] Sano H Peck GR Blachon S Lienhard GE . A potential link between insulin signaling and Glut4 translocation: Association of Rab10-Gtp with the exocyst subunit Exoc6/6b. Biochem Biophys Res Commun. (2015) 465:601–5. doi: 10.1016/j.bbrc.2015.08.069 26299925 PMC4564306

[34] Vazirani RP Verma A Sadacca LA Buckman MS Picatoste B Beg M . Disruption of adipose Rab10-dependent insulin signaling causes hepatic insulin resistance. Diabetes. (2016) 65:1577–89. doi: 10.2337/db15-1128 27207531 PMC4878419

[35] Li L Omata W Kojima I Shibata H . Direct interaction of rab4 with syntaxin 4*. J Biol Chem. (2001) 276:5265–73. doi: 10.1074/jbc.M003883200 11063739

[36] Sadacca LA Bruno J Wen J Xiong W McGraw TE . Specialized sorting of Glut4 and its recruitment to the cell surface are independently regulated by distinct Rabs. Mol Biol Cell. (2013) 24:2544–57. doi: 10.1091/mbc.E13-02-0103 23804653 PMC3744946

[37] Diaz-Vegas A Norris DM Jall-Rogg S Cooke KC Conway OJ Shun-Shion AS . A high-content endogenous Glut4 trafficking assay reveals new aspects of adipocyte biology. Life Sci Alliance. (2023) 6:e202201585. doi: 10.26508/lsa.202201585 36283703 PMC9595207

[38] Kopietz F Neuhaus M Borreguero-Muñoz A Kryvokhyzha D Stenkula KG . Focal adhesion kinase orchestrates Glut4 translocation and glucose uptake via cytoskeletal turnover in primary adipocytes. FASEB J. (2025) 39:e70660. doi: 10.1096/fj.202402764RR 40396386 PMC12093284

[39] Inoue M Chiang SH Chang L Chen XW Saltiel AR . Compartmentalization of the exocyst complex in lipid rafts controls Glut4 vesicle tethering. Mol Biol Cell. (2006) 17:2303–11. doi: 10.1091/mbc.e06-01-0030 16525015 PMC1446102

[40] Lizunov VA Lisinski I Stenkula K Zimmerberg J Cushman SW . Insulin regulates fusion of Glut4 vesicles independent of Exo70-mediated tethering. J Biol Chem. (2009) 284:7914–9. doi: 10.1074/jbc.M806460200 19155211 PMC2658084

[41] Kawanishi M Tamori Y Okazawa H Araki S Shinoda H Kasuga M . Role of Snap23 in insulin-induced translocation of Glut4 in 3t3-L1 adipocytes: Mediation of complex formation between Syntaxin4 and Vamp2*. J Biol Chem. (2000) 275:8240–7. doi: 10.1074/jbc.275.11.8240 10713150

[42] Kawaguchi T Tamori Y Kanda H Yoshikawa M Tateya S Nishino N . The T-Snares Syntaxin4 and Snap23 but not V-Snare Vamp2 are indispensable to tether Glut4 vesicles at the plasma membrane in adipocyte. Biochem Biophys Res Commun. (2010) 391:1336–41. doi: 10.1016/j.bbrc.2009.12.045 20006577

[43] Kanda H Tamori Y Shinoda H Yoshikawa M Sakaue M Udagawa J . Adipocytes from Munc18c-null mice show increased sensitivity to insulin-stimulated Glut4 externalization. J Clin Invest. (2005) 115:291–301. doi: 10.1172/jci22681 15690082 PMC546422

[44] Jagadish MN Fernandez CS Hewish DR Macaulay SL Gough KH Grusovin J . Insulin-responsive tissues contain the core complex protein Snap-25 (synaptosomal-associated protein 25) a and b isoforms in addition to Syntaxin 4 and Synaptobrevins 1 and 2. Biochem J. (1996) 317:945–54. doi: 10.1042/bj3170945 8760387 PMC1217577

[45] Fukuda N Emoto M Nakamori Y Taguchi A Miyamoto S Uraki S . Doc2b: A novel Syntaxin-4 binding protein mediating insulin-regulated Glut4 vesicle fusion in adipocytes. Diabetes. (2009) 58:377–84. doi: 10.2337/db08-0303 19033398 PMC2628611

[46] Kee AJ Yang L Lucas CA Greenberg MJ Martel N Leong GM . An actin filament population defined by the tropomyosin Tpm3.1 regulates glucose uptake. Traffic. (2015) 16:691–711. doi: 10.1111/tra.12282 25783006 PMC4945106

[47] Ishikura S Bilan PJ Klip A . Rabs 8a and 14 are targets of the insulin-regulated Rab-Gap As160 regulating Glut4 traffic in muscle cells. Biochem Biophys Res Commun. (2007) 353:1074–9. doi: 10.1016/j.bbrc.2006.12.140 17208202

[48] Takenaka N Yasuda N Nihata Y Hosooka T Noguchi T Aiba A . Role of the guanine nucleotide exchange factor in AKT2-mediated plasma membrane translocation of Glut4 in insulin-stimulated skeletal muscle. Cell Signal. (2014) 26:2460–9. doi: 10.1016/j.cellsig.2014.07.002 25025572

[49] Ueda S Kitazawa S Ishida K Nishikawa Y Matsui M Matsumoto H . Crucial role of the small Gtpase Rac1 in insulin-stimulated translocation of glucose transporter 4 to the mouse skeletal muscle sarcolemma. FASEB J. (2010) 24:2254–61. doi: 10.1096/fj.09-137380 20203090 PMC4183928

[50] Takenaka N Araki N Satoh T . Involvement of the protein kinase AKT2 in insulin-stimulated Rac1 activation leading to glucose uptake in mouse skeletal muscle. PloS One. (2019) 14:e0212219. doi: 10.1371/journal.pone.0212219 30735546 PMC6368313

[51] Lauritzen HP Galbo H Toyoda T Goodyear LJ . Kinetics of contraction-induced Glut4 translocation in skeletal muscle fibers from living mice. Diabetes. (2010) 59:2134–44. doi: 10.2337/db10-0233 20622170 PMC2927934

[52] Liu S Zhang J Qi R Deng B Ni Y Zhang C . Camkii and Kalirin, a Rac1-Gef, regulate AKT phosphorylation involved in contraction-induced glucose uptake in skeletal muscle cells. Biochem Biophys Res Commun. (2022) 610:170–5. doi: 10.1016/j.bbrc.2022.03.152 35462099

[53] Kjøbsted R Treebak JT Fentz J Lantier L Viollet B Birk JB . Prior Aicar stimulation increases insulin sensitivity in mouse skeletal muscle in an Ampk-dependent manner. Diabetes. (2014) 64:2042–55. doi: 10.2337/db14-1402 25552597

[54] Kjøbsted R Chadt A Jørgensen NO Kido K Larsen JK de Wendt C . Tbc1d4 is necessary for enhancing muscle insulin sensitivity in response to Aicar and contraction. Diabetes. (2019) 68:1756–66. doi: 10.2337/db18-0769 31175100

[55] Chen Q Xie B Zhu S Rong P Sheng Y Ducommun S . A Tbc1d1 (Ser231ala)-knockin mutation partially impairs Aicar- but not exercise-induced muscle glucose uptake in mice. Diabetologia. (2017) 60:336–45. doi: 10.1007/s00125-016-4151-9 27826658

[56] Whitfield J Paglialunga S Smith BK Miotto PM Simnett G Robson HL . Ablating the protein Tbc1d1 impairs contraction-induced sarcolemmal glucose transporter 4 redistribution but not insulin-mediated responses in rats. J Biol Chem. (2017) 292:16653–64. doi: 10.1074/jbc.M117.806786 28808062 PMC5633127

[57] Chen S Murphy J Toth R Campbell DG Morrice NA Mackintosh C . Complementary regulation of Tbc1d1 and As160 by growth factors, insulin and Ampk activators. Biochem J. (2008) 409:449–59. doi: 10.1042/bj20071114 17995453

[58] Taylor EB An D Kramer HF Yu H Fujii NL Roeckl KSC . Discovery of Tbc1d1 as an insulin-, Aicar-, and contraction-stimulated signaling nexus in mouse skeletal muscle *. J Biol Chem. (2008) 283:9787–96. doi: 10.1074/jbc.M708839200 18276596 PMC2442306

[59] Sun Y Bilan PJ Liu Z Klip A . Rab8a and Rab13 are activated by insulin and regulate Glut4 translocation in muscle cells. Proc Natl Acad Sci. (2010) 107:19909–14. doi: 10.1073/pnas.1009523107 21041651 PMC2993354

[60] Li H Ou L Fan J Xiao M Kuang C Liu X . Rab8a regulates insulin-stimulated Glut4 translocation in C2c12 myoblasts. FEBS Lett. (2017) 591:491–9. doi: 10.1002/1873-3468.12555 28079283

[61] Li Z Yue Y Hu F Zhang C Ma X Li N . Electrical pulse stimulation induces Glut4 translocation in C2c12 myotubes that depends on Rab8a, Rab13, and Rab14. Am J Physiol Endocrinol Metab. (2018) 314:E478–93. doi: 10.1152/ajpendo.00103.2017 29089333

[62] Chiu TT Patel N Shaw AE Bamburg JR Klip A . Arp2/3- and cofilin-coordinated actin dynamics is required for insulin-mediated Glut4 translocation to the surface of muscle cells. Mol Biol Cell. (2010) 21:3529–39. doi: 10.1091/mbc.E10-04-0316 20739464 PMC2954118

[63] Boguslavsky S Chiu T Foley KP Osorio-Fuentealba C Antonescu CN Bayer KU . Myo1c binding to submembrane actin mediates insulin-induced tethering of Glut4 vesicles. Mol Biol Cell. (2012) 23:4065–78. doi: 10.1091/mbc.E12-04-0263 22918957 PMC3469521

[64] Sun Y Jaldin-Fincati J Liu Z Bilan PJ Klip A Brennwald PJ . A complex of Rab13 with Mical-L2 and A-actinin-4 is essential for insulin-dependent Glut4 exocytosis. Mol Biol Cell. (2016) 27:75–89. doi: 10.1091/mbc.E15-05-0319 26538022 PMC4694764

[65] Sun Y Chiu TT Foley KP Bilan PJ Klip A . Myosin Va mediates Rab8a-regulated Glut4 vesicle exocytosis in insulin-stimulated muscle cells. Mol Biol Cell. (2014) 25:1159–70. doi: 10.1091/mbc.E13-08-0493 24478457 PMC3967978

[66] Randhawa VK Bilan PJ Khayat ZA Daneman N Liu Z Ramlal T . Vamp2, but not Vamp3/Cellubrevin, mediates insulin-dependent incorporation of Glut4 into the plasma membrane of L6 myoblasts. Mol Biol Cell. (2000) 11:2403–17. doi: 10.1091/mbc.11.7.2403 10888677 PMC14928

[67] Spurlin BA Park S-Y Nevins AK Kim JK Thurmond DC . Syntaxin 4 transgenic mice exhibit enhanced insulin-mediated glucose uptake in skeletal muscle. Diabetes. (2004) 53:2223–31. doi: 10.2337/diabetes.53.9.2223 15331531

[68] D'Andrea-Merrins M Chang L Lam AD Ernst SA Stuenkel EL . Munc18c interaction with syntaxin 4 monomers and Snare complex intermediates in Glut4 vesicle trafficking. J Biol Chem. (2007) 282:16553–66. doi: 10.1074/jbc.M610818200 17412693

[69] Hodgkinson CP Mander A Sale GJ . Protein kinase-Z interacts with Munc18c: Role in Glut4 trafficking. Diabetologia. (2005) 48:1627–36. doi: 10.1007/s00125-005-1819-y 15986239

[70] Jaldin-Fincati JR Pavarotti M Frendo-Cumbo S Bilan PJ Klip A . Update on Glut4 vesicle traffic: A cornerstone of insulin action. Trends Endocrinol Metabolism: TEM. (2017) 28:597–611. doi: 10.1016/j.tem.2017.05.002 28602209

[71] Watson RT Pessin JE . Intracellular organization of insulin signaling and Glut4 translocation. Recent Prog Horm Res. (2001) 56:175–93. doi: 10.1210/rp.56.1.175 11237212

[72] Kane S Sano H Liu SCH Asara JM Lane WS Garner CC . A method to identify serine kinase substrates: AKT phosphorylates a novel adipocyte protein with a Rab Gtpase-activating protein (Gap) domain *. J Biol Chem. (2002) 277:22115–8. doi: 10.1074/jbc.C200198200 11994271

[73] Sakamoto K Holman GD . Emerging role for As160/Tbc1d4 and Tbc1d1 in the regulation of Glut4 traffic. Am J Physiol Endocrinol Metab. (2008) 295:E29–37. doi: 10.1152/ajpendo.90331.2008 18477703 PMC2493596

[74] Sano H Kane S Sano E Mıinea CP Asara JM Lane WS . Insulin-stimulated phosphorylation of a Rab Gtpase-activating protein regulates Glut4 translocation *. J Biol Chem. (2003) 278:14599–602. doi: 10.1074/jbc.C300063200 12637568

[75] Chen Y Wang Y Zhang J Deng Y Jiang L Song E . Rab10 and myosin-Va mediate insulin-stimulated Glut4 storage vesicle translocation in adipocytes. J Cell Biol. (2012) 198:545–60. doi: 10.1083/jcb.201111091 22908308 PMC3514028

[76] Thurmond DC Ceresa BP Okada S Elmendorf JS Coker K Pessin JE . Regulation of insulin-stimulated Glut4 translocation by Munc18c in 3t3l1 adipocytes*. J Biol Chem. (1998) 273:33876–83. doi: 10.1074/jbc.273.50.33876 9837979

[77] Foster LJ Yaworsky K Trimble WS Klip A . Snap23 promotes insulin-dependent glucose uptake in 3t3-L1 adipocytes: Possible interaction with cytoskeleton. Am J Physiol Cell Physiol. (1999) 276:C1108–14. doi: 10.1152/ajpcell.1999.276.5.C1108 10329959

[78] Min J Okada S Kanzaki M Elmendorf JS Coker KJ Ceresa BP . Synip: A novel insulin-regulated syntaxin 4–binding protein mediating Glut4 translocation in adipocytes. Mol Cell. (1999) 3:751–60. doi: 10.1016/S1097-2765(01)80007-1 10394363

[79] Watson RT Shigematsu S Chiang S-H Mora S Kanzaki M Macara IG . Lipid raft microdomain compartmentalization of TC10 is required for insulin signaling and GLUT4 translocation. J Cell Biol. (2001) 154:829–40. doi: 10.1083/jcb.200102078 PMC2196453 11502760

[80] O'Neill HM . AMPK and exercise: glucose uptake and insulin sensitivity. Diabetes Metab J. (2013) 37:1–21. doi: 10.4093/dmj.2013.37.1.1 PMC3579147 23441028

[81] Merrill GF Kurth EJ Hardie DG Winder WW . Aica riboside increases Amp-activated protein kinase, fatty acid oxidation, and glucose uptake in rat muscle. Am J Physiol. (1997) 273:E1107–12. doi: 10.1152/ajpendo.1997.273.6.E1107 9435525

[82] Karlsson H Zierath JR Kane S Krook A Lienhard GE Wallberg-Henriksson H . Insulin-stimulated phosphorylation of the AKT substrate As160 is impaired in skeletal muscle of type 2 diabetic subjects. Diabetes. (2005) 54:1692–7. doi: 10.2337/diabetes.54.6.1692 15919790

[83] Livingstone R Bryant NJ Boyle JG Petrie JR Gould GW . Diabetes is accompanied by changes in the levels of proteins involved in endosomal Glut4 trafficking in obese human skeletal muscle. Endocrinol Diabetes Metab. (2022) 5:e361. doi: 10.1002/edm2.361 35964329 PMC9471587

[84] Kouidhi S Berrhouma R Rouissi K Jarboui S Clerget-Froidevaux MS Seugnet I . Human subcutaneous adipose tissue Glut 4 Mrna expression in obesity and type 2 diabetes. Acta Diabetol. (2013) 50:227–32. doi: 10.1007/s00592-011-0295-8 21604201

