# Supplementary material for: Molecular architecture of GLUT4 trafficking and exocytosis in adipocytes and skeletal muscle: A systematic review and integrated conceptual framework
Source: Front Endocrinol (Lausanne). 2026 Oct 19;17:1932455. doi: 10.3389/fendo.2026.1932455 (PMC13725575; doi:10.3389/fendo.2026.1932455)
Supplement: Supplementary file 1 [file Table1.pdf]

										Outcome					SYRCLE's Risk of Bias tool for animal studies (low risk/ unclear risk/ high risk)									Methodological rigor assessment for in vitro studies using a custom checklist (low risk/ unclear risk/ high risk)					
No.	DOI	First Author, Year	Country	Aim	Population	Intervention/Exposure	Assays	Time Points	Comparator	Key findings	Key Molecule	Effect (Inhibition/Stimulation)	Novel Mechanism	phase	Sequence generation (Selection Bias)	Baseline characteristics (Selection Bias)	Allocation concealment (Selection Bias)	Random housing (Performance Bias)	Blinding of caregivers (Performance Bias)	Random outcome assessment (Detection Bias)	Blinding of assessor (Detection Bias)	Incomplete outcome data (Attrition Bias)	Selective reporting (Reporting Bias)	Cell line authentication	Blinding of experimenters	Internal controls	Randomization of culture plates	Blinding of analysis	Statistical analysis appropriateness
A.1	10.2337/diabetes.49.10.1649	Ian P. Sal, 2000	UK	The main aim was to explore the mechanisms of action of AICAR and AMPK, which may provide insights into the mechanisms of insulin action	3T3-L1 adipocytes	• Incubation with 5-aminoimidazole-4-carboxamide ribonucleoside (AICAR)	• Plasma membrane lawn assay	• 60 min	Control under conditions without AICAR (vehicle control).	AICAR stimulated 2-deoxy-D-glucose transport twofold and reduced insulin-stimulated glucose uptake to 62% of the control transport rate, in a dose-dependent manner and closely correlated with AMPK Stimulation	AMPK	Inhibition	In adipocytes, Stimulation of AMPK by AICAR inhibits insulin-stimulated glucose transport by blocking GLUT4 (and IRAP) translocation. This mechanism occurs downstream of PI3K/PKB and is independent of the adenosine receptor or nitric oxide (NO). This contrasts with the mechanism in skeletal muscle (where AMPK stimulates glucose transport).	Signaling	Unclear risk (Not reported)	Low risk	Unclear risk (Not reported)	Unclear risk (Not reported)	Unclear risk (Not reported)	Unclear risk (Not reported)	Unclear risk (Not reported)	Low risk	Low risk	Unclear risk (Not reported)	Unclear risk (Not reported)	Low risk	Unclear risk (Not reported)	Unclear risk (Not reported)	Low risk
A.2	10.2337/diabetes.48.8.1667	Kurth-Kraczek et al., 1999	United States	To determine whether Stimulation of AMPK by AICA-riboside increases glucose uptake through translocation of GLUT4 from intracellular compartments to the plasma membrane	Male Sprague-Dawley rats	• Perfusion with AICA-riboside (AICAR). Concentration: 2 mmol/l.	• Measurement of glucose uptake using arteriovenous difference.<br>• Western blot analysis to quantify GLUT4	• Perfusate samples were collected at 0, 15, 30, and 45 min after perfusion	• Control with insulin treatment alone (10 nmol/l insulin) to assess the effect of AICAR on insulin-stimulated glucose transport	AICAR also inhibited insulin-stimulated GLUT4 translocation, as assessed by the plasma membrane lawn assay	AMPK	Inhibition	Unlike skeletal muscle, where AMPK Stimulation (by AICAR) promotes glucose transport to supply ATP as fuel, in adipocytes, AMPK Stimulation inhibits insulin-stimulated glucose transport through downstream PI3K signaling	Signaling	Unclear risk (Not reported)	Low risk	Unclear risk (Not reported)	Unclear risk (Not reported)	Unclear risk (Not reported)	Unclear risk (Not reported)	Unclear risk (Not reported)	Low risk	Low risk						
A.4	10.1074/jbc.M702472200	Hakuno et al., 2007	Japan	To identify proteins that interact with IRS-1 and regulate IRS-mediated signaling	• COS-7 cells (monkey kidney cells).<br>• 3T3-L1 adipocytes (mouse adipocytes).<br>• CHO-C400 cells (Chinese hamster ovary cells expressing the human insulin receptor).	• Transfection or electroporation of plasmids expressing 53BP2S<br>• Stimulation: Insulin (concentration 100 nM).<br>• Inhibition: LY294002 (PI 3-kinase inhibitor, 100 µM).	• Yeast two-hybrid screening<br>• GST pulldown assay<br>• Co-immunoprecipitation (Co-IP)<br>• Western blot (Immunoblotting)<br>• RT-PCR<br>• Immunofluorescence & Confocal microscopy	• Signaling: 2 min, 5 min, 10 min, 30 min, 60 min after insulin stimulation.<br>• Translocation: 20 min after insulin stimulation.	• Control plasmids: GST alone, pEGFP vector, pFLAG vector.<br>• Basal conditions<br>• Preimmune serum.	53BP2S caused uncoupling between Akt and Glut4 by inhibiting AS160 phosphorylation, leading to reduced glucose transport despite robust Akt Stimulation	53BP2S	Inhibition	53BP2S directly binds to the PTB domain and the central region (aa 750–861) of IRS-1 via its SH3 domain and ankyrin repeats.	Signaling										Unclear risk (Not reported)	Unclear risk (Not reported)	Low risk	Unclear risk (Not reported)	Unclear risk (Not reported)	Low risk
A.6	10.1091/mbc.E15-05-0319	Sun et al., 2016	Canada	To determine the role of MICAL-L2 as an effector of Rab13 in insulin-stimulated GLUT4 translocation and to elucidate the interaction mechanism among Rab13, MICAL-L2, and ACTN4	• Mouse skeletal muscle L6 cells<br>• CHO-IR cells	• Stimulation with insulin (100 nM).<br>• Transfection of vectors: GFP-MICAL-L2, mCherry-Rab13, GFP-Rab13.<br>• Gene knockdown using shRNA targeting MICAL-L2.<br>• Expression of truncated MICAL-L2-CT (containing only the Rab-binding region, lacking the actin/ACTN4-binding region) and MICAL-L2-ACT	• Spinning-disk confocal fluorescence microscopy<br>• Structured illumination microscopy – SIM<br>• Total internal reflection fluorescence microscopy – TIRF<br>• Pull-down assays and co-immunoprecipitation).	• Assessment performed 24–48 h after transfection	• Basal group<br>• Group expressing inactive Rab13<br>• Vector control group (GFP, unrelated shRNA)	MICAL-L2 is an effector of insulin-activated Rab13, links to GLUT4 through ACTN4, and localizes GLUT4 vesicles at the muscle cell periphery to enable their fusion with the membrane	MICAL-L2	Stimulation	MICAL-L2 functions as an effector of insulin-activated Rab13. Insulin stimulates Rab13 binding to MICAL-L2, exposing the ACTN4-binding site on MICAL-L2. This complex (Rab13–MICAL-L2–ACTN4) acts to anchor and localize GLUT4 vesicles at the cell periphery (via ACTN4–GLUT4 interaction) in preparation for membrane fusion.	Docking										Unclear risk (Not reported)	Unclear risk (Not reported)	Low risk	Unclear risk (Not reported)	Unclear risk (Not reported)	Low risk
A.7	10.1111/febs.12719	Takenaka et al., 2014	Japan	To clarify the functional link between Akt2 and Rac1 in insulin signaling in skeletal muscle and to identify the downstream role of Rac1 relative to Akt2	• Mouse skeletal muscle L6 cells with a C57BL/6 genetic background	• Expression of constitutively active Akt2 (Myr-Akt2) and constitutively active PtdIns3K (Myr–p110α)<br>• Gene knockdown using siRNA (Rac1, FLJ00068, Akt2).<br>• Insulin stimulation (100 nM).<br>• In vivo gene transfer into the gastrocnemius muscle by electroporation.	• Immunoblot (Western blot) analysis.<br>• Measurement of GLUT4 translocation by fluorescence/confocal microscopy.<br>• Pull-down assay<br>• RT-PCR	• Mice were killed 5 days after gene transfer, following a 16-h fast.	• Control siRNA group,<br>• Control plasmids<br>• Wild-type mice	Rac1 is a critical component of the Akt2 pathway in mouse skeletal muscle as well as in cultured muscle cells	Akt2<br>Rac1	Stimulation	Akt2 activates Rac1 through the guanine nucleotide exchange factor (GEF) FLJ00068. Rac1 plays an essential downstream role of Akt2 in regulating insulin-stimulated GLUT4 translocation in skeletal muscle.	Signaling	Unclear risk (Not reported)	Low risk	Unclear risk (Not reported)	Unclear risk (Not reported)	Unclear risk (Not reported)	Unclear risk (Not reported)	Unclear risk (Not reported)	Low risk	Low risk	Unclear risk (Not reported)	Unclear risk (Not reported)	Low risk	Unclear risk (Not reported)	Unclear risk (Not reported)	Low risk
A.8	10.3390/ijms20215443	Takenaka et al., 2019	Japan	To examine whether Rac1 is involved in insulin-dependent signaling to regulate glucose uptake in adipocytes	• L1-GLUT4 cell line (a stable cell line expressing GLUT4myc7-GFP) differentiated from 3T3-L1 fibroblasts	• Insulin stimulation (100 nM).<br>• Use of specific inhibitors: RI-II (Rac1 inhibitor), Wortmannin (PI3K inhibitor), AI-XII (Akt2 inhibitor).<br>• Overexpression of constitutively activated mutants: Myr-p110α (PI3K), Myr-Akt2, Rac1(G12V).<br>• Gene knockdown of RalA using shRNA	• Quantification of GLUT4 translocation by confocal laser scanning microscopy (using the GLUT4myc7-GFP reporter).<br>• Measurement of 2-deoxy-D-glucose uptake (2-DG uptake assay).<br>• Detection of active Rac1 and RalA (Rac1-GTP, RalA-GTP) by immunofluorescent microscopy with specific polypeptide probes.	• Insulin stimulation: 30 min.<br>• Inhibitor treatment: during the last 1.5 to 2 h of serum starvation.<br>• Glucose uptake measurement: incubation for 15 min	• Control group (Control/–) without treatment or infected with empty virus<br>• Group without inhibitors.	Rac1 is a critical link in insulin-dependent signaling, receiving signals from Akt2 and transmitting them downstream to RalA to regulate GLUT4 translocation	Rac1	Stimulation	In adipocytes, Rac1 functions downstream of Akt2 and upstream of RalA to regulate insulin-dependent GLUT4 translocation.	Signaling										Unclear risk (Not reported)	Unclear risk (Not reported)	Low risk	Unclear risk (Not reported)	Unclear risk (Not reported)	Low risk
A.9	10.1034/j.1600-0854.2000.010609.x	Foster et al., 2000	Canada	To investigate the distribution and function of VAP-33 in muscle and adipocyte cell lines, specifically its spatial and functional relationship with VAMP-2 and its role in insulin-stimulated GLUT4 transport	• Rat skeletal muscle cells (L6 skeletal myoblasts) stably expressing myc-tagged GLUT4 (L6-GLUT4myc).<br>• Mouse adipose cells (3T3-L1 adipocytes).	• Insulin stimulation (100 nM).<br>• Overexpression of wild-type (full-length) VAP-33.<br>• Microinjection of anti–VAP-33 antibodies.<br>• Co-overexpression of VAMP-2-GFP to rescue the inhibitory effect	• Indirect confocal immunofluorescence.<br>• Subcellular fractionation and immunoblotting (Western blot).<br>• Single-cell GLUT4myc translocation assay (measurement of cell-surface GLUT4myc).<br>• Plasma membrane lawn assay.<br>• Immunoadsorption.	• After transfection: 48 h.<br>• After microinjection: 90 min incubation before stimulation.<br>• Insulin treatment time: 20 min (L6) or 15 min (3T3-L1).	• Untransfected cells.<br>• Cells transfected with empty vector (pcDNA3.1) and GFP.<br>• Non-injected cells.<br>• Cells injected with irrelevant IgG.<br>• Basal (no insulin).	VAP-33 inhibits the ability of VAMP-2 to participate in the SNARE complex, thereby inhibiting fusion of GLUT4 vesicles and other vesicle fusion events	VAP-33	Inhibition	VAP-33 functions as a regulator of VAMP-2 availability. Excess VAP-33 or antibody-mediated sequestration of VAP-33 prevents VAMP-2 from participating in the SNARE complex required for GLUT4 vesicle fusion.	Fusion										Unclear risk (Not reported)	Unclear risk (Not reported)	Low risk	Unclear risk (Not reported)	Unclear risk (Not reported)	Low risk
A.10	10.26508/lsa.202201585	Diaz-Vegas et al., 2022	Australia	To develop and validate a high-content imaging platform to study endogenous GLUT4 trafficking in intact adipocytes, in order to identify novel regulatory factors and to compare with methods using overexpressed GLUT4	• Mouse 3T3-L1 adipocytes (differentiated 3T3-L1 adipocytes) and human SGBS adipocytes.<br>• In vitro model: adipocytes differentiated from fibroblasts	• Insulin stimulation (various concentrations: 0.5 nM, 1 nM, 100 nM).<br>• Insulin resistance–inducing treatments: Chronic Insulin (CI), TNF-α, Dexamethasone (DEX).<br>• Gene knockdown using siRNA (Slc2a4/Glut4, Irs1/2, Rab10, Kif13A, Tbc1d4, Trarg1, Stx6, Stx16, Vps45, Cltc, Rab14, Bcl9l).<br>• Use of signaling inhibitors: GDC0941 (PI3K inhibitor), GSK23344 (PDPK1 inhibitor), GDC0068 (Akt inhibitor), MK2206 (Akt inhibitor).<br>• Overexpression of HA-GLUT4-mRuby3 for comparison.	• High-content imaging using confocal microscopy.<br>• Immunofluorescence with antibodies against the exofacial epitope of GLUT4 (LM048, •LM052, LM059).<br>• Digital phase contrast microscopy (to measure lipid droplets).<br>• Western blot.<br>• Glucose uptake assay (using 2-[3H] deoxyglucose).<br>• Immunoprecipitation–mass spectrometry (IP-MS).	• Insulin stimulation: 20 min.<br>• Insulin resistance models: CI (24 h), TNF (4 days or 24 h), DEX (8 days).<br>• After siRNA transfection: cells were used after differentiation and treatment (the specific procedure extended over multiple days, with assessment after stimulation).	• Basal (unstimulated cells).<br>• Non-targeting siRNA (NT) control.<br>• Vehicle control (DMSO, Ethanol).<br>• Wild-type cells vs HA-GLUT4-mRuby3 expressing cells.<br>• Control (untreated) cells in insulin resistance experiments.	Vps45 and Rab14 are selective regulators of GLUT4, whereas knockdown of Trarg1, Stx6, Stx16, Tbc1d4, and Rab10 affected translocation of both GLUT4 and TfR. Thus, the translocation machinery of GLUT4 and TfR likely has some overlap upon insulin stimulation. In addition, we identified Kif13A—a Rab10-binding molecular motor—as a novel regulator of GLUT4 trafficking.	Kif13A	Stimulation	Identification of Kif13A (a kinesin motor protein binding to Rab10) as a novel regulator of endogenous GLUT4 trafficking. Discovery that Trarg1 regulates both GLUT4, TfR, and lipid droplet morphology, and interacts with Bcl9l to execute this function.	translocation										Low risk	Unclear risk (Not reported)	Low risk	Unclear risk (Not reported)	Unclear risk (Not reported)	Low risk
A.12	10.1016/j.cmet.2009.09.012	Ho et al., 2009	United States	The aim of the study was to explore the function of RIP140 in the cytoplasm of adipocytes, specifically its negative regulatory role in insulin-stimulated GLUT4 trafficking and the associated molecular mechanism.	• 3T3-L1 adipocytes (differentiated for 4 days and 8 days).<br>• G7 adipocytes (stably expressing myc-GLUT4-EGFP).<br>• Primary adipocytes from the epididymal fat pads of male mice (epididymal adipocytes).<br>• RIP140-null adipocytes.	• Insulin stimulation (100 nM or 170 nM).<br>• Gene knockdown (siRNA) targeting RIP140 and PKC-ε.<br>• Rescue expression of RIP140 variants (WT, CP – phosphomimetic, CN – phosphor-deficient) in RIP140-null cells.<br>• High-fat diet (HFD) for 5 weeks compared with normal diet (ND).	• Glucose uptake assay (measurement of 2-deoxyglucose uptake).<br>• Subcellular fractionation (PM, LDM, nucleus, cytoplasm) combined with Western blot.<br>• Immunofluorescence microscopy (observation of myc-GLUT4-EGFP rim).<br>• Co-immunoprecipitation (Co-IP) and GST pull-down assay (protein–protein interaction).<br>• In vitro kinase assay (measurement of Akt kinase activity toward AS160).	• Insulin stimulation: 20 min.<br>• Knockdown: performed on day 5, measurement on day 8 of differentiation.<br>• Mouse model: after 5 weeks of dietary intervention	• Control siRNA (Ctrl).<br>• Basal (no insulin).<br>• Mice fed a normal diet (Normal Diet – ND).<br>• RIP140-null cells transfected with empty vector.	RIP140 is an inhibitor of GLUT4 trafficking and glucose uptake	RIP140	Inhibition	Identification of a novel cytoplasmic function of RIP140: RIP140 directly interacts with AS160, preventing Akt-mediated phosphorylation of AS160 (maintaining AS160 in its active state), thereby inhibiting GLUT4 trafficking. This mechanism is initiated by PKC-ε–mediated phosphorylation of RIP140 in the nucleus, which promotes its export to the cytoplasm.	Signaling	Unclear risk (Not reported)	Low risk	Unclear risk (Not reported)	Unclear risk (Not reported)	Unclear risk (Not reported)	Unclear risk (Not reported)	Unclear risk (Not reported)	Low risk	Low risk	Unclear risk (Not reported)	Unclear risk (Not reported)	Low risk	Unclear risk (Not reported)	Unclear risk (Not reported)	Low risk
A.13	10.2337/db08-0312	Fukuda et al., 2008	Japan	The aim was to elucidate positive regulators of SNARE complex assembly involved in GLUT4 vesicle fusion, specifically to investigate the role of DOC2b in adipocytes.	• Mouse 3T3-L1 adipocyte	• Insulin stimulation (100 nM).<br>• Overexpression of wild-type DOC2b and calcium-interaction–deficient mutants.<br>• Gene knockdown of DOC2b using shRNA.<br>• Use of an intracellular calcium chelator (BAPTA-AM) to reduce Ca2+.	• Confocal immunofluorescence microscopy.<br>• In vitro GST pull-down assay.<br>• Immunoprecipitation.<br>• 2-deoxyglucose uptake assay	• Insulin stimulation: 10–20 min.	• Control (no insulin treatment).<br>• Cells expressing eGFP alone or empty vector.<br>• Cells expressing non-specific control shRNA.	DOC2b induces fusion of GLUT4-containing vesicles.	DOC2b (Double C2 domain protein beta)	Stimulation	DOC2b functions as a calcium (Ca2+) sensor and a positive regulator of GLUT4 vesicle fusion. Upon insulin stimulation, DOC2b translocates to the plasma membrane and directly interacts with Syntaxin4 in a Ca2+-dependent manner, thereby promoting SNARE complex formation.	Fusion										Unclear risk (Not reported)	Unclear risk (Not reported)	Low risk	Unclear risk (Not reported)	Unclear risk (Not reported)	Low risk
A.16	10.1038/srep35438	Liu et al., 2016	Japan	To identify IRS-1–binding proteins and examine their roles (specifically DGK-ζ) in GLUT4 translocation to the plasma membrane.	• 3T3-L1 adipocytes	• Insulin stimulation (100 nM).<br>• Gene knockdown using siRNA (DGK-ζ, PIP5K1-α).<br>• Overexpression of plasmids: FLAG-DGK-ζ, HA-PIP5K1-ζ, GFP-sDGK-ζ (short form).	• Yeast two-hybrid screening.<br>• Co-immunoprecipitation.<br>• Immunoblotting (Western blot).<br>• GLUT4 translocation assay.<br>• Glucose uptake assay.<br>• DGK activity assay & PIP5K activity assay (enzyme activity measurement).	• Insulin stimulation: 20 min (for translocation and glucose uptake), or 60 min (for protein interaction).<br>• After electroporation/transfection: 24 h or 3 days.	• Control siRNA (non-targeting control siRNA).<br>• Mock vector (empty vector).<br>• TB Basal (basal condition without insulin).	In the absence of insulin, DGK-ζ binds to IRS-1 and prevents GLUT4 translocation. Upon insulin stimulation, DGK-ζ dissociates and enhances GLUT4 translocation.	DGK-ζ	Inhibition	In the basal state (no insulin): DGK-ζ associates with IRS-1 and prevents GLUT4 translocation.<br>Upon insulin stimulation: the IRS-1–DGK-ζ complex dissociates. Free DGK-ζ activates PIP5K1-α (generating PIP2), thereby promoting GLUT4 translocation through a PI3K-independent pathway (a non-canonical pathway, potentially involving actin remodeling).	Signaling										Unclear risk (Not reported)	Unclear risk (Not reported)	Low risk	Unclear risk (Not reported)	Unclear risk (Not reported)	Low risk
A.19	10.1074/jbc.M110.146886	Zhou et al., 2010	United States	The aim of the study was to search for novel proteins that enhance insulin signaling to Akt and GLUT4 translocation, specifically to identify the role of the protein PHLDB1.	• Mouse 3T3-L1 adipocyte	• Insulin stimulation (1 nM or 100 nM).<br>• Gene knockdown using siRNA (PHLDB1, PHLDB2, Akt).<br>• Overexpression via adenovirus or electroporation: EGFP-PHLDB1, HA-PHLDB1.<br>• Use of inhibitor: Wortmannin (PI3K inhibitor).	• Western blot (immunoblotting).<br>• TIRF microscopy (total internal reflection fluorescence microscopy).<br>• Deoxyglucose uptake assay.<br>• Affymetrix GeneChip analysis & quantitative real-time PCR (gene expression measurement).<br>• Lipid binding assay (phosphoinositide binding assay).	• Insulin stimulation: 15 min (for immunoblotting), 30 min (for glucose uptake).<br>• After siRNA transfection: 72 h.<br>• After adenovirus infection: 48 h	• Scrambled siRNA (Scr) / control siRNA.<br>• Basal / control (no insulin stimulation).<br>• Mock vector / HA control virus (empty vector / control virus).	PHLDB1 is a novel positive regulator of Akt.	PHLDB1	Stimulation	PHLDB1 is a novel positive regulator of Akt. PHLDB1 contains a specific phosphoinositide-binding PH domain. Upon insulin stimulation, PHLDB1 translocates from the cytoplasm to the plasma membrane (together with Akt), where it facilitates Akt Stimulation and downstream signaling (such as p70S6K and GLUT4), independently of IRS-1 phosphorylation.	Signaling										Unclear risk (Not reported)	Unclear risk (Not reported)	Low risk	Unclear risk (Not reported)	Unclear risk (Not reported)	Low risk
A.20	10.1016/j.bbrc.2005.04.082	Steimle et al., 2005	United States	The aim of the study was to explore the role of myosin II in regulating dynamic cellular processes that promote insulin-stimulated glucose uptake in adipocytes.	• Mouse 3T3-L1 adipocyte	• Insulin stimulation (1 nM or 100 nM).<br>• Pharmacological inhibition: Blebbistatin (100 µM) – a specific inhibitor of nonmuscle myosin II.	• Glucose uptake assay (using [14C]2-deoxy-D-glucose).<br>• Immunoblot analysis (Western blot).<br>• Immunofluorescence confocal microscopy.<br>• Plasma lawn assay	• 4 h	• Basal (no insulin).<br>• Vehicle control (DMSO).	Myosin II is activated by insulin signaling and stimulates fusion of GLUT4 transport vesicles.	Myosin II (Myosin IIA, Myosin IIB).	Stimulation	Myosin II functions as an essential component for insulin-stimulated glucose uptake but does not participate in GLUT4 translocation. Instead, myosin II (particularly activated Myosin IIA recruited to the membrane) may play a role in cortical actin contraction to facilitate docking and/or fusion of GLUT4 vesicles with the plasma membrane. This represents the first evidence that actin contraction by myosin II is required for glucose uptake, in addition to actin remodeling.	Fusion<br>Docking										Unclear risk (Not reported)	Unclear risk (Not reported)	Low risk	Unclear risk (Not reported)	Unclear risk (Not reported)	Low risk
A.24	10.1016/j.bbrc.2015.08.069	Sano et al., 2015	United States	The aim of the study was to identify proteins interacting with Rab10 in its GTP-bound form in adipocytes and to examine whether these interactions link insulin signaling to the exocytic machinery during GLUT4 translocation, specifically investigating the roles of Exoc6 and Exoc6b.	• Mouse 3T3-L1 adipocyte	• Stimulation: Insulin (160 nM).<br>• Genetic intervention: siRNA knockdown (Exoc6, Exoc6b), expression of Rab10 mutants (Rab10 Q68L – GTP-bound form, Rab10 T23N – GDP-bound form).	• Yeast two-hybrid analyses.<br>• Flow cytometry.<br>• Immunofluorescence.<br>• Immunoblotting (Western blot).	• 3 days (after siRNA transfection).<br>• 30 min (after insulin treatment).	• Control siRNA.<br>• Basal (no insulin treatment).<br>• Wild-type Rab10.<br>• Rab10 T23N (inactive form).	The association between Rab10-GTP and Exoc6/6b is a molecular link connecting insulin signaling with the exocytic machinery during GLUT4 transport	• Rab10<br>• Exocyst subunits (Exoc6, Exoc6b).	Stimulation	Identification that Rab10 (in its GTP-bound form) directly interacts with the exocyst subunits Exoc6 and Exoc6b, serving as a molecular bridge to recruit the exocyst complex to GLUT4 vesicles.	Translocation										Unclear risk (Not reported)	Unclear risk (Not reported)	Low risk	Unclear risk (Not reported)	Unclear risk (Not reported)	Low risk
A.25	10.1111/j.1742-4658.2007.06232.x	Chen et al., 2008	China	To investigate intracellular trafficking of GLUT4-containing vesicles (GSVs) and the kinetics of docking at the plasma membrane under insulin stimulation under normal conditions and when microtubules are disrupted.	• Mouse 3T3-L1 adipocyte	• Stimulation: Insulin (100 nM). 1313<br>• Chemical inhibitor: Nocodazole (33 µM) to disrupt microtubules.	• Total Internal Reflection Fluorescence Microscopy (TIRFM).<br>• 2-deoxyglucose uptake assay	• 30 min (microscopy observation after insulin addition).<br>• 5 min (glucose uptake assay).	• Control group (no nocodazole treatment).<br>• Basal.	Transport of GLUT4-containing vesicles to the plasma membrane occurs along microtubules, and this transport is obligatory in insulin signaling to GLUT4.	• Microtubules	Stimulation	Identification of a "pre-docking" role of microtubules: microtubules are required to transport GSVs from intracellular compartments to the sub–plasma membrane region (recruitment). In the absence of microtubules, GSVs cannot reach the sites required for docking.	Translocation										Unclear risk (Not reported)	Unclear risk (Not reported)	Low risk	Unclear risk (Not reported)	Unclear risk (Not reported)	Low risk
A.26	10.1091/mbc.E14-06-1060	Karunanithi et al., 2014	United States	To identify Rab10 as a functional target of AS160 and to elucidate the relationship between Rab10 and RalA in GLUT4 trafficking, specifically to test the hypothesis that Rab10 activates RalA through recruitment of the guanine nucleotide exchange factor (GEF) Rlf.	• 3T3-L1 adipocytes<br>• COS-1 cells<br>• 293T cells	• Stimulation: Insulin (10 nM).<br>• Genetic manipulation: siRNA knockdown (Rlf, Rab10, RalA), protein overexpression of mutant forms (Rab10 Q68L, Rab10 T23N, RalA F39L, AS160 4A), expression of Rlf-CAAX.	• 2-deoxyglucose uptake assay.<br>• GLUT4 translocation assay (measurement of GLUT4 translocation by immunofluorescence).<br>• Immunoblotting (Western blot).<br>• Pull-down assay (using GST-Rim1-RBD, GST-Sec5-RBD, GST-RalBP1).<br>• Subcellular fractionation.	• 15 min (for translocation) and 30 min (for glucose uptake) after insulin treatment.	• Basal.<br>• Control siRNA.<br>• Vector control.<br>• Wild-type proteins.	Demonstration of the existence of a novel G protein cascade in insulin-stimulated GLUT4 membrane trafficking	• Rab10<br>• RalA	Stimulation	Identification of a G protein signaling cascade: Rab10 Stimulation recruits Rlf (a GEF), which in turn activates RalA. RalA subsequently interacts with the exocyst complex to promote GLUT4 translocation.	Docking										Unclear risk (Not reported)	Unclear risk (Not reported)	Low risk	Unclear risk (Not reported)	Unclear risk (Not reported)	Low risk
A.27	10.1091/mbc.E10-08-0665	Chen et al., 2011	United States	To identify and characterize the Ral GAP (RGC) complex that mediates downstream Stimulation of RalA in the PI 3-kinase/Akt signaling pathway in response to insulin.	• 3T3-L1 adipocytes (differentiated adipocytes)<br>• COS-1 cells<br>• 293T cells<br>• Mouse epididymal adipose tissue	• Stimulation: Insulin (concentrations ranging from 1 nM to 100 nM).<br>• Pharmacological inhibition: Wortmannin (PI3K inhibitor), Akti-1/2 (Akt inhibitor).<br>• Genetic intervention: siRNA knockdown (RGC1, RGC2, Akt1, Akt2, GARNL1), expression of plasmids (Myc-Akt, HA-RGC1, FLAG-RGC2, RalA mutants).	• Pull-down assay (to detect active RalA).<br>• Immunoprecipitation.<br>• Western blot.<br>• In vitro kinase assay.<br>• In vitro GAP assay.<br>• [14C]2-deoxyglucose uptake assay.<br>• Confocal microscopy (quantification of Myc-GLUT4-eGFP translocation).	• Molecular signaling: 2 min to 90 min after insulin stimulation.<br>• Glucose uptake: 30 min.<br>• siRNA: 4–7 days after siRNA transfection (knockdown).	• Basal.<br>• Control siRNA.<br>• Control IgG.<br>• Vector control.	The RGC complex serves as a molecular link connecting PI 3-kinase/Akt signaling to the transport mechanism responsible for delivering GLUT4 to the plasma membrane.	• RalA<br>• RGC1 (regulatory subunit)<br>• RGC2 (catalytic subunit / AS250)	Stimulation	Identification of the RGC complex (RGC1/RGC2) as a direct target of Akt. Akt phosphorylates RGC2, inducing a conformational change that reduces its accessibility/interaction with the substrate RalA, thereby allowing RalA to remain in an active (GTP-bound) state to recruit the exocyst complex and GLUT4	Tethering	Unclear risk (Not reported)	Low risk	Unclear risk (Not reported)	Unclear risk (Not reported)	Unclear risk (Not reported)	Unclear risk (Not reported)	Unclear risk (Not reported)	Low risk	Low risk	Unclear risk (Not reported)	Unclear risk (Not reported)	Low risk	Unclear risk (Not reported)	Low risk	Low risk
A.29	10.1074/jbc.M603274200	Ramm et al., 2006	Australia	to identify several 14-3-3 isoforms as AS160-interacting pr	• CHO-IR/IRS-1 cells<br>• 3T3-L1 adipocytes	• Stimulation: Insulin (100 nM).<br>• Pharmacological inhibition: Wortmannin (100 nM) (PI3K inhibitor).<br>• Genetic intervention: expression of mutant AS160 plasmids (AS160-WT, AS160-4P, AS160-T642A, AS160-4P-R1R, AS160-4P-R186K).	• Liquid chromatography–tandem mass spectrometry (LC-MS/MS).<br>• Immunoprecipitation.<br>• Western blotting.<br>• Immunofluorescence microscopy (to measure cell-surface GLUT4).	• 15 min (after insulin treatment).	• Basal.<br>• Vector control.<br>• Wild-type AS160.	Insulin-dependent binding between 14-3-3 and AS160 plays an important role in GLUT4 trafficking in adipocytes.	• 14-3-3 proteins	Stimulation	Identification that the physical interaction between 14-3-3 proteins and AS160 (triggered by Akt-mediated phosphorylation at Thr-642) is an essential step in regulating AS160 activity, thereby permitting GLUT4 translocation. This mechanism operates independently of the AS160–IRAP interactio	Signaling										Unclear risk (Not reported)	Unclear risk (Not reported)	Low risk	Unclear risk (Not reported)	Unclear risk (Not reported)	Low risk
A.31	10.1074/jbc.M010785200	Emoto et al., 2001	United States	To test the hypothesis that optimal insulin-mediated GLUT4 translocation requires a microtubule-based kinesin motor in the context of an intact microtubule and F-actin cytoskeleton.	• Mouse 3T3-L1 adipocyte	• Stimulation: Insulin (100 nM).<br>• Pharmacological inhibition: Nocodazole (30 µM), Colchicine (10 µM) to disrupt microtubules; Latrunculin B (10 µM) to disrupt F-actin.<br>• Genetic intervention: overexpression of hTau40 protein (using adenovirus) to inhibit kinesin function.	• Immunofluorescence microscopy (confocal).<br>• Plasma membrane lawn assay (analysis of the plasma membrane to quantify GLUT4).	• 0, 3, 6, 9, 12, 15 min (for time-course experiments with hTau40).<br>• 60 min (for pharmacological inhibition experiments).	• Basal.<br>• Control (no drug treatment).<br>• Control virus (empty adenovirus vector / GFP).	Insulin-driven delivery of GLUT4 to the plasma membrane is guided by both the actin- and microtubule-based cytoskeletons, together with a kinesin moto	• Kinesin<br>• Microtubules (tubulin)<br>• F-actin	Stimulation	Identification of a specific role for the kinesin molecular motor in transporting GLUT4 toward the plus (+) ends of microtubules (toward the plasma membrane). Insulin promotes this process, and hTau40 (which competes for kinesin-binding sites on microtubules) slows this transport.	Translocation										Unclear risk (Not reported)	Unclear risk (Not reported)	Low risk	Unclear risk (Not reported)	Unclear risk (Not reported)	Low risk
A.38	10.1007/s00125-016-4151-9	Zhu et al., 2016	China	To test the hypothesis that phosphorylation of TBC1D1 at Ser231 and/or its binding to 14-3-3 proteins may mediate AMPK-regulated glucose homeostasis and muscle glucose uptake	• Animals: Mice.<br>• Mouse models: Tbc1d1 Ser231Ala knock-in mice, Ampkα1/α2-mKO (skeletal muscle-specific knockout) mice, and wild-type (WT) mice.<br>• Tissue samples: Isolated soleus and EDL (extensor digitorum longus) muscles	• Chemicals (in vivo/ex vivo): AICAR (AMPK activator); 0.5 mg/g (intraperitoneal injection); 0.15 mmol/l, 0.25 mmol/l, 2 mmol/l (ex vivo muscle incubation).<br>• Chemicals (in vivo): Insulin (0.75 mU/g).<br>• Physiology: Exercise (treadmill running).<br>• Genetics: Knock-in mutation (Tbc1d1 Ser231Ala) and knockout (Ampkα1/α2).	• Blood glucose measurement.<br>• 2-deoxy-D-[1,2-³H]glucose uptake assay.<br>• Photolabelling of cell-surface GLUT4 (using Bio-LC-ATB-BGPA).<br>• Immunoblotting (Western blot), immunoprecipitation, 14-3-3 overlay assay.<br>• Immunofluorescence staining.	• Tolerance tests: 0, 15, 30, 45, 60, 90, 120 min after injection.<br>• Ex vivo uptake: 50 min incubation with AICAR + 10 min uptake.<br>• Exercise: 35 min running.	• Wild-type (WT).<br>• Basal.<br>• Resting	Phosphorylation of TBC1D1 at Ser231 and/or its binding to 14-3-3 proteins partially contributes to AMPK-regulated glucose homeostasis and muscle glucose uptake, depending on the cellular context (resting or stimulated).	• TBC1D1	Stimulation	Phosphorylation of TBC1D1 at Ser231 and/or its interaction with 14-3-3 proteins partially mediates AMPK-driven glucose homeostasis and muscle glucose uptake; however, this role is context-dependent (effective with low-dose AICAR but not with exercise).	Signaling	Unclear risk (Not reported)	Low risk	Unclear risk (Not reported)	Unclear risk (Not reported)	Unclear risk (Not reported)	Unclear risk (Not reported)	Unclear risk (Not reported)	Low risk	Low risk	Unclear risk (Not reported)	Unclear risk (Not reported)	Low risk	Unclear risk (Not reported)	Unclear risk (Not reported)	Low risk
A.41	10.1074/jbc.M117.806786	Whitfield et al., 2017	Canada	To explore the metabolic consequences of deleting TBC1D1 in skeletal muscle at rest and during contraction using a rat TBC1D1-KO model, particularly in the context that this model does not exhibit a compensatory reduction in total GLUT4 protein as observed in mice.	• Animals: Sprague-Dawley rats.<br>• Models: TBC1D1 knockout (TBC1D1 KO) rats and wild-type (WT) rats.<br>• Tissues: Red gastrocnemius (RG) and white gastrocnemius (WG), extensor digitorum longus (EDL).	• Genetic intervention: Tbc1d1 gene knockout.<br>• Insulin stimulation: Intravenous injection (1 U/kg) or intraperitoneal injection (1 U/kg).<br>• Contraction stimulation: Electrical stimulation of the sciatic nerve (100 Hz/3 s at 6–8 V, 15 min).<br>• Exercise: Treadmill running to exhaustion (time to exhaustion).<br>• Substrate oxidation experiments (ex vivo): Incubation of muscle strips with glucose or palmitate.	• Western blotting (measurement of TBC1D1, AS160, Akt2, AMPK, ACC, GLUT4, FAT/CD36 proteins).<br>• Isolation of plasma membranes (giant sarcolemmal vesicles) to measure GLUT4 translocation.<br>• In vivo glucose clearance (using tritiated 2-deoxy-D-glucose).<br>• Glucose tolerance test (GTT) and insulin tolerance test (ITT).<br>• Indirect calorimetry (measurement of RER, fat/carbohydrate oxidation).<br>• Mitochondrial respiration (Oxygraph-2K on permeabilized muscle fibers).	• Insulin: 30 min after injection.<br>• Contraction: After 15 min of electrical stimulation.<br>• Glucose/insulin tolerance: Monitored up to 120 min (GTT) and 45 min (ITT)	• Wild-type (WT) rats (wild-type control group).<br>• Comparison between basal and stimulated (insulin/contraction) conditions.	The role of TBC1D1 in exercise tolerance and contraction-mediated translocation of GLUT4 to the skeletal muscle plasma membrane.	• TBC1D1	Stimulation	The study identifies a critical role for TBC1D1 in regulating contraction-mediated GLUT4 translocation, but not insulin-stimulated pathways. This differs from previous mouse models confounded by reduced total GLUT4 levels; in this rat model, total GLUT4 content is unchanged, yet GLUT4 translocation is still impaired during contraction.	Translocation	Unclear risk (Not reported)	Low risk	Unclear risk (Not reported)	Unclear risk (Not reported)	Unclear risk (Not reported)	Unclear risk (Not reported)	Unclear risk (Not reported)	Low risk	Low risk	Unclear risk (Not reported)	Unclear risk (Not reported)	Low risk	Unclear risk (Not reported)	Unclear risk (Not reported)	Low risk
A.42	10.1074/jbc.M114.603977	Belman et al., 2015	United States	To investigate the role of acetylation of the protein TBC1D1 (referred to as TUG in this study) and the deacetylase enzyme SIRT2 in regulating intracellular retention of GLUT4-containing vesicles (GSVs) and insulin sensitivity.	• Cells: 3T3-L1 adipocytes, HEK293 cells.<br>• Animals: Male C57BL/6J mice (SIRT2 knockout and wild-type).	• Stimulation: Insulin (160 nM for cells; 1 U/kg or 8 units/kg for mice), glucose (for GTT).<br>• Chemicals: Nicotinamide (sirtuin inhibitor).<br>• Genetics: Expression of TUG mutants (TUG 5R, K549R), overexpression (SIRT2, ACBD3), shRNA knockdown (ACBD3, TUG), SIRT2 knockout (in mice).	• Immunoprecipitation (IP) and immunoblotting (WB) (detection of acetyl-lysine, protein–protein interactions).<br>• Cell surface biotinylation (measurement of surface GLUT4/IRAP).<br>• Flow cytometry (using GLUT4 reporter).<br>• Confocal microscopy (localization of ACBD3 and GLUT4).<br>• In vitro deacetylation assay.<br>• Intraperitoneal glucose tolerance tests (IPGTT).	• Cells (insulin): 7–12 min (e.g., "160 nM insulin for 7 min", "12 more minutes" for biotinylation).<br>• Mice (GTT): 0, 15, 30, 45, 60, 90, 120 min.<br>• Mice (TUG processing): 20 min after insulin injection.	• Basal (no insulin) vs insulin.<br>• TUG WT vs TUG mutants (5R, K549R).<br>• Control shRNA vs ACBD3 shRNA.<br>• Wild-type mice vs SIRT2 KO mice.	Acetylation of TUG regulates its interaction with Golgi matrix proteins and is regulated by SIRT2. Moreover, acetylation of TUG enhances its ability to retain GSVs within unstimulated cells and enhances insulin-stimulated glucose uptake.	• TUG (ASPSCR1)	Stimulation	Identification that TUG is acetylated at the C terminus (lysine 549). SIRT2 is the deacetylase enzyme for TUG. Acetylation of TUG modulates its interaction with Golgi matrix proteins (specifically reducing interaction with ACBD3, a newly identified anchoring protein), thereby enhancing retention of GLUT4 within insulin-responsive storage compartments (GSVs	Tethering	Unclear risk (Not reported)	Low risk	Unclear risk (Not reported)	Unclear risk (Not reported)	Unclear risk (Not reported)	Unclear risk (Not reported)	Unclear risk (Not reported)	Low risk	Low risk	Unclear risk (Not reported)	Unclear risk (Not reported)	Low risk	Unclear risk (Not reported)	Unclear risk (Not reported)	Low risk
A.45	10.1210/mend.13.3.0242	Zhou et al., 1999	United States	To determine whether IRS-3 and IRS-4 can mediate the effects of insulin in stimulating GLUT4 translocation, and to compare their efficacy with IRS-1 and IRS-2	• Primary rat adipocytes	• Stimulation: Insulin (concentrations ranging from 0.01 nM to 100 nM, maximum 60 nM).<br>• Genetic intervention: Cotransfection to express GLUT4-HA and recombinant IRS proteins (hIRS-1, mIRS-2, mIRS-3, hIRS-4) or mutant forms (mIRS-3-F4)	• Immunoblotting (Western blot) to detect tyrosine-phosphorylated proteins.<br>• Immunoprecipitation.<br>• Cell surface GLUT4 translocation assay (measurement of cell-surface GLUT4-HA using anti-HA antibody).	• 2 min (for phosphorylation after insulin stimulation).<br>• Overnight (after transfection to allow protein expression).	• Basal.<br>• Control vector (pCIS2 – empty vector)	IRS-3 and IRS-4 are capable of regulating PI 3-kinase–dependent metabolic actions of insulin in adipocytes, and IRS proteins play a physiological role in regulating GLUT4 trafficking.	• IRS-3, IRS-4	Stimulation	Demonstration that IRS-3 and IRS-4 (although IRS-4 is not endogenously expressed in mouse adipocytes) are capable of activating the PI 3-kinase pathway and promoting GLUT4 translocation similarly to IRS-1/2. This indicates functional redundancy within the IRS protein family in regulating GLUT4 via PI 3-kinase.	Signaling<br>Translocation										Unclear risk (Not reported)	Unclear risk (Not reported)	Low risk	Unclear risk (Not reported)	Unclear risk (Not reported)	Low risk
A.46	10.1074/jbc.271.30.17605	Martin et al., 1996	United States	To determine whether isolated Stimulation of phosphatidylinositol 3-kinase (PI3K) is sufficient to mediate actin reorganization and GLUT4 translocation.	• Mouse 3T3-L1 adipocyte	• Stimulation: Insulin (100 ng/ml).<br>• Genetic intervention (microinjection): microinjection of plasmids expressing constitutively active PI3K mutants or kinase-dead mutants.<br>• Pharmacological inhibition: Wortmannin (PI3K inhibitor).	• Single-cell microinjection.<br>• Immunofluorescence microscopy to detect GLUT4 and actin.	• 5–6 h after microinjection.<br>• 15 min after insulin treatment.	• Basal (no insulin, IgG control injection).<br>• Insulin (positive control).<br>• p110* Δkin (inactive mutant control).<br>• p110* + Wortmannin (specific inhibition control)	Isolated Stimulation of phosphatidylinositol 3-kinase alone can initiate signaling cascades leading to both actin rearrangement and GLUT4 trafficking even in the absence of insulin stimulation	• PI3K	Stimulation	Stimulation of phosphatidylinositol 3-kinase leads to actin rearrangement and GLUT4 translocation.	Signaling<br>Translocation										Unclear risk (Not reported)	Unclear risk (Not reported)	Low risk	Unclear risk (Not reported)	Unclear risk (Not reported)	Low risk
A.50	10.1128/MCB.21.22.7852-7861.2001	Braiman et al., 2001	Israel	To investigate the role of protein kinase C isoform ζ in insulin-stimulated glucose uptake and GLUT4 translocation using a primary cultured skeletal muscle cell model	• Primary rat skeletal muscle cells.	• Stimulation: Insulin (100 nM).<br>• Genetic intervention: Adenoviral infection to overexpress wild-type PKC-ζ (WTPKC-ζ), dominant-negative PKC-ζ (DNPKC-ζ – kinase-dead), and wild-type PKC-α (WTPKC-α).	• Immunoprecipitation.<br>• Western blot analysis.<br>• Cell fractionation (separation of plasma membrane, intracellular membranes, microsomes).<br>• PKC activity assay (kinase activity measurement).<br>• In vitro phosphorylation assay.<br>• Glucose uptake ([³H]2-deoxyglucose).	• Kinase activity and phosphorylation: 0, 1, 5, 15, 30 min after insulin stimulation.<br>• Glucose uptake and translocation (after viral infection): 16 h after infection, followed by 30 min insulin treatment.	• Control (no viral infection).<br>• Basal (no insulin treatment).<br>• WTPKC-α overexpression (to verify isoform specificity).	PKCα regulates insulin-stimulated GLUT4 translocation and glucose transport through the unique colocalization of this isoform with GLUT4-containing compartments.	• PKCζ	Stimulation	The study demonstrates that PKC-ζ physically associates with GLUT4-containing compartments and, when activated, directly phosphorylates VAMP2 at a serine residue. Phosphorylation of VAMP2 by PKC-ζ is a critical step regulating GLUT4 translocation and docking/fusion at the plasma membrane.	Fusion<br>Docking										Unclear risk (Not reported)	Unclear risk (Not reported)	Low risk	Unclear risk (Not reported)	Unclear risk (Not reported)	Low risk
A.51	10.1016/j.devcel.2007.07.007	Chen et al., 2007	United States	To identify the role of the small G protein RalA in insulin-stimulated GLUT4 trafficking, specifically the mechanism of interaction with the exocyst complex and the molecular motor Myo1c to direct GLUT4 vesicles to the plasma membrane.	• Mouse 3T3-L1 adipocytes<br>• Rat skeletal muscle cells (L6 myoblasts stably expressing Myc-Glut4).	• Stimulation: Insulin (100 nM).<br>• Pharmacological inhibition: Wortmannin (100 nM) to inhibit PI3K.<br>• Genetic intervention: Expression of RalA mutants: dominant-negative (S28N), constitutively active (G23V), fast-cycling (F39L). siRNA knockdown: RalA, RalB, Sec5, Exo84, Myo1c.	• RalA Stimulation assay (using GST-Sec5-RBD to pull down RalA-GTP).<br>• 2-deoxyglucose uptake assay.<br>• Plasma membrane sheet assay / confocal microscopy (measurement of GLUT4 translocation).<br>• Immunoprecipitation & immunoblotting (protein–protein interaction analysis).	• RalA Stimulation: 2, 5, 10, 20 min after insulin stimulation.<br>• Glucose uptake / translocation: after 20–30 min of insulin stimulation.	• Basal.<br>• Control siRNA / empty vector.<br>• RalB knockdown	RalA performs two functions in insulin action: acting as a cargo receptor for the Myo1c motor, and serving as a signal for assembly of the exocyst complex to target GLUT4 vesicles to the plasma membrane	• RalA	Stimulation	RalA plays a dual role:<br>1. It acts as an insulin-responsive signaling molecule on GLUT4 vesicles to recruit the exocyst complex (specifically Sec5 and Exo84), enabling vesicle tethering at the plasma membrane;<br>2. It serves as a molecular bridge linking GLUT4 vesicles to the Myo1c motor (via calmodulin), thereby supporting vesicle transport along actin filaments.	Translocation										Unclear risk (Not reported)	Unclear risk (Not reported)	Low risk	Unclear risk (Not reported)	Unclear risk (Not reported)	Low risk
A.52	10.1074/jbc.M201152200	Chen et al., 2002	United States	To test the hypothesis that AMPK increases glucose transport through atypical protein kinase C (aPKC) by activating proline-rich tyrosine kinase-2 (PYK2), components of the ERK pathway, and phospholipase D (PLD	• Animals: C57BL/6J mice (vastus lateralis muscle), Sprague-Dawley rats (extensor digitorum longus – EDL muscle).<br>• Cells: L6 myotubes (rat skeletal muscle cell line).	• Physiological/chemical stimulation: Exercise (treadmill running), AICAR (AMPK activator), DNP (dinitrophenol – disrupts oxidative phosphorylation), insulin.<br>• Pharmacological inhibition: Genistein (tyrosine kinase inhibitor), PD98059 and UO126 (MEK1 inhibitors), 1-butanol (PLD inhibitor), Wortmannin (PI3K inhibitor), cell-permeable myristoylated PKC-ζ pseudosubstrate (aPKC inhibitor).<br>• Genetic intervention (L6 myotubes): Cotransfection or adenoviral infection with dominant-negative/kinase-inactive mutants of PYK2, GRB2, SOS, RAS, RAF, ERK1, ERK2, and PKC-ζ.	• [³H]2-deoxyglucose uptake (measurement of glucose uptake).<br>• Immune complex kinase assay (measurement of ERK and aPKC activity).<br>• Immunoprecipitation & immunoblotting (detection of phospho-PYK2, GLUT4).<br>• PLD activity assay (measurement via formation of phosphatidylethanol/phosphatidylbutanol).<br>• Cell fractionation (membrane fractionation to measure GLUT4 translocation).	• Kinase activity: 5, 10, 15 min (with insulin/exercise); 40–45 min (with AICAR); 60 min (with DNP).<br>• Glucose uptake: measured during the last 5–10 min of incubation (30–60 min depending on stimulus).	• Basal / control (no treatment).<br>• Insulin-treated (positive control).<br>• Vector control (transfection control).<br>• Inhibitor-treated or mutant-expressing groups compared with AICAR/DNP-treated groups alone.	AMPK-dependent effects of exercise on glucose transport are regulated through PYK2, the ERK pathway, PLD, and aPKCs.	• PYK2	Stimulation	AMPK activates PYK2 (a tyrosine kinase), leading to sequential Stimulation of GRB2/SOS → RAS → RAF → MEK → ERK. ERK activates PLD to generate phosphatidic acid (PA), which subsequently activates aPKCs (ζ, λ), ultimately leading to GLUT4 translocation.	Signaling<br>Translocation	Unclear risk (Not reported)	Low risk	Unclear risk (Not reported)	Unclear risk (Not reported)	Unclear risk (Not reported)	Unclear risk (Not reported)	Unclear risk (Not reported)	Low risk	Low risk	Unclear risk (Not reported)	Unclear risk (Not reported)	Low risk	Unclear risk (Not reported)	Unclear risk (Not reported)	Low risk

A.53	10.1152/ajpendo.00456.2004	Yamaguchi et al., 2005	Japan	To determine whether the increase in glucose uptake following AMPK Stimulation in adipocytes is mediated by an increased rate of GLUT4 translocation, and to investigate the roles of PI3K and p38 MAPK in this process	• Mouse 3T3-L1 adipocytes	• Stimulation: Insulin (10^{-7} mol/L), DNP (10^{-3} mol/L), AICAR (10^{-3} mol/L).<br>• Inhibition: Wortmannin (3×10^{-7} mol/L – PI3K inhibitor), SB-203580 (10^{-5} mol/L – p38 MAPK inhibitor).<br>• Gene transfer: Microinjection of GLUT4-eGFP cDNA into the nucleus	• Laser scanning confocal microscopy.<br>• 2-deoxyglucose uptake assay.	• Translocation: Images captured every 1 min over 20 min (analysis at 4, 10, and 20 min).<br>• Glucose uptake: 20 min incubation with stimulants.	• Basal.<br>• Insulin only, DNP only, AICAR only (positive controls).<br>• Combined inhibitor groups (Insulin/DNP/AICAR + Wortmannin/SB203580).	These results suggest that AMPK-activated GLUT4 translocation in 3T3-L1 adipocytes is mediated through the insulin-signaling pathway distal to the site of activated phosphatidylinositol 3-kinase or through a signaling system distinct from that activated by insulin. On the other hand, the increase of glucose uptake dependent on AMPK activators AICAR and DNP would be additionally due to enhancement of the intrinsic activity in translocated GLUT4 protein, possibly through a p38 MAPK-dependent mechanism.	• PI3K<br>• p38 MAPK	Stimulation	AMPK-induced GLUT4 translocation (AICAR/DNP) proceeds via a signaling pathway distinct from insulin (PI3K-independent) or downstream of PI3K. However, the increase in total glucose uptake requires participation of p38 MAPK, possibly to enhance the intrinsic activity of GLUT4 after translocation.	Translocation										Unclear risk (Not reported)	Unclear risk (Not reported)	Low risk	Unclear risk (Not reported)	Unclear risk (Not reported)	Low risk	
A.54	10.1172/JCI22681	Kanda et al., 2005	Japan	To elucidate the physiological function of Munc18c in insulin-stimulated exocytosis of GLUT4.	• Animals: Mice (C57BL/6J) (Munc18c+/+, +/−, −/−).<br>• Cells: Adipocytes differentiated from mesenchymal embryonic fibroblasts (MEFs) obtained from mouse embryos at day 13.5.	• Stimulation: Insulin (1 nM, 100 nM).<br>• Chemicals: Wortmannin (100 nM) – PI3K inhibitor.<br>• Lipid: Exogenous phosphatidylinositol 3-phosphate (PI3P) (delivered into cells using a polyamine carrier).<br>• Genetics: Munc18c gene knockout; expression of GLUT4-myc7-GFP (retrovirus); re-expression of Munc18c (adenovirus) in knockout cells.	• Immunoblotting (Western blot) & immunoprecipitation.<br>• Subcellular fractionation.<br>• Immunofluorescence microscopy (confocal).<br>• 2-deoxy-D-[1,2-^{3}H]glucose uptake assay.<br>• Northern blot analysis.	• Insulin signaling (phosphorylation): 5 min.<br>• Translocation/externalization: 20 min after insulin stimulation.<br>• Glucose uptake: 20 min insulin incubation, uptake measured for 5 min	• Wild-type (Munc18c+/+) and heterozygous (Munc18c+/−) adipocytes.<br>• Basal (0 nM insulin) vs insulin-stimulated.<br>• Control adenovirus (empty vector).	These findings demonstrate that Munc18c inhibits insulin-stimulated externalization of GLUT4 in a wortmannin-sensitive manner, and they suggest that disruption of the interaction between syntaxin4 and Munc18c in adipocytes might result in enhancement of insulin-stimulated GLUT4 externalization	• Munc18c	Inhibition	Munc18c acts as a negative regulator of the fusion step of GLUT4 vesicles with the plasma membrane by sequestering Syntaxin4. Insulin (via PI3K) releases Syntaxin4 from Munc18c. In the absence of Munc18c, this step is no longer restrained, resulting in facilitated fusion (even in the presence of wortmannin or with low insulin concentrations). PI3P can activate this process in the absence of Munc18c.	Fusion	Unclear risk (Not reported)	Low risk	Unclear risk (Not reported)	Unclear risk (Not reported)	Unclear risk (Not reported)	Unclear risk (Not reported)	Unclear risk (Not reported)	Low risk	Low risk	Low risk	Unclear risk (Not reported)	Low risk	Unclear risk (Not reported)	Unclear risk (Not reported)	Low risk	
A.57	10.1034/j.1600-0854.2000.010206.x	Millar et al., 2000	United Kingdom and Australia	To compare the trafficking of adipsin and GLUT4 to test the hypothesis that insulin exerts a general effect on protein export from the trans-Golgi network (TGN).	• Mouse 3T3-L1 adipocytes	• Stimulation: Insulin (1 μM).<br>• Chemical inhibitor: Butan-1-ol (30 mM – phospholipase D inhibitor), Butan-2-ol (control).<br>• Cell biological intervention: Endosome ablation using transferrin–HRP (TfHRP) complexes.	• Adipsin secretion assay.<br>• Plasma membrane lawn assay (measurement of GLUT4/GLUT1 translocation).<br>• 2-deoxyglucose uptake assay.<br>• Subcellular fractionation.<br>• Immunoadsorption (using TGN38 antibody).<br>• Phospholipase D activity assay.	• Adipsin secretion: 5, 10, 30 min after stimulation.<br>• GLUT4 translocation / glucose uptake: 15–60 min after insulin stimulation	• Basal (no insulin).<br>• Butan-2-ol-treated (non-PLD-inhibitory control).<br>• Non-ablated cells (cells without endosome ablation).	These data suggest that insulin stimulates the activity of the constitutive secretory pathway in adipocytes, possibly by increasing the budding step at the TGN through a phospholipase D–dependent mechanism. Insulin activates multiple pathways directed toward the cell surface in adipocytes.	• Adipsin	Stimulation	Insulin stimulates the activity of the constitutive secretory pathway—specifically adipsin—by enhancing the budding step at the trans-Golgi network (TGN) via a phospholipase D (PLD)-dependent mechanism. However, GLUT4 uses a distinct pathway to reach the plasma membrane that is independent of PLD and of the TGN-to-endosome route used by adipsin.	Translocation										Unclear risk (Not reported)	Unclear risk (Not reported)	Low risk	Unclear risk (Not reported)	Unclear risk (Not reported)	Low risk	
A.59	10.1210/me.2004-0413	van Dam et al., 2005	Australia	To analyze the role of Akt in different steps of insulin-stimulated GLUT4 translocation using a low-temperature inhibition approach (19 °C).	• Mouse 3T3-L1 adipocytes	• Stimulation: Insulin (200 nM).<br>• Physical condition: Incubation at low temperature (19 °C) versus physiological temperature (37 °C).<br>• Pharmacological inhibition: Wortmannin (100 nM – PI3K inhibitor), Calyculin A (100 nM – phosphatase inhibitor).<br>• Genetic intervention: Expression of HA-GLUT4, hTfR, or EGFP-PH/ARNO (to detect PIP3).	• Quantitative GLUT4 translocation assay (fluorescent antibody–based assay in 96-well plates).<br>• Immunofluorescence microscopy (confocal).<br>• Glucose uptake assay (2-deoxy-[^{3}H] glucose uptake).<br>• Western blotting (measurement of Akt, IR, and IRS-1 phosphorylation).<br>• Transferrin (Tf) recycling assay.	• GLUT4 translocation: 0, 2, 5, 10, 15, 20, 25 min (time course).<br>• Signaling (Akt, PI3K): 5, 15, 30 min after insulin stimulation.<br>• Glucose uptake: 20 min incubation (or time course), uptake measured for 2 min.	• 37 °C (control) versus 19 °C.<br>• Basal versus insulin-stimulated.<br>• Pre-insulin (insulin preincubation at 19 °C) versus no preincubation.	Insulin-stimulated GLUT4 trafficking consists of at least two regulatory steps:<br><br>1. Redistribution of GLUT4 vesicles to the cell cortex (this step is Akt-independent and not blocked at 19 °C);<br><br>2. Docking and/or fusion of GLUT4 vesicles with the plasma membrane (this step is Akt-dependent and blocked at 19 °C). Akt acts at a late stage of GLUT4 translocation.	• Akt	Stimulation	Docking and/or fusion of GLUT4 vesicles with the plasma membrane—this process is likely the primary Akt-dependent step in the regulation of insulin-stimulated glucose transport	Signaling										Unclear risk (Not reported)	Unclear risk (Not reported)	Low risk	Unclear risk (Not reported)	Unclear risk (Not reported)	Low risk	
A.60	10.1074/jbc.RA118.005040	Mafakheri et al., 2018	Germany	To express, purify, and biochemically characterize full-length TBC1D1 in vitro to determine how phosphorylation by AKT and AMPK regulates its function (GAP activity versus protein–protein interactions).	• Recombinant proteins expressed in Sf9 insect cells and E. coli.<br>• HEK293 cells (for studying protein–protein interactions in cells).	• In vitro phosphorylation: Using purified AKT2 and AMPK kinases.<br>• Point mutations: Generation of TBC1D1 variants R125W (obesity-associated) and R854K (GAP-inactive).<br>• Protein interaction: Incubation with the cytoplasmic tail of IRAP (GST-CIRAP) or full-length IRAP.	• RabGAP assay (measurement of GTP hydrolysis rates of Rab10/Rab8a/Rab14 using radioactive [γ-^{32}P]GTP).<br>• GST pulldown assay (in vitro protein–protein interaction).<br>• Co-immunoprecipitation.<br>• Mass spectrometry (identification of phosphorylation sites).<br>• Western blot.	• GAP reactions: 10 min or 30 min.<br>• Phosphorylation reactions: 20–30 min.<br>• Protein interaction incubations: 1 h or 30 min.	• Non-phosphorylated TBC1D1 versus TBC1D1 phosphorylated by AKT2/AMPK.<br>• TBC1D1 wild-type versus mutants (R125W, R854K).<br>• Full-length TBC1D1 versus truncated GAP-domain constructs.<br>• Presence versus absence of IRAP/GST-CIRAP.	Regions outside the GAP domain may contribute to the catalytic activity of TBC1D1, and recruitment of TBC1D1 to GLUT4-containing vesicles is regulated by insulin- and contraction-mediated phosphorylation.	• TBC1D1	Inhibition	Phosphorylation of TBC1D1 does not directly regulate its enzymatic (GAP) activity but instead controls recruitment of TBC1D1 to GLUT4-containing vesicles through interaction with IRAP. Under basal conditions, TBC1D1 binds IRAP on vesicles to inhibit Rab (retaining GLUT4 intracellularly). Upon phosphorylation by insulin or contraction, TBC1D1 dissociates from IRAP (and binds 14-3-3), allowing Rab Stimulation to promote GLUT4 translocation.	Tethering										Unclear risk (Not reported)	Unclear risk (Not reported)	Low risk	Unclear risk (Not reported)	Unclear risk (Not reported)	Low risk	
A.61	10.1074/jbc.274.3.1458	Kupriyanova et al., 1999	United States	To identify endogenous protein kinase activity associated with GLUT4-containing vesicles and to determine the kinase responsible for phosphorylation of vesicle components in response to insulin.	• Primary rat adipocytes	• Stimulation: Insulin (10 nM).<br>• Pharmacological inhibition: Wortmannin (100 nM for live cells in vivo; 5 μM for in vitro reactions), PKI (PKA inhibitor), Calphostin C (PKC inhibitor).	• Immunoadsorption (immunoisolation of GLUT4 vesicles using the 1F8 antibody).<br>• In vitro protein kinase assay (using [γ-^{32}P]ATP).<br>• Western blot analysis.<br>• MonoQ anion-exchange chromatography.<br>• Immunoprecipitation.	• Time course of phosphorylation: 0, 2, 4, 8, 16 min after insulin stimulation.<br>• Wortmannin treatment: 20 min preincubation.	• Basal (no insulin).<br>• IgG control (beads coupled to nonspecific IgG as control for 1F8 beads).<br>• In vitro vs in vivo phosphorylation (comparison of wortmannin inhibition in intact cells versus isolated vesicles)	We show that GLUT4-containing vesicles immunoisolated from rat adipocytes possess a tightly associated protein kinase activity and several phosphorylation substrates. The vesicle-associated protein kinase was identified as Akt-2 by MonoQ chromatography and Western blot analysis. Phosphorylation of vesicle component proteins, as well as artificial substrates, by this vesicle-associated protein kinase is rapidly increased by insulin in a wortmannin-sensitive manner.	• Akt2	Stimulation	Identification of Akt-2 as the enzyme directly associated with GLUT4-containing vesicles that phosphorylates GLUT4 and other vesicle component proteins. This represents a direct molecular link between the PI3K/Akt signaling pathway and the GLUT4 vesicle trafficking machinery. Proposed mechanism: Insulin → PI3K → PIP3 accumulation on GLUT4 vesicles → recruitment and Stimulation of Akt-2 → Akt-2–mediated phosphorylation of vesicle proteins → release of vesicles from intracellular retention/tethering → translocation to the plasma membrane.	Signaling										Unclear risk (Not reported)	Unclear risk (Not reported)	Low risk	Unclear risk (Not reported)	Unclear risk (Not reported)	Low risk	
A.62	10.1016/j.jbc.2021.100637	Eickelschulte et al., 2021	Germany	To investigate the effects of phosphorylation of TBC1D4 (AS160) by AKT and AMPK on its enzymatic activity and protein–protein interactions, using full-length recombinant TBC1D4 rather than truncated forms used in previous studies.	• Recombinant proteins: Full-length TBC1D4 expressed in Sf9 insect cells.<br>• Cells: HEK293 cells (used for Co-IP), Sf9 insect cells.<br>• Animals: C57BL/6J mice (used to validate phosphorylation in vivo).	• In vitro phosphorylation: Using purified AKT and AMPK kinases.<br>• In vivo stimulation (mice): Insulin (1 IU/kg), AICAR (250 mg/kg).	• RabGAP assay (measurement of GTP hydrolysis activity of Rab10).<br>• Mass spectrometry (LC-MS) (identification and kinetics of phosphorylation sites).<br>• Co-immunoprecipitation (Co-IP) & GST pulldown (protein–protein interaction assays).<br>• Size-exclusion chromatography (SEC) (determination of molecular size/oligomerization).<br>• Western blot.	• GAP reactions: 30 min.<br>• Phosphorylation reactions: 5–50 min.<br>• In vivo stimulation (mice): 60 min.	• Full-length TBC1D4 versus truncated GAP-domain constructs.<br>• Non-phosphorylated TBC1D4 versus AKT- or AMPK-phosphorylated TBC1D4.<br>• Presence versus absence of IRAP/14-3-3.	Insulin and muscle contraction may regulate TBC1D4 function primarily by disrupting recruitment of the RabGAP to GLUT4-containing vesicles.	• TBC1D4 (AS160)	Inhibition	Insulin and muscle contraction regulate TBC1D4 function mainly by preventing recruitment of this RabGAP to GLUT4 vesicles through disruption of the interaction between TBC1D4 and IRAP, rather than by altering its catalytic enzyme activity. Full-length TBC1D4 forms oligomeric complexes (~600 kDa).	Tethering	Unclear risk (Not reported)	Low risk	Unclear risk (Not reported)	Unclear risk (Not reported)	Unclear risk (Not reported)	Unclear risk (Not reported)	Unclear risk (Not reported)	Low risk	Low risk	Unclear risk (Not reported)	Unclear risk (Not reported)	Low risk	Unclear risk (Not reported)	Unclear risk (Not reported)	Low risk	
A.64	10.1083/jcb.200408182	Yamada et al., 2005	Japan	To identify the molecular mechanism underlying Akt isoform specificity in regulating glucose metabolism, specifically the role of Akt2-mediated phosphorylation of Synip in docking and fusion of GLUT4-containing vesicles.	• Cells: CHO-IR cells (CHO cells overexpressing the human insulin receptor) and 3T3-L1 adipocytes.	• Stimulation: Insulin (100 nM).<br>• Pharmacological inhibition: Wortmannin (PI3K inhibitor), GF109203X (PKC inhibitor).<br>• Genetic intervention:<br>• siRNA knockdown: Akt1, Akt2.<br>• Plasmid expression: FLAG-WT-Synip, S99F-Synip (nonphosphorylatable mutant), CAP sorbin (dominant-interfering CAP), p85 mutant (dominant-interfering PI3K).	• Co-immunoprecipitation (to examine protein–protein interactions).<br>• Western blotting (immunoblotting) to detect proteins.<br>• Electroporation (gene transfer).	• 15 min after insulin stimulation	• Basal (no insulin) vs insulin-stimulated.<br>• Control siRNA vs Akt1/Akt2 siRNA.<br>• WT-Synip vs S99F-Synip.	Synip is a preferred Akt2-specific substrate with an unusual dual consensus phosphorylation site. Akt2-dependent phosphorylation at serine 99 is essential for insulin-stimulated dissociation of Synip from Syntaxin4, as well as for translocation and plasma membrane fusion of GLUT4-containing vesicles.	• Akt2	Stimulation	Identification of Synip as an Akt2-specific substrate (containing an unusual RxKxRS97xS99 motif, with only Ser99 phosphorylated by Akt2). Phosphorylation of Ser99 by Akt2 is required to release Syntaxin4 from Synip, allowing Syntaxin4 to participate in SNARE complex formation to mediate docking and fusion of GLUT4 vesicles. This mechanism explains the isoform-specific role of Akt2 in glucose metabolism.	Docking<br>Fusion										Unclear risk (Not reported)	Unclear risk (Not reported)	Low risk	Unclear risk (Not reported)	Unclear risk (Not reported)	Low risk	
A.65	10.1016/j.cellsig.2013.02.023	Nozaki et al., 2013	Japan	To investigate the role of Akt isoforms (particularly Akt2) in regulating Rac1 activity and to elucidate the relationship between the PI3K/Akt signaling pathway and Rac1 in insulin-stimulated GLUT4 translocation in skeletal muscle cells.	• Mouse skeletal muscle cells: C2C12 cell line	• Stimulation: Insulin (100 nM or 1 μM).<br>• Pharmacological inhibition: Wortmannin (100 nM – PI3K inhibitor), Akti-1/2 (10 μM – Akt inhibitor).<br>• Genetic intervention:<br>• siRNA knockdown: Akt1, Akt2, Rac1.<br>• Plasmid expression: Constitutively active Akt2 (myr-Akt2), dominant-negative Akt2 (Akt2-K181M), constitutively active Rac1 (Rac1-G12V), dominant-negative Rac1 (Rac1-T17N).	• GST pull-down assay (measurement of Rac1-GTP using GST-PAK1-CRIB).<br>• Immunoblotting (Western blot).<br>• Immunofluorescence microscopy (to visualize GLUT4-myc translocation).<br>• 2-deoxyglucose uptake assay.	• Rac1/Akt activity: 2, 5, 10, 20 min after insulin stimulation.<br>• GLUT4 translocation / glucose uptake: 20 min after insulin stimulation.	• Basal (no insulin) vs insulin.<br>• Control siRNA vs Akt1/Akt2/Rac1 siRNA.<br>• Vector control vs mutant plasmids (CA/DN).	Stimulation of Rac1 is a critical downstream event of the PI3K–Akt kinase cascade in insulin regulation of glucose transport in muscle cells. Notably, insulin-induced Rac1 Stimulation is completely sensitive to Akt inhibition. These results highlight a central role of Rac1 in Akt-mediated insulin signaling and propose a novel mechanism for insulin-dependent regulation of Rac1.	• Akt2	Stimulation	Akt2 functions upstream and is responsible for regulating Rac1 activity in insulin signaling. The proposed mechanism is that Akt2 phosphorylates a Rac1-specific guanine nucleotide exchange factor (GEF), preliminarily identified as FLJ00068/PLEKHG4 (homologous to P-Rex1), thereby activating Rac1 to promote GLUT4 translocation through actin cytkeleton remodeling.	Signaling										Unclear risk (Not reported)	Unclear risk (Not reported)	Low risk	Unclear risk (Not reported)	Unclear risk (Not reported)	Low risk	
A.67	10.1242/jcs.166561	Kioumourtzoglou et al., 2015	United Kingdom	To investigate the relationship between intracellular GLUT4-containing vesicle (IGV) populations that are positive for sortilin and those positive for cellugyrin, in order to define their roles in GLUT4 trafficking under basal conditions and upon insulin stimulation.	• Mouse 3T3-L1 adipocytes and HeLa cells.	• Stimulation: Insulin (100 nM).<br>• Genetic intervention: Expression of mutant cellugyrin plasmid (tdTomato-Cellugyrin FDL/AAA) to alter its localization to the plasma membrane.	• In situ Proximity Ligation Assay (PLA) (to detect protein proximity/interactions).<br>• Immunofluorescence microscopy.	• 5 min and 20 min after insulin stimulation.	• Basal (no insulin).<br>• Control (negative control in PLA, omission of primary antibody).<br>• Wild-type cellugyrin vs mutant cellugyrin (FDL/AAA)	Insulin stimulates trafficking from cellugyrin-containing membranes to sortilin-containing membranes, and cellugyrin-positive IGVs function as an insulin-sensitive reservoir to replenish GSVs following insulin-stimulated GLUT4 exocytosis. The data further support the existence of a pathway from cellugyrin-containing membranes directly to the surface of 3T3-L1 adipocytes that bypasses GSVs under basal conditions, with insulin diverting traffic from this pathway into GSVs.	• Cellugyrin (Synaptogyrin-2)<br>• Sortilin	Stimulation	Proposed two-pathway trafficking model:<br><br>1. A basal pathway that delivers GLUT4 directly from cellugyrin-containing vesicles to the plasma membrane;<br><br>2. An insulin-stimulated pathway that routes GLUT4 through sortilin-containing vesicles (GSVs). Insulin diverts trafficking from pathway 1 to pathway 2. Cellugyrin-containing vesicles act as a reservoir to replenish GSVs after GLUT4 exocytosis.	Translocation										Unclear risk (Not reported)	Unclear risk (Not reported)	Low risk	Unclear risk (Not reported)	Unclear risk (Not reported)	Low risk	
A.74	10.1074/jbc.M111.318238	Tan et al., 2012	Australia	To explore the relationships between individual components in the insulin signaling network (particularly Akt) and to determine whether this pathway is capable of demultiplexing upstream insulin signals into distinct downstream outputs.	• Mouse 3T3-L1 adipocytes and HeLa cells.	• Physiological stimulation: Insulin (concentrations from 0.1 nM to 100 nM) and PDGF (20 ng/ml).<br>• Genetic intervention: siRNA knockdown of Akt2; retroviral infection to express HA-GLUT4 or HA-GLUT4 together with PDGFR.<br>• Pharmacological inhibition:<br>• Isoform-specific Akt inhibitors (Akti1, Akti2), Akt inhibitor MK-2206, rapamycin (mTOR inhibitor), wortmannin (PI3K inhibitor).	• Western blot (measurement of protein phosphorylation levels).<br>• Quantitative GLUT4 Translocation Assay (quantitative measurement of HA-GLUT4 translocation using immunofluorescence in 96-well plates).<br>• Protein Synthesis Assay (measurement of protein synthesis via incorporation of [^{3}H]leucine).<br>• Cationic silica isolation (plasma membrane isolation).<br>• Computational modeling (CellWorks Virtual Adipocyte Platform).	• Signaling: 10, 20, 30, and 60 minutes after stimulation.<br>• GLUT4 translocation: 20 minutes after insulin stimulation.<br>• Protein synthesis: 20 minutes after insulin stimulation.	• Basal (unstimulated condition, no insulin).<br>• DMSO-treated control group (vehicle).<br>• IgG control group (for immunoprecipitation).<br>• Non-transfected cells versus HA-GLUT4–transfected cells.	Akt functions as a signal amplification node and enables insulin signaling to be split into distinct cellular responses	• Akt (including Akt1 and Akt2 isoforms).	Stimulation	The study identifies Akt not merely as a relay node but as a "demultiplexer" capable of amplifying sensitivity and splitting a single upstream insulin signal into distinct downstream metabolic outputs through sigmoidal relationships and different Stimulation thresholds for individual substrates.	Signaling										Unclear risk (Not reported)	Unclear risk (Not reported)	Low risk	Unclear risk (Not reported)	Unclear risk (Not reported)	Low risk	
A.75	10.1111/tra.12282	Anthony J. Kee, 2015	Australia	To investigate the role of tropomyosin Tpm3.1-defined actin filaments in regulating GLUT4 translocation and glucose uptake in adipose tissue and skeletal muscle.	• Differentiated 3T3-L1 adipocytes<br>• Mice (C57BL/6 and FVB/N strains), including transgenic mice overexpressing Tpm3.1 (Tg) and Tpm3.1 knockout (KO) mice<br>• Mouse white adipose tissue (WAT)<br>• Mouse skeletal muscle	• Insulin stimulation: 100 nmol/mL for 30 minutes in 3T3-L1 cells; intraperitoneal injection of 0.5 U/kg or 1.5 U/kg body weight in mice<br>• Pharmacological intervention: Tpm3.1 inhibitor (TR100) at concentrations of 20, 50, and 100 μM for 1 hour<br>• Genetic intervention: Overexpression of Tpm3.1 (Tg) or deletion of the Tpm3.1 gene (KO)<br>• Diet: High-fat diet (HFD – 45% of energy from fat) for 9–16 weeks	• ^{3}H-2-deoxyglucose (2-DG) uptake assay<br>• Western blotting<br>• Confocal fluorescence microscopy<br>• Glucose tolerance test (GTT)<br>• Insulin tolerance test (ITT)<br>• Illumina BeadArray analysis<br>• In vitro motility assays	• Measurement of blood glucose levels at 0, 15, 30, 45, 60, 75, 90, 105, and 120 minutes after glucose or insulin injection	• Wild-type (WT) mice<br>• DMSO-treated cells (vehicle control)<br>• Basal state (no insulin)	The balance among actin filament components determines the efficiency of GLUT4 vesicle transport and insertion into the plasma membrane.	• Tropomyosin Tpm3.1	Stimulation	• Tpm3.1 defines a specific population of cortical actin filaments<br>• These filaments recruit MyoIIA to generate localized contractile force<br>• Tpm3.1 inhibits the association of Myo1c with actin<br>• The balance between these two actin filament populations determines the efficiency of GLUT4 vesicle transport and fusion	Fusion	Unclear risk (Not reported)	Low risk	Unclear risk (Not reported)	Unclear risk (Not reported)	Unclear risk (Not reported)	Unclear risk (Not reported)	Unclear risk (Not reported)	Low risk	Low risk	Unclear risk (Not reported)	Unclear risk (Not reported)	Low risk	Unclear risk (Not reported)	Unclear risk (Not reported)	Low risk	
A.76	10.1152/japplphysiol.01484.2011	Bernard et al., 2012	United States (USA)<br>Taiwan	To investigate whether an amino acid mixture increases glucose uptake in perfused rat hindlimb skeletal muscle in the presence or absence of submaximal insulin concentrations, and whether this increase is associated with elevated plasma membrane GLUT4 content.	• Male Sprague-Dawley rats, approximately 9 weeks old	• Insulin: Submaximal concentration (200 μU/mL)<br>• Amino acids: 2 mM mixture (cysteine, methionine, valine, isoleucine, and leucine)<br>• Technique: Hindlimb perfusion technique	• Measurement of 2-deoxyglucose (2-DG) uptake<br>• Measurement of PI 3-kinase activity (IRS-1–associated and total)<br>• Western blot analysis (phosphorylation of AMPK, aPKC, and AS160)<br>• Plasma membrane fractionation to assess GLUT4 content	• Perfusion performed for a total of 25 minutes at 37°C<br>• Cumulative 2-DG uptake measured over 25 minutes<br>• Signaling proteins and plasma membrane GLUT4 analyzed from muscle tissue collected immediately after completion of perfusion	• Basal group: 6 mM carbohydrate only; no insulin, no amino acids<br>• AA group: Amino acids alone<br>• sINS group: Submaximal insulin alone	The beneficial effect of an amino acid mixture on skeletal muscle glucose uptake under submaximal insulin conditions is attributable to increased AS160 phosphorylation and plasma membrane–associated GLUT4, and is independent of PI 3-kinase and AMPK activation.	• Amino acids (Isoleucine, Leucine)	Stimulation	• Amino acid mixture enhances insulin-stimulated GLUT4 translocation<br>• Increased phosphorylation of AS160<br>• Increased plasma membrane–associated GLUT4<br>• Effect is independent of PI 3-kinase signaling<br>• Effect is independent of AMPK activation	Signaling	Unclear risk (Not reported)	Low risk	Unclear risk (Not reported)	Unclear risk (Not reported)	Unclear risk (Not reported)	Unclear risk (Not reported)	Unclear risk (Not reported)	Low risk	Low risk							
A.81	10.1186/1471-2121-9-25	Catherine A Heyward, 2008	United Kingdom	To identify and investigate the role of a potential lipid-binding motif within an intracellular loop of GLUT4 in insulin-stimulated vesicle translocation and membrane fusion.	• Mouse 3T3-L1 adipocytes<br>• L6 myoblasts	• Physiological stimulation: Insulin (83 nM)<br>• Genetic intervention: Plasmid transfection with GLUT4HA-GFP (wild type or 4A mutant, in which the SQWL motif was mutated to alanine)<br>• Pharmacological inhibition: Wortmannin (PI3K inhibitor; mentioned in the review section); Brefeldin A (ARF inhibitor)	• Phage display (to identify the phosphatidic acid–binding motif)<br>• Confocal microscopy and quantitative image analysis (Metamorph software)<br>• Cell-surface HA exposure assay (HA: GFP ratio) in nonpermeabilized cells	• 10 minutes after insulin stimulation (for microscopy)<br>• 30 minutes after insulin stimulation (for quantification of translocation)	• Wild-type (WT) versus 4A mutant (MUT)<br>• Basal state versus insulin-stimulated state	The potential phosphatidic acid–binding motif identified in this study is unique to GLUT4 among sugar transporters and may provide a distinct mechanism for regulating insulin-induced translocation via phospholipase D signalin	• Phosphatidic acid (PtdOH / phosphatidic acid)	Inhibition	• The SQWL motif within the first intracellular loop of GLUT4 (a region unique to GLUT4 within the GLUT family) functions as a phosphatidic acid (PtdOH)–binding motif<br>• Binding of PtdOH generated by phospholipase D induces conformational changes in GLUT4<br>• These changes facilitate fusion pore formation or enhance recognition of GLUT4 vesicles by regulatory proteins<br>• This process promotes the final membrane fusion step with the plasma membrane	Fusion										Unclear risk (Not reported)	Unclear risk (Not reported)	Low risk	Unclear risk (Not reported)	Unclear risk (Not reported)	Low risk	
A.83	10.1042/BST0320817	Zhou et al., 2004	United States	To use RNA interference (siRNA)–based gene silencing to analyze and identify key components of insulin signaling pathways that regulate the translocation of the glucose transporter GLUT4 to the plasma membrane.	• Fully differentiated mouse 3T3-L1 adipocytes	• Physiological stimulation: Insulin (basal and maximal concentrations)<br>• Genetic intervention: siRNA (single or pooled sets of four siRNAs) introduced by electroporation to deplete target proteins, including: Akt1, Akt2, PKC λ/ζ, SHIP2, PTEN, c-Cbl, Cbl-b, CAP, CrkII, phospholipase Cγ1, and others	• Radioactive [^{3}H]-deoxyglucose transport assay<br>• Western blot analyses to assess protein expression levels<br>• Affymetrix GeneChip microarray analysis<br>• Functional biological analysis (GLUT4 translocation)	• Cells incubated for 24–72 hours after siRNA electroporation prior to functional assays and protein measurements	• Scrambled (Scr) siRNA control group<br>• Comparison of silencing efficiency between different isoforms (e.g., Akt1 vs. Akt2; PKC λ vs. PKC ζ)<br>• Basal (no insulin) versus insulin-stimulated conditions	The study demonstrates that Akt2, but not Akt1 or PKC λ/ζ, is an essential downstream mediator in the PI3K pathway regulating insulin-stimulated GLUT4 translocation in adipocytes. PTEN is identified as a potent negative regulator of this pathway, whereas proposed alternative pathways such as TC10 or PLCγ are not essential for insulin-regulated GLUT4 trafficking.	• c-Cbl	Stimulation	• Akt2 is the key and indispensable downstream intermediate in the PI3K signaling pathway leading to GLUT4 translocation in adipocytes<br>• PTEN functions as a strong negative regulator of this pathway<br>• Alternative proposed pathways, including TC10 and PLCγ, are not essential for insulin-regulated GLUT4 translocation	Signaling										Unclear risk (Not reported)	Unclear risk (Not reported)	Low risk	Unclear risk (Not reported)	Unclear risk (Not reported)	Low risk	
A.85	10.1128/MCB.22.11.3599-3609.2002	Jun Liu, 2002	United States	To evaluate the functional role of the adaptor protein APS in insulin-responsive metabolic cells, specifically its ability to facilitate tyrosine phosphorylation of c-Cbl and GLUT4 translocation.	• Differentiated mouse 3T3-L1 adipocytes<br>• CHO cells overexpressing the human insulin receptor (CHO-IR)	• Physiological stimulation: Insulin (100 nM)<br>• Genetic interventions:<br>– Plasmid transfection (via electroporation or FuGene 6) to overexpress:<br>• Myc-tagged wild-type APS (Myc-APS/WT)<br>• APS mutant lacking tyrosine phosphorylation at residue 618 (APS/Y618F)<br>• Triple-HA-tagged c-Cbl and tyrosine mutants (Y371F, Y700F, Y731F, Y774F)<br>• FLAG-tagged CAP and deletion mutants (ΔSH3, ΔSoHo)<br>• Pharmacological inhibition: PP2 (Src kinase inhibitor)	• Immunoprecipitation (i.p.) and Western blot analysis<br>• Confocal fluorescence microscopy for protein localization<br>• Measurement of GLUT4-EGFP translocation to the plasma membrane<br>• In vitro GST pull-down assays	• 2 minutes after insulin stimulation (for phosphorylation signaling analyses)<br>• 30 minutes after insulin stimulation (for GLUT4 translocation analyses)	• Basal state (no insulin) versus insulin-stimulated state<br>• Empty vector–transfected control versus APS/WT or APS/Y618F transfection<br>• Comparison across different APS plasmid doses (dose-dependent analysis)	APS functions as an adaptor protein linking the CAP/Cbl pathway to the insulin receptor, and APS-facilitated insulin receptor–mediated tyrosine phosphorylation of c-Cbl is a critical event in insulin-stimulated glucose transport.	• APS<br>• c-Cbl<br>• CAP<br>• Crk<br>• Flotillin<br>• TC10<br>• C3G	Stimulation	• APS acts as an essential adaptor protein linking the insulin receptor to the CAP/Cbl pathway<br>• Insulin induces tyrosine phosphorylation of APS, creating a binding site for c-Cbl<br>• APS presents c-Cbl to the insulin receptor for tyrosine phosphorylation<br>• Tyrosine-phosphorylated c-Cbl recruits Crk/C3G<br>• Crk/C3G activation leads to TC10 activation<br>• This signaling pathway operates in parallel with PI3K and is required for GLUT4 translocation in adipocytes	Signaling										Unclear risk (Not reported)	Unclear risk (Not reported)	Low risk	Unclear risk (Not reported)	Low risk	Low risk	
A.87	10.1091/mbc.E10-04-0316	Chiu et al., 2010	Canada; United States	To test the hypothesis that insulin regulates the kinetics of actin remodeling through cycles of branching and depolymerization to stimulate GLUT4 translocation in muscle cells.	• Rat L6 muscle cells stably expressing myc-tagged GLUT4	• Physiological stimulation: Insulin (100 nM)<br>• Genetic interventions (siRNA knockdown):<br>– Arp2/3 complex subunits (Arp3, p34)<br>– Cofilin<br>– LIM kinase 1 (LIMK1)<br>– Slingshot-1 (SSH1)<br>• Protein re-expression:<br>– Arp3-GFP<br>– Cofilin-WT-GFP (Xenopus)<br>– Cofilin-S3E-GFP mutant<br>• Chemical inhibition:<br>– Latrunculin B (LB, 250 nM) to disrupt actin filaments	• Confocal laser-scanning microscopy to detect surface GLUT4myc and F-actin (rhodamine–phalloidin staining)<br>• Western blotting and protein ratio analysis (P-cofilin, P-Akt, P-LIMK1) using 1D and 2D electrophoresis<br>• [^{125}I]-transferrin recycling assay<br>• Quantification of fluorescence pixel intensity of remodeled actin at the dorsal cell surface (dorsal actin remodeling)	• Insulin stimulation for 10–30 minutes (cofilin phosphorylation kinetics measured at 0, 3, 5, 10, and 30 minutes)<br>• Data collected after 72 hours of siRNA treatment or 24 hours after cDNA transfection	• Basal state versus insulin-stimulated state<br>• Nonrelevant siRNA (siNR) versus target siRNA (siArp3, sip34, siCofilin, siSSH1)<br>• Wild-type cofilin versus S3E-cofilin mutant	Arp2/3 and cofilin coordinately regulate a dynamic cycle of actin branching and severing at the cell cortex, which is essential for insulin-mediated GLUT4 translocation in muscle cells.	• Arp2/3 complex (Arp3, p34)	Stimulation	• Insulin activates the Arp2/3 complex to promote actin branching and polymerization<br>• Accumulation of F-actin activates Slingshot-1<br>• Slingshot-1 dephosphorylates and activates cofilin<br>• Activated cofilin severs older actin filaments, releasing actin monomers<br>• Released monomers support subsequent rounds of actin polymerization<br>• The balance between actin polymerization (Arp2/3) and filament severing (cofilin) is essential to create a permissive environment for GLUT4 vesicle docking and fusio	Translocation										Unclear risk (Not reported)	Unclear risk (Not reported)	Low risk	Unclear risk (Not reported)	Unclear risk (Not reported)	Low risk	
A.88	10.1016/j.yexcr.2007.03.024	Ikonomov et al., 2007	United States	To investigate the role of the ArPIKfyve–PIKfyve complex and its lipid product phosphatidylinositol 3,5-bisphosphate (PtdIns 3,5-P_2) in insulin-stimulated GLUT4 translocation and glucose uptake in 3T3-L1 adipocytes.	• Mouse 3T3-L1 adipocytes	• Physiological stimulation: Insulin (100 nM)<br>• Genetic interventions:<br>– siRNA (Smart pool™)–mediated knockdown of PIKfyve<br>– siRNA-mediated knockdown of ArPIKfyve<br>– Combined knockdown of PIKfyve and ArPIKfyve<br>• Plasmid transfection: HA-GLUT4-eGFP or Myc-GLUT1-HA<br>• Stress condition: Hyperosmotic stress	• [^{3}H]-2-deoxyglucose uptake assay<br>• Fluorescence and confocal microscopy to assess GLUT4/GLUT1 translocation<br>• Cell-surface biotinylation of IRAP<br>• Immunoprecipitation and Western blot analysis<br>• Subcellular fractionation<br>• In vitro lipid labeling with [γ-^{32}P]ATP followed by HPLC/TLC analysis<br>• Measurement of PIKfyve lipid kinase and protein kinase activities	• Protein level assessment: 72 hours after siRNA transfection<br>• Glucose uptake / GLUT4 translocation: 20–30 minutes after insulin stimulation<br>• Protein–protein interaction analysis: 0–60 minutes after insulin stimulation<br>• Lipid labeling: 10–30 minutes after insulin stimulation	• Basal state (no insulin) versus insulin-stimulated state<br>• Control siRNA (cyclophilin B) versus target siRNA (PIKfyve or ArPIKfyve)<br>• Single knockdown versus combined knockdown of PIKfyve and ArPIKfyve	The study demonstrates for the first time a physical association between the functionally related proteins PIKfyve and ArPIKfyve in 3T3-L1 adipocytes and shows that the ArPIKfyve–PIKfyve–PtdIns 3,5-P_2 pathway is physiologically linked to insulin-activated GLUT4 translocation and glucose transport.	• PIKfyve–ArPIKfyve	Stimulation	• ArPIKfyve and PIKfyve form a stable physical complex in adipocytes, independent of insulin<br>• ArPIKfyve functions as an activator of PIKfyve lipid kinase activity<br>• The ArPIKfyve–PIKfyve–PtdIns 3,5-P_2 pathway represents a novel mechanism positively regulating GLUT4 trafficking from endosomal compartments to the plasma membrane<br>• This pathway is independent of the GLUT1 recycling pathway	Signaling										Unclear risk (Not reported)	Unclear risk (Not reported)	Low risk	Unclear risk (Not reported)	Unclear risk (Not reported)	Low risk	
A.91	10.1074/jbc.M111.226092	Koumanov et al., 2011	United Kingdom	To investigate whether the interaction between AS160 (TBC1D4) and 14-3-3 proteins participates in regulating the fusion of GLUT4 vesicles with the plasma membrane in response to insulin.	• Adipocytes isolated from male Wistar rats<br>• HEK293T cell line	• Physiological stimulation: Insulin (20 nM or 60 nM) and IGF1 (50 ng/mL)<br>• Genetic interventions: Electroporation-mediated transfection of recombinant protein constructs:<br>– Phosphotyrosine-binding (PTB) domains (single or tandem, residues 1–632)<br>– Truncated human AS160 variant (R363X)<br>– Overexpression of 14-3-3 proteins (β, γ, ε isoforms)<br>• Chemical inhibition: 14-3-3 inactivation, including R18 peptide (1–10 μM) and fusicoccin (1 μM)	• Cell-free in vitro GLUT4 vesicle fusion assay<br>• Immunoprecipitation and Western blot analysis<br>• GST fusion protein pull-down assays<br>• RT-qPCR to quantify mRNA levels of 14-3-3 isoforms<br>• Measurement of cell-surface HA-GLUT4 exposure using [illegible]	• Measurements at 5 minutes (in vitro fusion assay) or 20 minutes (cellular insulin/IGF1 stimulation)<br>• Data collected 5–18 hours after plasmid transfection	• Basal state versus insulin/IGF1-stimulated state<br>• Control groups (BSA or IgG) versus PTB domain constructs or 14-3-3 inhibitors<br>• Expression of full-length AS160 alone versus co-expression with PTB domain constructs	The PTB domains inhibit insulin-stimulated GLUT4 vesicle fusion by disrupting the interaction between AS160 and 14-3-3 proteins, rather than by inhibiting AS160 phosphorylation.	• 14-3-3 proteins (particularly the γ isoform)	Stimulation	• 14-3-3γ is identified as the principal isoform interacting with AS160 on GLUT4 vesicles<br>• The AS160–14-3-3 interaction, rather than AS160 phosphorylation alone, is required to convert AS160 into a GAP-inactive state<br>• This conversion permits Rab GTPases on GLUT4 vesicles to adopt the active GTP-bound state<br>• Active Rab GTPases enable the fusion of GLUT4 vesicles with the plasma membrane<br>• PTB domains act as competitive inhibitors of the AS160/14-3-3 interaction	Fusion										Unclear risk (Not reported)	Unclear risk (Not reported)	Low risk	Unclear risk (Not reported)	Unclear risk (Not reported)	Low risk	
A.93	10.1016/s0021-9258(19)84060-7	Kramer et al., 2006	United States	To determine the functional role of AS160 in regulating glucose uptake under basal conditions, insulin stimulation, and muscle contraction in mouse skeletal muscle in vivo.	• Tibialis anterior muscles of female ICR mice, 8 weeks old	• Physiological stimulation:<br>– Insulin (elicited via intravenous glucose injection, 1.0 g/kg, to induce endogenous insulin secretion)<br>– In situ muscle contraction (peroneal nerve electrical stimulation for 15 minutes)<br>• Genetic interventions: In vivo electroporation to overexpress DNA constructs:<br>– Wild-type AS160 (WT)<br>– AS160-4P mutant (nonphosphorylatable at four PAS sites)<br>– AS160-R/K mutant (Rab GAP–inactive)<br>– AS160-2M mutant (combined 4P and R/K mutations)	• In vivo [^{3}H]-2-deoxyglucose uptake assay<br>• Western blotting<br>• Measurement of blood glucose and muscle glycogen levels	• Protein expression assessed 7 days after gene delivery<br>• Glucose uptake measured over 45 minutes after tracer injection<br>• Muscle samples collected immediately after 15 minutes of contraction or insulin stimulation for signaling analyses	• Empty pCAGGS vector control<br>• Basal (sham) state versus insulin-stimulated or contraction-stimulated conditions<br>• Comparisons among different AS160 mutant constructs	AS160 regulates both insulin- and contraction-stimulated glucose metabolism in mouse skeletal muscle in vivo, and the effects of AS160 mutations on insulin and contraction responses are not identical, identifying AS160 as a critical convergence point for independent stimuli of skeletal muscle glucose uptake	• AS160 (TBC1D4)	Inhibition	• AS160 is identified as an essential convergence point for two independent signaling pathways: insulin (via Akt) and muscle contraction (via AMPK)<br>• Phosphorylation of AS160 inactivates its Rab GTPase–activating protein (GAP) domain<br>• Inactivation of the GAP domain relieves suppression of Rab proteins on GLUT4 vesicles<br>• This permits GLUT4 vesicle translocation and fusion, resulting in increased glucose uptake<br>• This study provides the first evidence that a single protein coordinates both insulin- and contraction-stimulated glucose uptake in skeletal muscle	Signaling	Unclear risk (Not reported)	Low risk	Unclear risk (Not reported)	Unclear risk (Not reported)	Unclear risk (Not reported)	Unclear risk (Not reported)	Unclear risk (Not reported)	Low risk	Low risk							
A.96	10.1042/BJ20050887	Miinea et al., 2005	United States	To determine whether AS160 is indeed a GTPase-activating protein (GAP) for Rab proteins, and if so, to define its Rab specificity.	• 3T3-L1 adipocytes<br>• HEK-293F cells<br>• Escherichia coli (strains BL21, Rosetta BL21, XL-1 Blue)	• Insulin treatment: Insulin (160 nM)<br>• Recombinant protein: GST-tagged AS160 GAP domain<br>• Point mutation: R973K mutation (arginine-to-lysine substitution at residue 973) to abolish GAP activity<br>• Pharmacological inhibition: LY294002 (PI3K inhibitor)	• Mass spectrometry (MS)<br>• Immunoblotting<br>• Rab GAP activity assays<br>• Thin-layer chromatography (TLC)	• 30 min after insulin treatment<br>• 0, 15, and 30 minutes after the addition of the GAP domain	• No insulin treatment<br>• Nonspecific rabbit immunoglobulin control<br>• R973K mutant (GAP-inactive control)<br>• Rab-only group (no GAP domain)	AS160 is identified as a functional GAP with specificity toward a small subset of Rab proteins (Rab2A, Rab8A, Rab10, and Rab14) that are present on GLUT4-containing vesicles.	• AS16	Inhibition	• AS160 functions as a Rab GTPase-activating protein for a restricted subset of Rab proteins (Rab2A, Rab8A, Rab10, Rab14)<br>• These Rab proteins are localized on GLUT4-containing vesicles<br>• Phosphorylation of AS160 by Akt inhibits its GAP activity<br>• Inhibition of GAP activity increases the pool of active Rab-GTP<br>• Increased Rab-GTP promotes GLUT4 vesicle translocation	Translocation										Unclear risk (Not reported)	Unclear risk (Not reported)	Low risk	Unclear risk (Not reported)	Unclear risk (Not reported)	Low risk	
A.99	10.1021/bi970202g	Shisheva et Czech, 1997	United States	To analyze cytosolic Rab4 complexes with GDI-1 or GDI-2 in intact cells in order to understand how insulin regulates Rab4 cycling.	• Differentiated 3T3-L1 adipocytes<br>• COS-1 cells<br>• CHO-T cells	• Physiological stimulation: Insulin (100 nM) for 10–20 minutes	• Cell fractionation<br>• Immunoprecipitation (IP)<br>• Western blotting<br>• ^{32}P radiolabeling	• Analysis at 10 minutes or 20 minutes after insulin stimulation	• No-insulin treatment (basal/resting cells)<br>• Preimmune or non-immune IgG used as controls for immunoprecipitation	Insulin selectively induces the formation of Rab4–GDI-1 complexes, a process that contributes to insulin-regulated membrane trafficking.	• GDP dissociation inhibitor (GDI)	Stimulation	• Insulin selectively promotes the formation of Rab4–GDI-1 complexes in the cytosol<br>• This process occurs independently of changes in phosphorylation<br>• Formation of Rab4–GDI-1 complexes facilitates the release of Rab4 from membranes into the cytosol<br>• This mechanism contributes to insulin-stimulated regulation of membrane trafficking	Translocation										Unclear risk (Not reported)	Unclear risk (Not reported)	Low risk	Unclear risk (Not reported)	Unclear risk (Not reported)	Low risk	
A.100	10.1210/en.138.6.2391	Cynthia Corley Mastick et Alaina L.Falick., 1997	United States	To investigate the role of fusion complexes containing NSF and SNAPs (soluble NSF attachment proteins) in GLUT4 membrane trafficking in primary mouse adipocytes.	• Primary mouse adipocytes	• Physiological stimulation: Insulin (10 nM) at 37 °C for 30 minutes	• Subcellular fractionation by differential centrifugation<br>• Immunoadsorption of GLUT4 vesicles<br>• Glycerol velocity gradient centrifugation<br>• Sucrose density gradient centrifugation<br>• SDS-PAGE and immunoblotting	• Analysis performed 30 minutes after insulin treatment	• Basal group<br>• Nonspecific antibody control	The data indicate that the steady-state association of NSF and SNAPs with GLUT4 vesicles and cellular membranes is independent of the formation of fusion complexes.	• NSF<br>• SNAPs	Stimulation	• NSF and α-SNAPs associate with GLUT4 vesicles and cellular membranes in a steady-state manner<br>• This association occurs independently of fusion complex formation<br>• NSF and α-SNAPs in the low-density microsome (LDM) fraction form large (~20S) ATP-sensitive complexes<br>• These complexes exclude v-SNARE proteins such as VAMP-2 and cellubrevin	Fusion<br>Docking										Unclear risk (Not reported)	Unclear risk (Not reported)	Low risk	Unclear risk (Not reported)	Unclear risk (Not reported)	Low risk	

A.101	10.1074/jbc.273.22.13808	Ayse G. Kayali et al., 1998	United States	To determine the functional significance of the interaction between phospholipase Cγ (PLCγ) and the insulin receptor (IR) in mediating insulin-stimulated GLUT4 translocation and glucose uptake.	• Differentiated 3T3-L1 adipocytes<br>• Primary mouse adipocytes	• Insulin treatment: 0.2–100 nM<br>• Pharmacological inhibition: PLC inhibitor U73122 (1–10 μM)<br>• Microinjection: Fusion proteins containing the SH2 domains of PLCγ	• Immunoprecipitation<br>• Western blotting<br>• 2-deoxyglucose uptake assay<br>• Fluorescence microscopy	• 1 minute after insulin stimulation (phosphorylation)<br>• 5 minutes (PI3K activity)<br>• 15 minutes (glucose uptake)<br>• 20–30 minutes (GLUT4 translocation)	• DMSO vehicle control<br>• Inactive analog U73343<br>• Basal (no insulin)<br>• Sheep IgG<br>• SH2 domains of SHC or GRB10	PLCγ is an active component of insulin receptor–mediated metabolic signaling, and potentially mitogenic signaling, in 3T3-L1 adipocytes.	• Phospholipase Cγ (PLCγ)	Stimulation	• PLCγ directly interacts with the insulin receptor via its SH2 domains<br>• This interaction contributes to insulin-stimulated signaling leading to GLUT4 translocation<br>• PLCγ functions as an active mediator in insulin receptor–dependent metabolic signaling	Signaling										Unclear risk (Not reported)	Unclear risk (Not reported)	Low risk	Unclear risk (Not reported)	Low risk	Low risk	
A.102	10.1073/pnas.0606881103	Jérôme Vicogme et al., 2006	United States	To investigate the requirement for acidic phospholipids in SNARE-dependent membrane fusion and to demonstrate the role of asymmetric distribution of phosphatidic acid (PA) and phosphatidylinositol 4,5-bisphosphate (PIP_2) between donor and acceptor membranes.	• In vitro reconstituted system using proteoliposomes	• Manipulation of phospholipid composition in donor and acceptor vesicles	• Lipid fluorescence mixing assay using NBD-PE and rhodamine-PE<br>• Hemifusion assay using sodium dithionite to quench outer leaflet fluorescence<br>• Membrane permeability assay using 5(6)-carboxyfluorescein<br>• Immunoblotting to determine protein levels	• Fluorescence measured every 2 minutes for 120 minutes<br>• Hemifusion index measured at 30 minut	• Vesicles containing phosphatidylcholine (PC) only<br>• Vesicles containing PC/phosphatidylserine (PS) without PA or PIP_2<br>• Homotypic fusion combinations (donor–donor or acceptor–acceptor)<br>• SNARE-free liposomes	The study demonstrates an unanticipated dependence of SNARE complex–mediated fusion on asymmetrically distributed acidic phospholipids and provides mechanistic insights into the roles of phospholipase D and PIP kinases in the late stages of regulated exocytosis.	• Phosphatidic acid (PA)	Stimulation	• GLUT4 vesicle fusion depends on asymmetric distribution of acidic phospholipids<br>• PA, which induces negative membrane curvature, is required in the acceptor membrane to stabilize the hemifusion intermediate<br>• PIP_2, which induces positive membrane curvature, is required in the donor membrane to overcome the rate-limiting step and drive progression from hemifusion to full fusion	Fusion										Unclear risk (Not reported)	Unclear risk (Not reported)	Low risk	Unclear risk (Not reported)	Unclear risk (Not reported)	Low risk	
A.104	10.1016/j.cellsig.2008.04.011	Halaby et al., 2008	United States	To investigate the function of ATM in insulin signaling, the mechanisms underlying insulin resistance, and GLUT4 translocation in muscle cells.	• Male Wistar rats (insulin resistance induced by a high-fat diet)<br>• Mouse embryonic fibroblast (MEF) cell lines A29 (ATM+/+) and A38 (ATM−/−)<br>• Rat L6 myoblast cell line	• Dietary intervention: High-fat diet (35% lard)<br>• Insulin treatment: In vivo (20 U); in vitro (100 nM)<br>• Pharmacological inhibition: ATM-specific inhibitor KU-55933 (10 μM)<br>• Gene transfection (overexpression):<br>– Wild-type ATM (WT-ATM)<br>– Kinase-dead ATM (KD-ATM)<br>– GLUT4myc	• Western blot analysis<br>• QUICKI index measurement (insulin sensitivity)<br>• ELISA<br>• 2-deoxyglucose (2-DG) incorporation assay<br>• Confocal microscopy and indirect immunofluorescence	• Weekly monitoring of blood glucose<br>• Muscle collection after 3 months of dietary intervention<br>• Akt phosphorylation measured 30 minutes after insulin injection in rats<br>• Akt phosphorylation measured 45 minutes after insulin treatment in L6 and MEF cells<br>• ATM inhibitor applied 1 hour prior to insulin treatment	• Rats fed a standard chow diet<br>• A29 cells (ATM+/+)<br>• Cells without ATM inhibitor treatment<br>• WT-ATM–transfected cells compared with KD-ATM–transfected cells	In L6 cells transfected with wild-type ATM, insulin induces a marked increase in cell surface GLUT4, whereas insulin-stimulated GLUT4 translocation is significantly inhibited in cells expressing kinase-dead ATM.	• ATM protein kinase	Stimulation	• ATM functions as a cytosolic protein kinase responsive to insulin<br>• ATM acts as an important upstream regulator required for full Akt activation<br>• ATM promotes Akt phosphorylation at Ser473 followed by Thr308<br>• Full Akt activation is required for regulation of GLUT4 translocation and glucose uptake in muscle cells	Signaling	Unclear risk (Not reported)	Low risk	Unclear risk (Not reported)	Unclear risk (Not reported)	Unclear risk (Not reported)	Unclear risk (Not reported)	Unclear risk (Not reported)	Low risk	Low risk	Unclear risk (Not reported)	Unclear risk (Not reported)	Low risk	Unclear risk (Not reported)	Unclear risk (Not reported)	Low risk	
A.106	10.3390/ijms221910753	Kiko Hasegawa et al., 2021	Japan	To investigate the physiological role of Rac1 in white adipocytes using adipocyte-specific Rac1 knockout mice (adipo-rac1-KO)	• Adipocyte-specific Rac1 knockout mice (adipo-rac1-KO) on a C57BL/6 genetic background<br>• Primary cultured mature white adipocytes isolated from subcutaneous white adipose tissue (sWAT)	• Genetic knockout: Rac1 deletion using the Adipoq-Cre system<br>• Physiological stimulation: Insulin (100 nM in vitro or intravenous injection in vivo)<br>• Expression of constitutively active mutants: Myr-p110α, Myr-Akt2, FLJ68ΔN, Rac1(G12V)<br>• Pharmacological inhibition: Akt2 inhibitor (AI-XII) and Rac1 inhibitor (RI-II)	• 2-deoxyglucose (2-DG) uptake assay<br>• Immunoblotting<br>• Measurement of GLUT4 translocation<br>• Fluorescence microscopy<br>• Quantitative RT-PCR	• 30 minutes after insulin stimulation (glucose uptake)<br>• 45 minutes after insulin stimulation (GLUT4 translocation / GTPase activation)<br>• 2 hours of inhibitor treatment<br>• Histological analysis in mice at 26 weeks of age	• Adipoq-Cre mice (control)<br>• Unstimulated cells (−)<br>• Cells expressing control proteins	Rac1 is involved in insulin-dependent glucose uptake and lipogenesis in white adipocytes, and reduced insulin responsiveness caused by Rac1 deficiency may explain the atrophy of white adipose tissues.	• Rac1	Stimulation	• Rac1 functions downstream of PI3K/Akt2 and upstream of RalA in insulin signaling in white adipocytes<br>• Rac1 is required for GLUT4 trafficking and lipogenesis<br>• Rac1 deficiency leads to impaired insulin responsiveness and white adipose tissue atrophy	Signaling	Unclear risk (Not reported)	Low risk	Unclear risk (Not reported)	Unclear risk (Not reported)	Unclear risk (Not reported)	Unclear risk (Not reported)	Unclear risk (Not reported)	Low risk	Low risk	Unclear risk (Not reported)	Unclear risk (Not reported)	Low risk	Unclear risk (Not reported)	Unclear risk (Not reported)	Low risk	
A.112	10.1016/j.cmet.2011.06.015	Xiangyang Xie et al., 2011	United States	To identify the function of CDP138, a C2 domain–containing phosphoprotein, in regulating insulin-stimulated GLUT4 translocation and glucose uptake.	• 3T3-L1 adipocytes<br>• HEK293T cells	• Physiological stimulation: Insulin (100 nM)<br>• Gene knockdown: CDP138 knockdown using RNA interference (siRNA)<br>• Overexpression: CDP138 mutants, including<br>– S197A mutant (loss of Akt phosphorylation site)<br>– C2 domain mutants lacking Ca^{2+}-binding capacity	• [³H]-2-deoxyglucose (2-DOG) uptake assay<br>• Total internal reflection fluorescence (TIRF) microscopy to monitor single-vesicle fusion events<br>• Western blot analysis and quantitative phosphoproteomics<br>• Measurement of cell surface GLUT4 using colorimetric or fluorescence-based assays (GLUT4-myc, GLUT4-GFP)	• 0–30 minutes after insulin stimulation	• Negative control siRNA (NC siRNA)<br>• Basal (no insulin)<br>• Wild-type (WT) CDP138 expression	CDP138 is a key molecule that links the Akt2 signaling pathway to the regulation of GLUT4 vesicle–plasma membrane fusion	• CDP138	Stimulation	• CDP138 functions as a molecular link between Akt2 signaling and GLUT4 vesicle fusion<br>• The C2 domain of CDP138 binds Ca^{2+} and lipid membranes<br>• This interaction facilitates the insertion of GLUT4 vesicles into the plasma membrane<br>• Vesicle insertion represents a rate-limiting step in insulin-stimulated GLUT4 translocation	Fusion										Unclear risk (Not reported)	Unclear risk (Not reported)	Low risk	Unclear risk (Not reported)	Unclear risk (Not reported)	Low risk	
A.113	10.1016/j.cellsig.2009.01.022	Maddalena Illario, 2009	Italy	To investigate the role of CaMKII in insulin signaling in a rat skeletal muscle cell line.	• Rat L6 skeletal muscle cells differentiated into myotubes	• Physiological stimulation: Insulin (1–100 nM)<br>• Pharmacological inhibition:<br>– KN93 (CaMKII inhibitor)<br>– antCaNtide (cell-permeable, specific CaMKII inhibitory peptide)	• Western blotting to assess phosphorylation and expression of proteins (Akt, Erk-1/2, CaMKII, IRS-1, GLUT4)<br>• Immunoprecipitation to examine protein–protein interactions (CaMKII with Raf-1; IRS-1 with p85 PI3K) and to measure kinase activities (Raf-1, CaMKII)<br>• [³H]thymidine incorporation assay to assess DNA synthesis (cell proliferation)<br>• [¹⁴C]-2-deoxyglucose uptake assay<br>• Subcellular fractionation to isolate plasma membrane fractions and assess GLUT4 translocation	• Protein signaling (Western blot/IP): 0, 5, 15, 30, 60, and 120 minutes after insulin stimulation<br>• DNA synthesis: 24 hours after insulin stimulation<br>• Glucose uptake: 0, 30, 60, and 120 minutes after insulin stimulation	• Basal (0 min, no stimulation)<br>• Insulin alone<br>• Insulin + CaMKII inhibitors (KN93 or antCaNtide)<br>• Insulin + reversed control peptide (R-antCaNtide)	CaMKII modulates insulin action on DNA synthesis and regulates a negative feedback mechanism that downregulates glucose uptake. CaMKII controls insulin-induced Erk-1/2 activation and cell proliferation, and following initial IRS-1/Akt pathway activation, CaMKII mediates negative feedback that reduces insulin-stimulated glucose uptake.	• CaMKII (α, β, γ isoforms)	Inhibition	• CaMKII interacts with and activates Raf-1, leading to Erk-1/2 activation and increased cell proliferation<br>• Erk-1/2, activated downstream of CaMKII, phosphorylates IRS-1 at Ser612<br>• This phosphorylation impairs IRS-1 association with PI3K<br>• Reduced PI3K signaling attenuates Akt activation and decreases GLUT4 abundance at the plasma membrane<br>• This constitutes a negative feedback loop controlling insulin-stimulated glucose uptak	Signaling										Unclear risk (Not reported)	Unclear risk (Not reported)	Low risk	Unclear risk (Not reported)	Unclear risk (Not reported)	Low risk	
A.114	10.1016/j.bbrc.2005.07.056	Susan Kane, 2005	United States	To identify proteins that interact with the GAP domain of AS160 and to determine the role of calmodulin binding to AS160 in insulin-stimulated GLUT4 translocation.	• Mouse 3T3-L1 adipocytes	• Physiological stimulation: Insulin (160 nM)<br>• Genetic mutation: Point mutation in AS160 to disrupt calmodulin binding (LW/GG mutant)<br>• Calcium manipulation: Alteration of Ca^{2+} levels by addition of $CaCl_2$ or chelation with EGTA	• Yeast two-hybrid screening to identify interacting proteins<br>• Biotinylated calmodulin binding assay to assess direct binding<br>• GST pull-down assays to examine protein–protein interactions in vitro<br>• Co-immunoprecipitation to assess protein interactions in vivo<br>• Single-cell fluorescence assay to quantify GLUT4 translocation to the plasma membrane	• Co-immunoprecipitation performed 10 minutes after insulin stimulation<br>• Binding assays incubated for 1 hour or 2 hours	• Basal versus insulin-stimulated conditions<br>• Presence of Ca^{2+} (+Ca^{2+}) versus Ca^{2+}-free conditions (−Ca^{2+}/EGTA)<br>• Wild-type AS160 versus mutant constructs (GG, 4P, 4P/GG)	AS160 directly binds calmodulin in a Ca^{2+}-dependent manner, but this interaction is not altered by insulin stimulation. Disruption of calmodulin binding does not impair insulin-stimulated GLUT4 translocation, indicating that calmodulin binding is not required for AS160 function in this process.	• AS160	No effect	• AS160 is identified as a calmodulin-binding protein<br>• The calmodulin-binding site is located in a small region immediately N-terminal to the GAP domain of AS160 (amino acids 834–857)<br>• Calmodulin binding is Ca^{2+}-dependent but insulin-independent<br>• Disruption of calmodulin binding does not affect insulin-stimulated GLUT4 translocation<br>• Thus, calmodulin binding is not required for AS160 function in insulin signaling related to GLUT4 translocation	Signaling										Low risk	Unclear risk (Not reported)	Low risk	Unclear risk (Not reported)	Unclear risk (Not reported)	Low risk	
A.115	10.2337/db07-0681	Henning F. Kramer, 2007	United States	To determine whether the calmodulin-binding domain (CBD) of AS160 regulates contraction-stimulated glucose uptake in mouse skeletal muscle, based on the hypothesis that contraction-induced Ca^{2+}/calmodulin signaling may modulate AS160 function.	• Female ICR mice (tibialis anterior muscles)	• In vivo gene transfer: Injection and electroporation of AS160 cDNA plasmids into muscle, including empty vector, wild-type AS160, CBD-mutant AS160, 4P mutant (nonphosphorylatable at four PAS sites), and double mutants (CBD+4P, CBD+R/K)<br>• Insulin stimulation: Intravenous glucose injection (1.0 g/kg) to induce physiological insulin secretion<br>• Muscle contraction: In situ electrical stimulation of the peroneal nerve for 15 minutes	• Immunoprecipitation of myc-tagged AS160 to assess calmodulin association<br>• Biotinylated calmodulin-binding assay to evaluate AS160–calmodulin interaction in vitro<br>• In vivo [³H]-2-deoxyglucose uptake assay<br>• Western blot analysis to measure AS160 expression and PAS phosphorylation<br>• Muscle glycogen measurement	• Protein expression assessed 7 days after DNA injection/electroporation<br>• Glucose uptake and blood sampling at 0, 5, 10, 15, 25, 35, and 45 minutes after glucose injection<br>• Muscle collection at 45 minutes (glucose uptake experiments) or after 15 minutes of contraction	• Empty vector–injected muscles versus AS160-transfected muscles (wild-type or mutants)<br>• Basal (saline/sham) versus stimulated conditions (insulin/glucose or contraction)<br>• Comparisons among AS160 mutants: CBD-mutant, 4P-mutant, CBD+4P, CBD+R/K	The calmodulin-binding domain of AS160 is essential for contraction-stimulated glucose uptake but is not required for insulin-stimulated glucose uptake. Muscle contraction regulates AS160 via Ca^{2+}/calmodulin signaling, whereas insulin acts through Akt-mediated phosphorylation.	• Calmodulin-binding domain (CBD) of AS160	Stimulation	• The calmodulin-binding domain of AS160 directly regulates contraction-stimulated glucose uptake but does not participate in insulin signaling<br>• Calmodulin provides an additional mechanism to modulate AS160 Rab-GAP function independently of phosphorylation<br>• During muscle contraction, binding of AS160 to calmodulin is required to inhibit AS160 GAP activity<br>• Inhibition of GAP activity relieves suppression of GLUT4 vesicle trafficking, allowing maximal glucose uptake	Signaling	Unclear risk (Not reported)	Low risk	Unclear risk (Not reported)	Unclear risk (Not reported)	Unclear risk (Not reported)	Unclear risk (Not reported)	Unclear risk (Not reported)	Unclear risk (Not reported)	Low risk	Unclear risk (Not reported)	Unclear risk (Not reported)	Unclear risk (Not reported)	Unclear risk (Not reported)	Unclear risk (Not reported)	Unclear risk (Not reported)	
A.116	10.1042/BJ20030681	David S. Paul, 2003	United States	To investigate the mechanism by which calpain influences insulin-stimulated glucose uptake in 3T3-L1 adipocytes.	• Differentiated mouse 3T3-L1 adipocytes	• Physiological stimulation: Insulin (100 nM or 1 μg/mL)<br>• Pharmacological inhibition of calpain:<br>– ALLN (100 μM)<br>– Leupeptin (100 μM)<br>– Pepstatin A (100 μM)<br>• Genetic inhibition: Tetracycline-inducible antisense CAPN10 (TET-ON system) to reduce CAPN10 protein expression	• Glucose uptake assay using [1-¹⁴C]-2-deoxy-D-glucose<br>• Calpain activity assay using a fluorescent substrate<br>• Immunofluorescence microscopy to visualize GLUT4 and actin (rhodamine-labeled phalloidin)<br>• Plasma membrane lawn assay to quantify GLUT4 at the cell surface<br>• Subcellular fractionation to isolate plasma membrane (PM) and low-density microsomes (LDM)<br>• Western blotting and immunoprecipitation to analyze protein expression (GLUT4, CAPN10, actin) and phosphorylation of insulin signaling components (IR, IRS-1, IRS-2, Akt)	• Glucose uptake measured after 10 minutes of insulin stimulation<br>• Calpain inhibitor treatment for 4 hours prior to measurements<br>• Antisense CAPN10 induction with doxycycline for 72 ho	• Basal (untreated) cells<br>• Insulin-stimulated cells<br>• Insulin + calpain inhibitors (ALLN, leupeptin, or pepstatin A)<br>• Antisense CAPN10 cells with doxycycline (+DOX) versus without doxycycline (−DOX)	Inhibition of calpain activity reduces insulin-stimulated glucose uptake by approximately 60% and prevents GLUT4 translocation to the plasma membrane without altering total GLUT4 protein levels. Although calpain inhibition does not affect proximal PI3K-dependent insulin signaling, it blocks insulin-stimulated cortical actin reorganization required for GLUT4 translocation. Specific inhibition of calpain-10 similarly reduces GLUT4 translocation and actin remodeling.	• Calpain (particularly Calpain-10 / CAPN10)	Stimulation	• Calpain, specifically calpain-10, is essential for insulin-stimulated cortical actin reorganization<br>• Actin remodeling is required for GLUT4 vesicle translocation to the plasma membrane<br>• This mechanism operates independently of proximal PI3K-dependent insulin signaling	Translocation										Unclear risk (Not reported)	Unclear risk (Not reported)	Unclear risk (Not reported)	Unclear risk (Not reported)	Unclear risk (Not reported)	Unclear risk (Not reported)	
A.117	10.1016/j.bbrc.2022.03.152	Sasa Liu, 2022	China	To investigate the relationship between CaMKII, Akt, and contraction-stimulated glucose uptake, specifically testing the hypothesis that CaMKII functions upstream of Rac1 and Akt in this process.	• Mouse C2C12 skeletal muscle cells (C2C12 myoblasts) differentiated into myotubes	• Electrical pulse stimulation (EPS): To mimic muscle contraction (20 V, 1 Hz, 60 minutes)<br>• Gene expression: Transfection with constitutively active CaMKII mutant (CaMKII-T286D)<br>• siRNA-mediated knockdown: CaMKII and Kalirin<br>• Pharmacological inhibition:<br>– Rac1 inhibitor II (Rac1 inhibition)<br>– KN-93 (CaMKII inhibition)<br>– ITX3 (Kalirin inhibition)<br>– MK2206 (Akt inhibition)	• Immunoblotting (Western blot) to measure phosphorylation of Akt (Ser473, Thr308), CaMKII (Thr286), and PAK1/2, and total protein levels of Kalirin and Actinin-1<br>• 2-NBDG uptake assay to quantify cellular glucose uptake using a fluorescent glucose analog	• EPS applied for 60 minutes<br>• Glucose uptake measured throughout the 1-hour EPS period<br>• Pharmacological inhibitors applied 30 minutes to 1 hour prior to EPS (or 20 hours for ITX3) and maintained during stimulation<br>• Measurements performed 48 hours after transfection	• Basal (−EPS) versus EPS-stimulated (+EPS) cells<br>• Vehicle controls (DMSO/vector) versus inhibitor-treated or transfected groups (KN-93, ITX3, CaMKII-T286D)<br>• Control siRNA (siNR) versus target-specific siRNA (siCaMKII, siKalirin)	EPS induces Akt activation via a CaMKII–Kalirin–Rac1 signaling pathway, which contributes to the regulation of contraction-stimulated glucose uptake in muscle cells. Akt functions downstream of this pathway and plays a partial role in contraction-induced glucose uptake.	• CaMKII (particularly the δ isoform)	Stimulation	• Muscle contraction activates CaMKII<br>• CaMKII activates Kalirin, a guanine nucleotide exchange factor for Rac<br>• Kalirin activates Rac1<br>• Rac1 promotes Akt phosphorylation and activation<br>• Activated Akt partially mediates contraction-stimulated glucose uptake	Signaling										Low risk	Unclear risk (Not reported)	Low risk	Unclear risk (Not reported)	Unclear risk (Not reported)	Low risk	
A.118	10.1016/j.cmet.2008.09.011	Ming Fai Yip, 2008	Australia	To identify insulin-regulated phosphoproteins through their interaction with 14-3-3 proteins and to uncover the role of the molecular motor Myo1c and its phosphorylation mechanism in GLUT4 translocation in adipocytes.	• 3T3-L1 adipocytes<br>• CHO cells expressing insulin receptor and IRS-1 (CHO/IR/IRS-1 cells)	• Stimulation: Insulin (100 nM); Ca^{2+} ionophore (A23187)<br>• Pharmacological inhibition:<br>– Wortmannin (PI3K inhibitor)<br>– KN-62 (CaMKII inhibitor)<br>– tat-CN21 (specific CaMKII inhibitory peptide)<br>– BAPTA-AM (intracellular Ca^{2+} chelator)<br>• Genetic interventions:<br>– Expression of Myo1c mutants: S701A (non-phosphorylatable), IQ1A (calmodulin-binding deficient), K111A (ATPase-dead)<br>– siRNA-mediated knockdown of CaMKIIδ and Myo1c	• 14-3-3 pulldown followed by mass spectrometry to identify interacting proteins<br>• Western blotting and immunoprecipitation to assess protein interactions (Myo1c with 14-3-3 or calmodulin) and phosphorylation status<br>• In vitro kinase assays to determine CaMKII-mediated phosphorylation of Myo1c<br>• In vitro ATPase assay to measure Myo1c motor activity<br>• Glucose uptake assay using 2-deoxyglucose<br>• Immunofluorescence (surface HA staining) to quantify GLUT4 translocation	• Insulin treatment typically 15–30 minutes (30 minutes for 14-3-3 pulldown; 15 minutes for glucose uptake)<br>• Inhibitor preincubation ranging from 10 minutes (wortmannin) to 45 minutes (KN-62)<br>• Genetic interventions (siRNA/transfection) assessed after 72 hours	• Basal (unstimulated) versus insulin-stimulated conditions<br>• Wild-type (WT) Myo1c versus Myo1c mutants (S701A, IQ1A, K111A, S701A/IQ1A)<br>• Control treatments (scrambled siRNA, empty vector, control peptide) versus targeted interventions (specific siRNA, mutants, inhibitory peptides)	Insulin stimulates phosphorylation of the motor protein Myo1c at Ser701, which enhances its association with 14-3-3 and reduces calmodulin binding. CaMKII phosphorylates Myo1c, increasing its ATPase activity, and Myo1c phosphorylation and motor activity are required for insulin-stimulated GLUT4 translocation to the plasma membrane.	• Myo1c (Myosin 1c)	Stimulation	• Insulin activates CaMKII via localized Ca^{2+} signaling<br>• CaMKII directly phosphorylates Myo1c at Ser701<br>• Phosphorylation reduces calmodulin binding and enhances 14-3-3 association<br>• Phosphorylated Myo1c exhibits increased ATPase (motor) activity<br>• Myo1c motor activity is required to drive GLUT4 vesicle translocation to the plasma membrane	Translocation										Unclear risk (Not reported)	Unclear risk (Not reported)	Low risk	Unclear risk (Not reported)	Unclear risk (Not reported)	Low risk	
A.127	10.1074/jbc.M208904200	Usui et al., 2003	United States (USA	To investigate the role of cdc42, a member of the Rho GTPase family, in insulin-stimulated glucose transport in 3T3-L1 adipocytes, and to determine its position within the signaling cascade involving Gαq/11 and phosphoinositide 3-kinase (PI3-kinase).	• Differentiated 3T3-L1 adipocytes	• Physiological stimulation: Insulin (0.5 nM, 1.7 nM, 17 nM)<br>• Inhibition of cdc42 function:<br>– Microinjection of anti-cdc42 antibody<br>– siRNA targeting cdc42<br>• Genetic manipulation:<br>– Expression of dominant-negative cdc42 (DN-cdc42)<br>– Adenoviral expression of constitutively active forms of cdc42 (CA-cdc42), Gαq (CA-Gαq), and PKCλ (CA-PKCλ)<br>• PI3-kinase inhibition: Wortmannin or LY294002<br>• PKC inhibition:<br>– Microinjection of anti-PKCλ antibody<br>– Expression of kinase-deficient PKCλ	• GLUT4 translocation: Immunofluorescence staining and quantification of cells displaying GLUT4 rings at the plasma membrane<br>• Glucose uptake: Measurement of 2-[³H]deoxyglucose (2-DOG) uptake<br>• Protein activity and interaction: Western blotting, PI3-kinase activity assays from immunoprecipitates, and cdc42-GTP pull-down assays	• GLUT4 translocation: 20 minutes after insulin stimulation<br>• Glucose uptake: 30 minutes after insulin stimulation<br>• Genetic manipulation efficacy: 48 hours after adenoviral infection or 72 hours after siRNA transfection<br>• cdc42 activity: measured at 0, 1, 3, 10, and 30 minutes after insulin stimulation	• Basal (non-stimulated) cells<br>• Cells injected with sheep IgG (control IgG) or control siRNA<br>• Cells infected with adenovirus expressing GFP (control virus)	cdc42 mediates insulin-stimulated GLUT4 translocation and glucose transport in a PI3-kinase–dependent manner. Insulin and constitutively active Gαq enhance cdc42 activity and its association with activated PI3-kinase; cdc42 functions downstream of Gαq/11 and upstream of PI3-kinase and PKCλ in this signaling pathway.	• Cdc42	Stimulation	• cdc42 is identified as a signaling molecule positioned downstream of Gαq/11 and upstream of PI3-kinase and PKCλ<br>• cdc42 activates PI3-kinase through direct interaction with the p85 regulatory subunit<br>• Activation of PI3-kinase leads to PKCλ activation and subsequent GLUT4 translocation<br>• This pathway differs from previous models in which Rho GTPases (e.g., TC10) were proposed to function independently of PI3-kinase	Signaling										Unclear risk (Not reported)	Unclear risk (Not reported)	Low risk	Unclear risk (Not reported)	Unclear risk (Not reported)	Low risk	
A.128	10.1074/jbc.M806531200	Okada et al., 2008	Japan	To examine CDK5-dependent phosphorylation of TC10α and to demonstrate that this event is required for regulating TC10α function, subcellular localization, and cortical F-actin reorganization in adipocytes.	• Differentiated 3T3-L1 adipocytes	• Physiological stimulation: Insulin (100 nM)<br>• Genetic manipulation:<br>– Transfection with plasmids expressing wild-type TC10α (WT)<br>– Nonphosphorylatable mutant TC10α (T197A)<br>– Phosphomimetic mutant TC10α (T197D)<br>• CDK5 modulation:<br>– Overexpression of CDK5/p35<br>– siRNA-mediated knockdown of CDK5<br>• Pharmacological inhibition: CDK5 inhibitor olomoucine	• GLUT4 translocation: Quantitative colorimetric assay detecting surface GLUT4-myc and confocal fluorescence microscopy<br>• Protein interaction and phosphorylation: Immunoprecipitation and Western blotting<br>• Kinase activity: In vitro CDK5 kinase assays<br>• TC10 activity: GTP-loading/pull-down assay using GST-Pak1-PBD<br>• Subcellular fractionation: Isolation of lipid raft (caveolae) and cytoskeletal fractions to assess actin organization	• Phosphorylation and activity assessment: 5 and 15 minutes after insulin stimulation<br>• GLUT4 translocation assessment: 30 minutes after insulin stimulation	• Basal (non-insulin-stimulated) cells<br>• Cells transfected with empty vector (pcDNA3) or scrambled siRNA	For GLUT4 translocation to occur, TC10α must be dephosphorylated at Thr197, allowing cortical actin to be reassembled. CDK5-dependent phosphorylation maintains TC10α in lipid raft compartments and disrupts cortical actin, whereas subsequent dephosphorylation permits F-actin reassembly required for insulin-stimulated GLUT4 translocation.	• TC10α (Rho GTPase)	Inhibition	• Insulin activates CDK5 (via Fyn)<br>• CDK5 phosphorylates TC10α at Thr197<br>• Phosphorylated TC10α is retained in lipid raft (caveolin-enriched) domains and promotes cortical actin depolymerization<br>• This actin state inhibits GLUT4 translocation<br>• Subsequent dephosphorylation of TC10α (via CDK5 inactivation) allows cortical actin re-polymerization<br>• Actin reassembly permits GLUT4 vesicle docking and fusion	Signaling										Unclear risk (Not reported)	Unclear risk (Not reported)	Low risk	Unclear risk (Not reported)	Unclear risk (Not reported)	Low risk	
A.135	10.1042/0264-6021:3630167	Fryer et al., 2002	United Kingdom (UK) and France	To determine the role of AMP-activated protein kinase (AMPK) in stimulating glucose transport and protein expression in a skeletal muscle cell line	• Mouse H-2Kᵇ skeletal muscle cell line	• Gene expression: Adenoviral infection expressing constitutively active AMPK (CA-AMPK) or dominant-negative AMPK (DN-AMPK)<br>• Chemical treatment / stress:<br>– 5-amino-imidazolecarboxamide riboside (AICAR, 500 μM)<br>– Sorbitol (hyperosmotic stress, 0.6 M)<br>– Insulin (100 nM)<br>– Phorbol 12-myristate 13-acetate (PMA, 1 μM)	• Glucose uptake: Measurement of 2-deoxy-D-[1-³H]glucose uptake<br>• GLUT localization: Cell-surface photoaffinity labeling with biotinylated bis-glucose photolabel (Bio-LC-ATB-BGPA) followed by Western blotting to detect GLUT1 and GLUT4 at the plasma membrane<br>• Protein expression: Western blotting to quantify total protein levels of GLUT1, GLUT4, hexokinase II, and related proteins<br>• Kinase activity: Measurement of ³²P incorporation into the SAMS peptide (AMPK activity assay)	• Glucose uptake assessed after 30 minutes of treatment (insulin, AICAR, sorbitol, PMA)<br>• Protein expression (long-term effects) assessed after 5 days of adenoviral expression of CA-AMPK<br>• Cell-surface photolabeling performed after 2 days of adenoviral infection	• Uninfected (control) cells<br>• Cells infected with null (empty) adenovirus<br>• Cells infected with alternative AMPK variants for comparison	Activation of AMPK is sufficient to stimulate glucose uptake in skeletal muscle cells and is required for glucose uptake induced by AICAR and hyperosmotic stress. In contrast, AMPK is not required for insulin- or phorbol-ester–mediated glucose transport. Long-term activation of AMPK increases protein expression of GLUT1, GLUT4, and hexokinase II, consistent with adaptive responses to exercise.	• AMPK (AMP-activated protein kinase)	Stimulation	• Acute effects: Activation of AMPK is sufficient to stimulate glucose transport by increasing translocation of GLUT1 and GLUT4 to the plasma membrane<br>• AMPK is required for glucose uptake induced by AICAR and hyperosmotic stress<br>• AMPK is not involved in insulin- or PMA-mediated glucose transport pathways<br>• Chronic effects: Prolonged AMPK activation (5 days) increases protein expression of GLUT1, GLUT4, and hexokinase II, mimicking exercise-induced muscle adaptation	Signaling										Unclear risk (Not reported)	Unclear risk (Not reported)	Low risk	Unclear risk (Not reported)	Unclear risk (Not reported)	Low risk	
A.139	10.1091/mbc.E14-09-1368	Sadler et al., 2015	United Kingdom	To quantify the expression levels, subcellular distribution, and interactions of all VAMP isoforms expressed in 3T3-L1 adipocytes in order to identify the principal v-SNARE involved in GLUT4 trafficking.	• 3T3-L1 adipocytes differentiated from fibroblasts	• Physiological stimulation: Insulin treatment (1 μM)<br>• Differentiation comparison: Fibroblasts (Day 0) versus adipocytes (Day 8)	• Protein quantification: Quantitative immunoblotting using recombinant protein standards to calculate molecules per cell<br>• Subcellular fractionation: Isolation of plasma membrane (PM), low-density membranes (LDM), and high-density membranes (HDM); iodixanol gradient fractionation to separate GLUT4 storage vesicles (GSVs) and endosomes<br>• Protein interactions: Immunoprecipitation and in vitro SNARE complex assembly assays	• Insulin stimulation: 20 or 30 minutes<br>• Differentiation stages: Day 0 and Day 8	• Basal versus insulin-stimulated conditions<br>• Comparison among VAMP isoforms (VAMP2, VAMP3, VAMP4, VAMP5, VAMP7, VAMP8)<br>• Comparison between fibroblasts and differentiated adipocytes	VAMP2 is the principal v-SNARE involved in GLUT4 trafficking to the plasma membrane in 3T3-L1 adipocytes. Although all VAMP isoforms can form stable SNARE complexes with Syntaxin4 and SNAP23 in vitro, expression levels, subcellular localization, and co-immunoprecipitation analyses indicate that VAMP2 uniquely fulfills this role in vivo.	• VAMP isoforms (VAMP2, VAMP3, VAMP4, VAMP5, VAMP7, VAMP8)	Stimulation	• VAMP2 is enriched in GLUT4 storage vesicles (GSVs)<br>• Insulin stimulation promotes physical interaction between VAMP2 and Syntaxin4<br>• VAMP3 is predominantly localized to endosomes<br>• VAMP8 does not respond to insulin stimulation<br>• Although multiple VAMPs can form SNARE complexes in vitro, only VAMP2 exhibits the appropriate expression level, localization, and insulin-responsive interaction required for GLUT4 vesicle fusion	Membrane Fusion										Unclear risk (Not reported)	Unclear risk (Not reported)	Low risk	Unclear risk (Not reported)	Unclear risk (Not reported)	Low risk	
A.146	10.1006/bbrc.1996.0474	Tamori et al., 1996	Japan	To investigate the role of synaptobrevin isoforms (VAMP-2 and cellubrevin) in insulin-stimulated GLUT4 translocation by using Botulinum neurotoxin B (BoNTx/B) to cleave these proteins in permeabilized 3T3-L1 adipocytes.	• 3T3-L1 adipocytes (differentiated from fibroblasts)<br>• Cells permeabilized with Streptolysin-O (SLO)	• Toxin treatment: Botulinum neurotoxin B (BoNTx/B); 150 nM<br>• Physiological stimulation: Insulin (10^{-7} M)	• Western blotting: Detection of VAMP-2, cellubrevin, and GLUT4 in subcellular fractions (LDM, PM) and assessment of toxin-mediated protein cleavage<br>• Immunoprecipitation: Isolation of GLUT4-containing vesicles using monoclonal antibody 1F8<br>• Glucose transport assay: Measurement of glucose transport using 2-deoxy-D-[1,6-³H]-glucose	• BoNTx/B incubation: ~20 minutes (optimal to preserve insulin responsiveness)<br>• ~60 minutes (to assess time-dependent cleavage)<br>• Insulin stimulation: 20 minutes	• Permeabilized 3T3-L1 adipocytes without BoNTx/B treatment<br>• Comparison between basal and insulin-stimulated conditions, with and without BoNTx/B	GLUT4-containing vesicles in 3T3-L1 adipocytes contain two synaptobrevin isoforms, VAMP-2 and cellubrevin, but not VAMP-1. These synaptobrevin isoforms are important components of insulin-dependent GLUT4 translocation to the cell surface.	• VAMP-2<br>• Cellubrevin (a synaptobrevin isoform, commonly referred to as VAMP-3)	Stimulation	Cleavage of VAMP-2 and cellubrevin by BoNTx/B reduces insulin-stimulated GLUT4 translocation to the plasma membrane and decreases glucose uptake, demonstrating that these synaptobrevin isoforms (v-SNAREs) are essential functional components of the insulin-regulated GLUT4 trafficking machinery in adipocytes.	Membrane Fusion										Unclear risk (Not reported)	Unclear risk (Not reported)	Low risk	Unclear risk (Not reported)	Unclear risk (Not reported)	Low risk	
A.148	10.1074/jbc.M109.060236	Ng et al., 2010	Australia	To resolve the apparent discrepancy between modest changes in Akt activity/expression and severe metabolic dysfunction, by examining insulin regulation of signaling cascade components using quantitative kinetic analysis, dose–response analysis, and hierarchical cluster analysis	• 3T3-L1 adipocytes (differentiated from fibroblasts)	• Physiological stimulation:<br>– Insulin (0–100 nM, multiple time points)<br>– PDGF (for comparison with insulin signaling)<br>• Pharmacological inhibition:<br>– Akt inhibitor (Akti-1/2)<br>– Rapamycin (mTOR inhibitor)<br>• Genetic manipulation:<br>– Expression of HA-GLUT4<br>– Expression of PDGF receptor (PDGFR)<br>– Expression of AS160 mutants (FLAG-tagged)<br>– Expression of PKCζ mutant	Western blotting; immunofluorescence microscopy; total internal reflection fluorescence (TIRF) microscopy; subcellular fractionation; hierarchical cluster analysis.	• Dose–response: Insulin stimulation for 20 minutes<br>• Time course: Measurements at 0, 15, 30, 60, 300, and 1800 seconds	• Total cellular Akt (whole-cell lysate) vs plasma membrane–localized Akt<br>• Akt substrates (AS160, GSK3, FoxO, TSC2) vs Akt phosphorylation<br>• Phosphorylation sites on AS160: Thr642 vs Ser588	• The spatial localization of Akt, rather than total cellular Akt abundance, determines its functional relevance; plasma membrane–localized Akt is critical for GLUT4 translocation, whereas total cellular Akt is not.<br>• Hierarchical cluster analysis showed that plasma membrane Akt substrates and GLUT4 translocation cluster together, while total Akt clusters separately. Microscopy further demonstrated that membrane-associated Akt exhibits an activity pattern distinct from that of total cellular Akt.<br>• AS160 is regulated by two distinct mechanisms at two separate phosphorylation sites:<br>– Thr642 is a direct Akt substrate and is enriched at the plasma membrane<br>– Ser588 segregates from the Akt substrate cluster and the GLUT4 cluster; experimental evidence indicates that this site is phosphorylated by atypical PKCζ, not Akt.	Akt, AS160 (TBC1D4)	AKT: Stimulation<br>AS160: Inhibition	• Spatial role of Akt: Total cellular Akt phosphorylation correlates poorly with GLUT4 translocation, whereas plasma membrane Akt phosphorylation correlates strongly with GLUT4 translocation and AS160 Thr642 phosphorylation.<br>• Mechanism of AS160 phosphorylation: AS160 contains two distinct phosphorylation sites regulated by different kinases. Thr642 is phosphorylated by Akt and is associated with GLUT4 translocation, whereas Ser588 is phosphorylated by atypical PKC (not Akt), and the two phosphorylation events are mutually exclusive.<br>• Lipid rafts: Thr642-phosphorylated AS160 is highly enriched in detergent-insoluble lipid rafts.	Signaling										Unclear risk (Not reported)	Unclear risk (Not reported)	Low risk	Unclear risk (Not reported)	Unclear risk (Not reported)	Low risk	
A.150		Jong-Sik Hah, 2000	Korea	To investigate the phosphorylation of different GLUT isoforms by PKC-ζ (a downstream effector of phosphatidylinositol 3-kinase) and to compare the phosphorylation of GLUT4 with GLUT2 in relation to the mechanism of translocation.	Adipocytes and hepatocytes isolated from male Sprague–Dawley rats.	• Cells treated with insulin.<br>• In vitro phosphorylation reactions performed using recombinant human PKC-ζ enzyme, lipid activators (phosphatidylserine, PS; phosphatidylinositol-3,4,5-trisphosphate, PIP3), and radiolabeled ATP (γ-32P-ATP).	• Phosphoimage scanning to quantify 32P incorporation.<br>• Western blotting / immunoblotting using anti-GLUT4 and anti-GLUT2 antibodies.<br>• Subcellular fractionation into the following compartments:<br>– Plasma membrane (PM)<br>– Lysosome (LYSO)<br>– High-density microsomes (HDM)<br>– Low-density microsomes (LDM)	• Insulin treatment: 20 minutes.<br>• In vitro phosphorylation reaction: 30 minutes.	• Basal / control group without insulin treatment.<br>• Comparative analysis between GLUT4 (insulin-responsive) and GLUT2 (insulin-unresponsive).	GLUT4 translocation may be attributed to phosphorylation of GLUT4 by PKC-ζ, whereas GLUT2 does not translocate because it is not phosphorylated by this kinase. Therefore, phosphorylation of GLUT proteins may be required for the GLUT translocation mechanism.	• PKC-ζ (Protein Kinase C-zeta)	Stimulation	Direct phosphorylation of GLUT4 by PKC-ζ (downstream of PI3-kinase) is necessary and may be responsible for insulin-stimulated GLUT4 translocation. In contrast, the absence of this phosphorylation event in GLUT2 explains why GLUT2 does not undergo translocation.	Signaling										Unclear risk (Not reported)	Unclear risk (Not reported)	Low risk	Unclear risk (Not reported)	Unclear risk (Not reported)	Low risk	

A.151	10.1073/pnas.1311232110	Haijia Yu et al., 2013	United States (USA) and Australia (Australia)	The research objective was to determine the molecular mechanism of Munc18c by reconstituting it in a defined membrane fusion reaction system containing GLUT4 exocytic SNAREs, and to compare it with Munc18-1 in order to identify conserved and divergent mechanisms.	• Recombinant liposome model (Proteoliposomes) containing purified SNARE proteins. • 3T3-L1 adipocytes (used for physiological validation).	• Addition of recombinant Munc18c protein into the in vitro fusion system. • Use of mutant forms of VAMP2 and Munc18-1. • Overexpression of Munc18c in 3T3-L1 adipocytes. • Insulin stimulation (in cell-based experiments).	• FRET-based lipid-mixing assay (fluorescence-based measurement of lipid mixing). • Content-mixing assay (measurement of content mixing inside liposomes). • Isothermal titration calorimetry (ITC) (heat measurement to determine binding affinity). • Liposome co-flotation/pull-down assay (liposome flotation/pull-down to assess protein binding). • Glucose uptake assay (measurement of glucose uptake in cells).	• Fusion reactions were continuously monitored for approximately 100 minutes (or 50–70 minutes depending on the figure), with initial rates calculated during the first 10 minutes. • Binding assay (ITC): measured for approximately 70 minute	• Group without Munc18c (Buffer/Basal). • Group using non-cognate SNAREs (Non-cognate SNAREs: Synaptic, Lysosomal, Yeast). • Group inhibited by the VAMP2 CD (cytoplasmic domain) fragment. • Control cell group without Munc18c overexpression.	• Munc18c binds to the trans-SNARE (soluble N-ethylmaleimide-sensitive factor attachment protein receptor) complex and strongly increases the membrane fusion rate; it recognizes both vesicle-associated SNAREs and target membrane-associated SNAREs. • The stimulation of fusion by Munc18 is specific to its cognate SNARE pairs. • Munc18c also binds to monomeric syntaxin-4 but does not inhibit SNARE complex formation at the target membrane. • Munc18c recognizes the N-terminal region of the vesicle-derived SNARE, whereas Munc18-1 requires the C-terminal region to recognize its SNAREs	Munc18c, Munc18-1	Stimulation	• Munc18c promotes the zippering of the trans-SNARE complex at the post-docking stage. • Munc18c recognizes the N-terminal region of the v-SNARE (VAMP2), whereas Munc18-1 requires the C-terminal region. This indicates that the architecture of the SNARE/SM complex differs among trafficking pathways.	Fusion										Unclear risk (Not reported)	Unclear risk (Not reported)	Low risk	Unclear risk (Not reported)	Unclear risk (Not reported)	Low risk	
A.153	10.1091/mbc.e06-01-0030	Mayumi Inoue et al., 2006	United States (USA)	The study aimed to evaluate whether the exocyst complex (including Exo70, Sec6, and Sec8) plays a critical role in insulin-stimulated Glut4 vesicle trafficking and glucose transport, with particular emphasis on the role of lipid rafts in this process.	• Adipocytes differentiated from the 3T3-L1 fibroblast cell line.	• Insulin stimulation. • Gene expression knockdown (KD) using RNAi (for Exo70, Sec6, Sec8, SAP97). • Expression of mutant forms (e.g., dominant-negative Exo70-N, TC10 mutants).	• Glucose uptake measurement: ^{14}C-2-deoxyglucose uptake assay. • Protein analysis: Immunoblotting (Western blot). • Immunoprecipitation. • Cell imaging: Immunofluorescence microscopy on plasma membrane sheets. • Subcellular fractionation: Sucrose gradient centrifugation to isolate lipid raft fractions.	• Glucose uptake: measured for 5 minutes after 30 minutes of insulin incubation. • Protein/Glut4 translocation: measured at 0, 2, 5, 10, 20, and 30 minutes after insulin stimulation.	• Basal group (no insulin). • Scrambled RNAi oligonucleotide control group. • Mock (mock transfection) group.	• The exocyst complex (including Exo70, Sec6, and Sec8) regulates the compartmentalization of GLUT4-containing vesicles at lipid raft domains in adipocytes.	Exocyst complex (Exo70, Sec6, Sec8)	Stimulation	• SAP97 was identified as an anchoring protein that retains Sec8 of the exocyst complex at lipid rafts. • Assembly of the exocyst complex (with Exo70 recruited by TC10 and Sec6/Sec8 anchored by SAP97) at lipid rafts creates tethering sites for Glut4-containing vesicles prior to membrane fusion.	Tethering										Unclear risk (Not reported)	Unclear risk (Not reported)	Low risk	Unclear risk (Not reported)	Unclear risk (Not reported)	Low risk	
A.154	10.1042/BJ20071114	Shuai Chen, 2008	United Kingdom (Scotland, U.K.)	The study investigated the regulation of phosphorylation and 14-3-3 protein binding of TBC1D1, in comparison with AS160, to elucidate the mechanisms regulating GLUT4 vesicle trafficking under the influence of growth factors, insulin, and AMPK activators.	• Human embryonic kidney cells (HEK-293). • Muscle cells (L6 myotubes) differentiated from rat myoblasts.	• Stimulation with: Insulin, IGF-1, EGF, PMA, Serum, Forskolin, A23187. • AMPK activation by: AICAR, Phenformin, A-769662. • Kinase inhibition by: LY294002, Wortmannin (PI3K), Compound C (AMPK), BI-D1870, Go6983.	• Immunoprecipitation. • 14-3-3 overlay assays (to determine 14-3-3 binding capacity). • Western blot (using site-specific antibodies against phosphorylated pSer237 and pThr596). • Mass spectrometry to identify phosphorylation sites. • Immunocytochemistry to examine the co-localization of TBC1D1 and GLUT4.	• Treatment times varied by stimulus: IGF-1/EGF/Serum (15 minutes); Insulin/PMA (30 minutes); AICAR/Phenformin/A-769662 (1 hour).	• Unstimulated / serum-starved group. • Comparison between wild-type (WT) proteins and mutant forms (Mutant: T596A, S237A...).	• TBC1D1 and AS160 are distinct but overlapping regulatory proteins that control GLUT4 vesicle trafficking in response to insulin and AMPK-activating stimuli in skeletal muscle.	TBC1D1 is phosphorylated	Stimulation	• The study identified complementary regulation between TBC1D1 and AS160: in skeletal muscle cells, AS160 primarily mediates insulin signaling (via phosphorylation at Thr642/Ser341 and 14-3-3 binding), whereas TBC1D1 primarily mediates AMPK signaling (energy/exercise-related) via phosphorylation at Ser237 and 14-3-3 binding. • Specific phosphorylation sites controlling 14-3-3 binding on TBC1D1 were identified as Ser237 (an AMPK target) and Thr596 (an Akt/PKB target). • An in vitro mutual inhibitory mechanism was identified: phosphorylation at Ser235 prevents subsequent phosphorylation at Ser237.	Signaling										Unclear risk (Not reported)	Unclear risk (Not reported)	Low risk	Unclear risk (Not reported)	Unclear risk (Not reported)	Low risk	
A.155	10.1042/BCJ20200542	Martin Alejandro Pavarotti et al., 2021	Argentina, Canada	The objective of the study was to investigate the expression and function of complexins in L6 skeletal muscle cells, to demonstrate their presence, Rac1-dependent redistribution to the plasma membrane (PM), and the involvement of complexin-2 in insulin-stimulated GLUT4 translocation.	• L6 rat skeletal muscle cells, including both myoblasts and myotube	• Insulin stimulation. • Genetic interventions: Complexin-2 knockdown by siRNA; overexpression of GFP-Complexin-2. • Pharmacological inhibitors: MK2206 (Akt inhibitor), NSC23766 (Rac1 inhibitor).	• GLUT4 translocation measurement: Colorimetric assay (ELISA-like surface assay) and immunofluorescence. • Glucose uptake measurement: Glucose uptake assay. • Protein and signaling analysis: Immunoblotting (Western blot), Immunoprecipitation. • Gene expression analysis: qPCR. • Actin remodeling observation: Actin polymerization staining (Phalloidin).	• GLUT4 translocation and signaling: measured after 20 minutes of insulin stimulation. • Complexin-2 redistribution by immunofluorescence: measured after 10 minutes of insulin stimulation.	• Basal group versus insulin-stimulated group. • Control siRNA / non-related siRNA group versus Complexin-2 knockdown group. • GFP control group versus GFP-Complexin-2 overexpression group.	• Complexin-2 is expressed and homogeneously distributed in L6 rat skeletal muscle cells. Upon insulin stimulation, a pool of complexin-2 redistributes toward the plasma membrane	Complexin-2	Inhibition (when concentration is altered)	• The study identified complexin-2 as a regulatory factor at the fusion node of GLUT4 vesicles. Specifically: • Insulin induces complexin-2 translocation to the plasma membrane via Rac1 signaling and actin remodeling, independently of the Akt pathway. • Complexin-2 is required to stabilize the trans-SNARE complex (between VAMP2, Syntaxin4, SNAP23). Loss of complexin-2 destabilizes this complex and prevents membrane fusion. Conversely, excess complexin-2 causes excessive stabilization ("fusion clamp"), locking the SNARE complex and also preventing membrane fusion. This demonstrates that complexin-2 concentration must be precisely fine-tuned to permit GLUT4 translocation.	Membrane Fusion										Unclear risk (Not reported)	Unclear risk (Not reported)	Low risk	Unclear risk (Not reported)	Unclear risk (Not reported)	Low risk	
A.164	10.1074/jbc.RA118.004614	Hatakeyama et al, 2018	Japan	The objective of the study was to analyze the cooperative functional activities between two RabGAP proteins, AS160 and Tbc1d1, in regulating the liberation of GLUT4 from intracellular storage compartments in response to insulin or exercise-mimicking stimuli.	• Cell-based reconstitution model: 3T3-L1 fibroblasts ectopically expressing Myc-GLUT4, HA-sortilin, and AS160/Tbc1d1. • Differentiated 3T3-L1 adipocytes.	• Insulin stimulation. • Exercise-mimicking stimulation: AICAR (AMPK activation). • Elevation of intracellular Ca2+ concentration (using the photolabile compound NPE). • Co-expression or individual expression of AS160 and Tbc1d1. • Use of mutant forms of AS160 and Tbc1d1.	• GLUT4 nanometry measurement: single-molecule tracking using quantum dots (QD) to measure GLUT4 mobility. • Multiple flow cytometry-based assays to analyze phosphorylation states.	• GLUT4 mobility was measured at 0, 5, 15, and 30 minutes after stimulation (insulin/AICAR). • Measurements were taken immediately (0.5 minutes) and 10 minutes after Ca2+ elevation.	• Comparison of cells expressing AS160 alone vs. Tbc1d1 alone vs. co-expression of both (AS160 + Tbc1d1). • Comparison between basal conditions vs. Insulin vs. AICAR vs. Insulin + Ca2+. • Comparison between wild-type (WT) proteins and mutant forms (T642A, S237A, T596A, R125W...).	• Tbc1d1 and AS160 cooperate as central regulatory nodes that convert insulin and/or exercise signals into the process of GLUT4 liberation.	AS160 (Tbc1d4), Tbc1d1	Stimulation	• Regulatory mode shift: Tbc1d1 requires priming signals such as AICAR or Ca2+ to switch into an "insulin-responsive" state. • Role of AS160: AS160 does not act independently only, but also facilitates the regulatory mode shift of Tbc1d1, enabling rapid GLUT4 liberation (as early as 0.5 minutes after Ca2+ elevation) rather than delayed release. • Molecular requirements: This cooperation depends on the phosphotyrosine-binding domain (PTB1) and the calmodulin-binding domain (CBD) of Tbc1d1, together with key phosphorylation sites on AS160 (T642) and Tbc1d1 (S237, T596)	Signaling										Unclear risk (Not reported)	Unclear risk (Not reported)	Low risk	Unclear risk (Not reported)	Unclear risk (Not reported)	Low risk	
A.166	10.1096/fj.09-137380	Ueda et al., 2010	Japan	The study examined the involvement of Rac1 in insulin action in mouse skeletal muscle.	• Mouse skeletal muscle (gastrocnemius) from wild-type mice and muscle-specific rac1 knockout mice (m-rac1-KO).	• Intravenous insulin injection. • Ectopic expression of constitutively activated Rac1 via in vivo electroporation.	• Immunofluorescent staining of GLUT4 with an exofacial epitope-tagged reporter (GLUT4myc7-GFP). • Immunogold electron microscopy.	• Measurements performed from 0 to 20 minutes after insulin injection.	• Wild-type (control) mice versus m-rac1-KO mice.	• Insulin activates Rac1 in mouse skeletal muscle. • Rac1 is required for the step of GLUT4 translocation to the plasma membrane.	Rac1 (Rho family GTPase)	Stimulation	• Rac1 plays a crucial role in insulin-dependent GLUT4 translocation to the skeletal muscle sarcolemma in vivo, regulating cortical actin remodeling	Translocation	Unclear risk (Not reported)	Low risk	Unclear risk (Not reported)	Unclear risk (Not reported)	Unclear risk (Not reported)	Unclear risk (Not reported)	Unclear risk (Not reported)	Low risk	Low risk	Low risk	Unclear risk (Not reported)	Low risk	Unclear risk (Not reported)	Unclear risk (Not reported)	Low risk	
A.176	10.1152/ajpendo.00316.2012	Melissa N. Lansey et al., 2012	United States (USA)	The study aimed to determine the function of AS160 in GLUT4 trafficking in skeletal muscle and primary adipocytes, and to investigate its role in glucose homeostasis.	• AS160 whole-body knockout mice (AS160(-/-)) and primary tissues/cells isolated from them: white adipocytes (from epididymal/periuterine fat), soleus muscle, extensor digitorum longus (EDL), gastrocnemius muscle, and quadriceps muscle.	• Genetic intervention: AS160 gene deletion (AS160 knockout). • Physiological intervention: Insulin stimulation (in vivo and in vitro)	• Glucose uptake measurement (using 2-deoxy-D-[1,2-^{3}H]glucose). • Western blot (immunoblotting) to measure protein expression (GLUT4, GLUT1, AS160, Akt, Tbc1d1). • Subcellular fractionation to isolate plasma membrane (PM).	• In vitro (isolated cells/muscles): after 15–20 minutes of insulin incubation. • In vivo (tolerance tests): measurements at 0, 15, 30, 45, 60, and 120 minutes after injection. • In vivo (tissue fractionation): 30 minutes after insulin injection.	• Wild-type littermates (AS160(+/+)) under basal conditions and insulin-stimulated conditions.	• Both insulin and exercise caused GLUT4 translocation in skeletal muscle.	AS160	Inhibition	• AS160 plays an essential role in retaining GLUT4 intracellularly under basal conditions (particularly in adipocytes). • Loss of AS160 leads to uncontrolled GLUT4 movement to the plasma membrane even in the absence of insulin, but total GLUT4 levels are downregulated, resulting in impaired insulin responsiveness. • The regulatory mechanisms of GLUT4 trafficking differ between skeletal muscle and adipose tissue in the absence of AS160.	Signaling	Unclear risk (Not reported)	Low risk	Unclear risk (Not reported)	Unclear risk (Not reported)	Unclear risk (Not reported)	Unclear risk (Not reported)	Unclear risk (Not reported)	Low risk	Low risk	Low risk	Unclear risk (Not reported)	Low risk	Unclear risk (Not reported)	Unclear risk (Not reported)	Low risk	
A.186	10.1210/endo.137.1.8536622	L. Adam Sherman et al., 1996	United States (USA) and France (France)	The study aimed to determine whether Rab4 (a small GTP-binding protein) is involved in the regulation of GLUT4 translocation in skeletal muscle in response to insulin and exercise.	• Male Sprague–Dawley rats, weighing 150–175 g. • Studied tissue: hind limb skeletal muscles.	• Insulin: intraperitoneal (ip) injection of 20 U porcine insulin. • Exercise: treadmill running for 1 hour at a speed of 20 m/min with a 10% incline.	• Subcellular fractionation to isolate plasma membrane and intracellular microsomal membrane fractions. • Immunoblotting (Western blot) to determine the distribution of GLUT4 and Rab4. • Immunoprecipitation to determine the colocalization of Rab4 and GLUT4 on vesicles	• Insulin: 30 minutes after injection. • Exercise: immediately after completion of the exercise bout.	• Basal group – rats not treated with insulin or exercise.	• Rab4 is abundantly present in microsomal membranes of rat skeletal muscle. • Although both insulin and exercise stimulate GLUT4 translocation from intracellular microsomal pools to the plasma membrane, only insulin is associated with a reduction of Rab4 bound to microsomal membranes.	Rab4	Stimulation	• Insulin regulates the subcellular distribution of both GLUT4 and Rab4, with Rab4 dissociating from GLUT4-containing vesicles at the microsomal membrane upon insulin stimulation, suggesting that Rab4 participates in insulin-stimulated GLUT4 vesicle trafficking. • In contrast, exercise stimulates GLUT4 translocation via a distinct mechanism or vesicle pool that is not associated with Rab4 redistribution.	Translocation	Unclear risk (Not reported)	Low risk	Unclear risk (Not reported)	Unclear risk (Not reported)	Unclear risk (Not reported)	Unclear risk (Not reported)	Unclear risk (Not reported)	Low risk	Low risk	Low risk	Unclear risk (Not reported)	Low risk	Unclear risk (Not reported)	Unclear risk (Not reported)	Low risk	
A.187	10.1074/jbc.M109093200	Romel Somwar et al., 2001	Canada	The study aimed to identify the bifurcation point of signaling pathways regulating GLUT4 translocation and activation, with particular emphasis on comparing the sensitivity of these two processes and their associated signaling pathways to PI 3-kinase inhibitors.	• Differentiated L6 muscle cells (L6 myotubes) stably expressing Myc-tagged GLUT4 (L6-GLUT4myc).	• Insulin stimulation (100 nM). • Inhibition with Wortmannin (various concentrations). • Inhibition with LY294002 (various concentrations). • Expression of dominant-negative PI 3-kinase mutant (Δp85).	• Measurement of 2-deoxyglucose uptake (glucose uptake). • Detection of cell-surface GLUT4myc using antibody-coupled colorimetric assay and immunofluorescence. • Western blot (immunoblotting) to detect phosphorylation of Akt and p38 MAPK. • In vitro kinase assays for PI 3-kinase, Akt, PKCζ/λ, and p38 MAPK.	• Inhibitor pretreatment: 20 minutes. • Insulin stimulation: 20 minutes (for glucose uptake / translocation) or 10 minutes (for kinase signaling).	• Basal versus insulin-stimulated conditions. • Insulin alone versus insulin plus inhibitors (Wortmannin / LY294002). • Non-transfected cells versus cells transfected with Δp85.	• Insulin-stimulated glucose uptake is more sensitive to PI3K inhibition than GLUT4 translocation. • With Wortmannin treatment, glucose uptake and GLUT4 translocation do not occur concurrently; this dissociation is not observed with LY294002. • p38 MAPK belongs to a distinct signaling branch: p38 MAPK is strongly inhibited by Wortmannin but is not affected by LY294002 or by dominant-negative PI3K (Δp85).	PI 3-Kinase, p38 MAPK	Stimulation	• Insulin activates p38 MAPK through a high-affinity Wortmannin target that is distinct from class IA PI 3-kinase. • This p38 MAPK pathway governs GLUT4 "activation" (increased intrinsic activity) required for maximal glucose uptake, and is mechanistically separate from GLUT4 translocation.	Signaling										Unclear risk (Not reported)	Unclear risk (Not reported)	Low risk	Unclear risk (Not reported)	Unclear risk (Not reported)	Low risk	
A.190	10.1074/jbc.M003883200	Lu Li et al., 2001	Japan	The study aimed to examine a potential direct interaction between Rab4 and Syntaxin 4, and to determine the regulatory mechanisms of this interaction (by insulin and guanine nucleotide status) during GLUT4 translocation.	• Primary adipocytes isolated from epididymal fat tissue of male rats (Rat adipocytes).	• Insulin treatment (100 nM). • Treatment with nucleotide analogs: GTPγS (non-hydrolyzable GTP), GDPβS. • Use of inhibitors: Wortmannin (PI 3-kinase inhibitor). • Use of recombinant mutant Rab4 proteins: Rab4Q67L (GTPase-deficient – constitutively GTP-bound), Rab4S22N (GTP-binding–defective – GDP-bound form). • Competitive intervention: addition of Munc-18c protein.	• Co-immunoprecipitation. • Immunoblotting / Western blotting. • In vitro binding assays / pull-down assays using GST-fusion proteins. • Quantification of [γ-^{32}P]GTP binding.	• Insulin / GTPγS / GDPβS treatments: typically 15–20 minutes. • Time-course analysis of insulin effects: 0, 2, 5, and 15 minutes.	• Basal condition without insulin. • Control groups without nucleotide treatment or treated with GDPβS (for comparison with GTPγS). • Wild-type (WT) Rab4 versus mutant variants (Q67L, S22N).	• (1) Rab4 interacts directly and specifically with Syntaxin 4; and (2) this interaction is regulated by the guanine nucleotide status of Rab4 as well as by the conformational status of Syntaxin 4.	Rab4 (small GTPase) and Syntaxin 4 (t-SNARE).	Stimulation	• Rab4 interacts directly and specifically with the cytoplasmic domain of Syntaxin 4. • This interaction depends on the GTP-bound state of Rab4 (the GTP-bound form has higher affinity than the GDP-bound form) and on the conformational state of Syntaxin 4 (Rab4 binds only when Syntaxin 4 is in the "open" state; binding of Munc-18c to Syntaxin 4 prevents Rab4 interaction).	Docking										Unclear risk (Not reported)	Unclear risk (Not reported)	Unclear risk (Not reported)	Unclear risk (Not reported)	Unclear risk (Not reported)	Unclear risk (Not reported)	
A.191	10.1074/jbc.M708688200	Li Jiang, 2008	China	To develop a novel dual-color fluorescent probe (TDimer2-IRAP-pHluorin) to monitor GLUT4 translocation in real time and to identify the specific role of AS160 in the docking and fusion steps of GLUT4 storage vesicles (GSVs).	• Differentiated 3T3-L1 adipocytes	• Insulin stimulation (100 nM). • Overexpression of a dominant negative mutant of AS160 (AS160-4P). • Use of the TDimer2-IRAP-pHluorin probe (RITS).	• Dual-color Total Internal Reflection Fluorescence Microscopy (TIRFM). • Ratiometric fluorescence measurement.	• Real-time measurement immediately after insulin stimulation. • Fusion events tracked every 20 seconds. • Observation extended to steady state (approximately 20–30 minutes after stimulation).	• Basal (no insulin) conditions versus insulin stimulation. • Control cell groups versus cells expressing the AS160-4P mutant.	• Insulin stimulation induces an approximately 40-fold increase in GSV fusion events in 3T3-L1 adipocytes compared with basal conditions. • Overexpression of the non-phosphorylatable AS160-4P mutant significantly reduces both the docking rate and the fusion rate of GLUT4-containing vesicles. • Expression of a dominant negative mutant of AS160 proportionally reduces both docking and fusion of GSVs, indicating that AS160 plays a role in the docking stage of GSVs but does not participate in the regulation of fusion after docking has occurred.	AS160	Inhibition	• AS160 regulates the docking step of GSVs to the plasma membrane. • AS160 does not regulate the fusion step after docking has occurred. • Once docking has occurred, AS160 no longer affects fusion. • A small fraction of GSVs undergo "kiss-and-run" fusion.	Docking										Unclear risk (Not reported)	Unclear risk (Not reported)	Low risk	Unclear risk (Not reported)	Unclear risk (Not reported)	Low risk	
A.192	10.1016/s0021-9258(18)43971-3	Theodoros Tsakiridis, 1994	Canada	To determine the role of the actin microfilament network in insulin-stimulated glucose transport and in the distribution of glucose transporters.	Differentiated mouse skeletal muscle cells (L6 myotubes).	•Use of Cytochalasin D (CD), an inhibitor of actin filament formation, combined with insulin stimulation.	• Measurement of glucose uptake (using 2-deoxy-[^{3}H]D-glucose). • Fluorescence microscopy (Rhodamine-labeled phalloidin staining to visualize actin). • Subcellular fractionation to isolate plasma membrane (PM) and intracellular membrane (IM) fractions. • Immunoblotting (Western blot) to quantify GLUT1, GLUT3, and GLUT4. • Immunoprecipitation of GLUT4-containing vesicles.	• Measurements performed after Cytochalasin D treatment for 2 hours and insulin stimulation during the final 30 minutes of incubation	• Untreated control group (no Cytochalasin D). • Basal group without insulin stimulation.	• An intact actin network is required for the correct intracellular localization of glucose transporters. • The actin network is necessary for the incorporation of glucose transporters into the plasma membrane in response to insulin. • A direct interaction may exist between the actin network and glucose transporter-containing vesicles, potentially mediated by a spectrin-containing skeleton attached to these vesicles.	Actin (microfilament network).	Stimulation	• The study identifies a direct association between GLUT4-containing vesicles and the cytoskeletal protein spectrin. • Spectrin may act as a linker connecting GLUT4 vesicles to the intracellular actin network, stabilizing the storage pool and supporting translocation to the plasma membrane.	Translocation										Unclear risk (Not reported)	Unclear risk (Not reported)	Low risk	Unclear risk (Not reported)	Unclear risk (Not reported)	Low risk	
A.193	10.1074/jbc.M708839200	Eric B. Taylor, 2008	United States (USA)	• To identify the protein responsible for the phospho-Akt-substrate (PAS) signal at approximately 160 kDa in different skeletal muscle types, specifically examining differences between soleus and TA/EDL muscles. • To determine the role of TBC1D1 as a novel signaling node stimulated by insulin, AICAR, and muscle contraction.	• Male ICR mice, 8–10 weeks of age. • Skeletal muscles examined included soleus, tibialis anterior (TA), and extensor digitorum longus (EDL).	• In vivo: • Intraperitoneal injection of insulin (1 unit). • Subcutaneous injection of AICAR (1 mg/g) to activate AMPK. • In situ electrical stimulation to induce muscle contraction via the peroneal nerve. • In vitro: • Incubation of immunoprecipitated TBC1D1 protein with recombinant AMPK or Akt enzymes.	• Immunoblotting (Western blot) using phospho-Akt-substrate (PAS) antibody and TBC1D1-specific antibody. • Immunoprecipitation to separate AS160 and TBC1D1. • Mass spectrometry (LC–MS/MS) for protein identification and phosphorylation site mapping. • Real-time PCR for measurement of mRNA expression levels.	• Insulin: 5, 10, and 20 minutes after injection. • AICAR: 10, 30, and 60 minutes after injection. • Muscle contraction: immediately after 15 minutes of electrical stimulation (tissues rapidly frozen).	• Basal/control group: mice injected with saline or sham-operated limbs without electrical stimulation.	• A PAS-immunoreactive protein distinct from AS160 exists in skeletal muscle and was identified as TBC1D1. • In vitro, TBC1D1 is phosphorylated by insulin, muscle contraction, and AICAR, with the majority of phosphorylation sites conforming to AMPK consensus motifs. • TBC1D1 is the major PAS-reactive protein in skeletal muscle and is phosphorylated in vivo by insulin, AICAR, and muscle contraction. • Both Akt and AMPK are capable of phosphorylating TBC1D1, but AMPK acts as the more robust regulator.	TBC1D1 (phosphorylated).	Stimulation	• TBC1D1 is the principal PAS-160 protein in fast-twitch skeletal muscles (TA, EDL), whereas AS160 predominates in slow-twitch muscle (soleus). • TBC1D1 is directly phosphorylated by both Akt (insulin-mediated) and AMPK (AICAR- and contraction-mediated). • AMPK regulates TBC1D1 phosphorylation more robustly than Akt at multiple specific sites (Ser-231, Thr-499, Ser-660, Ser-700), inducing an electrophoretic mobility shift.	Signaling	Unclear risk (Not reported)	Low risk	Unclear risk (Not reported)	Unclear risk (Not reported)	Unclear risk (Not reported)	Unclear risk (Not reported)	Unclear risk (Not reported)	Low risk	Low risk	Low risk	Unclear risk (Not reported)	Low risk	Unclear risk (Not reported)	Unclear risk (Not reported)	Low risk	
A.194	10.1093/emboj/19.14.3565	Thurmond DC and Pessin JE, 2000	United States (USA)	• To determine the temporal relationship between pre- and post-docking events. • To distinguish vesicle trafficking from membrane fusion in order to identify the rate-limiting step in insulin-stimulated GLUT4 translocation.	• Differentiated 3T3-L1 adipocytes.	• Expression of temperature-sensitive mutant Munc18c (Munc18c/TS – R240K) and wild-type Munc18c (Munc18c/WT) via electroporation. • Insulin stimulation (100 nM). • Manipulation of culture temperature (permissive temperature 23 °C and non-permissive temperature 37 °C) with temperature-shifting protocols.	• Confocal fluorescence microscopy on plasma membrane sheets to visualize GLUT4 and IRAP translocation (using antibodies or EGFP tagging). • Co-immunoprecipitation and immunoblotting to assess protein–protein interactions and expression levels.	• Measurements at 0, 5, 15, 30, 60, and 90 minutes after insulin stimulation. • Biochemical analyses performed after 4 hours of incubation at the indicated temperatures.	• Cells transfected with empty vector (pcDNA3). • Cells expressing wild-type Munc18c (Munc18c/WT). • Comparison between 23 °C and 37 °C temperature conditions.	• GLUT4 translocation reflects vesicle trafficking rather than vesicle fusion with the plasma membrane. • Insulin stimulates the trafficking of GLUT4 vesicles prior to membrane fusion. • Munc18c regulates GLUT4 translocation in a conformation-dependent manner.	Munc18c	Stimulation	• The interaction between Munc18c and syntaxin 4 is temperature-sensitive in the R240K mutant. • Release of docking inhibition by temperature shifting after insulin signaling activation accelerates GLUT4 translocation. • These findings demonstrate that vesicle trafficking to the plasma membrane, rather than docking or fusion, constitutes the rate-limiting step in insulin-stimulated GLUT4	Trafficking (vesicle transport) and Docking										Unclear risk (Not reported)	Unclear risk (Not reported)	Low risk	Unclear risk (Not reported)	Unclear risk (Not reported)	Low risk	
A.195	10.1016/j.jbc.2021.100482	Fujimoto et al., 2021	United States (USA)	To investigate the in vivo role of the exocyst subunit EXOC5 in skeletal muscle with respect to glucose homeostasis and insulin-stimulated glucose uptake, using a skeletal muscle–specific Exoc5 knockout mouse model.	• Skeletal muscle–specific Exoc5 knockout mice (Exoc5-SMKO) and littermate control mice. • Mice were generated by crossing Exoc5 floxed mice with HSA-MCM (Human Skeletal Actin Mer-Cre-Mer) mice.	• Genetic intervention: Tamoxifen-inducible knockout of Exoc5. • Physiological stimulation: Insulin (during hyperinsulinemic–euglycemic clamp studies and insulin injections for signaling analyses). • Dietary intervention: High-fat diet (HFD) for 16 weeks (in a subset of experiments).	• Glucose uptake: Hyperinsulinemic–euglycemic clamp using 2-[^{14}C] deoxyglucose and ^{3}H-glucose. • Whole-body metabolism: Indirect calorimetry to measure respiratory exchange ratio (RER) and energy expenditure. • GLUT4 localization: Immunofluorescence staining of skeletal muscle. • Additional assays: Glucose tolerance test (GTT), insulin tolerance test (ITT), and Western blot analysis.	• GTT/ITT: Measurements over 120 minutes (time points at 0, 30, 60, 90, and 120 minutes). • Glucose uptake (clamp): Performed in 20-week-old mice. • Indirect calorimetry: Continuous measurements over a 24-hour cycle.	• Littermate control mice, specifically Exoc5^fl/fl;+/+ genotypes without active Cre recombinase.	• EXOC5 and the exocyst complex are required for insulin-stimulated glucose uptake in skeletal muscle. • EXOC5 contributes to the regulation of whole-body glucose homeostasis in vivo.	EXOC5 (a subunit of the exocyst protein trafficking complex).	Stimulation	• EXOC5 is essential for insulin-stimulated GLUT4 exocytosis in skeletal muscle in vivo. • Loss of EXOC5 reduces skeletal muscle glucose utilization, promotes a metabolic shift toward lipid utilization, and leads to hepatic lipid accumulation despite the absence of intrinsic hepatic defects. • These alterations collectively disrupt systemic glucose homeostasis.	Docking	Unclear risk (Not reported)	Low risk	Unclear risk (Not reported)	Unclear risk (Not reported)	Unclear risk (Not reported)	Unclear risk (Not reported)	Unclear risk (Not reported)	Low risk	Low risk							
A.196	10.2337/db15-1128	Vazirani et al., 2016	United States (USA)	• To determine the role of Rab10 in regulating insulin-stimulated Glut4 trafficking and glucose uptake in primary adipocytes in vivo. • To evaluate the impact of adipose-specific Rab10 deficiency on whole-body glucose homeostasis	• Primary adipocytes isolated from white adipose tissue (WAT). • Female mice with adipose-specific Rab10 gene deletion (Rab10KO).	• Genetic intervention: Adipose-specific Rab10 knockout using the Cre–lox system. • Stimulation: Insulin (ex vivo insulin treatment of adipocytes or in vivo insulin infusion during clamp studies).	• Measurement of glucose uptake using radiolabeled ^{3}H-2-deoxyglucose. • Assessment of Glut4 translocation using HA-Glut4-GFP reporter and immunofluorescence microscopy. • Euglycemic–hyperinsulinemic clamp to measure glucose infusion rate and hepatic glucose production. • RNA sequencing (RNA-seq) of adipose tissue.	• Glucose uptake: Insulin stimulation for 20 minutes followed by glucose uptake measurement for 20 minutes. • Glut4 translocation: Insulin stimulation for 30 minutes. • Clamp studies: Measurements at steady state during continuous insulin infusion (2.5 mU/kg/min).	• Littermate control mice, including wild-type mice, Cre-only mice, or floxed-only mice without Rab10 deletion.	• Insulin-stimulated glucose uptake and Glut4 translocation to the plasma membrane are reduced by approximately 50% in adipocytes from adipose-specific Rab10 knockout mice. • The full effect of insulin on adipose glucose uptake reflects the integrated contribution of Rab10-dependent and Rab10-independent pathways. • These findings establish a divergence in insulin signal transduction regulating Glut4 trafficking.	Rab10 (Rab10 GTPase)	Stimulation	• Rab10 accounts for approximately 50% of insulin signaling regulating Glut4 translocation in primary adipocytes in vivo, indicating branching between Rab10-dependent and Rab10-independent pathways. • Partial impairment of adipose Glut4 translocation leads to systemic insulin resistance, primarily driven by hepatic insulin resistance due to impaired suppression of hepatic glucose production. • Insulin-mediated suppression of adipose lipolysis (free fatty acid release) remains intact despite Rab10 deficiency.	Translocation	Unclear risk (Not reported)	Low risk	Unclear risk (Not reported)	Unclear risk (Not reported)	Unclear risk (Not reported)	Unclear risk (Not reported)	Unclear risk (Not reported)	Low risk	Low risk	Unclear risk (Not reported)	Unclear risk (Not reported)	Low risk	Unclear risk (Not reported)	Unclear risk (Not reported)	Low risk	
A.197	10.1074/jbc.M510920200	Jie Huang, 2005	United States (USA)	• To investigate the effects of microtubule disruption by pharmacological agents (nocodazole, colchicine) on GLUT4 translocation. • To examine whether PDGF can stimulate GLUT4 translocation under conditions of disrupted microtubules	Differentiated 3T3-L1 adipocytes.	• Cytoskeletal disruption reagents: Nocodazole (microtubule depolymerization), Colchicine (microtubule depolymerization), Cytochalasin D (actin depolymerization), Jasplakinolide (actin stabilization). • Stimulation: Insulin, PDGF. • Signal inhibition: Wortmannin, LY294002 (PI3K inhibitors), PD98059 (MEK inhibitor). • Genetic intervention: Microinjection of siRNA targeting Myo1c (actin-based motor) and KIF3/KAP3A (microtubule-based motors).	• Immunofluorescence staining and confocal microscopy to visualize GLUT4 and F-actin localization. • Plasma membrane sheet assay to quantify GLUT4 at the plasma membrane. • Measurement of glucose uptake using 2-deoxyglucose. • Western blot analysis to assess Akt/PKB phosphorylation	• Pretreatment: Nocodazole (2 hours), Colchicine (3 hours). • Stimulation: Insulin or PDGF for 20 minutes (GLUT4 translocation) or 30 minutes (glucose uptake).	• Vehicle control group (DMSO). • Cells with intact microtubules versus cells with disrupted microtubules (nocodazole- or colchicine-treated). • Comparison between insulin stimulation and PDGF stimulation.	• Proper perinuclear localization of GLUT4 vesicles is required for insulin-specific stimulation of GLUT4 translocation. • Nocodazole treatment disperses GLUT4 vesicles from the perinuclear region, enabling their interaction with insulin- and PDGF-sensitive actin filaments. • These actin filaments participate in GLUT4 translocation in a phosphatidylinositol 3-kinase (PI3K)-dependent manner.	Microtubules.	Stimulation	• Upon microtubule disruption, GLUT4 vesicles disperse from the perinuclear region and become competent to engage an actin-based transport system under PDGF stimulation via the actin motor protein Myo1c. • These findings indicate that microtubule-dependent perinuclear localization determines insulin specificity, whereas loss of this spatial constraint permits PDGF-driven GLUT4 mobilization through an actin-dependent pathway.	Translocation										Unclear risk (Not reported)	Low risk	Low risk	Unclear risk (Not reported)	Low risk	Low risk	
A.203	10.7554/eLife.26896	Beg et al., 2017	United States (USA)	To define the specific roles of Akt2 phosphorylation sites (T309 and S474) in regulating insulin-stimulated glucose uptake mediated by Glut4 and Glut1 in adipocytes and proliferating cells.	• 3T3-L1 adipocytes (differentiated from mouse fibroblasts). • Mouse lung cancer cell line (KP1 lung cancer cells).	• Physiological stimulation: Insulin (1 nM–170 nM). • Genetic interventions: • Overexpression of Akt2 variants (Akt2-W80A, T309A, S474A, S474D, E17K, etc.). • Expression of HA-Glut4-GFP and HA-Glut1 reporters. • Knockdown of Rictor (mTORC2 component) using siRNA. • Pharmacological interventions: • Akt inhibitor (MK2206). • PDPK1 inhibitor (GSK2334470). • Indinavir.	• Glut4/Glut1 translocation: Fluorescence microscopy using HA-Glut4-GFP or HA-Glut1 reporters (surface-to-total signal ratio). • Akt signaling activity: Western blot analysis of Akt phosphorylation and downstream substrates (AS160, FOXO1, S6K). • Glucose uptake: 2-deoxy-D-glucose (2-DOG) uptake assay.	• Measurements performed 30 minutes after insulin stimulation (for translocation and phosphorylation analyses).	• Control adipocytes without genetic manipulation or transfected with empty vector. • Cells treated with MK2206 to inhibit endogenous Akt compared with cells expressing Akt2 mutants (W80A, T309A, S474A). • Basal (no insulin) conditions versus insulin-stimulated conditions.	• Insulin-stimulated Glut4-mediated glucose uptake requires PDPK1-dependent phosphorylation of the Akt kinase domain but does not require mTORC2-dependent phosphorylation of the hydrophobic motif (HM). • An intact HM domain of Akt is structurally required for Glut4-mediated glucose uptake despite the lack of requirement for S474 phosphorylation. • In contrast, Glut1-mediated glucose uptake requires phosphorylation at both T309 and S474, indicating a dependence on mTORC2 activity. • These findings demonstrate distinct and independent roles of Akt phosphorylation sites in regulating glucose uptake via Glut4 and Glut1.	Akt2	Stimulation	• The study reveals a functional dissociation between Akt2 phosphorylation sites. • Insulin-stimulated Glut4 translocation requires phosphorylation at T309 by PDPK1 but does not require phosphorylation at S474 by mTORC2, although the hydrophobic motif containing S474 remains structurally essential. • In contrast, Glut1 translocation requires phosphorylation at both T309 and S474, indicating an essential role for mTORC2-dependent HM phosphorylation. • mTORC2 therefore links Akt signaling to distinct physiological programs governing Glut4- versus Glut1-mediated glucose uptake.	Signaling										Unclear risk (Not reported)	Unclear risk (Not reported)	Low risk	Unclear risk (Not reported)	Unclear risk (Not reported)	Low risk	
A.206	10.2337/db06-0150	Henning F. Kramer, 2006	United States (USA)	To define the signaling network regulating AS160 phosphorylation in skeletal muscle in response to insulin, muscle contraction, and AICAR.	• Mouse skeletal muscle, including extensor digitorum longus (EDL), gastrocnemius, and tibialis anterior muscles. • Mouse models used: female ICR mice, Akt2 knockout (KO) mice, and AMPKα2-inactive (α2i) transgenic mice.	• Insulin: Intraperitoneal injection (in vivo) or muscle incubation (in vitro). • Muscle contraction: Treadmill exercise, in situ electrical stimulation, or in vitro electrical stimulation. • AICAR: Pharmacological activator of AMPK. • Wortmannin: PI3-kinase inhibitor. • Genetic manipulations: Use of Akt2 KO mice and AMPKα2-inactive mice.	• Immunoblotting (Western blot) using phospho-Akt substrate (PAS) antibodies and a phospho-AS160 (Thr642)-specific antibody	• Muscle samples were collected immediately and frozen in liquid nitrogen after the following interventions: • Insulin in vivo: 10 minutes after injection. • Insulin in vitro: 20 minutes. • Treadmill exercise: 30 minutes. • AICAR: 40 minutes. • Muscle contraction (in situ/in vitro): 5 or 10 minutes.	• Control groups included basal/resting muscle, saline-injected mice, sedentary (non-exercised) mice, sham-operated animals, wild-type mice (relative to genetically modified mice), and groups not treated with wortmannin.	• AS160 serves as a potential point of convergence linking insulin, contraction, and AICAR signaling pathways. • Akt activity is essential for insulin-stimulated AS160 phosphorylation. • AMPKα2 activity is essential for AICAR-stimulated AS160 phosphorylation. • Neither Akt nor AMPKα2 alone is indispensable for the full effects of muscle contraction on AS160 phosphorylation.	AS160 (Akt substrate of 160 kDa).	Inhibition	• Distinct signaling pathways converge on AS160 to regulate its phosphorylation. • Insulin-induced AS160 phosphorylation is fully dependent on Akt2, as evidenced by complete inhibition with wortmannin and in Akt2 knockout mice. • AICAR-induced AS160 phosphorylation is fully dependent on AMPKα2 and occurs primarily at PAS motifs (serine residues), but not at Thr642. • Contraction-induced AS160 phosphorylation is regulated by coordinated input from AMPKα2, Akt, and potentially additional unidentified kinases; deletion of either Akt2 or AMPKα2 alone results in only partial attenuation of contraction-induced AS160 phosphorylation.	Signaling										Unclear risk (Not reported)	Unclear risk (Not reported)	Unclear risk (Not reported)	Unclear risk (Not reported)	Unclear risk (Not reported)	Unclear risk (Not reported)	

A.215	10.1016/j.yexcr.2008.08.007	Fulcher et al., 2008	United States (USA)	• To define the specific roles of myosin II isoforms, particularly myosin IIA, in insulin-stimulated GLUT4-mediated glucose uptake. • To distinguish the upstream signaling mechanisms regulating myosin IIA activation, specifically MLCK versus Rho kinase (RhoK).	Differentiated 3T3-L1 adipocytes.	• Stimulation: Insulin (100 nM). • Pharmacological inhibition: • Blebbistatin (myosin II inhibitor). • ML-7 and ML-9 (MLCK inhibitors). • Y-27632 (Rho kinase inhibitor).	• Radiolabeled glucose uptake assay (¹⁴C-2-deoxy-D-glucose uptake). • Immunoblotting (Western blot). • Immunoprecipitation. • Immunofluorescence and confocal fluorescence microscopy.	• Standard glucose uptake: Insulin stimulation for 10 minutes followed by 10 minutes of uptake measurement. • Short-term glucose uptake (to assess intrinsic transporter activity): Insulin stimulation for 2 minutes. • Translocation time course: 2, 4, 6, and 10 minutes after insulin stimulation.	• Basal (untreated) cells. • Vehicle control (DMSO)–treated cells stimulated with insulin but without inhibi	• Insulin-stimulated glucose uptake via GLUT4 is dependent on myosin II activity. • Myosin IIA is the critical isoform mediating insulin-stimulated glucose uptake. • MLCK, but not Rho kinase, is required for insulin-stimulated glucose uptake. • Insulin stimulates phosphorylation of the regulatory light chain (RLC) of myosin IIA via MLCK. • Myosin IIA is required for proper GLUT4 vesicle fusion at the plasma membrane. • Myosin IIA also regulates the intrinsic activity of GLUT4 after insulin stimulation.	• Myosin IIA (myosin II isoform A). • Myosin Light Chain Kinase (MLCK).	Stimulation	• Insulin selectively induces phosphorylation of the regulatory light chain (RLC) of myosin IIA via MLCK, not via Rho kinase. • Activated myosin IIA is recruited to the plasma membrane after GLUT4 translocation has occurred. • At the plasma membrane, myosin IIA is required for GLUT4 vesicle fusion and for activation of the intrinsic glucose transport activity of GLUT4. • Myosin IIA therefore plays a dual and essential role in mediating insulin-stimulated glucose uptake in 3T3-L1 adipocytes.	Fusion										Unclear risk (Not reported)	Unclear risk (Not reported)	Low risk	Unclear risk (Not reported)	Unclear risk (Not reported)	Low risk	
A.220	10.1371/journal.pone.0208427	Shastri AA, 2018	United States (USA)	To test the hypothesis that the viral protein E4orf1 requires distal insulin signaling but not proximal insulin signaling to enhance cellular glucose uptake.	3T3-L1 preadipocytes engineered for inducible expression of E4orf1 or empty vector control (Null Vector, NV).	• Gene expression: Induction of E4orf1 expression using doxycycline. • Pharmacological inhibition of insulin signaling: • S961 (insulin receptor, IR inhibitor). • PPP (IGF-1 receptor inhibitor). • Combined S961 and PPP to block proximal insulin signaling. • Wortmannin (PI3K inhibitor) to block distal insulin signaling. • Stimulation: Insulin exposure (100 nM).	• Glucose uptake: Measurement of radiolabeled [³H]-2-deoxyglucose uptake. • Protein signaling: Western blot analysis of phosphorylated AKT (pAKT) and phosphorylated AS160 (pAS160). • GLUT4 translocation: Flow cytometry to detect cell-surface GLUT4.	• E4orf1 induction: 24 hours prior to experiments. • Inhibitor treatments: 2 hours for S961 and PPP; 30 minutes for wortmannin. • Insulin stimulation: 20 minutes. • Glucose uptake measurement: Within 5 minutes after addition of 2-deoxyglucose.	• Null vector (NV) cells not expressing E4orf1, subjected to corresponding experimental conditions (± insulin, ± inhibitors).	• E4orf1-enhanced glucose uptake does not require proximal insulin signaling through IR or IGF-1R. • Distal insulin signaling via the PI3K–AKT–AS160 pathway is essential for E4orf1-mediated glucose uptake. • Inhibition of PI3K abolishes the glucose uptake–enhancing effect of E4orf1.	E4orf1.	Stimulation	• E4orf1 bypasses proximal insulin signaling mediated by insulin receptor (IR) and IGF-1 receptor (IGF-1R). • E4orf1 strictly depends on distal insulin signaling through the PI3K–AKT–AS160 axis to drive GLUT4 translocation. • This mechanism allows enhancement of glucose uptake even under conditions of impaired proximal insulin signaling.	Signaling										Low risk	Unclear risk (Not reported)	Low risk	Unclear risk (Not reported)	Unclear risk (Not reported)	Low risk	
A.222	10.1210/endo.141.11.7766	Bandyopadhyay G, 2000	United States (USA)	To evaluate the role of atypical protein kinase C (aPKCs), specifically PKC-ζ/λ, in insulin-stimulated glucose transport and GLUT4 translocation in skeletal muscle cells using adenoviral gene transfer.	L6 myotubes (fully differentiated rat skeletal muscle cells derived from L6 myoblasts).	• Adenoviral gene transfer to express different forms of PKC-λ: • Wild-type PKC-λ. • Constitutively active PKC-λ. • Kinase-defective PKC-λ. • Stimulation: Insulin (100 nM).	• Glucose transport: Measurement of [³H]-2-deoxyglucose uptake. • GLUT4 translocation: Subcellular fractionation followed by plasma membrane Western blotting. • Enzyme activity: Immunoprecipitation kinase assays for PKC-ζ/λ and PKB (Akt).	• Gene expression: 48 hours after adenoviral infection. • Insulin exposure: • 30 minutes for glucose transport and GLUT4 translocation measurements. • 10 minutes for kinase activity assays.	• Cells infected with empty adenovirus (no transgene). • Cells infected with adenovirus encoding β-galactosidase (infection efficiency control). • Comparisons made between basal (no insulin) and insulin-stimulated conditions.	• Atypical PKCs are required and sufficient for insulin-stimulated GLUT4 translocation and glucose transport in L6 myotubes. • Constitutive activation of PKC-λ stimulates glucose transport and GLUT4 translocation in the absence of insulin. • Activation of protein kinase B (PKB/Akt) alone, without concurrent activation of aPKCs, is insufficient to induce glucose transport.	Protein Kinase C-λ (PKC-λ).	Stimulation	• Atypical PKCs (PKC-ζ/λ) function as essential and sufficient mediators of insulin-stimulated GLUT4 translocation and glucose transport in skeletal muscle cells. • Insulin-induced activation of PKB/Akt alone cannot drive glucose transport without concomitant activation of aPKCs. • These findings position aPKCs as indispensable downstream effectors in insulin signaling pathways regulating glucose uptake.	Signaling										Unclear risk (Not reported)	Unclear risk (Not reported)	Low risk	Unclear risk (Not reported)	Unclear risk (Not reported)	Unclear risk (Not reported)	
A.223	10.1371/journal.pone.0051709	Shicheng Cao, 2012	China	To investigate the potential mechanisms by which chronic and acute exercise improve metabolism, as well as the phosphorylation and expression of components of the AMPK signaling pathway and downstream PI3K components (particularly aPKC), in the soleus muscle of diabetic rats.	• Male Sprague–Dawley rats with type 2 diabetes induced by a high-fat diet (HFD) combined with low-dose streptozotocin (STZ). • Tissue examined: soleus muscle.	• Disease model: High-fat diet (HFD) for 20 weeks combined with a single low-dose STZ injection (30 mg/kg) at week 8. • Chronic exercise: Swimming 1 hour/day, 5 days/week, for 8 weeks. • Acute exercise: Two swimming sessions, each lasting 1.5 hours, with a 45-minute rest interval between sessions (performed after 20 weeks of HFD).	• Western blotting to measure protein expression and phosphorylation levels. • Real-time PCR to quantify mRNA expression. • Measurement of glucose uptake using radiolabeled 3-O-methyl-D-glucose (3MG uptake). • Blood biochemical assays (glucose, insulin, triglycerides, etc.).	• Chronic exercise group: Samples collected 24–36 hours after the final exercise session. • Acute exercise group: Samples collected 8–16 hours after exercise.	• Control group (CON): Standard diet. • HFD group: High-fat diet only. • HFD + STZ group: Diabetic rats without exercise intervention.	• Exercise modulates the phosphorylation and expression of components of the AMPK axis and downstream PI3K signaling pathways, including aPKC and Akt. • Both chronic and acute exercise improve GLUT4 trafficking in skeletal muscle.	AMPK, Akt (Akt1, Akt2), aPKC	Stimulation	• Both acute and chronic exercise reverse the diabetes-induced reduction in phosphorylation and expression of aPKC (atypical protein kinase C, specifically PKCζ/λ), a key downstream component of PI3K signaling. • Exercise restores the Akt–AS160 signaling axis, thereby enhancing GLUT4 trafficking in skeletal muscle. • These signaling adaptations contribute to improved glucose homeostasis in HFD+STZ-induced diabetic rat models.	Signaling	Unclear risk (Not reported)	Low risk		Unclear risk (Not reported)	Unclear risk (Not reported)	Unclear risk (Not reported)	Unclear risk (Not reported)	Unclear risk (Not reported)	Unclear risk (Not reported)	Low risk	Low risk	Unclear risk (Not reported)	Low risk	Unclear risk (Not reported)	Unclear risk (Not reported)	Low risk
A.225	10.1242/jcs.113.19.3427	Martin et al., 2000	Australia	To examine the colocalization of GLUT4 and CD-MPR in adipocytes in order to elucidate the mechanism underlying insulin-dependent selective recruitment of GLUT4, distinguishing between the protein sorting model and the vesicle sorting model.	• Adipocytes differentiated from the 3T3-L1 fibroblast cell line.	Stimulation: Insulin (1 μM).	• Immunofluorescence microscopy. • Subcellular fractionation. • Transferrin/horseradish peroxidase (Tf/HRP) ablation technique. • Immuno-electron microscopy (immuno-EM) on cryosections and whole-mount vesicles.	• Measurements performed 15 minutes after insulin stimulation.	Basal condition without insulin or vehicle-treated cells.	• GLUT4 is selectively sorted into a specialized vesicular compartment in adipocytes. • This compartment is specifically recruited to the plasma membrane in response to insulin stimulation.	GLUT4	Stimulation	• The findings support the vesicle sorting model of insulin-regulated GLUT4 trafficking. • GLUT4 is selectively sorted into a distinct population of small vesicles (approximately 30–200 nm in diameter) that are enriched in GLUT4 but contain little or no CD-MPR (GLUT4⁺/CD-MPR⁻). • This insulin-responsive vesicle population is preferentially mobilized to the plasma membrane, whereas vesicles containing both GLUT4 and CD-MPR, associated with the general recycling endosome system, are less sensitive to insulin stimulation.	Translocation										Unclear risk (Not reported)	Unclear risk (Not reported)	Low risk	Unclear risk (Not reported)	Unclear risk (Not reported)	Low risk	
A.226	10.1006/bbrc.1996.1400	Yuasa et al., 2009	Japan	To investigate whether activation of G protein–coupled Gαq receptors (GαqPCRs) promotes AS160 phosphorylation via AMPK; and to determine the role of AS160 in insulin- and Gαq-mediated GLUT4 vesicle distribution	• L6 skeletal muscle cells (L6 myotubes) differentiated from myoblasts. • 3T3-L1 adipocytes differentiated from fibroblasts. • Chinese hamster ovary (CHO) cells stably expressing GLUT4myc. • 3Y1 cells (rat fibroblast	Chemicals/Ligands: Insulin, Norepinephrine (NE – α1bAR receptor activation), Bradykinin (BK – BK2R receptor activation), AICAR (AMPK activator), PMA, Sodium Fluoride (NaF). Genetic interventions: siRNA knockdown (AMPKα1, AS160), exogenous gene expression (wild-type AS160, AS160 4P mutant)	Immunoprecipitation & Western blot: Measurement of phosphorylation levels of AS160, AMPK, Akt, and protein expression. Cell surface anti-c-myc antibody binding assay: Quantification of GLUT4 translocation to the plasma membrane. Immunofluorescence confocal microscopy: Observation of intracellular GLUT4 distributio	Measurement time points varied by experiment: 0, 10, 30, 60 minutes after stimulation.	Unstimulated/untreated cells (indicated by "-" or "control solution" in experiments). Cells transfected with Control siRNA (Negative Control #1 siRNA). Parental 3Y1-G4m cells lacking AS160 expression (used as a control baseline against cells expressing exogenous AS160WT or AS160 4P).	Activation of Gaq protein-coupled receptors (GaqPCRs) promotes the phosphorylation of AS160 via the AMPKa1 catalytic subunit. siRNA-mediated knockdown of AMPKa1 or AS160 promotes intracellular retention of GLUT4 vesicles, lowering basal cell-surface GLUT4. AS160 serves as a common regulator for intracellular GLUT4 vesicle distribution mediated by both insulin/Akt and Gaq/AMPK signaling pathways. AS160 phosphorylation is necessary for the intracellular release and proper distribution of GLUT4 vesicles, but it is not sufficient on its own to trigger insulin-induced GLUT4 translocation.	GαqPCRs	Stimulation	Beyond the established insulin-Akt pathway, AS160 is phosphorylated via a novel GaqPCR-mediated AMPKa1 axis. Under this mechanism, AS160 acts as a common downstream node regulating GLUT4 vesicle retention and spatial redistribution throughout the cytoplasm, integrating energy-sensing Gaq AMPK and nutrient/storage (insulin/Akt) signals.	signaling										Unclear risk (Not reported)	Unclear risk (Not reported)	Low risk	Unclear risk (Not reported)	Unclear risk (Not reported)	Low risk	
A.227	Biochem. J. (1999) 337, 461-470	Gautam Bandyopadhyay et al., 1999	United States of America (USA)	This study compared the effects of transient expression of different protein kinase C (PKC) isoforms—including atypical (ζ, λ), conventional (α, β), and novel (δ, ε) PKCs—on insulin-stimulated GLUT4 translocation in rat adipocytes. The primary focus was to assess the functional redundancy between PKC-ζ and PKC-λ.	• Primary adipocytes isolated from epididymal fat pads of male Sprague–Dawley rats.	• Physiological stimulation: – Insulin (10 nM). • Genetic manipulation (transfection/electroporation): – Expression of PKC wild-type (WT) constructs. – Expression of constitutively active (constit) PKC constructs. – Expression of kinase-inactive (KI) PKC constructs with dominant-negative properties. • PKC isoforms tested: – Atypical: PKC-ζ, PKC-λ – Conventional: PKC-α, PKC-β1, PKC-β2 – Novel: PKC-δ, PKC-ε • Pharmacological interventions: – Wortmannin (PI3K inhibitor). – RO 31-8220. – Gö 6976. – Myristoylated PKC pseudosubstrate peptide	• HAA–GLUT4 translocation assay: – Quantification of epitope-tagged HAA–GLUT4 at the cell surface using anti-HAA antibodies and 125 125 I labeling. • 2-Deoxyglucose (2-DOG) uptake assay: – Measurement of cellular glucose uptake. • Western blotting and immunoprecipitation: – Assessment of PKC protein expression and kinase activity.	• GLUT4 translocation and glucose uptake: – Measured after 30 minutes of insulin stimulation. • Protein expression after transfection: – Assessed 24 hours after gene transfer	• Control/vector group: – Cells transfected with empty vector. • Basal group: – Cells not stimulated with insulin. • Comparison among cells expressing different PKC isoforms – Atypical vs. conventional vs. novel PKCs. • Rescue experiments: – Co-expression of kinase-inactive (KI) and wild-type (WT) PKC isoforms to test functional recovery.	Within the aPKC group, the atypical isoforms ζ and λ play necessary and specific roles in the insulin effect on HAA-GLUT4 translocation, but they cannot substitute for each other.	Atypical protein kinase C isoforms: PKC-ζ and PKC-λ	Stimulation	• Insulin-stimulated GLUT4 translocation specifically requires atypical PKC activity. • PKC-ζ and PKC-λ act at the same signaling level and can substitute for each other functionally. • Classical and novel PKC isoforms do not participate in this insulin-regulated GLUT4 trafficking pathway.	Signaling										Unclear risk (Not reported)	Unclear risk (Not reported)	Low risk	Unclear risk (Not reported)	Unclear risk (Not reported)	Low risk	
A.229	10.1042/BSR20221181	Koester A.M. et al., 2022	United Kingdom (UK)	The study aimed to determine the role of EFR3 and phosphatidylinositol 4-kinase type IIIα (PI4K-IIIα) in insulin-stimulated GLUT4 translocation, with particular focus on the spatial organization and dispersal of GLUT4 within the plasma membrane.	• Differentiated 3T3-L1 adipocytes derived from murine fibroblasts.	• Genetic manipulation: siRNA-mediated knockdown of EFR3 and PI4K-IIIα via electroporation. • Physiological stimulation: – Insulin (100 nM).	• 2-Deoxy-D-glucose uptake assay to assess insulin-stimulated glucose transport. • Direct Stochastic Optical Reconstruction Microscopy (dSTORM) for single-molecule analysis of GLUT4 spatial distribution at the plasma membrane. • Immunoblotting (Western blot) to quantify protein expression levels and confirm knockdown efficiency.	• Analyses performed 72 hours after siRNA electroporation. • Insulin stimulation applied 20–30 minutes prior to measurements.	• Scrambled siRNA (Scr) control cells. • Basal (non-insulin-stimulated) versus insulin-stimulated conditions. • EFR3 knockdown and PI4K-IIIα knockdown compared with Scr control	EFR3 plays a previously unidentified role in controlling insulin-stimulated glucose transport by facilitating the dispersal of GLUT4 within the plasma membrane.	• EFR3 (primarily EFR3A isoform) • Phosphatidylinositol 4-kinase type IIIα (PI4K-IIIα)	Stimulation	• EFR3 functions as a membrane-associated scaffolding protein that recruits and stabilizes PI4K-IIIα at the plasma membrane. • This complex regulates local phosphoinositide organization, enabling insulin-induced dispersal of GLUT4 from membrane clusters into a functionally active, dispersed state. • In the absence of EFR3, GLUT4 translocation to the plasma membrane still occurs, but GLUT4 remains clustered, resulting in impaired glucose transport.	membrane Fusion										Unclear risk (Not reported)	Unclear risk (Not reported)	Low risk	Unclear risk (Not reported)	Unclear risk (Not reported)	Low risk	
A.230	10.1021/bi049970f	Park et al., 2004	Korea	The study aimed to identify previously uncharacterized regulatory proteins associated with insulin-responsive GLUT4 vesicles (IRGTVs) and to elucidate their role in insulin-stimulated recruitment of GLUT4 to the plasma membrane.	The study aimed to identify previously uncharacterized regulatory proteins associated with insulin-responsive GLUT4 vesicles (IRGTVs) and to elucidate their role in insulin-stimulated recruitment of GLUT4 to the plasma membrane.	• Physiological stimulation: – Insulin (100 nM). • Antibody-based intervention: – Treatment with anti-EHD2 antibody in cells permeabilized with streptolysin O (SLO). • Genetic / protein perturbation: – Treatment with dominant-negative GST-ΔEHD2 (EHD2 fragment Glu428–Glu535).	• Subcellular fractionation. • Immunoadsorption of GLUT4-containing vesicles. • MALDI-TOF mass spectrometry for protein identification. • RT-PCR analysis. • Immunoblotting (Western blot). • Co-immunoprecipitation. • GST pull-down assay.	• Analyses performed 20 minutes after insulin stimulation.	• Basal (non-insulin-stimulated) adipocytes. • Insulin-stimulated adipocytes. • Cells treated with normal IgG versus anti-EHD2 antibody. • Cells treated with GST alone versus GST-ΔEHD2.	EPS involves Rab8A, Rab13, and Rab14 but does not depend on Rab10; moreover, Rab10 and Rab13 are not activated by AMPK. C2C12-GLUT4HA cell lines constitute a valuable in vitro model for studying the molecular mechanisms of contraction-stimulated GLUT4 translocation.	EHD2	Stimulation	• EHD2 directly interacts with GLUT4 as well as adaptor complex subunits μ₁ and μ₂ and rCALM. • Insulin enhances the EHD2–GLUT4 interaction, especially in the LDM fraction enriched in GLUT4-containing vesicles. • Functional EHD2 is required for efficient insulin-stimulated recruitment of GLUT4 to the plasma membrane.	Translocation										Unclear risk (Not reported)	Unclear risk (Not reported)	Low risk	Unclear risk (Not reported)	Unclear risk (Not reported)	Low risk	
A.232	10.1152/ajpendo.00103.2017	Zhu Li, 2018	China	The study identified Rab GTPases involved in GLUT4 trafficking during muscle contraction (simulated by electrical pulse stimulation, EPS) and validated the suitability of the C2C12-GLUT4HA cell model for investigating upstream signaling mechanisms.	• Differentiated C2C12 myotubes stably expressing HA-tagged GLUT4 (C2C12-GLUT4HA)	• Physiological stimulation: – Electrical pulse stimulation (EPS) to induce muscle contraction (60 minutes, 20 V, 1 Hz, 24 ms pulse duration). • Pharmacological treatments: – Insulin. – AICAR (AMPK activator). – BTS (myosin II inhibitor). – Compound C (AMPK inhibitor). – KN93 (CaMKII inhibitor). • Genetic interventions: – siRNA knockdown of AMPK, LKB1, CaMKII, Rab8a, Rab10, Rab13, and Rab14. – Expression of the non-phosphorylatable AS160 mutant (AS160-4A	• Colorimetric assay using anti-HA antibody conjugated with HRP to quantify cell-surface GLUT4. • Western blot analysis to assess phosphorylation of signaling proteins (AMPK, ACC, CaMKII, AS160, TBC1D1). • 2-NBDG uptake assay to measure glucose uptake.	• Immediately after completion of 60 minutes of EPS. • 20 minutes after insulin treatment. • 60 minutes after AICAR treatment.	• Basal (unstimulated) group. • Insulin-treated group. • AICAR-treated group. • Vehicle control (DMSO). • Non-targeting siRNA control (siNR). • Wild-type C2C12 cells.	• EPS-induced GLUT4 translocation involves Rab8a, Rab13, and Rab14, but not Rab10. • Rab10 and Rab13 are not activated by AMPK stimulation alone. • C2C12-GLUT4HA cells represent a valuable in vitro model for studying molecular mechanisms of contraction-stimulated GLUT4 translocation.	Rab8a, Rab13, Rab14	Stimulation	• Contraction-induced GLUT4 translocation via EPS activates two parallel signaling pathways: LKB1–AMPK and CaMKII. • Both pathways converge on phosphorylation of AS160 and TBC1D1. • EPS specifically requires Rab8a, Rab13, and Rab14, whereas Rab10 is not required. • Activation of AMPK alone (by AICAR) selectively engages Rab8a and Rab14 but not Rab13	Signaling										Unclear risk (Not reported)	Unclear risk (Not reported)	Low risk	Unclear risk (Not reported)	Unclear risk (Not reported)	Low risk	
A.234	10.1074/jbc.M112.339457	Jonathan S. Bogan, 2012	United States (USA)	The study investigated the mechanism by which TUG regulates GLUT4 translocation, specifically testing the hypothesis that TUG undergoes site-specific endoproteolytic cleavage to release GLUT4 storage vesicles (GSVs).	• Differentiated 3T3-L1 adipocytes.	• Insulin stimulation. • RNA interference (shRNA/siRNA) to deplete TUG and TC10a. • Expression of cleavage-resistant TUG mutants (TUG GGAA, TUG S165M). • Treatment with the proteasome inhibitor MG-132.	• Immunoblotting (Western blot) to detect TUG cleavage fragments. • Immunoprecipitation. • Pulse-chase experiments. • Subcellular fractionation (plasma membrane, low-density membranes, high-density membranes). • Confocal microscopy. • Glucose uptake assay (2-deoxyglucose uptake).	• Insulin stimulation times: – 0, 2, 5, 10, and 20 minutes (for immunoblot analysis). – 15 minutes (for GLUT4 translocation). – 40 minutes (in the presence of MG-132). • Cell differentiation stages analyzed: day 0, day 5, and day 10.	• Unstimulated (basal) cells. • Control cells (mock-transfected). • Cells expressing TUG shRNA (endogenous TUG depleted). • Rescue cells expressing wild-type TUG or cleavage-resistant TUG mutants (GGAA, S165M).	• Cleavage-resistant forms of TUG do not support insulin-responsive GLUT4 translocation or glucose uptake in 3T3-L1 adipocytes. • Insulin activates TUG cleavage, which releases GLUT4 storage vesicles from the Golgi matrix and thereby promotes GLUT4 translocation to the cell surface.	Protein TUG (Tether containing a UBX domain for GLUT4).	Stimulation	• Insulin stimulates site-specific endoproteolytic cleavage of TUG at the junction between amino acids 164 and 165. • This cleavage separates the N-terminal domain (which binds GLUT4) from the C-terminal domain (which binds PIST and Golgin-160 at the Golgi matrix). • As a result, GLUT4 storage vesicles (GSVs) are released from intracellular tethering and are able to translocate to the plasma membrane. • This cleavage process is dependent on signaling through TC10a.	Translocation										Unclear risk (Not reported)	Unclear risk (Not reported)	Low risk	Unclear risk (Not reported)	Unclear risk (Not reported)	Low risk	
A.240	10.1074/jbc.M004046200	Mathias Fasshauer, 2000	United States (USA)	The study investigated the specific physiological role of IRS-2 in insulin-stimulated Glut4 translocation and glucose uptake in brown adipocytes.	• Brown adipocytes differentiated from preadipocytes isolated from neonatal wild-type (WT) mice and IRS-2 knockout (IRS-2 KO) mice	• Physiological stimulation: insulin (concentrations ranging from 0.1 nM to 1000 nM). • Pharmacological intervention: wortmannin (PI 3-kinase inhibitor). • Genetic interventions: knockout of the Irs2 gene, re-expression of IRS-2 via retroviral infection, and overexpression of IRS-1.	• Glucose uptake assays using 2-deoxy-[³H]glucose and measurement of radioactivity. • Glut4 translocation: subcellular fractionation into plasma membrane (PM) and low-density microsome (LDM) fractions, followed by Western blot analysis. • Signaling analysis: immunoprecipitation and Western blotting to assess IRS-1, IRS-2, Akt, and PI 3-kinase activity.	• Glucose uptake measured after insulin incubation for 5, 10, 20, 30, or 60 minutes. • Glut4 translocation assessed after 20 minutes of insulin stimulation.	• Wild-type (WT) brown adipocytes versus IRS-2–deficient (IRS-2 KO) brown adipocytes. • IRS-2 KO cells versus IRS-2 KO cells re-expressing IRS-2 or overexpressing IRS-1.	• IRS-2 acts as a mediator of insulin-stimulated GLUT4 translocation and glucose uptake in adipocytes. • This defect occurs without loss of total Akt activation or its downstream signaling, and may instead arise from altered compartmentalization of these downstream signals.	IRS-2	Stimulation	• IRS-2 accounts for a substantial fraction (~30%) of phosphotyrosine-associated PI 3-kinase activity and plays a critical role in targeting Akt to the plasma membrane. • IRS-2 deficiency reduces Akt abundance at the plasma membrane (despite unchanged total Akt activity), leading to impaired Glut4 translocation. • IRS-1 cannot compensate for this specific function of IRS-2 due to differences in signaling compartmentalization.	Signaling										Unclear risk (Not reported)	Unclear risk (Not reported)	Unclear risk (Not reported)	Unclear risk (Not reported)	Unclear risk (Not reported)	Unclear risk (Not reported)	
A.241	10.1016/S0014-5793(03)00049-8	Mónica Arribas et al., 2003	Spain	The study investigated the molecular mechanisms by which IRS-2 deficiency in brown adipocytes leads to impaired insulin-stimulated GLUT4 translocation and glucose uptake.	• Immortalized brown adipocyte cell lines isolated from brown adipose tissue of 3-day-old neonatal mice with three genotypes: – Wild-type (IRS-2+/+) – Heterozygous (IRS-2+/−) – IRS-2–deficient (IRS-2−/−) • Primary brown adipocytes were also used for validation.	• Insulin stimulation: cells treated with insulin at concentrations of 10–100 nM. • Genetic deficiency: deletion of the IRS-2 gene (IRS-2−/−). • Genetic interventions: (i) Reconstitution of IRS-2 expression using retroviral vectors; (ii) Expression of a kinase-inactive PKCζ mutant (KI-PKCζ) via adenoviral vectors.	• Analysis of cell signaling (phosphorylation status and enzyme activity). • Glucose transport assays. • Western blot analysis for protein expression.	• Cell signaling (phosphorylation / enzyme activity): measured 5 minutes after insulin stimulation. • Glucose transport: measured 10 minutes after insulin stimulation. • Protein expression after viral infection: analyzed 48 hours post-infection.	• Wild-type cells: IRS-2+/+. • Heterozygous cells: IRS-2+/−. • IRS-2–reconstituted cells: rec-IRS-2. • Empty-vector–infected cells: mock.	• PKCζ plays an essential role in insulin-stimulated glucose uptake, which is impaired in IRS-2–deficient brown adipocytes.	IRS-2 (Insulin Receptor Substrate-2), PKCζ (Protein Kinase C zeta), GLUT4.	Stimulation	• The IRS-2 / PI 3-kinase / PKCζ signaling pathway is essential and specific for insulin-stimulated glucose transport and GLUT4 translocation in brown adipocytes. • IRS-2 deficiency does not affect IRS-1 signaling or activation of Akt, GSK-3, or p70S6k, yet still results in impaired glucose uptake. • This demonstrates signaling divergence, in which PKCζ—rather than Akt—acts as the decisive downstream effector of IRS-2 in regulating GLUT4 translocation.	Signaling (IRS-2 → PI3K → PKCζ)										Unclear risk (Not reported)	Unclear risk (Not reported)	Low risk	Unclear risk (Not reported)	Unclear risk (Not reported)	Low risk	
A.242	10.1016/s0014-5793(98)00293-2	S. Lund et al., 1998	Denmark	The study examined whether Protein Kinase B (PKB/Akt) is a common convergence point in distinct signaling pathways leading to glucose transport and GLUT4 translocation induced by insulin and by muscle contraction	• Intact soleus muscle isolated from male Wistar rats (body weight 55–65 g)	• Insulin stimulation (1 mU/ml). • Muscle contraction induced by electrical stimulation (10 Hz, 5 minutes). • Use of wortmannin (1 μM) to inhibit PI 3-kinase.	• Measurement of glucose transport activity via accumulation of 3-O-[³H] methyl-D-glucose (3-OMG). • Measurement of cell-surface GLUT4 content using photolabeling with ATB-[³H] BMPA. • Measurement of Protein Kinase B (PKB) activity by immunoprecipitation followed by incorporation of radioactive phosphate ([γ-³²P]ATP).	• Immediately after 20 minutes of insulin incubation. • Immediately after 5 minutes of muscle contraction stimulation	• Basal group: muscles incubated in the absence of stimulatory agents.	• PKB is not a mediator of contraction-induced glucose transport and GLUT4 translocation	Protein Kinase B (PKB/Akt)	Stimulation	• The study provides negative evidence demonstrating that PKB does not mediate contraction-induced glucose transport and GLUT4 translocation, in contrast to insulin-stimulated pathways. • This confirms that muscle contraction activates glucose transport through a signaling pathway independent of the PI3K–PKB axi	Signaling										Unclear risk (Not reported)	Unclear risk (Not reported)	Unclear risk (Not reported)	Unclear risk (Not reported)	Unclear risk (Not reported)	Unclear risk (Not reported)	
A.243	10.1074/jbc.M409928200	Ewart MA et al., 2005	United Kingdom (UK)	The study aimed to determine the role of two exocyst components, rsec6 and rsec8, in insulin-stimulated Glut4 trafficking and glucose uptake in adipocytes.	• 3T3-L1 adipocytes.	• Insulin stimulation. • Overexpression of rsec6 or rsec8 using adenoviral vectors.	• 2-Deoxy-D-glucose transport assay. • Western blot analysis (immunoblotting) of cellular fractions	• Glucose transport measured after insulin incubation for 0–25 minutes. • Subcellular fractionation performed after 30 minutes of insulin stimulati	• Basal cells without insulin. • Cells infected with empty adenovirus (control / empty virus)	• The exocyst complex plays a role in insulin-stimulated glucose transport. • A model is proposed in which insulin-dependent relocation of the exocyst to the plasma membrane contributes to the specificity of GLUT4 vesicle docking and fusion with the adipocyte plasma membrane.	rsec6 and rsec8 (components of the exocyst complex)	Stimulation	• Insulin promotes redistribution of the exocyst complex (rsec6 and rsec8) to the plasma membrane and cytoskeletal fractions, thereby positioning Glut4-containing vesicles at the cell surface.	Tethering										Unclear risk (Not reported)	Unclear risk (Not reported)	Low risk	Unclear risk (Not reported)	Unclear risk (Not reported)	Low risk	
A.244	10.1073/pnas.93.16.8401	Morris AJ et al., 1996	United States (USA)	The study investigated the role of the interaction between the insulin receptor (IR) and insulin receptor substrate 1 (IRS-1) in the insulin signaling pathway that stimulates glucose transport (GLUT4 translocation).	• 3T3-L1 adipocytes.	• Microinjection of competitive IRS-1 inhibitors, including MBP–IRS-1 fusion protein, anti–IRS-1 antibodies, or NPXpY phosphopeptides, followed by insulin stimulation.	• Immunofluorescence microscopy to visualize GLUT4 translocation to the plasma membrane.	• Cells were incubated for 1 hour after microinjection of inhibitory agents. • Cells were then treated with insulin for 20 minutes prior to fixation and analysi	• Basal (unstimulated) cells. • Cells microinjected with sheep IgG (control) instead of inhibitory agents.	• Phosphorylated IRS-1 is not an essential component of the metabolic insulin signaling pathway leading to GLUT4 translocation, although it appears to be required for other biological effects of insulin.	IRS-1	No inhibition (Blockade of IRS-1 function does not reduce insulin-stimulated GLUT4 translocation.	• Insulin-stimulated GLUT4 translocation can occur via an IRS-1–independent signaling pathway	Signaling										Unclear risk (Not reported)	Unclear risk (Not reported)	Low risk	Unclear risk (Not reported)	Unclear risk (Not reported)	Low risk	
A.247	10.1038/ncb2226	Xiao-Wei Chen et al., 2011	United States (USA)	The study investigated the regulatory mechanism governing the interaction between RalA and the exocyst complex (specifically the Sec5 subunit) and clarified the mechanism of their disengagement to allow vesicle fusion during exocytosis (including GLUT4 trafficking).	• Differentiated 3T3-L1 adipocytes (used for GLUT4 studies). • Cos-1 cells. • MDCK cells. • Zebrafish embryos.	• Stimulation: insulin (in 3T3-L1 cells), EGF (in Cos-1 cells), TPA (PKC activator). • Inhibition: PKC inhibitors (G06976, Ro 31-8220, G06850), BAPTA-AM (Ca²⁺ chelator). • Genetic manipulation: expression of RalA mutants (G23V – constitutively active), Sec5 mutants (S89A – non-phosphorylatable, S89D – phosphomimetic), and RNAi knockdown (siRNA).	• Glucose uptake assay. • Western blot analysis (protein levels and phosphorylation). • Immunoprecipitation (IP). • Total internal reflection fluorescence microscopy (TIRFM) to visualize fusion of GLUT4/TfR-containing vesicles. • Cell-surface immunostaining to detect Myc-GLUT4 translocation.	• Glucose uptake: basal and after insulin stimulation. • Phosphorylation assays: 5 minutes after EGF treatment or 30 minutes after calyculin A treatment. • Vesicle fusion (TIRFM): real-time time-series measurements.	• Control / basal groups. • Cells expressing wild-type (WT) Sec5 or WT RalA.	• The exocyst functions as a "gatekeeper" for exocytic vesicles through a regulatory circuit involving engagement, disengagement, and re-engagement with G proteins	Sec5 (exocyst subunit), RalA (GTPase)	Stimulation	• Gatekeeper mechanism: after RalA (on vesicles) binds to Sec5 (within the exocyst complex), PKC phosphorylates Sec5 at Ser89. • This phosphorylation induces an allosteric conformational change that causes Sec5 to disengage from RalA. • This disengagement is required for vesicle fusion with the plasma membrane. • Contrary to previous assumptions, RalA does not need to hydrolyze GTP to disengage; instead, disengagement is driven by Sec5 phosphorylation.	Tethering										Unclear risk (Not reported)	Unclear risk (Not reported)	Low risk	Unclear risk (Not reported)	Unclear risk (Not reported)	Low risk	
A.248	10.1042/0264-6021:3560143	Mireille Cormont, 2001	France	The study sought to identify the specific role of Rab4 in the final steps of GLUT4 translocation	• Adipocytes isolated from the epididymal fat pads of male Wistar rats.	• Overexpression of Rab4 ΔCT (a cytosolic, non-prenylated form). • Overexpression of wild-type Rab4 (WT). • Overexpression of Rab4 S22N ΔCT (GTP-binding–deficient mutant). • Stimulatory conditions included: insulin, zinc sulfate (Zn²⁺), sorbitol (osmotic shock), expression of p110* (constitutively active PI3-kinase), and Gag-PKB (constitutively active PKB).	• Cell-surface epitope-tagged GLUT4 assay (quantification of Myc-tagged GLUT4 at the plasma membrane). • PI 3-kinase activity assay. • PKB kinase activity assay. • Immunoprecipitation. • Immunoblotting (Western blot).	• Cells were incubated for 16–24 hours after gene transfer (electroporation). • Cells were then treated with stimuli for 20–30 minutes (insulin, sorbitol, zinc) prior to measurements.	• Control group expressing GLUT4-Myc alone (without Rab4 ΔCT). • Basal group (no insulin). • Empty-vector–transfected control group.	• Rab4 ΔCT inhibits GLUT4 translocation induced by insulin, osmotic shock (sorbitol), constitutively active PI3-kinase (p110*), and constitutively active PKB (Gag-PKB). • Rab4 ΔCT does not inhibit GLUT4 translocation induced by zinc (Zn²⁺).	Rab4 ΔCT	Inhibition	• Cytosolic Rab4 ΔCT inhibits GLUT4 translocation only when it retains GTP-binding competence. • Rab4 ΔCT acts downstream of PI 3-kinase and PKB, blocking GLUT4 translocation without altering PI3K or PKB enzymatic activity.	Signaling / Translocation (Study identifies Rab4 action at the translocation stage, specifically downstream of PI3K/PKB activation.)										Unclear risk	Unclear risk	Low risk	Unclear risk	Unclear risk	Low risk	
A.250	10.1074/jbc.M409806200	Craig A. Eyster, 2005	United States (USA)	The study distinguished whether the actin cytoskeleton participates in the formation of insulin signaling complexes or is required for the movement of storage vesicles to the plasma membrane, and aimed to determine whether actin acts upstream of Akt/PKB activation (signaling complex formation) or downstream of Akt/PKB activation.	• Adipocytes differentiated from the 3T3-L1 fibroblast cell line	• Expression of myr-Akt (a constitutively active, membrane-targeted form of Akt). • Treatment with latrunculin B (an actin depolymerizing agent). • Insulin stimulation.	• GLUT4 translocation assay: confocal microscopy using HA-GLUT4-GFP. • GLUT4 recycling assay. • Western blot analysis to assess phosphorylation. • Kinase activity assays for PI 3-kinase and PKB/Akt.	• Experiments performed 18–24 hours after gene transfer (electroporation). • Latrunculin B treatment for 2 hours. • Insulin stimulation for 30 minutes (or time course 0–30 minutes).	• Basal group. • Control plasmid group versus myr-Akt–expressing group. • No latrunculin B treatment versus latrunculin B–treated group	• Latrunculin B reduces PI 3-kinase activity and inhibits phosphorylation/activation of endogenous Akt.	Actin, Akt	Stimulation	• The actin cytoskeleton plays a primary role in organizing insulin signaling complexes, specifically the steps leading to Akt activation such as phosphorylation at Thr-308 and Ser-473. • An intact actin network is not strictly required for the movement or fusion of GLUT4 vesicles once Akt has already been activated. • When Akt is constitutively active (e.g., myr-Akt), GLUT4 can still translocate to the plasma membrane even in the absence of an intact actin cytoskeleton.	Signaling, translocation										Unclear risk	Unclear risk	Low risk	Unclear risk	Unclear risk	Low risk	

A.251	10.1210/me.2003-0261	Claire Chaussade, 2003	France and Switzerland	The study used adenoviral vectors to express myotubularin (MTM1) in muscle and adipocyte cell lines to determine its biochemical function as a PtdIns(3)P phosphatase and to define the role of PtdIns(3)P in insulin-stimulated glucose transport.	• Muscle cell lines: L6 myotubes, C2C12 myotubes.<br>• Adipocyte cell lines: 3T3-L1 adipocytes (specifically the CAR"delta"1 line).	• Ectopic gene expression via adenovirus of myotubularin variants: wild-type (wt), catalytically inactive mutant (C375S), and substrate-trapping mutant (D278A).<br>• Insulin stimulation.	• Radiolabeling with [^{32}P]orthophosphate and phosphoinositide analysis by HPLC.<br>• Western blot analysis.<br>• Confocal fluorescence microscopy to track EEA1 and GLUT4-EGFP.<br>• Glucose uptake assay using [^{3}H]2-deoxyglucose.	• Cells analyzed 48 hours (or 3 days) after adenoviral infection.<br>• Insulin stimulation for 10 minutes for glucose uptake assays.	• Uninfected cells or empty-vector–infected cells versus cells infected with adenovirus expressing MTM1 wt, MTM1 D278A, or MTM1 C375S.<br>• Basal (no insulin) versus insulin-stimulated conditions	• The presence of myotubularin reduces PtdIns(3)P levels and inhibits insulin-stimulated GLUT4 translocation and glucose uptake.<br>• Increasing PtdIns(3)P levels (via D278A and C375S mutants that block myotubularin action on PtdIns(3)P) increases basal glucose uptake, but does not affect maximal insulin-stimulated glucose uptake.	PtdIns(3)P, Myotubularin (MTM1)	PtdIns(3)P: Stimulation Myotubularin (MTM1): Inhibition	• PtdIns(3)P is a required signaling molecule for directing GLUT4 to insulin-responsive compartments or enabling its translocation to the plasma membrane.<br>• Myotubularin regulates this process by reducing PtdIns(3)P levels, thereby antagonizing PI 3-kinase activity.<br>• Depletion of PtdIns(3)P (via MTM1 wt expression) prevents GLUT4 from reaching the correct compartments for exocytosis.	Signaling										Unclear risk	Unclear risk	Low risk	Unclear risk	Unclear risk	Unclear risk	
A.252	10.1186/1471-2121-9-48	Bharti Bisht, 2008	India	The study investigated whether Focal Adhesion Kinase (FAK) contributes to insulin-stimulated actin remodeling that accommodates GLUT4 for glucose transport, and whether reduced FAK expression impairs this process and leads to insulin resistance.	• C2C12 skeletal muscle cells differentiated into myotubes, studied under two conditions: insulin-sensitive (MF) and insulin-resistant (MFI).	• FAK overexpression (gain of function) by transfection with plasmids encoding wild-type FAK.<br>• FAK knockdown (loss of function) using specific siRNA.<br>• Insulin stimulation.<br>• PI3K inhibition using Wortmannin (WM)	• Immunofluorescence microscopy and confocal microscopy to assess actin remodeling, FAK localization, and GLUT4 translocation.<br>• Western blot analysis to determine protein expression levels.<br>• Glucose uptake assay using 2-deoxyglucose (2-DOG).	• Cells were serum-starved for 4 hours prior to insulin stimulation.<br>• Insulin stimulation for 30 minutes for microscopy and glucose uptake experiments.	• Insulin-sensitive (MF) versus insulin-resistant (MFI) cells.<br>• Basal (no insulin) versus insulin-stimulated conditions.<br>• Untransfected cells versus FAK-overexpressing cells versus FAK-silenced cells.<br>• No drug treatment versus Wortmannin-treated conditions.	• Insulin stimulation induces redistribution of FAK and its colocalization with remodeled actin.<br>• FAK overexpression enhances actin remodeling and GLUT4 translocation, even under insulin-resistant conditions.<br>• FAK silencing inhibits actin remodeling and GLUT4 translocation, resulting in insulin resistance.<br>• Wortmannin abolishes the effects of FAK on actin remodeling and GLUT4 translocation.	FAK	Stimulation	• FAK plays a central role in regulating insulin-stimulated actin remodeling into a mesh-like structure that accommodates and transports GLUT4 to the plasma membrane via a PI3K-dependent pathway.<br>• Under insulin-resistant conditions, impaired FAK activity or localization disrupts actin remodeling, leading to reduced GLUT4 translocation.	Translocation										Unclear risk	Unclear risk	Low risk	Unclear risk	Unclear risk	Low risk	
A.253	10.1096/fj.202402764RR	Franziska Kopietz, 2025	Sweden	The study investigated the role of Focal Adhesion Kinase (FAK) in glucose transport in adipocytes and explored the impact of acute FAK inhibition on key intermediate mechanisms leading to insulin-stimulated glucose uptake.	• Primary adipocytes isolated from subcutaneous or epididymal adipose tissue of male C57BL/6J mice.<br>• Primary adipocytes isolated from human abdominal subcutaneous adipose tissue.	• FAK inhibition using the pharmacological inhibitor PF-573228 (FAKi).<br>• Inhibition of the Arp2/3 complex (an actin-dependent regulator of GLUT4 translocation) using CK666.<br>• Rac1 activation using CN04.<br>• Insulin stimulation.	• Affinity purification combined with quantitative mass spectrometry (IP-MS) to monitor FAK protein–protein interactions.<br>• Glucose uptake assays (glucose tracer assays using D-[^{14}C]-glucose).<br>• Western blot analysis.<br>• Confocal microscopy to visualize GLUT4 and F-actin.<br>• Rac1 activity assay (G-LISA).	• Cells were pre-incubated with inhibitors for 1 hour.<br>• Insulin stimulation for 30 minutes (for glucose uptake / GLUT4 translocation).<br>• Insulin stimulation for 5 minutes (for kinase signaling and Rac1 activity)	• DMSO control group versus FAK inhibitor–treated group (PF/FAKi).<br>• Basal (no insulin) versus insulin-stimulated conditions.<br>• Arp2/3 inhibition group (CK666) versus control.<br>• Rac1 activation group (CN04) versus control.	• FAK inhibition reduces FAK interactions with actin-associated proteins (such as Arp2/3), decreases Rac1 activity, and reduces phosphorylation of PAK1/2.<br>• FAK inhibition impairs GLUT4 translocation to the plasma membrane and reduces glucose uptake.<br>• Notably, FAK inhibition does not affect phosphorylation of canonical insulin signaling molecules such as IRS-1, PKB/Akt, or AS160.<br>• Rac1 activation partially rescues the reduction in glucose uptake caused by FAK inhibition.	FAK (pY397)	Stimulation	• FAK coordinates glucose transport and GLUT4 translocation by controlling actin cytoskeletal turnover rather than through canonical insulin signaling pathways (IRS/PKB/AS160).<br>• FAK functions as a signaling hub to activate Rac1 and interact with the Arp2/3 complex, thereby regulating actin dynamics required for GLUT4 trafficking.	Translocation										Unclear risk	Unclear risk	Low risk	Unclear risk	Unclear risk	Low risk	
A.254	10.1016/j.cmet.2005.09.005	Lorena Eguez, 2005	United States (USA)	The study aimed to determine the role of AS160 (a Rab GTPase-activating protein, GAP) in regulating GLUT4 retention and exocytosis under basal conditions and upon insulin stimulation in adipocytes.	• Adipocytes differentiated from the 3T3-L1 cell line.	• AS160 knockdown (KD) using retrovirus containing shRNA (AS160 KD1, KD2).<br>• Rescue by re-expression of wild-type human AS160.<br>• Expression of GAP-domain mutant AS160 (AS160 R/K mutant).<br>• Expression of dominant-interfering AS160 (DI-AS160 / 4P).<br>• Insulin stimulation.<br>• Wortmannin treatment (PI3K inhibitor).	• Western blot: to determine the extent of AS160 knockdown and levels of other proteins (GLUT4, IRAP, Actin).<br>• Indirect immunofluorescence microscopy / fluorescence quantification: using the HA-GLUT4-GFP reporter to measure the surface-to-total distribution of GLUT4 at the single-cell level.<br>• Exocytosis kinetics assay: measurement of the rate of HA-GLUT4-GFP appearance at the plasma membrane over time using the HA.11 antibody.<br>• Hexose transport assay: use of [^{3}H]2-deoxyglucose to measure glucose transport activity.<br>• HRP ablation assay: to determine the intracellular distribution of GLUT4 between endosomes (transferrin receptor–containing, TR) and specialized compartments (IRAP-containing).	• Adipocytes were used on day 5 or day 10 after differentiation.<br>• Insulin stimulation for 30 minutes (steady-state conditions) or across time courses (kinetic studies).	• Control shRNA group versus AS160 shRNA KD groups (KD1, KD2).<br>• Basal versus insulin-stimulated conditions.<br>• AS160 KD without rescue versus AS160 KD rescued with WT AS160 or R/K mutant.<br>• DI-AS160–expressing cells versus control cells.	• Inhibition of AS160, specifically at the GAP domain, stimulates basal GLUT4 exocytosis (approximately threefold).<br>• AS160 knockdown causes redistribution of GLUT4 from the specialized IRAP-containing compartment (an insulin-regulated aminopeptidase that traffics with GLUT4) to endosomes.<br>• Expression of dominant-interfering AS160 (DI-AS160) reveals biphasic insulin-stimulated GLUT4 translocation: a rapid initial increase that is not sustained, followed by a slow later phase that nevertheless reaches a steady-state level similar to control cells.	AS160 (TBC1D4)	Inhibition	• The GAP activity of AS160 is required to maintain intracellular GLUT4 retention under basal conditions (basal retention).<br>• AS160 functions as a negative regulator of GLUT4 exocytosis.<br>• However, AS160 is not the sole gatekeeper; insulin stimulates GLUT4 exocytosis through both AS160-dependent and AS160-independent mechanisms.	Signaling										Unclear risk	Unclear risk	Low risk	Unclear risk	Unclear risk	Unclear risk	
A.256	10.1242/jcs.115.14.2857	Pierre-Henri Ducluzeau, 2002	United Kingdom (UK)	The study investigated the functional role of Protein Kinase B (PKB/Akt), specifically at the level of GLUT4 vesicles, in insulin-stimulated translocation to the plasma membrane.	• Adipocytes differentiated from the mouse 3T3-L1 cell line.	• Microinjection of plasmids encoding mutant PKB fusion proteins targeted to GLUT4 vesicles (PKB[KD]-GLUT4 or PKB[DD]-GLUT4) or IRAP-GFP; followed by insulin treatment.	• Laser-scanning confocal microscopy: visualization of the distribution and translocation of fluorescent proteins (GFP) and immunolocalization.<br>• Immunofluorescence: detection of HA-tagged proteins and endogenous IRAP expression.<br>• Quantification of translocation: counting cells exhibiting a clear fluorescent rim at the plasma membrane (IRAP-GFP rim) and measuring membrane fluorescence intensity.	• Measurements performed under basal conditions and after 30 minutes of insulin stimulation.	• Vector-only microinjection control group.<br>• Cells expressing cytosolic inactive PKB mutant (cytosolic PKB[KD]) not targeted to GLUT4 vesicles.<br>• Cells expressing inactive PKB mutant lacking the PH domain (ΔPH-PKB[KD])	• Cytosolic PKB[KD] exhibits little inhibitory activity; however, when directly targeted to GLUT4 vesicles, it exerts a strong inhibitory effect.<br>• Cytosolic PKB[DD] strongly stimulates exocytosis, does not synergize with insulin, whereas PKB[DD]-GLUT4 shows weaker stimulatory effects but exhibits additivity with insulin	Akt (PKB)	Stimulation	• Akt stimulates GLUT4 exocytosis by acting directly on GLUT4 vesicles or on local effectors in proximity to GLUT4 (localized action).<br>• In addition, Akt localization at the plasma membrane is also required to support GLUT4 exocytosis	Signaling										Unclear risk	Unclear risk	Low risk	Unclear risk	Unclear risk	Unclear risk	
A.257	10.1152/ajpcell.00105.2012	Diego Sbrissa, 2012	United States (USA)	The study aimed to determine the role of the PtdIns5P pool synthesized by PIKfyve in insulin-stimulated actin stress fiber disassembly, using the PIKfyve inhibitor YM201636 that targets PtdIns5P and PtdIns(3,5)P2.	• Differentiated 3T3-L1 adipocytes (3T3L1 adipocytes).<br>• Human embryonic kidney cells (HEK293).<br>• Chinese hamster ovary cells (CHO-T).	• Use of the inhibitor YM201636 at low concentrations (10–25 nM in vitro; 160 nM in vivo) and high concentration (800 nM in vivo).<br>• Insulin stimulation.	• In vitro lipid kinase assay & TLC (thin-layer chromatography): measurement of phosphorylated lipid products.<br>• HPLC (high-performance liquid chromatography): quantitative analysis of phosphoinositides (PIs) in radiolabeled cells.<br>• Fluorescence microscopy: visualization of the F-actin cytoskeleton (rhodamine–phalloidin staining).<br>• Immunoblotting: protein analysis (PIKfyve, Akt, Dok1…).	• YM201636 inhibitor incubation: 30–40 minutes.<br>• Insulin stimulation: 10 minutes.	• Vehicle-treated control group (vehicle/DMSO).<br>• Wortmannin-treated group (PI3K inhibitor) for specificity comparison.	• YM201636 inhibits PIKfyve-mediated synthesis of PtdIns5P more strongly than that of PtdIns(3,5)P2.<br>• PIKfyve-catalyzed PtdIns5P mediates insulin-induced F-actin stress fiber disassembly.<br>• Dok1 is not involved in the effects controlled by PIKfyve-derived PtdIns5P.	PIKfyve, PtdIns5P	Stimulation	• PtdIns5P generated by PIKfyve mediates insulin-stimulated remodeling of the actin cytoskeleton	Translocation										Unclear risk	Unclear risk	Low risk	Unclear risk	Unclear risk	Low risk	
A.258	10.1007/s00125-009-1298-7	D. S. Chappell, 2009	United States (USA)	The study aimed to determine the functional role of PKCβII and its substrate MARCKS in the molecular mechanism of insulin-stimulated glucose transport, specifically testing the hypothesis that PKCβII regulates this process through phosphorylation of MARCKS.	• L6 rat skeletal muscle cells (L6 myotubes).	• Stimulation: Insulin.<br>• Inhibition: use of pharmacological inhibitors (LY294002 for PI3K, CG53353 for PKCβII, W13 for calmodulin); use of siRNA to reduce PKCβII and MARCKS expression; transfection to express mutant MARCKS protein (S152A).	• Western blot analysis: measurement of protein phosphorylation and expression (PKCβII, MARCKS, Akt, PLD1…).<br>• 2-deoxyglucose uptake assay: measurement of glucose transport.<br>• Immunoprecipitation (with anti-actin antibody): determination of interactions between actin and PKCβII, GLUT4, and MARCKS.<br>• Subcellular fractionation: separation of cytosolic and membrane fractions to track protein translocation.<br>• Trypsin digestion assay: assessment of GLUT4 insertion into the plasma membrane.	• Insulin treatment: 15 minutes, 30 minutes.<br>• Glucose uptake measurement: after 6 minutes of incubation with substrate.<br>• Inhibitor treatment: 30 minutes or 2 hours prior to stimulatio	• Basal / BSA control group (no insulin treatment).<br>• Scrambled siRNA or control siRNA–treated groups.<br>• Empty vector–transfected group (pcDNA3).	• PKCβII is activated by insulin via PI3K.<br>• Insulin induces MARCKS phosphorylation through PKCβII and Ca^{2+}–calmodulin.<br>• Overexpression of mutant MARCKS inhibits glucose uptake.	Protein Kinase C-betaII (PKCβII) and MARCKS (Myristoylated alanine-rich C-kinase substrate).	Stimulation	• Insulin activates PI3K → PI3K activates PKCβII → PKCβII activates MARCKS → MARCKS stimulates GLUT4 exocytosis (potentially via activation of PLD (phospholipase D) involved in GLUT4 docking).	Signaling										Unclear risk	Unclear risk	Low risk	Unclear risk	Unclear risk	Low risk	
A.260	10.1042/bj3240217	S. Lance Macaulay, 1997	Australia	The study evaluated the functional involvement of SNARE proteins (including VAMP2, SNAP-25, and syntaxin 4) in GLUT4 trafficking by using protein-cleaving neurotoxins and specific competitive peptides.	• Differentiated 3T3-L1 adipocytes.	• Microinjection of toxins:<br>– Tetanus toxin light chain (TeTx) and Botulinum D toxin light chain (BoNT/D) to cleave VAMP.<br>– Botulinum A toxin light chain (BoNT/A) to cleave SNAP-25.<br>• Microinjection of competitive peptides:<br>– VAMP2 N-terminal peptide (residues 1–26).<br>– Syntaxin 4 peptide (residues 106–122).<br>– Control peptide Syntaxin 1c.<br>• Stimulation: Insulin (100 nM)	• Plasma-membrane lawn assay (combined with immunofluorescence): a technique generating plasma membrane sheets attached to glass slides, followed by immunofluorescent staining to quantify GLUT4 translocated to the membrane.<br>• Confocal laser scanning microscopy: to visualize and analyze fluorescence images of plasma membrane lawns.<br>• Flow cytometry: analysis of protein interactions (syntaxin 4, SNAP-25) with GLUT4-containing microsomes.	• Recovery after microinjection: 60–90 minutes prior to insulin stimulation.<br>• Insulin stimulation: 15 minutes.	• Non-injected cells on the same coverslip.<br>• Buffer-microinjected cells.<br>• Cells injected with GST protein or control peptide (syntaxin 1c).<br>• Comparison between basal conditions and insulin-stimulated conditions (+ INS).	• VAMP2 and syntaxin 4 participate in GLUT4 exocytosis.<br>• SNAP-25 does not participate.<br>• Syntaxin 4 functions as the t-SNARE for VAMP2.	VAMP2, syntaxin 4, SNAP-25	Stimulation (of VAMP2 and syntaxin 4)	• VAMP2 and syntaxin 4 stimulate GLUT4 exocytosis via an SNARE-dependent mechanism.<br>• VAMP2 functions as the v-SNARE and syntaxin 4 functions as the t-SNARE.<br>• SNAP-25, although capable of forming complexes with VAMP2 and syntaxin 4, does not stimulate GLUT4 exocytosi	Docking, Fusion										Unclear risk	Unclear risk	Low risk	Unclear risk	Unclear risk	Unclear risk	
A.262	10.1128/MCB.19.10.6765	Takeshi Imamura, 1999	USA	Evaluation of the role of the alpha-q G subunit (Gαq) of heterotrimeral G protein in the insulin signaling pathway leading to GLUT4 translocation and identification of the molecular mechanism involved in PI3-kinase.	3T3-L1 adipocytes	Inhibition of endogenous function: Microinjection of anti-Gaq/11 antibodies or RGS2 protein (a GAP protein that inactivates Gaq).<br>Overexpression: Use vector adenovirus to express wild Gaq (WT-Gaq) or constitutively active mutant Q209L-Gaq.<br>Signal Suppression: Use of Wortmannin (PI3K inhibitor), anti-p110 alpha, p110 gamma, PKC-λ, and Akt antibody microinjections.<br>Stimulant: Insulin	Immunostaining & Immunofluorescence microscopy: Evaluation of GLUT4 translocation and membrane ruffling.<br>2-Deoxyglucose uptake assay: Measures the uptake of glucose into cells.<br>PI3-kinase assay: Measuring PI3-kinase enzyme activity in immunoprecipitates of p110 alpha, p110 gamma<br>Western blotting: Determination of protein expression and degree of phosphorylation (of Gaq/11, MAPK, Akt, p70S6K).	GLUT4 translocation: After 20 minutes of insulin stimulation (for micro-injection experiments).<br>2-Deoxyglucose uptake: After 60 hours of adenovirus infection and 1 hour of insulin stimulation.<br>Tyrosine phosphorylation: Measured at 0, 0.5, 1, 2, 5, 10 minutes after insulin is added.	Sheep IgG injection group (Control IgG) or hollow vector virus infection (Mock control/lacZ).<br>The control group did not receive insulin (Basal state).<br>Comparison between Wild Gaq (WT) and Mutant Gaq (Q209L).	1. Gαq is involved in the transport of GLUT4 outside the cell membrane.<br>2. Frequent Gαq activation mutations increase GLUT4 transport 3. Gbetagamma subunits are not involved in the transmission process 4. Gαq is the upstream component of PI3K, in direct contrast to p110α as a subunit of Pi3K.	Gαq (subunit of G heterotrimeric protein)	Stimulation	Gαq stimulates GLUT4 exhaustion by interacting with the p110α subunit of PI3K and activates PI3K and remains in the insulin signaling chain	SIgnaling										Unclear risk	Unclear risk	Unclear risk	Unclear risk	Low risk	Unclear risk	
A.264	10.1074/jbc.271.43.26561	Kazuhiro Kishi, 1996	Japan	Determine which GTP-binding protein is responsible for GPT gamma-stimulated GLUT4 translocation, and examine whether receptors paired with Gq-type G proteins can trigger GLUT4 translocation in an insulin-independent pathway	Fat cells differentiated from the 3T3-L1 lineage (3T3-L1 adipocytes) and Chinese hamster ovarian cells (CHO cells). Specifically, transgenic cell lines expressing c-MYC-tagged GLUT4 (GLUT4myc) and target receptors (PAFR, α1-AR, EP3, EP4).	Stimulation: Use of ligands such as PAF (Platelet-activating factor), Norepinephrine, Prostaglandin E2.<br>Inhibition/Modulation: Pretreatment with Wortmannin (PI3K inhibition), IAP (Islet-activating protein - Gi inhibition), PDBu (to minimize Protein Kinase C), BAPTA-AM (intracellular Calcium catalyst), and beta GDP.	Cell Surface Anti-c-MYC Antibody Binding Assay: Quantitative measurement of the translocation of GLUT4myc to the cell membrane with anti-c-MYC antibodies.<br>2-Deoxyglucose Uptake Measurement: Measures the function of transporting sugars into cells.<br>[Ca2+]i Measurements: Measure intracellular calcium levels with Fura2-AM.<br>cAMP Assay: Measures cAMP concentrations.	Measure GLUT4 translocation and glucose uptake after ligand stimulation for about 10 minutes to 60 minutes (usually 10 minutes for dose experiments).<br>Pre-inhibitory treatments: PDBu and IAP for 24 hours; Wortmannin and BAPTA-AM for 20 minutes.	The control group was not stimulated (Buffer alone/Basal).<br>Parent cells do not express receptors compared to receptor-expressing cells.<br>Insulin stimulation group (Positive control).<br>The inhibitor-treated group compared to the non-processing group.	Platelet activating factor (PAF) receptor (PAFR) is thought to transmit signaling via IAP(Gi)-sensitive and non-IAP(Gq)-sensitive heterotrime-binding GTP-binding proteins.The GLUT4myc translocation pathway is stimulated by PAFs independent of PI3-kinases including p110 gamma. The insensitive pathway to IAP is observed in both cell lines thought to be mediated by Gq, the phosphoinositide-specific phospholipase C β activator (PLC beta) for the hydrolysis of phosphatidylinositol 4,5-bisphosphate (PIP2). The degradation products, inositol 1,4,5-trisphosphate (IP3) and 1,2-diacylglycerol, activate a Ca2+ channel and protein kinase C, respectively. a certain level of [Ca2+]i is required for PAF-stimulated GLUT4myc translocation.<br>- norepinephrine can transport endogenous GLUT4 of 3T3-L1 fat cells by G q binding	PAF, Norepinephrine, Gqi	Stimulation	PAF activates 2 types of heteromer G proteins: Gi (IAP sensitive) and Gq (non-IAP sensitive). When Gq is activated, they activate PLCs to hydrolyze PIP2 into IP3 and 1,2-diacylglycerol, thereby activating PKC and Ca2+ channels (substances thought to be involved in GLUT4 extinguishing) – Norepinephrine also stimulates GLUT4 exhaustion via Gq activation but does not appear to be caused by PIP2 degradatio	SIgnaling										Unclear risk	Unclear risk	Low risk	Unclear risk	Unclear risk	Low risk	
A.267	10.1016/j.cmet.2006.12.006	Irfan J. Lodhi, 2007	USA	To clarify the mechanism of intracellular retention of GLUT4 in the absence of insulin, by studying the function of the Gapex-5 protein (a protein that interacts with CIP4) and its target protein Rab31 during GLUT4 transport.	Fat cells differentiate from 3T3-L1 (3T3-L1 adipocytes) and Cos-1 cells (used for protein interaction experiments).	Overexpression: Gapex-5 (WT and mutants), Rab31 (WT, Q64L - continuous activation, S19N - dominant negative), CIP4.<br>Knockdown (KD): Uses siRNAs to reduce the expression of Rab31 and Gapex-5.<br>Stimulant: Insulin (100 nM or doses lower than 0.1-10 nM).	Yeast two-hybrid screen: To identify proteins that interact with CIP4 (find Gapex-5).<br>GST pull-down assay & Co-immunoprecipitation: Identification of protein-protein interactions (Gapex-5 with CIP4, Gapex-5 with Rab31/Rab5).<br>Confocal Immunofluorescence Microscopy: Observation of intracellular position and translocation of GLUT4, Gapex-5, CIP4.<br>2-Deoxyglucose uptake assay: Measures the function of glucose transport.<br>Rab activation assay (Pull-down with EEA1): Measures the activation (GTP-bound) level of the Rab31.	Insulin Stimulation: 30 minutes.<br>Rab31 Activity Measurement: After 0, 5, 10, 20 minutes of insulin stimulation.<br>After gene/siRNA transfer: 24 hours to 72 hours.	The control group transferred a hollow vector (Vector) or random siRNA (Scrambled control/SC).<br>Basal group (Basal - non-insulin) compared to the insulin-stimulating group.<br>Comparison between mutants (WT, Q64L, S19N, ΔGAP, ΔHB-VPS9).	1. CIP4's SH3 domain interacts specifically with Gapex-5's PXXP motif.<br>2. Gapex-5 permanently interacts with CIP4 and further CIP4 recruits Gapex-5 to the plasma membrane in response to insulin, through TC10 activation 3. Although the VPS9 domain (that of Gapex-5) interacts with Rab5 and Rab31 is GDP-tied; the affinity is markedly stronger for Rab31. Gapex-5 is a GEF for Rab31 and is necessary to maintain its activated state in these cells4. Gapex-5 overexpression inhibited basal plasma membrane localization and was decreased by insulin stimulation 5. The intracellular localization of Gapex-5 is critical for its ability to block Glut4 translocation and furthermore, the insulin-induced plasma membrane localization of Gapex-5 reduces the inhibitory effect of Gapex-5 on Glut4 translocation.<br>6. overexpression of wild-type Rab31 or permanent activity has prevented Glut4 translocation. The reduction of Rab31 in these cells significantly increased the Glut4 translocation stimulated by 1 nM of insulin7. insulin inactivates Rab31 but does not pass PI3K	Rab31, Gapex-5	Inhibition	In the absence of insulin stimulation, Gapex-5 binds to CIP4, inhibits GLUT4 exfoliates, and activates Rab31 (GEF activity) whereby Rab31 also inhibits GLUT4 exfoliates (Rab31 is not inhibited by the PI3K pathway) - There is an insulin signal that activates TC10, thereby transferring CIP4/Gapex-5 to the cell membrane causing inactivation of Gapex-5's GLUT4 proximal inhibition	signaling										Unclear risk	Unclear risk	Low risk	Unclear risk	Unclear risk	Low risk	
A.268	10.1042/BJ20090624	Yu Chen, 2009	China	Investigate the role of two guanine nucleotide dissociation inhibitors (GDI-1 and GDI-2) in insulin-stimulated GLUT4 translocation, and determine the specificity of their interaction with Rab10.	Fat cells differentiate from 3T3-L1 adipocytes and human embryonic kidney cells (HEK-293 cells).	Overexpression: Transgenic expression of fluorescent GDI (GDI-1, GDI-2) proteins (EGFP/mKO), Rab10, Rab4A.<br>Stimulus: Insulin (100 nM).<br>Chemical Agent: Brefeldin A (BFA) to disrupt trans-Golgi (TGN) network function.	Total Internal Reflection Microscopy (TIRFM): Measures the translocation of GLUT4-EGFP to the cell membrane.<br>Acceptor photobleaching FRET (in vivo FRET): Measures the direct interaction and affinity between GDI and Rab in living cells.<br>Immunoprecipitation & Western blotting: Determination of protein-protein interactions.<br>Confocal microscopy: Observation of the simultaneous positioning (colocalization) of proteins.	Insulin stimulation: Time-course monitoring for 30 minutes.<br>After gene transfer: 36 hours (HEK-293) or 2 days (3T3-L1).<br>BFA treatment: 20 minutes before insulin stimulation.	The control group did not treat BFA compared to the BFA treatment group.<br>The GDI-1 expression group compared to the GDI-2 expression group.<br>Hollow vector expression group (EGFP Vector) or control fluorescent protein (mKO-EGFP).<br>Comparison between Basal and Insulin Stimulation.	1. TGN (trans-golgi network) can be the membrane for insulin-stimulated GLUT4 translocation, where Rab10 can initiate the formation of GSV (GLUT4 storage vesicle) and GDIs can bring Rab10 back into recycling.<br>2. GDI-1 actually possesses a higher affinity than GDI-2 for Rab10.<br>3. Overexpression of either GDI protein partially inhibited, but significant, insulin-stimulated GLUT4 translocation	GDI-1, GDI-2, Rab10	Inhibition	GDI-1 has a high affinity for Rab10, a protein that stimulates GSV formation in TGN, and at normal concentrations, they help carry Rab10-GDP from the target membrane to TGN, continuing GLUT-4 exfoliation	Translocation										Unclear risk	Unclear risk	Low risk	Unclear risk	Unclear risk	Unclear risk	
A.269	10.1111/tra.12760	Shifeng Wang, 2020	China	Provides direct genetic evidence of the inhibitory role of tomosyn in insulin-stimulated GLUT4 exhaustion and elucidates the molecular mechanism of this inhibition.	Mouse adipocytes and preadipocytes.	Gene Knockout (DKO): Use CRISPR-Cas9 to delete both tomosyn-coding genes (Tomosyn-1 and Tomosyn-2).<br>Stimulant: Insulin (different concentrations, e.g. 100 nM).<br>Rescue: Re-expression of the human TOMOSYN-1 gene in DKO cells.<br>In vitro experiment: Addition of recombinant tomosyn, NSF, α-SNAP to the liposome fusion system.	Flow cytometry: Measures the transfer of GLUT4 (using the HA-GLUT4-GFP reporter) to the cell surface and quantifies lipid droplets (Nile red staining).<br>Confocal microscopy: Observation of cell imaging and GLUT4 distribution.<br>Immunoblotting (Western blot): Protein expression test (tomosyn, Akt, PPAR gamma, etc.).<br>FRET-based lipid mixing assay: Measures the membrane fusion of liposomes in vitro.<br>Liposome co-flotation assay: Determines the binding of proteins to liposomes.	GLUT4 translocation: After 10, 20, 30 minutes of insulin stimulation.<br>Liposome fusion: Continuous measurement for 60 minutes.	Wild-type (WT) cell group compared to Tomosyn-1/2 Double Knockout (DKO) group.<br>The DKO group was rescued by the TOMOSYN-1 gene.<br>The liposome group with tomosyn compared to the group without tomosyn.<br>The liposome group with NSF/α-SNAP supplementation compared to the group without.	Tomosyn does not regulate insulin signaling.<br>Significantly higher surface levels of the GLUT4 reporter were observed in Tomosyn-1/2 DKO adipocytes under both unstimulated and insulin-stimulated conditions.<br>Tomosyn binds to GLUT4 exocytic t-SNAREs (syntaxin-4 and SNAP-23) and blocks their pairing with the v-SNARE (VAMP2, VAMP3 and VAMP8) fusion reaction at an intermediate stage. It can also arrest membrane fusion when SNARE complexes are partially assembled; failing to dissociate VAMP2 from t-SNAREs once the SNARE complex is fully assembled.<br>NSF and α-SNAP directly dissociate tomosyn from GLUT4 exocytic t-SNAREs, correlating with their ability to relieve the inhibitory activity of tomosyn in fusion reactions.	Tymosin	Inhibition	Tymosin is not involved in signaling.<br>- Tymosin attaches to the t-SNARE complex (syntaxin-4 and SNAP-23) and prevents v-SNARE (VAMP2, VAMP3 and VAMP8) from binding. This process is not possible only when t-SNARE and v-SNARE are fully linked.<br>- NSF and α-SNAP directly separate tomosyn from GLUT4 progenitor t-SNAREs, correlating with their ability to reduce the inhibitory activity of tomosyn in their fusion response	Docking										Unclear risk	Unclear risk	Low risk	Unclear risk	Unclear risk	Low risk	
A.270	10.1016/j.bbrc.2015.10.111	Inmaculada Lopez-Sanchez, 2015	USA	To investigate how GIV proteins bind upstream insulin signals to downstream membrane transport events, specifically to determine the interaction of GIV with GLUT4 storage vesicles (GSVs) and the Exo-70 subunit of the exocyst complex to regulate GLUT4 excellence.	Line cells: HeLa cells, L6 rat skeletal myotubes, and Cos7 cells.	Stimulus: Insulin (100 nM)<br>Gene suppression: Depletion of GIV with siRNA (GIV-depletion).<br>Mutant gene expression: Expression of GIV variants: GIV-FA (GEF functional deficiency) and GIV-SD (simulation of a state inhibited by PKC).	Confocal fluorescence microscopy: Monitor the translocation of GLUT4 (HA-GLUT4-GFP) and Exo-70 to the plasma membrane.<br>Immunoblotting (Western Blot): Determine protein expression levels and knockdown efficiency.<br>Proximity Ligation Assay (PLA): Detect in-situ interaction between GIV and GLUT4 or Exo-70.<br>GST-pulldown assay and Co-immunoprecipitation: Determine direct protein-protein interactions (e.g., GIV with Exo-70).<br>Immunoisolation: Isolate GSV vesicles to examine the presence of GIV.	Insulin stimulation for 5 minutes, 10 minutes, or 15 minutes depending on the experiment.	Control cells (Scramble siRNA) vs GIV-depleted cells (GIV siRNA).<br>Cells express wild GIV (WT) compared to mutant bodies (GIV-FA, GIV-SD).<br>Starved vs. Insulin Stimulation.	1. InsR is functionally bound to Gαi/s proteins that are sensitive to pertussis toxin. Gi activation increases insulin sensitivity, enhances tyrosine phosphorylation of both InsR and IRS1, and activates the efficient translocation of GLUT4 to PM. GIV binds directly and permanently to the N-terminus of IRS1 and is a true bona fide enhancer of the PI3K-Akt pathway downstream of many RTKs, including InsR. GIV binds directly to InsR, IRS1, and PI3K, thereby strengthening the signaling chain of IRS1 and InsR.<br>2. The GEF function of GIV is necessary for the efficient release of GLUT4 and glucose uptake and the inhibition of its phosphorylation by PKC degrades both. 3. GIV can bind to GSV in a ligand-dependent manner.<br>4. GIV can bind to the exocyst complex, specifically the Exo-70 subunit, and that interaction is permanent (i.e., independent of ligand stimulation).<br>5. Permanent GIV-Exo70 complexes remain in the cytoplasm in starved cells and bind to GSV at PM only after insulin stimulation.	GIV	Stimulation	GIV is the GEF for G protein Gαi1/2/3 (which can be activated by insulin and stimulates tyrosine phosphorylation in both IRS1 and InsR). GIV binds directly to InsR and is the substrate of InsR, and also attaches to the N-end of IRS1 and binds directly to PI3K. Thereby strengthening the signaling chain of IRS1 and InsR. PKCtheta suppresses GIV → Activates insulin resistance.<br>- GIV usually binds to Exo-70 (non-insulin-dependent), when stimulated, GIV binds to GSV and carries Exo-70 to the cell membrane and binds them to GCV.Q8	Signaling										Unclear risk	Unclear risk	Low risk	Unclear risk	Unclear risk	Unclear risk	

A.271	10.1074/jbc.M106042200	G Bandyopadhyay, 2001	USA	Evaluate the impact of glucose on signaling mechanisms involved in glucose transporter (GLUT4) translocation and glucose transport, in particular identifying the novel signaling pathway that glucose uses to activate insulin-independent PKC-ζ/λ	Rat adipocytes and Rat skeletal muscles: soleus muscles and extensor digitorum longus (EDL).	Stimulation: Glucose (D-glucose, 20-25 mM), Insulin (10 nM), exogenous PLD.<br><br>Inhibition: Use of chemical inhibitors (Wortmannin, Genistein, PD98059, Dantrolene, GF109203X, n-butanol) and expression of dominant-negative (DN) or kinase-inactive (KI) protein forms of PKC-ζ, PYK2, GRB2, SOS, RAS, ERK.	2-[3H]Deoxyglucose uptake assay: Measurement of glucose transport.<br><br>Translocation assay (Epitope-tagged GLUT4/GLUT1): Measures the amount of GLUT4 or GLUT1 translocated to the cell membrane with antibodies.<br><br>Western blotting: Determination of the translocation of endogenous GLUT4/GLUT1 (separation of plasma membranes and microsomes).<br><br>Immunoprecipitation & Kinase activity assay: Measure the enzyme activity of PKC-ζ/λ, ERK, PKB, PLD.<br><br>Phospholipase D (PLD) assay: Measures PLD activity through the formation of [3H] phosphatidylethanol.	GLUT translocation: After 10-30 minutes of glucose/insulin stimulation.<br><br>Glucose transport: After 15-30 minutes of stimulation.<br><br>Kinase activation: After 5-20 minutes of stimulation.	The control group did not treat (Control/Basal) or treated with Mannitol (to control osmotic pressure).<br><br>Insulin stimulation group (Positive control).<br><br>The inhibitor-processing group or dominant-negative expression compared to the non-processed/empty vector group.	glucose-stimulated, but non-insulin-stimulated, PYK2-dependent GLUT4 translocation and PYK2-mediated activation of the ERK pathway, glucose-stimulated 2-[H]deoxyglucose (and therefore glucose) uptake by glucose, but not insulin-stimulated, was inhibited by PYK2 inhibitors, dantrolene, and MEK1 inhibitors, PD98059. Phosphhatidic acid (PA) has been reported to directly activate PKC-ζ by a mechanism that, like the one used by PIP3, is probably heterostereotypical in nature, but, unlike PIP3, is not accompanied by an increase in self-phosphorylation of PKC-ζ. More notably, the PA generated by the action of a PC-specific PLD, has also been shown to activate PKC-ζ. PLD, which is activated by glucose, can be active on the distal side compared to ERK, but on the proximal side compared to PKC-ζ/λ, during glucose action. As in fat cells, glucose activates PKC-ζ/λ in skeletal muscle via the PYK2/ERK pathway, and furthermore, it seems plausible to propose that this PYK2/ERK-dependent activation of PKC-ζ/λ contributes significantly to the increase in glucose uptake observed during skeletal muscle incubation at supraphysiological glucose concentrations	Glucose	Stimulation	Glucose actually increases GLUT4 translocation/glucose transport in these tissues, apparently by activating PKC-ζ/λ through a mechanism independent of PI 3-kinase but, interestingly, depends on the PYK2/GRB2/SOS/RAS/RAF/MEK1/ERK/PLD/ phosphatidic acid (PA) generating pathway, a known direct activator of PKC-ζ/λ (PYK2-activated glucose by dantrolene-sensitive Ca2+). This process is most clearly manifested in type 2 diabetes.	Signaling	Unclear risk	Unclear risk	Low risk	Unclear risk	Unclear risk	Low risk
A.273	10.1016/j.bmcl.2012.06.101	G. Naresh, 2012	India	To investigate the effects of 4-hydroxypipecolic acid (4-HPA) on glucose uptake and GLUT4 translocation in L6 mouse skeletal muscle cells, in order to explore the hypoglycemic mechanism of this compound.	L6-line mouse skeletal musculoskeletal cells express myc-tagged GLUT4 (L6-GLUT4myc myotubes).	Main compound: 4-hydroxypipecolic acid (4-HPA) in different concentrations (2.5, 5, 10, 25, 50 μM).<br><br>Processing Time: Incubate for 16 hours.<br><br>Positive control: Insulin (100 nM).<br><br>Inhibitors: Wortmannin (PI3K inhibitors).	2-Deoxyglucose (2-DG) uptake assay: Measures the uptake of glucose into cells.<br><br>Antibody-coupled colorimetric assay (Anti-myc antibody): Measures the translocation of GLUT4 to the cell surface (based on extracellular myc tags).<br><br>Semi-quantitative RT-PCR: Gene expression (mRNA) analysis of GLUT4, IRS-1, PKB (Akt).	Glucose absorption: After 16 hours of incubation with 4-HPA (and 20 minutes of insulin stimulation if available).<br><br>GLUT4 displacement: After 3, 6, 16, 24 hours (optimal time 16 hours).<br><br>Gene expression: After 16 hours of incubation with 4-HPA.	The control group did not process (Control/Basal).<br><br>Insulin processing group (Positive Control).<br><br>The treatment group combines 4-HPA and Insulin.<br><br>The group processed Wortmannin alone or in combination with 4-HPA/Insulin.	4-HPA, an unusual amino acid, has structural features that are close to the insulin secretion stimulants and increase insulin sensitivity of 4-hydroxyisoleucine and fagomine (pseudosugar). Both inputs (insulin and 4-HPA) can activate the same signal sequence (via PI3K) to stimulate GLUT4 translocation in the L6-GLUT4myc muscle ducts.	4-HPA	Stimulation	4-HPA stimulates the insulin-like Pi3K-mediated signaling pathway--> Potential for the treatment of type 2 diabetes mellitus	Signaling	Unclear risk	Unclear risk	Low risk	Unclear risk	Unclear risk	Low risk
A.276	10.1101/2023.06.06.543897	Anuttoma Ray, 2023	USA	Investigate the molecular mechanism that regulates the trafficking of GLUT4 in unexcitated human skeletal muscle cells and explore how AMPK activation alters this process through proximal proteome mapping.	The human skeletal muscle cell line (SKM) is differentiated into myotubes.	AMPK Stimulation: Use the activator AMPK PF739 (3 μM) or AICAR.<br><br>Insulin Stimulation: Use Insulin (10 nM).<br><br>Gene interference: CRISPR/Cas9 is used to generate TBC1D4 and Rab10 knockout cell lines; using siRNAs to knock down Rab8a and Rab10.<br><br>Proximity Labeling: Using the HA-GLUT4-APEX2 structure with biotin-phenol and H2O2 substrates.<br><br>Chemical Inhibition: Use Dynasore to inhibit dynamin.	Quantitative Immunofluorescence & Flow Cytometry: Measurement of GLUT4 displacement (signal-to-membrane ratio/total) using the HA-GLUT4-GFP reporter.<br><br>Exocytosis/Endocytosis kinetic assays: Measure the propulsion and intracellular kinetics of GLUT4 with anti-HA antibodies.<br><br>APEX2-Mass Spectrometry (Proteomics): Determination of GLUT4 (proximal proteome) neighboring proteins.<br><br>Western Blot: Evaluation of phosphorylation levels of ACC, AKT, TBC1D4 and determination of knockout/knockdown efficiency.	PF739 stimulation: 60 minutes (to achieve a new steady state).<br><br>Insulin Stimulation: 30 minutes.<br><br>APEX2 reaction: 1 minute of H2O2 treatment after 60 minutes of biotin-phenol incubation.<br><br>Cell differentiation: Day 3 after differentiation.	Basal/Unstimulated: Cells are not treated with stimulants.<br><br>Control group: Parental cells (Wild-type) versus Knockout cells (TBC1D4 KO, Rab10 KO).<br><br>APEX2 control: Sample without biotin-phenol (Negative control).	the effect of AMPK on GLUT4 propulsion is primarily to accelerate the migration of GLUT4 that is in equilibrium with the PM of unstimulated cells. TBC1D4 is phosphorylated by both AKT and AMPK. Rab10 is required for the activation of GLUT4 exertion of AMPK in human SKM cells. The redistribution of GLUT4 to PM is primarily achieved by changing the transport between these compartments rather than mobilizing a static GLUT4 reservoir in the non-stimulated cells.	AMPK	Stimulation	AMPK enhances GLUT 4 exocytes by activating TBC1D4 (AS160), which in turn activates Rab10--> Activates GLUT4 transport from various intracellular compartments (trans-Golgi, endosome,...)	Signaling	Unclear risk	Unclear risk	Low risk	Unclear risk	Unclear risk	Low risk
A.278	10.1091/mbc.e09-08-0751	Wenyong Xiong, 2010	USA	To investigate the role of FQQI (F5) and TELE-based (L489/490) motif sequences in the classification of GLUT4 into specialized vesicles and to determine how insulin regulates the accumulation of these vesicles under the plasma membrane as well as their membrane fusion.	3T3-L1 adipocytes	Gene mutations: Expression of mutations of GLUT4 (F5A - FQQI motif mutation; LA - TELE motif mutation; F5A/LA - double mutation) fluorescent mount (GFP/mCherry).<br><br>Stimulus: Insulin (170 nM).<br><br>Induced insulin resistance: Chronic treatment with insulin (10 nM for 18 hours) or TNF-α (6 ng/ml for 18 hours).	Total Internal Reflection Fluorescence Microscopy (TIRFM): Measures GLUT4 density in the evanescence field (just below the cell membrane, about 100-200nm).<br><br>Epifluorescence Microscopy: Measures the total amount of GLUT4 in the cell.<br><br>Flow cytometry: Measures the amount of GLUT4 on the cell surface (using anti-HA or anti-myc antibodies).<br><br>Uptake assay (Transferrin): Monitors the infusion of Transferrin.	Insulin stimulation: Measured in a steady state after 20-30 minutes or time-course monitoring (every 2 seconds in the TIRFM experiment).<br><br>Insulin Resistance: After 18 hours of chronic treatment.	Comparison between GLUT4 types: Wild-type (WT) versus mutants (F5A, LA, F5A/LA) and Transferrin Receptor (TR).<br><br>Condition comparison: Basal state vs. insulin stimulation.<br><br>Comparison of pathological models: Normal cells (Control) compared to insulin-resistant cells (Chronic insulin/TNF-α).	Here, we use an internal total reflector fluorescence microscopy to show that two transport motifs of GLUT4, an FQQI motif and a TELE-based motif, target GLUT4 to specialized vesicles that accumulate adjacent to the plasma membrane of unstimulated fat cells. Mutations of these motifs redistributed GLUT4 to recycled vesicles containing transferrin adjacent to the plasma membrane, and the degree of redistribution correlated with an increase in GLUT4 mutations in the plasma membrane of basal fat cells. These results establish that GLUT4 defaults to recycled endosomes when transport to specialized vesicles is interrupted, supporting the hypothesis that specialized vesicles originate from an endosome compartment. Insulin stimulates both the accumulation of GLUT4 in the evanescent field and this small portion of GLUT4 inserts into the plasma membrane. Inhibition of Akt activity prevents the insertion of GLUT4 vesicles into the plasma membrane but does not prevent their accumulation below the membrane.	GLUT4	Stimulation	GLUT4 has 2 transport motifs (FQQI and TELE-based motifs) that help transport GLUT4 into the intracellular vesicles near the plasma membrane (when insulin stimulation is absent), while preventing the incorporation of GSV into the membrane.<br>- Stimulating insulin also increases the accumulation of GLUT4 under the plasma membrane (the downstream PI3K pathway but different from Akt) and simultaneously stimulates membrane harmonization.	Tethering + Fusion	Unclear risk	Unclear risk	Low risk	Unclear risk	Unclear risk	Low risk
A.279	10.1016/j.bbrc.2015.03.094	Wenying Ren, 2015	USA	Characterize the palmitoylation of GLUT4 (specifically the altered amino acid site) and understand the role of this process in membrane trafficking of GLUT4 to maintain glucose homeostasis.	Fat cells differentiate from the 3T3-L1 lineage (3T3-L1 adipocytes), CHO-IR cells (human insulin receptor-expressing hamster ovarian cells), and HEK293 cells.	Gene mutation: Cysteine is replaced by serine at positions 223 (C223S), 361 (C361S), and 363 (C363S) on GLUT4 to prevent palmitoylation.<br><br>Stimulant: Insulin (10 nM).<br><br>Chemical Marking: Use 17-ODYA (artificial fatty acids) and Hydroxylamine (HyA) treatment to determine the thioester bond.	Thiopropyl captivation (TPC) assay: Detects palmitoylated proteins.<br><br>17-ODYA metabolic labeling & Click chemistry: Confirm the binding of fatty acids to proteins.<br><br>Subcellular fractionation & Western blotting: Separation of cell segments (plasma membrane - PM, low-density microsomes - LDM) and quantification of proteins.<br><br>Confocal microscopy/TIRF: Observation of intracellular distribution and translocation of fluorescent GLUT4 (GFP/mCherry).<br><br>Immunocytochemistry (non-permeabilized): Measures the amount of GLUT4 on the cell surface with anti-HA antibodies.	Insulin Stimulation: 30 minutes.<br><br>Metabolic labeling: Performed after protein expression (usually 24-48 hours after transgene transfer).	Wild GLUT4 expression group (WT) compared to mutant group (C223S, C361S, C363S).<br><br>Basal group vs. insulin stimulation group.<br><br>Hydroxylamine treatment group (HyA - palmitoyl removal) versus no treatment.	We found that palmitoylated Glut4 at Cys223 and palmitoylation of Glut4 at Cys223 is necessary for insulin-dependent Glut4 membrane translocation, since the replacement of Cys223 with a serine residue in Glut4 (C223S Glut4) reduces the responsiveness of Glut4 to insulin in membrane translocation in both adipocytes and CHO-IR cells. We examined the subcellular positioning of C223S Glut4 and observed that it was absent from the tubular-vesicle structure, where insulin-responsive Glut4 vesicles are present. Together, our studies discover a novel mechanism by which Glut4 palmitoylation regulates the classification of Glut4 to insulin-responsive vesicles, thereby regulating insulin-dependent Glut4 membrane translocation	GLUT4	Stimulation	The palmitoylation of Cys223 of GLUT4 allows GLUT4 to enter the tubular-vesicle structure under insulin stimulation, thereby transferring GLUT4 to the membrane	Signaling	Unclear risk	Unclear risk	Low risk	Unclear risk	Unclear risk	Low risk
A.280	10.1091/mbc.E02-02-0071	Anja Zeigerer, 2002	USA	To investigate the relationship between TR-containing endosomes and the specialized GLUT4 transport pathway in fat cells, specifically to identify the intracellular pools of GLUT4 and the role of rab11 in GLUT4 storage and translocation.	Fat cells differentiate from 3T3-L1 adipocytes and 3T3-L1 fibroblasts.	Insulin Stimulation: Insulin (170 nM).<br><br>Functional inhibition: Compartment ablation with HRP-transferrin (HRP-Tf) in combination with DAB/H2O2 is used.<br><br>Gene interference: Expression of rab11BP(334-504) protein fragment to inhibit rab11 function.	Quantitative Single-cell Fluorescence Microscopy: Measurement of the GLUT4 surface-to-total ratio using the HA-GLUT4-GFP reporter.<br><br>HRP-mediated Fluorescence Quenching Assay: Determines the degree of colocalization of GLUT4 with the Transferrin Receptor (TR) or vpTR by measuring the degree of fluorescence extinguishing.<br><br>Recycling Assay: Measures the recycling rate of TR and vpTR.	Insulin Stimulation: 15-30 minutes (to achieve a steady state).<br><br>Quenching reaction: Incubate for 4 hours with antibodies and HRP-Tf, then react for 30 minutes on ice.<br><br>Recycling assay: Measures after 60 minutes (with TR) or 120 minutes (with vpTR).	Basal State: No insulin.<br><br>Transporter comparison: GLUT4 vs. Transferrin Receptor (TR – represents the common endosome) and vpTR (represents the insulin pathway).<br><br>Cell comparison: Adipocytes vs. fibroblasts.<br><br>Intervention comparison: rab11BP expression group compared to control.	In this study on intact fat cells, we demonstrate using a compartment-specific fluorescence suppression assay, that GLUT4 is evenly distributed between two intracellular reservoirs: endosomes containing transferrin receptors and a specialized compartment that excludes transferrin receptors. These GLUT4 tanks have dynamic communication with each other and with the cell surface. The insulin-induced redistribution of GLUT4 to the surface requires the mobilization of both reservoirs. These data establish the role of the common endosomal system in the specialized, insulin-regulated transport of GLUT4. The transport through the general endosomal system is regulated by rab11. Here, we show that rab11 is necessary for GLUT4 transport from the endosome to the specialized compartment and for insulin-stimulated translocation to the cell surface, emphasizing the importance of the common endosomal pathway in the specialized transport of GLUT4. Based on these findings, we propose a two-step model for GLUT4 transport, in which the general endosome recycling compartment plays a specialized role in insulin-regulated GLUT4 transport. This compartment-based model provides a framework for understanding insulin-regulated transport at the molecular level.	Rab11	Stimulation	In fat cells, GLUT4 is evenly distributed between two intracellular reservoirs: endosomes containing transferrin receptors and a specialized compartment that excludes transferrin receptors (formed from TR endosomes during cell differentiation).<br>Insulin stimulates the mobilization of both reservoirs to transport GLUT4 to the cell surface (transported by Rab11) by 2-step transport: From the TR endosome to the specialized compartment, from the specialized compartment to the cell membrane.E16	Translocation	Unclear risk	Unclear risk	Low risk	Unclear risk	Unclear risk	Unclear risk
A.283	10.1042/0264-6021:3590639	Romel Somwar, 2001	Canada	Explore the hypothesis that insulin activates GLUT4 via the p38 MAPK-dependent pathway by analyzing the timing and kinetics of GLUT4 translocation stimulation, glucose uptake, and activation of p38 MAPK isoforms.	L6 series myotubes express stable GLUT4 tagged Myc in the extracellular (L6-GLUT4myc).	Stimulus: Insulin (100 nM).<br><br>Inhibition: The use of p38 MAPK inhibitors is SB202190 and SB203580.<br><br>Control: The analogue does not work SB202474.	2-deoxyglucose/3-O-methylglucose uptake: Measures glucose transport.<br><br>Antibody-coupled optical assay (Cell surface GLUT4myc): Measures the amount of GLUT4 translocated to the cell membrane.<br><br>Immunoblotting: Determination of the degree of phosphorylation of p38 MAPK, Akt, CREB.<br><br>In vitro kinase assay: Measures the enzymatic activity of p38 α, p38 β and Akt.	Time-course: 2, 5, 7, 10, 15, 20 minutes after insulin stimulation.<br><br>Absorption: For 30 seconds or 2 minutes.<br><br>Inhibitor Treatment: Incubate for 20 minutes.	Basal group vs. insulin stimulation group.<br><br>The inhibitor treatment group (SB202190, SB203580) compared to the control group (Control/SB202474).<br><br>Compare temperatures of 37 °C and 22 °C.	In L6 muscular tubules expressing stable GLUT4 with an extracellular Myc epitope, we find that GLUT4 translocation (t 1/2=2.5 min) precedes stimulation of 2-deoxyglucose uptake (t 1/2=6 min). The separation between glucose uptake and GLUT4 translocation becomes clearer when these two parameters are measured at 22 °C.<br>Pre-incubation with p38 MAPK inhibitors as SB202190 and SB203580 reduced the insulin-stimulated transport of either 2-deoxyglucose or 3-O-methylglucose by approximately 40–60%.<br>Pretreatment with SB203580 reduced the apparent Vmax of insulin-mediated 2-deoxyglucose and 3-O-methylglucose without any significant change in the apparent Km for both hexose sugars. Insulin induced a dose-dependent increase in phosphorylation of p38 MAPK (EC 50 = 15 nM) and time (t 1/2 = 3 minutes), peaking at 10 minutes (2.3 ±0.3 times). In vitro kinase assay of immune precipitates from insulin-stimulated muscle ducts showed activation of p38 alpha (2.6±0.3 times) and p38 beta (2.0±0.3 times) of MAPK.<br>These results suggest that GLUT4 activation follows GLUT4 translocation, and that both mechanisms contribute to the overall stimulation of glucose uptake by insulin. Furthermore, GLUT4 activation can occur via a SB203580-sensitive pathway, potentially involving p38 MAPK.	p38 MAPK	Stimulation	Insulin stimulates GLUT4 transport to the cell membrane via the whole p38 MAPK pathway	Signaling	Unclear risk	Unclear risk	Low risk	Unclear risk	Unclear risk	Low risk
A.285	10.1042/bj3270637	Paru B. Oatey, 1997	UK	Develop a system to monitor the movement of GLUT4 in living fat cells using the green fluorescent protein (GFP) to determine the specific step that insulin takes in the vesicle transport pathway (e.g., vesicle migration or membrane fusion).	3T3-L1 adipocytes	Gene expression: Microinjection of plasmids expresses GFP-GLUT4 into the cell nucleus.<br><br>Stimulant: Insulin (200 nM or 166 nM).<br><br>Chemical agents: Nocodazole (destroys microtubules), Cytochalasin D (destroys actin fibers).	Time-lapse confocal microscopy: Real-time monitoring of the kinetics and migration of GFP-GLUT4-containing vesicles in living cells.<br><br>Cell-surface biotinylation: Sulfo-NHS-biotin and Texas Red-avidin are used to differentiate GLUT4 on the plasma membrane from GLUT4 located just below the membrane.<br><br>Immunofluorescence: Staining tubulin and actin to observe the cell skeleton.	Observation: 24 hours after plasmid microinjection.<br><br>Insulin Stimulation: 1 hour.<br><br>Time-lapse: Take a photo every 30 seconds for a 15-minute period.	The group of cells in the basal/non-stimulated state.<br><br>The cell group is stimulated by insulin.<br><br>The group treated the drug that destroyed the skeleton cell (Nocodazole/Cytochalasin D).	Time-lapse confocal microscopy has demonstrated that the majority of vesicles containing GFP-GLUT4 in the basal state are relatively static, as if bound (or attached) to an intracellular structure. However, a proportion of these vesicles are highly dynamic and move quickly in a linear leap pattern between the inside of the cell and the plasma membrane. Insulin does not significantly increase the speed at which this vesicle moves. These data suggest that stimulation of GLUT4 translocation by insulin is likely related to the release of vesicles from intracellular retention and/or their fusion with the plasma membrane, rather than stimulation of vesicular transport on the cytoskeleton.	Insulin	Stimulation	stimulation of GLUT4 translocation of insulin is likely related to the release of vesicles from intracellular retention or their fusion with the plasma membrane, rather than stimulation of vesicular transport on the cytoskeleton (accelerated transport,..)	Signaling	Unclear risk	Unclear risk	Low risk	Unclear risk	Unclear risk	Unclear risk
A.286	10.1074/jbc.M804282200	Varinder K. Randhawa, 2008	Canada	Analysis of specific steps (recruitment/binding vs. membrane fusion) during GLUT4 transport is regulated by two branches of insulin signaling, Akt/AS160 and Rac/actin, in muscle cells.	Myoblasts and myotubes of the L6 line stably express Myc-tagged GLUT4 (L6-GLUT4myc).	Stimulus: Insulin (100 nM).<br><br>Gene interference: Use siRNA to knock down AS160, Rab8A, Rab10, Rab14, Myosin Va. Expression of mutations of Rac1 (constitutionally active - L61, or inactivated - N17).<br><br>Chemical agent: Latrunculin B (LatB) to destroy actin.	TIRF microscopy (Total Internal Reflection Fluorescence): Measures the density of GLUT4 vesicles in the evanescence field (about 100-200nm under the membrane), representing recruitment/docking.<br><br>Colorimetric assay (Cell surface GLUT4myc detection): Measures the true insertion of GLUT4 into the plasma membrane (membrane fusion) through the detection of extracellular Myc epitopes.<br><br>Confocal/Epifluorescence microscopy: Observation of the overall distribution of GLUT4 and actin.<br><br>Immunoblotting: Determines the knockdown effect and the degree of protein phosphorylation.	Insulin Stimulation: 10 minutes (for TIRF and surface experiments).<br><br>Live imaging: Record for 3 minutes (TIRF experiment).	Basal state vs. insulin stimulation.<br><br>Control group (Scramble siRNA/Vector) vs. intervention group (siRNA knockdown/Rac mutants/LatB).	In contrast, the breakdown of actin by Rac dominant negative or Latrunculin B eliminated insulin-induced superficial and submembrane GLUT4myc buildup. The expression of non-phosphorylated AS160 (AS160-4P) canceled the membrane insertion of GLUT4myc and partially reduced its cortical build-up, an effect amplified by the selective knockdown of Rab8A. We propose that insulin-induced actin dynamics are involved in the retention of GLUT4myc vesicles below the membrane, while phosphorylation of AS160 is essential for GLUT4myc-membrane vesicle binding/fusion and also contributes to the availability of GLUT4myc in the cerebral cortex via Rab8A.	Rac, AS160, Rab8A	Stimulation	Rab8A stimulates the accumulation of GLUT4 submembrane<br><br>AS160 inhibits the accumulation in the cell cortex of GLUT4 as well as docking/fusion - Rab8A stimulates the accumulation of GLUT4 in the cell cortex	Docking/ Fusion	Unclear risk	Unclear risk	Low risk	Unclear risk	Unclear risk	Low risk
A.287	10.1038/sj.emboj.7600297	Isao Usui, 2004	USA	Hypothesis test that GRK2 (G protein-coupled receptor kinase 2) acts as an endogenous inhibitor of the insulin signaling pathway leading to glucose transport, through the interaction and inhibition of the Gq subunit q/11 of the G protein.	3T3-L1 fat cells (3T3-L1 adipocytes).	GRK2 (Loss of function) inhibition: Microinjection of anti-GRK2 antibodies, siRNA gene transfer against GRK2, or expression of GRK2-K220R mutations (lack of kinase activity) and GRK2-D110A/K220R (lack of RGS activity).<br><br>Gain of function: Adenovirus infection expresses wild GRK2 (WT).<br><br>Stimulant: Insulin (different concentrations, e.g. 0.6 ng/ml or 100 ng/ml).	GLUT4 translocation assay (Immunofluorescence): Measures the translocation of GLUT4 to the cell membrane.<br><br>2-Deoxyglucose uptake assay: Measures glucose transport.<br><br>Immunoprecipitation & Western blotting: Determination of protein interactions (GRK2-Gq), q/11 and the degree of phosphorylation (Gq, IRS-1, Akt, MAPK).<br><br>Confocal microscopy: Observation of the localization of GRK2 and GLUT4.	Insulin stimulation: 2 minutes, 10 minutes, 20 minutes (usually measure GLUT4 displacement at 20-30 minutes).<br><br>After Adenovirus infection: 48 hours.	The control IgG injection group compared to the anti-GRK2 antibody injection group.<br><br>Hollow vector virus (LacZ) infection group compared to GRK2-WT infection group.<br><br>Basal group vs. insulin stimulation group.	In this study, we demonstrate that microinjection of anti-GRK2 antibodies or siRNAs against GRK2 increased the translocation of insulin-stimulated glucose transporter 4 (GLUT4) in response to insulin, while adenovirus-mediated overexpression of wild-type GRK2 or kinase deficiency inhibited GLUT4 translocation as well as uptake 2-deoxyglucose is stimulated by insulin. Importantly, a GRK2 mutation that lacks the RGS domain has no effect. Taken together, these results indicate that through its RGS domain, endogenous GRK2 acts as a negative regulator of insulin-stimulated glucose transport by interfering with Gαq/11 signaling to GLUT4 translocation. Furthermore, GRK2 inhibitors may lead to enhanced insulin sensitivity.	GRK2	Inhibition	GRK2 inhibits GLUT4 transport by inhibiting the Gαq/11 pathway by its RGS (regulator of G protein signaling) domain)	Signaling	Unclear risk	Unclear risk	Low risk	Unclear risk	Unclear risk	Low risk
A.288	10.1016/j.devcel.2006.07.008	Kazuho Kawase, 2006	Japan, USA	Elucidate the exact role of GTP hydrolysis by TC10 in cell exodus processes, specifically testing the hypothesis that GTP hydrolysis by TC10 triggers vesicle fusion using newly developed FRET probes.	Elucidate the exact role of GTP hydrolysis by TC10 in cell exodus processes, specifically testing the hypothesis that GTP hydrolysis by TC10 triggers vesicle fusion using newly developed FRET probes.	Stimulation: Epidermal Growth Factor (EGF) (for HeLa cells); Membrane depolarization with high-concentration KCl (for PC12 cells).<br><br>Mutant gene expression: Expression of mutations of TC10 (active constitution - Q75L, or inactivated/dominant-negative - T31N), Rac1-N17 (dominant-negative).<br><br>RNA interference (RNAi): Knockdown TC10 and p190RhoGAP-A by siRNA/shRNA.<br><br>Chemical agent: Diphenyleneiodonium chloride (DPI) to inhibit ROS production.	FRET imaging (Fluorescence Resonance Energy Transfer): Use the Raichu-TC10 probe to monitor the GTPase activity of TC10 (GTP/GDP ratio) in living cells in space and time.<br><br>TIRF microscopy (Total Internal Reflection Fluorescence): Observe single fusion events and measure the change in TC10 activity at the time of membrane fusion.<br><br>NPY secretion assay: Measures the amount of Neuropeptide Y (NPY) secreted from PC12 cells.<br><br>Time-lapse imaging: Observation of lamellipodia enlargement and cell migration.	Fusion events: Continuous observation (time-lapse) with high time resolution (every 0.2 - 1 second) around the time of fusion.<br><br>TC10 activity: Measured before and after EGF stimulation (0-20 minutes).<br><br>NPY secretion: After 20 minutes of depolarization stimulation.	Control group (Control/Vector/Scramble siRNA) versus intervention group (TC10 mutants/RNAi/DPI).<br><br>Comparison between the active probe (Raichu-TC10) and the negative control probe (Raichu-TC10-NC).<br><br>Comparison of intracellular sites: Plasma Membrane (PM) vs Vesicle vs Perinucleus (PNC).	Using a newly developed probe based on fluorescence resonance energy transfer (FRET), we found that TC10 binds to hydrolyzed GTP during insulin inducing GLUT4 release and subsequent endocytosis. The expression of GTP hydrolysis defective TC10 mutations has caused the accumulation of prongolic vesicles anchored at the plasma membrane. In contrast, a dominant-negative mutation of TC10 prevented pouch anchoring. Similarly, inhibition of interfering RNA (RNAi) of TC10 prevented the release of the norepinephrine transporter (NET). These observations suggest that TC10 activity is necessary for anchoring in the proliferation of GLUT4 and NET, and that hydrolysis of GTP by TC10 is necessary for vesicle fusion.	TC10	Inhibition	TC10-GTP promotes tethering of GLUT4 vesicles on the membrane but suppresses fusion. The GTP hydrolysis of the TC10 will trigger bag fusion.	Tethering/ Fusion	Unclear risk	Unclear risk	Low risk	Unclear risk	Unclear risk	Unclear risk

A.289	10.1074/jbc.273.21.13289	Jeffrey S. Elmendorf, 1998	USA	Investigate the mechanism by which GTP gamma (a non-hydrolyzed GTP analogue) stimulates the translocation of GLUT4 by comparing its signaling properties with insulin, particularly the role of PI 3-kinase, Akt kinase, and tyrosine kinase.	Differentiated 3T3-L1 adipocytes are perforated by Streptolysin-O (SL-O permeabilized).	Stimulant: Guanosine 5'-O-(3-thiotriphosphate) (GTPgammaS, 100 μM) or Insulin (10 nM).<br><br>Inhibition: Wortmannin (inhibits PI3K), Genistein and Herbimycin A (inhibits tyrosine kinase), GDPbetaS (GTP antagonist), antiphosphotyrosine monoclonal antibody (PY20) injection	Plasma membrane sheet assay & Immunoblotting: Isolation of plasma membrane array and immunoblotting to measure the amount of GLUT4 translocation.<br><br>Immunofluorescence microscopy: Direct observation of GLUT4 on plasma membrane plaques.<br><br>Akt Kinase Activity Assay: Measuring Akt enzyme activity in vitro (using Histone H2B substrate).<br><br>Immunoprecipitation & Western blotting: Determination of the degree of tyrosine phosphorylation of proteins (PYK2, IRS1, Insulin Receptor, etc.).	Stimulation: For 15 minutes (for GLUT4 translocation) or 5 minutes (for kinase activation).<br><br>Inhibitor treatment: Incubate for 15-30 minutes.	The control group (Control/Vehicle) compared to the stimulation group (Insulin/GTPgammaS).<br><br>The group with inhibitors (Wortmannin, Genistein, Herbimycin A, GDPbetaS) compared to the group without.	Treatment of permeabilized adipocytes with Guanosine 5'-O-(3-thiotriphosphate) (GTPγS) results in GLUT4 translocation similar to that produced by insulin. However, although the selective phosphatidylinositol 3-kinase inhibitor wortmannin completely blocked insulin-stimulated GLUT4 translocation, it had no effect on GTPγS-stimulated GLUT4 translocation. In addition, insulin is an effective stimulant, while GTPγS is a very weak activator of serine/threonine kinase Akt downstream. Consistent with the Akt-independent mechanism, guanosine 5'-O-2-(thio)diphosphate inhibited insulin-stimulated GLUT4 translocation without any effect on Akt kinase. Surprisingly, two differentially functional tyrosine kinase inhibitors, genistein and herbimycin A, as well as the microinjection of phosphotyrosine-specific monoclonal antibodies, both inhibit GLUT4 translocation induced by both GTPγS and insulin-stimulated. Phosphotyrosine immunosuppression and specific immunoprecipitation have demonstrated that GTPγS does not induce tyrosine phosphorylation of the insulin receptor or insulin-1 receptor substrate. In contrast to insulin, proteins in the range of 120-130 kDa and 55-75 kDa were phosphorylated to tyrosine after stimulation with GTPγS. Some of these proteins have been identified and include protein-tyrosine kinase 2 (also known as CAKB, RAFTK, and CADTK), pp125 focal adhesion tyrosine kinase, pp130 Crk-associated substrate, paxillin, and Cbl. These data demonstrate that GTPγS-induced GLUT4 translocation uses a novel tyrosine kinase pathway independent of both phosphatidylinositol 3-kinase and Akt kinase.	GTPγS	Stimulation	GTPγS stimulates GLUT4 transport along the insulin-independent pathway as well as PI3K and Akt. However, they are still regulated through the tyrosine kinase receptor	Signaling										Unclear risk	Unclear risk	Low risk	Unclear risk	Unclear risk	Unclear risk
A.290	10.1128/MCB.21.15.5262-5275.2001	Avirup Bose, 2001	USA	Determine the role of G alpha11 protein (activated by Endothelin-1) and its downstream factor ARF6 in regulating F-actin skeleton restructuring and GLUT4 transfer to the plasma membrane in 3T3-L1 adipocytes.	Fat cells differentiate from the 3T3-L1 line (3T3-L1 adipocytes).	Stimulation: Use Insulin (100 nM) or Endothelin-1 (100 nM).<br><br>Gene expression: Transfection of plasmids expressed Activated G alpha11 (Galpha11-QL), wild ARF6 (WT), activated ARF6 (Q67L), dominant-negative ARF6 (T27N).<br><br>Inhibitory agents: Clostridium difficile toxin B (inhibits Rho GTPases), C3 exoenzyme.	Quantitative Immunofluorescence (Translocation assay): Quantify the ratio of GLUT4 on the cell surface to the total amount (using the HA-GLUT4 reporter and anti-HA antibodies).<br><br>Confocal Microscopy: Fluorescence imaging of actin (phalloidin staining), GLUT4 and G/ARF proteins.<br><br>Subcellular Fractionation & Immunoblotting: Cell segmentation (membrane/cytoplasmic) separation and immunosuppression to determine the location and level of protein expression.	Stimulation: 15 minutes (for most translocation experiments).<br><br>Transfection: Measured 48 hours after transfection.	Basal state vs. Insulin/Endothelin-1 stimulation.<br><br>The hollow vector expression group (pcDNA3) compared to the group expressing mutant proteins (Galpha11-QL, ARF6-Q67L, ARF6-T27N).	The action of insulin recruits the intracellular glucose transporter GLUT4 to the plasma membrane of 3T3-L1 fat cells mimicked by endothelin 1, which transmits signals through trimeric proteins Gαq or Gα11. In contrast to insulin, activation of GLUT4 translocation of Gα11 occurs without activation of phosphatidylinositol (PI)3-kinase or Akt/protein kinase B. However, endothelin 1 stimulates ADP-ribosylation of ADP ribosylation factor 6 (ARF6), and the expression of ARF6-negative dominant mutations, ARF6(T27N), potently inhibits GLUT4 translocation in response to endothelin 1 or G expression<br>α11. Furthermore, Clostridium difficile B toxin inhibitors for Rho family GTPases or the expression of RhoA, Rac1, or Cdc42-negative dominant mutations inhibited Gα11-induced GLUT4 translocation. Expression of ARF6, Rac1, or permanently active RhoA did not mimic the effect of Gα11 in stimulating GLUT4 recruitment to the plasma membrane. However, the expression of the permanent active forms of ARF6 and RhoA together produced a strong synergistic response to GLUT4 translocation. Activation G<br>α11 results in pronounced actin polymerization at the pericellular periphery, which is dependent on the functional activity of ARF6 and RhoA. This actin polymerization is necessary for GLUT4 translocation stimulated by endothelin 1 or G<br>α11, because the actin fiber-destroying agent latrunculin B inhibited these reactions. Taken together, these data demonstrate that the signal path through the mediated G<br>α11 results in GLUT4 translocation in fat cells that act through an ARF6 and RhoA-based mechanism resulting in local actin restructuring that is important for this membrane transport.	Gα11, ARF6 and RhoA	Stimulation	Endothelin 1 (ET-1) activates the Gα11 and Gαq proteins, thereby Gα11 activates GLU4 not through PI3K or Akt but through ARF6 and Rac1 as actin restructure	Signaling										Unclear risk	Unclear risk	Low risk	Unclear risk	Unclear risk	Unclear risk
A.291	10.1074/jbc.M105894200	Xiaosong Song, 2001	USA	The role of the Gαi2 protein in the regulation of glucose transport is investigated, focusing on binding to insulin signaling and the translocation of GLUT4.	Transgenic mice (iQ205L) express the activated Gαi2(Q205L) mutation in adipose tissue, skeletal muscle and liver; along with wild-type littermates.	Gene Expression: Constitutively active Gα expression i2(Q205L).<br>Stimulants: Insulin (intravenous or in vitro administration), Lysophosphatidic acid (LPA).<br><br>Inhibition: Pertussis toxin (PTX) for inhibition of Gαi2	3-O-methylglucose uptake assay: Measures glucose transport.<br><br>Subcellular fractionation & Immunoblotting: Separation of cell segments (plasma membrane - PM, high/low density microsomes - HDM/LDM) and quantification of GLUT4 by Western blot.<br><br>PI 3-Kinase Activity Assay: Measures the activity of the PI 3-kinase enzyme.<br><br>Akt Activity Assay: Measures the activity and degree of phosphorylation of Akt.	Insulin stimulation: 5 minutes after injection (in vivo) or treatment (in vitro).<br><br>LPA stimulation: 15 minutes.	Wild-type mice vs. transgenic mice (Q205L).<br><br>Basal (Basal) vs. Insulin Stimulation (+Insulin).<br><br>No Processing vs. LPA/PTX Treatment.	Using mice carrying a transgenic gene that directs the expression of a permanently active Gαi2 mutation, deficient form of GTPase (Q205L) in adipose tissue, skeletal muscle, and liver, we demonstrate that Gαi2 regulates the translocation of the insulin-sensitive glucose transporter GLUT4 in skeletal muscle and adipose tissue. The expression of Q205L Gαi2 increases glucose transport and GLUT4 transposition to the plasma membrane in vivo in the absence of insulin stimulation. Adipose cells from Q205L Gαi2 mice expressed glucose transport and GLUT4 transposition to the insulin-stimulated plasma membrane to nearly twice the level of cells from control mice of the same age. The activities of Phosphatidylinositol 3-kinase and Akt are permanently activated in Q205L Gαi2-expressing tissues. Studies on fat cells from wild-type mice show short-term activation of phosphatidylinositol 3-kinase, Akt, and GLUT4 translocation in response to Gαi2 activation by lysophosphatidic acid, a sensitive reaction to pertussis toxin.	Gαi2	Stimulation	Gαi2 stimulates non-insulin-dependent GLUT4 exertion<br><br>Gαi2 expression suppresses defects in insulin signaling and GLUT4 translocation commonly seen in diet-induced obesity.	Signaling	Unclear risk	Low risk	Unclear risk	Unclear risk	Unclear risk	Unclear risk	Unclear risk	Unclear risk	Unclear risk						
A.292	10.1016/j.metabol.2019.154006	Jiahan Wu, 2019	China	Study on the effects of miR-130a-3p derived from hepatic exosomes on the regulation of glucose and lipid metabolism in adipose tissues.	FVB series mice (including WT wild mice, 130KO knockout mice, 130OE overexpress mice) and 3T3-L1 fat cell line.	In vivo: Genetically modified mice (130KO, 130OE); Feeding a high-fat diet (HFD); Intravenous injection of hepatic exosomes from different sources (WT-EXO, KO-EXO, OE-EXO) into 130KO mice.<br><br>In vitro: 3T3-L1 cell transfusion with miR-130a-3p mimics, inhibitors, or treatment with hepatic exosomes.	Glucose/Insulin Tolerance Tests (GTT/ITT): Measures glucose tolerance and insulin sensitivity.<br><br>Western blot: Determination of protein expression (FASN, SREBP, PPAR gamma, PHLPP2, AKT, AS160, GLUT4).<br><br>Glucose Uptake Assay (using 2-NBDG): Measures glucose uptake.<br><br>Oil Red O staining & Triglyceride Assay: Measures lipid accumulation.<br><br>Luciferase reporter assay: Determination of the target of miRNA (PHLPP2).<br><br>Co-immunoprecipitation (Co-IP): Determines protein interactions.<br><br>Immunofluorescence: Observe the translocation of GLUT4.	In vivo: After 12 weeks of dieting; after 28 days of continuous exosome injections (every 48 hours); Monitor exosomes after 12 hours and 24 hours of injection.<br><br>In vitro: Measure glucose absorption after 4 hours of treatment; Oil dyeing after 10 days of differentiation.	WT Mouse vs 130KO vs 130OE.<br><br>Standard Diet (SD) vs. High-Fat Diet (HFD).<br><br>Exosomes from different sources: WT-EXO, KO-EXO, OE-EXO.<br><br>Cell Processing Mimics, Inhibitor vs. Negative Control (NC).	After HFD feeding, the weight of 130KO mice was significantly higher than that of WT mice. Overexpression of miR-130a-3p in 130OE mice and intravenous administration of 130OE-EXO in 130KO mice contributed to a positive correlation with the restoration of insulin resistance. In addition, treatment of 3T3-L1 cells with miR-130a-3p mimics and 130OE-EXO showed a decrease in the production of lipid droplets and an increase in glucose uptake. In contrast, in vitro and in vivo inhibition of miR-130a-3p leads to opposite phenotypic changes. Furthermore, PHLPP2 has been identified as a direct target of miR-130a-3p, and hepatic exosome-derived miR-130a-3p can improve impaired glucose tolerance through inhibition of PHLPP2 to activate the AKT-AS160-GLUT4 signaling pathway in fat cells	miR-130a-3p	Stimulation	hepatic exosome-derived miR-130a-3p may improve impaired glucose tolerance through inhibition of PHLPP2 to activate the AKT-AS160-GLUT4 signaling pathway in fat cells	Signaling	Unclear risk	Unclear risk	Unclear risk	Unclear risk	Unclear risk	Unclear risk	Unclear risk	Unclear risk	Unclear risk	Unclear risk	Unclear risk	Unclear risk	Unclear risk	Unclear risk	Low risk
A.295	10.1016/j.ejmg.2005.04.004	Jonas Burén, 2003	Sweden	Studying the cytological effects of long-term exposure to high insulin and glucose levels on glucose transport and insulin signaling proteins (IRS-1, IRS-2, PKB).	Primary cultured rat adipocytes.	Long-term culture (24 hours): In environments with different glucose concentrations (5, 10, 15, 25 mmol/l) with or without high concentration insulin (10^4μU/ml = 60 nmol/l).<br><br>Acute stimulation: Insulin (concentrations between 25 and 10^4μU/ml) for 45 minutes to measure glucose uptake.	D-[U-^{14}C] glucose uptake assay: Measures the ability of cells to absorb glucose.<br><br>125<br>I-insulin binding assay: Measures the ability of insulin to bind to the receptor on the cell surface.<br><br>Western blotting (Immunoblotting): Quantify the protein expression levels of GLUT4, IRS-1, IRS-2, PKB (Akt), and phosphorylation levels of PKB (Ser473).	Chronic treatment: 24 hours.<br><br>Absorption/binding/protein measurement: After insulin/glucose washing of the culture step (recovery time/measurement after 24 hours).	Low glucose group (5 or 10 mmol/l) compared to high glucose group (15 or 25 mmol/l).<br><br>The group without insulin (Control) compared to the group with high insulin (Insulin-treated).	Results: High glucose (15 and 25 mmol/l) for 24 hours caused a decrease in basal and insulin-stimulated glucose uptake compared to control cells incubated in low glucose (5 or 10 mmol/l). Twenty-four hours of insulin therapy reduced insulin binding by approximately 40%, and shifted the dose-response curve for the acute effect of insulin on glucose uptake to the right by 2 to 3 times. Twenty-four hours of high-glucose and insulin combination treatment resulted in a 50% reduction in insulin maximum response, depletion of 60–70% of insulin receptor substrate proteins (IRS)-1 and -2, and caused a marked (approximately 50%) reduction in sensitivity of acute insulin-stimulated serine-473 protein kinase B (PKB) phosphorylation. Total cell GLUT4 levels are not altered by any conditions.<br><br>Conclusions: These results demonstrate that high glucose and high insulin interact to induce insulin resistance in cultured primary fat cells. We suggest that in type 2 diabetes, the combination of hyperglycemia and hyperinsulinemia (secondary to insulin resistance) exacerbates insulin resistance through depletion of IRS proteins and reduced sensitivity to PKB activation.	Glucose, prolonged high-concentration exposure insulin	Inhibition	Glucose, prolonged high-concentration exposure insulin depletes 60–70% of insulin receptor substrate proteins (IRS)-1 and -2, and causes a marked decrease in sensitivity (about 50%) of acute insulin-stimulated serine-473 protein kinase B (PKB) phosphorylation	Signaling										Unclear risk	Unclear risk	Low risk	Unclear risk	Unclear risk	Low risk
A.297	10.1042/bj3250727	J. Carlos Aledo, 1997	Scotland	Using the skeletal muscle segmentation process to identify and characterize intracellular GLUT4 pools, in order to look for evidence of the existence of separate compartments: one endosome-related and one insulin-sensitive.	Male Sprague-Dawley rats	Intervention: Supramaximal intravenous dose of human insulin, 1.5 units.<br><br>Extraction procedure: Fragmentation of muscle cell membranes using discontinuous sucrose gradients at concentrations of 25%, 30% and 35%.	Subcellular fractionation: Separation of membrane segments based on density to isolate reservoirs.<br><br>Western blot analysis: Quantification of proteins (GLUT4, GLUT1, TfR, Annexin II, vp165...) in segments.<br><br>Immunoisolation: Precipitation of GLUT4-containing vesicles for analysis of the accompanying protein composition.<br><br>Double-labelling immunoelectron microscopy: Observation of the colocation of GLUT4 and vp165 on cellular superstructures.	Sample collection: 30 minutes after insulin or saline injection.	The group treated with insulin compared to the control group (Saline control).<br><br>Comparison between different intracellular membrane segments: F30 Segment (floating on 30% sucrose) and F35 Segment (floating above 35% sucrose layer).	We have shown that after segmentation of coarse muscle membranes on an intermittent sucrose gradient, the majority of the immune response of GLUT4 is present mainly in two sucrose segments (30 and 35%, weight/weight [w/w], sucrose; denoted F30 and F35, respectively) containing intracellular membranes of varying buoyancy density. Here we show that these segments contain 44±6% and 49±7% of the GLUT4 reactivity capacity of the crude membrane, respectively, and can be separated from each other as well as from the plasma membrane and the musculoskeletal network marker proteins (Ca2+-ATPase) and the Golgi apparatus (α-mannosidase II). The F35 fraction is also rich in glucose transport protein GLUT1, the α1 subunit of the Na+/K+-ATPase pump, the transferrin receptor (TfR), and the cellubrevin synapse protein. In contrast, the F30 segment is largely devoid of these marker proteins but is rich in synaptic vesicle protein VAMP-2 (membrane proteins associated with vesicles-2). In vivo insulin treatment resulted in a 2.2 ±0.4-fold increase in GLUT4 content in the plasma membrane, accompanied by a 45±6% reduction in GLUT4 content in the F30 segment. In contrast, the amount of GLUT4 that binds to F35 does not change significantly in response to insulin. Immunosorbent studies using immunomodulatory antibodies specific to the extracellular portion of GLUT4 or TfR indicate that vesicles derived from F30 and F35 represent distinct membrane populations.	Regarding GLUT4 stockpiles	Inhibition	GLUT4 is stored in 2 intracellular compartments. 1 is the endosome, 2 is the insulin sensitivity blocker		Unclear risk	Low risk	Unclear risk	Unclear risk	Unclear risk	Unclear risk	Unclear risk	Unclear risk	Unclear risk						
A.298	10.1042/BJ20041845	Conrad P. Hodgkinson, 2005	UK	Identify proteins that interact with PKC zeta (specifically 80K-H) through bi-hybrid yeast screening and investigate whether the 80K-H protein plays any role in GLUT4 vesicle transport.	Cell lines: CHO cells (Cho-K1, Cho-mycGLUT4), 3T3-L1 fat cells, L6 muscle tubes (L6 myotubes), and COS-1 cells.	Gene expression: Overexpression of 80K-H (full-length, 80K-H 172, 80K-H 248) or PKC zeta structures.<br><br>Stimulus: Insulin (100 nM).<br><br>Screening: Yeast two-hybrid screen.	Yeast two-hybrid system: Identifies protein interactions.<br><br>GST pull-down assay: In vitro interaction validation.<br><br>Co-immunoprecipitation (Co-IP): Determines protein interactions in vivo (endogenous).<br><br>2-Deoxyglucose transport assay: Measuring glucose absorption.<br><br>Membrane fractionation & Western blotting: Measurement of GLUT4 translocation (plasma membrane distribution vs low-density microsomes - LDM).<br><br>In vitro kinase assay: Test the phosphorylation of 80K-H.	Insulin stimulation: 5 minutes (for Co-IP), 20 minutes (for glucose uptake/translocation).<br><br>Glucose Absorption Measurement: For 5 minutes.<br><br>Post-transplantation: 48 hours.	The insulin-free group (Basal) compared to the insulin-free group.<br><br>The control group (Control/GST/Vector) compared to the 80K-H expression group (172 or 248).	Immune precipitation of endogenous PKCζ from CHO cells, 3T3-L1 fat cells, or L6 muscle tubules containing endogenous 80K-H, demonstrate a physiological interaction. The stimulation of insulin strengthens this bond by 3–5 times. The immune precipitators of endogenous 80K-H contain endogenous Munc18c, and the immune precipitates of endogenous Munc18c contain endogenous PKCζ, with insulin increasing these bindings. 80K-H and Munc18c also link directly in vitro. A permanently active form of PKCζ increases the phosphorylation of 80K-H in intact cells. Importantly, knockdown of 80K-H with siRNA (small interfering RNA) inhibited insulin-stimulated GLUT4 translocation in 3T3-L1 fat cells. These results identify 80K-H as a protein that binds to PKCζ and Munc18c, and is a new component of the GLUT4 pouch transport apparatus.	80K-H, PKCζ	Stimulation	PKCζ is activated by PI3K, then phosphorylated by 80K-H, involved in complex interactions with PKCζ and Munc18c, thereby stimulating glucose transport	Signaling/ Fusion										Unclear risk	Unclear risk	Unclear risk	Unclear risk	Unclear risk	Unclear risk
A.299	10.1091/mbc.e09-03-0187	Jamie A. Lopez, 2009	Australia	Determine the role of cortical actin in distal trafficking of GLUT4, specifically the binding and fusion of the vesicle membrane with the plasma membrane under the influence of insulin.	3T3-L1 adipocytes	Stimulation: Use of Insulin (100 nM).<br><br>Actin inhibition: Use Latrunculin B (Lat-B, 10 μM) to disrupt actin polymerization.<br><br>Signal suppression: Use Wortmannin (PI3K inhibitor), Akti 1/2 (Akt inhibitor), and BAPTA-AM (calcium chelator).<br><br>Gene expression: Transfer of GLUT4-eGFP, IRAP-pHluorin, HA-GLUT4, and IRAP-TfR reporter structures	TIRFM (Total Internal Reflection Fluorescence Microscopy): High-frequency intra-total reflection microscopy for monitoring the kinetics of single vesicles in living cells.<br>Confocal Laser-scanning Microscopy: Observation of the skeletal structure of actin and tubulin in 3D space.<br><br>HA-GLUT4 Translocation Assay: Quantify the level of GLUT4 translocation to the cell surface.<br><br>Western Blot: Check for signal transmission events (phosphorylation Akt, TBC1D4...).<br><br>TIRF Explorer Algorithm: An automated algorithm for detecting and quantifying fusion events.	Insulin stimulation: Measure after 20-30 minutes.<br><br>Live Imaging: Continuous recording at 10 frames per second.<br><br>Pretreatment of inhibitors: Between 15 and 60 minutes in prison	Basal radiance compared to the insulin-stimulated state.<br><br>The control group (DMSO) compared to the actin-breaking group (Lat-B).<br><br>The group receiving a signal inhibitor (Wortmannin, Akti) compared to the group without	Using high-frequency internal total reflectance fluorescence microscopy (TIRFM) imaging, we show that insulin increases actin polymerization near PM, and that disruption of this process inhibits GLUT4 proliferation. Using TIRFM in combination with probes that can distinguish between vesicle transport and fusion, we found that faulty actin restructuring was accompanied by the accumulation of GLUT4 vesicles near normal PM under insulin regulation, but the eventual prosthetic fusion step was impaired. These data clearly decompose the many steps of the final stages of GLUT4 transport, demonstrating the important role of actin in the final stage of this process	Actin Shell	Stimulation	Envelope actin plays an important role in the final steps of GLUT4 exfoliation and acts near the cell membrane	Fusion										Unclear risk	Unclear risk	Low risk	Unclear risk	Unclear risk	Low risk

A.303	10.1074/jbc.M112.394148	Paul Langlais, 2012	USA	Determining the role of CLASP2 protein in the effects of insulin on GLUT4 transport and glucose uptake.	3T3-L1 adipocytes	Stimulation: Use of insulin at different concentrations (0.2–20 nM or 100 nM).<br><br>Gene suppression: Use siRNA to knock down CLASP2 expression.<br><br>Inhibition of signaling pathways: Use of Wortmannin (inhibition of PI3K) and PD98059 (inhibition of MAPK)	Mass spectrometry: HPLC-ESI-MS/MSn to identify proteins and novel phosphorylation sites.<br>Immunoprecipitation & Western blotting: Determine phosphorylation levels and protein expression.<br>Confocal & DIC Microscopy: Observe the colocalization of CLASP2, GLUT4, and actin.<br>Fluorescence Resonance Energy Transfer (FRET): Acceptor photobleaching technique to measure direct interaction between CLASP2 and GLUT4.<br>Glucose Uptake Assay: Measure the uptake of radiolabeled 3-O-methyl-D-glucose.	Insulin Stimulation: Measured after 15 minutes or 30 minutes.<br><br>After siRNA processing: 72 hours after gene transfer.<br><br>Glucose Absorption: Measured within 10 seconds.	Basal state group compared to insulin stimulation group.<br><br>The control group (Negative control siRNA) compared to the CLASP2 knockdown group (CLASP2 siRNA).<br><br>Inhibitor processing group (Wortmannin, PD98059) versus no treatment.	Using mass spectrometry-based protein identification in combination with phosphoantibody immunoprecipitation in L6 muscle ducts, we detected a 4.8-fold increase of CLASP2 in antiphosphoserine immune precipitates with insulin stimulation. Western blotting of CLASP2 immune precipitates with phosphoantibodies confirmed the finding that CLASP2 undergoes insulin-stimulated phosphorylation, and several new phosphorylation sites have been identified. Confocal imaging of the L6 muscle tubules reveals that CLASP2 cologues with GLUT4 at the plasma membrane in insulin-mediated cortical actin restructuring regions. CLASP2 is responsible for guiding the distal end of the microtubules to the cell shell, and it has been shown that GLUT4 moves along the microtubule rails. In support of the hypothesis that CLASP2 plays a role in GLUT4 transport at the pericellular pericell, the reduction of CLASP2 by siRNA in the L6 muscle tubule interfered with the localization of insulin-stimulated GLUT4 to the plasma membrane. Furthermore, siRNA-mediated reduction of CLASP2 in 3T3-L1 fat cells inhibited insulin-stimulated glucose transport. Therefore, we propose a novel model for CLASP2 in insulin activity, in which CLASP2 guides the delivery of GLUT4 to cell cortex landing regions that are important for insulin activity	CLASP2	Stimulation	CLASP 2 has the function of guiding the distal end of the microtubules to the cell shell, creating a track for GLUT4 to be transported to the cell shell, from which the cell exit	Translocation		Unclear risk	Unclear risk	Low risk	Unclear risk	Unclear risk	Unclear risk
A.304	10.1074/jbc.M800838200	Xiao Mei Song, 2008	USA	Identification of specific amino acid residues in the C-terminal cytoplasmic tail of the Glut4 protein plays an essential role for the process of translocation to the plasma membrane under the action of insulin.	3T3-L1 adipocytes	Gene mutations: Construct mutated Glut4 structures (tagged HA, eGFP, or DsRed) at potential motifs at the C-terminus, especially insulin-sensitive motifs (IRM: LXXLXPDEXD).<br><br>Stimulation: Use of insulin (670 nM or 1 μM).<br><br>Infection: Adenovirus is used to express mutated proteins in fat cells.	Laser Confocal Immunofluorescence Microscopy: Observation of intracellular distribution and colocalization of Glut4 with organelle markers.<br><br>Subcellular Fractionation: Cell segmentation for plasma membrane (PM), high-density microsomal (HDM), and low-density (LDM) separation.<br><br>Western Blotting: Quantify the level of protein expression.<br><br>Glycosylation Assays: Endo H and N-glycanase are used to examine the transport of proteins through the Golgi complex.<br><br>Labeling of Cell Surface Glut4: Glut4 marking on the surface of living cells with fluorescent anti-HA antibodies.	Insulin Stimulation: Measured after 30 minutes.<br><br>Post-infection: Experiments were conducted 48 hours after Adenovirus infection.	The wild Glut4 (WT) group acts as internal control.<br><br>The Glut4 group mutates at different sites: the FQQI motif at the N-terminus, the dileucine motif at the C-terminus, and the IRM motif.	In this study we define amino acid residues within the C-terminal cyto- plasmic tail of Glut4 that are essential for its insulin-stimulated translocation to the plasma membrane. The residues were iden- tified based on sequence similarity (LXXLXPDEXD) between cytoplasmic domains of Glut4 and the insulin-responsive amin- opeptidase (IRAP). Alteration of this putative targeting motif (IRM, insulin-responsive motif) resulted in the targeting of the bulk of the mutant Glut4 molecules to dispersed membrane ves- icles that lacked detectable levels of wild-type Glut4 in either the basal or insulin-stimulated states and completely abol- ished the insulin-stimulated translocation of the mutant Glut4 to the plasma membrane in 3T3L1 adipocytes. The bulk of the dispersed membrane vesicles containing the IRM mutant did not contain detectable levels of any subcellular marker tested. A fraction of the total IRM mutant was also detected in a wild-type Glut4/Syntaxin 6-containing perinuclear compartment. Inter- estingly, mutation of the IRM sequence did not appreciably alter the subcellular trafficking of IRAP	GLUT4	Stimulation	The IRM fragment at the C end of GLUT4 helps this molecule to be delivered to the plasma membrane under the stimulation of insulin, if this segment is missing, GLUT4 will only be dispersed throughout the cell+P30	Translocation		Unclear risk	Unclear risk	Low risk	Unclear risk	Unclear risk	Low risk
A.306	10.1016/S0014-5793(00)02021-4	Diane L. Cope, 2000	USA	Study the role of specific amino acids (Glu-491, Gln-492, and Glu-493) located immediately after the Leu-489Leu-490 motif at the C-tail of Glut4 in regulating intracellular transport of this protein	Fat cells differentiated from the 3T3-L1 fibroblast line of mice.	Gene mutations: Sequentially replace the amino acid radicals Glu-491, Gln-492, and Glu-493 with Alanine to produce the Glut4 mutant bodies of AQE, EAE, and EQA, respectively.<br><br>Stimulation: Use of 1 μM concentration of Insulin.<br><br>Cavity Destruction (Ablation): Use the Transferrin-HRP complex in combination with DAB and H2O2 to destroy the recycled endosome cavities	Differential centrifugation: Separation of plasma membrane (PM) and low-density microsomal (LDM).<br><br>Immunoblotting: Quantify the expression and distribution of mutated Glut4 proteins (using anti-Glut3 antibodies).<br><br>Plasma membrane lawn assay: Quantify the translocation of Glut4 and IRAP to the plasma membrane.<br><br>Endosomal ablation analysis: Determination of the level of localization of Glut4 in the transferrin receptor-containing endosomal system.	Insulin Stimulation: Performed for 15 minutes (for lawn assay) or 30 minutes (for fractional centrifugation).<br><br>Cell Stage: Use cells from day 8 to day 12 after the start of differentiation.	Mutant Glut4-expressing cell lines (AQE, EAE, EQA) compared to tagged wild Glut4-expressing cell lines (TAG-1).<br><br>Basal state vs. Insulin stimulated state (+Insulin).	In this study, we examined the role of adjacent amino acids (Glu-491, Gln-492, and Glu-493) by sequentially replacing them with Ala. Our results are consistent with the view that Glu-491 and Glu-493 play an important role in the subendosome transport of Glut4, since the replacement of these residues with Ala leads to increased levels of these proteins at the cell surface, decreased insulin-stimulated translocation and increased likelihood of endosomal ablation. These residues, along with other identified sequences in the C-end of Glut4, are likely important targeting factors that regulate the subcellular distribution of Glut4.	GLUT4	Stimulation	The Glu-491, Gln-492 and Glu-493 sequences at the C-end of GLUT4 are effective in transporting GLUT4 to the insulin-stimulated cell membrane	Translocation		Unclear risk	Unclear risk	Low risk	Unclear risk	Unclear risk	Unclear risk
A.308	10.1074/jbc.M111.306621	Denis Balamatsias, 2011	Australia	Identify the effectors of PI3K that promote GLUT4 translocation through the regulation of the actin skeleton in fat cells, specifically examining the role of P-Rex1.	3T3-L1 adipocytes	Insulin Stimulation: Use concentrations ranging from physiological concentration (1-10 nM) to saturation concentration (100 nM).<br>+3<br><br>Gene expression: Transfer of wild P-Rex1 (WT) structures and mutations (P-Rex1(ΔDH) - lack of activation domains Rac1, P-Rex1(Δ4P), P-Rex1(ΔN), P-Rex1(C1583S)).<br><br>Gene suppression: Use siRNA to knock down P-Rex1.<br><br>Regulates Rho GTPase: Expresses variants of Rho, Rac1, and Cdc42 in either constitutional activity (V12) or inhibitory (N17).<br>+1<br><br>Chemical agents: Use of LY294002 (inhibition of PI3K), Cytochalasin D and Latrunculin A (destruction of the actin skeleton)	Indirect immunofluorescence: Texas Red-phalloidin or DNase I is used to observe F-actin/G-actin and membrane ruffles structure.<br><br>GLUT4 translocation test: Use GFP-GLUT4 or Myc-GLUT4-GFP (extracellular Myc epitope) messenger proteins to measure the amount of GLUT4 on the plasma membrane.<br><br>Glucose Absorption Test: Use 2-deoxy-D-glucose (2-DOG) radioactive tracer (14C or H).<br><br>Immunoblotting (Western blot): Determines the level of protein expression and phosphorylation state of Akt.	Insulin Stimulation: Do for 30 minutes.<br>Glucose Absorption Measurement: Measure in the last 10-20 minutes of the stimulation process.<br>After gene intervention: Experiments are conducted after 48-72 hours of plasmid or siRNA transfer.	The control vector group compared to the P-Rex1 expression group (WT or mutation).<br><br>Basal state vs. insulin-stimulated state.<br><br>The control siRNA group (scrambled) compared to the P-Rex1-specific siRNA group.<br><br>The inhibitor-treated group (LY294002, LatA, CytoD) compared to the non-processing group.	Here we demonstrate that the PI3K-dependent Rac exchange factor, P-Rex1, enhances membrane ruffling in 3T3-L1 adipocytes and promotes GLUT4 trafficking to the plasma membrane at submaximal insulin con- centrations. P-Rex1-facilitated GLUT4 trafficking requires a functional actin network and membrane ruffle formation and occurs in a PI3K- and Rac1-dependent manner. In contrast, expression of other Rho GTPases, such as Cdc42 or Rho, did not affect insulin-stimulated P-Rex1-mediated GLUT4 trafficking. P-Rex1 siRNA knockdown or expression of a P-Rex1 dominant negative mutant reduced but did not completely inhibit glucose uptake in response to insulin. Collectively, these studies identify a novel RacGEF in adipocytes as P-Rex1 that, at physiological insulin concentrations, functions as an insulin-dependent regu- lator of the actin cytoskeleton that contributes to GLUT4 traf- ficking to the plasma membrane.	P-Rex1	Stimulation	P-Rex1 is activated by PIP3 from PI3K and they activate Rac1, which in turn restructures the actin skeleton and transports GLUT4 to the cell membrane	Signaling		Unclear risk	Unclear risk	Low risk	Unclear risk	Unclear risk	Low risk
A.315	10.1177/002215549704500806	Daniela Malide, 1997	USA	Use concomitant microscopy to localize in situ of GLUT4 relative to transport vesicle proteins, Golgi apparatus, and endosomals in fat cells to understand the nature of the intracellular cavity that stores GLUT4 in its baseline state	White adipose cells and brown adipose cells isolated from male Sprague-Dawley rats.	Stimulation: Use of 700 nM concentration of Insulin.<br><br>Control state: Basal state (no insulin).<br><br>Marking techniques: Single and dual fluorescence immunosuppression using GLUT4-specific antibodies and cell cavity markers (VAMP2, M6PR/IGFIIR, TGN38, mannosidase II, γ-adaptin, lgp-120, TfR, clathrin)	Laser Scanning Confocal Microscopy (CLSM): Optical tomography and 3D image restructuring to analyze the distribution of GLUT4 across the entire cell volume.<br><br>Glucose uptake measurement method: Using [U−14C]-glucose to confirm transport activity	After 30 minutes of insulin stimulation at 37°C	The group of cells in the basal state compared to the group stimulated by insulin.<br><br>Comparison of the colocation of GLUT4 with various intracellular cavity markers.<br><br>Comparison between white fat cells and brown fat cells.	Three-dimensional analyses of GLUT4 immune staining in basal cells reveal a dot-shaped, intracellular distribution in both the perinucleus and scattered throughout the cytoplasm. VAMP2 binds tightly to GLUT4 in a variety of dotted vesicular structures. A small fraction of GLUT4 overlaps with the TGN38-mannosidase II, γ-adaptin, and mannose-6-phosphate receptors in the perinucleus region, potentially corresponding to late endosomal structures and trans-Golgi networks. GLUT4 is not colocated with transferrin, clathrin, and lgp-120 receptors. After insulin therapy, GLUT4 partially redistributes to the cell surface and is reduced to the perinucleus. However, GLUT4 remains co-localized with TGN38-mannosidase II and γ-adaptin. Thus, the basic compartment from which GLUT4 transposes in response to insulin consists of specialized post-endosome VAMP2-positive vesicles, which are distinct from the constituent recycled endosomes. These results are consistent with a kinetic model in which GLUT4 is isolated through two or more successive intracellular reservoirs.	VAMP2	Stimulation	GLUT4 in the cell is contained in small compartments containing VAMP2, which are not contained in recycled endosomes, and these small compartments by stimulation of insulin activate GLUT4 exfoplasty	Docking and Fusion		Unclear risk	Unclear risk	Low risk	Unclear risk	Unclear risk	Unclear risk
A.321	10.1038/emm.2006.22	Young Ok Choi, 2006	Korea	Examining the Ca2+/calmodulin-dependent light-chain phosphorylation (RLC) of Myosin II by the MLCK enzyme involved in insulin-stimulated GLUT4 translocation in 3T3-F442A fat cells.	Mouse 3T3-F442A adipocytes (3T3-F442A murine adipocytes).	Stimulation: Use of 100 nM concentration of Insulin.<br><br>Inhibitors: ML-7 (MLCK selective inhibitors).<br><br>Blebbistatin and 2,3-butanedione monoxime (BDM) (Myosin II inhibitors).<br><br>BAPTA/AM and dimethyl-BAPTA/AM (intracellular calcium chelate).<br><br>Trifluoperazine (calmodulin antagonist)	2-Deoxyglucose (2-DG) uptake assay: Measuring the rate of glucose uptake using 2−[3H]DG.<br><br>Subcellular fractionation: Separation of plasma membrane (PM) and low-density microsomes (HSP).<br><br>Cell surface biotinylation: Marks surface proteins with biotin to quantify GLUT4 on the membrane.<br><br>RLC phosphorylation determination: Marking metabolism with [32P] orthophosphate and autoradiography to quantify the degree of RLC phosphorylation.<br><br>Immunoblotting (Western blot): Quantification of GLUT4, GLUT1 and RLC	Insulin Stimulation: For 30 minutes.<br><br>Inhibitor Treatment: Incubate for 30 minutes before adding insulin	Basal state vs. insulin stimulation.<br><br>The group without inhibitors (Control) compared to the group with the presence of inhibitors (ML-7, Blebbistatin, BAPTA/AM, etc.).<br>Presence of Extracellular Case 2+ compared to Case-free environment<br>2+EGTA)	Current studies in 3T3-F442A fat cells demonstrate the new finding that insulin significantly increases the RLC phosphorylation of myosin II in a Ca2+-dependent manner. In addition, ML-7, a selective inhibitor of MLCK, as well as myosin II inhibitors, such as blebbistatin and 2,3-butanedione monoxime, suppress insulin-stimulated GLUT4 translocation and subsequent glucose transport. Our studies suggest that MLCK may be a regulatory target of Ca2+/calmodulin and may play an important role in insulin-stimulated glucose transport in fat cells	MLCK, Ca2+/calmodulin	Stimulation	Insulin activates MLCK via Ca2+, which in turn stimulates the transport of GLUT4 to the cell membrane	Signaling		Unclear risk	Unclear risk	Low risk	Unclear risk	Unclear risk	Low risk
A.322	10.1074/jbc.274.40.28637	Matilde Caruso, 1999	Italy	Investigate the mechanisms involved in glucose's self-regulation of its own consumption by focusing on early events that occur in L6 muscle cells following glucose exposure	L6-line skeletal muscle cells expressing the human insulin receptor (L6hIR)	Stimulation by Glucose: Exposure to high glucose levels (25 mM).<br><br>Stimulation by Insulin: Use insulin at a concentration of 100 nM for comparison.<br><br>Gene suppression: Use specific antisense oligonucleotides to inhibit PKCα expression.<br><br>Receptor mutations: Use of L6 cell lines expressing mutated insulin receptors lacking kinase activity (3F1, 3F2)	2-Deoxy-D-glucose (2-DG) uptake assay: Measures the rate at which cells absorb glucose.<br>Cell-surface biotinylation: Marks surface proteins to quantify transporters (GLUT1, GLUT4) on the plasma membrane.<br><br>Western blotting: Quantifying the level of protein expression and distribution in cell segments.<br><br>PKC activity assay: Measuring the enzyme activity of Protein Kinase C in cell extracts or in immunoprecipitation complexes.<br><br>Immunoprecipitation: Isolation of insulin receptors for analysis of binding proteins and phosphorylation states.	Acute stimulation: Measured after 3 minutes of glucose exposure to observe immediate effects.<br>Transport displacement: Measure after 10 minutes.<br>Time-course: From 30 seconds to 60 minutes or 120 minutes.	Basal state (no glucose/insulin) vs. glucose-stimulated state (25 mM) or insulin (100 nM).<br>Wild insulin receptor expressing cell group (L6 hIR) compared to the kinase deficiency mutant receptor expression group (L6 3F).<br>The control group (scrambled oligonucleotide) compared to the PKCα inhibitor group (antisense).<br>Glucose compared to other sugars (xylose, sucrose) or pyruvate.	High glucose levels also activate insulin receptor kinases for endogenous insulin receptor (IRS)-1 and IRS-2 substrates. In contrast, in L6 cells expressing kinase-deficient insulin receptors, exposure to 25 mM glucose has no effect on glucose processing. In L6 cells hIR, the acute effect of glucose on the insulin kinase receptor occurred in parallel with a 2-fold reduction in protein kinase C (PKC) activity in both membranes and PKC co-precipitated with the insulin receptor, along with a 3-fold reduction in receptor Ser/Thr phosphorylation. Western blot analysis of receptor precipitates with isomer-specific PKC antibodies revealed that the decline in glucose-induced membrane-receptor-binding PKC activities was due to dissociation of PKCα and not of PKCβ or −δ. This decrease in PKCα is parallel to a similar-sized increase of PKCα in the cytoplasm. In L6 cells hIR intact, inhibition of PKCα expression using a specific anti-coding oligonucleotide caused a 3-fold increase in IRS phosphorylation by the insulin receptor. This effect is independent of insulin and is accompanied by a 2.5-fold increase in the cells' processing of glucose. Thus, in L6 skeletal muscle cells, glucose acute regulates its own use through the insulin signaling system, independent of insulin. Self-regulation by glucose appears to be related to the dissociation of PKCα from the insulin receptor and its translocation into the cytoplasm Interestingly, the time evolution of the effect of glucose on insulin receptor tyrosine kinase activity (Fig. 6B, IRTK) was inversely correlated with the effect on receptor PKC binding and receptor Ser/Thr phosphorylation	Glucose, PKCα	Stimulation	High glucose levels also activate the IRS-1 and 2 insulin receptors through PKCα dissociation --> Reduce receptor Ser/Thr phosphorylation --> Activation of the insulin-dependent GLUT4 expulsion pathway	Signaling		Unclear risk	Unclear risk	Low risk	Unclear risk	Unclear risk	Unclear risk
A.324	10.2337/diabetes.54.1.41	Matthew D. Bruss, 2005	USA	Characterizes the phosphorylation of Akt substrates (especially AS160) in rat skeletal muscle under the action of insulin (over time and dose) and contractile activity (contractile activity).	Skeletal muscle (epitrochlearis muscle) isolated from male rats of the Wistar lineage.	Insulin Stimulation: Use concentrations from physiological to maximum (0.15 to 120 nmol/l).<br><br>In vitro muscle contraction (Contraction): Electromyography for 5 minutes.<br><br>AMPK stimulation: Use AICAR (2 mmol/l)<br><br>Inhibition of PI 3-kinase: Use wortmannin (100 or 500 nmol/l).	Immunoprecipitation: Isolation of AS160 protein from muscle extract.<br><br>Western Blotting: PAS (phospho-Akt substrate) antibodies are used to detect the phosphorylation motif of Akt on proteins (160 kDa and 250 kDa); measure P-Akt (Ser473) and P-AMPK (Thr172).<br><br>Densitometry: Quantification of the density of protein bandages.	Insulin: Monitored at 1, 2.5, 5, 10, 15, 30, and 60 minutes.<br><br>Muscle contraction: Immediately after 5 minutes of stimulation.<br><br>AICAR: After 40 minutes of incubation.	Basal/No stimulus vs. Insulin/Contraction/AICAR.<br><br>The stimulation group alone compared to the stimulus group with the presence of Wortmannin inhibitors.	Therefore, we evaluated the effects in skeletal muscle of each stimulus on the phosphorylation of proteins, including AS160, on the phospho-Akt motif. The epitrochlearis muscles isolated from mice were incubated with insulin (to monitor time progression and dose response), stimulated to contract the muscles, or incubated with 5-aminoimidazole-4-carboxamide-1-β-D-ribofuranoside (AICAR) and used to evaluate the following factors: serine phosphorylation of Akt (P-Akt), ability to respond immunologically to a phospho-Akt (α-phospho-[Ser/Thr] Akt substrate [PAS] recognizable antibody), and the PAS immunoreactivity of samples immunoprecipitated with anti-AS160. P-Akt peaks after 5 minutes of insulin administration, and PAS immune response then peaks for 250 kDa (10 min) and 160 kDa (15 min) proteins. P-Akt, PAS-160 and PAS-250 increased significantly with 0.6 nmol/l of insulin. Muscle contraction activity leads to increased P-Akt and PAS immune response capacity of the 160 and 250 kDa proteins. The 160-kDa protein is confirmed as AS160 based on the increased PAS immune response capacity in AS160 immune precipitates. Wortmannin inhibits the effects of insulin (120 nmol/l) and muscle contraction on the phosphorylation of AS160. Annealing with AICAR induces increased phosphorylation of protein kinases activated by AMP and AS160 but not Akt. Our working hypothesis is that the phosphorylation of these hypothetical Akt substrates plays an important role in some of the biological effects of insulin and muscle contraction	Akt, AS160	Stimulation	Insulin signaling and muscle contraction increase phosphorylation Akt	Signaling		Unclear risk	Unclear risk	Low risk	Unclear risk	Unclear risk	Low risk
A.326	10.1074/jbc.RA119.010821	Shifeng Wang, 2019	USA and China	Determining the decisive role of the exocyst complex (specifically the Exoc7/Exo70 subunit) in insulin-stimulated GLUT4 exhaustion in adipocytes, using the induced CRISPR gene editing system.	Adipocytes differentiated from mouse preadipocytes and HeLa cells (for whole-genome CRISPR screening).	Inducible KO gene removal: Using the doxycycline-controlled CRISPR/Cas9 system to delete the Exoc7 gene during adipocyte differentiation.<br><br>Insulin Stimulation: Use different insulin concentrations (10 nM, 100 nM).<br><br>Gene Rescue: Human EXOC7 gene expression in cells that have been subjected to mouse gene KO.<br><br>Whole-genome CRISPR screening (GeCKO V2 library).	Flow cytometry: Quantify GLUT4 levels on the cell surface using the HA-GLUT4-GFP reporter.<br><br>Confocal microscopy: Observation of GLUT4 profoliation and the accumulation of lipid droplets.<br><br>Western blotting: Testing the efficacy of gene deletion (Exoc7, Cas9) and signaling proteins (p-AKT, p-AS160, Sort1, PPAR gamma).<br><br>Nile red staining: Evaluation of fat cell differentiation	Gene Induction: Add Dox 24 hours before the start of differentiation.<br><br>Differentiation: Analyzed after 6 days of adding the differentiated mixture.<br><br>Insulin stimulation: 10 minutes (for signaling) and 30 minutes (for translocation/exocellation).<br><br>Kinetics: Measured at timelines within 30 minutes.	Wild cells (WT) vs. Exoc7 KO cells.<br><br>Basal state vs. insulin stimulation.<br><br>The KO group compared to the group rescued by the EXOC7 gene.<br><br>Exoc7 KO has Dox (protein loss) compared to no Dox (normal protein expression).	Here, we first systematically analyzed data from a genome-wide CRISPR screening in HeLa cells that targeted virtually all known genes in the human genome, including 12 exocyst genes. Screening recovered only a subset of exocyst genes, including the exocyst complex component 7 (Exoc7/Exo70). However, other exocyst genes were not isolated in screening, likely due to functional redundancy. Our findings suggest that the selection of an appropriate exocyst gene is critical for genetic studies of exocyst function. Next, we developed an inducible lipid cell genome editing system that allows deletion of the Exoc7 gene in fat cells without disrupting fat cell differentiation. We observed that insulin-stimulated GLUT4 exfoliation was markedly inhibited in Exoc7 KO (gene elimination) fat cells. However, insulin signaling remains intact in these KO cells. These results indicate that exocysts play an important role in insulin-stimulated GLUT4 release in fat cells. We propose that the strategy outlined in this work could be instrumental for genetically analyzing membrane transport pathways in other fat cells.	Exocyst 8-component protein complex	Stimulation	An evolutionarily conservative anchor is the exocyst, a complex of eight anomalous components consisting of Exoc1/Sec3, Exoc2/Sec5, Exoc3/Sec6, Exoc4/Sec8, Exoc5/Sec10, Exoc6/Sec15, Exoc7/Exo70, and Exoc8/Exo84 (Figure 1A). As a member of the multi-subunit anchoring complex family, exocysts interact with lipids, small GTPases (e.g., Rabs), and SNAREs to promote vesicular anchoring in a variety of eukaryotic pathways.<br><br>In this study, it has been demonstrated that the exocyst complex plays an important role in the insulin-stimulated glucose release pathway and is behind the insulin signaling chain	Tethering		Unclear risk	Unclear risk	Low risk	Unclear risk	Unclear risk	Low risk

A.327	10.1074/jbc.M708934200	Jose A. Chavez, 2008	USA	Characterization of Tbc1d1 protein and determination of its role in regulating GLUT4 transport, especially in comparison with AS160 homologous proteins in terms of regulation by Akt and AMPK.	3T3-L1 adipocytes, L6 muscle cells (L6 cells), and tissues from wild rats or AS160 knockout mice.	Overexpression: Transfer of wild Tbc1d1-expressing plasmids (WT) or mutants (S237A, T642A, T596A).<br><br>Gene Suppression (Knockdown): Use specific shRNA to reduce the expression of endogenous Tbc1d1 protein.<br><br>Stimulation: Insulin use (160 nM), activation of AMPK by AICAR (1 mM), and expression of constituent-activated Akt (myrAkt).	Quantitative single-cell fluorescence assay: Measure the amount of GLUT4 on the cell surface using the HA-GLUT4-GFP reporter (Cy3/GFP ratio).<br><br>Immunoprecipitation: Isolation of Tbc1d1 protein for analysis.<br><br>Western blotting: Determines the level of protein expression and phosphorylation (pThr172-AMPK, pThr56-eEF2, PAS).<br><br>Mass spectrometry: Identification of peptides and phosphorylation sites.	Post-transplantation: 24 hours.<br><br>Insulin Stimulation: 30 minutes.<br><br>AICAR treatment: 70 minutes (of which insulin is added in the last 30 minutes).	Post-transplantation: 24 hours.<br><br>Insulin Stimulation: 30 minutes.<br><br>AICAR treatment: 70 minutes (of which insulin is added in the last 30 minutes).	In the present study, we show in 3T3-L1 adipocytes that Tbc1d1 is only 1/20 as abundant as AS160, that knockdown of Tbc1d1 has no effect on insulin-stimulated GLUT4 translocation, and that overexpressed Tbc1d1 also inhibits GLUT4 translocation elicited by activated Akt expres- sion. These results indicate that endogenous Tbc1d1 does not participate in insulin-regulated GLUT4 translocation in adipo- cytes and suggest that the GAP activity of Tbc1d1 is not sup- pressed by Akt phosphorylation. In addition, we discovered that Tbc1d1 is much more highly expressed in skeletal muscle than fat and that the AMP-activated protein kinase (AMPK) activator 5-aminoimidazole-4-carboxamide ribonucleoside partially reversed the inhibition of insulin-stimulated GLUT4 transloca- tion by overexpressed Tbc1d1 in 3T3-L1 adipocytes. 5-Amino- imidazole-4-carboxamide ribonucleoside activation of the kinase AMPK is known to cause GLUT4 translocation in mus- cle. The above findings strongly suggest that Tbc1d1 is a com- ponent in the signal transduction pathway leading to AMPK-stimulated GLUT4 translocation in muscle.	Tbc1d1	Inhibition	Tbc1d1 has an inhibitory effect on GLUT4 exfoliates in the AMPK pathway (AMPK phosphorylation of Tbc1d1 on Ser231 significantly reduces inhibition of proliferation). Although insulin has phosphorylated Tbc1d1 in Thr590, it has no inhibitory effect.	Signaling										Unclear risk	Unclear risk	Low risk	Unclear risk	Unclear risk	Unclear risk
A.328	10.1074/jbc.M002537200	Ko Kotani, 2000	Japan	Investigate whether ASIP (Atypical PKC isotype-specific interacting protein) contributes to the stimulation of glucose uptake by insulin by examining the effects of ASIP overexpression on this process in 3T3-L1 fat cells.	3T3-L1 fat cells	Overexpression: Using adenovirus vectors to overexpress the T7-tagged ASIP protein (AxCAASIP).<br><br>Stimulation: Use of insulin (100 nM), growth hormone (GH - 0.5 μg/ml), or osmotic shock (sorbitol - 300 mM).<br><br>Constitutional stimulation: Use of constituent activation mutations of PKC lambda (AAPD) and Akt (Myr-Akt).	Glucose Uptake Assay: Measure the absorption of 2-deoxy-D-[1,2-3H]glucose.<br><br>Subcellular Fractionation: Separation of low-density microsomal membrane (LDM), plasma membrane (PM) and cytoplasm segments to measure translocation of GLUT4 and PKC lambda using immunoblot.<br><br>Protein Kinase Assays: Measures the enzymatic activity of PI 3-kinase, Akt, and PKC lambda.<br><br>Co-immunoprecipitation (Co-IP): Identifies protein interactions between ASIP and signaling components.	Post-intervention: Experiments were conducted after ~48 hours of viral infection.<br><br>Stimulation: 5 minutes (for kinase activity), 10 minutes (for glucose uptake) and 15 minutes (for GLUT4 translocation).	The non-viral group (control) compared to the ASIP overexpression group.<br><br>Basal state vs. stimulation with Insulin, GH or Sorbitol.<br><br>Impact of ASIP on glucose uptake due to constitutionally activated PKCA compared to constitutionally activated Akt.	The potential role of ASIP in other cellular functions of atypical PKC has been investigated by examining the effects of ASIP overexpression on insulin-induced glucose uptake, which has previously been demonstrated to be PKCλ-mediated, in 3T3-L1 fat cells. When overexpressed in these cells, which contain PKCλ but do not contain PKCζ, ASIP precipitates immunity with endogenous PKCλ but does not precipitate with PKCε or with Akt. The subcellular localization of PKCλ is also altered in ASIP overexpressing cells. ASIP overexpression [illegible] inhibited insulin stimulation of both glucose uptake and the translocation of the glucose transporter GLUT4 to the plasma membrane, but it did not inhibit glucose uptake induced by growth hormone or high osmotic pressure, both of which promote glucose uptake in a PKCλ-independent manner. Furthermore, glucose uptake was stimulated by a permanent active mutation of PKCλ, but not the uptake caused by the active form of Akt, which was inhibited by ASIP. Insulin-induced PKCλ activation, but not phosphoinositide 3-kinase or Akt activation, is also inhibited by ASIP overexpression. These data suggest that ASIP overexpression inhibits insulin-induced glucose uptake by specifically interfering with signals transmitted through PKCλ	ASIP	Inhibition	ASIP inhibits insulin-stimulated GLUT4 activities via PKCλ	Signaling										Unclear risk	Unclear risk	Low risk	Unclear risk	Unclear risk	Unclear risk
A.329	10.1074/jbc.273.31.19740	Yoshikazu Tamori, 1998	Japan	Investigate the role of the Munc18c protein in regulating insulin-stimulated GLUT4 vesicle transport in 3T3-L1 fat cells, through interaction with the t-SNARE protein syntaxin4.	3T3-L1 adipocytes	Excessive gene expression: Use adenovirus vectors to overexpress Munc18c or c-MYC-tagged Munc18b.<br><br>Stimulation: Insulin (100 nM) or osmotic shock with D-sorbitol (600 mM).	2-Deoxy-D-glucose transport assay: Measures the ability of cells to absorb glucose.<br><br>Plasma membrane lawn assay: A technique for measuring the displacement of GLUT4 or GLUT1 to the plasma membrane through fluorescence immunosuppression staining.<br><br>Subcellular fractionation & Immunoprecipitation: Cell segmentation and immune precipitation to check the binding between proteins (syntaxin2, syntaxin4, Munc18c, SNAP-23).<br><br>In vitro binding assay: GST hybrid protein (GST-syntaxin) is used to test the binding affinity of the Munc18 isoforms.	Stimulation: Insulin is taken for 20 minutes; Sorbitol takes 40 minutes.<br><br>Post-viral infection: Experiments were conducted approximately 48 hours after adenovirus infection	Basal state vs. stimulation with Insulin or Sorbitol.<br><br>The group of cells that were not infected with the virus (Control) compared to the group that overexpressed Munc18c or Munc18b.<br><br>Comparison of the effect between Munc18c and Munc18b on the displacement of GLUT4 and GLUT1.	The role of Munc18c, previously identified as an n-Sec1/Munc18 homologous protein in 3T3-L1 fat cells, in insulin-regulated GLUT4 transport has now been studied in 3T3-L1 fat cells. In these cells, Munc18c primarily binds to syntaxin4, although it binds to both syntaxin2 and syntaxin4 to similar degrees in vitro. In addition, SNAP-23, a homologous protein in adipocytes of SNAP-25, binds to both syntaxins 2 and 4 in 3T3-L1 fat cells. Overexpression of Munc18c in 3T3-L1 adipocytes by adenovirus-mediated gene transfer results in an insulin-stimulated glucose transport inhibition in a viral dose-dependent manner (maximum effect ~50%) as well as sorbitol-induced glucose transport inhibition (approximately 35%), which is mediated by a different pathway than the one used by insulin. In contrast, Munc18b, which is also expressed in fat cells but does not bind to syntaxin4, has no effect on glucose transport. Furthermore, overexpression of Munc18c leads to inhibition of the translocation of GLUT4, but not of GLUT1, to the insulin-induced plasma membrane. These results suggest that Munc18c is involved in insulin-dependent GLUT4 transport from the intracellular storage compartment to the plasma membrane in 3T3-L1 fat cells by regulating the formation of a SNARE complex composed of syntaxin4	Munc18c	Inhibition	Munc18c inhibits GLUT4 flobules in the GLUT4 transport pathway to the membrane by regulating the formation of SNARE complexes including syntaxin4	Docking										Unclear risk	Unclear risk	Low risk	Unclear risk	Unclear risk	Unclear risk
A.331	10.1016/j.molmet.2025.102230	Ayushi Mittal, 2025	USA	Explore the role and mechanism of the Unc119b protein in regulating GLUT4 transport and glucose homeostasis, and determine the mechanism of action of the insulin-sensitizing compound C59 through Unc119b.	Mouse: C57BL/6 series (including WT wild mice, Unc119b KO gene-deleting mice, Unc119a KO gene deleting mice) and db/db leptin receptor mutant mice.<br><br>Cells: C2C12 muscle cell lines, HBG4 reporter cell lines (C2C12 expressing HiBiT-GLUT4), and HEK293T cells	In vivo: Induced obesity and insulin resistance with a high-fat diet (HFD) for 10 weeks; Systemic deletion of the UNC119b gene; Oral treatment with C59 compound; AAV virus injection expresses specific Unc119b in skeletal muscle.<br>In vitro: Silence the UNC119b gene with siRNA; Overexpression UNC119b; Insulin stimulation (100 nM) and treatment with C59 (10-30 μM)	IPGTT & ITT: Glucose tolerance and insulin sensitivity test in mice.<br><br>Radioactive glucose uptake assay: Measures the rate at which glucose is absorbed into tissues 3H-2-deoxyglucose.<br><br>HiBiT-GLUT4 translocation assay: Bioluminescence technique for real-time quantification of GLUT4 density on the cell surface.<br><br>PAK1 pulldown & G-LISA: Measures the activity of GTPase Rac1.<br><br>BRET assay: Measuring protein-protein interactions between UNC119b and Rac1 in living cells.<br><br>Crystallography: Crystallography of the UNC119b complex with lipid and drug ligands.	Insulin Stimulation: 5-10 minutes (for Rac1/Akt signaling events) and 20 minutes (for GLUT4 translocation).<br><br>Mouse model: After 10 weeks of HFD ingestion or 2 weeks of treatment with C59.<br><br>Glucose uptake: Measured at 90 minutes after glucose/tracer bolus injection	WT mice vs. Unc119b KO mice (under normal feeding conditions and HFD).<br><br>db/db control mice versus db/db treated with C59 or unc119b gene deletion.<br><br>Cells transfected with scrambled siRNA compared to Unc119b siRNA.<br><br>AAV-GFP expression group (control) versus AAV-UNC119b (intervention).	Methods: Here, we use in vitro systems and insulin-resistant rodent models with genetic manipulations for the expression of UNC119b to explore the role of this protein in regulating glucose homeostasis.<br><br>Results: We demonstrate that Unc119b is an endogenous inhibitor of GLUT4 translocation, contributing to the development of insulin resistance in obese individuals. We show that Unc119b interacts with Rac1 and inhibits its activation by insulin, resulting in a decrease in GLUT4 translocation. Both the prenylated C-terminus of Rac1 and C59 (a small molecule insulin sensitizer) bind to the same site within Unc119b, thus suggesting that C59 enhances GLUT4 translocation by interfering with the effects of Unc119b on Rac1.	Unc119b	Inhibition	Unc119b interacts with Rac1 at the prenylated C-terminus of Rac1 (Cys189) and inhibits Rac1 activity-> inhibits GLUT4 exocytosis; UNC119b is not regulated by Akt-S160<br><br>C59 improves GLUT4 exocytes by competitively inhibiting Rac1 in Unc119b binding Q41	Signaling	Unclear risk	Low risk	Unclear risk	Unclear risk	Unclear risk	Unclear risk	Unclear risk	Unclear risk	Unclear risk	Unclear risk	Unclear risk	Low risk	Unclear risk	Unclear risk	Low risk
A.334	10.1006/bbrc.2001.4616	Jens Eichhorn, 2001	USA	Investigate the ability of insulin and PDGF to stimulate PLC-γ1 enzyme activity and determine the role of this pathway in glucose transport and GLUT4 translocation	3T3-L1 adipocytes	Stimulation: Insulin (100 ng/ml) or PDGF (20 ng/ml) is used.<br><br>Inhibition: Use U73122 (PLC inhibitor), PCI peptide (PLC-γ1-specific inhibition), or Wortmannin (PI3K inhibitor).<br><br>Rescue: Use OAG (a synthetic DAG that can permeate the membrane).	PLC activity assay: Measuring the hydrolysis of [3 H]−PI−(4.5)P2 in artificial liposomes.<br><br>Measurement of DAG mass: Measurement of DAG mass using DAG kinase and [γ−32P]ATP.<br><br>2-Deoxyglucose uptake assay: Measures the amount of glucose uptake that traces the radioactivity.<br><br>Subcellular fractionation & Western blotting: Cell fractionation and immunosuppression for quantification of GLUT1 and GLUT4 on plasma membranes (PM) and low-density microbodies (LDMs).<br><br>Immunofluorescence microscopy: Observation and quantification of the proportion of cells with GLUT4 translocation to the membrane	PLC activity and DAG levels: Measured at 30 seconds to 30 minutes post-stimulation.<br><br>GLUT4 Translocation and Glucose Absorption: Measured after 20–30 minutes of insulin stimulation.	Basal state (Basal) vs. stimulated state (Insulin or PDGF).<br><br>The stimulant group alone compared to the group with the presence of inhibitors (U73122, Wortmannin, PCI peptides).<br><br>The group with PLC inhibition (by U73122) compared to the group rescued by OAG.	In this study, we investigated the ability of insulin and PDGF to stimulate PLC-γ1 enzyme activity as measured by PI−(4,5)P2 hydrolysis. Both insulin and PDGF induce a rapid increase (<1 min) of PLC activity binding to the respective receptors. Treatment with PDGF resulted in higher and longer-lasting stimulation of PLC-γ1 activity than insulin (0.95 pmol/min/mg vs. 0.68 pmol/min/mg). Furthermore, insulin and PDGF promoted an increase in total cellular DAG, one of the products of PI−(4,5)P2 hydrolysis. Insulin-stimulated PLC activity appears to be downstream of PI-3 Kinase because the increase in DAG was partially blocked by Wortmannin and the addition of PI−(3,4,5)P3 activated PLC-γ1 in vitro. Inhibition of PLCs with U73122 or an inhibitory peptide caused a decrease in 2-deoxyglucose transport and insulin-stimulated GLUT4 translocation, which was restored by the addition of OAG, a synthetic DAG that permeates the cell membrane.	PLC-γ1	Inhibition	PLC-γ1 is located downstream PI3K (inhibited by PIP3) that inhibits insulin-dependent GLUT4 exertion	Signaling										Unclear risk	Unclear risk	Low risk	Unclear risk	Unclear risk	Unclear risk
A.335	10.1074/jbc.274.36.25308	Mary L. Standaert, 1999	USA	Study of the intracellular localization and specificity of insulin-activated atypical Protein Kinase C (aPKC) isoforms in mouse adipocytes, as well as the requirements for phosphorylation of the PDK-1 activated loop and subsequent autophosphorylation.	3T3-L1 adipocytes	Stimulation: Use of insulin (10 nM or 100 nM).<br><br>Genetic Modification: Transfer of mutant structures of PKC-ζ/λ (Thr-410 mutation to Glu or Ala; amputation form Δ1-247).<br><br>Inhibition: Use of RO 31-8220 inhibitors, myristoylated PKC-ζ ζ pseudosubstrate peptides, and Wortmannin.	Immunoprecipitation: Immunoprecipitation to isolate PKC isoforms and GLUT4-containing vesicles.<br><br>Enzyme activity assay: Measure PKC enzyme activity by measuring the radioactivity of 32P integrates into the selective substrate.<br><br>Western blotting: Quantification of proteins and phosphorylation states (P-Thr410 of PKC-ζ, P-Ser473 of PKB).	Monitor over time after insulin stimulation at the following milestones: 0, 0.5, 1, 2, 5, 10, and 15 minutes.	Basal state vs. insulin stimulation.<br><br>Comparison between membrane segments: plasma membrane (PM) and microsome.<br><br>The control group compared to the group that used inhibitors or expressed PKC mutations.	The activity and self-phosphorylation of PKC-ζ and PKC-λ tagged with immune-precipitated epitopes was also increased by insulin in situ and phosphatidylinositol 3,4,5-(PO4)3 (PIP3) in vitro. Since phosphoinositide-dependent kinase-1 (PDK-1) is required for the phosphorylation of the activation loops of PKC-ζ/λ and protein kinase B, we compared their activation. Both RO 31-8220 and myristoylated PKC-ζ pseudosubstrate block insulin-induced activation and autophosphorylation of PKC-ζ/λ but do not inhibit (a) phosphorylation/activation of PDK-1-dependent B protein kinase protein or (b) phosphorylation of threonine 410 during the PKC-ζ activation cycle. In addition, insulin in situ and PIP3 in vitro activated and stimulated the autophosphorylation of a PKC-ζ mutation, in which threonine 410 is replaced by glutamate (but not by an inactivating alanine) and cannot be activated by PDK-1. Surprisingly, insulin activated a PKC-ζ that was cut off that lacked the regulatory domain (presumably the PIP3 binding domain); this may reflect the effect of PIP3 on PDK-1 or the transphosphorylation by PKC-ζ endogenous length. Our findings suggest that insulin activates both PKC-ζ and PKC-λ in the plasma membrane, microsomes, and GLUT4 vesicles by a mechanism that requires an increase in PIP3, PDK-1-dependent phosphorylation at activation loop sites in PKC-ζ and λ, and subsequent autophosphorylation and/or cross-phosphorylation.	PKC-ζ and PKC-λ	Stimulation	PKC-ζ and PKC-λ are activated by insulin through the PIP3 mechanism but are somewhat different from the Akt activation mechanism, or can be activated on their own. PKC-ζ and PKC-λ stimulate GLUT4 proliferation	Signaling										Unclear risk	Unclear risk	Low risk	Unclear risk	Unclear risk	Unclear risk
A.336	10.1074/jbc.C100645200	Bryce A. Nelson, 2002	USA	Study the regulation of insulin to the intracellular distribution of the Munc18-c protein and determine whether this process is altered in 3T3-L1 fat cells that are insulin-resistant by glucose or glucosamine.	3T3-L1 adipocytes	Induced insulin resistance: 18-hour cellular pretreatment with high-concentration glucose (25 mM) or glucosamine (2.5 mM) in combination with low-concentration insulin (0.6 nM).<br><br>Acute stimulation: Use Insulin (100 nM) for 15 minutes.	Subcellular fractionation: Differential centrifugation to separate plasma membranes (PMs) and low-density microsomes (LDMs).<br><br>Triton X-100 solubility separation: Separates the soluble (TS) and insoluble (TI) components in Triton X-100 detergent from the membrane segments.<br><br>Western Blotting: Quantification of Munc18-c, GLUT4, and Caveolin-1 protein expression levels.	Stimulation: 15 minutes for primary experiments; Time-course tracking at 2, 5 and 10 minute marks.	Basal state vs. insulin stimulation.<br><br>Low Glucose Pretreatment (5 mM) vs. High Glucose (25 mM) or Glucosamine.<br><br>The group had chronic insulin (0.6 nM) compared to no in the pretreatment phase.	We examined the subcellular distribution of Munc18-c in response to acute insulin stimulation (15 min, 100 nM) after pre-incubation in 5 or 25 mM of glucose ± 0.6 nM of insulin. Immunospermeability analysis detected Munc18-c mainly in the soluble plasma membrane fraction in Triton X-100 (TS-PM) and the insoluble low-density microsomal fraction in Triton X-100 (TI-LDM). Under each condition except for pre-incubation with high glucose + insulin, acute insulin increased Munc18-c (~50-200%) in TS-PM and decreased Munc18-c (~60%) in TI-LDM. The transport of Munc18-c is time-dependent with a delay of ~3 minutes compared to GLUT4. Pre-incubation with high glucose + 0.6 nM insulin significantly impaired acute insulin-stimulated Munc18-c transport and reduced basal Munc18-c in TI-LDM. Pre-brewing with glucosamine + insulin has the same effect. The total amount of Munc18-c of the cell remains constant. In summary, acute insulin stimulation promotes the translocation of Munc18-c, apparently from a compartment that binds to TI-LDM to TS-PM. Prolonged elevated glucose flow or exposure to glucosamine disrupts this process, which can negatively impact the fusion of GLUT4-containing vesicles with the plasma membrane	Munc18c, Glucose	Stimulation	Acute insulin stimulation promotes the translocation of Munc18-c to the cell membrane. Prolonged elevated glucose or exposure to glucosamine disrupts this process, which can negatively impact the fusion of GLUT4-containing vesicles with the plasma membrane	Tethering										Unclear risk	Unclear risk	Low risk	Unclear risk	Unclear risk	Low risk
A.339	10.1021/bi0018234	Mary L. Standaert, 2001	USA	Study the detailed mechanisms that PIP3 uses to activate PKC-ζ under the action of insulin, including the role of activation ring phosphorylation (T410), autophosphorylation (T560), and intrinsic configuration changes.	rat adipocytes and L6 myotubes	Stimulation: Use Insulin (10 nM) or directly add PIP3 (10 μM).<br><br>Genetically modified: Transfer of mutant structures of PKC-ζ (T410A, T410E, T560A, T560E, and corresponding double mutations) or the amputated form of Δ1–247.<br><br>Inhibition: Use Wortmannin (100 nM) to inhibit PI 3-kinase	Enzyme activity assay: Measures the enzyme activity of PKC-ζ through the level of substrate phosphorylation.<br><br>Autophosphorylation assay: Measures the autophosphorylation of PKC-ζ variants in vitro.<br><br>GLUT4 translocation assay: Quantify the translocation of GLUT4 tagged HA or MYC to the plasma membrane.<br><br>Western blotting: Use of anti-pT410 antibodies to assess the degree of phosphorylation of the activated ring	Acute stimulation: 10 minutes (for enzyme activity).<br><br>GLUT4 translocation: 30 minutes after insulin stimulation.<br><br>Gene expression: After 20-24 hours of plasmid transfer or viral infection	The unprocessed group (Basal) compared to the group stimulated by insulin or PIP3.<br><br>Wild PKC-ζ expression group (WT) compared to mutation-expressing groups (A or E at T410/T560)<br><br>The Wortmannin group compared to the control group.	Presently, we examined: (a) acute effects of insulin on threonine (T)-410 loop phosphorylation and (b) effects of (i) alanine (A) and glutamate (E) mutations at T410 loop and T560 autophosphorylation sites and (ii) N-terminal truncation on insulin- induced activation of PKC-ζ. Insulin acutely increased T410 loop phosphorylation, suggesting enhanced action of 3-phosphoinositide-dependent protein kinase-1 (PDK-1). Despite increasing in vitro autophos- phorylation of wild-type PKC-ú and T410E-PKC-ζ, insulin and PIP3 did not stimulate autophosphorylation of T560A, T560E, T410A/T560E, T410E/T560A, or T410E/T560E mutant forms of PKC-ζ; thus, T560 appeared to be the sole autophosphorylation site. Activating effects of insulin and/or PIP on enzyme activity were completely abolished in T410A-PKC-ζ, partially compromised in T560A-PKC-ζ, T410E/ T560A-PKC-ζ, and T410A/T560E-PKC-ζ, and largely intact in T410E-PKC-ζ, T560E-PKC-ζ, and T410E/ T560E-PKC-ζ. Activation of the T410E/T560E mutant suggested a phosphorylation-independent mechanism. As functional correlates, insulin effects on epitope-tagged GLUT4 translocation were compromised by expression of T410A-PKC-ζ, T560A-PKC-ζ, T410E/T560A, and T410A/T560E-PKC-ζ but not T410E-PKC-ζ, T560E-PKC-ζ, or T410E/T560E-PKC-ζ. Insulin, but not PIP3, activated truncated, pseudosubstrate-lacking forms of PKC-ζ and PKC-λ by a wortmannin-sensitive mechanism, apparently involving PI 3-kinase/PDK-1-dependent phosphorylations but independent of PIP -dependent conforma- tional activation. Our findings suggest that insulin, via PIP3, provokes increases in PKC-ζ enzyme activity through (a) PDK-1-dependent T410 loop phosphorylation, (b) T560 autophosphorylation, and (c) phosphorylation-independent/conformational-dependent relief of pseudosubstrate autoinhibition.	PKC-ζ	Stimulation	Insulin, via PIP3, induces an increase in PKC-ζ enzyme activity through (a) PDK-1-dependent T410 ring phosphorylation, (b) T560 self-phosphorylation, and (c) self-inhibition of non-phosphorylation/configuration-dependent pseudosubstrate relief	Signaling										Unclear risk	Unclear risk	Low risk	Unclear risk	Unclear risk	Unclear risk

A.340	10.1242/jcs.01421	Dora Török, 2004	Canada	Comparison of the cellular mechanisms used by insulin and PDGF to mobilize GLUT4 to the surface of muscle cells, focusing on the role of actin restructure and the v-SNARE VAMP2 protein.	L6 myoblasts express GLUT4 stably tagged with Myc (GLUT4myc).	Stimulation: Use of insulin (100 nM) or PDGF-BB (50 ng/ml).<br><br>Actin disruption: Use of Latrunculin B, cytochalasin D, and jasplakinolide to disrupt the kinetics of actin.<br><br>V-SNARE inhibition: Light-chain intake of tetanus toxin (TeTx) to cleave sensitive v-SNAREs such as VAMP2 and VAMP3.	Colorimetric assay: Colorimetric measurement to quantify GLUT4myc on the cell surface.<br><br>Immunofluorescence & Confocal microscopy: Confocal microscopy to observe the distribution of GLUT4 and actin restructuring morphology (actin-stained phalloidin).<br><br>2-deoxyglucose uptake assay: Measures the level of glucose uptake.<br><br>Immunoblotting (Western blot): Quantification of Akt and phosphorylation of Akt (Ser 473).	PDGF: Peaks GLUT4 translocation and glucose uptake after 5 – 7.5 minutes, then gradually decreases and disappears after 20 minutes.<br><br>Insulin: Peaks after 10 minutes and remains stable for at least 20-30 minutes.<br><br>Akt Signal: Measured at 1, 7 and 30 minute marks.	Basal state versus insulin or PDGF stimulation.<br><br>The control group (DMSO/Empty vector) compared to the actin inhibitor or TeTx treatment group.<br><br>Comparison between Insulin and PDGF in terms of dependence on cellular components	In Myc-tagged GLUT4-tagging L6 myoblasts, none of the stimulators affected the rate of GLUT4 cell entry, confirming that they work primarily by enhancing the propulsion to increase GLUT4 at the cell surface. Although GLUT4myc surfaces in response to insulin peak at 10 minutes and remain stable for 30 minutes, PDGF's action is transient, peaking at 5 minutes and disappearing at 20 minutes. These GLUT4myc translocation time developments reflect the phosphorylation of Akt by two excitators. Interestingly, insulin and PDGF cause distinct expressions of actin restructuring. Insulin causes discrete, long (> 5 μm) dorsal dorsal (dorsal) structures at the periphery of the cell, while PDGF causes many short dorsal structures (< 5 μm) throughout the cell, including above the nucleus. Latrunculin B, cytochalasin D, and jasplakinolide, which disrupted actin dynamics, prevented insulin- and PDGF-induced actin reconstruction but only significantly inhibited GLUT4myc translocation in response to insulin (75-85%, P<0.05), not with PDGF (20-30% inhibition). Furthermore, mild-chain transfusion of tetanus toxin, which cleaves v-SNARE VAMP2 and VAMP3, reduced insulin-induced GLUT4myc translocation by > 70% but did not affect the response to PDGF. These results suggest that insulin and PDGF rely on the actin cell skeleton and on tetanus toxin-sensitive VAMPs in different ways to mobilize GLUT4.	PDGF-BB (A Type of Growth Factor)	Stimulation	PDGF stimulates GLUT4 1 proliferation transiently and rapidly, and restructures the actin skeleton differently than insulin and is less affected by actin skeleton destruction than insulin and can act on VAMPs in a different way than insulin	Signaling										Unclear risk	Unclear risk	Low risk	Unclear risk	Unclear risk	Low risk	
A.341	10.1242/jcs.138982	Ariel Contreras-Ferrat, 2014	Chile	Examine the impact of H2O2 on glucose transport and insulin-dependent GLUT4 translocation in skeletal muscle cells, and determine the origin and mechanism of related calcium signals	L6 line skeletal muscle cells express stable GLUT4 tagged extracellular Myc (L6-GLUT4myc myotubes).<br>Primary skeletal muscle cells isolated from Sprague-Dawley neonatal rats	Stimulation: Use of Insulin (100 nM) or exogenous H2O2 (0.001 – 1 mM).<br><br>Oxidative Inhibition: Use of antioxidants N-acetylcysteine (NAC), Trolox, or Catalase-expressing adenovirus infusion (AdCat).<br><br>Inhibition of Enzymes/Channels: peptide gp91-ds-tat or siRNA to block NOX2/p47phox; Ryanodine to block the RyR channel; Xestospongin B for IP3R channel blocking; Ruthenium Red (RuRed) to block MCUs in mitochondria	Real-time ROS sensing: Use the HyPer molecular sensor to measure H2O2 in the cytoplasm in real time<br><br>Intracellular Ca imaging: Using fluorescent indicators Fluo4 am and Fluo3 am in combination with a homofocal microscope.<br><br>GLUT4 translocation assay: Quantify GLUT4myc on the cell surface using ELISA colorimetry (492 nm absorption) or single-cell fluorescence immunostaining.<br><br>Glucose uptake assay: Measures the amount of radioactive tracer glucose ([3H] -2-deoxyglucose) or fluorescent analogue (2-NBDG).<br><br>Redox modification detection: Using a high-resolution Proximity Ligation Assay (PLA) technique to detect specific S-glutathionylation on RyR1.	ROS and Ca Signals: Continuous measurement immediately after stimulation (H2O2 peak detected after ~7 seconds, Ca peak after seconds to minutes).<br><br>GLUT4 Translocation and Glucose Absorption: Measured after 20 minutes of stimulation by printing	Basal state vs. insulin or H2O2 stimulation state<br><br>The control group (Control/siSc/AdEmpty) versus the group that was inhibited or genetically interfered with (siRNA, AdCat, AdSOD1).<br><br>The stimulant group alone compared to the group that combined calcium channel inhibitors or antioxidants.	We tested the effects of H2O2 on insulin-dependent glucose transport and GLUT4 translocation in skeletal muscle cells. H2O2 increased the translocation of GLUT4 with an extracellular Myc-epitope tag between the first and second transmembrane domains (GLUT4myc), an effect that is cumulative to the effect of insulin. The antioxidants N-acetyl L-cysteine and Trolox, p47phox-NOX2 inhibitor peptides, pp91-ds-tat or p47phox knockdown each reduce insulin-dependent GLUT4myc translocation. Importantly, gp91-ds-tat suppressed the production of insulin-dependent H2O2. A ryanodine receptor channel (RyR) agonist stimulated GLUT4myc translocation and insulin stimulated the release of RyR1-mediated Ca2+ by promoting S-glutathionation of RyR1. This pathway works in tandem with insulin-mediated stimulation of Ca2+ channels activated by inositol-1,4,5-trisphosphate (IP3), in response to the activation of phosphatidylinositol 3-kinase and its downstream target of phospholipase C, resulting in the transfer of Ca2+ into the mitochondria. An inhibitor of IP3 receptors, Xestospongin B, reduced both insulin-dependent IP3 production and GLUT4myc translocation. We propose that, in addition to the α,β phosphatidylinositol 3-kinase to Akt pathway, insulin is also involved in both the release of RyR-mediated Ca2+ and the uptake of Ca2+ into mitochondria via the IP3 receptor, and that these signals together stimulate glucose uptake	H2O2, Ca2+	Stimulation	Insulin increases cytoplasmic H2O2 production through PKC-dependent NOX2 activation, thereby H2O2 induces the S-glutathionation of RyR1 in skeletal muscle cells, rapidly increasing the amount of GLUT4 expansion, thereby stimulating GLUT4 exposition.<br>- insulin also activates IP3R (due to increased intracellular IP3 levels), thereby increasing mitochondrial Ca2+, which in turn also stimulates GLUT4 transport to the cell membrane	Signaling										Unclear risk	Unclear risk	Low risk	Unclear risk	Unclear risk	Unclear risk	
A.342	10.1074/jbc.273.13.7201	Mónica R. Calera, 1998	USA	Investigate the subcellular distribution and regulation of Akt-2 by insulin in mouse fat cells, and examine the physical association between Akt-2 and GLUT4-containing vesicles.	Adipocytes isolated from the testicular crestal fat cushion of male Sprague-Dawley rats.	Stimulation: Use of 10 nM concentration of Insulin.<br>• Inhibition: Use a 100 nM concentration of Wortmannin to inhibit PI 3-kinase.	Subcellular fractionation: Differential centrifugation for the separation of plasma membranes (PM), cytoplasm (CYTO), low-density (LM) and high-density (HM) microbodies.<br>• Sucrose velocity gradient: Deep segmentation of microsomes to check the colocation of proteins.<br>• Immunoisolation: Using particle-bound 1F8 antibodies to pull GLUT4-containing vesicles and examine the accompanying protein.<br>Western Blotting: Quantify the expression and electrophoresis mobility displacement of Akt-1, Akt-2, and GLUT4 proteins.	Insulin Stimulation: For 10 minutes.<br>• Wortmannin Pretreatment: For 25 minutes before adding insulin.	Basal/Control compared to insulin stimulation.<br>• The stimulant group alone compared to the group with the Wortmannin inhibitor present.<br>• Distribution of Akt-1 vs. Akt-2.	In this report, an analysis of the subcellular distribution of Akt-2 in mouse fat cells shows that Akt-2 is present in significant amounts in various membrane compartments, as well as in the cytoplasm, and these membrane compartments include low-density microsomes where Glut4 is present in the basal state. The distribution of Akt-2 in resting fat cells was found to overlap significantly with the distribution of Glut4 when low-density microsoms were segmented by velocity sucrose concentration scale, indicating colocality. We confirmed the colocalization of Akt-2 and Glut4 in the baseline state by immunological purification of Glut4 vesicles, which showed a 5.5-fold increase in Akt-2 intake in response to insulin compared to Glut4 intake. These results are consistent with the possibility that Akt-2 may be involved in the translocation of the Glut4 vesicles.	Akt2	Stimulation	Akt-2 in the endothelial cavities is activated by insulin, which in turn stimulates GLUT4 exfoliation	Signaling										Unclear risk	Unclear risk	Low risk	Unclear risk	Unclear risk	Unclear risk	
A.344	10.1093/emboj/cdg402	Tania Maffucci, 2003	UK and Italya	Investigate whether insulin specifically stimulates the formation of PtdIns-3-P and determine the role of this lipid in the transfer of GLUT4 to the plasma membrane.	Skeletal muscle cells of the L6 line and fat cells of the 3T3-L1 line.	Stimulation: Insulin (300 nM), PDGF (20 ng/ml), or EGF.<br>• Genetic modification: Transfer of TC10 mutations (Q75L constitutional activation or T31N dominant inhibition) and Cdc42 mutations.<br>• Chemical inhibition: Use of PI 3-K inhibitors (Wortmannin, LY294002) and cholesterol extract drugs (MBCD).<br>• Lipid loading: Exogenous PtdIns-3-P is introduced into cells through polyamine carriers.	Phospholipid Analysis (HPLC): Extraction and separation of glycerophosphoinositides marked with [^3H]inositol.<br>• Confocal microscopy: Monitors the displacement of GFP-2XFYVEHrs fluorescent probes (specific for PtdIns-3-P) and GFP-tagged GLUT4.<br>• Glucose Absorption Test: Measures the amount of radioactive or fluorescent tracer 2-deoxyglucose absorption.<br>• Pak1 PBD pull-down assay: Measures the level of activation (GTP-mounted) of the TC10.<br>• Detergent-free fractionation: Separation of lipid rafts with sucrose gradients	Lipid Signals: Measured after 1, 3, 5, 10, 15, and 30 minutes of stimulation.<br>• GLUT4 Translocation and Glucose Absorption: Measured for approximately 10 - 30 minutes after stimulation.	Basal state vs. insulin or PDGF/EGF stimulation.<br>• Empty vector compared to TC10 mutation expression group.<br>• The stimulant group alone compared to the group with the presence of inhibitors (Wortmannin, LY294002, MBCD).<br>• Effects of exogenous PtdIns-3-P compared to other phosphoinositides (PtdIns-4-P, PtdIns-3,4,5-P3).	Here we show that insulin specifically stimulates the formation of PtdIns-3-P in insulin-responsive cells. Insulin-mediated PtdIns-3-P formation occurs through TC10 activation at the lipid rafts subregion of the plasma membrane. Exogenous PtdIns-3-P causes translocation to the plasma membrane of both overexpressed and endogenous GLUT4. These data indicate that PtdIns-3-P is specifically produced downstream from insulin-mediated activation of TC10 to promote metabolism to the plasma membrane of GLUT4.	PtdIns-3-P	Stimulation	PtdIns-3-P is produced through TC10 activation of insulin in lipid raft subregions on the plasma membrane. From there, activating GLUT4 transport to the membrane	Signaling										Unclear risk	Unclear risk	Low risk	Unclear risk	Low risk	Low risk	
A.347	10.1210/en.2003-0609	Ann Louise Olson, 2003	USA	The study aims to demonstrate that insulin stimulates the formation of polymerized microtubules that are independent of Phosphatidylinositol 3-Kinase (PI 3-kinase) but dependent on actin, and that the microtubule network is an intracellular target of the insulin signaling chain. Demonstrate that the partial depolymerization of microtubules is sufficient to inhibit insulin-mediated GLUT4 migration	3T3-L1 adipocytes	1. Insulin stimulation: 100 nM for time experiments (0, 1, 5, 15 minutes) and 0, 1, 10, 100 nM for dose response experiments.<br><br>2. Genistein (Tyrosine Kinase Inhibitor): 300 μM pretreatment for 1 hour.<br><br>3. PD98059 (MEK/MAPK inhibitor): 100 μM pretreatment for 1 hour.<br><br>4. Wortmannin (PI 3-kinase inhibitor): 100 nM pretreatment for 1 hour.<br><br>5. Latrunculin B (Actin breakdown): 60 μM pretreatment for 2 hours.<br><br>6. Nocodazole (Microtubule Depolymerization): 2 μM, 10 μM, and 33 μM to set measurement limits.<br><br>7. Taxol (Maximum Polymerization): 10 μM to set the measurement limit.	1. Quantification of Polymerized/Monomeric Tubulins: Differential detergent extraction, then Western Blot analysis for α-tubulin.<br><br>2. Morphological assessment/Microtubule fluorescence intensity measurement: Immunofluorescence using α-tubulin and γ-tubulin antibodies, analysis by confocal microscopy.<br><br>3. Measure GLUT4 displacement: Plasma membrane sheet assay and then analyze the Western Blot for GLUT4.<br><br>4. Signal Activity Evaluation: Western Blot for Measurement of Tyrosine Phosphorylation (using PY99 antibodies), Phospho-PKB/akt1 (Ser473), and Phospho-ERK1/2	1. Polymerization of Tubulin (Kinetics): 0, 1, 5, or 15 minutes of insulin stimulation (100 nM).<br><br>2. Polymerization of Tubulin (Dose Response): 15 minutes of insulin stimulation (0, 1, 10, 100 nM).<br><br>3. Microtubule Regrowth Experiment (γ-tubulin): 0, 1, 3, 10, and 30 minutes after nocodazole washing.<br><br>4. GLUT4 displacement: 20 minutes of insulin stimulation (100 nM), after 1 hour of nocodazole pretreatment.<br><br>5. Inhibitory Signal Rating (PKB/ERK): 0, 1, or 15 minutes of insulin stimulation (100 nM) after 1-hour inhibitory pretreatment	1. Control group (Basal): Cells without insulin treatment (0 min or 0 nM insulin time).<br><br>2. Maximum/Minimum Control Group: Cells treated with taxol (maximum polymerization) and nocodazole (maximum depolymerization, 33 μM).<br><br>3. Signal suppression group: Comparison of the pretreatment group with genistein, PD98059, wortmannin, or latrunculin B + insulin, with the insulin-only group.<br><br>4. Regrowth Group: Cells were recultured in an insulin-free versus insulin-free environment, after complete depolymerization with nocodazole.<br><br>5. GLUT4 Inhibitory Group: Cells were pretreated with 2 μM or 10 μM nocodazole compared to the control group.<br><br>1. Control group (Basal): Cells without insulin treatment (0 min or 0 nM insulin time).<br><br>2. Maximum/Minimum Control Group: Cells treated with taxol (maximum polymerization) and nocodazole (maximum depolymerization, 33 μM).<br><br>3. Signal suppression group: Comparison of the pretreatment group with genistein, PD98059, wortmannin, or latrunculin B + insulin, with the insulin-only group.<br><br>4. Regrowth Group: Cells were recultured in an insulin-free versus insulin-free environment, after complete depolymerization with nocodazole.<br><br>5. GLUT4 Inhibitory Group: Cells were pretreated with 2 μM or 10 μM nocodazole compared to the control group.	Insulin increases α-tubulin polymerization over time–dose and goes hand in hand with increased GLUT4 transport; this effect requires tyrosine kinase, PI3K/MAPK-independent, and intact actin network-dependent (completely lost with latrunculin B). Simply partial disruption of microtubules with 2 μM of nocodazole also reduced GLUT4 displacement by ~30%, suggesting that microtubule stability is essential for insulin GLUT4 response.	α-tubulin	Stimulation	Insulin signals to the microtubule network via a pathway independent of PI 3-kinase and independent of MAPK, but is dependent on Tyrosine Kinase and dependent on the intact actin reticulum. This mechanism does not promote de novo nucleation, but rather promotes the growth and/or stabilization of existing microtubules.	translocation										Unclear risk (Not reported)	Unclear risk (Not reported)	Low risk	Unclear risk (Not reported)	Unclear risk (Not reported)	Low risk	
A.348	10.1016/s0021-9258(18)46875-5	Michael J. Quon, 1994	USA	The study aimed to investigate the possible role of IRS-1 (Insulin Receptor Substrate 1) in mediating the effects of insulin to stimulate glucose transport in insulin-sensitive target tissue (specifically mouse fat cells)	Adipose cells isolated from epididymal fat pads of male rats, were primarily cultured and transient transfected with the epitope-attached GLUT4 encoding gene (GLUT4-HA)	1. Insulin stimulation with different concentrations (0, 0.024, 0.072, 0.3, 60 nM).<br><br>2. Knockdown of endogenous mouse IRS-1 expression: By mutating antisense ribozyme (rIRS1 Ribozyme) targeting the mRNA of the IRS-1 mice.<br><br>3. Exogenous IRS-1 overexpression: By transfusing human IRS-1 encoding cDNA (pCIS-hIRS1).<br><br>4. Rehabilitation experiments: Ribozyme and hIRS-1 co-transformers.	1. Assay for Cell Surface Epitope-tagged GLUT4.<br><br>2. Double antibody assay: Using anti-HA1 (12CA5) monoclonal antibody to identify the epitope of GLUT4, combined with the 125I-sheep anti-mouse IgG secondary antibody to quantify radiation on the cell surface.<br><br>3. Insulin dose-response curves analysis to determine the maximum half-stimulation dose (ED50)	1. Post-transformation culture time: 16–24 hours after electrophoresis.<br><br>2. Insulin Stimulation Time: 30 minutes at 37°C	1. Group 1 (Ribozyme Control): GLUT4-HA and Vector Ribozyme Control Cells.<br><br>2. Group 2 (Overexpression Control): GLUT4-HA transgenic co-cells and pCIS2 hollow vectors.<br><br>3. Group 3 (Co-transgenic control): Ribozyme rIRS1, pCIS2 and GLUT4-HA co-transgenic cells.	Reduced IRS-1 (due to ribozyme) decreases insulin sensitivity, which requires 4.4 times higher insulin levels to reach the maximum semi-responsive GLUT4 stimulation but constant maximum response; conversely, increased human IRS-1 expression increases surface GLUT4 even in the absence of insulin. When co-converters load human IRS-1 into ribozyme-bearing cells, insulin sensitivity is restored (left fluid curve). These results demonstrate that IRS-1 is a key factor in insulin-stimulated glucose transport.	IRS-1	Stimulation	This study demonstrates the role of IRS-1 in the metabolic signaling pathway of insulin that leads to GLUT4 shifting in a physiology-related cell type (fat cells), which is different from its known role in mitogenic effects	Signaling										Unclear risk (Not reported)	Unclear risk (Not reported)	Low risk	Unclear risk (Not reported)	Unclear risk (Not reported)	Low risk	
A.349	10.1074/jbc.272.47.29829	Lixin Zhou, 1997	USA	The study aims to demonstrate that IRS-2 (Insulin Receptor Substrate-2) has the ability to mediate the migration of the insulin-responsive glucose transporter GLUT4 in physiologically relevant insulin-sensitive target cells (mouse fat cells). Determine if IRS-2 can provide an alternative pathway for the vital metabolic actions of insulin	1. Primary subject: Adipose cells isolated from epididymal fat pads of male rats, primary cultured and transient transfected with the epitope-binding GLUT4 encoding gene (GLUT4-HA).<br><br>2. Secondary subject: Freshly isolated rat adipose cells for precipitation immunoassay	1. Insulin Stimulation: At low concentrations (0.07 nM) and maximum effective concentrations (60 nM).<br><br>2. Exogenous IRS-1 Overexpression (hIRS-1): By co-loading the hIRS-1 vector along with GLUT4-HA.<br><br>3. Increased expression of exogenous mouse IRS-2 (mIRS-2): By co-loading the mIRS-2 vector along with GLUT4-HA.<br><br>4. PI 3-kinase binding ability analysis: Stimulation of untransformed mouse fat cells with 60 nM insulin for 2 minutes	1. Measurement of GLUT4-HA on the cell surface: Using anti-HA monoclonal antibody (HA-11) in combination with 125I-labeled sheep anti-mouse IgG (Radioimmunoimmunology) secondary antibody.<br><br>2. Measurement of binding of PI 3-kinase to endogenous IRS-1 and IRS-2: Co-immunoprecipitation method followed by analysis of Western Blot with anti-p85 antibodies (subunits of PI 3-kinase).<br><br>3. Measurement of Tyrosine phosphorylation of exogenous IRS-1 and IRS-2: Immunoblotting on total membrane fractions using anti-phosphotyrosine (4G10) antibodies.<br><br>4. Protein Expression Level Test: Western Blot for IRS-1, IRS-2, and GLUT4-HA (using anti-IRS-1, anti-IRS-2, and anti-HA).	1. GLUT4 displacement measurement: After 20 hours since the feeding. Insulin Stimulation: The cells were treated with insulin (0.07 nM or 60 nM) for 30 minutes.<br><br>2. Measurement of PI 3-kinase binding and Tyrosine phosphorylation: Cells treated with insulin (60 nM) for 2 minutes.	1. Control group (Control/Empty vector): GLUT4-HA comodulated cells and hollow expression vector (pCIS2).<br><br>2. The group that increases IRS-1 expression: GLUT4-HA and hIRS-1 co-transformer cells.<br><br>3. The IRS-2 expression enhancement group: GLUT4-HA and mIRS-2 comodulated cells.<br><br>4. Endogenous comparison: Endogenous IRS-1 vs. Endogenous IRS-2 in binding to PI 3-kinase.<br><br>5. Basal state: The above groups do not stimulate insulin, compared to insulin stimulation (0.07 nM and 60 nM)	Co-immunity showed that IRS-1 binds PI3K twice as strongly as IRS-2 after insulin stimulation; Increased expression of IRS-1 or IRS-2 both increases surface GLUT4 in the baseline, and at 60 nM insulin, surface GLUT4 increases with 130% (IRS-1) and 135% (IRS-2) compared to controls. The results show that IRS-2 also activates the GLUT4 migration pathway, which acts as an alternative signaling arm to IRS-1 in the metabolic response of insulin.	IRS-2	Stimulation	This study directly demonstrates that IRS-2 has an intrinsic capacity to mediate the metabolic action of insulin (GLUT4 transfer) in fat cells, thereby providing direct evidence that IRS-2 acts as a parallel signaling pathway that compensates for IRS-1 in metabolic actions	Signaling										Unclear risk (Not reported)	Unclear risk (Not reported)	Low risk	Unclear risk (Not reported)	Unclear risk (Not reported)	Low risk	
A.350	10.1074/jbc.M301517200	Li-Bin Liu, 2003	Japan	The study aimed to investigate the physiological implications of microtubules in subcellular positioning and trafficking of the glucose transporter GLUT4 in primary mouse adipocytes.	Isolated adipocytes from epididymal adipose tissues of Sprague-Dawley rats	1. Destruction of microtubules: Nocodazole (concentrations of 1, 10, 100 μM) to disrupt microtubules.<br><br>2. Stimulation of displacement: Insulin (usually 10 nM) to stimulate glucose transport and GLUT4 displacement.<br><br>3. Other stimulation: GTPγS (1 mM) is used to stimulate glucose transport in electropermeable cells.<br><br>4. Inhibition of glucose transporter: Phloretin (1 mM)<br><br>5. Cell conditioning: Use intact cells or electrically permeabilized cells.<br><br>6. Intraphrystagnant Inhibition: Peptide Dk-(62–85)(50 μM)	1. Measurement of glucose uptake: Estimation of cellular glucose transport activity by measuring the rate of absorption of 3-O-methyl-D-glucose by the method of oil flotation.<br><br>2. Quantification of Polymerized/Monomeric Tubulins: Extraction and Immunoblotting (Western Blot) using anti-α-tubulin antibodies.<br><br>3. Measurement of GLUT4 displacement by membrane fractionation: Preparation of plasma membrane (PM) and low-density microsomal (LDM) fractionation by centrifugation, then immunoblotting for GLUT4, GLUT1, IRAP, VAMP-2, and VAMP-3.<br><br>4. Cell surface GLUT4 measurement (Translocation): Method of surface labeling using trypsin cleavage (35-kDa fragment generation).<br><br>5. Morphological Analysis/Positioning: Immunofluorescence and Confocal Microscopy for visualization of α-tubulin, GLUT4 and vimentin, including 3D image reconstruction.<br><br>6. Intraphrical measurement: Measure the intraphageal rate of the 35-kDa GLUT4 fragment in the LDM segment.<br><br>7. Insulin Signal Measurement: Immunoblotting to detect tyrosine phosphorylation (IRS-1/2, c-Cbl) and Akt phosphorylation (Ser-473).<br><br>8. Measurement of Phosphodiesterase Activity: Analysis of insulin-sensitive cAMP Phosphodiesterase activity.	1. Breaking the microtubule (Nocodazole): 30 minutes.<br><br>2. Insulin Stimulation (Glucose Transfer/Absorption): 30 minutes (most translocation experiments).<br><br>3. Insulin Stimulation (Signal): 1 minute or 20 minutes (for protein phosphorylation).<br><br>4. Stimulation of GTPγS (Electropermeable Cells): 20 minutes.<br><br>5. Endocytosis Measurement: Intervals of 0, 5, 10, or 20 minutes after washing trypsin.<br><br>6. Glucose transport stimulation kinetics: Intervals 0, 1, 3, 5, 10, 20, and 30 minutes.	1. Control Cells: Untreated (Basal) or solvent-only (Me2SO) cells.<br><br>2. Insulin Cells: Insulin Stimulating Cells vs. Control.<br><br>3. Nocodazole + Insulin Cells: Compared to the insulin-only group (to assess the inhibition of displacement).<br><br>4. Insulin Permeable Cells vs. GTPγS: Comparison of sensitivity to nocodazole between insulin stimulation and GTPγS stimulation.<br><br>5. Intracellular Inhibitory Cells: Nocodazole + Dk-(62–85) peptide treatment cells vs. Insulin + Dk-(62–85).<br><br>6. Direct transporter activity test: Comparison of Nocodazole (100 μM) with Wortmannin (inhibition of PI 3-kinase) and Phloretin (direct inhibition of glucose transporter) in ATP-deficient cells.	Microtubule disruption with nocodazole almost completely inhibited 50% insulin-induced glucose transport with reduced GLUT4 migration, while delaying t1/2 transport (7 vs. 2.3 minutes) in which GLUT4 endophagites are less affected. Inhibition of intraphages does not fully restore transport, suggesting a microtubule-dependent GLUT4 subpopulation. Nocodazole also decreased IRAP and VAMP-2 migration but did not affect GLUT1/VAMP-3, and in electropermeable cells, insulin reduced glucose transport by 40% when microtubules were broken, and GTPγS was not affected. Data demonstrate that insulin recruits GLUT4 from at least two distinct intracellular compartments via different microtubule-dependent transport routes.	Microtubes	Stimulation	Insulin recruits GLUT4 to the plasma membrane from at least two distinct intracellular reservoirs with different microtubule dependence<br>Pathway 1: Microtubule-dependent.<br>Pathway 2: Little or no microtubule dependence, which may correspond to the recycled endosome system.	Translocation										Unclear risk (Not reported)	Unclear risk (Not reported)	Low risk	Unclear risk (Not reported)	Unclear risk (Not reported)	Low risk	

A.351	10.1091/mbc.11.12.4079	Georg Ramm, 2000	Netherlands and Australia	The goal is to address the precise nature of GLUT4-carrying intracellular compartments by performing extensive morphological analysis, using a novel chemical immunoimmunology technique that enables high-performance labeling, and 3D resolution of cytoplasmic rims isolated from rat adipose cells.	Adipocytes isolated from epididymal fat pads of male Ola (SD-like)	1. Insulin Stimulation: Insulin treatment at a concentration of 700nM (maximum effective concentration) for 20 minutes.<br><br>2. New sample collection technique: Separation of cytoplasmic rims by clamping fat cells between EM grid and nitrocellulose membrane, then mechanical breakdown to obtain 3D intact cytoplasmic borders for analysis by Immunoelectron Microscopy (IEM).	1. Using a new morphological method: Whole Mount Transmission Electron Microscopy (EM) of cytoplasmic rims<br>.<br>2. Immuno-gold labeling with high labeling efficiency and 3-d resolution.<br><br>3. Quantitative IEM: Quantify the distribution of GLUT4 across different morphological structures	After insulin treatment: After 20 minutes of stimulation	Basal cells vs. insulin-treated cells	In basal cells, GLUT4 is localized in small vesicles, tubules, and vacuoles; Insulin increases GLUT4 on the surface mainly reduces small vesicles, while tubes and non-planers remain constant. Many small vesicles contained IRAP (88%) and VAMP2 (57%) but did not have an endosome/lysosome marker, while 56% of GLUT4 vesicles contained CD-MPR. Insulin mobilizes GLUT4 from both VAMP2 vesicles and CD-MPR vesicles, but GLUT4 in VAMP2 vesicles remains constant, while CD-MPR vesicles decreases, indicating that GLUT4 is recruited by both vesicle fusion and endosomal-TGN sorting.	GLUT4<br><br>Markers: VAMP2 (v-SNARE), IRAP, and CD-MPR	Stimulation	Insulin recruits GLUT4 onto the plasma membrane from two distinct functional intracellular compartments via two different transport mechanisms:<br>VAMP2-positive/CD-MPR-negative specialized cysts: Recruitment by direct fusion of preexisting VAMP2-carrying vesicles<br>TGN/Endosome System (CD-MPR-positive): Recruitment by the mechanism of sorting GLUT4 out of the dynamic endosomal (TGN system)	Translocation										Unclear risk (Not reported)	Unclear risk (Not reported)	Low risk	Unclear risk (Not reported)	Unclear risk (Not reported)	Low risk
A.352	10.1074/jbc.M806460200	Vladimir A. Lizunov, 2009	USA	Study on the effect of Exo70 protein on tethering and fusion of glucose transporter 4 (GSV) vesicles in primary isolated rat fat cells	Primary isolated adipose cells from male CD rats	1. Insulin stimulation: Insulin stimulation at a concentration of 70nM (for HA-GLUT4 exposure experiments) or 100nM (for TIRF experiments)1515.<br><br>2. Transfection:<br>- Exo70 Wild Type (Exo70-WT): Increases the expression of exo70 wild type16.<br>- Exo70 Mutant (Exo70-N): Increased expression of Exo70-N mutation (amino acid 1-384, dominant-negative effect) 171717.	1. Total Reflectance Microscopy (TIRF) Microscopy: Used to observe and quantify the traffic, tethering, and fusion of GLUT4 vesicles living near the cytoplasmic membrane.<br><br>2. Cell Surface Antibody Binding Assay: Use anti-HA antibodies (bound to GLUT4) under non-permeabilized conditions and I125-marked secondary antibodies to quantify the amount of GLUT4 fused into the plasma membrane (fusion evaluation)<br><br>3. Confocal Microscopy: Used to evaluate the level of protein co-expression and visualize the subcellular localization of GLUT4 and Exo70.	1. Basal state: Cells are not stimulated by insulin.<br><br>2. After insulin stimulation: 15 minutes or 30 minutes after insulin stimulation.<br><br>3. Real-time tracking: Collect time-lapse images.	1. Group 1 (Control): Cells only express GLUT4-GFP (or HA-GLUT4-GFP/mCherry).<br><br>2. Group 2 (WT experiment): Copper cells expressing GLUT4 and Exo70-WT.<br><br>3. Group 3 (Mutant Experiment): Copper cells expressing GLUT4 and Exo70-N (Mutant).<br><br>4.So Signal Comparison: Compare the above groups in the Basal (non-insulin) state and the Insulin state.	Insulin increases Exo70 near the membrane and stimulates both tethering and fusion of the GLUT4 vesicles. Exo70-WT overexpression does not change the fusion rate or reveal GLUT4, but Exo70-N mutant causes spontaneous, insulin-free tethering, even though these vesicles do not fusion. When insulin is added, the GLUT4 vesicles that have been pre-tethered by the Exo70-N remain fusion without moving, indicating that fusion is the speed-limiting, insulin-controlled downstream step of Exo70-mediated tethering.	Exo70	Stimulation	The fusion of GLUT4 vesicles, rather than tethering, is a speed-limiting step in insulin-regulated GLUT4 exfoliation, located downstream of Exo70-involved tethering	Tethering and fusion										Unclear risk (Not reported)	Unclear risk (Not reported)	Low risk	Unclear risk (Not reported)	Unclear risk (Not reported)	Low risk
A.354	10.1007/s00125-015-3627-3	Francoise Koumanov, 2015	UK and Australia	The goal is to identify Rab proteins and insulin-activated Rab effectors, which promote GLUT4 translocation.	1. Primary rat adipocytes: From the epididymal tissue of Wistar mice (used for most GTP and Rab3B mutant binding experiments).<br><br>2. 3T3-L1 fat cells: Used for siRNA knockdown experiments of Rab3 isoforms.	1. Insulin stimulation: (100 nmol/l).<br><br>2. Use photoaffinity agent (Photolabel): Bio-ATB-GTP to discover GTP-bound proteomes.<br><br>3. Genetic Modification (Overexpression): Expression of Rab3B (Wild-type, Q81L, T36N) and Noc2 (Wild-type, Noc2AAA) structures.<br><br>4. Gene silencing (siRNA silencing): Silencing of Rab3 isoforms (A, B, D) on 3T3-L1 cells.<br><br>5. Inhibition of PI 3-kinase: By Wortmannin (200 nmol/l).	1. Measurement of the GTP binding state of G-protein: Using Bio-ATB-GTP combined photoaffinity, bidirectional gel electrophoresis, and MALDI-TOF MS/nano-LC MS/MS mass spectrometry.<br><br>2. Measurement of GLUT4 translocation on the plasma membrane (PM translocation): Detection of HA-GLUT4 surface antigen on cells or using Bio-ATB-BGPA photoaffinity tag.<br><br>3. Glucose transport measurement: 2-deoxy[3H]glucose absorption measurement.<br><br>4. Protein interaction studies: Pull-down techniques using recombinant proteins (FLAG-Rab3B, GST-Noc2, MBP-Noc2) and Western blotting.<br><br>5. Subcellular site analysis: Membrane subfractionation and confocal microscopy to compare Noc2 and GLUT4 on the cell surface.	1. Stimulation of Rab3B GTP loading: 2 minutes and 20 minutes after insulin.<br><br>2. Measurement of GLUT4 Translocation and Glucose Transport: 30 minutes of insulin (steady state measurement).	1. Basal vs. Insulin-stimulated.<br><br>2. Empty vector control compared to Rab3B WT, Q81L (constitutively active), and T36N (dominant negative) structures.<br><br>3. Non-targeting siRNA (Non-targeting siRNA control) compared to Rab3 siRNA (A, B, D) alone or in combination.<br><br>4. Noc2 Wild-type (WT) vs. Noc2AAA (Rab-binding deficient mutant)<br><br>5. With and without Wortmannin treatment	Insulin activates Rab3B (a G-protein in fat cells), and the Rab3 isoform is the main regulator of GLUT4 translocation. Insulin promotes Rab3 to bind to GTP, which disrupts the Rab3–Noc2 interaction, causing Noc2 to leave the plasma membrane and lose its inhibitory effect, thereby enhancing GLUT4 translocation.	Rab3B and other Rab3 isoforms	Stimulation	Insulin regulates GLUT4 translocation by activating the cleavage of the Rab3-Noc2 complex, which causes Noc 2 to be displaced from the membrane into the cell, promoting GSV translocation and fusion.	Docking and fusion										Unclear risk (Not reported)	Unclear risk (Not reported)	Low risk	Unclear risk (Not reported)	Unclear risk (Not reported)	Low risk
A.355	10.1074/jbc.M500501200	Manabu Ishiki, 2005	Canada	The study focused on the role of phosphatidylinositol (PI) 3-kinase type IA and specific phosphoinositides in the arrival and fusion steps of GLUT4 with the cell membrane. The objective was to simultaneously score the arrival and fusion of GLUT4myc-containing vesicles at the plasma membrane of myotubes, and to determine the effects of insulin, PI(3,4,5)P3, and PI3P in these events	GLUT4myc cells: Mouse muscle cells differentiate into myotubes with stable expression of GLUT4myc fusion protein	1. Insulin stimulation (100 nM).<br><br>2. Wortmannin inhibitor (100 nM) (inhibitor of class IA PI 3-kinase).<br><br>3. LY294002 inhibitor (25 µM) (inhibitor of class IA PI 3-kinase).<br><br>4. PI(3,4,5)P3 signaling molecule (10 µM or 20 µM, which is introduced into the cell via a neomycin carrier).<br><br>5. PI3P signaling molecule (10 µM or 20 µM, which is introduced into the cell via the neomycin carrier). 6. The PI (4,5)P2 signaling molecule (10 µM or 20 µM, is introduced into the cell via the neomycin carrier).<br><br>7. High salt treatment (500 mM NaCl or 650 mM LiCl) to clean the membrane plaque.	1. Confocal Fluorescence Microscopy to quantify the fluorescence intensity of myc epitopes (outer/inner vesicles) and C terminus (cytoplasmic side) on plasma membrane lawns and rounded-up cells.<br><br>2. Immunoelectron Microscopy to directly detect GLUT4-containing vesicles docked on plasma membrane arrays.	1. Insulin (100 nM) is stimulated for 10 minutes.<br><br>2. Wortmannin/LY294002 is pretreated for 20 minutes.<br><br>3. Phosphoinositide Treatment: 10 uM for 20 minutes.	1. Basal (non-stimulated) basal membrane cells/plaques.<br><br>2. Insulin stimulation compared to Basal.<br><br>3. Wortmannin/LY294002 vs. Basal.<br><br>4. Wortmannin/LY294002 + Insulin vs. Insulin alone or Inhibitor alone.<br><br>5. Phosphoinositide + Carrier vs. Single Carrier or Basal.<br><br>6. High salt treatment compared to PBS (Basal).	Research shows that insulin regulates membrane arrival, fusion, and expression of the C-end of GLUT4 through separate phosphoinositides. PI (3,4,5)P3 promotes GLUT4 to the membrane and fuses but does not reveal the C-terminus, whereas PI3P promotes GLUT4 to the membrane and reveals the C-end without fusion. Experiments with wortmannin and LY294002 showed that GLUT4myc existed in unfused docked vesicles, and insulin increased the detection of the C end more than myc, demonstrating the unmasking of the C end.	PI(3,4,5)P3<br><br>PI3P<br><br>PI 3-kinase IA	Stimulation	Insulin regulates GLUT4 transport through three distinct steps that are controlled by different phosphoinositides.<br><br>Three steps of adjustment:<br><br>1. Arrival: Stimulated by Insulin, PI(3,4,5)P3, and PI3P<br><br>2. Fusion: Requires PI(3,4,5)P3 (product of Class IA PI3-kinase)<br><br>3. C-terminus Unmasking: Requires PIP3 (and/or PI 3-kinase C2α type 2 products)	Signaling/fusion/docking										Unclear risk (Not reported)	Unclear risk (Not reported)	Low risk	Unclear risk (Not reported)	Low risk ("scored blindly")	Low risk
A.358	10.1091/mbc.E06-07-0585	Eva Gonzalez, 2006	USA	The goal of the study was to identify specific steps in the GLUT4 trafficking process that are regulated by Akt signals. The study uses acute pharmacological inhibition and shRNA Akt reduction techniques to achieve this goal	3T3-L1 adipocytes	1. Hormone stimulation: Insulin (concentration of 1 nM or 170 nM).<br><br>2. Inhibition:<br>- Selective Akt isozyme inhibitors: AI1/2 (Akti-1/2) (1 µM).<br>- PI3-kinase inhibitor: Wortmannin (100 nM).<br><br>3. Knockdown: Akt1, Akt2, and AS160-specific shRNAs	1. Total Reflectance Fluorescence Microscopy (TIRF Microscopy): Used to measure the redistribution of HA-GLUT4-GFP-containing vesicles within ~250 nm near the cell membrane (PM) (measurement of recruitment/mooring).<br><br>2. Fusion assay by TIRF microscopy: Measure the fusion efficiency of HA-GLUT4 with PM by the ratio of HA-GLUT4 inserted into PM (measured in intact, impermeable cells) to the total amount of HA-GLUT4 in the TIRF region (measured in permeated cells).<br><br>3. Exocytosis/Internalization Kinetic Studies: Using anti-HA epitope (HA.11) antibodies to measure the rate of occurrence of HA-GLUT4 on the cell surface over time.<br><br>4. Hexose Transport Measurement: Glucose absorption is measured by measuring the absorption [3H]2-deoxy-glucose.<br><br>5. Western Blot Immunoblots (Immunoblots): Analysis of phosphorylation states of Akt (Ser473), GSK3β, AS160 and ERK1/2 to confirm inhibitor activity	1. Akt inhibitor (AI1/2) is incubated before 1 hour, or Wortmannin is incubated before 30 minutes, before insulin stimulation.<br><br>2. Insulin stimulation (1 nM) for 30 minutes is used for measurements of GLUT4 to PM displacement, glucose uptake, and fixed-point TIRF assays. Insulin stimulation for 15 minutes is used for Western blot analysis.<br><br>3. Kinetics: Exocytosis and time-lapsed TIRF microscopy experiments were performed for 30 minutes after insulin was added, with data collected continuously (two frames per minute or every 30 seconds).	1. Control (Insulin + Solvent) vs. Insulin + AI1/2 (Inhibition of Akt) or Insulin + Wortmannin (Inhibition of PI3-kinase)<br><br>2. Scrambled shRNA (Control) vs. Akt1 knockdown vs. Akt2 knockdown (All Stimulated Insulin)<br><br>3. Akt2 knockdown + Insulin vs. Akt2 knockdown + Insulin + AI1/2<br><br>4. AS160 knockdown + Insulin vs. AS160 knockdown + Insulin + AI1/2<br><br>5. Shipping of HA-GLUT4-GFP vs. IRAP-TR vs. TR (with/without AI1/2)<br><br>6. Basal State vs. Insulin Stimulation vs. Insulin + AI1/2 vs. Insulin + Wortmannin	Akt is necessary for insulin-stimulated GLUT4 exfollication, but is not the only target because inhibition of Akt still does not completely prevent translocation. Akt activity is required for the pre-fusion step, which involves the recruitment and/or anchoring of GLUT4 vesicles near the plasma membrane (~250 nm). Whereas, the fusion of insulin-stimulated GLUT4 vesicles can occur independently of Akt but depends on the activity of phosphatidylinositol 3'-kinase (PI3-kinase). Therefore, the insulin signal branches to independently regulate both the pre-fusion step and the fusion step, ensuring that GLUT4 is fully distributed into the plasma membrane.	Protein kinase Akt/PKB<br><br>PI3-kinase.	Stimulation	Signaling Divergence: Insulin signals branching to independently regulate the two main steps in GLUT4 exfoliation, in order to achieve maximum GLUT4 translocation, insulin needs to activate both of these pathways.<br><br>- Recruitment/Docking: The path depends on Akt.<br><br>- Fusion: Akt-independent pathway (but PI3-kinase-dependent).	Signaling/fusion/docking										Unclear risk (Not reported)	Unclear risk (Not reported)	Low risk	Unclear risk (Not reported)	Unclear risk (Not reported)	Low risk
A.361	10.1073/pnas.1332633100	Zhen Y. Jiang, 2003	USA	The goal is to analyze insulin signaling via Akt/PKB by gene silencing both the Akt1 and Akt2 isoforms in cultured fat cells, in order to determine their role in the regulation of glucose transport.	3T3-L1 adipocytes	1. siRNA Knockdown: Use synthetic siRNA duplexes to selectively silence Akt1, Akt2, or a combination of both.<br><br>2. Probe protein: Myc-GLUT4-EGFP (used for GLUT4 translocation experiments).<br><br>3. Insulin stimulation (100 nM and 1 nM).	1. Glucose Transport Measurement: 2-deoxyglucose uptake assay after 5 minutes.<br><br>2. GLUT4 (Translocation Assay): Measure the ratio of surface Myc/Total GFP<br><br>3. Western Blotting: Measurement of protein concentrations of Akt1, Akt2, and control proteins (myosin IIB, Arp3D, PKCζ/λ), along with Akt/Akt2 levels of phosphorylation (activation) of Akt (at Thr-308/309) and GSK-3α/β (at Ser-21/9).<br><br>4. Immunofluorescence Microscopy: Visualization of Myc-GLUT4-EGFP translocation on the membrane (nm signal) and silencing of Lamin A/C	1. Gene silence: Cells are analyzed 42 hours (or 48 hours) after siRNA gene transfer.<br><br>2. Insulin stimulation is performed in 15 minutes (to measure Akt phosphorylation) or 30 minutes (to measure glucose and GLUT4 transport)	1. Negative control: Cells are transfected by scrambled siRNA.<br><br>2. Baseline comparison: Basal state vs. insulin-stimulated cell state<br><br>3. Mechanism Comparison: Comparison of the effects of reducing Akt1 alone, Akt2 alone, and Akt1 + Akt2 combined.	Reducing Akt1 alone only slightly reduces insulin-induced hexose transport and does not affect GSK-3 phosphorylation. In contrast, a 70% reduction in Akt2 causes about half of the insulin response. When Akt1 and Akt2 are simultaneously reduced, the effects of insulin on GLUT4 translocation, hexose transport, and GSK-3 phosphorylation are more strongly impaired. These data suggest that Akt2 plays a leading role, but Akt1 and Akt2 have significant functional redundancies, and in particular, Akt kinases are absolutely essential for the regulation of glucose transport and GSK-3 phosphorylation in adipocytes.	Akt2<br>Akt1	Stimulation	This study demonstrates the existence of functional redundancy between Akt1 and Akt2, but Akt2 has a dominant role. Evidence suggests that Akt protein kinases are required for insulin signaling to glucose transport and GSK-3 phosphorylation	Signaling										Unclear risk (Not reported)	Unclear risk (Not reported)	Low risk	Unclear risk (Not reported)	Unclear risk (Not reported)	Low risk
A.365	10.1074/jbc.M109657200	Makoto Kanzaki, 2001	USA	The goal is to explore the functional role of actin polymerization in GLUT4 translocation by reconstructing the formation of actin comet tails on GLUT4 vesicles in vitro and in the broken adipocyte system	3T3-L1 adipocytes	1. Insulin: 100nM for 5 minutes<br><br>2. Stimulates the polymerization of actin in vitro (typically 100 µM GTPγS and 1 mM sodium orthovanadate).<br><br>3. The inhibitor Latrunculin B (20 µM) is used to inhibit the polymerization of actin.<br><br>4. N-WASP/δvca (dominant-interfering N-WASP mutant) Used to inhibit N-WASP function (typically 25 µg/ml as GST fusion protein in in vitro experiments or cDNA expression).<br><br>5. Clostridium difficile Toxin B (0.5 µg/ml) specifically inhibits small GTP-binding proteins of the Rho family	1. Actin-based Motility Assay: Uses rhodamine-actin and GLUT4-EGFP to visualize and observe actin comet tail formation.<br><br>2. GLUT4 Displacement Measurement<br>- Count Positive Cell Rate (%Cells): Count the number of cells that display GLUT4-EGFP fluorescence continuously on the plasma membrane.<br>- Quantify the Myc/EGFP ratio: Compare the Myc label (surface) strength with the EGFP strength.<br><br>3. Confocal Microscopy: To visualize actin comet tails and cortical actin structures.	1. Actin-based Mobility Assay: Uses rhodamine-actin and GLUT4-EGFP to visualize and observe actin comet tail formation.<br><br>2. GLUT4 Displacement Measurement:<br>- Count Positive Cell Rate (% Cells): Count the number of cells that display GLUT4-EGFP fluorescence continuously on the plasma membrane.<br>- Quantify the Myc/EGFP ratio: Compare+Jell0	1. Basal/Untreated: Cells are not treated with Insulin or GTPγS.<br><br>2. Vehicle control: Compared to the group without inhibitors such as latrunculin B or Toxin B.<br><br>3. Vector control (Empty vector): Transgenic co-cell with empty vector (pcDNA3).<br><br>4. N-WASP Wild Type (N-WASP/WT): Cells expressing wild N-WASP	Insulin can cause actin comet tail on GLUT4-containing vesicles, which is necessary for efficient GLUT4 translocation from intracellular storage sites to the plasma membrane. In vitro GLUT4 vesicle-based actin comet tail formation is inhibited by latrunculin B and the dominantly interfering N-WASP mutation (N-WASP/δVCA). Insulin stimulation of the actin comet tail as completely inhibited by Clostridium difficile toxin B, demonstrating the specific involvement of a Rho GTPase	Actin	Stimulation	Actin comet tail formation (stimulated by insulin) is shown to be necessary for effective GLUT4 translocation. This mechanism depends on the N-WASP protein and a small GTP-binding protein of the Rho family (identified as TC10 in the resulting section).	Translocation										Unclear risk (Not reported)	Unclear risk (Not reported)	Low risk	Unclear risk (Not reported)	Unclear risk (Not reported)	Low risk
A.366	10.1152/ajpendo.00466.2011	Vladimir A. Lizunov, 2012	USA and Sweden and Germany	Study the kinetics of GLUT4 vesicles in live skeletal muscle fibers, determining exactly which step in exocytosis (tethering or fusion) is regulated by insulin	Skeletal muscle fibers from transgenic mice express HA-GLUT4-GFP muscle specificity	1. Insulin (for insulin tolerance test - ITT): 0.75 IU/kg for peritoneal injection.<br><br>2. Insulin (stimulates glucose uptake): 2000 uU/ml for 10 minutes for muscle isolation.<br><br>3. Insulin (stimulates GLUT4 translocation in vivo): 0.75 IU/kg peritoneal injection, 10 minutes before sacrifice.<br><br>4. Insulin (stimulated GLUT4 translocation ex vivo): 15-30 minutes incubated in oxidized KRBH solution.	1. Western blot analysis: Analysis of endogenous HA-GLUT4-GFP and GLUT4 protein expression by α-HA and α-GLUT4 antibodies.<br><br>2. Glucose tolerance test (GTT) and insulin tolerance test (ITT): Measure blood glucose levels after glucose or insulin injections.<br><br>3. Glucose uptake in isolated muscles: Measure the uptake of 2-deoxy-[14C] glucose in isolated soleus and EDL muscles.<br><br>4. Total Internal Reflection Fluorescence (TIRF) Microscopy and Confocal Microscopy: GLUT4 translocation, dynamics and fusion event measurements	1. Glucose uptake: 15 minutes incubated with 2-deoxy-[14C] glucose after 10 minutes of insulin stimulation.<br><br>2. ITT: Measure blood glucose at 0, 15, 30, 45, 60, 90, 120 minutes after insulin injection.<br><br>3. GTT: Measure blood glucose at 0, 15, 30, 60, 120 minutes after glucose injection.<br><br>4. GLUT4 translocation in vivo: 10 minutes after insulin injection.<br><br>5. Actin comet tailing/Mobility Assay: Typical recording time is 2-4 minutes	1. Control group: Wild-type mice (WT - Wild-type C57BL/6J) tests on glucose/insulin tolerance and glucose acquisition).<br><br>2. Stimulated group: Cells/muscles are stimulated by insulin or glucose compared to the basal/nonstimulated state.<br><br>3. Bag classification: Stationary bags (stationary/tethered) vs. mobile bags when analyzing membrane compatibility.	In the musculoskeletal transgenic mouse model expressing HA-GLUT4-GFP under the MCK promoter, stimulating insulin redirects HA-GLUT4-GFP to the plasma membrane and T-tubules, accurately simulating endogenous GLUT4. Only about 10% of intracellular GLUT4 vesicles are mobile, while the vast majority remain stationary or tethered in sarcolemma or T-tubules. Most insulin-stimulated exocytosis comes from pretethered vesicles, while cellular pouch pools are less affected. This suggests that the cellular vesicles are not the primary target of insulin, but that the insulin effect mainly targets the pre-anchored GLUT4 vesicles, which are responsible for the initial stage of prosthesis.	insulin	Stimulation	In skeletal muscle, the main effect of insulin is to activate the fusion of GLUT4 vesicles in the sarcolemma and T-tubule regions, rather than stimulating tethering or the recruitment of GLUT4 vesicles from deeper intracellular cavities. Insulin-stimulated GLUT4 release mainly occurs from docked vesicles, which mimic the attachment and conjunction of synaptic vesicles and neuroendocrine cells.	Fusion	Unclear risk (Not reported)	Low risk	Unclear risk (Not reported)	Unclear risk (Not reported)	Unclear risk (Not reported)	Unclear risk (Not reported)	Unclear risk (Not reported)	Low risk	Low risk	Unclear risk (Not reported)	Unclear risk (Not reported)	Low risk	Unclear risk (Not reported)	Unclear risk (Not reported)	Low risk
A.368	10.1128/MCB.01203-13	Dimitrios Kioumourtzoglou, 2014	UK	Using an in-situ PLA neighborhood binding assay to test the hypothesis that insulin stimulates GLUT4 transport by regulating the assembly of the SNARE complex containing Syntaxin4 (Sx4).	3T3-L1 adipocytes	1. Insulin: 100 nM concentration for 5 minutes (for cell experiments).<br><br>2. Munc18c mutation: Use the phosphomimetic variant (simulating the phosphorylation state) Y521E in in vitro tests.<br><br>3. Pre-incubation: Pre-incubation of recombinant protein components (Sx4 with VAMP2 or SNAP23) before complex assembly.	1. In situ Proximity Ligation Assay (PLA): Measure the association/proximity between endogenous proteins (Sx4, SNAP23, VAMP2, Munc18c) in cells.<br><br>2. In vitro SNARE complex assembly assay: Measure the formation of SDS-resistant SNARE complexes by SDS-PAGE electrophoresis and Western blot (Immunoblot) methods.<br><br>3. Pull-down assay: Use Glutathione-Sepharose or IgG-Sepharose granules to examine direct interactions between recombinant proteins (e.g., Sx4 and VAMP2).	1. PLA: After 5 minutes of insulin stimulation.<br><br>2. SNARE complex assembly (in vitro): At 5, 15, 30 minutes (or 5, 20, 60 minutes depending on the experiment).<br><br>3. Sx4-VAMP2 Interaction (Pull-down): 5, 120 minutes or overnight (O/N).	1. Cells: Basal group (Basal - unprocessed) vs. Insulin processed group.<br><br>2. Recombinant proteins:<br>- Wild Munc18c (WT) vs. mutant Munc18c (Y521E).<br>- Wild Sx4 compared to mutant forms of Sx4 (Open-Sx4, ΔN36Sx4, ΔHabcSx4).<br>- Pre-incubation vs. No incubation.	This study demonstrates for the first time that insulin stimulates an increase in the formation of syntaxin4-containing SNARE complexes in fat cells. This process occurs through the mobilization of an inactive amount of syntaxin4 under baseline conditions. In addition, phosphorylation of the regulatory protein Munc18c, a direct target of the insulin receptor, was identified as the molecular switch that coordinates the formation of the SNARE complex, which provides the molecular mechanism of how insulin regulates GLUT4 transport to the plasma membrane.	Syntaxin4 (Sx4)	Stimulation	Insulin activates the tyrosine kinase receptor leading to phosphorylation of Munc18c (at Tyr521). Phosphorylated Munc18c acts as a "molecular switch", freeing Syntaxin4 from its inhibitory binding state to VAMP2 (inactive reserve state), thereby enabling the assembly of functional SNARE complexes (Sx4/SNAP23/VAMP2) to promote GLUT4 membrane fusion.	Fusion										Unclear risk (Not reported)	Unclear risk (Not reported)	Low risk	Unclear risk (Not reported)	Unclear risk (Not reported)	Low risk
A.369	10.1083/jcb.200412069	Vladimir A. Lizunov, 2005	USA and Russia	Observe the kinetic behavior of GLUT4-containing vesicles located near the plasma membrane (PM) in primary fat cells to determine the main mechanism of action of insulin with GLUT4 translocation and to test the hypothesis that insulin is involved in regulating the tethering of these vesicles.	White adipose cells were isolated from the epididymal fat pads of male CD mice.	1. Insulin Stimulation: 67 nM (10 mU/ml)<br><br>2. Transfection: HA-GLUT4-GFP plasmid 8 µg/ml, tubulin-GFP plasmid 0.25 µg/ml<br><br>3. Wortmannin (to verify the signaling mechanism)	1. Internal Total Reflectance Fluorescence Microscopy (TIRFM): To monitor the movement, docking, and fusion of individual GLUT4 vesicles near the plasma membrane.<br><br>2. Confocal Microscopy: To observe the 3D distribution of GLUT4.<br><br>3. Pair-distance correlation analysis: To evaluate the degree of clustering of pockets.<br><br>4. Fluorescence Recovery After Photobleaching (FRAP): To analyze the mobility of GLUT4 on the membrane.	After adding insulin: continuously in real time (time-lapse), and at specific milestones such as 2 minutes, 5 minutes, 10 minutes, 15 minutes.	1. Group 1: Basal state<br><br>2. Group 2: Insulin-stimulated state - Fat cells treated with 67 nM of insulin.<br><br>3. Group 3 (Pharmacological Control): Fat cells treated with wortmannin.	In the basic state, the GLUT4 vesicles move rapidly along the microtubule network near the membrane and are only loosely anchored. In the presence of insulin, this movement is stopped by anchors that hold the vesicles tightly to the membrane, where they form clusters. From here, the membrane vesicles slow down and release GLUT4 in a restricted, non-free-diffuse pattern. In general, insulin converts the cellular GLUT4 vesicles into anchored and membrane vesicles at specialized membrane fusion sites.	insulin	Insulin stimulates the tethering and fusion of GLUT4 vesicles; at the same time, it inhibits (halting) the movement of GLUT4 vesicles near the plasma membrane.	This study on primary fat cells showed that insulin works by "halting" the GLUT4 vesicles that are moving freely near the membrane (constitutive trafficking), prompting them to tightly tethering to specific fusion sites and then binding to the membrane.	Tethering, docking, fusion										Unclear risk (Not reported)	Unclear risk (Not reported)	Low risk	Unclear risk (Not reported)	Unclear risk (Not reported)	Low risk
A.371	10.1091/mbc.e04-04-0333	Anja Zeigerer, 2004	USA	Identify the role of AS160 RabGAP (a substrate of Akt) in insulin-stimulated GLUT4 transport, specifically understanding how insulin signaling travels to the GLUT4 transport apparatus and differentiating its impact on excocellation and cell entry.	3T3-L1 adipocytes	1. Insulin: Stimulates cells with 170 nM insulin.<br><br>2. AS160-4P: Overexpression of the dominant inhibitory mutation form of AS160 (AS160-4P), in which four of the six potential phosphorylation sites of Akt have been mutated into alanines.<br><br>3. myr-Akt1: Expresses myristylated Akt1 (myr-Akt1), a form of continuous activity because it is targeted at the cell membrane.<br><br>4. p110caax: Expresses a continuous active form, targeting the membrane of the PI 3-kinase p110 catalytic subunit.<br><br>5. AS160 (wild type): Excessive expression of wild-type AS160.	1. Single-cell quantitative fluorescence microscopy: Measuring the surface-to-total ratio of HA-GLUT4-GFP using fluorescent anti-HA antibodies (for the surface) and GFP signals (for total amount).<br><br>2. Total Reflectance Fluorescence Microscopy (TIRF Microscopy): To measure the amount of GLUT4 located in the evanescent field of about 250nm close to the cell membrane.<br><br>3. HRP compartment ablation method: Use HRP-transferrin to determine the intracellular distribution of GLUT4 between endosomes and specialized cavities.<br><br>4. Trafficking Assays: Measure efflux rate constant and internalization rate constant by monitoring the accumulation or disappearance of anti-HA antibodies over time.	1. Steady-state: After 15 minutes of insulin stimulation.<br><br>2. Kinetics: Measurements of the rate of cell exocytosis are carried out in a time course lasting up to 45-60 minutes or more to reach the saturation state.	1. Comparison of states: Cells in the basal state vs. cells that are stimulated by insulin.<br><br>2. Comparison of gene intervention: Control cells (expressing GLUT4 only) compared to co-cells expressing mutant proteins (p110caax, myr-Akt1, AS160, AS160-4P).<br><br>3. Comparison of subjects: Transferrin receptor (TR) is used as a control for the general membrane trafficking pathway compared to GLUT4.	Insulin controls blood glucose by increasing the amount of GLUT4 on the surface of muscle and fat cells through a sharp increase in exocytosis and a slight relief of endocytosis. The results showed that AS160 (RabGAP, the target of Akt) is a mandatory factor for insulin to stimulate GLUT4 exocytosis. AS160 inhibitory mutations block insulin in the pre-membrane fusion step but do not affect the effect of insulin in endocyt suppression	AS160, Akt	Akt: Stimulation<br>AS160: Inhibition	Insulin signals the exocytosis apparatus along the AS160-dependent pathway, while the effect on GLUT4 internalization takes place via the AS160-independent pathway.	signaling										Unclear risk (Not reported)	Unclear risk (Not reported)	Low risk	Unclear risk (Not reported)	Unclear risk (Not reported)	Low risk
A.372	10.1083/jcb.135.2.415	Weichen Wang, 1996	USA	Determine the subcellular distribution of Glut1 and Glut4 in skeletal muscle, the basal and insulin-stimulated glucose transport sites, and examine the effects of insulin on the T-tubule structure and the epitope expression of Glut4.	Skeletal muscles of transgenic mice overexpress Glut1 or Glut4	1. Insulin: Used in three main protocols:<br>- In vivo peritoneal administration: Inject 6 units of insulin with glucose 30 minutes before fixation.<br>- Euglycemic, hyperinsulinemic clamp: Infusion of 20 mU/kg/minute of insulin for at least 60 minutes.<br>- In Vitro Muscle Incubation: Use 10 mU/ml of insulin for 20 minutes or 30 minutes of incubation.<br><br>2. Glycerol Shock: Selectively disrupt the T-tubule by incubating the muscle in a Ringer's buffer containing 400 mM of glycerol (60 minutes) and then switching to a non-glycerol buffer (30 minutes).<br><br>3. T-tubule Recovery: Restore the T-tubule morphology by returning the shocked muscle sample to Ringer's buffer containing glycerol for 40 minutes.	1. Immunoelectron microscopy (Immunogold labeling): Specific primary antibodies (F349 for Glut4, F350 for Glut1) and colloidal gold binding secondary antibodies are used on ultra-thin slices embedded in Lowicryl HM20.<br><br>2. Laser confocal immunofluorescence microscopy: Using fluorescent binding antibodies (F349 and WUI26) on frozen slices.<br><br>3. Western blot: Quantitative analysis of total Glut4 protein.<br><br>4. Glucose transport assay: Measures the absorption of 3-O-methylglucose.	1. Immunogold/Confocal: After 30 minutes (peritoneal injection/in vitro incubation) or 90 minutes (clamp) insulin stimulation.<br><br>2. Glucose transport: After 20 minutes of incubation with 3-O-methylglucose.	1. Basal state vs insulin treatment according to protocols<br><br>2. Control muscle vs muscle with glycogen shock vs muscle when glycogen shock then recover	These data show that Glut1 and Glut4 are localized in different plasma membrane regions in skeletal muscle. Glut1 is responsible for basal glucose transport at sarcolemma, while the majority of glucose transport is stimulated by insulin is carried out by Glut4 in the T-tubules. In addition, insulin increases the surface area of T-tubules in skeletal muscle and causes epitope expression at the COOH end in skeletal muscle, similar to that in mouse fat cells.	GLUT4, GLUT1	Stimulation	The mechanism of glucose transport stimulated by insulin in skeletal muscle primarily occurs via Glut4 localized at the T-tubules, and insulin works through increasing the surface area of the T-tubules and unmasking the hidden epitope at the COOH end of Glut4, similar to that in fat cells. Glut1 is responsible for the basic transport at sarcolemma.	Translocation	Unclear risk (Not reported)	Low risk	Unclear risk (Not reported)	Unclear risk (Not reported)	Unclear risk (Not reported)	Unclear risk (Not reported)	Unclear risk (Not reported)	Unclear risk (Not reported)	Unclear risk (Not reported)	Unclear risk (Not reported)	Unclear risk (Not reported)	Low risk	Unclear risk (Not reported)	Unclear risk (Not reported)	Unclear risk (Not reported)

A.373	10.1016/S0021-9258(18)53472-X	Garret J. Etgen, Jr., 1993	USA	Investigate the relationship between low-molecular-weight GTP-binding proteins and the translocation of GLUT4 proteins in rat skeletal muscle by examining their subcellular distribution under baseline, insulin-stimulated and muscle-contracting conditions.	Lean Zucker rats, 14 weeks old.	Insulin: Insulin perfusion at a concentration of 10 mU/ml for 7 minutes.<br><br>Contraction: Stimulation of the sciatic nerve with a pulse sequence of 100 Hz, 8-12 V, each pulse lasting 0.1 ms, speed of 1 pulse/second in two 5-minute intervals (1 minute rest between two phases).	1. Glucose absorption measurement: Using the radioisotopes 2-deoxy-D-[1-3H] glucose (2-DG) and [1-14C]mannitol.<br><br>2. Measurement of GLUT4 protein: Western blotting technique with anti-GLUT4 (F349) and 125I-labeled antibodies on plasma membrane and low-density microsomal (LDM) segments.<br><br>3. Measurement of GTP-binding proteins: GTP [α-32P]GTP bonding technique on nitrocellulose membranes after SDS-PAGE electrophoresis.	Insulin group: After 7 minutes of insulin infusion.<br><br>Muscle contraction group: After a total of about 10 minutes of muscle contraction stimulation and 7 minutes of infusion (muscle samples are taken immediately afterwards).	Basal group: No insulin, no muscle contraction.<br><br>Insulin group: Stimulated by insulin.<br><br>Contraction: Stimulated by electromechanical contraction.	Insulin and muscle contraction both increase the amount of GTP-binding proteins and GLUT4 proteins at the plasma membrane, suggesting a functional relationship between them. However, insulin reduces their amount at the low-density microsome, while muscle contraction does not change.	Low molecular weight GTP-binding proteins	Stimulation: insulin and muscle contraction both stimulate an increase in GLUT4 and GTP-binding proteins at the membrane	The study found that insulin and muscle contraction mobilize GLUT4 and GTP-binding proteins from separate sites. Specifically, insulin is mobilized from the LDM segment, while muscle contraction is mobilized from a source other than LDM	Translocation	Low risk ("randomly assigned")	Low risk	Unclear risk (Not reported)	Unclear risk (Not reported)	Unclear risk (Not reported)	Unclear risk (Not reported)	Unclear risk (Not reported)	Unclear risk (Not reported)	Unclear risk (Not reported)							
A.375	10.1021/pr0502626	Leonard J. Foster, 2006	Denmark, USA, Germany	Application of stable isotope marking with amino acids in cell culture (SILAC) to identify proteins that interact with GLUT4 in an insulin-regulated manner.	The L6 series myotubes stably express the GLUT4myc protein.	1. Insulin stimulation: 100 nM insulin for 10 minutes (for SILAC experiment), 20 minutes (for Immunoblotting), or 5 minutes (for fluorescence microscopy).<br><br>2. Isotope Marker (SILAC): Cultured in a medium containing stable isotope leucine (L-Leucine-5,5,5-D3,LeuD3) or normal leucine (LeuD0).<br><br>3. Latrunculin B: Treated with a concentration of 250 nM for 20 minutes before adding insulin to break down cytoskeleton actin (in a controlled experiment).	1. SILAC combines LC-MS/MS: Liquid chromatography/tandem mass spectrometry to quantify and identify interacting proteins.<br><br>2. Immunoprecipitation: Use anti-myc antibodies to pull GLUT4myc and binding proteins.<br><br>3. Immunoblotting (Western Blot): To verify the interaction of GAPDH and a-actinin-4 with GLUT4.<br><br>4. Laser scanning confocal immunofluorescence microscopy: To observe the co-localization of GLUT4, a-actinin-4 and actin.	1. After 10 minutes of insulin stimulation (for SILAC mass spectrometry analysis).<br><br>2. After 20 minutes of insulin stimulation (for Western Blot analysis).<br><br>3. After 5 minutes of insulin stimulation (for microscopic analysis).	1. Basal/Unstimulated Cells cultured with LeuD0 vs. Insulin Stimulated Cells cultured with LeuD3.<br><br>2. L6-GLUT4myc cells compared to wild-type L6 cells do not express GLUT4myc (negative control).<br><br>3. Latrunculin B processing cells compared to non-vehicle cells.	Of the 603 proteins dosed, 36 altered proteins interacted with GLUT4myc by insulin by more than 1.5 times. Cytoskeleton proteins increase in the presence of insulin (especially α-actinin-4), while components of the ubiquitin-proteasome system decrease, and the proteins involved in vesicular transport also change their insulin-response interactions. As such, insulin alters GLUT4's interaction network with many proteins, especially cytoskeletons, to aid in the transport of GLUT4 to the plasma membrane.	GLUT4<br><br>Framework proteins, ubiquitine degrading system proteins, metabolic enzymes	Insulin stimulates the interaction of GLUT4 with cytoskeletal proteins and metabolic enzymes.<br><br>Insulin inhibits the interaction of GLUT4 with proteins belonging to the ubiquitin-proteasome degradation system.	Insulin regulates the protein-protein interaction of GLUT4, recruiting cytoskeleton proteins, particularly α-actinin-4, which bind to the transporter to facilitate the recruitment of GLUT4 to the plasma membrane. It also affects ubiquitine proteins, metabolic enzymes.	Translocation										Unclear risk (Not reported)	Unclear risk (Not reported)	Low risk	Unclear risk (Not reported)	Unclear risk (Not reported)	Low risk	
A.380	10.24546/81000139	Kazuo Takazawa, 2008	Japan	Determine whether the conventional kinesin motor system, specifically the kinesin 2 light chain (KLC2), plays a regulatory role in insulin-stimulated GLUT4 translocation in C2C12 myoblasts, similar to the proposed role in fat cells.	1. Myoblasts of the C2C12 line<br><br>2. Other: CHO-IR cells (yellow guinea pig ovarian cells overexpressing insulin receptors), 293 cells (human embryonic kidney cells)	1. Insulin: 100 nM for 30 minutes.<br><br>2. Rabies KLC2 gene expression and mutations: KLC2-WT, KLC2-S575A, or KLC2-ΔTPR.<br><br>3. Other stimulants (to test KLC2/14-3-3 binding): AICAR (500 μM), Forskolin (50 μM), Isoproterenol (10 μM), A23187 (1 μM).	1. Immunoprecipitation and Immunoblot analysis (Western blot analysis): Determine the interaction between proteins (KHC, KLC2, 14-3-3) and the level of protein expression.<br><br>2. GST-pull down assay: Check the alignment between KLC2 and 14-3-3 in vitro.<br><br>3. Immunofluorescence: Detection of externalized GLUT4 by anti-Myc antibodies on non-membrane cells.<br><br>4. Confocal laser scanning microscopy: Observe the displacement of GLUT4-GFP to the periphery of the cell.	1. GLUT4 translocation analysis: After 30 minutes of insulin stimulation.<br><br>2. Protein bond analysis: After 30 minutes of treatment with stimulants (insulin, forskolin...).<br><br>3. Protein expression: 24 hours after adenovirus infection or 48 hours after plasmid infection transfer.	1. Basal cells vs. insulin-stimulated cells.<br><br>2. LacZ-encoded adenovirus infected cells (control) compared to KLC2-WT, KLC2-S575A, or KLC2-ΔTPR infected cells.	In C2C12 myoblasts, overexpression of KLC2-WT, KLC2-S575A, or KLC2-ΔTPR does not significantly affect the translocation of GLUT4 to the cell membrane and insulin-stimulated outward expression, suggesting that conventional kinesin is not necessary for insulin-induced GLUT4 translocation in cultured myoblasts. In addition, the study confirms that the cAMP-PKA pathway can induce KLC2 phosphorylation and binding to 14-3-3 in muscle cells, but this mechanism is not involved in insulin-stimulated GLUT4 transport here.	Research shows that conventional kinesin is not necessary for insulin-induced GLUT4 translocation in cultured myoblasts, while it usually plays an important role in fat cells	None	Research shows that conventional kinesin is not necessary for insulin-induced GLUT4 translocation in cultured myoblasts, while it usually plays an important role in fat cells	Translocation										Unclear risk (Not reported)	Unclear risk (Not reported)	Low risk	Unclear risk (Not reported)	Unclear risk (Not reported)	Low risk	
A.381	10.1128/MCB.23.14.4892-4900.2003	Takeshi Imamura, 2003	USA	Investigate the role of Rab4 (a small GTP-binding protein) and KIF3 motor protein (kinesin II in mice) in insulin-stimulated GLUT4 release in 3T3-L1 fat cells.	3T3-L1 adipocytes	1. Insulin: Stimulation with a concentration of 17 nM or 100 nM for a period of 10 to 30 minutes.<br><br>2. Chemical Inhibitors:<br>- LY294002: Inhibits PI3-kinase (50 μM).<br>- PD98059: Inhibits MEK (30 μM).<br>- Myr-PKC-λ pseudosubstrate: PKC-λ (50 μM) inhibitor peptide.<br><br>3. Transfection/Adenovirus infection: N121I-Rab4, WT-Rab4, DN-PKC-λ, CA-PKC-λ<br><br>4. Microinjection:<br>- Anti-KIF3B, anti-KAP3A antibodies<br>- siRNA (KAP3A, insulin receptor)	1. Photoaffinity labeling: Use [γ-32P]GTP-azidoanilide to measure the activity level (GTP loading) of Rab4.<br><br>2. GLUT4 Translocation Assay (Ring assay): Use fluorescence microscopy to evaluate the translocation of GLUT4-eGFP or endogenous cell membrane GLUT4 (forming a ring of light).<br><br>3. Microtubule capture assay: Measure the binding ability of KIF3 to microtubules in vitro.<br><br>4. Co-immunoprecipitation (Co-IP): Determines the physical interactions between proteins (Rab4-KIF3, Rab4-PKC-λ).<br><br>5. Microinjection & Immunofluorescence: Evaluation of the effect of antibodies or siRNA on GLUT4 translocation.	1. Rab4 activation: After 10 minutes of insulin stimulation.<br><br>2. GLUT4 translocation: After 20 minutes of insulin stimulation.<br><br>3. KIF3 activation: After 10 minutes of insulin stimulation.<br><br>4. Co-IP: Moments 0, 5, 10, 15, 20, 30 minutes after stimulation.	1. Basal group: No insulin stimulation.<br><br>2. Control group: Transfection with empty vector (Mock), Control IgG injection, Scrambled siRNA injection.<br><br>3. Intervention groups: Groups treated with inhibitors, spike protein expression, or specific antibody/siRNA injections.	Combined results show that insulin activates Rab4, increases Rab4–kinesin KIF3 binding, and promotes KIF3 interaction with microtubules, thereby supporting GLUT4 vesicle transport. This entire process depends on the insulin-activated PI3K-dependent PKC-λ and is an important step in promoting GLUT4 exocytosis in 3T3-L1 fat cells.	Rab4, KIF3	Stimulation	Insulin via the PI3-kinase/PKC-λ signaling pathway, activates Rab4 and KIF3. Activated Rab4 (GTP-bound) binds directly to the KIF3 motor protein (kinesin II). This functional interaction allows KIF3 to transport GLUT4-containing vesicles along microtubules to the cell membrane (exocellular).	Signaling, translocation										Unclear risk (Not reported)	Unclear risk (Not reported)	Low risk	Unclear risk (Not reported)	Unclear risk (Not reported)	Low risk	
A.382	10.1042/bj3010177	Ingo Uphues, 1994	Germany and France	Unravels the possible functional relationship between small-molecular-mass G-proteins and the insulin-responsive glucose transporter GLUT4 in rat heart muscle.	Lean male Zucker rats	1. Insulin: Tail-vein injection at a dosage of 4 units/100g of body weight, 20 minutes before taking the heart.<br><br>2. Immunoadsorption: Anti-GLUT4 antibodies are used to isolate GLUT4-enriched membrane vesicles.	1. Ligand blotting with [α-32P]GTP: For the detection and quantification of GTP-binding proteins on the nitrocellulose membrane after SDS/PAGE electrophoresis.<br><br>2. Immunoblotting (Western blot): Use specific antibodies (anti-GLUT4, anti-ral/1A, anti-rab2, anti-rab4A, anti-rab5 and 125I-Protein A to determine the presence and distribution of proteins.<br><br>3. Bio-imaging analysis: Using the FUJIX BAS 1000 system to quantify radioactive signals with high sensitivity.	1. Insulin stimulation (In vivo): The heart is removed 20 minutes after insulin injection.<br><br>2. Protein A incubation: Western blot filters are incubated with 125I-Protein A for 2 hours.<br><br>3. Antibody incubation (Antiserum): The filters are incubated with anti-GLUT4 or anti-rab serum for 16 hours.<br><br>4. GTP Binding Brewing: Incubation with [α-32P]GTP takes place in 30 minutes.	1. Hormone state: insulin-stimulated rats (insulin-stimulated rats) compared to basal rats.<br><br>2. GTP binding control: use [α-32P] GTP vs. [α-32P]GTP+GTP[S]<br><br>3. Cell Segmentation (for Distribution Test): Plasma Membrane (PM), Microsomal Membrane (MM), and Cytosol (C)	In rat heart tissue, two GTP-binding G-proteins of 24 kDa and 26 kDa were detected in both plasmacyte and microsomal membranes. When the GLUT4-rich vesicle was separated, only the 24 kDa protein remained, suggesting that it binds specifically to GLUT4 vesicles. In vivo insulin does not alter the total amount of G-protein in microsomes, but reduces the 24 kDa protein in GLUT4-vesicles by about 36%. This suggests that the small G protein 24 kDa detaches from the vesicle when GLUT4 is mobilized to the membrane and has a functional role in this process.	Small-molecular-mass GTP-binding proteins	Stimulation	A small unidentified G protein (24 kDa) attaches to the GLUT4 reservoir and keeps GLUT4 in an intracellular state, insulin separates this protein from the GLUT4 vesicles, thereby releasing GLUT4 for transfer to the plasma membrane.<br><br>In contrast, Rab4A that exits the membrane with GLUT4 (in the presence of insulin) can regulate GLUT4 recycling back to the surface, thereby keeping GLUT4 concentrations on the membrane high.	Translocation	Unclear risk (Not reported)	Unclear risk (Not reported)	Unclear risk (Not reported)	Unclear risk (Not reported)	Unclear risk (Not reported)	Unclear risk (Not reported)	Unclear risk (Not reported)	Unclear risk (Not reported)	Unclear risk (Not reported)							
A.384	10.1074/jbc.M007610200	Ann Louise Olson, 2001	USA	Determine whether the exocytosis of GLUT4 depends on an intact microtubule network.	3T3-L1 adipocytes	1. Insulin: Handle with a concentration of 100 nM or 175 nM for a period of 20 to 30 minutes.<br><br>2. Nocodazole: Treated with a concentration of 33 μM for a period of 20 minutes to 3 hours to destroy (depolymerize) microtubules.<br><br>3. Taxol (Paclitaxel): Treated with a concentration of 12 μM for 3 hours to stabilize (polymerize) microtubules.	1. Plasma Membrane Sheet Assay: Analyze the concentration of GLUT4, GLUT1 on the plasma membrane by Western blot.<br><br>2. 2-Deoxyglucose Uptake: Measures glucose transport activity.<br><br>3. Adipsin Secretion: Measures adipsin secretion (as a control for other exfoliation).<br><br>4. In Vitro Microtubule Binding Assay: Evaluate the binding ability of GLUT4 vesicles and signaling proteins (such as IRS-1) to polymerized microtubules.<br><br>5. Western Blotting: Assesses the degree of phosphorylation of signaling proteins (IRS-1, PKB/Akt) and the presence of other proteins.	1. Nocodazole/Taxol treatment: 3 hours (main time).<br><br>2. Insulin Stimulation:<br>- 20 minutes (for GLUT4/GLUT1 translocation and 2-deoxyglucose absorption).<br>- 30 minutes (for PI 3-Kinase, Phospho-Tyrosine, and Adipsin secretion).<br><br>3. Test Nocodazole time: 0, 20, or 120 minutes before insulin stimulation to measure GLUT4 translocation.	1. GLUT4 Translocation and Glucose Absorption Experiment:<br>- Control group: 3T3-L1 cells with/without insulin.<br>- Microtubule Destruction Group (Nocodazole): 3T3-L1 cells process 33 μM Nocodazole with/without insulin.<br>- Microtubule Stability Group (Taxol): 3T3-L1 cells process 12 μM Taxol with/without insulin.<br><br>2. Experiment Specificity (Specific pathways):<br>- GLUT1 translocation: Comparison of GLUT1 accumulation on cell membranes between the control group and the Nocodazole group (in the presence of insulin).<br>- Adipsin secretion: Comparison of adipsin secretion between the control group and the Nocodazole group (in the presence of insulin).<br><br>3. Insulin Signaling Experiment: Comparison of signaling activities (IRS-1/receptor phosphorylation, PI 3-kinase activity, PKB activation) between the control group and the Nocodazole group (in the presence of insulin).<br><br>4. In Vitro Bonding Experiment: Comparison of the binding of proteins (GLUT4, IRAP, IRS-1, insulin receptor β-subunit) with polymerized tubulin (in the presence of Taxol).	Nocodazole destroys the microtubule by more than 80% of insulin delivery of GLUT4 to the membrane. However, the early signaling steps of insulin (IRS-1, PI3K, Akt) are still functioning normally. This suggests that the microtubule is not needed for the initial insulin signal, but is specifically needed for GLUT4 transport. Nocodazole does not affect GLUT1 or adipsin, demonstrating specificity. The GLUT4 and IRS-1 vesicles can both attach to the microtubule, suggesting that the microtubule helps direct the GLUT4 vesicle to the membrane	Microtubes	Stimulation	The microtubule is the key structure that helps insulin deliver GLUT4 to the membrane, both as a support and as a rail for the vesicle to move. The fact that GLUT4-vesicle and IRS-1 adhere directly to the microtubule suggests that the microtubule is the intersection between the insulin signal and the pouch delivery system.	Translocation										Unclear risk (Not reported)	Unclear risk (Not reported)	Low risk	Unclear risk (Not reported)	Unclear risk (Not reported)	Low risk	
A.385	10.1074/jbc.271.17.10200	Robin A. Heller-Harrison, 1996	USA	Test the hypothesis that insulin directs the binding of PI 3-kinase (via the IRS-1-PI 3-kinase complex) to intracellular GLUT4-containing vesicles, which in turn play a role in the migration of GLUT4 to the plasma membrane.	1. 3T3-L1 adipocytes<br><br>2. Extra: rat adipocytes (Rat adipocytes)	1. Insulin: Stimulates cells with a concentration of 100 nM at different intervals (from 0.5 to 15 minutes, typically 3.5 minutes).<br><br>2. Wortmannin: Use a concentration of 100 nM to inhibit PI 3-kinase activity.<br><br>3. Competitive peptides: Use the COOH terminal peptide of GLUT4 (1 mM) to test the specificity of the immunoadsorption process.	1. Immunoadsorption: Anti-GLUT4 antibodies are used to isolate GLUT4-containing vesicles from low-density microsomals (LDMs).<br><br>2. Sucrose Velocity Gradient Centrifugation: Isolation of GLUT4-containing vesicles from mouse fat cells based on sedimentation rate.<br><br>3. PI 3-Kinase Assay: Measured PI 3-kinase enzyme activity in immunoprecipitates (anti-IRS-1 or anti-p85) or directly on GLUT4-containing vesicles, using the substrates PI, PI 4-P, and PI 4,5-P2.<br><br>4. HPLC (High Performance Liquid Chromatography): Analysis of phosphoinositide products (PI 3-P, PI 3,4-P2, PI 3,4,5-P3).<br><br>5. Immunoblotting (Western Blot): Determines the presence and amount of GLUT4, p85 (a subunit of PI 3-kinase) in membrane segments.	1. Time-course: 0, 0.5, 1, 2, 5, 15 minutes after insulin stimulation to monitor GLUT4 translocation.<br><br>2. PI 3-Kinase activity: After 3.5 minutes of insulin stimulation (the optimal time for PI 3-kinase activity).	1. Comparison of Insulin (Basal vs. Stimulated): Non-Insulin Group (Basal) vs. 100 nM Insulin Stimulation Group (3.5 min).<br><br>2. Peptide Inhibition Specificity: comparison between the group without the inhibitor peptide and the group with the GLUT4 COOH-terminal peptide (1 mM).<br><br>3. Comparison of enzyme specificity (Wortmannin Inhibition): comparison between the group without the inhibitor and the group with 100 nM Wortmannin.	In the insulin response, the IRS-1–PI3K complex is detected directly on the vesicles containing GLUT4. Insulin increases ~2.3-fold p85 and PI3K activity on these vesicles; HPLC shows in situ production of PI3P, $PI(3,4)P_2$, and $PI(3,4,5)P_3$, demonstrating insulin recruitment and activation of PI3K right on the GLUT4 vesicle.	PI 3-kinase, IRS-1	Stimulation	Insulin causes IRS-1 to bind and activate PI3K, and then the IRS-1–PI3K complex is recruited to the correct intracellular GLUT4-containing vesicles. Here, PI3K produces local phosphoinositides, which act as signals to promote the transport of GLUT4 vesicles to the plasma membrane.	Signaling										Unclear risk (Not reported)	Unclear risk (Not reported)	Low risk	Unclear risk (Not reported)	Unclear risk (Not reported)	Low risk	
A.386	10.1073/pnas.0901933106	Eva Gonzalez and Timothy E. McGraw, 2009.	USA	Study of the mechanisms by which the signaling specificity of Akt isoforms (especially Akt2) in the regulation of insulin-induced glucose transport in adipocytes (adipocytes).	3T3-L1 adipocytes	1. Insulin: Stimulates cells at a concentration of 10 nM or 100 nM.<br><br>2. Gene expression (Transfection/Electroporation)<br><br>3. siRNA: Knockdown Akt1, Akt2 or both.<br><br>4. Inhibitors: Wortmannin (PI3K inhibitors).	1. TIRF Microscopy: Measures the accumulation of Akt at the plasma membrane (PM) in living and fixed cells.<br><br>2. Immunoblotting (Western Blot): Measures the phosphorylation level of Akt (pT308/9, pS473/4), GSK3β, AS160 and protein expression levels.<br><br>3. In vitro Akt kinase assay: Measure the kinase activity of Akt using the substrate peptide GSK3β.<br><br>4. GLUT4 translocation assay: Measure the Surface-to-Total GLUT4 distribution of GLUT4 using the HA-GLUT4-GFP reporter.<br><br>5. Quantitative microscopy (Permeabilized cell assay): Measure the amount of membrane-bound AS160 in the membrane permeable cell.	1. Signaling (Phosphorylation/Activity): 0, 5, 15, 30 minutes after insulin stimulation.<br><br>2. TIRF Imaging: Real-time tracking (time-lapse) or at fixed times (e.g., 30-35 minutes).<br><br>3. GLUT4 Translocation: After 15-30 minutes of insulin stimulation.	1. Insulin: Basal vs. insulin stimulation.<br><br>2. Akt Isoforms: Comparison between Akt1 and Akt2 (and their variants/chimera).<br><br>3. Knockdown: Control siRNA vs Akt1 siRNA vs Akt2 siRNA.<br><br>4. Inhibitory comparison: Wortmannin-treated cells vs. non-processed cells	Insulin activates both Akt1 and Akt2 in fat cells, but they are distributed differently under the cell. Akt2 is recruited to the stronger plasma membrane, which is consistent with the specific role of Akt2 in the control of GLUT4 transport. Akt2 also phosphorylates AS160 and attaches AS160 to the membrane, which helps control GLUT4. Shows that insulin-generated subcellular partition determines the individual functional determinants of each Akt isoform.	Akt1 and Akt 2, AS160	Akt1, Akt 2: stimulation<br><br>AS160: inhibitor	Subcellular partitioning due to insulin: Insulin causes Akt2 to accumulate predominantly at the plasma membrane compared to Akt1. This local accumulation allows Akt2 to better access and phosphorylate the specific substrate AS160 at the membrane, resulting in the release of AS160 from the membrane and allowing GLUT4 to transpose	Signaling										Unclear risk (Not reported)	Unclear risk (Not reported)	Low risk	Unclear risk (Not reported)	Unclear risk (Not reported)	Low risk	
A.387	10.1091/mbc.E20-06-0356	Alexandria Brumfield, 2021	USA and Germany	This study aims to determine whether insulin regulates the mobilization of GLUT4 from the perinucular cavity and what molecular mechanisms control this process, namely the role of the TBC1D4-RAB10 signaling module	3T3-L1 adipocytes	1. Insulin: Stimulated with a concentration of 1 nM or 10 nM for a period of 10 to 30 minutes.<br><br>2. Copper/CuCl2: Stimulated at a concentration of 200 μM.<br><br>3. BCS (Bathocuproinedisulfonic acid disodium salt): Treated with a concentration of 200 μM to create low copper conditions.<br><br>4. Nocodazole: Treated with a concentration of 3 μM to destroy microtubules.<br><br>5. MK2206: AKT inhibitor (1 μM).<br><br>6. Knockdown (KD)/siRNA<br><br>7. Gene expression	SILAC Mass Spectrometry (Proteomics): Quantitative comparison of co-immunoabsorbed proteins with GLUT4 variants (F5Y vs. WT/F5A) to determine the protein composition of the GLUT4 compartment.<br><br>Photoconversion & Live-cell Imaging: Tracking the mobilization kinetics of GLUT4 from the perinuclear region using mEos3.2-tagged GLUT4 (green-to-red photoconversion).<br><br>Immunofluorescence (IF) & Confocal Microscopy (Airyscan): Observing the colocalization of proteins (GLUT4, STX6, TGN46, ATP7A, RAB10, SEC16A) and organelle structures.<br><br>Western Blotting: Confirming the presence of proteins in immunoabsorbed samples.<br><br>Quantitative RT-PCR: Confirming the knockdown efficiency of siRNA.	1. Photoconversion Assay: Monitor for 20 minutes after color gradation (1 shot every 2 minutes).<br><br>2. IF/Localization: After 30 minutes of insulin stimulation or 2 hours of copper stimulation.<br><br>3. Proteomics: Sample collection after detergent-free cell lysis.	1. WT vs F5Y vs F5A GLUT4: Comparison of binding proteins to determine cavity characterization.<br><br>2. Basal vs Insulin: Comparison of GLUT4 mobilization rate and protein site.<br><br>3. Control vs Knockdown (TBC1D4, RAB10, SEC16A, KIF13): Functional role evaluation.<br><br>4. Basal vs Copper (BCS/CuCl2): Comparison of ATP7A mobilization.<br><br>4. Control vs Nocodazole: Evaluation of the role of microtubules and spatial structure.	TBC1D4–RAB10 signaling controls the release of GLUT4 from the reservoir in TGN, suggesting that insulin affects both intracellular depths and not just membranes. The fact that insulin does not mobilize ATP7A and copper does not mobilize GLUT4, along with the fact that RAB10 does not need to mobilize ATP7A, demonstrates that insulin exploits a TGN region that is shared for many commodities, but selectively mobilizes GLUT4 from there.	RAB10, TBC1D4 (AS160)	RAB10: stimulation<br><br>AS160: inhibitor	GLUT4 is stored in a dedicated TGN area that handles air-conditioned transport goods, not just GLUT4. Insulin activates the TBC1D4–RAB10 axis to release GLUT4 vesicles from this TGN region (RAB10 is anchored here thanks to SEC16A). The mobilization of GLUT4 from TGN through RAB10 takes place without the need for microtubes.	Translocation										Unclear risk (Not reported)	Unclear risk (Not reported)	Low risk	Unclear risk (Not reported)	Unclear risk (Not reported)	Low risk	
A.388	10.1002/elps.1150181419	Michelle M. Hill, 1997	Australia	To study the subcellular distribution of insulin-regulated phosphoproteins in adipocytes, specifically to test the hypothesis that insulin signaling mediators are located in close proximity to the intracellular GLUT4 reservoir	3T3-L1 adipocytes	1. Insulin Stimulation: Incubate the cells with 1uM of insulin for 15 minutes.<br><br>2. Radioactive marking: Annealed with 32P (orthophosphate).<br><br>3. Detergent Treatment: Treat membrane segments with 1% Triton X-100.	1. Subcellular fractionation: Separation of plasma membrane (PM), cytosol, and high-speed residue (HSP - containing intracellular GLUT4).<br><br>2. Bidirectional gel electrophoresis (2-DE): Protein separation based on isoelectric point (IEF) and molecular mass (SDS-PAGE).<br><br>3. Autoradiography: Detection of phosphorylated (32P-labeled) proteins.<br><br>4. Western blotting: Detect GLUT4 and Caveolin.	15 minutes after insulin stimulation.	1. Basal vs. Insulin: Comparison between unprocessed and insulin-stimulated cells.<br><br>2. Segments: Comparison between Plasma membrane (PM), Cytosol and High-speed pellet (HSP).<br><br>3. Solubility: Comparison between soluble and insoluble parts in the Triton X-100.	Many phosphoproteins in fat cells are concentrated in the HSP segment containing intracellular GLUT4. Insulin stimulates the phosphorylation of 12 phosphoproteins in this region. The majority of the insoluble HSP phosphoproteins in Triton X-100, unlike GLUT4 or caveolin, suggest that they organize into insoluble microdomains/complexes, independent of caveolin.	The phosphoproteins are regulated by insulin	Insulin stimulates the phosphorylation of proteins and GLUT4 translocation	Insulin organizes the phosphoproteins in HSPs into insoluble complexes, independent of caveolin, which can act as scaffolds that transmit signals to GLUT4.	Signaling										Unclear risk (Not reported)	Unclear risk (Not reported)	Low risk	Unclear risk (Not reported)	Unclear risk (Not reported)	Low risk	

A.389	10.1091/mbc.E10-02-0158	Ingrid Jordens, 2010	USA	To investigate the role of IRAP in GLUT4 transport, specifically to examine whether IRAP regulates the retention and sorting of GLUT4 from the endosome to specialized cavities.	3T3-L1 adipocytes	1. Insulin: Stimulate cells with a concentration of 1 nM for 30 minutes to reach a steady state.<br><br>2. IRAP Knockdown (KD): Use stable shRNA or siRNA to reduce IRAP expression.<br><br>3. GLUT4 Knockdown (KD): Use stable shRNA to reduce GLUT4 expression.<br><br>4. AS160 Knockdown (KD): Use siRNA to reduce AS160 expression.<br><br>5. Gene expression (Re-expression/Overexpression):<br>- IRAP (full-length).<br>- IRAP-TR (chimeric protein: IRAP cytoplasmic domain fused to Transferrin Receptor).<br>- AS160-4P (dominant-interfering mutant).<br>- Mutations of GLUT4 (F5A, E488A,E490A).	1. Translocation Assay (Surface-to-Total Ratio): Measure the ratio of GLUT4 or IRAP on the cell surface to total using single-cell immunofluorescence.<br><br>2. Kinetic Assays (Exocytosis/Internalization): Measures the exocytosis and intracellular rate constants of GLUT4.<br><br>3. Ablation Assay (Compartment ablation): Use HRP-transferrin and DAB to determine the distribution of GLUT4 in recycling endosomes.<br><br>4. Permeabilized Cell Assay: Measures the amount of membrane bond AS160.<br><br>5. Immunoadsorption: Isolation of GLUT4-containing cavities to test binding to AS160.	1. Translocation: After 30 minutes of insulin stimulation.<br><br>2. Kinetic assays: Monitor for long periods of time (up to 420 minutes for exocytosis, 30 minutes for internalization).<br><br>3. Ablation assay: After 3-4 hours annealing with Tf-HRP.	1. Basic function: Control adipocytes vs. IRAP-KD cells (IRAP knockdown).<br><br>2. Functional salvage: IRAP-KD cells compared to IRAP-KD cells are reexpressed full IRAP or IRAP-TR (a hybrid protein structure with only the cytoplasmic domain name of IRAP).<br><br>3. AS160 Independent Role: IRAP-KD Cells vs. AS160 Cells knockdown (KD) and AS160/IRAP Cells double knockdown.<br><br>4. Sorting step: GLUT4 Wild-Type (WT) compared to GLUT4 F5A and E488A, E490A mutations in IRAP-KD cell background.<br><br>5. Vesicle specialization: GLUT4 is exemplified in IRAP-KD cells compared to vesicles controlled by AS160-4P (dominant interference mutation).<br><br>6. Cargo Specialization: Comparison of GLUT4 transport with Transferrin Receptor (TR) transport in IRAP-KD cells.	In fat cells, IRAP plays an important role in the storage and classification of GLUT4 into the specialized cavity (GSV). IRAP knockdown increases GLUT4 on the membrane in the unstimulated state and repositions GLUT4 into the endosome, but does not affect AS160. IRAP is both a commodity and a primary regulator of the insulin-regulated GLUT4 transport pathway.	IRAP, TBC1D4 (AS160)	IRAP: inhibition<br>AS160: inhibitor	IRAP regulates the classification of GLUT4 from the endosome into specialized vesicles (GSVs) based on the cytoplasmic domain, but is not required for AS160 recruitment. IRAP and AS160 operate independently in GLUT4 storage, IRAP is both a commodity and a conditioning element.	Translocation										Unclear risk (Not reported)	Unclear risk (Not reported)	Low risk	Unclear risk (Not reported)	Unclear risk (Not reported)	Low risk
A.390	10.1074/jbc.M114.555714	Paul Duffield Brewer, 2014	USA and Australia	Identify novel regulatory steps of Glut4 transport by comparing the trafficking kinetics of Glut4, the transferrin receptor (Tf receptor), and LRP1 in fibroblasts and adipocytes (adipocytes) before and after differentiation, as well as under the influence of insulin and AS160 knockdown.	3T3-L1 cells (in the form of undifferentiated fibroblasts and differentiated fat cells)	1. Insulin: Stimulation with a concentration of 100 nM for 30-45 minutes.<br><br>2. Differentiation: Comparison between fibroblasts and adipocytes.<br><br>3. AS160 Knockdown: Uses shRNA to reduce AS160 expression.<br><br>4. Nystatin: Processing to inhibit nystatin-dependent cell entry pathway.<br><br>5. LY294002 (LY): PI3K inhibitor (50 μM) for inhibition of cell extinction.	1. Flow Cytometry: Quantitative measurement of Glut4, Tf receptor, LRP1 levels on the cell surface and total (using HA-Glut4/GFP reporter).<br><br>2. Kinetic Assays (Exocytosis/Endocytosis):<br>- a-HA Uptake Assay: Measures the rate of exocitation (Kex).<br>- Transition Experiments: Measure steady-state change (Basal to Insulin, Insulin + LYi) to infer transport steps.<br>- Transferrin Efflux: Measures the rate at which Tf receptors are exfoliated.<br>- α2-M Uptake: Measures the cell entry rate of LRP1.<br><br>3. Mathematical Modeling: Simulating kinetic data to determine speed constants.	1. Steady state: After 30-45 minutes incubation with insulin or not.<br><br>2. Time course: Continuous monitoring for a period of 0 to 300 minutes (for uptake assay) or 0-60 minutes (for transition/efflux assay).	1. Fibroblasts vs Adipocytes: Comparison Between Two Differentiated States.<br><br>2. Basal vs Insulin: Comparison Between Basal State and Insulin Stimulation.<br><br>3. Control vs AS160 KD: Comparison between normal cells and AS160 deficient cells.	Membrane GLUT4 levels are determined by 6 independent steps: cell entry, degradation, classification, isolation, release, and anchoring/fusion.<br><br>GLUT4 has a very different transport kinetics than the transferrin receptor: it internalizes more slowly, recycles through a slow pathway, and as the cell differentiates into fat cells, the total amount and level of GLUT4 on the membrane increase significantly. In fat cells, GLUT4 is not only recycled from the endosome, but is also classified into specialized storage vesicles (GSVs). Insulin strongly regulates this process, especially at the step of releasing GLUT4 from the GSV to release the membrane. AS160 plays a role in controlling this release step and can also participate in the introduction of GLUT4 into the GSV, while the final steps such as anchoring, docking, and fusing the GSV to the membrane take place independently of AS160.	Tf receptor, LRP1<br>AS160 (TBC1D4).	AS160: Inhibition<br>Tf receptor, LRP1: stimulation	The study offers a "Dynamic Retention" model, in which the level of GLUT4 on the membrane is controlled in 6 steps. The key finding was that the isolation of GLUT4 into GSV was strongly regulated by insulin rather than passively. The AS160 not only controls the release of GLUT4 from the GSV, but also controls the introduction of GLUT4 and LRP1 into the GSV. Finally, the tethering/docking/fusion step of GSV exits the insulin-dependent but non-AS160-dependent membrane, creating the second regulatory layer of the GLUT4 prongoma.	Signaling										Unclear risk (Not reported)	Unclear risk (Not reported)	Low risk	Unclear risk (Not reported)	Unclear risk (Not reported)	Low risk
A.394	10.1128/MCB.21.14.4785-4806.2001	Jonathan S. Bogan, 2001	USA	Study of cell specificity and culture conditions influencing insulin-responsive GLUT4 transport; specifically comparing GLUT4 translocation kinetics between 3T3-L1 fat cells and CHO cells, and determining the role of amino acid concentrations in this process.	1. 3T3-L1 cells (differentiated fibroblasts and fat cells)<br>2. Other: CHO-K1 cells, NIH 3T3 cells, AML12 liver cell line	1. Insulin: Stimulates with concentrations of 80 nM, 160 nM, or 480 nM at different intervals.<br><br>2. Culture medium: Change the amino acid concentration by using DMEM (high concentration), MEM (medium), F12 (low), or MEM without amino acids.<br><br>3. Rapamycin: Treat with concentrations of 20, 50, 100, 200, 500 ng/ml for 36 hours.<br><br>4. PI3K inhibitors: Wortmannin (100 nM) and LY294002 (50 μM).	1. Flow cytometry (FACS): Measure the fluorescence ratio of extracellular Myc epitopes (PE staining) to the total amount of GLUT4 reporter protein (GFP) per living cell to quantify the ratio of GLUT4 on the plasma membrane.<br><br>2. Subcellular fractionation & Immunoblotting: Separation of membrane segments (PM, LDM) and determination of endogenous GLUT4 by Western blot.<br><br>3. Immunofluorescence microscopy: Observe the distribution of GLUT4 reporters in cells.<br><br>4. Sucrose density gradient centrifugation: Analysis of the distribution of GLUT4 in intracellular vesicles.<br><br>5. Vesicle immunopurification: Determination of the colocation of the GLUT4 reporter and endogenous GLUT4.	1. Steady-state: After 20-30 minutes of insulin stimulation.<br><br>2. Kinetics (Time course): Measure at multiple times after adding insulin (e.g., 0.5, 1, 2, 3, 4, 5, ... 30 minutes) to observe the "overshoot" phase and the steady state.	1. Cells: 3T3-L1 adipocytes vs 3T3-L1 preadipocytes vs CHO cells vs NIH 3T3 cells.<br><br>2. Environmental: DMEM (high amino acids) vs F12 (low amino acids) vs MEM (different aa concentrations).<br><br>3. Treatment: Basal (non-insulin) vs Insulin-stimulated.<br><br>4. Inhibitors: Control vs Rapamycin vs PI3K inhibitors.	Insulin mobilizes GLUT4 from an insulin-sensitive intracellular cavity at both 3T3-L1 and CHO, with similar kinetics and levels when cultured under the same conditions. However, in CHO cells, the direction of GLUT4 into this insulin-responsive cavity depends on the culture conditions: amino acid concentrations determine GLUT4 distribution through a rapamycin-sensitive pathway. Amino acids also regulate the level of insulin-stimulated GLUT4 translocation in 3T3-L1 fat cells.	Amino acids	Amino Acids: Stimulating	Research shows that amino acid metabolism can directly control GLUT4 transport: amino acid concentrations (most likely mTOR) determine GLUT4's classification into the insulin-sensitive peripheral cavity. When amino acids are deficient or inhibited by rapamycin, GLUT4 is retained in the perinucleus region and cannot participate in the rapid mobilization phase in the presence of insulin.	Translocation										Unclear risk (Not reported)	Unclear risk (Not reported)	Low risk	Unclear risk (Not reported)	Unclear risk (Not reported)	Low risk
A.396	10.1042/bj3170945	Mittur N. Jagadish, 1996	Australia	Identify and characterize cDNA molecules encoding SNAP-25 isomers A and B, syntaxin 4, and synaptobrevin 1 and 2 from major human insulin-responsive tissues (skeletal muscle and fat) to understand the fusion mechanism of glucose-transporting protein (GLUT4) carrier vesicles with the plasma membrane.	human skeletal muscle tissue and adipose tissue; mouse 3T3-L1 fat cell line; PC12 cells (positive control).	1. Insulin: Used to stimulate differentiated 3T3-L1 cells and laboratory rats (to obtain cell membranes from adipose tissue).<br><br>2. Cloning & PCR: Using specific primer pairs to amplify and sequence the SNAP-25, syntaxin 4, synaptobrevin 1 and 2 genes.<br><br>3. Yeast two-hybrid system: Used to test protein-protein interactions.<br><br>4. Flow cytometry: Used to analyze the binding of recombinant proteins to low-density microsomes.	1. PCR & Southern hybridization: Determination of the presence and sequence of transcriptional transcripts (mRNAs) of SNARE proteins.<br><br>2. Immunofluorescence microscopy: Determines the location and presence of SNAP-25 and GLUT4 proteins in cells.<br><br>3. Immunoprecipitation & Western blotting: Analysis of SNAP-25 protein from cell extracts and plasma membranes.<br><br>4. Yeast two-hybrid system assay: Measures β-galactosidase activity to evaluate protein interactions.<br><br>5. Flow cytometry: Fluorescence measurement to determine the binding of recombinant proteins (syntaxin 4, SNAP-25) to GLUT4-containing vesicles.	There is no specific time of intervention due to mainly molecular presence studies	1. Tissue/Cells: Comparison between adipose tissue and skeletal muscle tissue; between 3T3-L1 cells and PC12 cells.<br><br>2. Isoform: Comparison between SNAP-25 A and B; between synaptobrevin 1 and 2.<br><br>3. Proteins: Comparison of the interaction of syntaxin 4 with synaptobrevin 1 versus synaptobrevin 2.	Insulin-sensitive tissues contain SNAP-25 A/B, syntaxin 4, and synaptobrevin 1/2. Isoform A is mainly in adipose tissue, B in skeletal muscle. SNAP-25 is present in 3T3-L1 and fat cell membranes. Syntaxin 4 from skeletal muscle has nucleotide changes, synaptobrevin 1 has Thr102→Ala changes. Syntaxin 4 interacts with synaptobrevin 2 but not with 1; The syntaxin complex 4–SNAP-25 binds strongly to the GLUT4 vesicles, whereas SNAP-25 alone binds poorly.	SNAP-25, Syntaxin 4, Synaptobrevin 1 and 2	Syntaxin 4 & SNAP-25: Stimulation<br><br>Synaptobrevin 2 (VAMP2): Stimulation<br><br>Synaptobrevin 1: Non-Interactive	Research shows that the core SNARE trio (Syntaxin 4, SNAP-25, Synaptobrevin 2) are present in insulin-sensitive muscles and fats. The t-SNARE complex (Syntaxin 4–SNAP-25) on the plasma membrane creates a strong binding site to v-SNARE Synaptobrevin 2 on the GLUT4 vesicle, regulating docking and fusion of the insulin-responsive GLUT4 vesicle.	Docking, fusion										Unclear risk (Not reported)	Unclear risk (Not reported)	Low risk	Unclear risk (Not reported)	Unclear risk (Not reported)	Low risk
A.398	10.1042/BJ20060793	Tsung-Yin J. YEH, 2007	USA	To investigate the role of GLUT4, IRAP and tankyrase in regulating the migration of GLUT4 storage vesicles (GSV) in 3T3-L1 fat cells.	3T3-L1 adipocytes	1. Insulin: 20 nM for 20-30 minutes.<br><br>2. siRNA: To knock down GLUT4, IRAP, or tankyrase. Specific siRNAs include: G4-A, G4-B (GLUT4); vp-A, vp-B (IRAP); T1A, T1B (tankyrase).<br><br>3. Sorbitol: 600 mM for 20 minutes (for osmotic shock).<br><br>4. PJ34: 80 uM for 45-60 min (to inhibit PARP).	1. Western blotting (Immunoblotting): To measure protein levels in whole-cell extracts, plasma membrane (PM) fractions, and other subcellular fractions.<br><br>2. Glucose uptake assay: Measure the absorption of [3H]deoxy-D-glucose.<br><br>3. Differential centrifugation: To separate subcellular segments (PM, HDM, LDM, cytosol).<br><br>4. Iodixanol equilibrium sedimentation: To analyze the density of GLUT4 and IRAP-containing vesicles.<br><br>5. Immunoadsorption: To isolate vesicles containing GLUT1.	1. Knockdown: 2-3 days after electroporation of siRNA (day 8 or 9 of differentiation).<br><br>2. Insulin/Sorbitol/PJ34 stimulation: Just before cell harvesting or glucose uptake measurement.	1. GLUT4/IRAP/Tankyrase Knockdown experiment: control group vs GLUT4/IRAP/Tankyrase Knockdown<br><br>2. PARP Inhibition Experiment (PJ34): Control Group vs PJ34 Inhibition Group (80 μM)<br><br>3.. Iodixanol Gradients: Basal State vs Tankyrase KD vs Insulin Stimulation Group	IRAP knockdown sharply reduces GSV displacement. Reducing tankyrase also reduces GSV translocation without affecting insulin phosphorylation signaling, and shifts GLUT4 and IRAP into less floating endosome cavities. These findings suggest that physiological GSV transport depends in part on the presence of IRAP in these vesicles, and that this process is regulated by tankyrase (PARP activity)	IRAP, Tankyrase	Stimulation	Tankyrase, through PARP activity, regulates the sorting of GLUT4 and IRAP into insulin-sensitive vesicles (low floating vesicles). When tankyrase is deficient or inhibited, GLUT4/IRAP is trapped in a high-density cavity, insulin insensitivity, and decreased cell exorgesis. IRAP plays a more important role than GLUT4 in navigating GSV bags.	Translocation										Unclear risk (Not reported)	Unclear risk (Not reported)	Low risk	Unclear risk (Not reported)	Unclear risk (Not reported)	Low risk
A.399	10.24546/81000871	Takayuki Kawaguchi, 2008	Japan	This study aims to analyze exactly the steps that the wortmannin-sensitive insulin signaling pathway takes in the entire GLUT4 translocation process	Adipocytes of the 3T3-L1 lineage.	1. Insulin: Stimulates cells at a concentration of 100 nM for 5 or 20 minutes.<br><br>2. Wortmannin: Cell treatment with a concentration of 100 nM for 30 minutes before insulin stimulation to inhibit PI 3-kinase class IA.	1. 2-deoxy-D-glucose absorption assay: Measures glucose transport activity.<br><br>2. Immunoblot analysis (Western blot): Evaluation of the phosphorylation level of Akt (pAkt) and total amount of Akt.<br><br>3. Confocal immunofluorescence microscopy: Observation of the migration of GLUT4myc7-GFP (green GFP signaling) and its presence on the cell surface (red anti-Myc/PE antibody signaling) on intact cells.<br><br>4. Plasma membrane lawn assay: Observe the tethering/docking of GLUT4 vesicles with plasma membrane by ultrasonic cell disruption leaving membrane fragments attached to the glass slats and fluorescent immunosuppression staining.	1. Glucose absorption: After 20 minutes of insulin stimulation (measured in 5 minutes of absorption).<br><br>2. pAkt signal: After 5 minutes of insulin stimulation.<br><br>3. GLUT4 translocation (microscopy): After 20 minutes of insulin stimulation	1. Basal (Basic): No insulin stimulation, no wortmannin treatment.<br><br>2. Insulin: Stimulate insulin, do not treat wortmannin.<br><br>3. Wortmannin + Insulin: Treat wortmannin first, then stimulate insulin.<br><br>4. Wortmannin (Basal): Treats wortmannin, does not stimulate insulin (shown in some charts).	Wortmannin blocks the membrane fusion step of GLUT4 vesicles with the plasma membrane but does not affect vesicle migration or anchoring/binding to the membrane in 3T3-L1 fat cells. Shows that the PI3K-dependent signal branch mainly controls fusion	PI 3-kinase	Stimulation	The study found that PI3K-dependent insulin signaling primarily controls the membrane conjugation step of the GLUT4 vesicle, while migration and tethering/docking appear to be regulated by the PI3K-dependent signaling branch	Signaling/fusion										Unclear risk (Not reported)	Unclear risk (Not reported)	Low risk	Unclear risk (Not reported)	Unclear risk (Not reported)	Low risk
A.401	10.1074/jbc.C111.226908	Hiroyuki Sano, 2011	USA	Determination of guanine nucleotide exchange factor (GEF) for Rab10 involved in insulin-stimulated GLUT4 translocation	1. Adipocytes (adipocytes) of the 3T3-L1 line<br><br>2. HEK293 cells.	1. Insulin Stimulation: Fat cell stimulation with 160 nM insulin.<br><br>2. Knockdown: Use siRNAs (small interfering RNAs) to inhibit the expression of Dennd4C and Rabin8 proteins.<br><br>3. Ectopic Expression: Wild-type Dennd4C (WT) or non-phosphorylated mutant Dennd4C (S1043A/S1096A/S1321A mutant).	1. Rab10 GEF Assay: Measure the ratio of Rab10 to GTP mount by marking 32P and TLC analysis.<br><br>2. Flow Cytometry: Measure the amount of GLUT4 on the cell surface (using the HA-GLUT4-GFP reporter).<br><br>3. Immunoblotting: Determination of levels of Dennd4C, Rabin8, Akt, TBC1D4, IRAP proteins.<br><br>4. Phosphoproteomic analysis (SILAC): Analysis of phosphorylation sites of Dennd4C.	1. GLUT4 translocation: After 30 minutes of insulin stimulation.<br><br>2. Knockdown effect: 72 hours after siRNA transfusion.<br><br>3. Overexpression effect: 24 hours after plasmid infusion.	1. Comparison of Basal and Insulin<br><br>2. Knockdown comparison: groups with and without siRNA transfer<br><br>3. Comparison of Overexpression: Cells are electrolyzed with a hollow vector (vector) and a mutant Dennd4C WT or Dennd4C transfer group that cannot be phosphorylated.<br><br>4. Comparison of GEF Activity (in HEK293 cells): Rab10 expressed with Dennd4C compared to Rab10 expressed without Dennd4C.	The study showed that Dennd4C was the key GEF for GLUT4 translocation: knockdown Dennd4C drastically reduced the translocation, while the expression of displacement increased only slightly. Dennd4C was also detected in GLUT4 vesicles in isolation.	Dennd4C, Rab10	Stimulation	Dennd4C is a Rab10-activated GEF on the GLUT4 vesicles, which activates translocation (Dennd4C is located just above the GLUT4 vesicles, which is suitable for this role). Insulin has increased phosphorylation of Dennd4C but does not alter its GEF activity.	Translocation										Unclear risk (Not reported)	Unclear risk (Not reported)	Low risk	Unclear risk (Not reported)	Unclear risk (Not reported)	Low risk
A.402	10.1074/jbc.M108297200	Makoto Kanzaki, 2001	USA	Determining the role of cortical actin and its restructuring in insulin-stimulated GLUT4 translocation in fat cells.	Adipocytes of the 3T3-L1 lineage.	1. Insulin Stimulation: Stimulate cells with 100 nM insulin.<br><br>2. Actin-depolymerizing agents:<br>- Latrunculin B: Use at different concentrations (1, 5, 20, 60 μM) to break down the shell actin structure.<br>- C. difficile toxin B (Toxin B): Used at different concentrations (0.01, 0.1, 0.5, 1 μg/ml) to inactivate Rho GTP-binding proteins.<br><br>3. Actin-stabilizing agent:<br>- Jasplakinolide: Used at different concentrations (0.5, 1, 10, 20 μM) to promote the formation and/or stabilization of actin filaments.<br><br>4. Mutated gene expression: Microinjection of TC10/T31N (dominant-interfering) or Cdc42/T17N encoded plasmids.	1. Confocal fluorescence microscopy: Observation of the structure of actin (using rhodamine-phalloidin or YFP-actin) and GLUT4 (immunofluorescence) on intact cells or plasma membrane sheets.<br><br>2. 2-Deoxyglucose uptake assay: Measures glucose transport activity.<br><br>3. Time-lapse microscopy: Observation of the kinetics of actin (YFP-actin) on living cells.<br><br>4. Western blotting & Immunoprecipitation: Evaluation of insulin signaling (phosphorylation IR, IRS, Akt, Cbl).	1. Glucose absorption: After 30 minutes of insulin stimulation (measured in 10 minutes of absorption).<br><br>2. GLUT4 translocation (PM sheet): After 30 minutes of insulin stimulation.<br><br>3. Actin remodeling (Time-lapse): Continuous observation before and after adding insulin (0, 5, 10 minutes...).	1. Glucose Transport/GLUT4 Translocation Experiment and Insulin Signaling Experiment: control group and pharmacological intervention group (Latrunculin B, Toxin B, or Jasplakinolide)<br><br>2. Actin Observation Experiment: control group, pharmacological intervention group (Latrunculin B, Toxin B, or Jasplakinolide) and mutation interference group (TC10/T31N (dominant-interfering mutant (TC10/T31N expressing cells) compared to non-expressing or Cdc42/T17N expressing cells (control mutant))	F-actin is concentrated in the cortical actin of 3T3-L1 fat cells. Disruption or over-stabilization of this actin layer inhibits GLUT4 translocation and glucose uptake even though the initial insulin signal (IR/IRS/Akt) is intact. Time-lapse observations show that insulin triggers dynamic restructuring of cortical actin, followed by increased perinuclear actin polymerization demonstrating cortical actin (rather than stress fibers/lamellipodia/filopodia) and that its restructuring is essential for GLUT4 translocation.	Cortical Actin (F-actin)	Stimulation	Insulin stimulates a dynamic restructuring of the cortical actin layer through the TC10 protein. This restructuring is necessary to allow GLUT4 vesicles to move through the actin envelope and fuse with the plasma membrane. Shell actin not only serves as a static barrier but must undergo dynamic change to support GLUT4 translocation.	Translocation										Unclear risk (Not reported)	Unclear risk (Not reported)	Low risk	Unclear risk (Not reported)	Unclear risk (Not reported)	Low risk
A.403	10.1074/jbc.270.47.27991	Tetsuro Haruta, 1995	USA	The objective was to evaluate the transcellular signaling pathway that leads to insulin-stimulated GLUT4 translocation.	Adipocytes of the 3T3-L1 lineage.	1. Insulin stimulation: at a maximum concentration of 10 ng/ml.<br><br>2. Microinjection of inhibitors and activators (Microinjection):<br>- N17 Ras (dominant-negative mutant p21ras protein): 2.0 mg/ml.<br>- Anti-p21ras antibody: 5 mg/ml.<br>- GST-p85 SH2 fusion protein (PI3-kinase inhibition): 12 mg/ml.<br>- Monoclonal anti-phosphotyrosine antibody (PY20) (anti-phosphotyrosine antibody): 5 mg/ml.<br>- Anti-Shc antibody: 5 mg/ml.<br>- T24 Ras (carcinogenic p21ras protein, continuous activity): 2.0 mg/ml.	1. Immunofluorescence Microscopy: To detect GLUT4 translocation to the cell membrane (using anti-GLUT4 F349 or 1F8 antibodies) and c-Fos expression in the nucleus.<br><br>2. BrdUrd incorporation assay: To measure DNA synthesis (evaluate the impact on the mitogenic pathway) in HIRc cells (positive control for the activity of microinjectable agents).	1. GLUT4 translocation: After 20 minutes of insulin stimulation (done 1 hour after microinjection).<br><br>2. c-Fos expression: After cell immobilization (performed 1 hour after T24 Ras microinjection, no insulin required).	1. Basal (Basic): No insulin stimulation.<br><br>2. Insulin: Stimulates insulin (10 ng/ml).<br><br>3. Control IgG: Non-specific sheep antibody microinjection (control for microinjection).<br><br>4. Agent microinjection groups: Comparison of GLUT4 translocation efficacy of cells injected with N17 Ras, Anti-p21, T24 Ras, Anti-Shc, GST-p85 SH2, PY20 with the control IgG injection group.	Inhibition of p21ras activation (T24 Ras) does not affect insulin-induced GLUT4 translocation. Conversely, inhibition of phosphotyrosine or PI3-kinase inhibits this response. This suggests that the p21/MAPK branch is not involved, and tyrosine phosphorylation and PI3-kinase activity are essential for GLUT4 translocation.	p21ras (Ras), PI3-kinase	P21RAS: No Impact<br><br>PI3-kinase: stimulation	Research shows that insulin signaling splits into two branches: the metabolic branch (GLUT4 translocation) and the mitotic branch. GLUT4 translocation requires phosphorylation of tyrosine and PI3-kinase, but does not require p21ras/MAPK, although p21ras is key for mitotic signaling.	Signaling										Unclear risk (Not reported)	Unclear risk (Not reported)	Low risk	Unclear risk (Not reported)	Unclear risk (Not reported)	Low risk
A.404	10.1006/bbrc.1993.2111	Fumihiko Kanai, 1993	Japan	Investigate the role of IRS-1 and PI3-kinase in the signaling pathway of insulin-stimulated GLUT4 translocation	Chinese Hamster Mouse Ovarian Cells (CHO cells).	1. Insulin: Stimulates cells at different concentrations (0.01, 0.1, 1, 10, 100 nM) for 10 minutes.<br><br>2. Wortmannin: Treat cells with different concentrations (0.025, 0.05, 0.1, 1.0 uM) for 20 minutes before adding insulin or NaF.<br><br>3. NaF: Stimulates cells at a concentration of 10 mM for 10 minutes.	1. Anti-myc antibody binding assay: Measure the amount of GLUT4myc translocated to the cell surface by measuring the amount of anti-c-myc antibody binding to the cell<br><br>2. Immunoblotting (Western Blot): Use anti-phosphotyrosine (PY20) antibodies to detect tyrosine phosphorylation of IR and IRS-1.<br><br>3. PI3-kinase assay: Measures PI3-kinase activity in immunoprecipitation with anti-p85 antibodies.	1. Insulin stimulation (for measurement of phosphorylation activity and PI3-kinase): 10 minutes.<br><br>2. Insulin or NaF stimulation (for measurement of GLUT4 translocation after Wortmannin treatment): 20 minutes.	1. CHO-GLUT4myc expresses normal insulin receptor (IR) compared to CHO-GLUT4myc expresses mutant receptor (IRF972).<br><br>2. Treated Wortmannin vs. Non-Treated.<br><br>3. Insulin stimulation versus NaF stimulation.	In cells carrying the IRF972 mutation receptor, both IRS-1 phosphorylation and GLUT4 translocation are reduced. Wortmannin inhibits PI3-kinase and insulin-induced GLUT4 translocation, but does not affect NaF-induced GLUT4 response. Shows that tyrosine phosphorylation of IRS-1 and activation of PI3-kinase are important components of the insulin-induced GLUT4 translocation signaling pathway.	IRS-1, PI3-kinase	Stimulation	Tyrosine phosphorylation of IRS-1 and activation of PI3-kinase are key signaling steps between insulin receptors and GLUT4 translocation. PI3-kinase is a specific mediator of insulin signaling	Signaling										Unclear risk (Not reported)	Unclear risk (Not reported)	Low risk	Unclear risk (Not reported)	Unclear risk (Not reported)	Low risk
A.405	10.1038/35073608	Shian-Huey Chiang, 2001	USA	Investigating the insulin signaling pathway that leads to GLUT4 translocation through activation of the CAP-dependent TC10 protein.	Adipocytes of the 3T3-L1 lineage.	1. Insulin: Stimulates cells with a concentration of 100 nM for 30 minutes<br><br>2. Wortmannin: PI(3)K inhibitor, used at concentrations of 0, 0.1, 1, 10, 100 nM.<br><br>3. Mutant gene expression: Transfection/Electroporation of TC10-expressing plasmids (WT, T31N, Q75L, F42L), Cdc42 (WT, T17N), CAP (WT, ΔSH3), C3G (WT, Δcdc25), p110CAAX.<br><br>4. Microinjection: TC10 or Cdc42 cDNA is injected along with MBP-Ras (marker) into the cell nucleus.	1. Glucose Absorption: Measure the absorption rate [3H]2-deoxyglucose (2-DG)<br><br>2. Immunofluorescence microscopy: Observation of the transfer of GLUT4 to the plasma membrane on plasma membrane sheets or intact cells expressing GLUT4-EGFP.<br><br>3. GST-Pak1 PBD pull-down assay: Measures the activity level (GTP-bound) of TC10, Cdc42, Rac1.<br><br>4. Guanine nucleotide exchange assay: Measure the nucleotide exchange activity of C3G for TC10 in vitro.<br><br>5. Immunoprecipitation & Western blotting: Analysis of protein interactions and protein expression levels (Cbl, CAP, CrkII, C3G, TC10...).<br><br>6. Subcellular fractionation: Separates soluble and insoluble fractions in Triton X-100 (lipid raft) to monitor protein translocation.	1. Glucose uptake/Translocation: After 30 minutes of insulin stimulation.<br><br>2. Signaling (TC10 activation, translocation of C3G/CrkII): 0, 1, 2, 3, 5, 10, 15, 20, 30 minutes after insulin stimulation.	1. Comparison of Functional Inhibition: Vector control compared to GTPase mutation/WT proteins (TC10/WT, TC10/T31N, CDC42/WT, CDC42/T17N)<br><br>2. Signal Pathway Comparison:<br>- Akt Activation (PI(3)K dependent) vs. TC10 Activation<br>- Insulin stimulation versus PDGF<br><br>3. CAP-dependent comparison: LacZ (control) or CAP/WT expressing cells vs. CAPΔSH3 (Dominant-interfering CAP) expressing cells<br><br>4. Comparison of Signal Completeness: Insulin vs. C3G (TC10 Activation) + p110CAAX (PI (3)K Activation)	Insulin activates Cbl and pulls the CrkII–C3G complex to the lipid rafts, where C3G activates TC10 (PI3K-independent). TC10 is required for glucose uptake and GLUT4 translocation, and this pathway works in tandem with PI3K. TC10 and PI3K together stimulation is necessary to produce maximum GLUT4 response.	TC10 (Rho family GTPase), PI(3)K	Stimulation	Identify a second signaling pathway, parallel to the PI(3)K pathway, which is necessary for full GLUT4 translocation. This pathway includes: Insulin -> Phosphorylation Cbl -> Cbl-CAP Complex Recruits CrkII-C3G to lipid raft -> C3G (at lipid raft) TC10 activation (converts GDP to GTP)	Signaling										Unclear risk (Not reported)	Unclear risk (Not reported)	Low risk	Unclear risk (Not reported)	Unclear risk (Not reported)	Low risk

A.407	10.1074/jbc.C300063200	Hiroyuki Sano, 2003	USA	Determine the role of the AS160 protein and its phosphorylation in regulating insulin-stimulated GLUT4 translocation.	Adipocytes of the 3T3-L1 lineage.	1. Insulin: Stimulates cells with a concentration of 160 nM for 10 or 30 minutes.<br><br>2. Gene expression + point mutation: Cell transfection with mutated AS160-coding plasmids at phosphorylation sites and/or mutations at the GAP domain.	1. Mass Spectrometry (MS/MS): Determination of phosphorylation sites on AS160.<br><br>2. Fluorescence Microscopy: Observe and quantify the displacement of HA-GLUT4-GFP to the cell membrane (ratio of Cy3 surface signals to total GFP signals).<br><br>3. Immunoblotting (Western Blot): Check the expression level of AS160 and mutant forms.	1. Insulin stimulation (main): 30 minutes (to measure GLUT4 displacement).<br><br>2. Insulin stimulation (phosphorus localization): 10 minutes (to purify AS160 for MS/MS).	1. Comparison of 4 groups of Phosphorylation/GAP Mutations: All groups with/without Insulin:<br>- Vector Control Group<br>- Wild-type group AS160 (WT)<br>- Group 4P (Mutation of 4 phosphorus sites to Alanine): Decreases the function of phosphorus regulation.<br>- Group R/K (GAP to Lysine Mutation): Inactivates the GAP function.<br>- Group 4P, R/K (4P mutation combined with R/K)<br><br>2. Comparison of the number of phosphorylation sites Mutation: Comparison of the translocation inhibition effect of Ser/Thr cumulative mutations to Alanine: mutations from 1P to 6P	The study identified six phosphorylation sites of AS160, of which five sites increased phosphorylation in the presence of insulin. When mutating from two or more sites to alanine, AS160 strongly inhibits GLUT4 translocation, but when it is simultaneously inactivated at GAP, the inhibitory effect disappears. Shows that phosphorylation of AS160 is required to deinhibit and allow GLUT4 to transfer to the membrane.	AS160	Inhibition	In the presence of insulin, Akt phosphorylation AS160 causes GAP inactivation, Rab switches to the active GTP form, activating GLUT4 translocation.	Signaling										Unclear risk (Not reported)	Unclear risk (Not reported)	Low risk	Unclear risk (Not reported)	Unclear risk (Not reported)	Low risk
A.408	10.1074/jbc.M109.035568	Grantley R. Peck, 2009	USA and Sweden	Determination of phosphorylation sites of TBC1D1 in insulin-stimulated C2C12 muscle cells and its role in regulation of GLUT4 translocation	1. C2C12 muscle cells.<br>2. 3T3-L1 fat cells.<br>3. Human muscle biopsy samples: Taken from healthy people and type 2 diabetics.	1. Insulin: Stimulates cells with a concentration of 160 nM<br><br>2. AICAR: Stimulates cells with a concentration of 1 mM<br><br>3. Mutated gene expression:<br>- Transfection of 3T3-L1 cells with mutated TBC1D1-expressing plasmids at phosphorylation sites and/or mutations at the GAP domain<br>- TBC1D1 R125W body associated with obesity	1. Mass Spectrometry (LC-MS/MS): Determination of phosphorylatation sites on TBC1D1 isolated from C2C12 myotubes.<br><br>2. GLUT4 Translocation Assay (Flow cytometry): Measure the amount of HA-GLUT4-GFP on the surface of 3T3-L1 cells using anti-HA antibodies and flow analysis.<br><br>3. Transferrin Receptor Translocation Assay: Measures the amount of endogenous transferrin receptors on the cell surface.<br><br>4. Immunoblotting (Western Blot): Check the level of TBC1D1 protein expression in human cells and tissue samples.	1. Phosphorus Locating: Insulin 15 minutes; AICAR 45 minutes.<br><br>2. GLUT4 translocation: Insulin 30 minutes; AICAR 70 minutes; or AICAR 70 minutes + Insulin last 30 minutes.<br><br>3. TfR translocation: Insulin 15 minutes.	1. Basal (Basic) vs Insulin/AICAR.<br><br>2. Vector control (V) vs Wild-type TBC1D1 (WT) vs Mutant TBC1D1 (1P, 2P, 3P, R/K...)<br><br>3. Control vs Type 2 Diabetic: Comparison of TBC1D1 levels in human muscle tissue.	Non-phosphorylated TBC1D1 mutations inhibit GLUT4 translocation, AMPK can partially reduce this inhibitory effect. This suggests that phosphorylation of TBC1D1 is a mandatory step for GLUT4 to be mobilized.<br><br>The obesity-associated R125W variant also inhibits GLUT4 in the same way as TBC1D1 WT, TBC1D1 levels in healthy human muscles and type 2 diabetes are the same.	TBC1D1	Inhibition	In the presence of insulin, Akt phosphorylates TBC1D1 at Thr-590, Ser-501, Thr-499, thereby reducing GAP activity, allowing Rab to activate GLUT4 translocation. AMPK can also phosphorylate TBC1D1, creating an additional parallel/complementary conditioning layer.	Signaling										Unclear risk (Not reported)	Unclear risk (Not reported)	Low risk	Unclear risk (Not reported)	Unclear risk (Not reported)	Low risk
A.410	10.1091/mbc.e04-12-1124	Ping Huang, 2005	USA	Explore the potential role of Phospholipase D1 (PLD1) and its product Phosphatidic acid (PA) in the translocation and membrane fusion of insulin-stimulated GLUT4.	Adipocytes of the 3T3-L1 lineage.	1. Changes in PLD1 (Chronic):<br>- Increased function: Adenovirus infection expressing rabies-type PLD1.<br>- Decreased function: PLD1-K898R-expressing adenovirus infection or use of RNAi (retrovirus-induced) to eliminate 85–90% of endogenous PLD1 expression.<br><br>2. Stimulate cells with insulin<br><br>3. Chemical/lipid intervention:<br>- Use 1-butanol to measure PLD activity.<br>- Use of Lysophosphatidylcholine to rescue membrane fusion defects caused by PA deficiency.	1. PLD activity assay: Measures the accumulation of phosphatidylbutanol in vivo (transphosphatidylation).<br><br>2. 2-Deoxyglucose uptake: Measurement of glucose uptake with radioactive markers.<br><br>3. Myc-Glut4-EGFP / HA-Glut4-EGFP translocation assays: Measures the ratio of surface fluorescent signals (anti-myc/anti-HA on impermeable cells) to total (GFP).<br><br>4. Plasma membrane sheet assay: Evaluate the docking of GLUT4 bags on plasma membrane plates.<br><br>5. Western blotting: Confirm the level of protein expression and phosphorylation.<br><br>6. Confocal microscopy: Observe the colocation and displacement of PLD1 and GLUT4.	1. PLD activity: Measured at 2, 5, 10, 20 minutes after insulin stimulation (peak at 2-5 minutes).<br><br>2. Translocation/Fusion/Glucose uptake: Usually measured after 5 minutes of insulin stimulation (to assess the initial rate) or up to 20-30 minutes.	1. Status: Basal Cells vs. Insulin Stimulating Cells.<br><br>2. PLD1 expression: Control cells (Vector/Control) compared to PLD1 overexpressing cells (Ad-PLD1) or PLD1-RNAi cells or PLD1-K898R expressing cells.<br><br>3. Salvage: PLD1-RNAi + Insulin vs. PLD1-RNAi + Insulin + LPC.<br><br>4. Docking: PLD1-RNAi vs. Exo70-N (docking inhibition).	The PLD1 on the GLUT4 vesicle is activated by insulin and supports the membrane fusion step by PA generation. Reducing PLD1 does not affect GSV's transport or docking, but it blocks the fusion step, causing GLUT4 to not release the membrane. Exogenous lipid replenishment can save this phenomenon.	Phospholipase D1 (PLD1), Phosphatidic acid (PA)	Stimulation	The PA generated by PLD1 is the rate-limiting step in the final stage of GSV fusion. PA reduces the energy required to bend the membrane and expand the fusion pore, or to recruit proteins that support fusion. When PLD1 is missing, the GLUT4 bag still arrives and docks, but the fusion step cannot be performed.	Fusion										Unclear risk (Not reported)	Unclear risk (Not reported)	Low risk	Unclear risk (Not reported)	Unclear risk (Not reported)	Low risk
A.411	10.1073/pnas.93.26.15169	Bentley Cheatham, 1996	USA and Canada	Determining the role of SNARE proteins (e.g., VAMP-2, cellubrevin, syntaxin-4) in insulin-stimulated GLUT4 translocation	Adipocytes of the 3T3-L1 line are permeated with Streptolysin O	1. Insulin: Stimulates cells with a concentration of 100 nM for 10 minutes.<br><br>2. Botulinum neurotoxin D (BoNT D): Treatment of membrane permeable cells at a concentration of 100 nM for 20-30 minutes (for cleavage of VAMP-2 and cellubrevin).<br><br>3. Botulinum neurotoxin C (BoNT C): Treat membrane permeable cells at a concentration of 100 nM for 20-30 minutes (to cleave syntaxin 1, 2, 3 but not syntaxin 4).<br><br>4. IgA protease: Cell treatment with a concentration of 940 nM for 20-30 minutes (for VAMP-2-specific cleavage).<br><br>5. GST fusion proteins: Cell treatment with soluble GST-VAMP-2 or GST-syntaxin-4 fusion proteins (without transmembrane domain) at a concentration of 10uM	1. Immunofluorescence of Plasma Membrane Sheets: Observe and evaluate the transfer of GLUT4 to the plasma membrane on cell membrane fragments.<br><br>2. Immunoblot analysis (Western blot): Examine the degradation of SNARE proteins (VAMP-2, cellubrevin) in low-density microsomal membrane (LDM) segmentation.<br><br>3. Co-immunoprecipitation (Co-IP): Determines the physical interaction between VAMP-2 and endogenous syntaxin-4.	After 10 minutes of insulin stimulation (after pre-incubation with GST toxins or proteins).	1. BoNT Experiment (VAMP-2/Cellubrevin Role Test): Control group (buffer only) vs BoNT D group (VAMP-2/cellubrevin inhibition), BoNT C group (syntaxins-1A/1B/2/3 inhibition) (all insulin-processing).<br><br>2. IgA Protease Experiment (VAMP-2 and Cellubrevin Differentiation): Control group (no protease) vs IgA Protease group (all insulin processing).<br><br>3. Soluble Protein Experiment (Competitive Inhibition): Control group (buffer only) vs GST group, GST-VAMP2 group, GST-syntaxin-4 group (all insulin processing).<br><br>4. Protein Interaction (IP) experiments: anti-syntaxin-4 IP group vs. Non-immunosuppressive Serum IP group.	BoNT/D treatment (excision of VAMP-2 and cellubrevin) completely loses the ability of insulin to stimulate GLUT4 translocation. Similarly, introducing soluble forms of VAMP-2 or syntaxin-4 (GST-fusion) into cells also blocks this process. Demonstrate that the SNARE complex (VAMP-2–syntaxin-4–SNAP-25) is essential for insulin-activated GLUT4 translocation.	SNARE proteins (VAMP-2, Cellubrevin, Syntaxin-4)	Stimulation	Adipocytes using an endocrine neuron-like docking–fusion mechanism, dependent on the SNARE complex: VAMP-2 on the GLUT4 vesicle and Syntaxin-4 on the membrane are essential for insulin-induced GLUT4 exocytosis. Cellubrevin may be present on a different vesicle store (endosome-derived) and is less involved in acute reactions to insulin than VAMP-2.	Docking/ Fusion										Unclear risk (Not reported)	Unclear risk (Not reported)	Low risk	Unclear risk (Not reported)	Unclear risk (Not reported)	Low risk
A.413	10.1042/BJ20070802	Natalie P. Smithers, 2008	UK	Study of the interaction between munc18c and syntaxin-4 in intact living cells using fluorescence correlation spectroscopy (FCS) to clarify the mechanism by which insulin regulates the binding of GLUT4 vesicles.	1. Adipocytes (adipocytes) of the 3T3-L1 line<br>2. Chinese hamster ovarian cells (CHO)	1. Insulin: Stimulates cells with a concentration of 100 nM for 20 minutes.<br><br>2. Gene expression: Transfection of munc18c-encoded plasmids (full-length or short snippets) tagged EYFP or Xpress, syntaxin-4 tagged GST or EYFP, and 80K-H.	1. Fluorescence Correlation Spectroscopy (FCS): Measures the diffusion coefficient of fluorescent binding proteins (EYFPs) in living cells to infer the binding state.<br><br>2. Co-immunoprecipitation (Co-IP): Determination of physical interactions between proteins (munc18c, syntaxin-4, 80K-H) in cell extracts.<br><br>3. GST pull-down assay: Identify the binding region on munc18c with syntaxin-4 in vitro.<br><br>4. Immunoblotting: Check the level of protein expression.	1. Serum Starvation: 24 hours before FCS measurement or insulin treatment.<br><br>2. Insulin Stimulation: 20 minutes (for all FCS, IP and Pull-down measurements).	1. FCS Experiment: Comparison of total Munc18c (MFL) and Munc18c-295 (M295) (alone or co-expressed with Syntaxin-4) under Basal conditions versus Insulin on CHO and 3T3-L1 cells.<br><br>2. Co-IP Experiment: Comparison of Precipitation Capacity of MFL and M295 with Syntaxin-4 under Basal Versus Insulin Conditions.<br><br>3. 80K-H Experiment (FCS): Comparison of the mobility of MFL and M295 with or without 80K-H under Basal vs. Insulin conditions.<br><br>4. GST Pull-Down Experiment: Comparison of Syntaxin-4 interaction with M295 and shortened variants (M308, M326, M338) in the presence of insulin.	Munc18c has two binding sites: one lost by insulin and another activated by insulin. Thanks to this "rearrangement", insulin causes syntaxin-4 to switch to an open state, allowing it to pair with VAMP-2 on the GLUT4 vesicle and promote docking/fusion of the GLUT4 vesicle to the membrane.	Munc18c, Syntaxin-4	Munc18c: Inhibition<br><br>VAMP-2 and Syntaxin-4: Stimulation	Insulin does not separate munc18c from syntaxin-4, but controls how munc18c binds to syntaxin-4. Insulin disrupts the N-terminal binding site of munc18c with syntaxin-4 and activates the second binding site (aa 295–308). Allows syntaxin-4 to open the profile (which can be supported by the 80K-H protein) to pair with VAMP-2 on the GLUT4 vesicle and promote docking + fusion of the GLUT4 vesicle with the cell membrane.	Docking/ Fusion										Unclear risk (Not reported)	Unclear risk (Not reported)	Low risk	Unclear risk (Not reported)	Unclear risk (Not reported)	Low risk
A.416	10.1210/mend.16.5.0836	Satoshi Shigematsu, 2002	USA	Investigate the potential role of microtubules in regulating the transport of insulin-responsive glucose transporter (GLUT4).	Adipocytes of the 3T3-L1 lineage.	1. Insulin: Stimulates cells with a concentration of 100 nM for a period of 15 to 60 minutes.<br><br>2. Microtubule depolymerization agent: Nocodazole, Colchicine, Vinblastine<br><br>3. Microtubule Stabilizing Agent: Taxol (Paclitaxel)<br><br>4. GLUT4-EGFP Expression Cells: Use for real-time kinetics monitoring.	1. Confocal fluorescence microscopy: Observation of the microtubule structure (α-tubulin) and distribution of GLUT4 (endogenous or GLUT4-EGFP) on intact cells or plasma membrane sheets.<br><br>2. 2-Deoxyglucose uptake assay: Measures glucose transport activity.<br><br>3. GLUT4 Recycling Assay: Measure the endocytosis and exocytosis of GLUT4 by c-Myc antibody marker (on GLUT4-myc-EGFP).<br><br>4. Live cell imaging: Monitoring the kinetics of GLUT4-EGFP in living cells.	1. Glucose absorption: After 15 minutes of insulin stimulation (measured in 5 minutes of absorption).<br><br>2. GLUT4 translocation (PM sheet): After 30 minutes of insulin stimulation.<br><br>3. Live cell imaging: Continuous observation at all times -7, 10, 27, 43, 60, 87, 104, 121 minutes.<br><br>4. Recycling assay: Measured at 0, 5, 10, 15, 20, 25, 30 minutes after the 2nd insulin stimulation.	1. GLUT4 (Translocation) experiment: Control group (Untreated) compared to Nocodazole/Taxol treatment group (each group compared baseline and insulin state).<br><br>2. Glucose absorption experiment: The group only pretreated Nocodazole (rinsed when measuring) compared to the group that maintained Nocodazole during the measurement process.	Microtubule disruption or stabilization does not significantly affect insulin-induced GLUT4 translocation. Nocodazole reduces glucose uptake because of its direct impact on GLUT4, which is not related to translocation. The depolymerization of microtubules does not slow down the intrinsic GLUT4 intake, but the intrasex vesicles are trapped in the paramembrane region, not entering the perinuclear cavities to be classified. However, these GLUT4 sacs still respond fully to the second insulin stimulation.	Microtubes	None	The study shows that microtubules do not need to produce insulin-stimulated GLUT4 proliferation or early exit and entry reactions, but mainly support long transport from the membrane to the perinucleus. If the microtubule is broken, GLUT4 will be trapped in the submembrane vesicles but still maintain its ability to respond to insulin.	Translocation										Unclear risk (Not reported)	Unclear risk (Not reported)	Low risk	Unclear risk (Not reported)	Unclear risk (Not reported)	Low risk
A.418	10.1128/MCB.23.13.4611-4626.2003	Nish Patel, 2003	Canada	Studying insulin-induced actin restructuring will direct the location of signaling molecules, especially the lipid product of PI3K, which facilitates GLUT4 to bind to the plasma membrane.	L6 myotubes express GLUT4myc.	Insulin: Cells were stimulated with 100 nM insulin.<br>Latrunculin B (LatB): Treated at concentrations of 1–5 μM to depolymerize actin filaments.<br>Wortmannin: Treated at a concentration of 100 nM to inhibit PI3K (phosphoinositide 3-kinase).<br>GFP-PH/Akt expression: Cells were transfected with a plasmid encoding the GFP-tagged Pleckstrin Homology (PH) domain of Akt to detect intracellular PI(3,4,5)P3 in live cells.<br>Swinholide A: An alternative actin-disrupting agent used for comparison with LatB.<br>Jasplakinolide: An actin-stabilizing agent (actin polymerizing agent).	1. Live-cell confocal fluorescence microscopy: Real-time monitoring of GFP-PH/Akt kinetics and actin restructuring.<br><br>2. Immunofluorescence: Observe the distribution of actin, GLUT4, p85 (subunit of PI3K), and PI(3,4,5)P3.<br><br>3. Biochemical fractionation: Cell separation to measure the amount of p85 and PI3-kinase activity binding to the cell skeleton.<br><br>4. Immunoblotting: Quantification of proteins and phosphorylation levels (Akt).	1. Protein/Actin Positioning (immunofluorescence): 10 minutes after insulin stimulation.<br><br>2. GLUT4myc (Translocation assay): 30 minutes after insulin stimulation.<br><br>3. Live-cell imaging: Continuous observation, taking images every 10-30 seconds, from 2 minutes before to 11 minutes after adding insulin	1. Comparison of baseline and insulin stimulation/processing and non-processing of inhibitors<br><br>2. Comparison of PI3-K isoforms: Comparison of the repositioning of the p110α and p110β catalytic subunits of PI3-K in the presence of insulin<br><br>3. Comparison of Downstream Effector Proteins (Effectors): Comparison of the repositioning of Akt-1 and atypical protein kinase C ζ (aPKCζ) in the presence of insulin.<br><br>4. Comparison of the role of PI (3,4,5)P₃ in actin restructuring and GLUT4 translocation:<br>- Cells expressing low concentrations of GFP-PH-GRP1 compared to cells expressing high concentrations of GFP-PH-GRP1 or GFP-PH-Akt<br>- GFP-PH-GRP1 (WT) expressing cells compared to GFP-PH(K273A)-GRP1 mutant expressing cells.	Insulin makes IRS-1 and PI3K p110α focus on the reconstructed actin regions. Akt-1 also enters these regions, but PKCζ does not. PI(3,4,5)P₃ is also concentrated to the periphery and is located right in this actin network. When GFP-PH-GRP1 is overexpressed, both actin restructuring and GLUT4 onto the membrane are blocked, suggesting that PI (3,4,5)P₃ is required for these two processes.	Actin, PI 3-kinase, PI (3,4,5)P₃	Stimulation	The restructured actin filaments serve as a scaffolding to organize space for insulin signaling (concentrating PIP3 at defined sites under the membrane), which is needed to regulate GLUT4 into the plasma membrane.	translocation										Unclear risk (Not reported)	Unclear risk (Not reported)	Low risk	Unclear risk (Not reported)	Unclear risk (Not reported)	Low risk
A.425	10.1007/s00424-011-1008-4	Deborah L. Grainger, 2011	UK	Investigate the involvement of lipid phosphoinositide PI5P in insulin-stimulated glucose uptake in the muscle tubes (myotubes) of the L6 skeletal muscle cell line.	Myotubes differentiated from the L6 myoblast cell line	1. Insulin: Stimulates cells at a concentration of 100 nM for 15 minutes (for glucose uptake) or over time (for lipids/signaling).<br><br>2. PIP4Kα overexpression: Transfection plasmid encodes the PIP4Kα enzyme to deplete intracellular PI5P.<br><br>3. Exogenous P5P: Introduce PI5P into cells with a histone carrier (Histone-PI5P complex) for 30 minutes.<br><br>4. Wortmannin: Administration at a concentration of 100 nM for 15 minutes before adding PI5P/insulin to inhibit PI3K.	1. 2-Deoxyglucose uptake assay: Measures glucose transport activity.<br><br>2. Mass assay for PI5P: Quantify PI5P by radioactive enzyme method (using recombinant PIP4Kα and [γ-32P]ATP).<br><br>3. HPLC (High Performance Liquid Chromatography): Analysis of other phosphoinositides (such as PI(3,4,5)P3).<br><br>4. Immunoblotting (Western Blot): Measures the degree of phosphorylation of Akt (pSer473).<br><br>5. Immunofluorescence & Colorimetric assay: Measure GLUT4-myc translocation to the cell surface.	1. PI5P: Measured at 0, 5, 10, 15, 20 minutes after insulin stimulation.<br><br>2. Glucose Absorption: Incubate for 20 minutes with insulin or PI5P, then measure absorption for 10 minutes.<br><br>3. GLUT4 Translocation and Cell Signaling (Akt): Measured after 20 minutes of processing.<br><br>4. PI(3,4,5)P3: Measured at 0, 5, 10, 15, 20 minutes.	1. Insulin Stimulation: Basal Cells (Basal/Unstimulated) vs. Insulin Stimulating Cells (100 nM).<br><br>2. PIP4Kα overexpression: PIP4Kα overexpressed cells vs. LacZ expression controls.<br><br>3. Exogenous PI5P: PI5P Processing Cell (5 μM) vs. Carrier Control (Carrier 3) vs. Non-Processing.<br><br>4. Lipid Specificity: PI5P Processing Cells vs. PI3P Processing Cells.<br><br>5. Signal Dependents (Inhibitors): PI5P + Inhibitors (Wortmannin/Herbimycin A) vs PI5P + Solvent Control (DMSO).	Insulin transiently increases PI5P levels in cells. When PIP4Kα enzyme expression is increased, this increased amount of PI5P disappears, loses the ability to increase glucose uptake, and also loses the PIP3 peak produced by insulin. Conversely, if PI5P is introduced from the outside into muscle cells (without insulin), PI5P increases Akt phosphorylation, pulls GLUT4 from the intracellular store to the plasma membrane and increases glucose uptake. The action of this PI5P requires the activity of Tyrosine kinase and PI3-kinase.	PI5P, PIP4Kα	PI5P: excitation<br>PIP4Kα: inhibition	Insulin stimulates the production of PI5P, which is essential for amplifying or maintaining the activity of PI3-kinase through Tyrosine kinase. As a result, the cell produces a high PIP3 peak, activates Akt, pulls GLUT4 out of the membranes and increases glucose uptake	Signaling	Unclear risk (Not reported)	Low risk	Unclear risk (Not reported)	Unclear risk (Not reported)	Unclear risk (Not reported)	Unclear risk (Not reported)	Unclear risk (Not reported)	Low risk	Low risk	Unclear risk (Not reported)	Unclear risk (Not reported)	Low risk	Unclear risk (Not reported)	Unclear risk (Not reported)	Low risk
A.427	10.1371/journal.pone.0212219	Nobuyuki Takenaka, 2019	Japan	Investigate the role of Akt2 in insulin-induced Rac1 activation and glucose uptake in rat skeletal muscle.	Musculoskeletal fibers from the gastrocnemius muscle of male C57BL/6 mice (conventional wild-type mice)	1. Insulin: Intravenous insulin injection (175.5 μg/kg by weight).<br><br>2. Knockdown Akt2 (siRNA): Introduces two duplex of Akt2-specific siRNAs into the leg abdominal muscles by electroporation.<br><br>3. Overexpression of activator proteins:<br>- Myr−p110α (permanently active PI3K form),.<br>- Rac1(G12V) (permanently active Rac1 form),.	1. Quantitative RT-PCR & Immunoblot: To effectively determine the knockdown of mRNA and protein levels of Akt2.<br><br>2. Immunofluorescent microscopy: On isolated muscle fibers and frozen slices to locate and quantify the signal.<br><br>3. In situ activation assay (Overlay assay): Use specific binding probes (GST-POSH for Rac1-GTP and GST-Sec5 for RalA-GTP) to detect the activity patterns of Rac1 and RalA.<br><br>4. GLUT4 reporter assay: Measure the ratio of the fluorescence intensity of Myc (surface) to GFP (total) from the GLUT4myc7-GFP structure to assess the degree of GLUT4 transfer to the sarcolemma membrane.	Analyses were performed 5 days after electrophoresis gene/siRNA transfer. The mice were fasted for 16 hours before insulin injection and muscle samples were collected immediately afterwards (the sample fixation time after insulin injection was 40 minutes for single muscle fibers).	1. siRNANC control: Mouse muscle treated with non-targeted siRNA (NC siRNA) compared to mouse muscle treated with anti-Akt2 siRNA (#1 and #2).<br><br>2. Insulin Stimulation: NC siRNA + Insulin vs. Akt2 siRNA + Insulin (to measure Rac1, RalA, and GLUT4 translocation).<br><br>3. PI3K activation: NC siRNA + Myr−p110α vs. Akt2 siRNA + Myr−p110α (to prove Akt2 is downstream PI3K).<br><br>4. Activation of Rac1: NC siRNA + Rac1(G12V) vs. Akt2 siRNA + Rac1(G12V) (to prove that Akt2 is upstream of Rac1),.	Knockdown Akt2 causes complete loss of Rac1 activation due to insulin or due to PI3K continuous activation mutations, RalA is also not activated, and GLUT4 cannot transfer to the membrane. If Rac1 is activated directly (by a continuous activation mutation), GLUT4 still releases the membrane normally even if Akt2 is knocked down. Demonstrates that Rac1 is located downstream of Akt2 and is necessary for the activation of RalA and the release of GLUT4 to the membrane in skeletal muscle.	Akt2, Rac1	Stimulation	Confirmation of the signal sequence in skeletal muscle: Insulin → PI3K → Akt2 → Rac1 → RalA → GLUT4 Translocation.<br><br>The study refutes the old hypothesis that Akt2 and Rac1 work in tandem.	Signaling	Unclear risk (Not reported)	Unclear risk (Not reported)	Unclear risk (Not reported)	Unclear risk (Not reported)	Unclear risk (Not reported)	Unclear risk (Not reported)	Unclear risk (Not reported)	Unclear risk (Not reported)	Low risk	Unclear risk (Not reported)	Unclear risk (Not reported)	Low risk	Unclear risk (Not reported)	Unclear risk (Not reported)	Low risk
A.429	10.1210/me.2014-1353	Hye Jeong Lee, 2015	Korea	To investigate the effects of irisin on glucose uptake and to identify the mechanisms of molecular signaling involved (specifically AMPK) in skeletal muscle cells in order to understand its role in hypoglycemia.	1. L6 rat myoblast cells have differentiated into myotubes.<br><br>2. Primary myoblasts isolated from the front and hind legs of mouse pups.	1. Irisin: Stimulation of cells (L6 myotubes/primary myoblasts) with recombinant irisin, mainly at an optimal concentration of 62 ng/mL.<br><br>2. Inhibitors:<br>- Compound C (2 μM): Inhibits AMPK.<br>- SB202190 (5 μM): Inhibits p38 MAPK.<br>- N-acetyl-cysteine (NAC): ROS scavenger (ROS scavenger).<br>- STO-609 (2 μM): Inhibits CaMKK (on primary cells).<br>- BAPTA: Ca2+ blocker (on L6 cells).<br><br>3. siRNA: Knockdown proteins p38 MAPK and AMPKβ2.<br><br>4. Insulin: 100 ng/mL.	1. Glucose absorption measurement: Using the radioactive isotope 2-deoxy-D-[H3] glucose.<br><br>2. Western blot: Analysis of protein and phosphorylation levels of AMPK, ACC, p38 MAPK, myogenin, β-actin.<br><br>3. ROS measurement: Using DCF-DA fluorescence indicator and confocal microscope.<br><br>4. Intracellular Calcium Measurement: Using Fluo-3 AM fluorescence indicator and concocular microscopy.<br><br>5. GLUT4 displacement measurement: Using a colorimetric absorption assay with anti-myc antibodies on the cell surface.	1. Glucose absorption: Incubate irisin for 15, 30, 60, 180 minutes (optimal at 30-60 minutes).<br><br>2. Signal (Western blot): 0, 15, 30, 60, 180, 360 minutes.<br><br>3. ROS/Calcium: Measure between 0-60 seconds or after 1 hour of incubation.	1. Irisin vs. Basal: Irisin-stimulated cells vs. untreated cells (basal condition)<br><br>2. Dosage effect: Compare different irisin concentrations (up to 62 ng/mL) to determine the optimal dose.<br><br>3. Functional inhibition: Irisin + Inhibitors/siRNAs: irisin in the presence of Compound C (AMPK inhibition), SB202190 (p38 MAPK inhibition), NAC (ROS blocker), STO-609 (CaMKK inhibitor), or siRNA (AMPKβ2 or p38 MAPK) compared to irisin alone.<br><br>4. siRNA control: Transgenic cells with specific siRNA compared to cells transgenicated with nontargeted control siRNA.<br><br>5. Signal sequence (Ca2+ vs. ROS): Comparison of intracellular Ca2+ in the presence of NAC (blocking ROS) and ROS production in the presence of BAPTA (blocking Ca2+)	Irisin stimulates glucose uptake in muscle cells by generating ROS and increasing intracellular Ca^{2+}, thereby activating CaMKK and AMPK. AMPK then activates p38 MAPK, resulting in GLUT4 transfer to the plasma membrane.<br><br>When ROS, AMPK, CaMKK, or p38 MAPK are inhibited, the full glucose uptake effect of irisin is lost, demonstrating that the ROS–Ca^{2+}–AMPK–p38 MAPK signaling axis is required for this effect.	Irisin	Stimulation	Irisin increases intracellular Ca^{2+} concentrations → activates CaMKK → activates AMPK → produces ROS → activates p38 MAPK → promotes GLUT4 transfer to cell membranes → increases glucose uptake.<br>(Research identifies the mediating role of ROS and Calcium in the Irisin-activated AMPK pathway)	Signaling										Unclear risk (Not reported)	Unclear risk (Not reported)	Low risk	Unclear risk (Not reported)	Unclear risk (Not reported)	Low risk

A.430	10.1074/jbc.M306782200	Sun Sik Bae, 2003	USA	Determine whether the phenotypic differences between Akt1/PKBα and Akt2/PKBβ deficient mice are due to tissue-specific differences in the expression of isoforms or to the distinct intrinsic signaling capacity of these two proteins.	1. Primary adipocytes isolated from wild mice (WT) and Akt2-deficient mice.<br>2. Primary mouse embryonic fibroblasts (MEFs) and immortalized lines are differentiated into fat cells from genotypes: WT, Akt1 deficiency, Akt2 deficiency, and both.<br>3. Brown preadipocytes isolated from mice.	1. Insulin: Stimulates fat cells with 100 nM insulin (to measure GLUT4 and GLUT1 translocation). In some experiments, insulin was used at different concentrations (0 to 100 nM) to measure glucose uptake on primary fat cells.<br>2. Akt (Re-expression): Akt2/PKBβ or Akt1/PKBα expression (both of which tag Hemagglutinin [HA]) by retroviral vector into 2KO cells to test the rehabilitation of hexose uptake and GLUT4 translocation.<br>3. Differentiation: Cell treatment with differentiated mixtures (insulin, dexamethasone, isobutylmethylxanthine, and Troglitazone) to induce differentiation into fat cell walls.	1. Hexose/Glucose Absorption Measurement: Use 2-Deoxy-D-[glucose-14C] with radioactive tracers to measure glucose uptake<br>2. GLUT4/GLUT1 displacement analysis (Plasma membrane sheet assay/Immunocytochemistry)<br>3. GLUT4/GLUT1 and caveolin fluorescent immunosuppression staining on plasma membrane plates or intact cells, analyzed by confocal microscopy.<br>4. Western Blotting: Determines the level of protein expression (Akt1, Akt2, C/EBPα, aP2, GLUT4...).	1. Hexose/Glucose Absorption: 20 minutes (on immortalized MEF/adipocytes) or 30 minutes (on primary fat cells) after insulin treatment.<br>2. GLUT4/GLUT1 translocation: 20 minutes after 100 nM insulin exposure.<br>3. Experiments on differentiated cells: Experiments are performed 8–10 days after the initiation of fat cell differentiation.	1. Genotype: WT cells vs. deficiency cells (1KO, 2KO, DKO).<br>2. Treatment: Unprocessed cells (Basal/-) vs. Insulin processing cells (+).<br>3. Rescue: 2KO cells express the hollow vector (2KO-Vec) compared to 2KO cells that reexpress Akt1 (2KO-Akt1) or Akt2 (2KO-Akt2).	Akt2 is an essential isoform for insulin signaling that leads to sugar absorption. Primary adipocytes, differentiated MEFs, and Akt2-deficient brown fat cells all drastically reduced glucose uptake and decreased GLUT4 (and also GLUT1) translocation, while Akt1 deficiency had almost no effect. Only reexpression of Akt2 restores a full response to insulin, while Akt1 does not, confirming its specific role	Akt2/PKBβ and Akt1/PKBα	Stimulation	On primary adipocytes, differentiated MEF and brown fat cells, insulin signaling specific for glucose uptake and GLUT4 translocation is dependent on Akt2/PKBβ, Akt1 is irreplaceable, even when overexpressed, suggesting that this specificity is not merely due to expression levels or cellular context.	Signaling										Unclear risk (Not reported)	Unclear risk (Not reported)	Low risk	Unclear risk (Not reported)	Unclear risk (Not reported)	Low risk	
A.434	10.2337/db10-0233	Lauritzen HPMM, 2010	USA and Danmark	Use a novel imaging system (intravital imaging) to clarify the kinetics of GLUT4 translocation induced by muscle contraction in skeletal muscle in live mice and AMPKα2 function during this process	1. Male rats Imprinting Control Region (ICR) (7-10 weeks old).<br>2. Muscle-specific AMPKα2 inactivated transgenic mice on FVB and control mice, respectively.<br>3. Quadriceps muscle fibers of live mice.	1. Contraction: Electrical stimulation is applied directly to the muscle fibers to cause single twitch contractions. There are two processes:<br>- High voltage: Frequency 2 Hz, duration 60 ms, voltage 1.1–3.0 V, 3 waves x 5 minutes, rest 90 seconds.<br>- Low voltage: Frequency 2 Hz, duration 60 ms, voltage 0.1–0.9 V, 3 waves x 10 minutes, rest 90 seconds.<br>2. Stimulation by AICAR: Intravenous administration of AICAR (AMPK activator) at a bolus dose of 1 g/kg.<br>3. Transfection: Shoot the GLUT4-EGFP gene into the quadriceps muscles with a gene gun.	1. Confocal imaging: Time-lapse images on live animals for GLUT4-EGFP observation.<br>2. Quantitative image analysis: Using Metamorf software to measure the fluorescence of GLUT4-EGFP at sarcolemma, t-tubules, and intracellular vesicle depots.<br>3. T-tubule staining: Use intravenous Sulforhodamine B dye to locate the t-tubule.	1. For muscle contraction: Images are collected at t = 0 (before contraction), at the end of each contraction episode (t = 5, 10, 15... minutes depending on the regimen), and every 10 minutes after cessation of contraction until 130 minutes (recell reentry phase).<br>2. With AICAR: Images are collected every 10 minutes within 240 minutes after injection.	1. Experiment comparing the strength of muscle contraction affecting GLUT4 displacement: high-voltage group with low-voltage group<br>2. Experiment to evaluate the role of AMPKα2 in GLUT4 translocation due to muscle contraction: rabies-type group and AMPKα2 inactivated transgene group (both electrical stimulation groups)<br>3. Experiment to evaluate the role of AMPKα2 in AICAR-induced GLUT4 translocation: the rabies-type group and the AMPKα2-inactivated transgenic group (both intravenous AICAR groups)	Muscle contraction increases GLUT4 out of both sarcolemma and t-tubules with the same kinetics, the increase being intense of contraction. GLUT4 only begins to re-internalize after 10 minutes of rest and is completed after ~130 minutes. AICAR causes similar translocation, but unlike muscle contraction, this response is AMPKα2-dependent.	AMPKα2, AICAR	Stimulation (for insulin, not for muscle contractions)	The study found that AMPKα2 is not a mandatory factor for GLUT4 translocation due to muscle contraction in vivo, refuting the previous hypothesis. At the same time, GLUT4 translocation during muscle contraction occurs simultaneously in both sarcolema and t-tubules, as opposed to insulin signaling, which causes delays in t-tubules.	Translocation	Unclear risk (Not reported)	Low risk	Unclear risk (Not reported)	Unclear risk (Not reported)	Unclear risk (Not reported)	Unclear risk (Not reported)	Unclear risk (Not reported)	Unclear risk (Not reported)	Unclear risk (Not reported							
A.436	10.1074/jbc.M115.658815	Sophie Trefely, 2015	Australia and USA	Screening identifies new kinases that are involved in regulating or interfering with the insulin/IGF-1 signaling pathway (ISP), determining the role of PFKFB3 and glucose metabolism on insulin signaling.	1. HA-GLUT4-HeLa stable expression HeLa cells (HA-GLUT4-HeLa cells) used for large-area screening.<br>2. 3T3-L1 mouse adipocytes (3T3-L1 adipocytes) used to verify the mechanism.<br>3. HEK293 cells used for overexpression experiments.	1. Large-area screening: Transfection with siRNA library targets 779 kinases in humans.<br>2. Stimulation: Insulin or IGF-1 (100 ng/ml or other concentrations depending on the experiment)<br>3. PFKFB3 intervention: Using siRNA to knock down PFKFB3, using 3PO (100uM) pharmacological inhibitors, overexpression (overexpression): PFKFB3 (plasmid Flag-PFKFB3).<br>4. Glycolysis regulation: Use UK-5099 (inhibitor of pyruvate transport into mitochondria - MPC) use CHC (inhibitor of lactate transport - MCT1) to inhibit glycolysis, supplement extracellular Lactate or agonist GPR81 (3-HBA, 3,5-DHBA).	1. GLUT4 displacement measurement: Using a high-throughput image analysis system (IN Cell Analyzer) to detect HA-GLUT4 on the cell surface.<br>2. Glucose absorption measurement: Using the radioactive isotope 3H-2-deoxyglucose.<br>3. Metabolic measurement: Lactate concentration (extracellular/intracellular), extracellular acidification rate (ECAR - Seahorse), Fructose-2,6-bisphosphate (F2,6BP) level measurement.<br>4. Western Blot: Evaluate the degree of phosphorylation of signaling proteins (Akt, AS160, GSK3, S6K).	1. GLUT4/Signal Transfer: Measured after 15-20 minutes of stimulation with Insulin/IGF-1.<br>2. SiRNA efficacy: 72 hours (HeLa) or 96 hours (3T3-L1) after transfection.<br>3. Inhibitor Treatment: Incubate for 30 minutes (with 3PO) or 2 hours (with UK-5099/CHC) before insulin stimulation.	1. Kinome Screening (HeLa) Target siRNA (Target siRNA) vs. Non-Targeted SIRNA (NTC) Cells.<br>2. Knockdown PFKFB3 (3T3-L1): PFKFB3 siRNA (siPFK) vs. Scrambled siRNA (scr) Infected Cells.<br>3. Drug PFKFB3 inhibition (3T3-L1): 3PO inhibition processing cells vs. control cells (DMSO).<br>4. PFKFB3 (HEK293) overexpression: PFKFB3 plasmid transfer cells vs. hollow vector (Vec) transfer cells.<br>5. Lactate Impact (3T3-L1): Lactate Processing Cell (25 mM) vs. Control Cell (NaCl).<br>6. Glycolytic Flow Regulation (3T3-L1): Inhibitor Processing Cell (UK-5099 or CHC) vs. Control Cell (Control).	PFKFB3, a positive regulator of glycolysis, is highly expressed in adipocytes and cancer cells, promoting GLUT4 translocation and Akt signaling. Inhibition of PFKFB3 decreases GLUT4 translocation and phosphorylation of Akt/AS160, while overexpression increases these reactions. Changes in intracellular glycolysis also affect Akt, while extracellular lactate does not. Demonstrated PFKFB3 promotes GLUT4 through intracellular regulation of glycolysis and Akt.	PFKFB3	Stimulation	Feedback loop relationship between metabolism and signaling:<br>- Akt-activated insulin signaling promotes glycemic sugar<br>- Glycolysis flow (lactate formation) regulated by PFKFB3 reverses to enhance Akt activation<br>This mechanism is not through the lactate receptor (GPR81) but through the directing of glucose into the lactate pathway (rather than intramitochondrial oxidation), which can affect the redox state or intracellular metabolites acting on Akt.	Signaling										Unclear risk (Not reported)	Unclear risk (Not reported)	Low risk	Unclear risk (Not reported)	Unclear risk (Not reported)	Low risk	
A.439	10.1007/s10863-012-9438-z	Xue-Qi Zeng, 2012	China	The goal of this study was to explore the effect of NYGGF4 (a gene associated with obesity) on the insulin sensitivity of skeletal muscle cells	C2C12 differentiated mouse musculoskeletal cells	1. Gene Knockdown: Use shRNA (pGPU6-NYGGF4-shRNA) to silence the expression of NYGGF4.<br>2. Insulin Stimulation: Treat cells with 100 nM concentration of insulin.<br>3. Inhibitor use: Cell treatment with PI3K (LY294002) inhibitor at a concentration of 30 μmol/L.	1. Quantify gene expression levels: Use Real-time PCR to measure the mRNA level of NYGGF4.<br>2. Glucose absorption measurement: Using the radioactive isotope 2-Deoxy-D-[3H] glucose.<br>3. Western blotting: Measures the protein level and phosphorylation state of signaling molecules (GLUT4, IR, IRS-1, AKT).	1. Differentiation: Cells are harvested or processed after 7 to 10 days of differentiation.<br>2. Inhibitor/Insulin Treatment: Incubate with LY294002 for 30 minutes, then incubate with insulin for 30 minutes.<br>3. Glucose absorption measurement: Measure within 10 minutes after adding radioactive glucose.	1. Comparison of knockdown efficacy: Blank cells, negative control (NC) transfer cells, and NYGGF4 shRNA vector transfer cells (NYGGF4 RNAi).<br>2. Comparison of glucose uptake: NC cells vs. NYGGF4 RNAi cells under the following conditions: basal (basal), insulin-stimulating (ins), and insulin + PI3K inhibitors (ins + LY294002).<br>3. Signaling/GLUT4 Comparison: NC cells vs. NYGGF4 RNAi cells with or without insulin.	Knockdown NYGGF4 increases glucose uptake and insulin-induced GLUT4 translocation in C2C12 (PI3K-dependent) cells. At the same time, NYGGF4 silencing strongly activates the phosphorylation of IRS-1 and Akt, suggesting that NYGGF4 inhibits glucose transport via the IRS-1/PI3K/Akt pathway.	NYGGF4 (gen)	Inhibition	Knockdown NYGGF4 increases glucose transport in muscle cells through activation of the IRS-1/PI3K/AKT insulin signaling pathway	Signaling										Unclear risk (Not reported)	Unclear risk (Not reported)	Low risk	Unclear risk (Not reported)	Unclear risk (Not reported)	Low risk	
A.441	10.3892/mmr.2011.443	Rui Cheng, 2011	China	Study of early oncogenic antigen involvement 1 (EEA1) and/or PI3K pathway in the molecular mechanisms behind the impact of transmembrane protein 4 (STEAP4) on insulin sensitivity of human fat cells	Human preadipocytes are cultured and differentiated into adult fat cells.	1. Gene Knockdown: Use shRNA-containing retroviruses to reduce the expression of STEAP4 (shSTEAP4) and EEA1 (shEEA1).<br>2. Overexpression: Use retrovirus to overexpress STEAP4.<br>3. Insulin Stimulation: Treat cells with insulin concentrations of 100 nM or 50 nM (during differentiation).	1. Glucose Absorption Measurement: Using fluorescent glucose derivative 2-NBDG to monitor glucose uptake into living cells.<br>2. GLUT4 displacement measurement: Using the GLUT4-7Myc-GFP reporter system and detecting by fluorescent anti-Myc antibody (PE-conjugated).<br>3. Western blotting: Measures the level of protein expression (STEAP4, EEA1, Akt, p-Akt).<br>4. FRET (Fluorescence Resonance Energy Transfer): Analysis of protein-protein interactions between STEAP4 and EEA1 in living cells.	1. Differentiation: Cells are evaluated after 14-17 days of differentiation into mature fat cells.<br>2. Insulin Treatment: Measure after 30 minutes of stimulation with insulin.<br>3. Glucose Absorption Measurement: The reaction ends after 15 minutes of incubation with 2-NBDG.<br>4. FRET: Measured after 24 hours of co-transfection.	1. STEAP4 Experiment: Comparison with and without insulin.<br>- Blank control (non-viral cells).<br>- Negative control (non-targeted shRNA/GFP virus-infected cells).<br>- Intervention group (shSTEAP4 - cells infected with the STEAP4 shRNA virus).<br>2. EEA1 and STEAP4 Experiments: Comparison with and without insulin.<br>- Blank control<br>- Negative control group<br>- Intervention group 1: shEEA1 (Knockdown EEA1).<br>- Intervention group 2: shEEA1 + STEAP4 (Knockdown EEA1 combined with STEAP4 Overexpression).	Knockdown STEAP4 reduces glucose uptake and insulin-induced GLUT4 translocation in the adipocyte, through reduced phosphorylation of Akt. This effect works independently of EEA1, as STEAP4 does not affect expression, function, or interaction with EEA1.	STEAP4	Stimulation	STEAP4 promotes glucose uptake and GLUT4 translocation by maintaining or increasing Akt phosphorylation, which is completely independent of EEA1.	Signaling										Unclear risk (Not reported)	Unclear risk (Not reported)	Low risk	Unclear risk (Not reported)	Unclear risk (Not reported)	Low risk	
A.442	10.1242/jcs.258375	Black HL, 2022	UK and USA	Understanding the role of syntaxin-4 (Sx4) in involvement in GLUT4 translocation, adiponectin secretion, and glucose transport	Adipocytes of the 3T3-L1 lineage.	1. Gene Knockout: Syntaxin-4 (Sx4) gene removal using the CRISPR/Cas9 system.<br>2. Insulin Stimulation: Treat cells with insulin (typically 100 nM, or a concentration range of 0.02 to 100 nM) to stimulate glucose transport and GLUT4 translocation.<br>3. Foreign protein expression: GLUT4 expression tags HA and GFP (HA-GLUT4-GFP) to monitor translocation kinetics.<br>4. Use inhibitors: Use BAY-876 (inhibit GLUT1/GLUT4) and Wortmannin (inhibit cell entry).	1. Glucose absorption measurement: Use 2-deoxy-D-glucose (2DG) with radioactive markers.<br>2. Immunoblot Analysis (Western Blot): Measures the protein levels of GLUT4, Sx4, other SNAREs, and signaling proteins (pAkt, pERK).<br>3. Subcellular fractionation: To determine the location of GLUT4 and other proteins in membrane segments.<br>4. ELISA: Measures the amount of adiponectin secreted in the culture medium.<br>5. Flow cytometry (FACS): Analysis of recycling and translocation kinetics of HA-GLUT4-GFP on a single cell surface.	1. Signal Stimulation (Akt/ERK Phosphorylation): After fasting (2 hours), incubate with insulin (100 nM) for 15 minutes<br>2. Glucose Uptake (2DG Uptake): Insulin stimulation (100 nM) for 20 minutes and absorption measurement for 5 minutes<br>3. Measurement of GLUT4 Displacement (Maximal PM levels): Insulin stimulation (100 nM) for 45 minutes<br>4. Measurement of Adiponectin Excretion: Collect the culture medium after 24 hours of incubation in a serum-free medium.<br>5. Intracellular Measurement (Endocytosis Ken): After 45 minutes of insulin stimulation, add Wortmannin and measure between 0 and 60 minutes	1. Knockout phenotype evaluation: Sx4-KO cells vs. WT cells (under Basal and Insulin conditions).<br>2. GLUT4 kinetics (HA-GLUT4-GFP): Sx4-KO cells vs. WT cells (same HA-GLUT4-GFP expression): Wortmannin Processing Cells vs. Non-Processing (Insulin Background).<br>3. GLUT1/GLUT4 Differentiation: Insulin Stimulating Cells vs. Insulin Cells + 20 nM BAY-876 (performed on both WT and Sx4-KO).	Knockout Syntaxin-4 reduces glucose uptake due to insulin and adiponectin secretion by ~50%, and reduces total GLUT4 intake. However, the cell retains significant GLUT4 translocation, with only a slight reduction in the rate of propulsion and virtually no effect on cell entry, suggesting that Syntaxin-4 is not always a speed-limiting factor of GLUT4 to the membrane.	Syntaxin-4	Stimulation	Syntaxin-4 shows that it is not necessary for insulin-induced GLUT4 translocation (fusion still takes place in the absence of Sx4), but is important for maintaining total GLUT4 intake and ensuring effective adiponectin secretion.	Fusion										Low risk	Unclear risk (Not reported)	Low risk	Unclear risk (Not reported)	Unclear risk (Not reported)	Low risk	
A.448	10.1074/jbc.M111.253880	Paul Duffield Brewer, 2011	USA	Measuring the impact of AS160 knockdown on the constant rate of expulsion (kex), cell entry (ken), and release of Glut4 from retention in the active circulatory group in 3T3-L1 fat cells	3T3-L1 fat cells express HA-Glut4/GFP protein	Attenuation of AS160 (AS160 knockdown) using shRNA targeting AS160<br>Inhibition of phosphatidylinositol 3-kinase (PI 3-kinase) by LY294002 (LYi)<br>Inhibition of Akt (protein kinase B) by Akt inhibitor VIII/Akti-1/2 (Akti)	Flow cytometry (measurement of Glut4 surface and total); Uptake kinetics; Transition kinetics; Mathematical modeling.	After 45 minutes of insulin stimulation (for a steady state); and in a continuous time series from 0 to 300 minutes (for uptake and transition kinetic experiments).	The control group (Control shRNA/Scrambled shRNA) and compared the basal state (Basal) with the insulin-stimulated state (+/- inhibitor).	AS160 regulates the release of Glut4 from retention in the non-circulating/slow-circulating GSV reserve cavity into the active circulating population.<br>- A second pathway that relies on Akt and PI3 kinases but does not depend on AS160 regulates the rate of exit of Glut4 from the circulatory population.<br>- knockdown AS160 prepares vesicles (intracellular vesicles) for rapid membrane fusion after insulin stimulation	AS160	Inhibition	AS160 regulates the release of Glut4 from retention in the non-circulating/slow-circulating GSV reserve cavity into the active circulating population.	Signaling										Low risk (ATCC)	Unclear risk (Not r	Low risk	Unclear risk (Not rep	Unclear risk (No	Low risk	
A.449		Holly McDonough, 2017	USA	Investigate whether CHIP protein deficiency leads to disturbances of metabolic homeostasis, especially glucose/insulin signaling	Mouse lacks CHIP (CHIP -/- mice)<br>- Mouse skeletal muscle cell C2C12 (rabid type and CHIP deficient)<br>- Mouse embryonic fibroblasts (rabid and CHIP-deficient)<br>- Muscle fibers isolated from rabies-type or CHIP-deficient mice after fasting or glucose injections	Peritoneal Injection (IP) 20% D-glucose<br>- Insulin peritoneal injection (IP)<br>- Performing super-insulin-hyperglycemic clamping on adult rats	Glucose tolerance test (GTT); Insulin tolerance test (ITT); Hyperinsulinemic-euglycemic clamp; Measure the absorption of 2-deoxyglucose in skeletal muscle; OPD (Glut4 Surface) colorimetric test; Immunoblotting (Western blot); Immunofluorescence; 2D-DIGE proteomic analysis.	In mice (in vivo): 0, 15, 30, 60, 120 minutes after injection (GTT/ITT); 40 minutes after glucose injection (2-deoxyglucose absorption).<br>In vitro (in vitro): 0, 30, 60, 90 minutes after insulin stimulation.	Wild-type mice vs. CHIP mice-/- (on both genetic backgrounds); C2C12 shCONT cells (control) compared to C2C12 shCHIP cells (CHIP deficiency).	CHIP is essential for optimal glucose homeostasis	CHIP	Stimulation	Role of CHIP (carboxyl terminus of Hsc/Hsp70-interacting protein) in regulating microtubule polymerization to support the migration of glucose transporter Glut4	Translocation	Unclear risk (Not r	Low risk	Unclear risk (Not r	Unclear risk (Not r	Unclear risk (Not r	Unclear risk (Not r	Unclear risk (Not r	Low risk	Low risk	Unclear risk (Not r	Unclear risk (Not r	Low risk	Unclear risk (Not rep	Unclear risk (No	Low risk	
A.451	10.1152/ajpendo.00597.2012	Hidemitsu Sakagami, 2014	Japan	Investigate the role of hypoxia inductor factor-1α (HIF-1α) in insulin-stimulated glucose uptake in skeletal muscle cells	C2C12 myoblast	C2C12 myoblasts were stably transfected with a short-hairpin RNA (shRNA) expression plasmid to severely compromise the expression of HIF-1α<br>- Cells were generated expressing a chimeric CA-HIF-1 alpha protein to achieve hypoxia-independent transcriptional activity<br>- Wild-type and modified C2C12 myoblasts were differentiated into myotubes (skeletal muscle cells) using horse serum and then serum-starved before assays	Glucose uptake assay (2-deoxy-D-[3H] glucose uptake); Immunoblot (Western Blot); Immunoprecipitation; Subcellular fractionation for separation of plasma membrane (PM) and low-density microsomal (LDM); Real-time PCR.	30 minutes after insulin stimulation (for glucose uptake and translocation); 5 minutes after stimulation (for phosphorylatation); Monitor protein expression over time series (0, 5, 10, 30, 60, 120 minutes).	C2C12 Wild-type (WT) cells versus C2C12 knockdown HIF-1α (ΔHIF-1α); WT mice vs. HIF−1α mice +/−. Cells express CA-HIF-1α compared to WT; Basal vs. Stimulation (Insulin/AICAR).	HIF-1 alpha is a determinant for GLUT4-mediated glucose uptake in the skeletal muscle cells	HIF-1 alpha	Stimulation	Phosphorylation of AS160 and GLUT4 trafficking are increased in cells expressing constitutively active HIF-1.	Signaling	Unclear risk (Not r	Low risk	Unclear risk (Not r	Unclear risk (Not r	Unclear risk (Not r	Unclear risk (Not r	Unclear risk (Not r	Low risk	Low risk	Low risk (RIKEN)	Unclear risk (Not r	Low risk	Unclear risk (Not rep	Unclear risk (No	Low risk	
A.452	10.1083/jcb.200709108	Dumaine Williams, 2008	USA	To functionally identify the site of action of the vesicle-associated membrane protein (VAMP) family of R-SNAREs	3T3L1 adipocytes	Gene Silencing: Using specific siRNAs to knockdown (reduce expression) of each VAMP protein (VAMP2, 3, 4, 5, 7, 8).<br>Reporter: Expression of myc-GLUT4-GFP reporter protein.<br>Stimulation: Cell treatment with Insulin (100 nM) or Osmotic Shock (Sorbitol 600 mM).	confocal fluorescent microscopy<br>- Plasma membrane sheet assay<br>- Immunofluorescence and image analysis<br>- Immunoblotting	Knockdown Effect: 72 hours after siRNA infusion.<br>Translocation: 72 hours after siRNA infusion, measured immediately after 30 minutes of insulin or sorbitol stimulation.<br>Biosynthetic sorting: SiRNA infusion for 60 hours, then myc-GLUT4-GFP infusion and measurement after the next 12 hours.<br>Endocytosis: Measured at different times after rewarming at 37°C (up to 360 minutes).	The control group was 3T3L1 fat cells transfused with "random siRNA" (siRNA with a non-specific random sequence) and the basal (non-stimulated) group, which was used to compare with the siRNA knockout groups	The requirement of VAMP2 and VAMP7 for insulin and osmotic shock trafficking from the vesicle storage sites, respectively, and fusion with the plasma membrane.	VAMP2<br>VAMP7	Stimulation	VAMP2 and VAMP7 required for GLUT4 translocation and fusion	Translocation and fusion										Low risk (ATCC)	Unclear risk (Not r	Low risk	Unclear risk (Not rep	Unclear risk (No	Low risk	
A.457	10.1016/j.cmet.2010.12.005	Shuai Chen, 2011	The United Kingdom, The United States	To define physiological roles of AS160-Thr649 phosphorylation and 14-3-3 binding in glucose homeostasis, we substituted this residue by a nonphosphorylatable alanine by knockin mutation in mice.	_ AS160 Thr649Ala knockin mice<br>- epididymal fat pads<br>- Primary adipose cells and isolated soleus muscles	- The Thr649Ala mutation was introduced into exon 10<br>- Intraperitoneal (i.p.) injection of glucose (2 mg/g body weight)<br>- Insulin injection	- Immunoprecipitation – IP<br>- Immunoblotting analysis<br>- Measuring blood glucose, plasma insulin, adiponectin, leptin, and free fatty acids (FFAs)	GTT: Measure blood glucose at 0, 15, 30, 45, 60, 120 minutes after glucose injection.<br>Clamp: The metric is averaged during the 80- to 120-minute stabilization phase of insulin clamping.<br>Signaling: Measured after 20 minutes of insulin or saline injection.<br>In vivo uptake: Measured after 60 minutes of injecting glucose containing radioactive material.	Comparison between the group of mice carrying the Knockin mutation (KI) (especially homozygous) and the group of wild-type (WT) mice of the same age. In biochemical/transport experiments, a control-controlled comparison between baseline (Basal/Saline) and stimulated conditions (Insulin/Glucose) between the two genotypes.	Removal of Thr649 phosphorylation and 14-3-3 binding of AS160 leads to insulin resistance and systemic glucose intolerance, primarily due to reduced insulin-stimulated glucose transport and reduced GLUT4 migration in muscle	AS160	Stimulation	The phosphorylation of Thr649 and the 14-3-3 bonding of AS160 is required of GLUT4 translocation	Translocation	Unclear risk (Not r	Low risk	Unclear risk (Not r	Unclear risk (Not r	Unclear risk (Not r	Unclear risk (Not r	Unclear risk (Not r	Low risk	Low risk	Unclear risk (Not r	Unclear risk (Not r	Low risk	Unclear risk (Not rep	Unclear risk (No	Low risk	
A.458	10.1016/j.heliyon.2023.e15539	Narges Zolfaghari, 2023	Iran	To investigate the role of miR-96-5p in this signaling pathway.	In vitro: mouse retinal pigment epithelial cells (mRPE cells) and human umbilical venous endothelial cells (HUVECs).<br>In vivo: NMRI-breed male rats (6-8 weeks old).	High Glucose (HG) Medium: Cell culture in a high glucose medium (30 mM) to simulate diabetes.<br>Overexpression: Using the mmu-miR-96 gene-carrying AAV-2 virus vector to increase miR-96-5p expression (both in vitro and in vivo intraocular injection).<br>Inhibition: Use antagomiR-96 to inhibit miR-96-5p.<br>Diabetes Model: Streptozotocin (STZ) injection to induce type 1 diabetes in mice.	RT-qPCR: Measures the level of gene expression (mRNA and miRNA).<br>Western blot: Measures the level of protein expression.<br>MTT assay: Measure cell proliferation.<br>Wound healing assay: Measures the ability of cells to migrate.<br>Tube formation assay: Measures the ability to form a vascular tube (angiogenesis).<br>TUNEL assay: Detection of apoptosis.<br>Immunohistochemistry & Whole-mount retina staining: Isolectin B4 staining to observe blood vessels and HE staining to measure the thickness of the retinal layers.	Cell proliferation (MTT): 24, 48, and 72 hours after transgene.<br>Wound healing: 24 hours after scratching.<br>Tube formation: 18 hours after incubation of HUVECs in a conditioned environment.<br>AAV injection mice: 2 weeks after intraocular injection.<br>STZ diabetic rat model: 10 weeks after STZ injection.	In vitro: Comparison of the control group (Untreated/GFP vector/Scrambled/Normal Glucose) and the intervention group (miR-96 vector/AntagomiR/High Glucose).<br>In vivo: Comparison of control mice (PBS/AAV-GFP) and laboratory mice (STZ-induced diabetes/AAV-miR-96).	The expression of miR-96-5p target genes associated with the INS/AKT/GLUT4 signaling pathway decreases after miR-96-5p is overexpressed.	miR-96-5p	Inhibition	The expression of miR-96-5p target genes associated with the INS/AKT/GLUT4 signaling pathway decreases after miR-96-5p is overexpressed.	Signaling	Unclear risk (Not r	Low risk	Unclear risk (Not r	Unclear risk (Not r	Unclear risk (Not r	Unclear risk (Not r	Unclear risk (Not r	Low risk	Low risk	Unclear risk (Not r	Unclear risk (Not r	Low risk	Unclear risk (Not rep	Unclear risk (No	Low risk	
A.460	10.1016/j.mce.2012.09.005	Li-Zhong Liu, 2013	China	Examine the morphological changes of microtubules and actins, as well as their roles in insulin signaling, GLUT4 translocation, and glucose uptake.	L6 skeletal muscle cells express GLUT4 tagged with c-myc (L6myc cells), which are differentiated into myotubes.	Stimulus: Insulin (100 nM).<br>Chemicals that inhibit/disrupt the cell skeleton: Nocodazole, Colchicine (disrupt microtubules); Jasplakinolide (JPL), Cytochalasin D (CD), Latrunculin B (LB) (actin interference).<br>Signal Inhibitor Chemical: Genistein (tyrosine kinase inhibitor), Wortmannin (PI3K inhibitor), U0126 (MAPK inhibitor), Pseudosubstrate (PKCζ inhibitor), API2 (Akt inhibitor).	Confocal laser microscopy: Observation of morphology and colocation of microtubules, actin, and PKCζ.<br>Western blot & Immunoprecipitation: Measures the degree of phosphorylation of signaling proteins (IRS1, Akt, PKCζ, ERK).<br>Cell fractionation: Separation of the plasma membrane to measure the distribution of PKCζ.<br>2-Deoxy-[3H]deoxyglucose uptake: Measures glucose uptake.<br>Densitometric assay: Quantify GLUT4myc on the cell surface by enzymatic colorimetry.	Morphology: 0, 1, 3, 5, 10 minutes after insulin stimulation.<br>Inhibitor treatment: 20-30 minutes or 1 hour (with genistein) before adding insulin.<br>Measure GLUT4 and glucose uptake: After 10 minutes of insulin stimulation.	Comparison between basal and insulin-stimulated state<br>Comparison between the control group and the group treated with cytoskeleton inhibitors or kinase inhibitors (Nocodazole, Colchicine, Wortmannin, etc.).	Insulin quickly leads to the clustering of microtubules from the ventral side to the dorsal side of the cell.<br>The microtubule fibers are rearranged to make space for the newly formed actin structures.<br>The disruption of the microtubule prevented insulin-stimulated actin restructuring and distal insulin signaling, and reduced surface GLUT4 and glucose uptake.<br>Although microtubules mediate actin restructuring through PKCζ, microtubule reorganization is dependent on tyrosine phosphorylation of insulin receptors; this mechanism differs from insulin-stimulated actin restructuring, which is dependent on the activity of PI3-kinase and PKCζ.<br>The microtubule network is necessary for insulin-stimulated actin signaling and restructuring in skeletal muscle cells.	Microtubules and actins	Stimulation	Microtubule clustering is an early occurrence event that depends on the activity of insulin receptor tyrosine kinase but not PI3K. Microtubules mediate the transport and activation of PKCζ at the plasma membrane. PKCζ then activates actin restructuring. Actin restructuring is dependent on PI3K and PKCζ.	Translocation										Unclear risk (Not r	Unclear risk (Not r	Low risk	Unclear risk (Not rep	Unclear risk (No	Low risk	

A.461	10.7554/eLife.83338	Jonas R Knudsen, 2023	Denmark	Investigate the role of microtubule networks on the location and intracellular transport of GLUT4 in human skeletal muscle and adult rats, as well as the effect of insulin resistance on this process.	Adult mouse muscle fibers: Flexor digitorum brevis (FDB) muscle (for observation of living cells), Soleus muscle, and Extensor digitorum longus (EDL) (for measuring glucose uptake).<br><br>Human muscle: Vastus lateralis muscle from healthy men.<br><br>Cell line: L6 myoblasts/myotubes.	Disruption of microtubules: Use of Nocodazole (Noco) or Colchicine.<br><br>Motor protein interference: Use Kinesore (Kinesin-1 modulator) or shRNA to knock down Kif5b (Kinesin-1 heavy chain).<br><br>Insulin Resistance Model:<br><br>In vitro: Treatment of muscle fibers with C2 ceramide.<br><br>In vivo: The mice were fed a high-fat diet (HFD).<br><br>Stimulant: Insulin (30 nM or 100 nM).	Structured Illumination Microscopy (SIM): A super-resolution microscope for observing microtube and GLUT4 structures.<br><br>Live-cell imaging: Live cell filming to monitor the movement of GLUT4-GFP along the microtubule.<br><br>Muscle-on-a-chip: Microfluidic system integrates electrochemical glucose sensors to measure glucose uptake in real time.<br><br>2-Deoxyglucose (2-DG) transport assay: Measures the transport of radioactive glucose.<br><br>Western blot: Analysis of phosphorylation signals (Akt, TBC1D4).	Microtubule disruption (Nocodazole): Short-term effects (5 minutes) vs. long-term effects (2 hours or 15 hours).<br><br>Recovery: 9 hours after Nocodazole removal.<br><br>Insulin Resistance Model: Feeding HFD for 10 weeks; C2 ceramide treatment for 2 hours.<br><br>Insulin stimulation: 15-30 minutes before imaging or cell lysis.	Time comparison: Noco 5 minutes (acute) vs Noco 2 hours (prolonged).<br><br>Condition Comparison: Basal (basic) vs Insulin.<br><br>Comparison of interventions: Control (DMSO) vs Nocodazole/Colchicine/Kinesore.<br><br>Pathological comparison: Mice eating regular food (Chow) vs Rats eating a high-fat diet (HFD); Control vs C2 Ceramide.<br><br>Genetic comparison: shScramble (control) vs shKIF5B (knockdown).	In adult skeletal muscle fibers, the microtubule network plays an essential role in the transport of GLUT4 inside muscle cells; It is more likely that it works to maintain a "storehouse" of GLUT4 that can be mobilized to the cell surface in the presence of insulin, via a transport mechanism mediated by kinesin-1.	Microtubule	Stimulation	The microtubule network is necessary for maintaining the distribution of intracellular GLUT4 compartmentalization through transport by Kinesin-1 (KIF5B).	Transposition	Unclear risk (Not n	Low risk	Unclear risk (Not n	Unclear risk (Not n	Unclear risk (Not n	Unclear risk (Not n	Unclear risk (Not n	Low risk		Low risk	Unclear risk (Not n	Low risk	Unclear risk (Not re	Unclear risk (No	Low risk
A.470	10.1016/j.jbc.2024.105782	Furong Liu, 2024	China	To fully address the role of Munc18c in membrane fusion, it is necessary to study whether Munc18c promotes the trans-SNARE assembly and membrane fusion under the regulation of a-SNAP and NSF	In vitro reconstituted liposomes (In vitro reconstituted liposomes). Details:<br><br>T-liposomes: Contains Syntaxin-4 and SNAP-23 (t-SNAREs of GLUT4).<br><br>V-liposomes: Contains VAMP2 (v-SNARE of GLUT4) or VAMP8.	Additional Regulatory Proteins: Munc18c (WT and mutant Munc18c-TolA), α-SNAP (WT and mutants F27S/F28S, K122E/K163E), NSF.<br><br>Ionic condition: Mg 2+ (NSF activated) or EDTA (NSF inhibitor).<br><br>Replace SNARE: Use VAMP8 instead of VAMP2 for specificity testing.	FRET-based lipid mixing assay: Measures lipid mixing (membrane fusion).<br><br>Content mixing assay: Measures the mixing of contents inside liposomes (using sulforhodamine B).<br><br>Liposome co-flotation assay: Examines protein binding to liposomes/SNAREs.<br><br>Trans-SNARE disassembly assay: Measures the level of resolution of the trans-SNARE complex.	Fusion assays (Lipid/Content mixing): Continuous monitoring for 60 minutes.<br><br>Disassembly assay: Measured at 0 minutes and 30 minutes.	α-SNAP inhibition experiment:<br><br>- None (t-SNARE + v-SNARE only)<br>- α-SNAP WT<br>- α-SNAP FS (hydrophobic mutant)<br>- α-SNAP KE (SNARE-binding mutant)<br><br>Munc18c rescue experiment:<br><br>- None<br>- α-SNAP<br>- Munc18c<br>- α-SNAP + Munc18c<br><br>NSF-containing experiment:<br><br>- NSF/α-SNAP (Mg^{2+} premix)<br>- NSF/α-SNAP (EDTA premix)<br>- Munc18c + NSF/α-SNAP (Mg^{2+}/EDTA)<br><br>SLP mechanistic experiment:<br>- Comparison of Munc18c WT with Munc18c-TolA (SLP-region mutant).	α-SNAP blocks the GLUT4 SNAREs-mediated membrane fusion. Munc18c interacts with t-SNAREs to displace α-SNAP, which overcomes the fusion inhibition. Furthermore, Munc18c shields the trans-SNARE complex from NSF/α-SNAP-mediated disassembly and accelerates SNARE- dependent fusion kinetics in the presence of NSF and α-SNAP. Munc18c protects the prefusion SNARE complex from α-SNAP and NSF, promoting SNARE-dependent mem- brane fusion through its SLP.	α-SNAP, Munc18c	α-SNAP: inhibition<br>Munc18c: excitation	Munc18c protects the trans-SNARE prefusion complex from resolution caused by NSF and α-SNAP. This mechanism is entirely dependent on the SNARE-like peptide (SLP) region in the 3a domain of Munc18c, which binds to t-SNAREs to prevent α-SNAP binding.	Fusion										Unclear risk (Not n	Unclear risk (Not n	Low risk	Unclear risk (Not re	Unclear risk (No	Low risk
A.471	10.1128/MCB.20.1.379-388.2000	Debbie C. Thurmond, 2000	USA	Examining the functional role of the interaction between Munc18c and Syntaxin4 in regulating the translocation of GLUT4 in 3T3L1 fat cells.	Differentiated 3T3-L1 adipocytes.	Overexpression: Munc18c (full-length), Syntaxin 4 (WT and ΔTM soluble form), peptide fragments of Munc18c (18c/pep1, 18c/pep2, 18c/pep3).<br><br>Microinjection: Synthetic peptide 18c/pep3 and scrambled peptide.<br><br>Stimulus: Insulin (100 nM).	Whole-cell immunofluorescence: Observation of whole-cell GLUT4/IRAP translocation (confocal microscopy).<br><br>Plasma membrane sheet assay: Analysis of protein integration into the plasma membrane on isolated membrane plates.<br><br>Plasma membrane competition assay: Measuring binding competitiveness with Syntaxin 4.	After transfection recovery: 18-24 hours.<br><br>After Insulin Stimulation: 30 minutes.	Syntaxin Rescue Experiment 4:<br>- Control (Vector)<br>- Syn4/WT (Wild-type)<br>- Syn4/ΔTM (Cytosolic domain)<br>- Munc18c<br>- Munc18c + Syn4/WT<br>- Munc18c + Syn4/ΔTM<br>Peptide Experiments:<br>- Vector (Control)<br>- Munc18c<br>- 18C/PEP1<br>- 18C/PEP2<br>- 18C/PEP3<br>Micro-injection experiments:<br>- Scrambled peptide (Control)<br>- 18C/Pep3 peptide	the interaction between Munc18c and syntaxin 4 is necessary for the integration of GLUT4 and IRAP storage vesicles into the plasma membrane, but not for insulin-stimulated cell surface transport and access	Munc18c, syntaxin 4	Stimulation	The interaction between Munc18c and Syntaxin4 is required for the fusion/integration of GLUT4-containing vesicles into the plasma membrane, but is not necessary for the transport and anchoring of the vesicle at the membrane. The 18c/pep3 peptide disrupts this interaction allowing the vesicle to travel to the membrane but is stuck in a docked state without being able to bind to the membrane.	Fusion										Unclear risk (Not n	Unclear risk (Not n	Low risk	Unclear risk (Not re	Unclear risk (No	Low risk
A.472	10.1074/jbc.M610818200	Matthew D'Andrea-Merrins, 2007	USA	determine the molecular properties of the interaction between Munc18c and syntaxin 4; specifically how the Munc18c-syntaxin 4 complex affects the formation of the SNARE core complex during the GLUT4 transport cycle.	Cells: Differentiated 3T3-L1 fat cells (3T3L1 adipocytes) and HEK293-S3 cells.	Mutagenesis: Expression of mutations of Syntaxin 4 (L173A/E174A [LE], I241A, D3R, I217A) and Munc18c (R240L) in cells.<br><br>Stimulus: Insulin (100 nM).<br><br>Gene expression: Cotransfection of fluorescent protein-coding plasmids (CFP, YFP, RFP, GFP) or epitope tag (Myc, Flag).<br><br>Photobleaching: Photobleaching of the cytoplasm or cell membrane to measure restorative kinetics (FRAP).	FRET (Sensitized Emission): Measures protein-protein interactions directly in living cells (3T3-L1 and HEK293).<br><br>Co-immunoprecipitation (Co-IP): Immunoprecipitation from HEK293 cytolytic fluid.<br><br>Confocal Microscopy/Imaging: Observation of GLUT4 (Myc-GLUT4-GFP) translocation and protein localization.<br><br>FRAP (Fluorescence Recovery After Photobleaching): Measures fluorescence recovery to evaluate the alignment/diffusion of SNAP23.	FRET/Imaging: 24 hours after electroporation/transfection.<br><br>Insulin: 30 minutes.<br><br>FRAP: Recovery time 1.5 minutes.	FRET (Intracellular Interaction) Experiment:<br><br>- Munc18c + Syntaxin 4 WT (Wild type).<br><br>- Munc18c + Syntaxin 4 LE (Open conformation).<br><br>- Munc18c + Syntaxin 4 binding mutations (I241A, I217A, D3R, ΔH3/TM).<br>Co-IP & FRAP (Complex Formation) Experiments:<br>- Syntaxin 4 WT + SNAP23 (+/- Munc18c).<br><br>- Syntaxin 4 LE + SNAP23 (+/- Munc18c).<br>GLUT4 (3T3-L1) Transport Experiment:<br>- Munc18c + Syntaxin 4 WT (+/- Insulin).<br><br>- Munc18c + Syntaxin 4 LE (+/- Insulin).<br><br>- Munc18c + Syntaxin 4 I241A (+/- Insulin).<br><br>- Munc18c + Syntaxin 4 LE+I241A (+/- Insulin).	_ Munc18c also binds to an affinity almost equivalent to that of a mutant form of syntaxin 4 in the open state<br>- both the N and C terminals of syntaxin 4 are required for Munc18c binding<br>- Munc18c blocks the recruitment of SNAP23 into wild syntaxin 4 but can bind to the 4LE–SNAP23 syntaxin complexes of the Q-SNARE group.<br>- Expression of syntaxin 4LE and Munc18c helps the GLUT4 vesicle to attach to the plasma membrane without the need for insulin. This effect was attenuated by reducing the Munc18c–syntaxin 4LE interaction with the I241A mutation, suggesting that Munc18c plays a role in supporting the docking process of the vesicles.<br>- The structural "opening" of syntaxin 4 — rather than the separation of Munc18c from syntaxin 4 — is the key event required for GLUT4 pocket docking.	Munc18c, syntaxin 4	Syntaxin 4: Stimulation<br>Munc18c: the role of conditioning	The transition of Syntaxin 4's configuration to an open conformation state is a key event to enable docking, rather than a complete decoupling of Munc18c. Munc18c remains bound to Syntaxin 4 in the open state (requiring additional interaction with the N-end of Syntaxin 4) and this complex facilitates the docking of GLUT4 vesicles.	Docking/fusion										Unclear risk (Not n	Unclear risk (Not n	Low risk	Unclear risk (Not re	Low risk (Blindi	Low risk
A.473	10.1083/jcb.201007176	Jenna L. Jewell, 2011	USA	Identify the kinase responsible for the phosphorylation of Munc18c and test the hypothesis that tyrosine phosphorylation of Munc18c is functionally necessary for syntaxin-mediated proliferation 4 (specifically GLUT4).	Cells: Differentiated 3T3-L1 adipocytes (3T3-L1 adipocytes) and CHO cells express insulin receptor stability (CHO/IR).<br><br>Animal tissue: Mouse hind-limb skeletal muscle.<br><br>Recombinant proteins: Recombinant His-Munc18c, GST-Munc18c.	Stimulants: Insulin (100 nM or 10 U/kg), Pervanadate (pV, 0.1 mM).<br><br>Pharmacological inhibition: HNMPA-(AM)3 (inhibits IR kinase activity), Wortmannin (inhibits PI3K).<br><br>Genetic interference (Knockdown): siRNA/shRNA targets Munc18c (siMunc18c).<br><br>Gene expression (Mutants): Flag-Munc18c Wild-type (WT), Phosphodefective (Y219F, Y521F) and Phosphomimetic (Y219E, Y521E, Y219E/Y521E) mutants.	Immunoprecipitation (IP) & Immunoblotting (IB): Measures protein interactions (IR-Munc18c, Munc18c-Syntaxin 4) and tyrosine phosphorylation levels.<br><br>In vitro kinase assay: Measures the integration of 32P into Munc18c.<br><br>Myc-GLUT4-GFP exocytosis assay: Measurement of translocation and membrane fusion of GLUT4 (detection of extracellular Myc by fluorescence microscopy).<br><br>2-deoxyglucose uptake: Measurement of glucose uptake (auxiliary data).	Signaling/Phosphorylation: 0.5 minutes after insulin stimulation.<br><br>In vitro kinase: 1, 3, 5, 10, 20, 30 minutes.<br><br>GLUT4 Exocytosis: 30 minutes after insulin stimulation.	Excitatory state: Basal (0 min/Saline) versus Insulin (5 min/30 min).<br><br>Inhibition: Vehicle vs. HNMPA-(AM)3 or Wortmannin.<br><br>Munc18c Genotype:<br><br>siControl vs siMunc18c.<br><br>Rescue by Munc18c-WT compared to mutations (Y219F, Y521F, Y219E, Y521E).<br><br>Interactions: Munc18c alone versus Syntaxin-linked Munc18c 4 (in vitro kinase assay).	1. IR binds to and tyrosine phosphorylates Munc18c<br>2. Munc18c tyrosine phosphorylation requires IR kinase activity in a pathway independent of PI3K activation<br>3. Munc18c serves as a novel IR kinase substrate in vitro<br>4. Munc18c phosphorylation at Tyr219 and Tyr521 is required for insulin-stimulated GLUT4 vesicle docking/fusion	Munc18c	Munc18c when phosphorylated stimulated	Insulin Receptor (IR) acts directly as a tyrosine kinase for Munc18c (especially at Tyr521 in vitro, and also Tyr219/Tyr521 in vivo). This direct phosphorylation is a novel signaling step, independent of the traditional PI3K/Akt pathway, which results in Munc18c cleavage from Syntaxin 4, thereby allowing for the formation of the SNARE complex and GLUT4 proliferation.	Fusion										Low risk (H. Green	Unclear risk (Not n	Low risk	Unclear risk (Not re	Unclear risk (No	Low risk
A.474	10.1074/jbc.M007419200	Ahmir H. Khan, 2001	USA	Creating a transgenetic mouse strain expressing GLUT4-EGFP specific in rhabdomyopathy to examine intracellular transport and translocation of GLUT4, and to determine the functional role of Munc18c in this process in different membrane regions (sarcolemma and transverse tubules).	Animals: Transgenic mice express GLUT4-EGFP under the control of human rhabdomyocardial actin promoter (HSA). C57Bl/6 mouse background.<br><br>Tissues: Rhabdomyocardia (quadriceps, twin muscles - gastrocnemius), heart, adipose tissue.	Gene expression (Adenovirus): Adenovirus encoding Munc18c or Munc18b is injected into the quadriceps muscles of mice.<br><br>Stimulation:<br><br>- Insulin (peritoneal injection, 21 units/kg).<br><br>- Exercise (running on a treadmill).<br><br>- Glucose (peritoneal injection 2g/kg for tolerance test).	Immunoblotting (Western Blot): Determines protein expression in tissues and cell segments.<br><br>Glucose Tolerance Test (GTT): Measures glucose tolerance.<br><br>Subcellular Fractionation: Separation of cell membranes by sucrose gradient centrifugation.<br><br>Confocal Immunofluorescence Microscopy: Observation of GLUT4-EGFP translocation and protein localization on frozen muscle tissue slices.	GTT: 0, 30, 60, 90, 120 minutes after glucose injection.<br><br>Insulin stimulation: 30 minutes after injection.<br><br>Exercise: 60 minutes of running.<br><br>Adenovirus expression: 4 days after viral injection.	Tissue Expression Experiments:<br><br>- Wild-type mice (WT) vs Transgenic mice (TG).<br><br>- Comparison between tissues: Heart (H), Diaphragm (D), Quadriplex (Q), Twin Muscle (G), Adipose Tissue (F).<br>Glucose Tolerance Test:<br><br>- Wild-type mice (WT) vs Transgenic mice (TG).<br><br>GLUT4 Displacement Experiment:<br><br>- Basal/Untreated) vs. Stimulated (Insulin or Exercise).<br><br>- Membrane location: Sarcolemma (using β-dystroglycan marker) vs Transverse-tubules (using α1-VDCC marker).<br><br>Munc18 Intervention Experiment:<br><br>- Noninfected/Surrounding fibers vs Overexpressing fibers.<br><br>- Adenovirus Munc18b vs Adenovirus Munc18c.<br><br>- Basal vs Insulin.	1. The GLUT4–EGFP protein is localized in the same intracellular cavity as the endogenous GLUT4 protein and undergoes translocation to both the sarcolema membrane and the transverse tubules (T-tubules) when stimulated by insulin or motor. Consistent with previous studies on fat cells, the increase in vivo expression of the isoform Munc18c, which is potentially binding to syntaxin 4, but not the associated isoform Munc18b, selectively inhibited insulin-stimulated GLUT4–EGFP translocation.<br>2. Munc18c only inhibits GLUT4 translocation to the transverse tubular membrane without affecting the translocation to the sarcolema membrane. The ability of Munc18c to prevent GLUT4–EGFP translocation to the transverse tubular membrane, but not to the sarcolema membrane, is consistent with the significant reduction in syntaxin 4 levels in the transverse tubular membrane.<br>3. Munc18c has a specific role in regulating GLUT4 translocation in each cavity, specifically to the transverse duct system in skeletal muscle.	Munc18c	Inhibition	Munc18c specifically regulates the translocation of GLUT4 to the transverse tube system (T-tubules) without affecting the sarcolema membrane. The proposed mechanism is due to differences in Syntaxin 4 amounts: Syntaxin 4 is abundant in sarcolema membranes (difficult to saturate by exogenous Munc18c) but low in T-tubules (susceptible to saturation/inhibition by Munc18c).	Fusion	Unclear risk (Not n	Low risk	Unclear risk (Not n	Unclear risk (Not n	Unclear risk (Not n	Unclear risk (Not n	Unclear risk (Not n	Low risk		Low risk	Unclear risk (Not n	Low risk	Unclear risk (Not re	Unclear risk (No	Low risk
A.475	10.1152/ajpendo.00573.2005	Nadeeja Wijesekara, 2006	Canada	Investigate the impact of membrane depolarization on the GLUT4 transport cycle and identify the associated regulatory signals (separate from muscle contraction).	L6 rhabdomyocytes (L6 myotubes) stably express myc-tagged GLUT4 (L6-GLUT4myc).	Depolarization: Use a medium with high concentrations of K+ (40, 80, 120 mM Potassium gluconate) or Gramicidin (1μM).<br>Control stimulation: Insulin (100 nM), DNP (0.5 mM).<br>Pharmacological Inhibitions: Indinavir (GLUT4 inhibition), Wortmannin (PI3K inhibition), Cytochalasin D (actin breakdown), Dantrolene (inhibition of Ca2+ release from SR), EGTA/BAPTA-AM (Ca2+ locking), KN62 (CaMK inhibition), BIM/Calphostin C (PKC inhibition).<br>Genetic intervention: siRNAs targeting AMPK α1 and AMPK α2	Glucose Uptake: Measures the absorption of 2-Deoxy-D-[3H]glucose and 3-O-methylglucose.<br><br>Surface GLUT4 measurement: A colorimetric absorption assay using anti-myc antibodies on non-permeabilized cells.<br><br>Intracellular Ca 2+ : Fluorescence measurement with Fura 2-AM.<br><br>Immunoblotting: Determines the degree of phosphorylation of Akt, AMPK, CaMKII.<br><br>GLUT4 Kinetics: Measures the rate of externalization/exocytosis and internalization/endocytosis of GLUT4myc.	Glucose Uptake: 5 minutes (after 20 minutes of stimulation).<br><br>Surface GLUT4: After 20 minutes of stimulation.<br><br>Signaling (Phosphorylation): After 10 minutes of stimulation.<br><br>Kinetics: Monitoring over time (up to 240 minutes for exocytosis, 20 minutes for endocytosis).	Basal group: Non-stimulating.<br><br>Stimulation group: K+ depolarization, Insulin, DNP.<br><br>Inhibitor group: Stimulant + Inhibitor (Wortmannin, Dantrolene, KN62, BIM, etc.).<br><br>SiRNA group: Non-related siRNA (NR) vs. AMPKα1/2 siRNA.	1. K⁺-induced membrane depolarization increases GLUT4myc on the cell surface; This effect, combined with the effect of insulin, is not prevented by inhibition of phosphatidylinositol 3-kinase (PI3K) or actin polymerization, and is not associated with Akt activation.<br>2. Instead, depolarization increases Ca^{2+} in the cytoplasm, and the increase in superficial GLUT4myc is suppressed by dantrolene (an inhibitor of Ca^{2+} release from the muscular reticulum) as well as by extracellular Ca^{2+} chelate complexation. Ca^{2+}–calmodulin-dependent protein kinase II (CaMKII) was not phosphorylated after 10 min of K⁺ depolarization, and the CaMK inhibitor KN62 also did not prevent the increase of GLUT4myc on the surface.<br>3. Although the kinase activated by 5'-AMP (AMPK) is phosphorylated when depolarization occurs, the reduction of AMPKα by siRNA does not alter the increase in GLUT4myc on the surface. In contrast, this response is eliminated by the PKC inhibitors bisindolylmaleimide I and calphostin C.<br>4. Unlike insulin, K⁺ depolarization only causes a slight increase in the proliferation of GLUT4myc but drastically reduces its cell entry.<br>5. K⁺ depolarization reduces the internalization of GLUT4 through signals and mechanisms different from those induced by insulin. This pathway (or pathways) is largely independent of PI3K, Akt, AMPK, and CaMKII, but may be related to PKC.	K+, Ca2+	Stimulation	The mechanism of increasing surface GLUT4 due to membrane depolarization is completely different from that of insulin. While insulin primarily promotes exocytosis via PI3K/Akt/Actin, membrane depolarization increases GLUT4 primarily by decreasing the rate of endocytosis (slowing down cell entry). This pathway depends on an increase in Ca2+ intracellular and PKC activity, but independent of AMPK and CaMKII.	Signaling										Unclear risk (Not n	Unclear risk (Not n	Low risk	Unclear risk (Not re	Unclear risk (No	Low risk
A.476	10.1152/ajpcell.00277.2008	Shuhei Ishikura, 2008	Canada	Explore the downstream events of AS160 and TBC1D1 (specifically Rab proteins and myosin Vb) regulating GLUT4 translocation in muscle cells.	L6 muscle cells express myc-tagged GLUT4 (L6-GLUT4myc), including both myoblast and myotube stages.	siRNA Knockdown: Targeting AS160, TBC1D1, Rab8A, Rab8B, Rab10, and Rab14.<br><br>Overexpression: Plasmids encoding GFP-Rab8A (WT, CA, DN), GFP-Rab11A, and Myosin Vb fragments (FLAG-MVb-GT, FLAG-MVb-Tail).<br><br>Stimulation: 100 nM insulin for 20 minutes.	Colorimetric absorbance assay: Quantifying surface GLUT4myc levels.<br><br>Immunoblotting (Western blot): Determining protein expression levels and phosphorylation states.<br><br>Real-time quantitative PCR (RT-qPCR): Measuring TBC1D1 mRNA levels.<br><br>Confocal fluorescence microscopy: Observing the intracellular distribution of Rab proteins and GLUT4.	Post-siRNA transfection: 48 hours (myoblasts) or 72 hours (myotubes).<br>Post-insulin stimulation: 20 minutes.	siRNA Comparison: Control group (siNR - non-related siRNA) versus knockdown groups (siAS160, siTBC1D1, siRab8A, siRab8B, siRab10, siRab14, and double-knockdown combinations).<br><br>Stimulation Comparison: Basal (unstimulated) state versus insulin-stimulated state.<br><br>Vector Comparison: Untransfected or control vector-transfected cells versus cells expressing Myosin Vb fragments (Tail/GT).	Silencing AS160 or TBC1D1 increased cell-surface GLUT4 under basal conditions, but did not prevent insulin-stimulated GLUT4 translocation.<br><br>Knockdown of Rab8A and Rab14, but not Rab8B or Rab10, inhibited insulin-stimulated GLUT4 translocation. Furthermore, silencing Rab8A or Rab14, but not Rab8B or Rab10, restored basal intracellular retention of GLUT4, which was impaired upon AS160 or TBC1D1 knockdown.<br><br>Overexpression of a fragment of myosin Vb, a protein recently identified to interact with Rab8A, inhibited insulin-stimulated GLUT4 translocation and altered the subcellular distribution of GTP-bound Rab8A.<br><br>AS160, Rab8A, and myosin Vb are required for insulin-stimulated GLUT4 translocation in muscle cells, potentially functioning as components of a linear signaling cascade.	AS160 (TBC1D4), TBC1D1.	Stimulation	These results support a model whereby AS160, Rab8A, and myosin Vb are required for insulin- induced GLUT4 translocation in muscle cells, potentially as part of a linear signaling cascade.	Signaling										Unclear risk (Not n	Unclear risk (Not n	Low risk	Unclear risk (Not re	Unclear risk (No	Low risk
A.478	10.1152/ajpendo.00030.2013	Ognian C. Ikonomov, 2013	USA	Investigate the role of PIKfyve in muscle and systemic glucose metabolism by developing a new mouse model with the specific rhabdomyoscopic disrupted Pikfyve gene (MPIfKO).	Animal: Mouse (C57BL/6J background). Specifically, MPIfKO (Muscle-specific PIKfyve Knockout) mice and control mice.<br>Tissue: Isolated rhabdomyoplasty (soleus muscle, long toe extensor muscle - EDL, lower leg twin muscle - gastrocnemius) and adipose tissue.	Genetic intervention (Knockout): Using the Cre-loxP system with the promoter MCK (Muscle Creatine Kinase) to delete the specific Pikfyve gene in rhabdomyopathy.<br><br>Stimulation: Insulin (peritoneal injection or ex vivo incubation), Glucose (peritoneal injection).<br><br>Diet: The standard diet for rodents.	Glucose/Insulin Tolerance Tests (GTT/ITT): Measures glucose tolerance and systemic insulin sensitivity.<br><br>2-deoxyglucose (2DG) uptake: Measurement of glucose uptake in vivo and ex vivo (using radioisotopes).<br><br>Subcellular fractionation: Separation of cell membranes and intracellular membranes to evaluate GLUT4 translocation.<br><br>Western blotting: Determination of protein levels and phosphorylation (Akt, GLUT4, PIKfyve, etc.).<br><br>EchoMRI: Measures body composition (fat/lean mass).<br><br>In vitro lipid kinase activity assay: Measure the lipid kinase activity of PIKfyve.	Mouse age: 8-10 weeks, 4-5 months, 6 months, 9-10 months (to monitor phenotypic progress).<br><br>After insulin stimulation: 15 minutes (for glucose uptake and fractionation), 5 and 15 minutes (for signaling).	Control mice (PIKfyve fl/fl) vs. mutant mice (MPIfKO).<br><br>Basal (Basal/Saline) vs. Insulin Stimulation.<br><br>Oxidative muscle (soleus) vs. glycolytic muscle (EDL).	1. MPIfKO mice are glucose intolerant, insulin resistant, and have hyperinsulinemia.<br>2. Reduces insulin-stimulated glucose uptake and reduces GLUT4 accumulation on the cell surface in the muscles of MPIfKO mice.<br>3. Insulin-dependent Akt phosphorylation is impaired in MPIfKO rat muscles.<br>4. Muscle fiber type in MPIfKO mice did not change despite metabolic disorders.	Pikfyve	Stimulation	IKfyve in muscle is essential for GLUT4 translocation and glucose uptake regulated by insulin in vivo. The proposed molecular mechanism is that PIKfyve (or its lipid product such as PtdIns5P) is necessary to maintain prolonged Akt phosphorylation after insulin stimulation. PIKfyve deficiency leads to premature deterioration of the Akt signal, which in turn damages the GLUT4 translocation.	Signaling	Unclear risk (Not n	Low risk	Unclear risk (Not n	Unclear risk (Not n	Unclear risk (Not n	Unclear risk (Not n	Unclear risk (Not n	Low risk		Low risk	Unclear risk (Not n	Low risk	Unclear risk (Not re	Unclear risk (No	Low risk

A.480	10.1091/mbc.E12-04-0263	Shlomit Boguslavsky, 2012	Canada	Identify the molecular mechanisms involved in tethering GLUT4 vesicles and examine the role of the motor protein Myo1c in insulin-stimulated GLUT4 transport in muscle cells.	Cells: L6 myoblasts express myc-tagged GLUT4 (L6-GLUT4myc) or fluorescent-tagged GLUT4 (GLUT4-RFP/GFP).<br>Tissue: Muscle fibers separated from the short toe flexor muscles (FDB fibers) of male rats (12 weeks of age).	Stimulus: Insulin (100 nM).<br>Knockdown (siRNA): Targets Myo1c (siMyo1c), MyoIIA, Clathrin heavy chain, CaMKII.<br>Gene expression (Overexpression): WT-Myo1c-YFP, T-Myo1c-YFP (actin binding inability mutation), Dynamin-2 K44A, CaMKII variants.<br>Chemical: Latrunculin B (destroys actin).	TIRF microscopy: Total reflection microscopy to observe the kinetics and anchoring of GLUT4 vesicles close to the cell membrane.<br>Cell-surface GLUT4 detection: Measures the amount of GLUT4 on a surface (immunofluorescence or colorimetry).<br>Immunoprecipitation (IP): Immunoprecipitation for protein interaction testing (GLUT4-Myo1c).<br>Actin free barbed-end assay: Measures actin coincidence.<br>Glucose uptake: The absorption of 2-deoxy-[3H]glucose.	Insulin stimulation: 10 minutes (for microscopy/TIRF) or 20 minutes (for glucose uptake/translocation).<br>After transfection: 24-48 hours.	Status: Basal (basic) vs. Insulin.<br>SiRNA: siNR (control) vs. siMyo1c.<br>Myo1c variant: WT-Myo1c (wild) vs. T-Myo1c (tail shortening mutation - no actin).	1. Myo1c knockdown or overexpression of an actin binding-deficient Myo1c mutant abolished insulin-induced vesicle immobilization, increased GLUT4 vesicle velocity in the TIRF zone, and prevented their externalization.<br>2. Myo1c overexpression immobilized GLUT4 vesicles in the TIRF zone and promoted insulin-induced GLUT4 exposure to the extracellular milieu. Myo1c also contributed to insulin-dependent actin filament remodeling.<br>3. Interaction of vesicular Myo1c with cortical actin filaments is required for insulin-mediated tethering of GLUT4 vesicles and for efficient GLUT4 surface delivery in muscle cells.	Myo1c	Stimulation	Myo1c is an essential component for the tethering step of GLUT4 bags. Myo1c binds to the GLUT4 vesicle interacting with cortical actin filaments under the plasma membrane. This interaction reduces the immobilization of the bag, keeping the bag in the TIRF region to facilitate the next docking and fusion steps. This process is independent of CaMKII.	Tethering										Unclear risk (Not r	Unclear risk (Not r	Low risk	Unclear risk (Not rep	Unclear risk (No	Low risk
A.481	10.1128/MCB.02298-06	Takeshi Yoshizaki, 2007	USA	Investigate whether an actin-based motor protein is a target of Akt and its role in regulating insulin-stimulated GLUT4 transport.	Differentiated 3T3-L1 adipocytes.	Stimulant: Insulin (0.5 nM or 17 nM).<br>Pharmacological inhibition: Wortmannin (PI3K inhibition).<br>Genetic interference (Knockdown): siRNAs target Myosin 5a, Myosin 5b, Myosin 5c, Akt1, Akt2, PKClambda.<br>Gene expression (Overexpression): Adenovirus expresses dominant-negative Akt (DN-Akt); Plasmids express the tail of Myosin 5a (MGT-WT, MGT-Ser1650Ala, MGT-Ser1812Ala).	Immunoprecipitation & Immunoblotting: Determination of phosphorylation and protein interactions (using phospho-Akt substrate - PAS antibodies).<br>In vitro kinase assay: Measure the direct phosphorylation of Akt2 to Myosin 5a.<br>2-Deoxyglucose uptake: Measures glucose uptake.<br>GLUT4 translocation assay.<br>Ring assay: Single-cell fluorescence immunity for endogenous GLUT4 belt detection.<br>Ratiometric assay: Use HA-GLUT4-eGFP to measure the surface GLUT4/total ratio.<br>Cosedimentation assay: Measures the binding capacity of Myosin 5a to F-actin.	Signal (Phosphorylation): 5, 10, 30 minutes after insulin stimulation.<br>Glucose Transport/Absorption: 20 minutes after insulin stimulation.<br>Post-electroporation (siRNA/Plasmid): 48 hours.	Status: Basal (non-insulin) vs Insulin.<br>SiRNA genotype: Control siRNA (scrambled) compared to targeted siRNAs (Myo5a, Akt1, Akt2....).<br>Protein variant: MGT-WT (Wild type) vs. MGT-Ser1650Ala (non-phosphorylated mutation).<br>Akt Activity: Adenovirus GFP (control) versus Adenovirus DN-Akt.	1. Inhibition of myosin 5a by small interfering RNA (siRNA) as well as the expression of the dominant-negative form of myosin 5a impair glucose transport and insulin-stimulated GLUT4 translocation.<br>2. Knockdown of Akt2 or negative dominant Akt expression (DN-Akt) eliminated insulin-stimulated myosin 5a phosphorylation, inhibited the binding of myosin 5a to actin, and blocked insulin-stimulated glucose transport.<br>3. Myosin 5a is a newly identified direct substrate of Akt2 and, when phosphorylated under the action of insulin, myosin 5a promotes anterograde migration of GLUT4 vesicles along the actin system to the cell surface.	Myosin 5a	Stimulation	Myosin 5a is a novel direct substrate of Akt2. When insulin activates Akt2, it phosphorylates Myosin 5a at Ser1650. This event activates Myosin 5a's motility and strengthens its binding to actin, allowing Myosin 5a to transport GLUT4 vesicles (delivered from the microtubule/KIF3/Rab4 system) along the actin filament to the plasma membrane for cell exocytosis.	Translocation										Unclear risk (Not r	Unclear risk (Not r	Low risk	Unclear risk (Not rep	Low risk (Blind c	Low risk
A.482	10.1016/j.bbrc.2009.12.004	Le Thi Kim Chung, 2010	Japan	To investigate the specific role of myosin IIA in distal events of Glut4 vesicle transport (GSVs), namely docking and formation of SNARE complexes at the plasma membrane.	Differentiated 3T3-L1 adipocytes.	Knockdown: Using siRNAs that target myosin IIA (siMyosin IIA).<br>Stimulus: Insulin (100 nM).<br>Gene expression: Plasmid transfer pcDNA3-myc-Glut4 (myc-tagging Glut4 expression).	Western blot analysis: Determination of the level of protein expression and phosphorylation (Akt).<br>Glucose uptake assay: Measure the absorption of 2-deoxy-D-[3H]glucose.<br>Immunofluorescence: Observe the translocation of Glut4 to the cell membrane margin.<br>Colorimetric assays: Measures the amount of myc-Glut4 expressed on the cell surface.<br>Immunoprecipitation (IP): Check the interaction between VAMP2 and Syntaxin 4.	Signaling/SNARE complex: 10 minutes or 15 minutes after insulin stimulation.<br>Glucose Absorption: 15 minutes of stimulation + 4 minutes of measurement.<br>Superficial Glut4 expression: 30 minutes after insulin stimulation.<br>After electroporation: 48 hours.	siRNA group: siControl (control) versus siMyosin IIA (knockdown).<br>Status: Basal (non-insulin) vs Insulin.	1. Knockdown of myosin IIA reduces both glucose uptake and myc-tagged GLUT4 levels expressed on the cell surface in insulin-stimulated cells, but does not affect insulin signaling.<br>2. Knockdown myosin IIA does not reduce insulin-dependent GLUT4 transport to the cell membrane.<br>3. In myosin IIA-knocked cells, insulin-stimulated binding between the SNARE protein of GSVs which is vesicle-associated membrane protein 2 (VAMP2) and the SNARE protein of the cell membrane syntaxin 4 is inhibited.<br>4. Myosin IIA plays a role in insulin that stimulates the docking of GSVs onto the cell membrane in 3T3-L1 adipocytes through the formation of SNARE complexes.	myosin II	Stimulation	Myosin IIA is not involved in the translocation of the Glut4 vesicle from the intracellular to the periphery, but plays an essential role in the docking of the Glut4 vesicle into the plasma membrane. The specific mechanism, Myosin IIA, supports the interaction between v-SNARE (VAMP2) on the vesicle and t-SNARE (Syntaxin 4) on the membrane to form the SNARE complex, thereby allowing Glut4 to insert into the membrane.	Docking										Unclear risk (Not r	Unclear risk (Not r	Low risk	Unclear risk (Not rep	Unclear risk (No	Low risk
A.484	10.1091/mbc.E13-08-0493	Yi Sun, 2014	Canada	Investigate whether the motor protein Myosin Va (MyoVa) is an effector of Rab8A during the regulation of the transport of GLUT4-containing vesicles to the muscle cell membrane under insulin stimulation.	Cells: L6 muscle cells express GLUT4 tagged myc (L6-GLUT4myc), including myoblasts and myotubes.<br>Other Cells: CHO cells express insulin receptors (CHO-IR) for lysates.	1. Stimulation: Insulin (10 nM or 100 nM).<br>2. Gene expression (Overexpression):<br>GFP-MyoVa (Full length).<br>GFP-MyoVa-CT (Cargo-binding COOH tail, acting as a dominant negative).<br>MyoVa-CT mutations (M1829K, D1834K, N1838K).<br>Rab8A (mCherry-Rab8A, the active form constitutes Q67L, the inactivated form T22N).<br>3. Knockdown: shRNA targets MyoVa.<br>4. Pharmacological inhibition: Wortmannin (PI3K inhibitor).	GST-pulldown assay: Examines the direct interaction between the MyoVa tail segment and the Rabs.<br>Immunoprecipitation (Co-IP): Examines intracellular protein interactions.<br>TIRF microscopy (Total Internal Reflection Fluorescence): Observation of docking/fusion events of GLUT4 vesicles in the submembrane region.<br>Confocal microscopy: Observation of colocalization.<br>Colorimetric surface GLUT4 assay: Measures the amount of GLUT4 on the cell surface.	Insulin stimulation: 10 minutes or 20 minutes.<br>After transfection: 24-48 hours.	1. State: Basal (basic) vs. Insulin.<br>2. Gene expression:<br>GFP (Control) vs. GFP-MyoVa-CT.<br>MyoVa-CT WT vs. MyoVa-CT mutants (M1829K, etc.).<br>Control shRNA vs MyoVa shRNA.<br>3. Interactive: Rab8A vs. Rab10 and Rab13.	The insulin signal acts on the molecular "switch" of Rab8A which is linked to the MyoVa motor protein to mobilize GLUT4-containing vesicles to move towards the plasma membrane of muscle cells.	Myosin Va (MyoVa), Rab8A	Stimulation	Myosin Va is a direct effector of Rab8A (activated by insulin) in muscle cells. Rab8A interacts with Myosin Va to mobilize GLUT4 vesicles from the perinuclear or intracellular reservoirs to the periphery/cell membrane. This interaction is necessary for the GLUT4 vesicle to reach and dock at the plasma membrane.	Translocation										Unclear risk (Not reported)	Unclear risk (Not reported)	Low risk	Unclear risk (Not reported)	Unclear risk (No	Low risk
A.485	10.1016/j.abb.2004.01.011	Seung Y. Park, 2004	Korea	The study aimed to determine the identity and function of an unknown 85-kDa protein found in GLUT4-vesicles in adipose tissue cells, thereby determining its role in regulating GLUT4 traffic.	Adipocytes were isolated from male rats of the Sprague–Dawley strain.	Fat cell treatment with the specific NAALADase inhibitor 2-[(hydroxymethylphosphinyl)pentanedioic acid (2-PMPA) at a concentration of 50 µM for 1 hour, followed by insulin stimulation (30 nM).	Subcellular fractionation, Immunoadsorption, SDS-PAGE and Western blot electrophoresis; Mass spectrometry analysis (MALDI-TOF MS) for protein identification; RT-PCR and cloning; 3-O-methyl-D-glucose equilibrium exchange flux.	Measures after incubation with 2-PMPA inhibitors for 1 hour and insulin stimulation for 20 minutes.<br>Glucose Transport Measurement: Time course tracking from 0 to 100 seconds.	Comparison between the control group (no 2-PMPA treatment) and the experimental group (with 2-PMPA treatment) in both conditions: basal (basal - no insulin) and with insulin stimulation.	NAALADase may function as a regulator required for the insulin-stimulated GLUT4 vesicle movement and/or its exocytosis, thus may regulate insulin-induced GLUT4 recruitment in rat adipocytes.	NAALADase	Stimulation	The study found that NAALADase (which is known as a neuronal enzyme) is present on intracellular GLUT4 vesicles (especially the sorting endosome) in fat cells. It acts as an essential regulator for the mobilization of GLUT4 to the cell membrane under the action of insulin. This mechanism works independently of the insulin signaling pathway (without affecting IRS-1 and Akt phosphorylation).	Translocation										Unclear risk (Not reported)	Unclear risk (Not reported)	Low risk	Unclear risk (Not reported)	Unclear risk (No	Low risk
A.487	10.1111/jdi.13623	Kazuhisa Watanabe, 2021	Japan	The study aims to elucidate the physiological mechanisms that regulate N4bp2l1 gene expression and the role of N4BP2L1 protein on the physiological function of fat cells (adipocytes), specifically in GLUT4 transport and glucose uptake.	• Animal: Male rat C57BL/6 (8 weeks old).<br>• Cells: 3T3-L1 fat cells (differentiated from pre-fat cells) and HEK293T cells.	Genes: Overexpression of the N4bp2l1 gene by adenovirus or Knockdown with shRNA.<br>Chemical: Insulin stimulation (1 µM) or insulin signaling inhibition with PI3K inhibitors (LY294002, 50 µM).<br>Nutrition (on mice): Fasting and refeeding; Chow diet versus high-fat diet	Quantification of gene expression: Real-time PCR (qPCR).<br>Protein quantification: Western blot.<br>Protein interactions: Co-immunoprecipitation.<br>DNA-Protein interactions: Chromatin immunoprecipitation (ChIP) assay, Electrophoretic mobility shift assay (EMSA).<br>Promoter activity: Luciferase reporter assay.<br>Cell Function: GLUT4-HiBiT translocation assay, Glucose Uptake-Glo assay	In rats: Fasted for 24 hours, then measured at re-feeding times: 4, 6, 8, and 12 hours.<br>• On cells (N4BP2L1 expression): 0, 1, 2, 4, 8 hours after insulin administration.<br>• GLUT4 (Translocation): Measured at 0, 0.25, 0.5, 1, 2, 4, 8 hours after stimulation.<br>• Glucose Absorption: Measured after 1 hour of insulin stimulation.	In vivo tests: Fasting rats compared to refed rats.<br>• In cellular assays: Control group (GFP or shLacZ) versus intervention group (N4bp2l1 overexpression or shRNA knockdown).<br>• Processing Conditions: With insulin vs No insulin; With LY294002 inhibitors vs None.	The results showed that N4bp2l1 is a novel target gene for FoxO1 and that the expression of this gene is regulated by insulin through the regulatory mechanism of FoxO1. N4BP2L1 interacts with the dynactin complex, which binds to the dynein microtubule motor protein, suggesting that N4BP2L1 is involved in GLUT4 transport and glucose uptake in 3T3-L1 fat cells.	N4bp2l1	Stimulation	1. Protein interactions: N4BP2L1 interact with dynactins (specifically DCTN3–6 subunits and possibly a new component of the pointed-end complex), which in turn binds to the dynein molecular motor to participate in GLUT4 transport.<br>2. Gene Regulation: N4bp2l1 is a new target gene of FoxO1. Insulin stimulates FoxO1 to leave the nucleus, leading to a decrease in the expression of the N4bp2l1 gene (negative feedback mechanism).	Translocation	Unclear risk (Not r	Low risk	Unclear risk (Not r	Unclear risk (Not r	Unclear risk (Not r	Unclear risk (Not r	Unclear risk (Not r	Low risk	Low risk	Unclear risk (Not reported)	Unclear risk Not reported)	Low risk	Unclear risk (Not reported)	Unclear risk (No	Low risk
A.490	10.1074/jbc.M203669200	Silvia Mora, 2002	USA (USA) and Canada	To determine the function of NCS-1 in regulating insulin-stimulated GLUT4 translocation, based on the hypothesis that NCS-1 acts as a regulatory subunit of PI 4-kinase and that GLUT4-containing vesicles are enriched with PI 4-kinase activity.	Fully differentiated 3T3-L1 adipocytes	Chemicals: Stimulation with Insulin (100 nM), Endothelin-1 (100 nM), or Ionomycin (1 µM).<br>Gene Expression: Co-transfection of NCS-1, GLUT4-EGFP, GLUT1-EGFP, MBPR-EGFP, or PI 4-kinase (Wild-type and Mutant forms).	Translocation Measurement: Confocal fluorescence microscopy (quantification of cells with membrane fluorescence rims).<br>Calcium Measurement: FURA-2 indicator using a digital image analysis system.<br>Protein/Signaling Analysis: Immunoprecipitation and Western Blot (measuring Akt, PI 4-kinase, NCS-1, etc.).	Cell Differentiation: Monitored at days 0, 2, 4, 6, 10, and 12.<br>Post-transfection: 24-hour recovery period prior to experimentation.<br>Insulin Stimulation: Duration of 30 minutes.<br>Calcium Measurement: Recorded every 10 seconds over a period of 15–20 minutes.	Gene Expression: NCS-1 expressing group (Intervention) vs. Empty vector transfected group (Control).<br>Stimulation Conditions: Basal state (-Insulin) vs. Stimulated states: Insulin, Endothelin-1, or Ionomycin (to assess the role of Calcium).<br>Mechanism (Rescue): Co-transfection of NCS-1 with Wild-type PI 4-kinase vs. Co-transfection of NCS-1 with Kinase-deficient Mutant.	PI 4-kinase functions to negatively regulate GLUT4 translocation through its interaction with NCS-1.	NCS-1, PI 4-kinase	Inhibition	1. NCS-1 functionally interacts with Phosphatidylinositol 4-kinase (PI 4-kinase).<br>2. The enzymatic activity of PI 4-kinase acts as a negative regulatory signal. NCS-1 activates PI 4-kinase to inhibit GLUT4 translocation.<br>3. This mechanism is independent of intracellular Calcium alterations or known insulin signaling pathways such as Akt phosphorylation or Cbl mobilization.	Signaling										Low risk (ATCC)	Unclear risk (Not reported)	Low risk	Unclear risk (Not reported)	Unclear risk (No	Low risk
A.493	10.1371/journal.pone.0055634	Andrew Lee, 2013	Canada	To determine the role of nexilin (a cytoskeletal protein) as a novel component of the insulin signaling network, specifically examining its impact on the assembly of the IRS1/PI3K complex and Akt activation leading to glucose uptake in skeletal muscle cells.	L6 rat skeletal muscle cells (myotubes) and 3T3-L1 mouse adipocytes.	Chemicals: Stimulation with Insulin (1 nM, 10 nM, 100 nM); Treatment with actin-disrupting agent (Latrunculin B), actin-stabilizing agent (Jasplakinolide), or PI3K inhibitor (LY294002).<br>Genetics: Nexilin knockdown via siRNA or Overexpression via Adenovirus with Flag-tag.	Immunoprecipitation (IP).<br>Western Blot (Immunoblotting).<br>Confocal immunofluorescence microscopy.<br>Glucose uptake assay using radioactive isotope [³H] 2-Deoxy-D-Glucose..	Insulin Stimulation: Time points at 0, 5, 10, 20, 30, and 60 minutes.<br>Glucose Uptake: Insulin incubation for 20–30 minutes, followed by a 5-minute uptake measurement.<br>Drug Treatment: Latrunculin B (20 minutes), Jasplakinolide (30 minutes).	State Comparison: Basal (Starve) vs. Insulin-stimulated group.<br>Gene Intervention: Scramble siRNA control (scr) vs. Nexilin siRNA (si-nex); Adenovirus-GFP control (Ad-GFP) vs. Adenovirus-Nexilin (Ad-Nex).<br>Specificity: Immunoprecipitation with anti-IRS1 antibody vs. anti-IRS2 antibody.<br>Drug Treatment: Control group vs. Latrunculin B or Jasplakinolide treated groups.	Exposure of L6 myotubes to Latrunculin B disrupts the spatial patterning of nexilin and its transient association with IRS1. Functional silencing of nexilin has no effect on insulin-stimulated IRS1 tyrosine phosphorylation, however it enhances recruitment of p85a to IRS1 resulting in increased PI- 3, 4, 5-P3 formation, coincident with enhanced AKT activation and glucose uptake. By contrast, overexpression of nexilin inhibits transmission of IRS1 signals to AKT. Based on these findings we propose that nexilin may tether IRS1 to actin-rich structures under basal conditions, confining IRS1 signaling to specific subcellular locations in the cell.	Nexilin	Inhibition	Nexilin specifically binds to IRS1 (but not IRS2) via its coiled-coil (CC) domain. Under basal conditions, nexilin acts as a tether, anchoring IRS1 to actin structures, thereby restricting IRS1 interaction with PI3K (p85). Upon insulin stimulation, actin remodeling occurs, releasing this complex and allowing IRS1 to bind PI3K and transmit signals. Disrupting actin structure (via Latrunculin B) or rigidifying it (via Jasplakinolide) prevents this insulin-induced release of the nexilin-IRS1 complex.	Signaling										Unclear risk (Not r	Unclear risk (Not r	Low risk	Unclear risk (Not rep	Unclear risk (No	Low risk
A.501	10.1128/mcb.19.7.4684	Dong Chen, 1999	USA (USA) and Switzerland	To investigate the crosstalk between insulin and osmotic shock signaling pathways and to identify the molecular mechanism by which osmotic shock inhibits insulin-stimulated downstream biological responses (glucose transport, glycogen, and lipid synthesis).	3T3-L1 adipocytes.	Stimulation: Insulin (100 nM).<br>Osmotic Shock: Sorbitol (600 mM).<br>Inhibition: PI3K inhibitor (Wortmannin, 100 nM).<br>Phosphatase Inhibition: Okadaic acid (1 µM) or Calyculin A (25 nM).	Transport/Metabolism: 2-[³H] deoxyglucose uptake measurement; Lipogenesis and Glycogen synthesis measurement using radioactive glucose.<br>Protein Translocation: Plasma membrane sheet assay and Western blot for GLUT4 quantification.<br>Cell Signaling: Immunoprecipitation (IP), Western blot (using phosphospecific antibodies for PKB, Insulin Receptor, IRS1), In vitro kinase assays (PKB kinase assay, p70S6 kinase assay, PI 3-kinase assay).	Sorbitol Treatment (Osmotic Shock): Pre-incubation for 15, 30, or 45 minutes.<br>Insulin Stimulation: Time points at 2, 5, 10, 15, 30, 35, 50, 60, and 90 minutes.<br>Glucose Uptake: Measured over 5 minutes.<br>Lipid/Glycogen Synthesis: Measured over 1 hour.	Stimulation Conditions: Control (Untreated) vs. Insulin only (I) vs. Sorbitol only (S) vs. Sorbitol pretreatment followed by Insulin (S+I).<br>Inhibitor Comparison: Groups with vs. without Wortmannin (positive control for glucose transport inhibition). Groups with vs. without phosphatase inhibitors (Okadaic acid, Calyculin A) to test PKB phosphorylation recovery.	Osmotic shock-induced insulin resistance of downstream biological responses results from an inhibition of insulin-stimulated PKB activation. Osmotic shock pretreatment was found to completely prevent any further insulin stimulation of glucose transport activity and GLUT4 protein translocation. In contrast, osmotic shock pretreatment markedly inhibited the insulin stimulation of protein kinase B (PKB) and p70S6 kinase activities.	Protein Kinase B (PKB/Akt)	Stimulation (of Phosphatase activity) / Inhibition (of Insulin Signaling)	Osmotic shock induces insulin resistance not by inhibiting upstream nodes (Insulin Receptor, IRS1, PI3K), but by activating a phosphatase activity (PP2A-like). This phosphatase dephosphorylates PKB (at both Ser473 and Thr308), maintaining PKB in an inactive state even in the presence of insulin. Phosphatase inhibitors (Okadaic acid, Calyculin A) prevent this dephosphorylation.	Signaling										Unclear risk (Not r	Unclear risk (Not r	Low risk	Unclear risk (Not rep	Unclear risk (No	Low risk
A.502	10.1074/jbc.272.43.27401	Dong Chen, 1997	USA (USA)	To identify common and distinct signaling pathways leading to increased glucose transport by comparing the effects of insulin and osmotic shock on GLUT4 translocation in 3T3-L1 adipocytes.	Differentiated 3T3-L1 adipocytes.	Chemical Stimulation: Insulin (100 nM), Osmotic shock via D-Sorbitol (600 mM).<br>Inhibitors: Wortmannin (PI3K inhibitor), Genistein (Tyrosine kinase inhibitor), BAPTA-AM/EGTA (Intracellular Calcium chelators).<br>Technical Intervention: Microinjection of anti-phosphotyrosine antibody (PY20); Vaccinia virus expression of the Syntaxin-4 cytoplasmic domain (Syn4ΔTM) for competitive inhibition.	Glucose Transport: 2-deoxyglucose uptake assay.<br>GLUT4 Translocation: Plasma membrane sheet assay combined with Immunoblotting and Immunofluorescence.<br>Cell Signaling: Immunoprecipitation (IP), Western blot using anti-phosphotyrosine (PY20) and phospho-specific antibodies (p38 MAPK), Akt kinase activity assay.	Stimulation: Insulin or Sorbitol incubation for 30 minutes (time-course experiments at 5, 10, 15 minutes).<br>Glucose Transport: Measured over 10 minutes.<br>Inhibitor Treatment: 15-minute pre-incubation (Wortmannin, Genistein, BAPTA/EGTA).<br>Viral Infection: 4 hours prior to stimulation.	Stimuli: Control vs. Insulin vs. Sorbitol (and metabolic controls: Glucose, Xylose).<br>PI3K Inhibition: Without Wortmannin (-Wort) vs. With Wortmannin (+Wort).<br>Tyrosine Kinase Inhibition: Without Genistein (-Gen) vs. With Genistein (+Gen); IgG Microinjection (Control) vs. PY20 Antibody Microinjection.<br>Fusion Mechanism: Empty Virus (Vector) vs. Mutant Syntaxin-4 Virus (Syn4ΔTM).<br>Calcium: Vehicle vs. Calcium chelators (BAPTA/EGTA).	Osmotic shock-stimulated GLUT4 translocation in adipocytes proceeds via a novel tyrosine kinase pathway independent of both phosphatidylinositol 3-kinase (PI3K) and Akt kinase.	Osmotic shock-associated tyrosine kinases and substrates: CADTK (Calcium-dependent tyrosine kinase/Pyk2), p130cas, and Cbl.	Stimulation	Osmotic shock activates GLUT4 translocation via a novel tyrosine kinase pathway independent of the insulin receptor, IRS-1, PI 3-kinase, Akt kinase, and intracellular calcium levels (distinct from the traditional insulin pathway). This pathway involves tyrosine phosphorylation of CADTK (Pyk2), p130cas, and Cbl. However, both pathways (insulin and osmotic shock) converge at the final docking/fusion step, which requires Syntaxin-4 (t-SNARE).	Signaling										Unclear risk (Not r	Unclear risk (Not r	Low risk	Unclear risk (Not rep	Unclear risk (No	Low risk
A.503	10.1074/jbc.271.29.16987	Hideki Katagiri, 1996	Japan	To elucidate the involvement of Phosphatidylinositol 3-kinase (PI 3-kinase) in insulin-stimulated glucose transport activity.	3T3-L1 adipocytes.	Genetic: Overexpression of the PI 3-kinase catalytic subunit p110α via adenovirus-mediated gene transduction.<br>Chemical: Stimulation with Insulin at various concentrations.	Immunoblotting: Quantification of p110α, GLUT1, and GLUT4 expression levels.<br>PI 3-kinase Activity Assay: IP with anti-phosphotyrosine or anti-p85α antibodies followed by kinase activity measurement.<br>Glucose Transport Assay: Measurement of 2-Deoxy-D-[3H]glucose uptake.<br>Subcellular Fractionation: Separation of Plasma Membrane (PM) and Low-Density Microsomes (LDM) to track transporter distribution.<br>Membrane Sheet Assay & Immunofluorescence: In situ visualization of GLUT4 on the plasma membrane.	Experiments performed 3 to 4 days post-adenovirus infection.<br>Assay Protocol: 3-hour serum starvation, 45-minute glucose starvation, followed by measurement after 15-minute incubation with or without insulin.	Genetic: Control Adenovirus (LacZ) vs. Adenovirus expressing p110α.<br>Stimulation: Basal state (-Insulin) vs. Insulin-stimulated state (+Insulin).	An increment in PI 3-kinase activity induced by overexpression of p110α of PI 3-kinase stimulates glucose transport activity with translocation of glucose transporters, i.e., mimics the effect of insulin.	Phosphatidylinositol 3-kinase catalytic subunit p110α (p110α PI 3-kinase).	Stimulation	The study demonstrates that a specific increase in the p110α catalytic subunit (leading to increased association with the p85 regulatory subunit and elevated basal PI 3-kinase activity) is sufficient to stimulate glucose transport and GLUT4 translocation without insulin stimulation. This confirms the central and sufficient role of PI 3-kinase activation in the glucose transport signaling pathway (contrasting with contemporary hypotheses suggesting a requirement for an additional parallel signaling pathway).	Signaling										Unclear risk (Not r	Unclear risk (Not r	Low risk	Unclear risk (Not rep	Unclear risk (No	Low risk
A.505	10.1128/MCB.21.5.1633-1646.2001	Tsutomu Wada, 2001	Japan	To investigate the role of SHIP2 5'-phosphatase activity in insulin-stimulated metabolic signaling (glucose transport and glycogen synthesis) by overexpressing wild-type SHIP2 and phosphatase-deficient mutant SHIP2.	3T3-L1 adipocytes.	Genetic: Adenovirus-mediated overexpression of: Wild-type SHIP2 (WT-SHIP2) or Phosphatase-deficient mutant SHIP2 (ΔIP-SHIP2, acting as a dominant-negative).<br>Chemical: Stimulation with Insulin (17 nM or varying concentrations depending on the experiment).	Phospholipid Analysis: HPLC to measure PI(3,4,5)P3 and PI(3,4)P2 levels (In vivo generation of 32P-labeled phosphoinositides).<br>Kinase/Enzyme Activity: Assays for PI 3-kinase, Akt, PKC lambda, PP1, and Glycogen synthase activity.<br>Biological Function: 2-Deoxyglucose uptake assay (2-DOG uptake) and Glycogen synthesis assay (14C-Glucose incorporation).<br>Protein Translocation: Immunostaining and confocal microscopy to measure GLUT4 translocation.	Post-infection: 24 to 48 hours.<br>Insulin Stimulation: 5 minutes (for kinase/phospholipid measurements), 15–20 minutes (for glucose uptake/GLUT4), 30–60 minutes (for glycogen synthesis).	Genetic Comparison: Control group (LacZ) vs. WT-SHIP2 expressing group vs. ΔIP-SHIP2 expressing group.<br>Stimulation Comparison: Basal state vs. Insulin-stimulated state.	SHIP2 negatively regulates metabolic signaling of insulin via the 5'-phosphatase activity and that PI (3,4,5)P3 rather than PI(3,4)P2 is important for in vivo regulation of insulin-induced activation of downstream molecules of PI 3-kinase leading to glucose uptake and glycogen synthesis.	SHIP2	Inhibition	SHIP2 negatively regulates insulin signaling by hydrolyzing PI (3,4,5)P3 into PI(3,4)P2 via its 5'-phosphatase activity. The study demonstrates that PI(3,4,5)P3 (rather than PI(3,4)P2) is the critical signaling molecule for the in vivo activation of downstream targets such as Akt and PKCλ. Therefore, SHIP2-mediated reduction of PI(3,4,5)P3 levels leads to the inhibition of insulin's metabolic effects.	Signaling										Unclear risk (Not r	Unclear risk (Not r	Low risk	Unclear risk (Not rep	Low risk (Blind c	Low risk

A.508	10.1074/jbc.M910391199	Tomoichiro Asano, 2000	Japan	To compare the roles of two PI 3-kinase catalytic subunit isoforms (p110α and p110β) in regulating insulin action, specifically focusing on phosphoinositide metabolism and glucose transport.	3T3-L1 adipocytes differentiated from fibroblasts.	Genetic: Adenovirus-mediated overexpression of p110α or p110β. Microinjection of specific neutralizing antibodies (anti-p110α or anti-p110β).<br>Chemical: Stimulation with Insulin (100 nM).	Protein Expression: Immunoblotting (Western blot) to monitor protein levels during differentiation.<br>Enzyme Activity: PI 3-Kinase Assay (measured in immunoprecipitates).<br>Glucose Transport: Glucose Transport Assay (2-Deoxy-D-[^{3}H]glucose uptake).<br>GLUT4 Translocation: Membrane Sheet Assay combined with Immunofluorescence to visualize GLUT4 on the plasma membrane following microinjection.	Differentiation Process: Monitored daily from Day 1 to Day 6.<br>Post-infection: 48 hours (Day 6 post-differentiation initiation).<br>Insulin Stimulation: 5 minutes (kinase activity), 15 minutes (glucose transport and GLUT4 translocation).<br>Glucose Uptake Measurement: Over 4 minutes.	Isoform Comparison: p110α expressing group vs. p110β expressing group.<br>Control Comparison: LacZ virus (Control) vs. p110 virus groups.<br>State Comparison: Basal state vs. Insulin-stimulated state.<br>Microinjection Comparison: Control IgG injection vs. anti-p110α antibody injection vs. anti-p110β antibody injection.	p110β plays a crucial role in cellular activities evoked acutely by insulin.	PI 3-kinase catalytic subunits p110β and p110α.	Stimulation	p110β is identified as the critical isoform for acute insulin responses such as GLUT4 translocation. Conversely, p110α exhibits constant expression levels and high basal activity but is less responsive to insulin stimulation. p110α appears to serve a "housekeeping" role (maintaining basal activity, anti-apoptosis) rather than mediating acute insulin signaling.	Signaling										Unclear risk (Not r	Unclear risk (Not r	Low risk	Unclear risk (Not re	Unclear risk (No	Low risk
A.509	10.1091/mbc.e05-01-0072	Toshio Hosaka, 2005	USA (USA)	To identify cytoplasmic proteins interacting with the N-terminal domain of IRAP (a major cargo protein of GLUT4 vesicles) and determine their role in GLUT4 storage vesicle (GSV) trafficking.	3T3-L1 adipocytes and COS7 cells.	Genetic: Overexpression of fluorescence-tagged p115 structural fragments (N-terminal p115-EGFP, C-terminal p115-EGFP) via electroporation or transfection.<br>Chemical: Stimulation with Insulin (100 nM or 1.7 μM).<br>Biochemical: Use of IRAP-CBD (Chitin Binding Domain) affinity columns to pull down interacting proteins.	Protein Interaction: Immunoprecipitation.<br>Protein Identification: Affinity chromatography and Mass spectrometry.<br>Protein Interaction: Co-immunoprecipitation and Pulldown assays.<br>Protein Distribution: Subcellular Fractionation and Western blotting.<br>Localization and Translocation: Immunofluorescence and Confocal microscopy.	Insulin Stimulation: 15 minutes.<br>Post-electroporation/transfection: 48 hours.	Genetic Structure Comparison: Empty vector/EGFP (Control) vs. N-terminal p115-EGFP vs. C-terminal p115-EGFP.<br>State Comparison: Basal state (-Insulin) vs. Insulin-stimulated state (+Insulin).<br>Interaction Comparison: IRAP-CBD column vs. CBD-only column (Control).	p115 has an important and specific role in insulin-stimulated Glut4 translocation, probably by way of tethering insulin-sensitive Glut4 vesicles at an as yet unknown intracellular site.	p115 N-terminal fragment overexpression.	Inhibition	p115 (known as a Golgi tethering protein) interacts directly with the cytoplasmic N-terminus of IRAP on GLUT4 storage vesicles (GSVs). In adipocytes, p115 acts as a tether, anchoring GSVs in the perinuclear region and coordinating their trafficking. Overexpression of the N-terminal fragment of p115 (containing the binding domain but lacking other functional regions) acts competitively against endogenous p115, causing GSVs to disperse from the perinuclear region and lose their ability to translocate upon insulin signaling. This indicates p115 plays an essential and specific role in the localization and insulin-sensitive trafficking of GSVs	Translocation										Unclear risk (Not r	Unclear risk (Not reported)	Low risk	Unclear risk (Not reported)	Unclear risk (No	Low risk
A.510	10.1172/jci.insight.141344	Vitor F. Martins, 2022	USA (USA)	To investigate the combined contribution of the two acetyltransferases p300 and CBP to insulin-mediated glucose uptake.	Animals: Skeletal muscle-specific p300/CBP knockout mice (PCKO mice) and related compound heterozygous lines.<br>Cells: L6 myoblasts/myotubes, 3T3-L1 adipocytes, and primary human skeletal muscle cells.	Genetic: p300 and CBP knockout induction using tamoxifen.<br>Chemical:<br>* Insulin stimulation (physiological concentration 0.36 nM or high concentration 100 nM/6 nM).<br>* Inhibition of p300/CBP acetyltransferase activity using C646 or A-485 inhibitors.	Metabolism: Oral glucose tolerance test (OGTT), 2-deoxyglucose uptake (2DOG uptake) ex vivo and in vitro.<br>Molecular Analysis: Western blot (Akt, GSK3b phosphorylation), Microarray analysis (gene expression), Proteomics analysis (protein abundance).<br>Cytology: Flow cytometry (lysine acetylation and GLUT4 fusion), Total Internal Reflection Fluorescence (TIRF) microscopy (single-molecule GLUT4 kinetics).	Mice: Days 1 (D1), 3 (D3), or 5 (D5) post-tamoxifen administration.<br>Inhibitor Treatment (C646/A-485): Pre-incubation for 1 hour (Pre), concurrent treatment, or post-insulin stimulation treatment.<br>Insulin Stimulation: 20–30 minutes (for glucose uptake), 10–30 minutes (for signaling/translocation).	Genetic Comparison: Wild-type (WT) vs. PCKO (Double Knockout) vs. Single-allele rescue mice (p300 KO/CBP HZ, CBP KO/p300 HZ).<br>Chemical Comparison: Control (DMSO) vs. Inhibitor-treated groups (C646 or A-485).<br>Stimulation Comparison: Basal state vs. Insulin-stimulated state.<br>Timing Comparison: C646 treatment Pre- vs. Concurrent vs. Post-insulin.	Acetylation and p300/CBP are essential for the direct regulation of insulin-stimulated glucose transport in skeletal muscle and adipocytes.	p300, CBP (Lysine acetyltransferases).	Stimulation	p300/CBP directly regulates insulin-stimulated glucose uptake (non-transcriptionally) by controlling the exocytic translocation of GLUT4. Specifically, p300/CBP acetyltransferase activity is required for the movement of GLUT4 into the TIRF zone (near the plasma membrane) and for GLUT4 membrane fusion. This mechanism occurs downstream of or independently from Akt phosphorylation signaling (as inhibition of p300/CBP does not prevent GSK3 beta phosphorylation despite reducing Akt phosphorylation).	Translocation	Unclear risk (Not r	Low risk	Unclear risk (Not r	Unclear risk (Not r	Unclear risk (Not r	Unclear risk (Not r	Unclear risk (Not r	Low risk	Low risk	Low risk	Unclear risk (Not reported)	Low risk	Unclear risk (Not reported)	Unclear risk (No	Low risk
A.517	10.1042/bj3130125	Jing Yang, 1996	UK and Japan	To determine the site of involvement of PI 3-kinase in the recycling pathway of glucose transporters (GLUT1 and GLUT4), specifically determining whether PI 3-kinase promotes membrane retention or stimulates intracellular exocytosis.	3T3-L1 adipocytes.	Chemical: Stimulation with Insulin (1 μM) to reach steady state, followed by treatment with Wortmannin (100 nM or 1 μM) - a potent and specific PI 3-kinase inhibitor.<br>Labeling: Use of ATB-BMPA (membrane-impermeable photoaffinity label) to quantify cell surface GLUTs.	Glucose Transport: 2-deoxy-D-glucose uptake assay.<br>Surface GLUT Quantification: Photoaffinity labeling with ATB-BMPA followed by Immunoprecipitation with anti-GLUT1 or anti-GLUT4 and radioactivity measurement.<br>Kinetic Analysis: Monitoring changes in surface GLUT levels over time post-inhibition to calculate endocytosis and exocytosis rate constants.<br>Affinity Measurement: Binding assay (using ATB-BMPA binding to GLUT1/GLUT4).	Wortmannin Treatment: Monitored at 0, 3, 6, 10, 15, 20, 30, 45, and 60 minutes post-addition.<br>Glucose Uptake: Measured over 3 minutes or 10 minutes (depending on experiment).	State Comparison: Basal cells vs. Insulin-stimulated cells.<br>Inhibition Comparison: Cells under continuous Insulin stimulation (Positive Control) vs. Insulin-stimulated cells treated with Wortmannin.<br>Isoform Comparison: Comparison of effects on GLUT1 vs. GLUT4.	PI 3-kinase acts at an intracellular membrane site to promote the exocytosis of GLUT4 and GLUT1.	Phosphatidylinositol 3-kinase (PI 3-kinase).	Stimulation	PI 3-kinase promotes the exocytosis of both GLUT4 and GLUT1.	Signaling										Low risk	Unclear risk (Not reported)	Low risk	Unclear risk (Not reported)	Unclear risk (No	Low risk
A.518	10.1128/MCB.00203-06	Anne M. Kong, 2006	Australia	To determine whether endogenous PtdIns(3)P regulates GLUT4 trafficking and to elucidate the metabolic pathways synthesizing PtdIns(3)P at the plasma membrane. Specifically, to analyze the function of endogenous PtdIns(3)P in GLUT4 translocation and/or insertion into the plasma membrane by overexpressing the 72-5ptase enzyme.	3T3-L1 adipocytes.	Genetic: Overexpression of wild-type 72-kDa inositol polyphosphate 5-phosphatase (72-5ptase) or catalytically inactive mutant (D480N) via adenovirus vector or electroporation.<br>Chemical: Stimulation with Insulin (100 nM).<br>Inhibition: PI3K inhibition with Wortmannin (100 nM); Actin disruption with Latrunculin A or Cytochalasin D; Golgi inhibition with Brefeldin A.<br>Co-expression: Co-transfection with biosensors (GFP-PH/ARNO, GFP-FYVE) or regulatory proteins (Rab11, Rab5).	Phosphoinositide Analysis: PtdIns 5-phosphatase assays, Immunoprecipitation, Thin-layer chromatography (TLC).<br>Localization/Translocation: Confocal microscopy monitoring GFP-tagged biosensors (GFP-FYVE/EEA1 for PtdIns (3)P, GFP-PH/PLCδ for PtdIns(4,5)P2).<br>GLUT4 Translocation: GLUT4 translocation assay (counting cells with membrane fluorescence rims), Subcellular fractionation, Plasma membrane sheets assay.<br>Glucose Uptake: 2-Deoxy-D-glucose (2-DOG) uptake assay.<br>Gene/Protein Expression: Northern blot, Immunoblotting.	Insulin Stimulation: 2, 3, 5, 10, 30 minutes.<br>Post-transfection/Infection: 24 or 48 hours.<br>Glucose Uptake: Measured over 20 minutes.	Genetic Comparison: Empty vector (HA-vector) vs. Wild-type 72-5ptase (HA-72-5ptase) vs. Inactive mutant (HA-72-D480N5ptase).<br>State Comparison: Unstimulated/Basal vs. Insulin-stimulated (+INS).<br>Inhibition Comparison: With and without Wortmannin, Latrunculin A, or dominant-negative proteins (Rab11 S25N).	Endogenous PtdIns(3)P at the plasma membrane promotes GLUT4 translocation.	PtdIns(3)P	Stimulation of GLUT4 translocation to the membrane but no increase in glucose uptake.	The 72-5ptase enzyme hydrolyzes PtdIns(3,5)P2 to generate PtdIns(3)P directly at the plasma membrane. This endogenous PtdIns(3)P is sufficient to promote GLUT4 translocation and insertion into the plasma membrane independently of the traditional insulin signaling pathway (requiring no Class I PI3K or PtdIns(3,4,5)P3, and insensitive to Wortmannin). This process relies on actin remodeling (inhibited by Latrunculin A) and the Rab11 pathway, but is independent of TC10 or Rab5. Although GLUT4 is recruited to the membrane, glucose uptake does not increase, suggesting an additional signal is required to activate the transport activity of GLUT4.	Translocation										Low risk	Unclear risk (Not reported)	Low risk	Unclear risk (Not reported)	Unclear risk (No	Low risk
A.519	10.3390/biom13121738	Dimitrios Kioumourtzoglou, 2023	UK	To provide a mechanistic link between insulin receptor-mediated phosphorylation of Syntaxin 4 and the formation of SNARE complexes required for GLUT4 exocytosis.	HeLa cells (expressing GLUT4) and 3T3-L1 adipocytes.	Genetic: Expression of phosphomimetic Syntaxin 4 mutant (Phos-Sx4: Y115E/Y251E).<br>Biochemical: In vitro phosphorylation by Cytosolic Insulin Receptor Kinase (CIRK).	Surface GLUT4: Flow cytometry.<br>Protein Interaction: Proximity Ligation Assay (PLA) for in vivo SNARE interaction.<br>Assembly: In vitro SNARE assembly assay.	Cells: 48 hours post-transfection.<br>Biochemical: Reactions monitored over 20–150 minutes.	Protein Comparison: Wild-type Syntaxin 4 (WT) vs. Phosphomimetic Syntaxin 4 (Phos-Sx4).<br>Cellular Comparison: Transfected vs. Non-transfected cells.	Tyr-115 and Tyr-251 of Syntaxin 4 are direct substrates of activated insulin receptors, and these residues modulate the protein's conformation, thereby regulating the rate of SNARE complex formation for GLUT4 delivery to the cell surface.	Phosphorylated Syntaxin 4	Stimulation	The insulin receptor directly phosphorylates Syntaxin 4 at Tyr-115 and Tyr-251. This phosphorylation induces a conformational change from a "closed" to an "open" state, allowing stronger interaction with SNAP23 and VAMP21, thereby accelerating SNARE complex assembly and facilitating GLUT4 vesicle fusion.	Fusion										Unclear risk (Not reported)	Unclear risk (Not reported)	Low risk	Unclear risk (Not reported)	Unclear risk (No	Low risk
A.520	10.1126/scisignal.aah5085	Maeran Uhm, 2017	USA (USA)	To examine how phosphorylation of the exocyst complex (specifically the Exo84 subunit) by TBK1 kinase regulates insulin-stimulated glucose transport and GLUT4 translocation in adipocytes.	3T3-L1 adipocytes and Adipocyte-specific TBK1 knockout mice (TBK1 AKO)	Genetic: TBK1 knockdown (siRNA), TBK1 knockout (Cre-LoxP), Overexpression of Wild-type TBK1 (WT) or Kinase-dead mutant (K38A), Expression of Exo84 phosphomimetic (S8E) or non-phosphorylatable (S8A) mutants.<br>Chemical: Stimulation with Insulin (10 nM); TBK1 inhibition with Amlexanox or CAY10576; Akt inhibition with Akti-1/2.	Metabolism: [^{14}C]2-DG uptake assay in cells and ex vivo isolated adipose tissue.<br>GLUT4 Translocation: Immunostaining of dual-tagged GLUT4 (MYC-GLUT4-eGFP) to detect surface MYC signal.<br>Interaction/Phosphorylation: Co-immunoprecipitation, Western blot, LC-MS/MS, In vitro kinase assay.<br>Distribution: Subcellular fractionation and sucrose density gradient centrifugation.	Insulin Stimulation: 15–30 minutes.<br>Inhibitor Incubation: 1 hour prior to stimulation.<br>Post-transfection: 24–96 hours.	Genetic Comparison: Control (Scrambled siRNA/WT mice/Vector) vs. Intervention (TBK1 siRNA/TBK1 AKO mice/TBK1 K38A/Exo84 mutants).<br>Chemical Comparison: Basal/No Treatment (NT) vs. Insulin (Ins). Insulin only vs. Insulin + Inhibitor (Amlexanox/Akti).	TBK1 controls GLUT4 vesicle engagement and disengagement from the exocyst, suggesting that exocyst components not only constitute a tethering complex for the GLUT4 vesicle but also act as "gatekeepers" controlling vesicle fusion at the plasma membrane.	TBK1	Stimulation	TBK1 directly phosphorylates the Exo84 subunit of the exocyst complex. This phosphorylation triggers the disengagement of Exo84 from the RalA G-protein and other exocyst components. This "release" step is obligatory for the GLUT4 vesicle (tethered by the exocyst) to proceed to fusion with the plasma membrane. Without TBK1 (or if Exo84 cannot be phosphorylated), GLUT4 vesicles remain "stuck" at the tethering complex and cannot complete exocytosis.	Fusion	Unclear risk (Not r	Low risk	Unclear risk (Not r	Unclear risk (Not r	Unclear risk (Not r	Unclear risk (Not r	Unclear risk (Not r	Low risk	Low risk	Unclear risk (Not reported)	Unclear risk (Not reported)	LOW risk	Unclear risk (Not reported)	Unclear risk (No	Low risk
A.526	10.1210/mend.11.13.0027	Li-Na Cong, 1997	USA (USA)	To investigate the impact of inhibiting endogenous Akt on insulin-stimulated GLUT4 translocation in rat adipocytes, aiming to define the physiological role of Akt in glucose transport.	Primary cultures of rat adipose cells.	Genetic: Transfection of various Akt constructs:<br>* Akt-WT (Wild type).<br>* Akt-myr (Constitutively active, myristoylated).<br>* Akt-K179A (Dominant inhibitory kinase-inactive).<br>* Myr-K179A (Kinase-inactive membrane-targeted).<br>Chemical: Stimulation with Insulin at varying concentrations (0, 0.024, 0.072, 0.3, 60 nM).	GLUT4 Translocation: Use of epitope-tagged GLUT4 (GLUT4-HA) and anti-HA1 mouse monoclonal antibody combined with [^{125}I]-labeled anti-mouse antibody to quantify cell surface GLUT4.<br>Kinase Activity: Akt kinase assay (measuring [^{32}P]ATP incorporation into histone 2B substrate in immunoprecipitates).<br>Protein Expression: Immunoblotting (Western blot).	Post-transfection: 20 hours.<br>Insulin Stimulation: 20 minutes (for GLUT4 experiments) or 2 minutes (for kinase experiments).	Vector Comparison: Control group (empty pCIS2 vector) vs. Akt transfection groups (WT, myr, K179A, Myr-K179A).<br>State Comparison: Basal vs. Insulin-stimulated.<br>Dosage Comparison: Low DNA concentration (4 µg) vs. High concentration (7 µg) for dominant inhibitory mutants.	Akt is not only capable of stimulating the translocation of GLUT4 but that endogenous Akt is likely to play a significant physiological role in insulin-stimulated glucose uptake in insulin targets such as muscle and adipose tissue.	Akt	Stimulation	The study confirms that endogenous Akt is physiologically necessary and critical for regulating insulin-stimulated GLUT4 translocation (demonstrating that Akt is not merely sufficient when overexpressed, but is an essential required component of the native pathway).	Signaling										Unclear risk (Not reported)	Unclear risk (Not reported)	Low risk	Unclear risk (Not reported)	Unclear risk (No	Low risk
A.527	10.1016/j.bbadis.2019.03.010	Alexander W. Fischer, 2019	Germany and USA (USA)	To investigate whether PID1 regulates GLUT4 translocation and function through interaction with the cytoplasmic domain of LRP1.	Primary brown adipocytes from mice and mouse models: Wild-type (WT), Whole-body PID1 knockout, and Adipocyte-specific PID1 knockout.	Environmental: Cold stimulation (6°C, 22°C) vs. Neutral temperature (30°C).<br>Genetic: Pid1 Knockout (whole-body or adipocyte-specific). Lrp1 Knockout (using Cre adenovirus in cells).<br>Chemical: Stimulation with Insulin (10 nM or in vivo injection).<br>Diet: Chow diet vs. High Fat Diet (HFD).	Expression: qPCR, Western blot (PID1, LRP1, GLUT4, pAKT).<br>Localization: Immunofluorescence and Confocal microscopy to assess co-localization and membrane translocation.<br>Interaction: Co-immunoprecipitation (Co-IP) and GST-pulldown.<br>Metabolism: Glucose tolerance test (GTT), Insulin tolerance test (ITT), In vivo [^{3}H]-deoxyglucose uptake.	Insulin Stimulation (Cells): 10 minutes.<br>Insulin Stimulation (In vivo): 10 minutes (for signaling).<br>Glucose Uptake (In vivo): 2 hours post-tracer.<br>HFD Duration: 18–20 weeks.<br>Temperature Acclimatization: 1 week.	Genetic Comparison: WT mice/cells vs. Pid1-/- (whole-body or tissue-specific).<br>Condition Comparison: Basal (no insulin) vs. Insulin-stimulated.<br>Environmental Comparison: 30°C vs. 22°C vs. 6°C.<br>Diet Comparison: Chow vs. HFD.	The data provide evidence that PID1 serves as an insulin-regulated retention adaptor protein controlling translocation of LRP1 in conjunction with GLUT4 to the plasma membrane of adipocytes. Notably, loss of PID1 corrects for insulin resistance-associated hyperglycemia emphasizing its pivotal role and therapeutic potential in the regulation of glucose homeostasis.	PID1	Inhibition	PID1 interacts with the non-phosphorylated NPxY motif on the intracellular domain of LRP1 to retain GLUT4 storage vesicles (GSVs) intracellularly. Insulin triggers the phosphorylation of this motif, causing PID1 to dissociate, thereby allowing GLUT4 vesicles to translocate to the membrane. In the absence of PID1, this "locking" mechanism is lost, resulting in constitutively higher surface GLUT4 levels, which improves glucose tolerance and reduces hyperglycemia even under insulin-resistant conditions (obesity).	Translocation	Unclear risk (Not r	Low risk	Unclear risk (Not r	Unclear risk (Not r	Unclear risk (Not r	Unclear risk (Not r	Unclear risk (Not r	Low risk	Low risk	Unclear risk (Not reported)	Unclear risk (Not r	Low risk	Unclear risk (Not reported)	Unclear risk (No	Low risk
A.528	10.1007/s11010-013-1714-7	Prasenjit Manna, 2013	USA (USA)	To investigate the direct roles of PIP3 and PIP2 in glucose metabolism and GLUT4 translocation in 3T3-L1 adipocytes under high glucose (HG) conditions, and to identify the intermediate signaling pathways.	3T3-L1 adipocytes.	Chemical:<br>* High glucose medium (HG, 25 mM).<br>* Exogenous PIP3 or PIP2 supplementation (1, 5, 10 nM) combined with Histone H1 carrier.<br>* Insulin stimulation (10, 25, 50, 100 nM).<br>Genetic: siRNA knockdown of AKT2 and PKC-ζ.	Glucose Transport: 6-NBDG uptake measurement (fluorescence).<br>Glucose Utilization: Glucose utilization assay (measuring residual glucose in medium).<br>GLUT4 Translocation: Flow cytometry (surface GLUT4).<br>Cell Signaling: Western blot.<br>Cytokines: ELISA (MCP-1, Resistin, Adiponectin).	PIP2/PIP3 Treatment: Pre-incubation for 2 or 4 hours, followed by 20 hours in HG.<br>Glucose Uptake/Utilization: Measured after 20 hours of HG treatment.<br>Surface GLUT4: Measured after treatment.	Lipid Comparison: Control group (HG) vs. PIP3 supplementation vs. PIP2 supplementation.<br>Dose Comparison: Concentrations of 1, 5, 10 nM.<br>Genetic Comparison: Control siRNA vs. AKT2 siRNA vs. PKC-ζ siRNA.<br>Insulin Comparison: Insulin alone vs. Insulin + PIP3 vs. Insulin + PIP2.	This study for the first time demonstrates that PIP3 but not PIP2 plays an important role in GLUT4 upregulation and glucose metabolism mediated by AKT/PKCζ/λ phosphorylation.	PIP3	Stimulation	Exogenous PIP3 (but not PIP2) can directly enter cells and activate the AKT/PKC-ζ/λ signaling pathway to upregulate GLUT4 and improve glucose metabolism under high glucose conditions, independent of or synergistic with endogenous insulin. Specifically, the study indicates that PKC-ζ/λ plays a more critical role than AKT2 in mediating the effects of PIP3 on GLUT4 and glucose utilization.	Signaling										Unclear risk (Not reported)	Unclear risk (Not r	Low risk	Unclear risk (Not reported)	Unclear risk (No	Low risk
A.530	10.1016/j.bbrc.2017.12.064	Bangli Deng, 2018	China (China)	To explore the detailed mechanism by which increased intracellular Ca^{2+} (mimicking exercise/muscle contraction) regulates GLUT4 transport, specifically the role of PKC isoforms and Rab proteins in controlling GLUT4 endocytosis and exocytosis.	L6 myoblast muscle cells stably expressing myc-tagged GLUT4 (GLUT4myc).	Chemical:<br>* Ca^{2+} increase stimulation via Ionomycin (1 μM).<br>* Insulin (100 nM) (used in comparative experiments).<br>Genetic (Gene Silencing):<br>* PKC isoform knockdown (siRNA): siPKCα, siPKCθ, siPKCδ, siPKCε.<br>* Rab protein knockdown (siRNA): siRab8a, siRab10, siRab13, siRab14.<br>* GAP protein knockdown (siRNA): siAS160, siTBC1D1.	Surface GLUT4: Colorimetric assay using anti-myc antibody and OPD in non-permeabilized cells.<br>Endocytosis/Exocytosis: Kinetic assays based on the disappearance or appearance of surface GLUT4myc over time.<br>Cell Signaling: Western blot (p-AS160, p-TBC1D1, PKC, Rab levels).	Ionomycin Stimulation: 30 minutes.<br>Endocytosis Measurement: 0, 5, 10, 15, 20 minutes.<br>Exocytosis Measurement: 0, 5, 10, 15, 20 minutes.<br>Post-siRNA Transfection: 48 hours.	Chemical Comparison: Basal vs. Ionomycin vs. Insulin.<br>Genetic Comparison: Control siRNA (Scramble) vs. Target siRNA (PKC, Rab, GAP).	Ionomycin-promoted exocytosis of GLUT4 is partly reversed by siPKCθ, whereas ionomycin-inhibited endocytosis of GLUT4 requires both siPKCα and siPKCθ. PKCα and PKCθ contribute to ionomycin-induced phosphorylation of AS160 and TBC1D1. Rab13 is required for ionomycin-regulated GLUT4 exocytosis.	PKC and Rab13	Stimulation	Ca^{2+} signaling (via Ionomycin) regulates GLUT4 through two parallel branches:<br>1. Inhibition of Endocytosis: Mediated by PKCα and PKCθ, which phosphorylate AS160 and TBC1D1.<br>2. Stimulation of Exocytosis: Mediated by PKCθ and Rab13. This mechanism is distinct from the Insulin signaling pathway (which primarily relies on Akt and Rab10) or the AMP pathway (involving AMPK).	Signaling										Unclear risk (Not reported)	Unclear risk (Not r	Low risk	Unclear risk (Not reported)	Unclear risk (No	Low risk
A.531	10.1002/jcp.22320	Wenyan Niu, 2010	China and Canada	To explore the role of protein kinase C (PKC), particularly novel PKC isoforms, in regulating contraction-stimulated GLUT4 traffic.	C2C12 myotubes stably expressing myc-tagged GLUT4 (GLUT4myc)	Chemical Stimulation: Carbachol (1 mM) - an acetylcholine analog to induce muscle contraction.<br>Chemical Inhibition:<br>* Conventional/novel PKC inhibitors: GF-109203X, Gö 6983, Ro-31-8425.<br>* Selective conventional PKC inhibitor: Gö 6976.<br>* Translocation inhibitor peptides: myr-PKC epsilon V1-2, myr-PKC delta V1-1.<br>Genetic: siRNA knockdown of PKCε.	Surface GLUT4: Colorimetric assay on non-permeabilized cells or Immunofluorescence.<br>Cell Signaling/Phosphorylation: Western blot (using phospho-substrate or isoform-specific antibodies).<br>Gene Expression: RT-PCR (to determine PKC isoforms).<br>PKC Translocation: Cell fractionation (membrane vs. cytosol) followed by Western blot.	Carbachol Stimulation: 5 minutes (for PKC translocation), 30 minutes (for GLUT4 translocation).<br>Inhibitor Incubation: 20–30 minutes prior to stimulation.<br>Post-siRNA Transfection: 48 hours.	Stimulation Comparison: Basal vs. Carbachol vs. Insulin.<br>Inhibition Comparison: Vehicle (No inhibitor) vs. Inhibitor treated (GF, Gö, Ro, Peptide inhibitors).<br>Genetic Comparison: Scrambled siRNA vs. PKCε siRNA.	These results support a role for novel PKC isoforms, especially PKCε, in contraction-stimulated GLUT4 traffic in skeletal muscle cells.	PKCε	Stimulation	Contraction (mimicked by carbachol) triggers the translocation of novel PKC isoforms (specifically PKCε and PKCδ) to the cell membrane. However, only PKCε is essential for contraction-stimulated GLUT4 transport, whereas PKCδ is not involved (based on specific peptide inhibitor results). This demonstrates a specific role for the PKCε isoform in the contraction signaling pathway leading to glucose uptake, independent of Ca^{2+}-dependent conventional PKCs like PKCα.	Signaling										Unclear risk (Not reported)	Unclear risk (Not reported)	Low risk	Unclear risk (Not reported)	Unclear risk (No	Low risk

A.534	10.1006/excr.2002.5570	Margarita Lorenzo, 2002	Spain (Spain) and USA (USA)	To investigate the role of Phospholipase C-γ in insulin-stimulated glucose transport via the activation of PKC-ζ in brown adipocytes.	Primary brown adipocytes from rats and HeLa cells (expressing GLUT4-GFP).	Chemical:<br>* Insulin stimulation (100 nM).<br>* PLC inhibition with U73122 (10 μM) or inhibitory peptide (Y1021).<br>* PI3K inhibition with Wortmannin (100 nM).<br>* DAG-kinase inhibition with R59949 (10 μM).<br>* Supplementation with Phosphatidic acid (PA).<br>Genetic: Transfection of a dominant-negative mutant of PLCγ (lip-PLCγ) into HeLa cells.	Glucose Transport: 2-deoxy-D-[^{3}H] glucose uptake measurement.<br>GLUT4 Translocation: Western blot (membrane fractionation) and Confocal microscopy (GLUT4-GFP).<br>Cell Signaling: Western blot (p-Insulin Receptor, p-Akt, PKCζ), Immunoprecipitation (PLCγ, PKCζ), Kinase activity assays (PLC, PI3K, PKCζ).<br>Cytoskeleton: Actin staining with phalloidin-TRITC.	Insulin Stimulation: 1, 3, 5, 10, 30 minutes (depending on experiment).<br>Inhibitor Incubation: 30 minutes (U73122, Wortmannin) or 4 hours (inhibitory peptide).<br>Glucose Uptake: Measured over 10 minutes.	Chemical Comparison: Basal vs. Insulin group.<br>Inhibitor Comparison: Insulin vs. Insulin + U73122 (or Wortmannin, R59949). Active U73122 vs. Inactive U73343 (negative control).<br>Genetic Comparison: HeLa cells transfected with empty vector vs. PLC-γ mutant.	In summary, these data indicate that PLCγ, activated at least partially by PI3-kinase, is a link between insulin receptor and PKCζ through the production of PA and could mediate insulin-induced glucose uptake and GLUT4 translocation.	Phospholipase C-γ and Protein Kinase C-ζ	Stimulation	The study proposes a novel signaling pathway where PLCγ acts as a critical intermediate link between PI3K and PKCζ to regulate glucose transport. Specifically: Insulin → PI3K → PLCγ → DAG → (DAG-kinase) → Phosphatidic Acid (PA) → PKCζ → GLUT4 Translocation.<br>A key finding is that PA (a metabolite of DAG produced by PLC) is the direct activator of PKCζ in this pathway, rather than DAG or IP3 directly.	Signaling										Unclear risk (Not reported)	Low risk	Low risk	Unclear risk (Not reported)	Low risk	Low risk
A.535	10.3389/fcell.2022.1033501	Shaun K. Bremner, 2022	UK	To understand the role of Syntaxin 16 (Sx16) and the secretory pathway in adipocyte function by generating Sx16-deficient 3T3-L1 adipocytes using CRISPR/Cas9 technology.	3T3-L1 adipocytes	Genetic: Sx16 knockout using CRISPR/Cas9 (frameshift mutation).<br>Chemical:<br>* Acute insulin stimulation.<br>* Chronic insulin/Hyperinsulinemia (300 nM for 18 hours).<br>* LPS (Lipopolysaccharide) stimulation.	Glucose Transport: 2-[^{3}H]-deoxyglucose uptake measurement.<br>Protein Analysis: SDS-PAGE and Immunoblotting (Western blot) to quantify GLUT4, GLUT1, Sx16, Sx6, VAMP4, Sortilin, IRAP, etc.<br>Gene Expression: RNA extraction and qPCR.<br>Lipid Accumulation: Oil Red O staining.<br>Subcellular Distribution: Subcellular fractionation separating plasma membrane (PM) and intracellular membranes (HDM/LDM).<br>Secretion: ELISA (for MCP-1) and Lactate-Glo Assay (for lactate).	Differentiation: Day 10 or 11 post-induction.<br>Glucose Uptake: Measured over 3 minutes.<br>Acute Insulin Treatment: 30 minutes.<br>Chronic Insulin Treatment: 18 hours.	Genetic Comparison: Wild-type (WT) cells vs. Sx16 Knockout (Sx16 KO) cells.<br>Condition Comparison: Basal vs. Insulin; Control vs. Hyperinsulinemia.	GLUT4 mRNA and protein levels were reduced in Sx16 knockout adipocytes and insulin stimulated GLUT4 translocation to the cell surface was reduced. Strikingly, neither basal nor insulin-stimulated glucose transport were affected. By contrast, GLUT1 levels were upregulated in Sx16 knockout cells. Levels of sortilin and insulin regulated aminopeptidase were also increased in Sx16 knockout adipocytes which may indicate an upregulation of an alternative GLUT4 sorting pathway as a compensatory mechanism for the loss of Sx16. In response to chronic insulin stimulation, Sx16 knockout adipocytes exhibit elevated insulin-independent glucose transport and significant alterations in lactate metabolism. We further show that the adipokine secretory pathways are impaired in Sx16 knockout cells. Together this demonstrates a role for Sx16 in the control of glucose transport, the response to elevated insulin, cellular metabolic profiles and adipocytokine secretion.	Syntaxin 16	Stimulation (of secretory/sorting pathways)	Sx16 exerts pleiotropic effects. Loss of Sx16 triggers a compensatory mechanism by upregulating GLUT1 and other sorting proteins (Sortilin, IRAP), maintaining overall glucose transport despite reduced GLUT4 levels. Additionally, Sx16 plays a critical role in controlling adipokine secretion and metabolic adaptation (lactate metabolism) to chronic hyperinsulinemia.											Unclear risk (Not r	Unclear risk (Not reported)	Low risk	Unclear risk (Not reported)	Unclear risk (No	Low risk
A.537	10.1016/s0014-5793(99)01472-6	Sophie Grillo, 1999	France	To determine the role of PDK1 (3-phosphoinositide-dependent protein kinase 1) and its pleckstrin homology (PH) domain in insulin-stimulated GLUT4 translocation in adipocytes.	Freshly isolated rat adipocytes.	Genetic: Cotransfection of plasmid encoding GLUT4-myc with PDK1-myc (wild-type) or vPH-PDK1-myc (PH domain deletion mutant).<br>Chemical:<br>* Insulin stimulation (100 nM).<br>* Okadaic acid stimulation (1 μM) - a phosphatase 2A inhibitor.	GLUT4 Translocation: Measurement of cell surface GLUT4-myc using anti-myc antibody combined with [^{125}I]-labeled Protein A.<br>PKB Activity: Immunoprecipitation of HA-PKB and kinase activity measurement using Crosstide substrate.	Post-transfection: 16–24 hours.<br>Insulin/Okadaic acid treatment: 30 minutes (for GLUT4 translocation), 10 minutes (for PKB activity).	Genetic Comparison: Control cells (GLUT4-myc only) vs. cells expressing PDK1-myc or vPH-PDK1-myc.<br>Condition Comparison: Basal (no insulin) vs. Insulin vs. Okadaic acid.	Overexpression of PDK1 (3-phosphoinositide-dependent protein kinase 1), a downstream effector of PI 3-kinase, stimulated GLUT4 translocation in adipocytes.	PDK1	Stimulation	PDK1 acts as an intermediate component in the insulin signaling pathway leading to GLUT4 translocation.<br>The PH domain of PDK1 is required for full insulin-stimulated GLUT4 translocation. The PH domain deletion mutant (vPH-PDK1) acts as a dominant negative, likely by preventing Serine 473 phosphorylation of PKB (a PI3K-dependent event that may require the PDK1 PH domain for correct localization or interaction), even though it can still phosphorylate Threonine 308 (causing partial PKB activation and increased basal GLUT4).	Signaling										Unclear risk (Not reported)	Unclear risk (Not reported)	Low risk	Unclear risk (Not reported)	Unclear risk (No	Low risk
A.538	10.1210/endo.138.5.5136	Jean-François Tanti, 1997	France and The Netherlands	To determine the potential role of protein kinase B (PKB) in insulin-stimulated glucose transporter 4 (GLUT4) translocation in adipocytes.	Freshly isolated rat adipocytes and 3T3-L1 adipocytes.	Chemical:<br>* Insulin stimulation (100 nM).<br>* Okadaic acid stimulation (1 mM) – a PP1/PP2A phosphatase inhibitor.<br>* PDGF stimulation (50 ng/ml).<br>Genetic: Transfection of plasmids encoding epitope-tagged GLUT4 (GLUT4-myc) and constitutively active PKB (Gag-PKB).	PKB Activity: Gel shift assay on Western blot and in vitro kinase assay using Crosstide substrate following immunoprecipitation.<br>PI3K Activity: p85 immunoprecipitation followed by lipid kinase assay.<br>GLUT4 Translocation: Measurement of cell surface GLUT4-myc using anti-myc antibody and ^{125}I-labeled Protein A.<br>Subcellular Distribution: Subcellular fractionation into plasma membrane (PM), low-density microsomes (LDM), high-density microsomes (HDM), and cytosol (CYTO), followed by Western blot.	Kinase Activation (Insulin/PDGF): 5 minutes.<br>Okadaic Acid Stimulation: 30 minutes.<br>Post-transfection: 16–24 hours.<br>GLUT4 Translocation: After 30 minutes of incubation with/without insulin.	Stimulus Comparison: Basal vs. Insulin vs. Okadaic acid vs. PDGF.<br>Genetic Comparison: Empty vector (pCIS) transfected cells vs. Gag-PKB transfected cells.	PKB could play a role in promoting GLUT4 appearance at the cell surface following exposure of adipocytes to insulin and okadaic acid stimulation.	Protein Kinase B (PKB/Akt).	Stimulation	The study provides evidence that PKB (Akt) serves as a direct link between PI3K and GLUT4 translocation.<br>1. Activation of PKB (via membrane-targeted Gag-PKB) is sufficient to induce GLUT4 translocation without insulin stimulation.<br>2. Okadaic acid stimulates GLUT4 translocation through a PI3K-independent activation of PKB.<br>3. The failure of PDGF to stimulate GLUT4 translocation may be due to insufficient PKB activation or failure to activate PKB in the correct subcellular compartment (LDM) required for GLUT4 mobilization.	Signaling										Unclear risk (Not reported)	Unclear risk (Not reported)	Low risk	Unclear risk (Not reported)	Unclear risk (No	Low risk
A.540	10.1091/mbc.E02-03-0174	Elena Shumay, 2002	USA (USA)	To investigate the role of pp60Src (Src) kinase in insulin-stimulated β2-adrenergic receptor sequestration and its connection to other insulin signaling pathways such as PI3K and GLUT4 translocation.	A431 human epidermoid carcinoma cells (expressing β2 AR-GFP or GLUT4-GFP), 3T3-L1, and 3T3-F442A mouse adipocytes.	Chemical:<br>* Insulin stimulation (100 nM).<br>* Isoproterenol stimulation (10 μM) – a β-adrenergic agonist.<br>* PI3K inhibitor: LY294002 (20 μM).<br>* Src inhibitor: PP2 (50 nM).<br>* MEK inhibitor: PD98059 (10 μM).<br>Genetic:<br>* Overexpression of β2 AR-GFP or GLUT4-GFP.<br>* Src depletion using antisense morpholinos.<br>* Expression of constitutively active Src mutant (CA-Src).	Localization: Epifluorescence and Confocal microscopy to track GFP.<br>Protein Activation: Western blot with phospho-specific antibody (anti-phospho-Src Y416).<br>Kinase Activity: Src kinase assay (peptide substrate) and PI3K activity assay (TLC lipid product measurement).<br>Protein Interaction: Co-immunoprecipitation.	Insulin Stimulation: 10, 15, 30 minutes (maximal effect at 30 min).<br>Inhibitor Treatment: 30 minutes prior to stimulation.<br>Morpholino Treatment: 48 hours.	Stimulus Comparison: Control vs. Insulin vs. Isoproterenol.<br>Inhibitor Comparison: Insulin alone vs. (Insulin + LY294002) vs. (Insulin + PP2) vs. (Insulin + PD98059).<br>Genetic Comparison: Sense morpholino (Control) vs. Antisense morpholino (Src depletion). Wild-type vs. CA-Src.	Src mediates signals from insulin to phosphatidylinositol 3-kinase and to $β_2$-adrenergic receptor intracellular trafficking.	pp60Src	Stimulation	The study proposes a novel role for Src as a critical intermediate transmitting signals from the insulin receptor to PI3K and the vesicle trafficking machinery.<br>1. Insulin activates Src. This activated Src is required to activate (or maintain) PI3K activity.<br>2. Activated Src phosphorylates Tyrosine residues (Tyr-350/354) on the C-tail of the β2 AR.<br>3. This phosphorylation recruits GRK2 via its SH2 domain, leading to GRK2 membrane translocation, further receptor phosphorylation, and subsequent endocytosis/sequestration.<br>4. This Src → PI3K → Trafficking mechanism also applies to the regulation of GLUT4 translocation.	Signaling										Unclear risk (Not reported)	Unclear risk (Not reported)	Low risk	Unclear risk (Not reported)	Unclear risk (No	Low risk
A.541	10.2337/db14-1402	Rasmus Kjøbsted, 2014	Denmark (Denmark)	To determine whether AMPK activation directly increases skeletal muscle insulin sensitivity for glucose uptake.	Female mice strains: Wild-type (WT) C57BL/6J, Muscle-specific Kinase Dead α2 (AMPK KD), Muscle-specific Double Knockout α1/α2 (AMPK mdKO), Whole-body Knockout (AMPK γ3 KO). Isolated Soleus and Extensor Digitorum Longus (EDL) muscles.	Prior Stimulation: Incubation with AICAR (1 mM) for 50 minutes to activate AMPK (mimicking exercise).<br>Recovery: 4 hours (Soleus) or 6 hours (EDL) recovery period to allow acute AICAR effects to subside.<br>Insulin Stimulation: Incubation with submaximal insulin (100 μU/ml) for 30 minutes post-recovery.<br>Genetic: In vivo gene electro-transfer of TBC1D4 variants (WT, T649A, S711A) into mouse muscle.	Glucose Uptake: [^{3}H]2-deoxyglucose accumulation assay using [^{14}C]mannitol as an extracellular marker.<br>Kinase Activity: AMPK activity assay (specific trimer complexes after immunoprecipitation).<br>Cell Signaling: Western blot (phosphorylation of AMPK, ACC, Akt, TBC1D1, TBC1D4 at specific Ser/Thr sites).	AICAR Stimulation: 50 minutes.<br>Recovery: 4–6 hours.<br>Glucose Uptake: Measured during the last 10 minutes of the 30-minute insulin stimulation.	Genetic Comparison: WT vs. Transgenic strains (AMPK KD, mdKO, γ3 KO).<br>Condition Comparison: Control (No AICAR) vs. Prior AICAR. Basal (No Insulin) vs. Insulin.	AMPK is sufficient to increase skeletal muscle insulin sensitivity. Moreover, TBC1D4 phosphorylation may facilitate the effect of prior AMPK activation to enhance glucose uptake in response to insulin.	AMPK	Stimulation	The study identifies the molecular mechanism by which prior exercise/AICAR enhances insulin sensitivity:1. Activation of AMPK (specifically the α2β2γ3 complex) is a prerequisite.2. Activated AMPK leads to enhanced phosphorylation of TBC1D4 (AS160) at critical regulatory sites (Ser711 and Thr649) during subsequent insulin stimulation.3. This enhanced phosphorylation of TBC1D4 (rather than upstream Akt signaling) correlates directly with increased glucose uptake.	Signaling	Unclear risk (Not r	Low risk	Unclear risk (Not r	Unclear risk (Not r	Unclear risk (Not r	Unclear risk (Not r	Unclear risk (Not r		Low risk	Unclear risk (Not reported)	Unclear risk (Not reported)	Low risk	Unclear risk (Not reported)	Unclear risk (No	Low risk
A.543	10.1074/jbc.274.40.28087	Patrick G. P. Foran, 1999	UK	To investigate the role of SNARE proteins (SNAP-23, synaptobrevin-2/cellubrevin) in insulin- and Protein Kinase B (PKB)-stimulated trafficking of GLUT4, GLUT1, and transferrin receptor (TfR) using Clostridium botulinum toxins (BoNT/B and BoNT/E).	3T3-L1 adipocytes.	Chemical:<br>* Insulin stimulation (200 nM).<br>* Microinjection of BoNT/B toxin (2 μM) or BoNT/E toxin (1 μM).<br>Genetic: Microinjection of plasmids encoding:<br>* GFP-GLUT4 (translocation reporter).<br>* PKB-DD (Constitutively active PKB).<br>* hTfR (Human transferrin receptor).	GLUT4 Translocation: Confocal microscopy monitoring GFP-GLUT4 (counting cells with membrane rims or quantifying membrane fluorescence intensity).<br>GLUT1/TfR Translocation: Immunofluorescence using anti-GLUT1 or anti-hTfR antibodies.<br>SNARE Cleavage: Western blot to assess cleavage of SNARE proteins (SNAP-23, VAMPs) in permeabilized cells.	Microinjection: 16–24 hours prior to stimulation.<br>Insulin Stimulation: 1 hour.<br>Toxin Treatment: Co-injected with plasmids and incubated for 16–24 hours (sufficient for SNARE cleavage).	Stimulus Comparison: Basal vs. Insulin vs. PKB-DD expression.<br>Toxin Comparison: No toxin vs. BoNT/B vs. BoNT/E.<br>Substrate Comparison: GLUT4 vs. GLUT1 vs. TfR.	Insulin induces GLUT4 translocation via two distinct routes, only one of which involves SNAP-23 and synaptobrevin-2/cellubrevin and can be mobilized by PKB-DD. The PKB-, SNAP-23-, and synaptobrevin-2/cellubrevin-independent GLUT4 translocation pathway may involve movement through recycling endosomes, together with GLUT1 and transferrin receptors.	Protein Kinase B (PKB/Akt), SNAREs.	Stimulation	nsulin activates two parallel GLUT4 translocation pathways:<br>1. PKB-dependent, BoNT-sensitive pathway: Requires SNAP-23 and VAMP2/3. This pathway mobilizes GLUT4 from specialized GLUT4 Storage Vesicles (GSVs) that do not contain GLUT1/TfR.<br>2. PKB-independent, BoNT-insensitive pathway: Does not require the targeted SNAREs (or uses different ones). This pathway mobilizes GLUT4 from recycling endosomes, along with GLUT1 and TfR.<br>This explains why constitutively active PKB stimulates only GLUT4 (from GSVs) but not GLUT1/TfR, and why inhibiting GSV SNAREs does not completely block insulin's effect (as the recycling pathway remains active).	Signaling										Unclear risk (Not reported)	Unclear risk (Not reported)	Low risk	Unclear risk (Not reported)	Unclear risk (No	Low risk
A.544	10.1128/MCB.19.6.4008	Qinghua Wang, 1999	Canada	To investigate the role of protein kinase B alpha/Akt1 (PKBα/Akt1) in insulin-stimulated GLUT4 translocation to the cell surface in L6 myoblasts.	L6 myoblasts stably transfected with GLUT4 cDNA carrying an exofacial myc-epitope tag (L6-GLUT4myc myoblasts).	Chemical:<br>* Insulin stimulation (100 nM).<br>* PI3K inhibition with Wortmannin (100 nM).<br>Genetic (Transfection):<br>* Expression of constitutively active PKB (GagPKB).<br>* Expression of dominant-negative PKB mutants: AAA-PKB (K179A, T308A, S473A), AA-PKB (T308A, S473A), A-PKB (K179A).<br>* Expression of dominant-negative PI3K.<br>* Expression of GST-BAD (to measure intracellular kinase activity).	GLUT4 Translocation: Immunofluorescence detection of the myc-epitope on the surface of non-permeabilized cells.<br>Kinase Activity: Immunoprecipitation of PKB followed by in vitro kinase assay using Crosstide as a substrate.<br>Protein Phosphorylation: Western blot measuring phosphorylation levels of BAD (Ser136).<br>Cytoskeleton: Actin staining with Rhodamine-phalloidin to observe actin bundling/ruffling.	Insulin Stimulation: 10 minutes (for kinase activity and BAD phosphorylation), 20 minutes (for GLUT4 translocation), 5 minutes (for actin remodeling).<br>Post-transfection: 48 hours.<br>Serum Deprivation: 5 hours prior to the experiment.	Stimulus Comparison: Basal (no insulin) vs. Insulin.<br>Genetic Comparison: Transfected cells (GFP positive) vs. Non-transfected cells (adjacent).<br>Variant Comparison: Empty vector (pcDNA3) vs. GagPKB vs. AAA-PKB (and other mutants).	These results outline an important role for PKB alpha/Akt1 in the stimulation of glucose transport by insulin in muscle cells in culture.	Protein Kinase B α/Akt1	Stimulation	The study identifies PKB/Akt1 as an essential component downstream of PI3K in the insulin signaling pathway leading to GLUT4 translocation in muscle cells. Activation of PKB is necessary and sufficient (partially) to induce GLUT4 translocation. Importantly, the study demonstrates a bifurcation of PI3K signaling: one branch proceeds through PKB to regulate GLUT4, while another branch (independent of PKB) regulates actin remodeling (membrane ruffling).	Signaling										Unclear risk (Not reported)	Unclear risk (Not reported)	Low risk	Unclear risk (Not reported)	Unclear risk (No	Low risk
A.545	10.1124/mol.104.004200	Jun Chen, 2005	USA (USA)	To assess the role of conventional and novel PKC isoforms (c/nPKC) as well as Protein Kinase D (PKD), specifically PKD3, in glucose transport in L6 myotubes.	L6 myotubes - a rat skeletal muscle cell line.	Chemical:<br>* PMA (Phorbol 12-myristate 13-acetate): Short-term treatment (100 nM, 10–20 min) to activate c/nPKC or long-term (1 μM, 24 h) to down-regulate c/nPKC.<br>* Insulin (100 nM).<br>* Inhibitors: Go6976, Go6983, GF 109203X, Ro 31-8220, Wortmannin.<br>Genetic:<br>* Adenovirus carrying wild-type PKD3 (wt-PKD3) or dominant-negative PKD3 (DN-PKD3).<br>* siRNA targeting PKD3 (PKD3-targeted siRNA).<br>* Transfection with GFP-GLUT4 to track translocation.	Glucose Transport: [^{3}H]2-deoxy-D-glucose uptake measurement.<br>Protein Analysis: Western blot (PKC, PKD, Akt, p-Akt, Actin).<br>Kinase Activity: Immunoprecipitation followed by in vitro kinase assay (PKCζ, PKD3) using peptide substrates and [γ-^{32}P]ATP.<br>GLUT4 Translocation: Confocal microscopy tracking GFP-GLUT4 and immunostaining of endogenous GLUT1.	PMA Treatment: Short-term (10–20 min) or Long-term (24 hours).<br>Insulin Stimulation: 20 minutes.<br>Inhibitor Treatment: 1 hour (insulin added during the last 20 minutes).<br>Post-Adenovirus/siRNA: 24–48 hours.	Stimulus Comparison: Basal (no insulin) vs. Insulin.<br>PMA Comparison: No PMA vs. Short-term PMA vs. Long-term PMA.<br>Inhibitor Comparison: Control vs. Go6976/Go6983/GF 109203X/Ro 31-8220.<br>Genetic Comparison: Adv-null (empty vector) vs. Adv-wt-PKD3 vs. Adv-DN-PKD3; Scramble siRNA vs. PKD3 siRNA.	PKD, specifically PKD3, directly contributes to insulin-independent basal glucose uptake in L6 skeletal muscle cells.	Protein Kinase D3 (PKD3)	Stimulation (Basal)	PKD3 is a constitutively active kinase in L6 muscle cells, responsible for regulating basal (insulin-independent) glucose transport levels and the translocation of GLUT4 (and GLUT1) in the resting state. This mechanism operates independently of the insulin signaling pathway (PI3K/Akt) and independently of conventional/novel PKC isoforms (c/nPKC).	Signaling										Unclear risk (Not reported)	Unclear risk (Not reported)	Low risk	Unclear risk (Not reported)	Unclear risk (No	Low risk
A.546	10.1074/jbc.272.48.30075	Mary L. Standaert, 1997	USA (USA) and Spain (Spain)	To investigate the role of Protein Kinase C-zeta (PKC-ζ) as a downstream effector of PI3-kinase in insulin-stimulated glucose transport in rat adipocytes.	Freshly isolated rat adipocytes.	Chemical:<br>* Insulin stimulation (10 nM or other concentrations).<br>* PI3K Inhibition: LY294002 (250 μM) and Wortmannin (100 nM).<br>* PKC Inhibition: RO 31-8220 (various doses); PKC-ζ pseudosubstrate.<br>* Activators: Phosphatidylinositols (PI-3,4-P2, PI-3,4,5-P3, PI-4,5-P2), pYXXM-containing peptide (PI3K activation).<br>* TPA Treatment (1 μM overnight) to deplete DAG-sensitive PKCs.<br>Genetic: Electroporation of plasmids encoding HA-GLUT4 alongside PKC-ζ variants (wild-type, constitutive, dominant-negative) or Δp85 (dominant-negative PI3K).	PKC-ζ Activity: Immunoprecipitation (IP) of PKC-ζ and in vitro kinase assay using a specific peptide substrate ([159Ser] PKC-ε(AA153–164)-NH2).<br>Glucose Transport: 2-deoxy-D-[^{14}C] glucose uptake measurement (2-DOG uptake).<br>GLUT4 Translocation: Measurement of HA-GLUT4 on the cell surface using anti-HA antibody and ^{125}I-secondary antibody.<br>Protein Phosphorylation: Measurement of ^{32}P incorporation into PKC-ζ (autophosphorylation) in vitro or in vivo (cells labeled with ^{32}P-orthophosphate).	Insulin Stimulation: 10 minutes (for kinase activity), 30 minutes (for glucose transport and GLUT4 translocation).<br>TPA Treatment: Overnight.	Stimulus Comparison: Basal (no insulin) vs. Insulin.<br>Inhibitor Comparison: Control vs. (Insulin + LY294002/Wortmannin/RO 31-8220/Pseudosubstrate).<br>Genetic Comparison: Empty vector vs. WT PKC-ζ vs. Constitutive PKC-ζ vs. Dominant-negative PKC-ζ.	insulin activates PKC-ζ through PI 3-kinase, and PKC-ζ may act as a downstream effector of PI 3-kinase and contribute to the activation of GLUT4 translocation.	Protein Kinase C-zeta (PKC-ζ)	Stimulation	The study establishes PKC-ζ as a critical link between PI3-kinase and GLUT4 translocation in the insulin signaling pathway.<br>1. Insulin activates PI3-kinase → generates D3-polyphosphoinositide lipids (such as PIP3) → these lipids directly activate PKC-ζ (and promote autophosphorylation).<br>2. Activated PKC-ζ subsequently participates in stimulating the GLUT4 translocation machinery.<br>3. This activation is independent of DAG-sensitive PKCs (as TPA treatment had no effect).	Signaling										Unclear risk (Not reported)	Unclear risk (Not reported)	Low risk	Unclear risk (Not reported)	Unclear risk (No	Low risk
A.548	10.1007/s00109-007-0232-z	Xiao-Jun Liu, 2007	China	To investigate whether PKCζ regulates GLUT4 translocation through cytoskeletal remodeling in CHO cells.	Chinese Hamster Ovary cells (CHO-K1 cells) and CHO-K1 cells cotransfected with myc-tagged GLUT4.	Chemical:<br>* Insulin stimulation.<br>* Actin disruption with Latrunculin B (2 μM).<br>* PKCζ inhibition with pseudosubstrate (PS) (5 μM)<br>Genetic: Overexpression of PKCζ using pcDNA3-HA-PKCζ vector.	SDS-PAGE and Immunoblotting (Western blot).<br>Fluorescence microscopy and Laser confocal microscopy.	Insulin Stimulation: 2, 5, 20 minutes (for Western blot) or 15 minutes (for microscopy/translocation).<br>Latrunculin B Treatment: 2 hours.<br>PS Treatment: 45 minutes.<br>Serum Starvation: 3 hours prior to treatment.	Basal group vs. Empty vector control group (pcDNA3).<br>Untreated control group (Control/Untreated).	PKCζ mediates the stimulatory effect of insulin on GLUT4 translocation through its interaction with actin cytoskeleton.	Protein Kinase C-ζ (PKCζ)	Stimulation	PKCζ mediates the stimulatory effect of insulin on GLUT4 translocation via interaction with the actin cytoskeleton and induction of actin remodeling.<br>Specifically, insulin promotes the colocalization of PKCζ and actin.	Signaling										Unclear risk (Not r	Unclear risk (Not r	Low risk	Unclear risk (Not re	Unclear risk (No	Low risk

A.549	10.1210/mend.13.12.0393	Liora Braiman, 1999.	Isarel	To investigate whether insulin-induced activation of Protein Kinase C delta (PKC-δ) participates in mediating insulin's effects on glucose transport in skeletal muscle.	Rat skeletal muscle cells.	Chemical:<br>* Insulin stimulation.<br>* Selective pharmacological inhibition with Rottlerin.<br>Genetic: Overexpression of wild-type and dominant-negative mutant PKC isoforms using Adenovirus vectors.	Western Blot: Assessment of protein translocation and phosphorylation.<br>Kinase Activity: Measurement following immunoprecipitation.<br>Glucose Transport: Radioactive glucose uptake assay.	Phosphorylation/Translocation: Measured at 1, 5, 10, and 30 minutes post-insulin stimulation.<br>Glucose Uptake: Incubated for 15 minutes following insulin stimulation.	Control Comparison: Control vs. Insulin-stimulated (IN) group.<br>Inhibitor Comparison: Rottlerin-treated vs. Untreated.<br>Isoform Comparison: Comparison between isoforms.<br>Genetic Comparison: Cells expressing Wild-type PKC vs. Dominant Negative (D.N.) mutant.	Insulin activates PKC and activated PKC is a major signaling molecule in insulin-induced glucose transport.	Protein Kinase C-δ (PKC-δ)	Stimulation	Insulin induces tyrosine phosphorylation of PKC-δ and promotes its translocation from the cytoplasm to the plasma membrane, resulting in increased kinase activity.<br>This activated PKC-δ specifically regulates GLUT4 translocation.	Signalling										Unclear risk (Not n	Unclear risk (Not n	Unclear risk (Not n	Unclear risk (Not re	Unclear risk (No	Unclear risk (Not reported)
A.550	10.1091/mbc.E05-10-0969	Li-Zhong Liu, 2006	China	To investigate the effect of PKC-ζ on actin remodeling and glucose transport, and to elucidate the relationship between PKC-ζ, Rac-1, and actin remodeling in L6 muscle cells.	Differentiated L6 rat myotubes expressing myc-tagged GLUT4 (L6myc cells) or overexpressing PKC-ζ.	Chemical:<br>* Insulin stimulation (100 nmol/l).<br>* Inhibitors: Wortmannin (PI3K inhibitor) and PKC pseudosubstrate peptide.<br>Genetic:<br>* Stable overexpression of PKC-ζ.<br>* Transient transfection of Rac-1 mutants: Constitutively active (Rac-1 CA) and Dominant negative (Rac-1 DN).	Imaging: Laser confocal scanning microscopy.<br>Enzymatic: PKC enzyme activity assay.<br>GLUT4 Quantification: Optical colorimetric assay (OD 492 nm) for cell surface GLUT4.<br>Glucose Transport: 2-deoxy-[³H] deoxyglucose uptake assay.<br>Protein Analysis: Western blot and subcellular fractionation.	Insulin Stimulation: 5 minutes (for microscopy and enzyme activity) or time-course (0, 5, 10, 20 minutes) for GLUT4 translocation and glucose uptake.<br>Inhibitor Treatment: Wortmannin (25 minutes), Pseudosubstrate peptide (55 minutes).	State Comparison: Basal (Insulin-) vs. Insulin-stimulated (Insulin+).<br>Cell Type Comparison: Control L6myc cells vs. L6myc-PKC-ζ overexpression cells.<br>Inhibitor Comparison: No inhibitor vs. Wortmannin (WM) vs. Pseudosubstrate (PS).	PKC mediates the effect of insulin on glucose transport through actin remodeling in muscle cells.	Protein Kinase C-ζ (PKC-ζ)	Stimulation	Insulin activates PKC-ζ in a PI3K-dependent manner.<br>Activated PKC-ζ spatially interacts with Rac-1 and actin to promote actin remodeling. This newly formed actin structure serves as a scaffold to recruit GLUT4-containing vesicles to the plasma membrane.	Signalling										Unclear risk (Not n	Unclear risk (Not n	Low risk	Unclear risk (Not re	Unclear risk (No	Low risk
A.552	10.1007/s00125-005-1819-y	C. P. Hodgkinson, 2005	Anh	To identify missing links between upstream insulin-regulated kinases (such as PKC-ζ) and the GLUT4 vesicle trafficking machinery.	3T3-L1 adipocytes and L6 muscle cells.	Chemical:<br>* Insulin stimulation (100 nmol/l).<br>* PI3K inhibitor (LY294002).	Interaction Screening: Yeast two-hybrid screening.<br>In Vitro Interaction: GST pull-downs.<br>In Vivo Interaction: Co-immunoprecipitation.<br>Protein Analysis: Western blotting.<br>Metabolism: Glucose uptake assay.<br>Translocation: Membrane fractionation to assess GLUT4 translocation.	Protein Interaction (IP): 5 minutes post-insulin stimulation.<br>Glucose Uptake/Translocation: 20 minutes post-insulin stimulation.	State Comparison: Control/Basal vs. Insulin-stimulated.<br>Genetic Comparison: Cells expressing full-length Munc18c (FL) vs. Mutant Munc18c lacking PKC-ζ binding ability.<br>Inhibitor Comparison: Cells with and without LY294002.	We identified a physiological interaction between Munc18c and PKCζ regulated by insulin. The results show that PKCζ regulates Munc18c and suggest a model whereby insulin triggers the docking of PKCζ to Munc18c, resulting in enhanced GLUT4 translocation to the plasma membrane.	Protein Kinase C-ζ (PKC-ζ)	Stimulation	A direct, insulin-regulated interaction between PKC-ζ and Munc18c was discovered (increased 3-fold with insulin).<br>* The interaction site involves the N-terminus of PKC-ζ and amino acids 295–338 of Munc18c.<br>* Proposed Mechanism: Insulin triggers the docking of PKC-ζ to Munc18c, which reduces the affinity of Munc18c for Syntaxin-4 (releasing the "clamp"). This allows VAMP-2 to bind Syntaxin-4 for membrane fusion.<br>* Munc18c is heavily phosphorylated, but PKC-ζ is not the direct kinase responsible for this phosphorylation.	Docking										Low risk	Unclear risk (Not n	Unclear risk (Not n	Unclear risk (Not re	Unclear risk (No	Unclear risk (Not reported)
A.554	10.1074/jbc.M313745200	Takeshi Yoshizaki, 2004.	Japan	To clarify the physiological role of Protein Phosphatase-2C-α (PP2C-α) in insulin signaling and glucose metabolism in adipocytes, specifically examining whether it acts as a positive or negative regulator.	3T3-L1 adipocytes.	Chemical: Insulin stimulation (1, 10, 100 ng/ml).<br>Genetic:<br>* Adenovirus-mediated overexpression of Wild-type PP2C-α (WT).<br>* Adenovirus-mediated overexpression of Dominant negative mutant D174G-PP2C-α (phosphatase-dead).	Phosphorylation: Immunoblotting (Western blot) for Akt, ERK, IRS-1, p70S6K.<br>Enzyme Activity: In vitro PI 3-kinase lipid kinase assay; PP2C phosphatase activity assay.<br>Glucose Transport: 2-Deoxyglucose uptake assay.<br>Translocation: Subcellular fractionation for GLUT4.<br>Interaction: Co-immunoprecipitation.	Insulin Signaling: 2, 5, 10, 20, 30 minutes post-stimulation.<br>Glucose Uptake: Measured over 10 minutes post-stimulation.<br>Post-infection: 48 hours.	State Comparison: Basal vs. Insulin.<br>Genetic Comparison: Control (Ad-LacZ) vs. WT-PP2C alpha expressing group vs. D174G-PP2C alpha expressing group.	PP2C is a new positive regulator of insulin sensitivity that acts through a direct activation of PI3K in 3T3-L1 adipocytes.	Protein Phosphatase-2C-α (PP2C-α)	Stimulation	PP2C-α interacts directly and constitutively with the p85 regulatory subunit of PI3K.<br>The phosphatase activity of PP2C-α directly dephosphorylates p85 (at serine/threonine residues), leading to the direct activation of the lipid kinase activity of PI3K.	Siganlling										Unclear risk (Not n	Unclear risk (Not n	Low risk	Unclear risk (Not re	Unclear risk (No	Low risk
A.555	10.1074/jbc.272.12.8026	Hui Chen, 1997.	USA	To directly assess the ability of PTP1B and Syp to regulate insulin-stimulated GLUT4 translocation in a physiological insulin target cell (adipocytes).	Primary cultured rat adipocytes.	Genetic: Transient transfection of PTP1B constructs (Wild-type PTP1B-WT, Catalytically inactive PTP1B-C/S) or Syp constructs (Wild-type Syp-WT, Mutant Syp-C/S). Co-transfection with epitope-tagged GLUT4 (GLUT4-HA).<br>Chemical: Insulin stimulation at various concentrations.	Surface GLUT4: Epitope-tagged GLUT4 assay using anti-HA monoclonal antibody and secondary antibody.<br>Protein Expression: Immunoblotting (Western blot).<br>Enzyme Activity: PTPase activity measured by pNPP hydrolysis.	Stimulation: 30 minutes post-insulin.<br>Gene Expression: 20 hours post-electroporation.	Genetic Comparison: Control group (pCIS2 empty vector + GLUT4-HA) vs. Experimental groups (PTP1B-WT/C/S or Syp-WT/C/S).<br>Stimulus Comparison: Maximal insulin (60 nM) vs. No insulin (Basal).	Our data suggest that PTP1B may be a negative regulator of insulin-stimulated glucose transport, while Syp may play a minor role as a positive mediator of insulin's metabolic effects.	PTP1B (Protein-tyrosine phosphatase 1B)	Inhibition	PTP1B acts as a potent inhibitor of insulin signaling leading to GLUT4 translocation (reducing surface GLUT4 even in basal and stimulated states).<br>Conversely, Syp acts as a positive mediator, as inhibition of endogenous Syp (via Syp-C/S) reduces GLUT4 translocation.<br>This demonstrates specificity among tyrosine phosphatases in regulating different aspects of metabolic insulin signaling.	Signalling										Unclear risk (Not n	Unclear risk (Not n	Low risk	Unclear risk (Not re	Unclear risk (No	Unclear risk (Not reported)
A.562	10.1083/jcb.201111091	Yu Chen, 2012.	China	To investigate the involvement of Rab proteins in GLUT4 trafficking, focusing on Rab proteins that directly regulate the delivery of GLUT4 storage vesicles (GSV) to the plasma membrane.	3T3-L1 adipocytes	Chemical: Insulin stimulation (100 nM).<br>Genetic:<br>* Expression of fluorescence-tagged Rab proteins, IRAP-pHluorin probe, and Myosin-Va.<br>* Knockdown of Rab10 and Rab14 using shRNA/siRNA.<br>* Expression of Rab10 mutants (Constitutively active Rab10-QL) and AS160.	Imaging: Dual-color Total Internal Reflection Fluorescence (TIRF) microscopy to observe docking and colocalization at the plasma membrane.<br>Fusion Assay: IRAP-pHluorin fusion assay (measuring fusion events based on pH change).<br>Analysis: Colocalization analysis and Western blotting.	Fusion/Translocation: 3 minutes post-insulin stimulation, or continuous time-course monitoring over 30 minutes.<br>Knockdown/Transfection Efficiency: 48 hours.	State Comparison: Basal vs. Insulin-stimulated.<br>Genetic Comparison: Control cells (Scrambled shRNA) vs. Knockdown cells (shRab10/siRab14).<br>Cell Type Comparison: Fibroblasts (no GSV) vs. Adipocytes (GSV present).<br>Isoform Comparison: Comparison between different Rabs (Rab4, Rab8, Rab10, Rab14, etc.).	Rab10 directly facilitated GSV translocation to and docking at the PM. Rab14 mediated GLUT4 delivery to the PM via endosomal compartments containing transferrin receptor (TfR), whereas Rab4A, Rab4B, and Rab8A recycled GLUT4 through the endosomal system.	RAB10	Stimulation	Rab10 (not Rab14 or others) is the primary factor associated with GSVs that is mobilized to the plasma membrane upon insulin stimulation (via inhibition of AS160).<br>Once activated and bound to GSVs, Rab10 interacts with the molecular motor Myosin-Va.<br>Myosin-Va acts as a bridge, helping GSVs traverse the cortical actin network to reach the docking site at the membrane.	translocation										Unclear risk (Not n	Unclear risk (Not n	Low risk	Unclear risk (Not re	Unclear risk (No	Low risk
A.564	10.4161/cib.23779	Yu Chen & Jennifer Lippincott-Schwartz, 2013	USA	To identify specific Rab proteins that mediate the trafficking of GLUT4 storage vesicles (GSVs) to the plasma membrane (PM) upon insulin stimulation.	3T3-L1 adipocytes.	Chemical: Insulin stimulation.<br>Imaging: Total Internal Reflection Fluorescence (TIRF) microscopy for live-cell imaging.<br>Labeling: Use of IRAP-pHluorin probe to selectively mark GSVs undergoing fusion.<br>Genetic: Expression of constitutively active Rab10 mutant.	TIRF Microscopy: Direct observation of GLUT4 trafficking and fusion events within the ~150 nm evanescent field adjacent to the plasma membrane.	Post-insulin stimulation.	Rab Protein Comparison: Rab10 vs. Rab14 vs. Rab4A vs. Rab4B vs. Rab8A.<br>Compartment Comparison: GLUT4 Storage Vesicles (GSVs - Rab10 positive, TfR negative) vs. Endosomal compartments (Rab14 positive, TfR positive).	Rab10 as the only Rab protein that binds this compartment [GSV]. Rab14 was found to label transferrin-positive, endosomal compartments containing GLUT4. Rab4A, 4B and 8A, were found to mediate GLUT4 intra-endosomal recycling	Rab10	Stimulation	Rab10 is the unique Rab protein residing on GSVs and directly regulates the transport of GSVs to the cell membrane upon insulin stimulation.<br>Rab14 regulates GLUT4 transport through endosomal compartments (containing TfR).<br>Rab4A, 4B, and 8A are responsible for the intra-endosomal recycling of GLUT4 to return GLUT4 to the GSVs.	translocation										Unclear risk (Not n	Unclear risk (Not n	Low risk	Unclear risk (Not re	Unclear risk (No	Unclear risk (Not reported)
A.567	10.1016/j.cmet.2007.03.001	Hiroyuki Sano, 2007	USA	To demonstrate the role of Rab10 in the insulin signaling pathway as a downstream target of AS160.	3T3-L1 adipocytes.	Genetic: Overexpression of GTP-bound Rab10 mutant (Rab10-QL active mutant). Knockdown of Rab10 using siRNA/shRNA. Rescue of AS160-knockdown cells by subsequent Rab10 knockdown.<br>Chemical: Insulin stimulation (1 nM and 170 nM).	Surface GLUT4: Fluorescence measurement determining the ratio of surface to total GLUT4.<br>Protein Analysis: Western Blotting (expression levels and phosphorylation).<br>TIRF Microscopy: Measurement of GLUT4 within the evanescent field near the plasma membrane.<br>Exocytosis Kinetics: HA-antibody uptake assay.	GLUT4 Translocation: 30 minutes post-insulin.<br>Phosphorylation (Akt, AS160): 15 minutes post-insulin.<br>Genetic Efficiency: 24–48 hours post-transfection/infection.<br>Exocytosis Kinetics: 0 to 90 minutes.	State Comparison: Basal (no insulin) vs. Insulin-stimulated.<br>Genetic Comparison: Non-specific shRNA (Control) vs. Rab10 wild-type expressing cells vs. Rab10 knockdown cells.	Rab10 functions as a downstream target of AS160 in the insulin-signaling pathway that regulates GLUT4 translocation in adipocytes.	Rab10	Stimulation	Rab10 is a direct substrate of the GAP domain of AS160.<br>Insulin (via Akt) inhibits the GAP activity of AS160, thereby increasing the level of active GTP-bound Rab10. This active Rab10 is required for the mobilization of GLUT4-containing vesicles.	Docking										Unclear risk (Not n	Unclear risk (Not n	Low risk	Unclear risk (Not re	Unclear risk (No	Low risk
A.572	10.1073/pnas.1009523107	Yi Sun, 2010.	Canada	To determine the specific roles of Rab8A, Rab10, and Rab13 in insulin-regulated GLUT4 translocation in L6 muscle cells.	L6 rat skeletal muscle cells.	Chemical: Insulin stimulation (100 nM).<br>Genetic: siRNA knockdown specific for Rab8A, Rab13, Rab10. Expression of plasmids containing Rab sequences.	Rab Activation: Affinity precipitation using p67phox-PBD or MICAL-L1 binding domains to detect active Rab.<br>Surface GLUT4: Biotinylation or colorimetric immunoassay.<br>Protein Analysis: Western blotting (levels and phosphorylation of Akt, AS160).<br>Localization: Fluorescence microscopy.	Rab Activation/Phosphorylation: 0, 2, 5, 10, 20, and 30 minutes post-insulin.<br>GLUT4 Translocation: 20 minutes post-insulin.	State Comparison: Basal vs. Insulin.<br>Genetic Comparison: Control siRNA vs. Specific siRNA (Rab8A, Rab13, Rab10).<br>Isoform Comparison: Comparison of efficacy between Rab8A, Rab10, and Rab13.	Rab13 and Rab8A are Rab GTPases activated by insulin, and acting downstream of AS160, they regulate the traffic of GLUT4 vesicles, potentially operating at distinct steps and intracellular sites.	Rab8A and Rab13	Stimulation	Insulin activates both Rab8A and Rab13.<br>Rab8A is activated prior to Rab13, and the activation of Rab13 is dependent on Rab8A (knockdown of Rab8A reduces Rab13 activation, but not vice versa).<br>Rab13 acts at a late stage, regulating the movement of GLUT4 from the cortical actin zone to the plasma membrane.	Docking										Unclear risk (Not n	Unclear risk (Not n	Unclear risk (Not n	Unclear risk (Not re	Unclear risk (No	Unclear risk (Not reported)
A.573	10.1002/1873-3468.12555	Hanbing Li et al., 2017	China	To identify which specific Rab proteins participate in insulin-stimulated GLUT4 translocation in C2C12 myoblasts and to clarify the functional role of Rab8A.	C2C12 mouse myoblasts.	Genetic: Overexpression of Rab8A mutants: Dominant-negative (T22N), Constitutively active (Q67L), and Wild-type (WT). siRNA gene silencing of Rab8A. Rescue with RNAi-resistant Rab8A.	Imaging: Total Internal Reflection Fluorescence Microscopy (TIRFM) to observe colocalization and exocytosis kinetics.<br>Quantification: Surface to total GLUT4 fluorescence ratio.<br>Colocalization: Pearson correlation coefficient analysis.	Measurement: 30 minutes post-insulin stimulation.<br>Post-transfection: 18–24 hours.	State Comparison: Basal state vs. Insulin state.<br>Genetic Comparison: Wild-type C2C12 or Scramble siRNA (Control) vs. Transfected/Knockdown cells.	Rab8A (but not Rab10 or Rab11) exhibits significant colocalization with GLUT4 in C2C12 cells. The dominant-negative Rab8A (T22N) significantly reduces insulin-stimulated GLUT4 translocation, whereas the constitutively active form (Q67L) enhances this process. Knockdown of Rab8A inhibits GLUT4 translocation, and re-expression of Rab8A rescues this function. Rab8A resides on GLUT4 vesicles and appears at the exocytosis site immediately prior to fusion pore opening.	Rab8A	Stimulation	Rab8A (but not Rab10 or Rab11) shows substantial colocalization with GLUT4 in C2C12 cells.<br>Rab8A participates directly in the exocytosis event of GLUT4 vesicles at the plasma membrane.<br>Rab8A signal appears on the GLUT4 vesicle just before the fusion pore opening and disappears once the vesicle has fully fused with the cell membrane.	Fusion										Unclear risk (Not n	Unclear risk (Not n	Low risk	Unclear risk (Not re	Unclear risk (No	Low risk
A.576	10.1016/j.bbrc.2006.12.140	Shuhei Ishikura et al., 2007	Canada	To determine which Rab proteins (specifically Rab8A, Rab10, and Rab14) are physiological targets of AS160 mediating GLUT4 translocation in muscle cells, by examining their ability to rescue translocation inhibition caused by the 4P-AS160 mutant.	L6 myoblasts.	Genetic: Co-transfection of plasmids encoding the 4P-AS160 mutant with GFP-Rab variants (Wild-type or Constitutively active forms of Rab8A, Rab10, Rab14).<br>Chemical: Insulin stimulation.	Surface GLUT4: Laser scanning fluorescence microscopy to detect surface GLUT4myc.	Measurement: Post-insulin stimulation (20 minutes).<br>Post-transfection: 15–18 hours.	State Comparison: Basal vs. Insulin-stimulated.<br>Genetic Comparison: Control cells (4P-AS160 only) vs. Cells co-expressing 4P-AS160 with Rab variants.	Co-transfection of GFP-Rab8A with 4P-AS160 completely prevents the reduction in surface GLUT4myc levels caused by 4P-AS160 during insulin stimulation. Rab14 also restores the insulin response of GLUT4myc almost completely to the maximal levels of non-transfected cells. In contrast, GFP-Rab10 is completely unable to restore the reduction in surface GLUT4 caused by 4P-AS160. The structurally active form Rab8Q67L also has a similar rescue effect, while Rab10Q68L does not.	Rab8A and Rab14	Stimulation	Rab8A and Rab14 act downstream of AS160 in L6 myoblasts. When overexpressed (or in their active forms), they can bypass the blockade created by the 4P-AS160 mutant, demonstrating that under normal conditions, AS160 inhibits these specific Rabs to retain GLUT4 intracellularly. This contrasts with adipocytes where Rab10 is often the primary target.	Translocation										Unclear risk (Not n	Unclear risk (Not n	Low risk	Unclear risk (Not re	Unclear risk (No	Low risk
A.578	10.1371/journal.pone.0155292	Nobuyuki Takenaka et al., 2016	Japan	To provide in vivo evidence confirming that FLJ00068 functions downstream of Akt2 as a regulator of Rac1 in mouse skeletal muscle.	Skeletal muscle of adult male C57BL/6 mice and L6-GLUT4 rat myoblasts.	In Vivo Gene Transfer: Electroporation of plasmid DNA (GLUT4 reporter, Myr-p110α, Myr-Akt2, FLJ68ΔN) and siRNA into mouse gastrocnemius muscle.<br>Gene Silencing: Specific siRNA (#1 and #2) to inhibit FLJ00068 expression.<br>Signaling Activation: Intravenous insulin injection or expression of constitutively active mutants of PI3K (Myr-p110α) and Akt2 (Myr-Akt2).	GLUT4 Reporter Assay: Measurement of GLUT4 translocation to the sarcolemma in isolated muscle fibers via Myc/GFP fluorescence intensity ratio.<br>Rac1 Activation Assay: In situ detection of activated Rac1 using a specific polypeptide probe and immunofluorescence.<br>Subcellular Fractionation & Immunoblotting: Cytosol/membrane separation and Western blot.<br>Immunohistochemical Staining: Localization of proteins in tissue sections.	Gene Transfer: 5 days prior to experiment.<br>Fasting: 16 hours.<br>Insulin Stimulation: 30 minutes (for fractionation) or immediately prior to muscle excision (IV injection).	Genetic Comparison: Control siRNA vs. FLJ00068 siRNA (#1, #2).<br>State Comparison: Basal (Unstimulated) vs. Insulin-stimulated.<br>Expression Comparison: Wild-type protein expression vs. Constitutively active mutants (Myr-p110α, Myr-Akt2).	Knockdown of FLJ00068 markedly diminished GLUT4 translocation to the sarcolemma induced by insulin or constitutively active mutants (Myr-p110α, Myr-Akt2). Insulin and active mutants caused Rac1 activation at the muscle fiber periphery, which was abrogated by FLJ00068 knockdown. Insulin stimulated the translocation of FLJ00068 from the cytosol to the cell periphery (plasma membrane) in both L6 cells and mouse muscle. A novel polypeptide probe method allowed detection of activated Rac1 in frozen muscle sections.	FLJ00068	Stimulation	Insulin stimulates the translocation of FLJ00068 to the plasma membrane. This event is obligatory for FLJ00068 to activate Rac1 downstream of Akt2, subsequently leading to GLUT4 translocation. FLJ00068 acts as a critical link between the Akt2 signaling node and the cytoskeletal remodeling machinery (Rac1).	Signaling	Unclear risk (Not n		Unclear risk (Not n		Unclear risk (Not n	Unclear risk (Not n	Unclear risk (Not n	Unclear risk (Not n	Low risk	Unclear risk (Not n	Unclear risk (Not n	Unclear risk (Not n	Unclear risk (Not re	Unclear risk (No	Unclear risk (Not reported)
A.585	10.1016/j.cmet.2008.02.008	Yvonne Ng et al., 2008	Australia	To determine if rapid and specific activation of Akt2 is "sufficient" to stimulate GLUT4 translocation to the plasma membrane, addressing the debate on whether additional signaling pathways alongside Akt2 are required.	3T3-L1 adipocytes.	Specific Activation: Use of A/C heterodimerizer (Rapalog) to activate an engineered artificial Akt2 (iAkt2).<br>Control Stimulation: Insulin (100 nM).<br>Inhibition: Wortmannin (PI3K inhibitor) to verify iAkt2 independence from upstream PI3K.	Glucose Transport: 2-deoxy-D-[³H] glucose uptake assay.<br>GLUT4 Translocation: Plasma membrane sheet assay (confocal microscopy) and TIRF microscopy on live cells for kinetics.	Signaling: 2, 5, 10, 20, 30 minutes post-rapalog.<br>Glucose/GLUT4: Measured after 20–30 minutes.	State Comparison: Basal vs. Rapalog-treated vs. Insulin-treated.<br>Inhibitor Comparison: Rapalog + Wortmannin vs. Insulin + Wortmannin.	Rapalog induced rapid iAkt2 activation (maximal at 10 min) and phosphorylation of downstream substrates (AS160, GSK3) comparable to insulin. iAkt2 activation increased glucose transport and GLUT4 translocation to levels equivalent to insulin stimulation. Crucially, the effect of iAkt2 was not inhibited by Wortmannin (PI3K inhibitor), whereas the insulin effect was completely blocked, demonstrating iAkt2 acts downstream of PI3K.	Akt2	Stimulation	The study definitively proves that activation of Akt2 alone (without parallel signals from the insulin receptor or PI3K, and without Akt1) is sufficient to fully trigger the GLUT4 translocation machinery and glucose uptake to physiological levels. This establishes Akt2 as the master switch for this process in adipocytes	Signaling										Low risk	Unclear risk (Not n	Unclear risk (Not n	Unclear risk (Not re	Unclear risk (No	Unclear risk (Not reported)
A.589	10.1210/me.2008-0111	Jacqueline Stöckli et al., 2008	Australia	To determine the role of phosphorylation in regulating the association of AS160 (TBC1D4) with cellular membranes, and the mechanism by which this localization change affects GLUT4 translocation.	3T3-L1 adipocytes.	Stimulation: Insulin (100 nM) or PDGF.<br>Mutant Expression: Expression of AS160/TBC1D4 mutants:<br>* 4P mutant: Phosphorylation-deficient (S318A, S588A, T642A, S751A).<br>* R/K mutant: PTB domain mutant (R109K) preventing phosphoinositide/membrane binding.<br>Chemical: Wortmannin (PI3K inhibitor), ATP-gammaS.	Subcellular Fractionation: Separation of Low-Density Microsomes (LDM) and Cytosol followed by Western blot analysis.<br>In Vitro Binding Assay: Using recombinant GST-TBC1D4 with LDM.<br>GLUT4 Translocation Quantification: Plasma membrane lawn assay on cells expressing HA-GLUT4 or GFP-GLUT4.	Measured after 20 minutes of insulin stimulation.	State Comparison: Basal (Unstimulated) vs. Insulin.<br>Genetic Comparison: Cells expressing Wild-type AS160 (WT) vs. Cells expressing mutants (4P, R/K).<br>Inhibitor Comparison: With Wortmannin vs. Without Wortmannin.	In its ground state, AS160 binds to vesicles containing GLUT4. Insulin dissociates AS160 from the membrane into the cytoplasm.<br>The 4P mutation (non-phosphorylated) is retained on the LDM membrane even in the presence of insulin and strongly inhibits GLUT4 translocation.<br>The R/K mutation (damaged PTB domain) is located primarily in the cytoplasm and does not inhibit GLUT4 translocation (null-like effect).<br>Direct phosphorylation of AS160 is necessary but insufficient to dissociate it from the membrane; the involvement of cytoplasmic factors (such as protein 14-3-3) that bind to phosphorylated AS160 is required to "pull" it out of the membrane.	AS160 (TBC1D4)	Inhibition (in basal state)	In the basal state, AS160 binds to GLUT4 vesicles via its PTB domain. Insulin activates Akt, which phosphorylates AS160 on GLUT4 vesicles. This phosphorylation generates binding sites for 14-3-3 proteins. The binding of cytosolic 14-3-3 proteins to phosphorylated AS160 is required to "extract" or dissociate AS160 from the vesicle membrane into the cytosol, thereby removing its inhibitory GAP activity and allowing GLUT4 translocation. Phosphorylation alone is necessary but not sufficient for release; cytosolic factors (14-3-3) are required.	Signaling										Unclear risk (Not n	Unclear risk (Not n	Low risk	Unclear risk (Not re	Unclear risk (No	Unclear risk (Not reported)
A.591	10.1074/jbc.273.50.33876	Debbie C. Thurmond et al., 1998	USA	To investigate the functional role of Munc18c in modulating insulin-stimulated GLUT4 vesicle translocation, specifically testing the hypothesis that Munc18c acts as an inhibitor of SNARE interaction.	3T3-L1 adipocytes.	Overexpression: Transfection of plasmids encoding Munc18c (Flag-Munc18c, eGFP-Munc18c), Munc18b, or Syntaxin 4.<br>Stimulation: Insulin (100 nM).<br>Protein Interaction: Yeast three-hybrid system to test competition between Munc18c, VAMP2, and Syntaxin 4.	Subcellular Fractionation: Western blot of membrane/cytosol fractions.<br>Yeast Two/Three-hybrid Analysis: Assessment of protein-protein interactions and competition.<br>Confocal Microscopy: Observation of GLUT4-eGFP and eGFP-IRAP translocation.<br>Plasma Membrane Sheet Assay: Quantification of endogenous surface GLUT4/GLUT1 via immunofluorescence.	Stimulation: Insulin for 30 minutes.<br>Measurement: Performed 40–48 hours post-electroporation recovery.	State Comparison: Basal (no insulin) vs. Insulin.<br>Genetic Comparison: Control (empty pcDNA3 vector) vs. Munc18b overexpression vs. Munc18c overexpression.<br>Cargo Comparison: GLUT4/IRAP (insulin-responsive) vs. GLUT1 (constitutive).	Munc18c directly competes with VAMP2 for binding sites on Syntaxin 4 (mutually exclusive interaction).<br>Overexpression of Munc18c (but not Munc18b) strongly inhibits insulin-stimulated translocation of GLUT4 and IRAP/vp165 to the cell membrane.<br>Overexpression of Munc18c does not affect the circulation of GLUT1.<br>Insulin stimulation reduces the amount of Munc18c bound to Syntaxin 4 (causing complex dissociation), although the total amount of Munc18c at the membrane remains unchanged (suggesting Munc18c has moved to a different binding site on the membrane).	Munc18c	Inhibition	Munc18c inhibits docking/fusion by locking Syntaxin 4 and preventing VAMP2 binding (mutually exclusive interaction). Insulin stimulates the dissociation of Munc18c from Syntaxin 4 and sequesters Munc18c to a distinct site on the plasma membrane, thereby freeing Syntaxin 4 to interact with VAMP2 and proceed with fusion.	Docking										Unclear risk (Not n	Unclear risk (Not n	Low risk	Unclear risk (Not re	Unclear risk (No	Unclear risk (Not reported)

A.593	10.1016/j.yexcr.2013.12.011	Richard Stall et al., 2014	USA	To elucidate the relationship between Myosin IIA (MyoIIA) and filamentous actin (F-actin), specifically examining their colocalization at the plasma membrane under insulin stimulation, to determine if MyoIIA interacts with F-actin to regulate GLUT4 fusion at the plasma membrane.	3T3-L1 adipocytes.	Stimulation: Insulin (100 nM).<br>Pharmacological Inhibition:<br>* Blebbistatin (MyoIIA ATPase inhibitor).<br>* ML-7 (Myosin Light Chain Kinase - MLCK inhibitor).<br>* Latrunculin B (LatB - actin polymerization inhibitor).<br>* BAPTA-AM (intracellular calcium chelator).	Confocal Microscopy: Observation and quantification of MyoIIA and F-actin colocalization at the plasma membrane.<br>Glucose Uptake: 2-[1,2-³H]-deoxy-D-glucose uptake assay.<br>Western Blot: Determination of Myosin Light Chain phosphorylation (p-MLC) levels.<br>Image Analysis: Pixel fluorescence intensity quantification to assess F-actin redistribution.	Stimulation: Insulin for 10–30 minutes.<br>Inhibitor Pre-treatment: 30–45 minutes prior.	State Comparison: Basal vs. Insulin.<br>Treatment Comparison: Insulin alone vs. Insulin + Inhibitors (Blebbistatin, ML-7, BAPTA-AM).<br>Calcium Condition Comparison: BAPTA-AM vs. A23187.	Insulin stimulates a significant increase in the colocalization of MyoIIA and F-actin at the plasma membrane. Inhibition of MyoIIA activity (via Blebbistatin) or inhibition of myosin light chain phosphorylation (via ML-7) attenuates glucose uptake and prevents cortical actin loss—a requisite step for GLUT4 membrane access. Latrunculin B treatment reduces MyoIIA membrane recruitment, indicating MyoIIA requires F-actin for proper localization. Furthermore, MyoIIA/F-actin colocalization is calcium-dependent; calcium chelation (BAPTA-AM) blocks this interaction and MLC phosphorylation, whereas calcium ionophore treatment (A23187) rescues MLC phosphorylation even in the absence of insulin.	Myosin IIA	Stimulation	Insulin activates MLCK via Calcium signaling, leading to phosphorylation of the Myosin IIA regulatory light chain (RLC). This phosphorylation promotes the interaction of MyoIIA with cortical F-actin. The contraction or interaction of this Actomyosin complex is required to breach the cortical actin barrier (cortical actin remodeling/loss), creating access for GLUT4 vesicles to dock and fuse with the plasma membrane.	Fusion										Unclear risk (Not re	Unclear risk (Not re	Low risk	Unclear risk (Not rep	Unclear risk (No	Unclear risk (Not reported)	
A.594	10.1371/journal.pone.0077248	Shelly Woody et al., 2013	USA	To identify the upstream regulators of Myosin Light Chain Kinase (MLCK) during insulin-stimulated glucose uptake and to determine which Myosin II isoform is required for this process.	3T3-L1 adipocytes.	Stimulation: Insulin (100 nM).<br>Gene Intervention (siRNA): Knockdown of Myosin IIA, Myosin IIB, MLCK, CaMKII delta, ERK2.<br>Chemical/Inhibition:<br>* BAPTA-AM (intracellular calcium chelator).<br>* A23187 (Calcium ionophore).<br>* U0126 (MEK/ERK inhibitor).<br>* KN-62 (CaMKII inhibitor).	Glucose Uptake: [1-¹⁴C]-2-deoxy-D-glucose uptake using radioactive isotope.<br>Western Blot: Protein expression levels and phosphorylation status (p-MLCK, p-RLC, ERK, CaMKII).<br>Confocal Microscopy: Localization and colocalization of GLUT4 and Myosin IIA.	Glucose Uptake: Measured over 10 minutes post-insulin stimulation (total insulin time usually 20 min).<br>siRNA Treatment: 72 hours post-electroporation.<br>Inhibitor Treatment: Pre-incubation 15–20 minutes before insulin.	State Comparison: Basal (Unstimulated) vs. Insulin.<br>Genetic Comparison: Scramble siRNA (Control) vs. Target siRNA (MyoIIA, ERK2, CaMKII delta).	Knockdown of Myosin IIA (60% reduction) decreased insulin-stimulated glucose uptake by 40%, whereas Myosin IIB knockdown had no additive effect. Insulin stimulates a 4-fold increase in MLCK phosphorylation. Calcium chelation (BAPTA-AM) completely abolished glucose uptake, inhibited MLCK and RLC phosphorylation, and prevented the translocation of Myosin IIA and GLUT4 to the plasma membrane. Conversely, increasing intracellular calcium with A23187 (without insulin) was sufficient to stimulate MLCK and RLC phosphorylation and Myosin IIA translocation, but insufficient to induce GLUT4 translocation or increase glucose uptake. Inhibition of ERK (via U0126 or ERK2 siRNA) reduced MLCK phosphorylation, blocked Myosin IIA translocation, and decreased glucose uptake. Inhibition of CaMKII with KN-62 reduced glucose uptake and RLC phosphorylation, but specific knockdown of the CaMKII delta isoform did not affect MLCK phosphorylation or glucose uptake.	Myosin Light Chain Kinase (MLCK)	Stimulation	Insulin activates MLCK via two simultaneous signaling pathways: increased intracellular Calcium and activation of MAPK/ERK (specifically ERK2). Activated MLCK subsequently phosphorylates the regulatory light chain (RLC) of Myosin IIA, leading to the translocation of Myosin IIA to the plasma membrane. This event is necessary (but not sufficient in the absence of other GLUT4 signals) to support fusion or membrane remodeling for GLUT4.	Fusion										Unclear risk (Not re	Unclear risk (Not re	Low risk	Unclear risk (Not rep	Unclear risk (No	Low risk	
A.599	10.1091/mbc.E12-10-0725	Hiroyasu Hatakeyama and Makoto Kanzaki, 2013	Japan	To dissect the functional role of Tbc1d1, specifically focusing on GLUT4 liberation, using single-molecule nanometry of GLUT4 combined with a cell-based reconstitution model.	3T3-L1 fibroblasts.	Stimulation: AICAR (1 mM, AMPK activator), Insulin (100 nM), Ionomycin (Calcium increase), or Photolysis of caged-Ca²⁺ (NPE).<br>Sequence: Sequential treatment (AICAR first, wash, then Insulin) or simultaneous treatment.<br>Mutant Expression: Expression of Tbc1d1 mutants: S237A (AMPK phosphorylation defective), T596A (Akt-phosphorylation defective), R125W and F168V (PTB1 domain mutants).	GLUT4 Nanometry: Single-molecule tracking using quantum dots to calculate intracellular GLUT4 velocity and diffusion coefficient.<br>Western Blotting: Determination of Tbc1d1 phosphorylation levels at Ser-237 and Thr-596.<br>Calcium Imaging: Monitoring intracellular Ca²⁺ using fluo-4 indicator.	AICAR: 30 minutes.<br>Insulin: 30 minutes.<br>Wash: 60–120 minutes post-AICAR removal.<br>Ca²⁺: Immediate (30 seconds) and 10 minutes post-photolysis.	State Comparison: Basal vs. Stimulated (AICAR/Insulin/Ca²⁺).<br>Pre-treatment Comparison: Naive cells vs. AICAR-pretreated cells.<br>Genetic Comparison: Wild-type Tbc1d1 (WT) vs. Mutants.	In the basal state, insulin alone failed to liberate static GLUT4 in Tbc1d1-expressing cells (unlike AS160). Tbc1d1 acquires insulin responsiveness (to liberate GLUT4) only after prior stimulation with AICAR (mimicking exercise) or increased intracellular Ca²⁺. This "memory" or "acquisition" of insulin responsiveness requires phosphorylation at Ser-237 (AMPK site) and the integrity of the PTB1 domain. PTB1 domain mutants (R125W, F168V) completely abolish this acquisition, despite normal initial AICAR response. Thr-596 phosphorylation (Akt/insulin site) is required for the actual GLUT4 liberation step once Tbc1d1 is in the responsive mode.	Phosphorylated Tbc1d1	Stimulation (via Priming)	Tbc1d1 is initially refractory to insulin. Phosphorylation at Ser-237 (by AMPK or Ca²⁺) acts as a priming signal. This signal, mediated via the PTB1 domain, switches Tbc1d1 into a state where it becomes responsive to a subsequent insulin signal (Thr-596 phosphorylation) to liberate GLUT4. This "memory" persists for a period after the priming signal has ceased.	Translocation										Unclear risk (Not re	Unclear risk (Not re	Low risk	Unclear risk (Not rep	Unclear risk (No	Low risk	
A.602	10.1006/bbrc.1995.1509	Kei Kotani et al., 1995	Japan	To investigate the role of PI 3-kinase in GLUT4 translocation in 3T3-L1 adipocytes by comparing the activation capabilities of insulin versus EGF, and examining the effect of microinjecting a dominant-negative p85 mutant (Δp85).	3T3-L1 adipocytes.	Stimulation: Insulin (0.1 μM or 1 μM) and EGF (1 μM).<br>Microinjection: Recombinant p85 regulatory subunit of PI 3-kinase: Wild-type (Wp85) or Dominant-negative mutant (Δp85 - lacking p110 binding site).	PI 3-Kinase Activity: Immunoprecipitation with anti-p85 antibody followed by TLC lipid kinase assay.<br>GLUT4 Translocation: Plasma membrane lawn assay combined with GLUT4 immunofluorescence staining.	PI 3-Kinase Activity: 15 minutes post-growth factor incubation.<br>GLUT4 Translocation: 10 minutes post-insulin stimulation.	Stimulus Comparison: Basal vs. Insulin vs. EGF.<br>Injection Comparison: Wp85 injection vs. Δp85 injection vs. No injection (None)/Dye only.	Insulin induced a 3-fold increase in PI 3-kinase activity, whereas EGF had no effect (despite both activating the MAPK pathway).<br>Insulin robustly stimulated GLUT4 translocation, whereas EGF failed to induce GLUT4 translocation.<br>Microinjection of the dominant-negative Δp85 mutant inhibited insulin-stimulated GLUT4 translocation by approximately 80%.<br>Microinjection of wild-type Wp85 had no effect on GLUT4 translocation.	Phosphoinositide 3-kinase (PI 3-kinase)	Stimulation	PI 3-kinase is identified as the specific intermediate signal for insulin in activating GLUT4 in 3T3-L1 adipocytes, distinguishing it from the MAPK pathway (activated by both insulin and EGF but not leading to translocation). Blocking the p85-p110 interaction (via Δp85) abolishes the insulin signal to GLUT4.	Signaling										Unclear risk (Not re	Unclear risk (Not re	Low risk	Unclear risk (Not rep	Unclear risk (No	Unclear risk (Not reported)	
A.605	10.1021/bi049222q	Mary L. Standaert et al., 2004	USA	To examine the importance of pYXXM motifs on Cbl (specifically Tyr-371 and Tyr-731) in binding the p85 subunit of PI 3-kinase and Crk protein, and their role in insulin-stimulated aPKC activation and glucose transport.	3T3-L1 adipocytes.	Mutant Expression: Adenovirus or electroporation of plasmids encoding Wild-type Cbl (WT) or Tyrosine-to-Phenylalanine mutants at positions 371 (F371), 731 (F731), or both (F371/731).<br>Stimulation: Insulin (100 nM or 10 nM).<br>Control: LacZ Adenovirus.	Glucose Transport: [³H]2-deoxyglucose uptake.<br>Enzyme Activity: Immunoprecipitation kinase assays for aPKC and PI 3-kinase.<br>Protein Interaction: Immunoprecipitation and Western blot for Cbl binding to p85, PI3K, Crk, and Flotillin.<br>Subcellular Fractionation: Low-density membrane (LDM) vs. Cytosol separation.<br>Confocal Microscopy: GLUT4 and aPKC translocation.	Glucose Transport: 10 minutes post-insulin (48 hours post-infection).<br>Kinase/Binding: 5–10 minutes post-insulin.	State Comparison: Basal vs. Insulin.<br>Genetic Comparison: LacZ (Control) vs. WT Cbl vs. Mutants (F371, F731).	Insulin stimulated Tyrosine phosphorylation of Cbl and the association of Cbl with the p85 subunit of PI 3-kinase, Crk, and CAP. Expression of Cbl mutants (F371, F731) significantly reduced Cbl binding to p85 and Crk, and inhibited the activation of Cbl-associated PI 3-kinase and aPKC. These mutants also inhibited insulin-stimulated glucose transport and the translocation of GLUT4/aPKC to the plasma membrane.	Cbl Protein	Stimulation	Cbl, when phosphorylated at Tyr-371 and Tyr-731, serves as a direct docking site for the p85 subunit of PI 3-kinase. This Cbl-PI3K complex is localized to lipid rafts via Cbl's interaction with CAP/Flotillin. Here, PI 3-kinase generates local PIP3 to activate aPKC and promote GLUT4 translocation. This pathway operates in parallel but is spatially distinct from the IRS-1/PI3K pathway.	Signaling										Unclear risk (Not re	Unclear risk (Not re	Low risk	Unclear risk (Not rep	Unclear risk (No	Low risk	
A.608	10.1242/jcs.007310	Gavin I. Welsh et al., 2007	UK	To explore insulin regulation of Rab11-interacting proteins class I (Rip11, RCP, FIP2) and determine the specific role of Rip11 in GLUT4 trafficking and glucose uptake in adipocytes.	3T3-L1 adipocytes.	Stimulation: Insulin (100 nM), PDGF (50 ng/ml).<br>Gene Intervention:<br>* Overexpression: GFP-Rip11, GFP-RCP, GFP-FIP2, mRFP-Rip11.<br>* Knockdown: siRNA Rip11 (Rip11A, Rip11B).<br>* Mutants: Rip11-I630E (Rab11 binding defective), MyrPKB.<br>Inhibition: Wortmannin (PI3K inhibitor).	Confocal Microscopy: Translocation of Rip11 and colocalization with GLUT4.<br>Glucose Transport: 2-deoxyglucose uptake assay.<br>GLUT4 Fusion/Translocation: HA-GLUT4-GFP reporter assay distinguishing total (GFP) vs. surface (HA) GLUT4.<br>mRNA Quantification: Real-time quantitative RT-PCR.	Glucose Uptake: 48 hours post-siRNA; 30 minutes insulin.<br>Protein Interaction: 10 minutes insulin prior to lysis.	State Comparison: Basal vs. Insulin vs. PDGF vs. MyrPKB.<br>Genetic Comparison: Vector Control vs. Rip11 Overexpression; Scramble siRNA vs. Rip11 siRNA.	Only Rip11 (not RCP or FIP2) translocates to the plasma membrane in response to insulin; this is PI3K and PKB dependent. Rip11 partially colocalizes with GLUT4 in the perinuclear region but not with recycling endosome (TfR) or lysosome markers. Overexpression of Rip11 inhibits cell surface GLUT4 appearance, specifically blocking the fusion step of translocated vesicles. siRNA knockdown of Rip11 significantly inhibits insulin-stimulated glucose uptake. Rip11 complexes with AS160; insulin induces AS160 phosphorylation and dissociation from Rip11.	Rip11	Stimulation (Regulation of Fusion)	Rip11 acts as a scaffold protein binding AS160 (RabGAP). In the basal state, the Rip11-AS160 complex may maintain Rab inactivity or tether vesicles. Upon insulin stimulation, AS160 is phosphorylated and dissociates from Rip11, allowing the fusion of GLUT4 vesicles with the plasma membrane. Rip11 directly regulates this fusion step rather than just translocation.	Fusion										Unclear risk (Not re	Unclear risk (Not re	Low risk	Unclear risk (Not rep	Unclear risk (No	Unclear risk (Not reported)	
A.610	10.1042/BJ20150218	Nobuyuki Takenaka et al., 2015	Japna	To examine the role of the small GTPase RalA downstream of Rac1 in insulin signaling in skeletal muscle fibers, and to elucidate the mechanism by which Rac1 stimulates GLUT4 translocation.	L6 myoblasts.	Knockdown: siRNA silencing of RalA or Myo1c.<br>Stimulation: Insulin (100 nM or 1 mU/ml).<br>Inhibition: Wortmannin (PI3K inhibitor).	GLUT4 Translocation:<br>* Muscle fibers: Myc/ECFP ratio quantification using myc-GLUT4-ECFP reporter.<br>* L6 cells: TIRF microscopy using GLUT4myc7-GFP.<br>Glucose Transport: 2-Deoxyglucose uptake assay.<br>GTPase Activity: Pull-down assay using GST-Sec5(RBD) for RalA-GTP and GST-PAK1(CRIB) for Rac1-GTP.<br>Interaction: Immunoprecipitation.	Translocation: 20 minutes post-insulin.<br>RalA Activation: 2–30 minutes (peak at 5 min).<br>Gene Expression: 3–6 days (fibers) or 48h (L6 cells).	State Comparison: Basal vs. Insulin.<br>Genetic Comparison: WT vs. Muscle-specific Rac1-KO (m-Rac1-KO).<br>Intervention Comparison: Control vs. RalA-ON/RalA-siRNA/Myo1c-siRNA.	Negatively dominant RalA expression (S28N) or RalA knockdown significantly reduces GLUT4 translocation and insulin-stimulated glucose uptake in mouse muscle fibers and L6 cells.<br>Insulin stimulates RalA activation; this activation is inhibited by Wortmannin (PI3K inhibitor) and completely suppressed in the muscle of knockdown Rac1 mice (m-Rac1-KO).<br>Structurally active Rac1 expression (L61) strongly stimulates GLUT4 translocation, but this effect is blocked by RalA knockdown.<br>Myo1c has been identified as a novel effector of RalA: RalA interacts with Myo1c in a GTP-dependent manner, and Myo1c knockdown also inhibits GLUT4 translocation similarly to RalA inhibition.	Rac1	Stimulation	In skeletal muscle, Rac1 acts upstream to activate RalA. Once activated, RalA interacts with and recruits the molecular motor Myo1c (Myosin 1c) to facilitate the transport of GLUT4 vesicles to the cell membrane. This provides the mechanistic explanation for Rac1-mediated GLUT4 regulation.	Translocation										Unclear risk (Not re	Unclear risk (Not re	Low risk	Unclear risk (Not rep	Unclear risk (No	Low risk	
A.616	10.1074/jbc.272.41.25839	Yasushi Kaburagi et al., 1997	Japan	To investigate the role of endogenous substrates (specifically IRS-1 and pp60) in regulating insulin-stimulated glucose transport and GLUT4 translocation by analyzing adipocytes isolated from IRS-1 knockout mice.	Primary adipocytes.	Stimulation: Insulin at various concentrations.<br>Genotype: Wild-type vs. IRS-1 null mice.<br>Inhibition: Wortmannin (100 nM) to inhibit PI 3-kinase.	PI 3-Kinase Activity: Immunoprecipitation with anti-phosphotyrosine antibody followed by kinase assay.<br>Immunoblotting: Tyrosine phosphorylation of IRS-1, IRS-2, pp60 and p85 association.<br>Glucose Transport: 3-O-methyl-D-[U-¹⁴C] glucose uptake.<br>GLUT4 Translocation: Membrane fractionation and Western Blot.	Signaling: 2 minutes post-insulin.<br>Glucose Uptake: 30 minutes post-insulin (with prior wortmannin if applicable).	Genotype Comparison: Wild-type vs. IRS-1 null.<br>State Comparison: Basal vs. Insulin.<br>Inhibitor Comparison: Untreated vs. Wortmannin.	In IRS-1 null adipocytes, insulin-induced PI 3-kinase activity was 54% of wild-type levels. pp60 was the major tyrosine-phosphorylated protein associated with PI 3-kinase in null cells, whereas IRS-2 phosphorylation was almost undetectable (unlike in muscle/liver). Maximal insulin-stimulated glucose transport and GLUT4 translocation were preserved at 52% and 68% of wild-type levels, respectively. Wortmannin completely abolished glucose transport in both groups. Basal glucose transport and cell surface GLUT4 were significantly elevated in IRS-1 null cells.	Insulin Receptor Substrate 1 (IRS-1)	Stimulation	In adipocytes lacking IRS-1, the protein pp60 serves as the primary alternative substrate to bind and activate PI 3-kinase to maintain GLUT4 translocation, rather than IRS-2 (which compensates in other tissues).	Signaling	Unclear risk (Not re	Low risk	Unclear risk (Not re	Unclear risk (Not re	Unclear risk (Not re	Unclear risk (Not re	Unclear risk (Not re	Unclear risk (Not re	Low risk	Unclear risk (Not re	Unclear risk (Not re	Low risk	Unclear risk (Not rep	Unclear risk (No	Unclear risk (Not reported)	
A.618	10.1074/jbc.275.11.8240	Masatoshi Kawanishi, 2000	Japan	To determine the necessity of SNAP23 for insulin-stimulated GLUT4 translocation and to investigate its role in mediating the formation of the syntaxin4-VAMP2 complex.	Differentiated 3T3-L1 adipocytes (for functional assays) and COS cells (for protein interaction assays).	Overexpression: Adenovirus-mediated expression of SNAP23 variants: Wild-type (WT), C-terminal deletion mutants (ΔC8, ΔC49).<br>Stimulation: Insulin (100 nM) and Endothelin-1 (10 nM).	Interaction: Immunoprecipitation and Immunoblot analysis (Western Blot).<br>Function: 2-Deoxy-D-Glucose Transport Assay.<br>Localization: Confocal Immunofluorescence Microscopy.<br>Surface Exposure: Plasma Membrane Lawn Assay.	Glucose transport measured after 20 minutes of insulin stimulation.	State Comparison: Basal vs. Stimulated (Insulin/Endothelin-1).<br>Variant Comparison: WT SNAP23 vs. Mutants (ΔC8, ΔC49).	These results demonstrate that SNAP23 contributes to insulin-dependent trafficking of GLUT4 to the plasma membrane in 3T3-L1 adipocytes by mediating the interaction between t-SNARE (syntaxin4) and v-SNARE (VAMP2).	SNAP23	Stimulation	SNAP23 acts as an obligatory bridge enabling the binding of Syntaxin4 to VAMP2 to form a ternary complex; Syntaxin4 cannot bind VAMP2 in the absence of SNAP23.<br>Inhibitory Mechanism of SNAP23-ΔC8: This mutant can bind to Syntaxin4 but fails to bind VAMP2, thereby competitively blocking Syntaxin4 from participating in functional ternary complexes with endogenous VAMP2.	Fusion										Unclear risk (Not re	Unclear risk (Not re	Low risk	Unclear risk (Not rep	Unclear risk (No	Low risk	
A.619	10.1016/j.cellsig.2014.07.002	Nobuyuki Takenaka, 2014	Japan	To clarify the role of the guanine nucleotide exchange factor (GEF) FLJ00068 in Rac1 activation mediated by Akt2 in relation to GLUT4 translocation in mouse skeletal muscle and cultured muscle cells.	L6-GLUT4 myocytes derived from adult male rat and mouse myoblasts, and muscle-specific rac1 knockout mice.	In Vitro (L6): Insulin stimulation; Overexpression of constitutively active mutants of Akt2, PI3K, and FLJ00068; siRNA knockdown of Rac1, Akt2, and FLJ00068.<br>In Vivo: Electroporation of plasmids into gastrocnemius muscle.	GLUT4 Translocation: Fluorescence quantification.<br>Rac1 Activation: Activation-specific probe.<br>Protein Analysis: Western Blot; Immunofluorescence microscopy; RT-PCR.	Cells: 2 hours serum-starvation; 15 minutes insulin stimulation (for phosphorylation).<br>Mice: 5 days post-electroporation; 16 hours fasting prior to sampling.	Genetic Comparison: Control siRNA vs. Target siRNA; Wild-type mice (rac1+/+ HSA-Cre) vs. Knockout mice (m-rac1-KO).<br>State Comparison: Basal vs. Insulin.	- Continuously activated FLJ00068 causes GLUT4 migration via a Rac1-dependent and Akt2-independent mechanism in L6 cells.<br>- The Rac1 activation and GLUT4 migration induced by continuously activated phosphoinositide 3-kinase were inhibited by reduced FLJ00068 expression.<br>- In mouse gastrocnemius muscle, continuously activated FLJ00068 actually causes GLUT4 migration to the sarcolemma membrane.<br>- Detection of Rac1 activation after continuously activated FLJ00068 expression in gastrocnemius muscle by immunofluorescence microscopy.	FLJ00068	Stimulation	Akt2 does not directly activate Rac1 but acts through FLJ00068. Constitutively active FLJ00068 can induce Rac1 activation and GLUT4 translocation even without Akt2 activity, indicating it lies downstream of Akt2. Conversely, FLJ00068 has no retrograde effect on Akt2 phosphorylation.	Signaling										Unclear risk (Not re	Unclear risk (Not re	Low risk	Unclear risk (Not rep	Unclear risk (No	Low risk	
A.621	10.1128/MCB.15.10.5403	Michael J. Quon, 1995	USA	To investigate the roles of 1-phosphatidylinositol 3-kinase (PI 3-kinase) and Ras, two signaling proteins downstream of tyrosine phosphorylation, in regulating GLUT4 translocation.	Isolated rat adipose cells.	Genetic: Co-transfection of expression vectors for PI 3-kinase mutants, Ras mutants (pCIS-L61-ras and pCIS-ν-ras) along with epitope-tagged GLUT4 (GLUT4-HA).<br>Chemical: Wortmannin treatment (PI 3-kinase inhibitor) and Insulin stimulation.	Surface GLUT4: Quantification of epitope-tagged GLUT4 (GLUT4-HA) on the cell surface using anti-HA monoclonal antibody (12CA5) combined with radiolabeled anti-mouse IgG.	Transfection: 20 hours prior to experiment.<br>Stimulation: Insulin treatment for 30 minutes.	Control: Cells co-transfected with empty vector (pCIS2 or SRα) and GLUT4-HA.<br>Inhibition: Control cells treated with Wortmannin.<br>Mutant: Cells overexpressing constitutively active Ras (L61-ras).	In addition, treatment of control cells with wortmannin (an inhibitor of PI 3-kinase) abolished the ability of insulin to recruit epitope-tagged GLUT4 to the cell surface. In contrast, overexpression of a constitutively active mutant of Ras (L61-ras) resulted in high levels of cell surface GLUT4 even in the absence of insulin that were comparable to levels seen in control cells treated with a maximally stimulating dose of insulin.	PI3K and Ras	Stimulation (Both)	PI 3-kinase is essential for insulin-stimulated GLUT4 recruitment (Wortmannin block abolishes the effect).<br>Activated Ras can independently induce GLUT4 translocation to the plasma membrane to levels comparable to maximal insulin stimulation, and this Ras-mediated pathway operates independently of PI 3-kinase.	Signaling	Unclear risk (Not re	Low risk	Unclear risk (Not re	Unclear risk (Not re	Unclear risk (Not re	Unclear risk (Not re	Unclear risk (Not re	Unclear risk (Not re	Low risk	Unclear risk (Not re	Unclear risk (Not re	Low risk	Unclear risk (Not rep	Unclear risk (No	Low risk	
A.625	10.1038/cr.2009.68	Xiangyang Xie, 2009	China	To identify novel interaction partners of AS160 and investigate their roles in insulin-stimulated GLUT4 translocation.	3T3-L1 adipocytes.	Knockdown: siRNA (siRUVBL2) to deplete endogenous RUVBL2.<br>Rescue: Expression of human RUVBL2 (hRUVBL2 - resistant to mouse siRNA).<br>Overexpression: FLAG-RUVBL2 co-expression with AS160 for interaction studies.<br>Stimulation: Insulin treatment.	Translocation: Total Internal Reflection Fluorescence Microscopy (TIRFM) using GLUT4-EGFP.<br>Glucose Uptake: [³H] deoxyglucose uptake assay.<br>Phosphorylation: Western blotting for pAS160.<br>Identification: Mammalian Tandem Affinity Purification (TAP) combined with mass spectrometry.	Knockdown: 3 days post-transfection.<br>Translocation: 30 minutes post-insulin.<br>Differentiation: Expression monitored day 0 to day 10.	Genetic Comparison: Scrambled RNA (Control) vs. siRUVBL2 (Knockdown).<br>Rescue Comparison: Knockdown cells vs. hRUVBL2-rescued cells.<br>Model Comparison: Normal mice (C57BL/6J) vs. Diabetic mice (KKAy).	Ruvb like protein 2 (RUVBL2) is identified as a new AS160-binding protein using mammalian tandem affinity purification (TAP) combined with mass spectrometry. Depletion of RUVBL2 in adipocytes inhibits insulin-stimulated GLUT4 translocation and glucose uptake through reducing insulin-stimulated AS160 phosphorylation.	RUVBL2 and AS160	Stimulation	RUVBL2 is identified as a novel AS160-binding protein. It regulates GLUT4 translocation by modulating the insulin-stimulated phosphorylation of AS160. Reduced RUVBL2 levels lead to impaired AS160 phosphorylation, thereby inhibiting GLUT4 trafficking and glucose uptake.	Signaling	Unclear risk (Not re	Low risk	Unclear risk (Not re	Unclear risk (Not re	Unclear risk (Not re	Unclear risk (Not re	Unclear risk (Not re	Unclear risk (Not re	Low risk	Unclear risk (Not re	Unclear risk (Not re	Low risk	Unclear risk (Not rep	Unclear risk (No	Low risk	
A.631	10.1016/j.bcp.2014.08.033	Ragadeepthi Tunduguru, 2014	USA	To verify the hypothesis that PAK1 signaling is required for insulin-stimulated GLUT4 vesicle translocation to the plasma membrane by contributing to actin remodeling in skeletal muscle cells.	L6 skeletal muscle cells.	Chemical Inhibition: Use of IPA3 (selective allosteric inhibitor for group I PAKs).<br>Gene Expression: Transfection of PAK1 auto-inhibitory domain (PID) to create a dominant-negative effect.<br>Knockdown: siRNA-mediated depletion of PAK2 (siPAK2).<br>Stimulation: Insulin.	GLUT4 Translocation: Cell surface GLUT4myc detection using colorimetric/fluorescence assay.<br>Glucose Uptake: Radiolabeled 2-Deoxyglucose uptake measurement.<br>Actin Remodeling: Live-cell imaging using LifeAct-GFP biosensor.<br>Protein Analysis: Immunoblotting (Western blot).	Pre-incubation: IPA3 for 40–60 minutes.<br>Stimulation: Insulin for 10–20 minutes.	Chemical Comparison: Vehicle/DMSO vs. IPA3-treated group.<br>State Comparison: Basal vs. Insulin-stimulated.<br>Genetic Comparison: PID-L107F (non-inhibitory mutant) vs. PID expressing group.<br>Knockdown Comparison: Control siRNA (siCon) vs. PAK2 siRNA (siPAK2).	- Insulin-stimulated PAK1 phosphorylation is essential for GLUT4 vesicle transport and glucose uptake into skeletal muscle cells.<br>- PAK1 signaling is necessary for insulin-induced F-actin remodeling in skeletal muscle fibroblasts.	PAK1	Stimulation	PAK1 operates within the PI3K-Rac1 signaling branch (independent of Akt) and is responsible for remodeling the actin cytoskeleton to facilitate the transport of GLUT4 vesicles to the plasma membrane.	Transposition										Unclear risk (Not re	Unclear risk (Not re	Low risk	Unclear risk (Not rep	Unclear risk (No	Low risk	
A.635	10.1371/journal.pone.0043662	Jennine M. Dawicki-McKenna, 2012	USA	To investigate the spatial and kinetic involvement of microtubules in relation to GLUT4 vesicle trafficking in 3T3-L1 adipocytes.	3T3-L1 adipocytes.	Stimulation: Insulin (100 nM).<br>Disruption: Microtubule disruption with Nocodazole (10 μM or 2 μM); Actin disruption with Cytochalasin D or Latrunculin B.<br>Expression: HA-GLUT4-eGFP, mCherry-tubulin, 3xGFP-EMTB (microtubule marker), and IRAP-pHluorin (fusion marker).	TIRF Microscopy: Observation of events at or near the plasma membrane.<br>Parallax Microscopy: 3D tracking of microtubules near the membrane.<br>Dual-view Imaging: Simultaneous dual-wavelength imaging to correlate microtubule position with GLUT4 fusion events.<br>Quantitative Image Analysis: Analysis of microtubule curvature and density.	Starvation: 2–12 hours serum-starved.<br>Disruption: Nocodazole treatment for at least 20 minutes prior to insulin.	Treatment Comparison: Untreated (0 μM) vs. Nocodazole treated (10 μM).<br>State Comparison: Basal vs. Insulin-stimulated.<br>Spatial Comparison: Actual fusion sites vs. Random locations.	- Increased microtubule density in response to insulin stimulation in 3T3-L1 adipocytes<br>- Insulin stimulation induces microtubule curvature<br>- The fusion event preferentially occurs near microtubules	Microtubules	Stimulation	Insulin generates forces on microtubules, causing them to curve and be pulled close to the cell cortex. The primary role of microtubules at the plasma membrane in 3T3-L1 adipocytes is not for long-distance transport immediately prior to fusion, but to provide spatial information determining fusion sites. GLUT4 vesicles rarely travel long distances along microtubules just before fusion, but fusion events occur in close proximity to microtubules.	Fusion & Docking										Unclear risk (Not re	Unclear risk (Not re	Low risk	Unclear risk (Not rep	Unclear risk (No	Low risk	

A.637	10.1091/mbc.01-10-0490	Makoto Kanzaki, 2002	USA	To investigate the potential role of TC10 in regulating actin dynamics in adipocytes.	3T3-L1 adipocytes.	Genetic: Nuclear microinjection or transfection of DNA constructs: Constitutively active TC10 (TC10/Q75L), N-terminal deletion mutant (ΔN-TC10/Q75L), Dominant-interfering mutant (TC10/T31N). Chemical: Treatment with Latrunculin B and Clostridium difficile toxin B.	Real-time laser scanning confocal microscopy, single-cell nuclear microinjection, actin-based motility assay in Xenopus extracts, and co-immunoprecipitation.	Recovery: 24 hours post-injection/transfection. Transport Assay: Measured at 0, 10, 20, 30, 60, and 120 minutes after temperature shift to 32°C.	Injected cells vs. Surrounding non-injected cells.	TC10 can differentiate between the two types of fibroblast actin in adipocytes depending on their distinct functional domains and subunit distribution.	TC10 and Rac	Stimulation	TC10 regulates actin via two distinct mechanisms: (1) Stimulation of perinuclear actin polymerization, dependent on N-WASP and direct interaction with the COPI coat protein (via a C-terminal dilysine motif). (2) Disruption of cortical actin, dependent on the unique 16-amino acid N-terminal extension of TC10.	Signaling and Transposition	Unclear risk (Not r	Low risk	Unclear risk (Not r	Unclear risk (Not r	Unclear risk (Not r	Unclear risk (Not r	Unclear risk (Not r	Unclear risk (Not r	Low risk						
A.640	10.1074/jbc.M706873200	Yiqun Bao, 2008	Singapore	To identify novel proteins interacting with the Exo70 subunit of the exocyst complex to support GLUT4 vesicle tethering and docking, and to study the role of Snapin in regulating GLUT4 trafficking.	Mouse 3T3-L1 adipocytes.	Knockdown: siRNA-mediated depletion of Snapin. Overexpression: Lentiviral or transfection-mediated expression of Snapin and its mutants.	Co-immunoprecipitation (Co-IP), Western blot, Confocal microscopy (localization/colocalization), Glucose uptake assay, Subcellular fractionation.	Stimulation: Insulin for 20 minutes. Glucose Uptake: 72 hours post-siRNA. Interaction: 24 hours post-transfection/infection.	Genetic Comparison: Control siRNA vs. Snapin siRNA; GFP expression (Control) vs. Snapin expression. State Comparison: Basal vs. Insulin-stimulated.	Reducing snapin levels in fat cells using RNA interference inhibits insulin-stimulated glucose uptake --> Snapin interacts with exocysts and plays a regulatory role in the transport of GLUT4 cytoplasmic particles.	Snapin and Exo70	Stimulation	Snapin interacts directly with the Exo70 subunit of the exocyst complex via the N-terminal coiled-coil domain of Exo70 and the C-terminal helical domain of Snapin. Interestingly, Exo70 and the t-SNARE SNAP23 compete for the binding site on Snapin.	Tethering, Docking and Fusion										Unclear risk (Not r	Unclear risk (Not r	Low risk	Unclear risk (Not rep	Unclear risk (No	Unclear risk (Not reported)
A.646	10.1096/fj.05-4661fje	Katia Fecchi, 2006	USA	To investigate the hypothesis that flotillin-1 and caveolin-3 coordinate insulin-stimulated signaling cascades leading to GLUT4 translocation to the plasma membrane in skeletal muscle cells.	Conditionally immortalized skeletal muscle cell lines (normal endogenous caveolin-3 or caveolin-3 deficient).	Stimulation: Insulin (160 nM). Inhibition: Wortmannin (PI3K inhibitor), PKCζ pseudosubstrate inhibitor. Genetic: Use of caveolin-3 deficient cells; siRNA knockdown of flotillin-1.	Western blot (Immunoblot), Detergent-resistant membrane (DRM) fractionation via sucrose density gradient, Immunofluorescence and Confocal microscopy, Co-immunoprecipitation (Co-IP), Radiolabeled 2-deoxy-glucose uptake assay.	Insulin Treatment: 2, 5, 10, 20 minutes. Glucose Uptake: 10 minutes (with insulin).	Cell Type Comparison: Control vs. Caveolin-3 deficient cells. State Comparison: Basal (-Ins) vs. Insulin stimulated (+Ins). Genetic Comparison: Control siRNA vs. Flotillin-1 siRNA.	- GLUT4 is localized in extranuclear storage sites containing flotillin-1 in skeletal muscle cells. Insulin stimulation within 10 minutes leads to the translocation of flotillin-1/GLUT4-containing domains to the plasma membrane via a PI3K and PKCζ-dependent pathway. - Caveolin-3 is required for insulin receptor-mediated activation of the PI3K-dependent pathway.	Flotillin-1 and Caveolin-3	Stimulation	GLUT4 translocation in skeletal muscle cells is regulated in two steps: (1) Initially, insulin activates receptors in caveolae (containing caveolin-3) to initiate the PI3K/Akt/PKCζ pathway, driving GLUT4/flotillin-1 vesicles away from the perinuclear region. (2) Subsequently, caveolin-3 moves to the cytoplasm to interact with GLUT4/flotillin-1 vesicles, effectively transferring the insulin receptor to flotillin-1 to activate the Cbl/C3G/TC10 pathway, completing docking and fusion.	**All**										Unclear risk (Not r	Unclear risk (Not r	Low risk	Unclear risk (Not rep	Unclear risk (No	Unclear risk (Not reported)
A.647	10.1091/mbc.E13-02-0103	L. Amanda Sadacca, 2013	USA	To determine the roles and coordination of RAB10 and RAB14 along with other factors (RalA, MUNC18C, CDP138) in regulating GLUT4 translocation steps in adipocytes.	3T3-L1 adipocytes.	Stimulation: Insulin. Knockdown: siRNA/shRNA for RAB10, RAB14, AS160, DENND4C, RalA, MUNC18C, CDP138. Overexpression: RAB10, RAB14, AS160 (WT and 4A mutant).	Quantitative Immunofluorescence, Western blot, TIRF microscopy, qPCR.	Measured after 30 minutes of insulin stimulation.	Genetic Comparison: Control cells vs. Knockdown/Overexpression cells. State Comparison: Basal vs. Insulin.	- RAB14 acts before RAB10 in the sorting of GLUT4 into specialized transport vesicles that deliver GLUT4 to the plasma membrane. - RAB10 and its GTPase-activated protein (GAP), AS160, form the key signaling module under insulin receptor activation, regulating the accumulation of GLUT4 transport vesicles at the plasma membrane.	RAB10 and RAB14	Stimulation	RAB14 and RAB10 operate sequentially but at independent steps: RAB14 regulates the upstream sorting of GLUT4 into GSVs. RAB10 (controlled by the GAP AS160) regulates the accumulation/docking of GSV vesicles at the plasma membrane	Transportaion and Docking										Unclear risk (Not r	Unclear risk (Not r	Low risk	Unclear risk (Not rep	Unclear risk (No	Low risk
A.654	10.1042/BJ20061798	William G. Roach, 2007	USA	To investigate the GAP domain activity of Tbc1d1 and its effect on GLUT4 translocation in 3T3-L1 adipocytes.	3T3-L1 adipocytes.	Transfection: Overexpression of Wild-type Tbc1d1 (WT) and mutants: GAP-inactive (R854K), phosphorylation-deficient (T596A). Stimulation: Insulin (160 nM or 1 μM). Chemical Inhibition: Wortmannin (PI3K inhibitor).	GAP Assay: Measurement of GTPase activity of the Tbc1d1 GAP domain towards a panel of Rab proteins. GLUT4 Translocation: Single-cell fluorescence microscopy using HA-GLUT4-GFP reporter to measure surface (Cy3) to total (GFP) signal ratio. Phosphorylation: Immunoprecipitation and Immunoblotting with anti-PAS (phospho-Akt substrate) antibody to detect Tbc1d1 phosphorylation.	GAP Assay: 15 minutes (or 6–10 minutes for specific Rabs). Translocation: 24 hours post-transfection; 30 minutes insulin stimulation. Phosphorylation: 10 minutes insulin stimulation.	Assay Comparison: Rab alone vs. Rab + GAP domain vs. Rab + GAP mutant (R854K). Cell Comparison: Empty vector (V) vs. WT Tbc1d1 vs. Mutants (TA, RK, TA/RK, RW, RW/RK). State Comparison: Basal vs. Insulin-stimulated. Inhibitor Comparison: Wortmannin-treated vs. Insulin-treated.	- In addition to AS160, the animal genome also encodes a closely related protein called Tbc1d1. - Ectopic expression of Tbc1d1 significantly inhibits GLUT4 transport. - These results suggest that Tbc1d1 may regulate GLUT4 transport through its GAP activity.	Tbc1d1	Inhibition	Tbc1d1 functions as a Rab-GAP (similar to AS160) that negatively regulates GLUT4 translocation. It maintains Rab proteins in an inactive GDP-bound state, thereby preventing vesicle trafficking. Its inhibitory activity is modulated by phosphorylation (e.g., by Akt/AMPK), which presumably suppresses its GAP function to allow translocation.	Signaling										Unclear risk (Not r	Unclear risk (Not r	Low risk	Unclear risk (Not rep	Unclear risk (No	Unclear risk (Not reported)
A.660	10.1074/jbc.273.30.18784	Shane Rea, 1998	Australia (Australia) and USA (USA)	To identify the cognate t-SNAREs for v-SNARE VAMP2 in mouse 3T3-L1 adipocytes and demonstrate their role in insulin-stimulated GLUT4 translocation.	Mouse 3T3-L1 adipocytes.	Microinjection: Anti-Syndet antibodies. Peptide Treatment: Incubation of permeabilized cells with a synthetic peptide containing the C-terminal 24 amino acids of Syndet. Stimulation: Insulin.	Interaction: Far Western blotting (to detect VAMP2-binding proteins) and Surface Plasmon Resonance (SPR). Purification: Anion exchange chromatography. Function: GLUT4/GLUT1 translocation assays.	Translocation: Measured after 15 minutes of insulin stimulation (following peptide/antibody treatment). Interaction: Real-time SPR measurement; stability tested after 48 hours.	Injection Control: Mock-injected or irrelevant IgG-injected. Peptide Control: Randomized control peptide. State Comparison: Basal vs. Insulin-stimulated.	Identification of two VAMP2-binding proteins in the plasma membrane of 3T3-L1 adipocytes: Syndet (mSNAP-23) and Syntaxin-4 Functional role of Syndet in GLUT4 transport Implantation of GLUT1 stimulated by insulin remains unchanged in the presence of Syndet peptide	Syndet (mSNAP-23) and Syntaxin-4	Inhibition (of GLUT4 by interfering with Syndet)	Syndet (mouse homolog of SNAP-23), Syntaxin-4, and VAMP2 form a stable ternary SNARE complex. Interfering with Syndet function (via antibody or competitive peptide) specifically inhibits insulin-stimulated GLUT4 translocation without affecting basal GLUT1 trafficking. This proves that the specific assembly of this ternary complex is critical for the docking/fusion of GLUT4-containing vesicles but not for constitutive trafficking.	Docking and Fusion										Unclear risk (Not r	Unclear risk (Not r	Low risk	Unclear risk (Not rep	Unclear risk (No	Unclear risk (Not reported)
A.661	10.1074/jbc.M113.465450	Haijia Yu, 2013	USA	To determine the molecular mechanism of Synip protein in regulating SNARE-dependent vesicle fusion during GLUT4 exocytosis.	In vitro reconstituted artificial membrane systems (Proteoliposomes and Nanodiscs) containing GLUT4 exocytosis SNAREs (Syntaxin-4, SNAP-23, VAMP2).	Protein Addition: Purified recombinant Synip protein added to the fusion reaction mixture.	Lipid Mixing: FRET-based lipid mixing assay. Content Mixing: Nanodisc-liposome content mixing assay using calcein dye. Interaction: Liposome co-flotation assay and Liposome docking assay.		Control: Basal SNARE-only reaction. Negative Control: Addition of VAMP2 cytoplasmic domain (VAMP2 CD) to block SNARE complex formation. Specificity Control: Lysosomal SNAREs (Syntaxin-7/8, Vti1b, VAMP8) and Yeast exocytosis SNAREs (Sso1p, Sec9p, Snc2p).	- Synip inhibits the assembly of the three-component SNARE complex, but does not affect the formation of the t-SNARE complex. - Synip inhibits membrane coagulation via SNARE. - The inhibitory function of Synip predominates over the stimulatory activity of Munc18c—	Synip	Inhibition	Synip operates via a unique selective inhibitory mechanism: It binds specifically to the pre-formed t-SNARE complex (Syntaxin-4 and SNAP-23) on the target membrane, thereby preventing the initiation of ternary SNARE complex assembly (blocking the incorporation of the v-SNARE VAMP2). This arrest of fusion occurs at the docking/priming stage.	Docking and Fusion										Unclear risk (Not r	Unclear risk (Not r	Low risk	Unclear risk (Not rep	Unclear risk (No	Unclear risk (Not reported)
A.665	10.1016/s1097-2765(01)80007-1	Jing Min, 1999	USA	To isolate and characterize binding protein(s) capable of interacting with and regulating Syntaxin 4 in insulin-responsive tissues.	Differentiated 3T3-L1 adipocytes, CHO/IR cells, HEK293T cells, and mouse tissues.	Stimulation: Insulin (100 nM). Expression/Injection: Full-length Synip (WT), N-terminal domain (NT), C-terminal domain (CT).	Distribution: Northern blot. Interaction: Co-immunoprecipitation and GST pulldown. Function: 2-deoxyglucose transport assay. Localization: Confocal immunofluorescence microscopy on plasma membrane sheets.	Stimulation: 15–20 minutes prior to lysis/measurement.	State Comparison: Basal vs. Insulin. Control: Empty vector (pcDNA3) or GST only. Specificity: Comparison with other Syntaxins (1A, 1B, 2, 3). Mutant Comparison: WT vs. NT/CT mutants.	- Introduction to Synip protein and its relationship with Syntaxin 4 - Insulin disrupts the interaction between Synip and Syntaxin 4, thereby enabling Syntaxin 4 to bind to VAMP2 and bring GLUT4 to the surface.	Synip (Syntaxin 4-interacting protein)	Inhibition (of Syntaxin 4)	Under basal conditions, Synip binds specifically to Syntaxin 4, and this interaction is mutually exclusive with VAMP2 (preventing VAMP2 binding). The novel regulatory mechanism discovered is that insulin stimulation reduces the binding affinity between Synip and Syntaxin 4, causing the dissociation of the complex. This "releases" Syntaxin 4, enabling it to bind VAMP2 and facilitate GLUT4 translocation.	Docking	Unclear risk (Not r	Low risk	Unclear risk (Not r	Unclear risk (Not r	Unclear risk (Not r	Unclear risk (Not r	Unclear risk (Not r	Unclear risk (Not r	Low risk	Unclear risk (Not r	Unclear risk (Not r	Low risk	Unclear risk (Not rep	Unclear risk (No	Unclear risk (Not reported)
A.668	10.1091/mbc.7.7.1075	Volchuk A, 1996	Canada	To determine the expression, subcellular distribution, insulin regulation of Syntaxin isoforms in 3T3-L1 adipocytes, and the role of Syntaxin 4 in insulin-dependent glucose transport.	Differentiated 3T3-L1 adipocytes.	Stimulation: Insulin (100 nM). Treatment: Incubation of permeabilized cells with anti-syntaxin 4 antibody (aSy4).	Expression: Northern Blot, Immunoblotting. Distribution: Subcellular Fractionation. Interaction: Immunoisolation of GLUT4 vesicles. Function: Glucose Transport Assay in Streptolysin O-permeabilized cells.	Stimulation: 20 minutes. Antibody Incubation: 15 minutes prior to insulin addition.	State Comparison: Basal vs. Insulin. Control: Irrelevant IgG or No antibody.	"- Syntaxin 4 in plasma membranes and/or secretory vesicles is involved in the anchoring and/or fusion of GLUT4 vesicles on the cell surface of 3T3-L1 adipocytes."	Syntaxin 4	Stimulation	Insulin induces a redistribution of Syntaxin 4: decreasing in the low-density microsome (LDM) fraction and increasing at the plasma membrane (PM). Interestingly, a portion of intracellular Syntaxin 4 co-localizes on GLUT4-containing vesicles, suggesting it may play a role in vesicle docking/fusion dynamics from both the vesicle and target membrane sides.	Docking/Fusion	Unclear risk (Not r	Low risk	Unclear risk (Not r	Unclear risk (Not r	Unclear risk (Not r	Unclear risk (Not r	Unclear risk (Not r	Unclear risk (Not r	Low risk	Unclear risk (Not r	Unclear risk (Not r	Low risk	Unclear risk (Not rep	Unclear risk (No	Unclear risk (Not reported)
A.669	10.2337/diabetes.53.9.2223	Spurlin BA, 2004	USA	To investigate the physiological impact of Syntaxin 4 (Syn4) protein surplus on insulin-stimulated GLUT4 translocation and whole-body glucose homeostasis in vivo.	Transgenic mice overexpressing Syntaxin 4 on a C57BL/6J background (both sexes, 4-6 months old).	Genetic: Overexpression of Syntaxin 4 protein (5-fold in skeletal muscle, 3-fold in adipose tissue) under the control of a tetracycline-repressible promoter.	Metabolism: Intraperitoneal glucose tolerance test (IPGTT); Hyperinsulinemic-euglycemic clamp with radioactive tracers. Protein Analysis: Immunoblotting (Western Blot); Subcellular fractionation.	Fasting: 16–18 hours overnight. Clamp: 2-hour duration. Glucose Tolerance: 0, 30, 60, 90, 120 minutes post-glucose injection.	Genotype Comparison: Transgenic mice vs. Wild-type littermates. Treatment Comparison: Transgenic mice with vs. without Tetracycline (to turn off the gene).	- Insulin transport stimulates GLUT4 transport vesicles from an intracellular compartment to the cell membrane in 3T3-L1 adipocytes, regulated via a mechanism dependent on syntaxin 4 (Syn4) and Munc18c. - Increased Syn4 leads to increased Munc18c protein, suggesting that Syn4 regulates Munc18c expression in vivo.	Syntaxin 4	Stimulation	Increasing Syn4 protein levels leads to a parallel increase in Munc18c protein in vivo (Syn4 regulates Munc18c expression). This presumably increases the number of functional Syn4-Munc18c fusion sites at the plasma membrane, allowing a greater number of GLUT4 vesicles to dock and fuse, thereby enhancing glucose uptake.	Fusion	Unclear risk (Not r	Low risk	Unclear risk (Not r	Unclear risk (Not r	Unclear risk (Not r	Unclear risk (Not r	Unclear risk (Not r	Unclear risk (Not r	Low risk						
A.670	10.1128/MCB.17.5.2425	Ann Louise Olson,1997	USA	To determine the functional roles of Syntaxin (t-SNAREs) and VAMP (v-SNAREs) protein isoforms in insulin-stimulated GLUT4 translocation in adipocytes.	Differentiated 3T3-L1 adipocytes and rat epididymal fat pads (for mRNA).	Stimulation: Insulin (100 nM). Overexpression: Recombinant vaccinia virus infection expressing cytoplasmic domains of Syntaxin 4, Syntaxin 3, VAMP2, and VAMP3/cellubrevin. Microinjection: Single-cell injection of GST-fusion proteins (Syntaxin 4 and 3). Mutation: Syntaxin 4 deletion mutant lacking the VAMP-binding domain.	Translocation: Plasma membrane sheet assay with immunoblotting; Immunofluorescence and Confocal microscopy. Gene Expression: Northern blotting. Interaction: Immunoprecipitation (Co-IP)	Infection: 4 hours prior to experiment. Microinjection: 2 hours prior to experiment. Stimulation: 15 minutes.	Control: Uninfected cells; Vaccinia virus expressing LacZ. Specificity: Syntaxin 3 expression. Mechanism Control: Syntaxin 4 deletion mutant expression. State Comparison: Basal vs. Insulin.	Syntaxin 4, VAMP2, and/or VAMP3/cellubrevin are functional SNAP receptors on target membranes and transport vesicles for insulin-stimulated GLUT4 transport. Expression of the cytoplasmic domain of syntaxin 4 inhibits insulin-stimulated GLUT4 transport to the plasma membrane.	Syntaxin 4	Stimulation	Insulin stimulates the formation of a physical complex between Syntaxin 4 and GLUT4-containing vesicles. This interaction relies on the VAMP-binding domain of Syntaxin 4. Syntaxin 4 acts as the specific t-SNARE on the plasma membrane, pairing with v-SNAREs (VAMP2/3) on the GLUT4 vesicles to execute membrane fusion.	Fusion										Unclear risk (Not reported)	Unclear risk (Not reported)	Low risk	Unclear risk (Not reported)	Unclear risk (Not reported)	Unclear risk (Not reported)
A.672	10.1371/journal.pone.0042782	Saito T, 2012	Japan	To determine the binding capability of Synip to PIP3 via its WW domain and the role of this interaction in Synip localization at the plasma membrane and GLUT4 translocation.	Differentiated 3T3-L1 adipocytes and CHO/IR cells.	Stimulation: Insulin (100 nM). Genetic: Electroporation of Synip variants: Wild-type (WT), WW domain deletion (ΔWW), PDZ domain deletion (ΔPDZ), Coiled-coil domain deletion (ΔCC). Chemical: PI3K inhibitor (LY294002).	Interaction: Lipid-protein binding assay; GST pull-down assay. Translocation: Colorimetric Glut4 translocation assay (Myc-tagged). Localization: Subcellular fractionation & Immunoblotting.	Stimulation: 15 minutes (or 10 minutes for translocation). Recovery: 2 hours post-insulin removal. Post-electroporation: 24 or 48 hours.	Genetic Comparison: Synip WT vs. Mutants (ΔWW, ΔPDZ, ΔCC) vs. Empty vector. State Comparison: Basal vs. Insulin. Inhibitor Comparison: Untreated vs. LY294002.	Synip binds specifically to PIP3 via the WW domain: Synip is capable of binding to phosphatidylinositol (3,4,5) triphosphate (PIP3), but not to PIP or PIP2. The WW domain plays a crucial role in the localization of Synip at the plasma membrane: Upon insulin stimulation, Synip detaches from Syntaxin 4. However, Synip remains anchored at the plasma membrane through the interaction between the WW domain and PIP3. Overexpression of the Synip WW variant (lacking the PIP3 binding domain) in 3T3-L1 adipocytes reduces basal plasma membrane Glut4 levels and inhibits insulin-stimulated Glut4 translocation. Binding to PIP3 helps Synip maintain its proximity to Syntaxin 4 even after detaching, allowing it to quickly re-bind to Syntaxin 4 to prevent further Glut4 fusion upon insulin signaling. Stop.	Synip (Syntaxin 4 interacting protein)	Inhibition	Synip binds directly to PIP3 via its WW domain. Under insulin stimulation, Synip dissociates from Syntaxin 4 (allowing fusion) but remains anchored to the plasma membrane via this PIP3 interaction. This mechanism keeps Synip in close proximity to Syntaxin 4, enabling rapid re-association and inhibition of fusion as soon as the insulin signal terminates (and PIP3 levels drop).	Docking,Fusion										Unclear risk (Not reported)	Unclear risk (Not reported)	Low risk	Unclear risk (Not reported)	Unclear risk (Not reported)	Low risk
A.673	10.1074/jbc.273.39.25480	Frevert EU, 1998	USA	To determine if the direct interaction of Phosphoinositide 3-kinase (PI3K) with GLUT4 vesicles is critical for insulin's effect on GLUT4 translocation, by targeting constitutively active PI3K directly to these vesicles.	Differentiated 3T3-L1 adipocytes.	Genetic: Adenovirus-mediated co-expression of PI3K catalytic subunit p110a (p110) with fusion proteins targeting it to GLUT4 (iSH2-G4_c or G4_n-iSH2-G4_c) or untargeted (iSH2). Stimulation: Insulin (100 nM or 500 nM).	Enzyme Activity: PI3K Assay in cellular fractions. Function: Glucose Transport Assay; DNA Synthesis Assay ([³H]thymidine). Localization: Subcellular Fractionation & Western Blotting; Immunoadsorption of GLUT4 vesicles.	Transduction: 24 hours prior. Glucose Transport: 10 minutes (after 30 min insulin). DNA Synthesis: 17 hours. Fractionation: 15 minutes post-insulin.	Control: No virus; Beta-gal virus. Targeting Comparison: Untargeted PI3K (iSH2/p110) vs. GLUT4-targeted PI3K.	Direct interaction of phosphoinositide 3-kinase (PI3K) with GLUT4-containing vesicles is not necessary or sufficient to activate GLUT4 translocation and stimulate glucose transport.	PI3K	Stimulation	The study demonstrates that increasing PI3K activity specifically on the GLUT4 vesicle is not required or not sufficient to trigger GLUT4 translocation and glucose transport. This challenges the hypothesis that direct PI3K activation on the vesicle is the primary driver of translocation, suggesting PI3K must act at a different subcellular location (e.g., cytoskeleton or specific membrane subfraction) to effect change.	Signaling										Unclear risk (Not reported)	Unclear risk (Not reported)	Low risk	Unclear risk (Not reported)	Unclear risk (Not reported)	Low risk
A.675	10.1111/j.1600-0854.2012.01397.x	Davey JR, 2012	Australia	To identify RabGAP proteins regulating GLUT4 trafficking and link them to their corresponding Rabs, specifically clarifying the molecular role of TBC1D13 and Rab35 in the GLUT4 translocation pathway.	3T3-L1 adipocytes, HeLa cells, HEK293 cells.	Overexpression: TBC/RabGAP proteins (TBC1D1, TBC1D4, TBC1D11, TBC1D13, TBC1D15) via electroporation. Mutant Expression: TBC1D13 Wildtype (WT) vs. Catalytically inactive (RA). Active Rabs: Rab35 QL, Rab10 QL. Knockdown: siRNA for TBC1D13 and Rab35. Stimulation: Insulin (100 nM).	Translocation: HA-GLUT4 reporter assay. Function: Glucose transport assay. Interaction: Yeast two-hybrid screen; In vivo GAP assay (Rab effector pull-down with RBD35). Enzyme Activity: In vitro GAP assay.	Translocation: 20 minutes post-insulin. Post-electroporation: 48–72 hours.	Genetic Comparison: TagRFP (Control) vs. TBC1D13 (and others) WT vs. RA mutant. State Comparison: Basal vs. Insulin. Interaction Comparison: TBC1D13 alone vs. TBC1D13 + Rab35 QL (or Rab10 QL).	TBC1D13 is a RabGAP specific to Rab35 and plays a crucial role in regulating insulin-stimulated GLUT4 transport in fat cells.	TBC1D13 and Rab35	Stimulation	TBC1D13 acts as a specific GAP for Rab35 (promoting GTP hydrolysis to inactivate it) and not for Rab10 (despite interacting with Rab10). Rab35 plays a critical role in insulin-stimulated GLUT4 translocation. TBC1D13 likely functions as a bridge in a Rab cascade, where it interacts with Rab10 but regulates the activity of Rab35 to control GLUT4 flow (specifically the fast recycling step from endosomes to the plasma membrane).	Translocation										Unclear risk (Not reported)	Unclear risk (Not reported)	Low risk	Unclear risk (Not reported)	Unclear risk (Not reported)	Low risk
A.677	10.2337/db18-0769	Kjøbsted R, 2019	Denmark	To determine whether TBC1D4 is required for the enhancement of muscle insulin sensitivity following AICAR stimulation or contraction.	Whole-body Tbc1d4 knockout mice (Whole-body D4KO) and muscle-specific Tbc1d4 knockout mice (Muscle-specific mD4KO), along with Wild-type (WT) littermates. Isolated Extensor Digitorum Longus (EDL) and Tibialis anterior muscles.	AMPK Stimulation: Incubation with AICAR (1 mmol/L) or in situ muscle contraction (electrical stimulation of the common peroneal nerve). Insulin Stimulation: Post-recovery (6 hours after AICAR or 3 hours after contraction), muscles were stimulated with submaximal (100 μU/mL) or maximal (10,000 μU/mL) insulin. Genetic: TBC1D4 gene knockout.	Glucose Uptake: 2-Deoxyglucose uptake assay using radiolabel ([³H]2-deoxyglucose). Protein Analysis: Western Blotting for phosphorylation and total levels of signaling proteins (AMPK, ACC, TBC1D1, TBC1D4, Akt). Glycogen: Muscle glycogen measurement.	Acute: Immediately after 50 minutes of AICAR or 10 minutes of contraction. Recovery (Sensitivity Phase): 6 hours post-AICAR or 3 hours post-contraction.	Genotype Comparison: Wild-type (WT) vs. TBC1D4 Knockout (D4KO/mD4KO). Condition Comparison: Resting/Control vs. Prior AICAR/Prior contraction. Stimulus Comparison: Basal (No Insulin) vs. Insulin (Submaximal/Maximal).	TBC1D4 is essential for enhancing insulin sensitivity in skeletal muscle after muscle activation by AICAR or muscle contraction (simulating exercise). Although AMPK (cell energy sensor) signaling remains normally activated after muscle contraction or AICAR administration in both WT and TBC1D4-deficient mice, the insulin-stimulated increase in glucose uptake (representation of increased insulin sensitivity) occurs only in normal mice, while it is completely absent in TBC1D4-deficient mice.	Phosphorylated TBC1D4 (AS160)	Stimulation	The study provides genetic evidence confirming that the AMPK-TBC1D4 signaling axis is obligatory for the exercise-induced enhancement of muscle insulin sensitivity. Specifically, prior activation of AMPK leads to enhanced phosphorylation of TBC1D4 (particularly at Ser711 and Thr649) during subsequent insulin stimulation, thereby promoting glucose uptake. Without TBC1D4, the muscle's "memory" of prior exercise to sensitize insulin response is completely lost.	Signaling										Unclear risk (Not reported)	Unclear risk (Not reported)	Low risk	Unclear risk (Not reported)	Unclear risk (Not reported)	Low risk
A.679	10.1210/en.2006-1167	Louise Chang, 2007	USA	To characterize endogenous TC10 protein and determine if it is essential for insulin action specifically glucose transport) in insulin-responsive 3T3L1 adipocytes.	Differentiated 3T3L1 adipocytes.	Stimulation: Insulin (100 nM). Knockdown: siRNA specific for TC10α and TC10β. Inhibition: PI3K inhibitor (Wortmannin 100 nM). Comparison: PDGF (10 ng/ml) for specificity	GTPase Activity: GST-PBD pull-down assay. Expression: Immunoblot analysis (Western blot) and Real-time quantitative PCR (qPCR). Function: 2-[U-¹⁴C]deoxyglucose (2-DG) uptake assay. Translocation: GLUT4 translocation assay using myc-GLUT4-eGFP and fluorescence/confocal microscopy.	TC10α Activation: 0, 2, 5, 10, 15 minutes post-insulin. Glucose Uptake: 30 minutes post-insulin. Translocation: 0, 3, 5, 10, 15, or 30 minutes post-insulin.	Genetic Control: Scrambled control (Sc) RNAi. State Comparison: Basal (0 min) vs. Insulin. Inhibitor Control: Without Wortmannin.	Endogenous TC10α isoform is activated by insulin and is a mandatory factor for GLUT4 translocation and glucose uptake. TC10α knockdown also prevents the translocation of effector CIP4.	TC10α	Stimulation	Endogenous TC10α (but not TC10β) is rapidly activated by insulin via a PI3K-independent pathway. This activation is required for the translocation of the effector CIP4 and GLUT4 to the plasma membrane to facilitate glucose uptake	Signaling										Unclear risk (Not reported)	Unclear risk (Not reported)	Low risk	Unclear risk (Not reported)	Unclear risk (Not reported)	Unclear risk (Not reported)
A.680	10.1093/emboj/cdg262	Shian-Huey Chiang, 2003	USA	To identify and characterize TCGAP (a novel multi-domain protein) as a potential effector of Rho family GTPases, and to determine its role in insulin-stimulated glucose transport.	Mouse 3T3-L1 adipocytes, Cos-1, HEK293T, and CHO/IR cells.	Stimulation: Insulin (100 nM) following serum starvation. Overexpression: TCGAP (full-length, N-term, C-term, Y124A, K1228A mutants). Co-expression: Mutant GTPases (RhoA V14, Rac1 V12, Cdc42 Q61L, TC10α Q67L, TC10β Q69L) and CAP mutants (CAPΔSH3, CAPΔSorbo).	Activity: In vitro GAP assay. Interaction: Immunoprecipitation and Immunoblotting. Localization: Immunofluorescence and Confocal microscopy. Binding: Protein-lipid overlay assay. Function: 2-deoxyglucose uptake assay.	TCGAP Translocation: 2, 5 minutes post-insulin. Glucose Uptake: 5 minutes post-insulin. GLUT4 Translocation: 30 minutes post-insulin. Recovery: 24h, 36h, 48h post-transfection.	Control: Myc-tagged vector, LacZ control vector, Non-specific IgG. State Comparison: Basal vs. Insulin. Genetic Comparison: Wild-type vs. Mutants (Y124A, K1228A).	TCGAP is a protein that interacts with Cdc42 and TC10 beta and translocates to the plasma membrane upon insulin stimulation. Overexpression of TCGAP inhibits insulin-stimulated glucose transport and GLUT4 translocation.	TCGAP (TC10/cdc42 GTPase activating protein)	Inhibition	TCGAP acts as a downstream effector of TC10β (and Cdc42), linking the CAP/Cbl/Crk signaling pathway to GLUT4 translocation. TCGAP contains a PX domain (binding PI(4,5)P2) and a proline-rich region (binding CrkII), both of which are required for its insulin-dependent translocation to the plasma membrane.	Signaling										Unclear risk (Not reported)	Unclear risk (Not reported)	Low risk	Unclear risk (Not reported)	Unclear risk (Not reported)	Unclear risk (Not reported)

A.681	10.2337/db1518471928	Yoko Shimoda, 2015	Japan	To demonstrate that Tctex1d2 is induced during 3T3-L1 adipocyte differentiation and negatively regulates insulin-stimulated GLUT4 translocation and glucose uptake by directly binding to syntaxin 4.	Mouse 3T3-L1 adipocytes.	Stimulation: Insulin (100 nM or 0.2 nM); GIP (500 nM).<br><br>Overexpression: Tctex1d2 via plasmid (pcDNA3.1-Tctex1d2); Doc2b overexpression.<br><br>Knockdown: Tctex1d2 RNAi.	Expression: Western Blot.<br><br>Translocation: EGFP-GLUT4 translocation assay (confocal and colorimetric).<br><br>Function: 2-deoxyglucose uptake assay.<br><br>Interaction: Immunoprecipitation and MBP fusion protein pull-down.	Differentiation: Days 0, 4, 8.<br><br>Translocation/Uptake: 30 minutes post-insulin.<br><br>Signaling: 5, 15, 30 minutes post-insulin.<br><br>Internalization: 30, 60, 120 minutes post-insulin removal.	Control: Empty vector (pcDNA3.1), Scrambled RNAi.<br><br>State Comparison: Untreated (Basal) vs. Insulin.<br><br>Stimulus Comparison: Without GIP vs. With GIP.	Overexpression of Tctex1d2 inhibits GLUT4 translocation and insulin-stimulated glucose uptake, while knockdown of Tctex1d2 increases these processes.<br>Tctex1d2 binds to Syntaxin 4 (t-SNARE at the membrane), and this binding is increased in the presence of insulin. Conversely, the binding of Doc2b (fusion-promoting factor) to Syntaxin 4 is decreased in the presence of insulin.<br>Tctex1d2 directly competes with Doc2b for binding sites on Syntaxin 4. When Tctex1d2 is overexpressed, it prevents Doc2b from binding to Syntaxin 4, thereby inhibiting GLUT4 vesicle fusion. GIP (Gastric inhibitory peptide) reverses this inhibitory effect by reducing Tctex1d2-Syntaxin 4 binding.	Tctex1d2 (Tctex1 domain containing 2)	Inhibition	Tctex1d2 is a novel Syntaxin 4 binding protein that acts as a negative regulator of GLUT4 translocation. Specifically, Tctex1d2 functionally competes with Doc2b for binding to Syntaxin 4 upon insulin stimulation, thereby preventing the formation of the fusion-competent complex. GIP reverses this inhibitory effect by reducing the Tctex1d2-Syntaxin 4 interaction.	Fusion										Unclear risk (Not reported)	Unclear risk (Not reported)	Low risk	Unclear risk (Not reported)	Unclear risk (Not reported)	Low risk
A.685	10.2337/db14-0337	Kenneth K.Y. Cheng, 2014	Hong Kong	To investigate the physiological role of APPL2 in regulating glucose metabolism and the molecular mechanism involving its interaction with TBC1D1 in skeletal muscle.	C2C12 and L6 myotubes, isolated skeletal muscles (EDL, soleus), and HEK293 cells.	Stimulation: Insulin (10 nM, 60 μU/mL, or 0.5 units/kg).<br><br>Overexpression: APPL1, APPL2, TBC1D1 (and mutants) via adenovirus.<br><br>Knockdown: RNAi for APPL1, APPL2, TBC1D1.<br><br>Inhibition: PI3K inhibitor (LY294002).	Function: 2-deoxy-[³H]-glucose uptake assay; GTT and ITT.<br><br>Translocation: Antibody-coupled densitometric GLUT4 assay (L6-Myc-GLUT4).<br><br>Interaction: Immunoprecipitation, Mass spectrometry.<br><br>Signaling: Immunoblotting.	Function: 20–30 minutes post-insulin.<br><br>Signaling: 5, 10, 15, 20, 30 minutes post-insulin.<br><br>Tolerance Tests: 90–120 minutes.	Control: Luciferase (Luci) overexpression; Scrambled RNAi.<br><br>Genotype: WT littermates vs. Transgenic mice.<br><br>State Comparison: Basal vs. Insulin.	APPL2 acts as a negative regulator of insulin-stimulated glucose transport by interacting with TBC1D1. This interaction prevents TBC1D1 phosphorylation at Thr596 (a necessary step to activate GLUT4 translocation).<br>AppL2 overexpression inhibits, while APPL2 knockdown enhances glucose uptake and insulin-stimulated GLUT4 translocation.<br>AppL2 overexpression reduces insulin-stimulated TBC1D1 phosphorylation at Thr596.<br>TBC1D1-S235A mutations (unable to bind to APPL2) eliminate the inhibitory effect of APPL2 on Thr596 phosphorylation and glucose uptake.	APPL2	Inhibition	APPL2 interacts directly with TBC1D1 via the BAR domain. This interaction is dependent on the insulin/Akt-induced phosphorylation of TBC1D1 at Ser235. The binding of APPL2 to TBC1D1 prevents the subsequent phosphorylation of TBC1D1 at Thr596 (a step required for GLUT4 translocation), thereby inhibiting glucose uptake. This identifies APPL2 as a negative regulator, contrasting with the positive role of APPL1.	Signaling										Unclear risk (Not reported)	Unclear risk (Not reported)	Low risk	Unclear risk (Not reported)	Unclear risk (Not reported)	Low risk
A.688	10.1074/jbc.M112.357699	Wenying Ren, 2012	USA	To investigate the regulatory effects of ClipR-59 protein on AS160 phosphorylation and subsequent glucose transport in adipocytes.	Mouse 3T3-L1 adipocytes and Human Embryonic Kidney (HEK293) cells.	Stimulation: Insulin (100 nM).<br><br>Overexpression: Wild-type ClipR-59.<br><br>Mutant Expression: ClipR-59 mutants (ΔANK - ankyrin repeats deletion, Δ2CAP - CAP-Gly domains deletion), AS160 mutant (4P-AS160), Akt mutant (E299K-Akt2).<br><br>Chemical: PI3K inhibitor (LY294002) and Akt inhibitor VIII.	Interaction: Co-immunoprecipitation (co-IP), GST Pulldown Assay.<br><br>Localization: Subcellular Fractionation (sucrose gradient), Cell Imaging (Immunostaining).<br><br>Signaling: Western Blotting (Immunoblotting).<br><br>Function: Glucose Uptake Assay (2-[³H] deoxyglucose).	Stimulation: 30 minutes.<br><br>Expression: 24, 36, or 48 hours post-transfection/transduction.	Control: GFP or Empty Vector.<br><br>State Comparison: Basal (no insulin) vs. Insulin.<br><br>Genetic Comparison: Wild-type ClipR-59 vs. Mutant (ΔANK-ClipR-59).	ClipR-59 interacts directly with AS160 via its ankyrin repeats, and this interaction is essential for promoting AS160 phosphorylation (by Akt) and GLUT4 translocation to the plasma membrane	ClipR-59	Stimulation	ClipR-59 acts as a scaffold protein for Akt and AS160. It interacts directly with AS160 via its ankyrin repeats and recruits activated Akt. By bringing Akt into proximity with AS160, ClipR-59 facilitates AS160 phosphorylation, thereby inhibiting its GAP activity and releasing GLUT4 for translocation. This interaction is negatively regulated by insulin signaling (AS160 phosphorylation reduces affinity).	Signaling										Unclear risk (Not reported)	Unclear risk (Not reported)	Low risk	Unclear risk (Not reported)	Unclear risk (Not reported)	Low risk
A.689	10.1038/cr.2012.52	Hui-Ling Guo, 2012	China	To identify molecular components and regulatory mechanisms of insulin-stimulated glucose uptake, specifically focusing on the role of the Axin/TNKS/KIF3A complex.	Differentiated 3T3-L1 adipocytes and HEK293T cells.	Stimulation: Insulin (200 nM).<br><br>Knockdown: siRNA/adenovirus for Axin, TNKS1/2, KIF3A.<br><br>Overexpression: Axin, TNKS, KIF3A.<br><br>Chemical: MG132 (proteasome inhibitor), Akt inhibitor VIII, XAV939 (TNKS PARsylase inhibitor).<br><br>In Vivo: Fasting and Re-feeding.	Interaction: IP, Co-IP, In vitro pull-down, Yeast two-hybrid screen.<br><br>Localization: Immunofluorescence staining / Confocal microscopy, Plasma membrane fractionation.<br><br>Function: Glucose uptake assay (2-deoxy-D-glucose), GTT & ITT.<br><br>Signaling: Western Blotting.	Stimulation: 30 minutes.<br><br>Knockdown: 48 hours post-infection.<br><br>Starvation: 4 hours.<br><br>Glucose Uptake: 10 minutes (after 20 min insulin).	State Comparison: Basal (- Ins) vs. Insulin.<br><br>Control: IgG control, Scrambled siRNA.<br><br>Genotype: Wild-type vs. Transgenic/Knockdown models.	Axin acts as a scaffold to directly bind both TNKS and KIF3A, forming a ternary complex. This complex co-localizes with GLUT4 at the trans-Golgi network (TGN). Upon insulin stimulation, Akt suppresses the PARsylase activity of TNKS. This reduces the PARsylation and subsequent ubiquitination/degradation of Axin and TNKS, thereby stabilizing the Axin/TNKS/KIF3A complex. Stabilization of this complex allows the motor protein KIF3A to transport GLUT4-containing vesicles along microtubules to the plasma membrane, promoting glucose uptake.	Axin, TNKS1/2 (Tankyrase), KIF3A	Stimulation	Axin scaffolds a ternary complex with TNKS and the motor protein KIF3A. Insulin (via Akt) stabilizes this complex by inhibiting TNKS-mediated auto-PARsylation and degradation. The stabilized complex enables KIF3A to drive the transport of GLUT4 vesicles from the TGN to the plasma membrane.	Translocation										Unclear risk (Not reported)	Unclear risk (Not reported)	Low risk	Unclear risk (Not reported)	Unclear risk (Not reported)	Low risk
A.697	10.1038/nature01533	Mayumi Inoue, 2003	USA	To determine the role of the exocyst complex (specifically the Exo70 component) and its interaction with the G-protein TC10 in the targeting and fusion of Glut4 vesicles to the plasma membrane under insulin stimulation.	3T3L1 adipocytes and 293T cells.	Stimulation: Insulin (100 nM).<br><br>Overexpression: Exo70 (WT, truncated mutants), TC10 (Constitutively active Q67L, Inactive T23N).<br><br>Method: Transfection and Electroporation.	Interaction: Yeast two-hybrid screen, IP, In vitro GST pull-down.<br><br>Localization: Immunofluorescence/Confocal microscopy.<br><br>Function: 2-deoxyglucose uptake assay, Glut4 translocation assay (Glut4-eGFP, HA-Glut4-GFP).<br><br>Signaling: Immunoblotting.	Exo70 Translocation: 2, 5, 8, 10 minutes post-insulin.<br><br>Function: 30 minutes post-insulin.	Control: Empty Vector.<br><br>State Comparison: Basal vs. Insulin.<br><br>Genetic Comparison: Inactive TC10 vs. Active TC10; WT Exo70 vs. Exo70-N mutant.	The exocyst complex (via Exo70) is essential for the targeting and tethering of GLUT4 vesicles at the plasma membrane in response to insulin. Active TC10 interacts directly with Exo70 and recruits it to the plasma membrane. Insulin stimulates Exo70 translocation via TC10. The Exo70-N mutant does not prevent GLUT4 translocation to the cell periphery (GFP ring visible) but completely prevents GLUT4 exposure on the cell surface (fusion block). This proves the exocyst is required for the final tethering/docking step.	TC10 and Exo70	Stimulation	Insulin activates TC10, which interacts directly with Exo70 in a GTP-dependent manner to recruit it to the plasma membrane. Exo70 then facilitates the assembly of the exocyst complex (including Sec6, Sec8). This complex serves as a critical tether for GLUT4 vesicles, ensuring they are positioned correctly for fusion.	Tethering										Unclear risk (Not reported)	Unclear risk (Not reported)	Unclear risk (Not reported)	Low risk	Unclear risk (Not reported)	Low risk
A.698	10.1152/ajpendo.00109.2019	Brent A. Fujimoto, 2019	USA	To investigate the impact of exocyst complex activity (modulated via inhibition or knockout) on GLUT4 trafficking and glucose uptake in response to insulin in skeletal muscle cells.	Rat L6 myoblasts and differentiated myotubes.	Stimulation: Insulin (100 nM).<br><br>Pharmacological Inhibition: Endosidin-2 (ES2 - 100 μM, exocyst inhibitor).<br><br>Genetic: CRISPR/Cas9 heterozygous knockout of Exoc5.	Protein Analysis: Western blotting, Subcellular fractionation.<br><br>Function: Glucose uptake assays (2-NBDG, 2DG), Seahorse technology.<br><br>Localization: Immunofluorescence, Proximity ligation amplification (PLA).	Stimulation: 30 minutes (concurrent with ES2 if used).	State Comparison: Basal vs. Insulin.<br><br>Treatment Comparison: DMSO control vs. ES2 treated.<br><br>Genetic Comparison: Wild-type vs. Exoc5 heterozygous knockout.	Under basal conditions, exocyst subunits (EXOC1, EXOC5, EXOC7) co-localize with GLUT4 in the perinuclear region; upon insulin stimulation, they traffic with GLUT4 to the plasma membrane and increase association with each other. The inhibitor Endosidin-2 (ES2) prevents exocyst assembly and recruitment, leading to impaired GLUT4 surface delivery and glucose uptake. Disrupted Exoc5 expression leads to reduced EXOC1 expression, impaired myotube differentiation, and significantly decreased insulin-stimulated GLUT4 surface delivery and glucose uptake.	Exocyst Complex (EXOC1, EXOC5, EXOC7)	Stimulation	In skeletal muscle, insulin triggers the holocomplex assembly of the exocyst and recruits it to the plasma membrane. This complex is essential for the docking of GLUT4 vesicles prior to fusion. Inhibition of assembly (via ES2) or loss of a subunit (Exoc5) disrupts this trafficking step.	Tethering										Unclear risk (Not reported)	Unclear risk (Not reported)	Low risk	Unclear risk (Not reported)	Unclear risk (Not reported)	Low risk
A.709	10.1002/1873-3468.13939	Nobuyuki Takenaka, 2020	Japan	To investigate the regulatory mechanisms of Rac1 in insulin signaling in adipocytes, specifically the role of the guanine nucleotide exchange factor (GEF) FLJ00068.	In vitro differentiated 3T3-L1 adipocytes and L1-GLUT4 cell line (3T3-L1 stably expressing myc7-GFP-GLUT4).	Stimulation: Insulin (100 nM for 30 min).<br><br>Genetic:<br><br>* Knockdown of FLJ00068 using siRNA (20 nM).<br><br>* Knockdown of Rac1 using shRNA via adenovirus vector.<br><br>* Overexpression of constitutively active FLJ00068 and Akt2.<br><br>Pharmacological: Rac1 inhibitor.	Expression: RT-PCR (mRNA levels).<br><br>Function/Signaling: Confocal immunofluorescence microscopy (GLUT4 reporter assay and Rac1 activation).<br><br>Protein Analysis: Immunoblot analysis (Western blot).<br><br>Localization: Subcellular fractionation.	30 minutes post-insulin stimulation.	Control: Negative control siRNA, untreated group (-), or empty vector group.<br><br>Experimental: Insulin-treated, Knockdown, or Overexpression groups.	FLJ00068 is a guanine nucleotide exchange factor (GEF) specific for Rac1 (a small GTPase important for GLUT4 translocation). Knockdown of FLJ00068 reduces insulin-induced Rac1 activation, thereby decreasing GLUT4 translocation and glucose uptake in adipocytes. Insulin stimulates Akt phosphorylation of FLJ00068 at the Ser-533 site. This phosphorylation increases the GEF activity of FLJ00068 against Rac1. Upon insulin signaling, FLJ00068 migrates to the plasma membrane to perform its function.	FLJ00068	Stimulation	The study identifies FLJ00068 as a specific Guanine Nucleotide Exchange Factor (GEF) acting downstream of Akt2 to activate Rac1, thereby promoting GLUT4 translocation to the plasma membrane in adipocytes. Insulin stimulates the phosphorylation of FLJ00068 at Ser-533 by Akt2, which increases its GEF activity and induces its translocation to the plasma membrane to execute its function.	Signaling										Unclear risk (Not reported)	Unclear risk (Not reported)	Low risk	Unclear risk (Not reported)	Unclear risk (Not reported)	Low risk
A.721	10.2337/db06-0900	Farah S.L. Thong, 2007	Canada	To test the hypothesis that AS160 serves as a molecular link between diverse signaling cascades (Akt, PKC, AMPK) converging to regulate GLUT4 translocation.	L6 GLUT4myc myoblasts and CHO-IR cells.	Stimulation: Insulin (100 nmol/l), PDGF (50 ng/ml), AICAR (2 mmol/l), DNP (0.5 mmol/l), Sucrose (0.45 mol/l), PMA (1 μmol/l), K+ depolarization (120 mmol/l K gluconate).<br><br>Genetic: Transfection of Wild-type AS160 (WT) and mutants: 4P-AS160 (phosphorylation-deficient), RK-AS160 (GAP-inactive), 4PRK-AS160, AAA-Akt (kinase-inactive), α2-AMPK.<br><br>Inhibition: LY294002 (PI3K), Bisindolylmaleimide - BIM (c/n PKC).Stimulation: Insulin (100 nmol/l), PDGF (50 ng/ml), AICAR (2 mmol/l), DNP (0.5 mmol/l), Sucrose (0.45 mol/l), PMA (1 μmol/l), K+ depolarization (120 mmol/l K gluconate).<br><br>Genetic: Transfection of Wild-type AS160 (WT) and mutants: 4P-AS160 (phosphorylation-deficient), RK-AS160 (GAP-inactive), 4PRK-AS160, AAA-Akt (kinase-inactive), α2-AMPK.<br><br>Inhibition: LY294002 (PI3K), Bisindolylmaleimide - BIM (c/n PKC).	Signaling: Immunoprecipitation, Immunoblotting (Western blot).<br><br>Translocation: Immunofluorescence confocal microscopy for surface GLUT4myc detection.	Stimulation: Insulin (10 min), PDGF (7 min), K+ (20 min), PMA (30 min), Sucrose (30 min), DNP (20 min), AICAR (40 min).<br><br>Transfection: 18–24 hours prior.	State: Basal (Unstimulated) vs. Stimulated.<br><br>Control: Untransfected/Empty vector.<br><br>Genetic: WT-AS160 vs. Mutants (4P, RK, 4PRK).	AS160 acts as a convergence point for signals from Akt, Protein Kinase C (PKC), and AMP-activated protein kinase (AMPK) to regulate GLUT4 transport.<br><br>The non-phosphorylating AS160 variant (4P-AS160) completely eliminates the ability to increase surface GLUT4 induced by insulin, PDGF, K+ depolarization, or AICAR.<br><br>PKC activators (such as PMA, K+ depolarization) increase AS160 phosphorylation, and this increase is inhibited by PKC inhibitors (bisindolylmaleimide - BIM).<br><br>Stimulation of AS160 phosphorylation by AICAR or DNP (AMPK activators) depends on the presence of the α2-AMPK subunit.<br><br>Although osmotic shock increases AS160 phosphorylation, the 4P-AS160 variant only partially inhibits it. This suggests that this pathway may utilize a different mechanism independent of AS160 or another phosphorylation site.	AS160 (TBC1D4)	Stimulation	AS160 functions as a signal integrator where inputs from AKT, PKC (conventional/novel), and AMPK (α2) converge. These kinases phosphorylate AS160 to inhibit its GAP activity, thereby releasing GLUT4 vesicles from intracellular retention and allowing translocation. However, GLUT4 translocation induced by hyperosmolarity operates via a distinct mechanism independent of AS160 phosphorylation.	Signaling										Unclear risk (Not reported)	Unclear risk (Not reported)	Low risk	Unclear risk (Not reported)	Unclear risk (Not reported)	Low risk
A.722	10.1128/MCB.00761-12	Shi-Xiong Tan, 2012.	Australia	To investigate novel functional characteristics of the second phosphotyrosine-binding (PTB) domain of AS160, specifically its phospholipid binding ability and role in targeting AS160 to the plasma membrane (PM) and regulating GLUT4 trafficking.	Differentiated 3T3-L1 adipocytes, HEK293E cells, CHO IR/IRS-1 cells.	Stimulation: Insulin (0.1 - 100 nM), 2-Deoxyglucose (metabolic stress).<br><br>Genetic: Overexpression of AS160 variants (WT, lipid-binding mutant KK215/216AA, PM-targeted Lyn-AS160, phosphorylation-deficient 4P, GAP-inactive R/A).<br><br>Inhibition: MK-2206 (Akt inhibitor).	Imaging: Live-cell TIRF microscopy.<br><br>Localization: Subcellular fractionation, Cationic silica isolation of PM.<br><br>Interaction: Protein-lipid overlay assay, Immunoprecipitation, Western blotting.<br><br>Function: 2-Deoxyglucose uptake assay, Quantitative GLUT4 translocation assay.	Insulin: 10–30 minutes.<br><br>Inhibitor: 15–30 minutes.	State: Basal vs. Insulin.<br><br>Genetic: EGFP/Vector (Control) vs. AS160 variants.<br><br>Chemical: DMSO vs. MK-2206.	AS160 contains a second phosphotyrosine-binding domain (PTB) (amino acids 190-365) capable of binding to membrane phospholipids (PI(3)P, PI(4)P, etc.) and not just phosphopeptides.<br><br>This lipid-binding domain (specifically the conserved lysines K215/K216) is responsible for transporting AS160 to the plasma membrane. The K215/216AA mutation causes loss of membrane binding.<br><br>The second PTB domain has two distinct binding sites: one lipid-binding site (for membrane localization) and one site that binds to the GLUT4 vesicle protein (like IRAP). The K215/216AA mutation prevents membrane binding but does not affect IRAP binding.<br><br>The GLUT4 translocation inhibitory function of AS160 is achieved through interaction with GLUT4-containing vesicles (via IRAP), not through interaction with the membrane itself. cell membrane.<br><br>When AS160 is artificially introduced to the cell membrane (Lyn-AS160), it increases the amount of surface GLUT4. This effect requires AS160 to be phosphorylated and bound to 14-3-3 (to inactivate GAP).	AS160 (TBC1D4)	Inhibition (on vesicles) / Stimulation (on membrane)	A novel atypical phospholipid-binding domain was discovered within the second PTB domain of AS160 (involving residues K215/K216), responsible for targeting AS160 to the plasma membrane. At the PM, AS160 is phosphorylated by Akt and binds 14-3-3, which inhibits its GAP activity and switches AS160 to a facilitative role, promoting GLUT4 vesicle docking/fusion. AS160 acts as a dual regulatory switch: inhibitory when bound to vesicles (unphosphorylated) via IRAP, and stimulatory when recruited to the PM (phosphorylated).	Signaling										Unclear risk (Not reported)	Unclear risk (Not reported)	Low risk	Unclear risk (Not reported)	Unclear risk (Not reported)	Low risk
A.727	10.1074/jbc.M704357200	Marco Falasca, 2007	UK	To determine the in vivo product of PI3K-C2α upon insulin activation, its activation mechanism, and its precise intracellular role in insulin signaling, specifically regarding GLUT4 translocation and glucose uptake.	L6 muscle cells.	Stimulation: Insulin (100 - 300 nM), PDGF.<br><br>Genetic: Knockdown of PI3K-C2α (shRNA). Overexpression of Myc-TC10 (active/dominant-negative), HA-GLUT4, EGFP-GLUT4, GFP-2xFYVE (PtdIns-3-P probe).	Lipid Analysis: HPLC for phosphoinositides.<br><br>Imaging: Confocal microscopy, TIRFM.<br><br>Protein Analysis: Western blotting, Subcellular fractionation, In vitro kinase assay.<br><br>Function: Glucose uptake assay (2-deoxy [3H]glucose).	1.5 to 20 minutes post-insulin.	Control: Scrambled shRNA.<br><br>State: Unstimulated vs. Insulin-stimulated.<br><br>Specificity: Insulin vs. PDGF.	Insulin activates PI3K-C2α via a small GTPase called TC10. Dominant-negative mutations in TC10 inhibit insulin-induced activation of PI3K-C2α.<br><br>Insulin stimulates PI3K-C2α to translocate from the intracellular space to lipid rafts on the plasma membrane to perform its function.<br><br>Knocking down PI3K-C2α significantly reduces GLUT4 translocation to the membrane and decreases glucose uptake under insulin's influence."	PI3K-C2α (Phosphoinositide 3-kinase class II alpha)	Stimulation	The study identifies PtdIns-3-P as the unique in vivo product of PI3K-C2α in the insulin signaling pathway (distinct from Class I PI3K which produces PIP3). PI3K-C2α is activated via translocation to plasma membrane lipid rafts, mediated by TC10. The PtdIns-3-P generated by PI3K-C2α is crucial for the initial step of GLUT4 trafficking (recruitment to the cell periphery/plasma membrane - Step 1), operating in parallel with the Class I PI3K/Akt pathway (required for fusion - Step 2).	SIgnaling										Unclear risk (Not reported)	Unclear risk (Not reported)	Low risk	Unclear risk (Not reported)	Unclear risk (Not reported)	Low risk
A.732	10.1074/jbc.M300664200	Jun Liu, 2003	USA	To clarify whether Cbl-b undergoes tyrosine phosphorylation and acts as an adaptor protein in the insulin pathway, and how c-Cbl and Cbl-b interact with the insulin receptor to regulate glucose transport.	Differentiated 3T3-L1 adipocytes and CHO-IR cells.	Stimulation: Insulin (100 nM).<br><br>Overexpression: c-Cbl (WT, mutants: δLZ, 1-700), Cbl-b (WT, mutants: G298E, Y665F/Y709F), APS (WT), CAP (WT, mutant δSH3C), GLUT4-EGFP.<br><br>Inhibition: PD098059 (MEK inhibitor), Wortmannin (PI3K inhibitor), PP2 (Src kinase inhibitor).	Interaction/Phosphorylation: Immunoprecipitation and Immunoblotting (Western blot).<br><br>Localization: Subcellular Fractionation (LDM vs PM).<br><br>Translocation: Fluorescence Microscopy and Confocal microscopy for Cbl and GLUT4.	Stimulation: 2, 5, 10, 15, 30 minutes post-insulin.<br><br>Expression: 30 hours post-electroporation.	State Comparison: Basal (-Insulin) vs. Insulin (+Insulin).<br><br>Genetic Comparison: Empty vector (Control) vs. Wild-type/Mutant protein expression.	Similar to c-Cbl, the Cbl-b protein in 3T3-L1 adipocytes undergoes rapid tyrosine phosphorylation when stimulated by insulin. The primary phosphorylation sites identified are Tyrosine 665 and 709.<br><br>The phosphorylation and translocation of Cbl-b depend on its interaction with the CAP protein. Mutations in the CAP binding region will prevent this process.<br><br>Cbl-b and c-Cbl have interchangeable functions. Inhibition of Cbl-b function (by N-terminal mutations) will prevent GLUT4 translocation and glucose uptake.	c-Cbl and Cbl-b	Stimulation	Cbl-b (like c-Cbl) participates in an insulin signaling pathway independent of PI3K/Akt. Both proteins require dimerization (via the leucine zipper region) to interact effectively with APS and be phosphorylated by the insulin receptor. The dimerized and phosphorylated form of Cbl recruits the Crk-C3G complex to lipid rafts via CAP, thereby activating TC10 and promoting GLUT4 translocation. Disrupting this interaction (e.g., via c-Cbl/1-700) inhibits GLUT4 translocation.	Signaling										Unclear risk (Not reported)	Unclear risk (Not reported)	Low risk	Low risk	Unclear risk (Not reported)	Unclear risk (Not reported)
A.733	10.1210/en.138.11.4941	Peter Vollenweider, 1997	USA	To evaluate the role of Rab4 in insulin-stimulated GLUT4 translocation and actin filament rearrangement in 3T3-L1 cells.	Differentiated 3T3-L1 adipocytes.	Stimulation: Insulin (17 nM or 100 ng/ml).<br><br>Microinjection: Cytoplasmic injection of GST-Rab4WT, GST-Rab4N121I (dominant negative), GST-Rab4Q67L (constitutively active), or anti-Rab4 antibody.<br><br>Inhibition: Wortmannin (PI3K inhibitor).	Microinjection: Single cell microinjection.<br><br>Detection: Indirect Immunofluorescence for GLUT4 and Actin (Rhodamine-phalloidin).<br><br>Quantification: Counting cells positive for continuous plasma membrane GLUT4 rims.	30 minutes post-insulin stimulation.	Control: Basal/Unstimulated; GST alone or Preimmune IgG injection.<br><br>Experimental: Rab4 mutants/antibody injection.	"Inhibition of Rab4 function (by antibody or the negative-dominant N121I mutation) reduces insulin-stimulated GLUT4 translocation by approximately 50%.<br><br>The continuously activated mutant form of Rab4 (Q67L) is capable of stimulating GLUT4 translocation to the membrane even in the absence of insulin.<br><br>Insulin causes actin filament rearrangement/membrane ruffling. Rab4 is directly involved in this process: inhibition of Rab4 prevents insulin-induced actin rearrangement, and activation of Rab4 itself causes actin rearrangement.<br><br>The study concludes that Rab4 is involved in regulating GLUT4 translocation through the reorganization of the cellular actin scaffold."	Rab4	Stimulation	The study establishes a functional link between Rab4 and cytoskeletal actin rearrangement during GLUT4 translocation. Specifically, Rab4 is not only involved in vesicle transport but is also required for insulin-stimulated membrane ruffling (actin rearrangement). The active (GTP-bound) form of Rab4 is necessary and sufficient for both of these processes.	Translocation										Unclear risk (Not reported)	Unclear risk (Not reported)	Low risk	Unclear risk (Not reported)	Unclear risk (Not reported)	Unclear risk (Not reported)
A.736	10.1242/jcs.114.2.445	Luke H. Chamberlain, 2001	UK	To identify and characterize regulators of GLUT4 translocation, specifically examining the expression, localization of cysteine-string protein (Csp) in adipocytes and its interaction with syntaxin 4.	Differentiated 3T3-L1 adipocytes and L6 skeletal muscle cells.	Stimulation: Insulin (1 μM for 15 min).<br><br>Chemical: Hydroxylamine (depalmitoylation), Triton X-100 extraction.<br><br>Overexpression: Csp expression.	Localization: Subcellular fractionation (sucrose/iodixanol gradients); Whole cell immunofluorescence.<br><br>Interaction: Western blotting, Plasma membrane lawn assay, Protein binding studies (GST/His-tag).	15 minutes post-insulin.	State Comparison: Basal vs. Insulin.<br><br>Cell Type: Fibroblast vs. Adipocyte.<br><br>Treatment: Tris (Control) vs. Hydroxylamine.	In 3T3-L1 adipocytes, cysteine chain protein (CSP) primarily binds to the plasma membrane rather than residing in intracellular vesicles as in neurons.<br><br>CSP is not located in the same site as GLUT4 or VAMP2, but in the same site as Syntaxin 4 on the plasma membrane. CSP interacts with Syntaxin 4 and SNAP-25 (t-SNAREs), but not with the v-SNARE VAMP2.<br><br>Overexpression of CSP significantly reduces the translocation of GLUT4 to the membrane upon insulin stimulation, suggesting a negative or competitive regulatory role.	Csp1 (Cysteine-string protein 1)	Inhibition (Regulatory/Negative)	The paper identifies Csp1 as a plasma membrane-resident protein in adipocytes (distinct from its synaptic vesicle location in neurons). Here, Csp1 interacts directly with Syntaxin 4. Given the slower kinetics of GLUT4 fusion compared to neurotransmission, plasma membrane Csp1 may act as a chaperone for Syntaxin 4 (modulating conformation) or participate in SNARE complex assembly/disassembly to regulate the fusion of GLUT4 vesicles with the plasma membrane.	Fusion										Unclear risk (Not reported)	Unclear risk (Not reported)	Low risk	Unclear risk (Not reported)	Unclear risk (Not reported)	Unclear risk (Not reported)
A.737	10.1016/j.bbrc.2009.12.045	Takayuki Kawaguchi, 2010	Japan	To determine the precise roles of v-SNARE (VAMP2) and t-SNAREs (syntaxin4, SNAP23) in specific steps of GLUT4 trafficking (mobilization, tethering/docking, and fusion) in adipocytes.	Differentiated 3T3-L1 adipocytes.	Stimulation: Insulin (100 nM).<br><br>Knockdown: siRNA via electroporation for VAMP2, Syntaxin4, or SNAP23.<br><br>Reporter: GLUT4myc7-GFP via retrovirus.	Translocation/Fusion: Confocal immunofluorescence microscopy (GFP vs. surface Myc).<br><br>Docking: Plasma membrane lawn assay (tethering assessment).<br><br>Function: 2-deoxy-D-glucose transport assay.<br><br>Expression: Immunoblot analysis.	Stimulation: 20 minutes (translocation), 5 minutes (signaling).<br><br>Knockdown: 2 days post-electroporation.	Genetic: Control siRNA vs. Target siRNA.<br><br>State: Basal vs. Insulin.	Knockdown of the v-SNARE VAMP2 using interfering RNA (siRNA) in 3T3-L1 adipocytes inhibited the fusion of GLUT4 vesicles with the plasma membrane, but did not affect their tethering ability. Conversely, removal of the t-SNAREs syntaxin4 or SNAP23 completely inhibited both tethering and fusion of GLUT4 vesicles.<br><br>The study confirms that t-SNAREs are essential components for keeping GLUT4 vesicles docked/tethered at the plasma membrane, while VAMP2 only plays a role in facilitating subsequent membrane fusion.	VAMP2, Syntaxin4, SNAP23	Stimulation (Docking/Fusion)	The study provides direct evidence distinguishing SNARE roles: t-SNAREs (Syntaxin4 and SNAP23) are indispensable for the tethering/docking step of GLUT4 vesicles at the plasma membrane (a role previously attributed mainly to the exocyst), whereas the v-SNARE (VAMP2) is not required for docking but is essential for the subsequent fusion step. This suggests t-SNAREs may coordinate with the exocyst during docking.	Tethering Fusion										Unclear risk (Not reported)	Unclear risk (Not reported)	Low risk	Unclear risk (Not reported)	Unclear risk (Not reported)	Low risk

A.738	10.1042/bj3120393	Purificación Muñoz, 1995	Spain	To determine the distribution of insulin-responsive membrane proteins (GLUT4, insulin receptor, SCAMPs) at transverse tubules (T-tubules) and the sarcolemma of rat skeletal muscle, and to assess their redistribution upon insulin stimulation.	Male Wistar rats (250-300g); white portions of gastrocnemius and quadriceps muscles.	Physiological Stimulation: Intravenous Insulin injection (10 units/kg) and intraperitoneal D-glucose injection (1 g/kg).	Subcellular Fractionation: Combined with wheat germ agglutinin (WGA) agglutination to separate sarcolemma and T-tubules.<br>Vesicle Immunoisolation: Using agarose bead-coupled antibodies.<br>Protein Analysis: Immunoblot analysis (Western blot).	30 minutes post-insulin injection.	State Comparison: Basal/Fasted (Overnight) vs. Insulin-injected group.	In rat skeletal muscle, insulin stimulates the recruitment of GLUT4 (as well as transferrin and integrin α2/β1 receptors) primarily to the transverse tubule segment (T-tubule) rather than the sarcolemma.<br>Analysis showed that T-tubules contain five times more GLUT4 than the basal sarcolemma, and insulin increased the GLUT4 content in T-tubules by 2.4 times.	GLUT4, Insulin Receptor, SCAMPs (SCAMP 37, 39).	Stimulation (Recruitment to T-tubules)	The transverse tubule (T-tubule) is identified as a major cell surface destination for insulin-regulated membrane protein recycling. Insulin stimulates GLUT4 translocation specifically to T-tubules (which possess a larger surface area in fast-twitch muscle than the sarcolemma) to facilitate direct glucose uptake for adjacent glycolytic enzymes and mitochondria. Concurrently, insulin receptor endocytosis also actively occurs at this site.	Translocation	Unclear risk (Not reported)	Low risk	Unclear risk (Not reported)	Unclear risk (Not reported)	Unclear risk (Not reported)	Unclear risk (Not reported)	Unclear risk (Not reported)	Unclear risk (Not reported)	Unclear risk (Not reported)							
A.740	10.1073/pnas.202495599	Louise Chang, 2002	USA	To identify effector proteins of TC10 and elucidate the role of the newly identified TC10-interacting protein CIP4/2 in insulin-stimulated glucose transport and Glut4 translocation.	3T3-L1 adipocytes and COS-1 cells (for protein interaction).	Stimulation: Insulin (1–30 minutes).<br>Overexpression: Myc-CIP4/2 (Wild-type and mutants), HA-TC10 (Wild-type, Constitutively active Q75L, Dominant-negative T31N).	Interaction: Yeast two-hybrid screen, Immunoprecipitation, Western blot, In vitro GST pull-down assay.<br>Localization/Function: Immunofluorescence microscopy for protein localization and Glut4 translocation assay.	Glut4 Translocation: 30 minutes post-insulin.<br>CIP4/2 Translocation: Time-course (0, 1, 2, 5, 10, 30 minutes).	State Comparison: Basal (No insulin) vs. Insulin-stimulated.<br>Genetic Comparison: Vector control vs. CIP4/2 WT vs. CIP4/2 Mutants.	The study identifies CIP4/2 (Cdc42-interacting protein 4/2) as a direct effector interacting with the GTPase TC10. CIP4/2 resides in an intracellular compartment under basal conditions but translocates to the plasma membrane upon insulin stimulation. This translocation is blocked by a dominant-negative TC10 mutant. Overexpression of CIP4/2 mutants (lacking the N-terminus or with reduced TC10 binding) inhibits insulin-stimulated GLUT4 translocation, demonstrating the critical role of this protein in the insulin signaling pathway.	CIP4/2 (Cdc42-interacting protein 4/2)	Stimulation	CIP4/2 acts as a downstream effector of TC10. Upon insulin activation of TC10 (via the CAP/Cbl/CrkII/C3G pathway), TC10 recruits CIP4/2 from the intracellular compartment to the plasma membrane. At the membrane, CIP4/2 functions as an adaptor protein (via FCH, coiled-coil, and SH3 domains) to coordinate cytoskeletal events or protein interactions required for the translocation and docking of Glut4 vesicles.	Translocation<br>Docking										Unclear risk (Not reported)	Unclear risk (Not reported)	Low risk	Unclear risk (Not reported)	Unclear risk (Not reported)	Unclear risk (Not reported)	
A.742	10.1074/jbc.275.10.7167	Makoto Kanzaki, 2000	USA	To investigate the potential role of trimeric GTP-binding proteins (specifically Gα subunits Gq, Gi, Gs) in regulating GLUT4 translocation in adipocytes.	Differentiated 3T3-L1 adipocytes.	Stimulation: Insulin (100 nM for 30 min).<br>Genetic: Co-expression of GLUT4-EGFP with constitutively active Gα mutants (Gαq-Q209L, Gαi-Q205L, Gαs-Q227L) or wild-type subunits.<br>Microinjection: Cytoplasmic injection of anti-Gαq/11 antibody.<br>Chemical: Wortmannin (PI3K inhibitor).	Imaging: Confocal fluorescence microscopy (GLUT4-EGFP).<br>Quantification: Single cell microinjection and counting of cells with plasma membrane GLUT4 rims.	30 minutes post-insulin stimulation.	State Comparison: Basal vs. Insulin-stimulated.<br>Genetic Comparison: Empty vector/Irrelevant G proteins (Gi, Gs) vs. Gq/G11 expression.<br>Injection Comparison: Control IgG vs. Anti-Gαq/11 antibody.	Microinjection of anti-Gαq/11 antibodies inhibits GLUT4 translocation, thus demonstrating the endogenous role of this protein.<br>The continuously activated mutant expression of Gq (Gq/Q209L) prevents insulin-stimulated GLUT4 translocation, while similar mutations in Gi and Gs do not produce this effect.<br>The Gq/Q209L mutant inhibits GLUT4 translocation but does not affect upstream signaling such as IRS-1 phosphorylation, PI3K activity, or Akt.	Gαq and Gα11 (Trimeric GTP-binding protein subunits)	Stimulation (Physiologically required)	The study identifies the alpha subunit of the Gq/G11 heterotrimeric G-protein family as an essential intermediate component in the insulin signaling pathway leading to GLUT4 translocation in adipocytes. Gq/G11 activation occurs downstream of the insulin receptor and likely operates in parallel or in concert with PI3K (as Gq effects remain wortmannin-sensitive).	Signaling										Unclear risk (Not reported)	Unclear risk (Not reported)	Low risk	Unclear risk (Not reported)	Unclear risk (Not reported)	Unclear risk (Not reported)	
A.743	10.1074/jbc.275.17.12889	Naoki Nakashima, 2000	USA	To determine the role of PTEN in insulin signaling by overexpressing PTEN in 3T3-L1 adipocytes using an adenovirus gene transfer system.	Differentiated 3T3-L1 adipocytes.	Stimulation: Insulin (0.5 nM, 17 nM, or 100 ng/ml).<br>Genetic: Adenovirus infection expressing PTEN (Ad-PTEN) or GFP (Ad-GFP, control) at various MOIs.<br>Microinjection: Anti-PTEN antibody (5 mg/ml) or control IgG nuclear injection.<br>Chemical: Wortmannin (100 nM, PI3K inhibitor).	Function: 2-Deoxyglucose uptake assay.<br>Imaging: Immunofluorescence microscopy for GLUT4 and PTEN translocation; Membrane ruffling assay (rhodamine-phalloidin).<br>Signaling: Western blotting (p-Akt, p70S6K, MAPK, IRS-1, IR); PI 3-Kinase Activity assay	Translocation: 20 minutes post-insulin.<br>Uptake: 1 hour post-insulin.<br>Signaling: 5–15 minutes post-insulin.<br>Expression: 72–96 hours post-infection.	State Comparison: Basal vs. Insulin.<br>Genetic Comparison: Uninfected/Ad-GFP vs. Ad-PTEN.<br>Injection Comparison: IgG vs. Anti-PTEN antibody.	Overexpression of PTEN (a lipid phosphatase) significantly reduces insulin-stimulated glucose uptake and GLUT4 translocation. PTEN functions by reducing PIP3 levels, thereby preventing Akt kinase activation (without affecting MAPK). A catalytically inactive PTEN mutant (C124S) enhances insulin signaling, leading to increased glucose uptake and GLUT4 translocation.	PTEN (Phosphatase and tensin homolog)	Inhibition	PTEN acts as a negative regulator of the PI3K-dependent insulin signaling pathway in adipocytes. By dephosphorylating PtdIns 3,4,5-P3 (PIP3) at the D-3 position (3'-phosphatase activity), PTEN decreases intracellular PIP3 levels. This prevents the activation of downstream kinases such as Akt and p70S6K, resulting in reduced GLUT4 translocation and glucose uptake. Endogenous PTEN plays a physiological role in controlling the efficiency of this pathway	Signaling										Unclear risk (Not reported)	Unclear risk (Not reported)	Low risk	Unclear risk (Not reported)	Unclear risk (Not reported)	Low risk	
A.744	10.1074/jbc.M508317200	Avirup Bose, 2005.	USA	To identify protein components of specialized GLUT4-containing vesicles in adipocytes via proteomic analysis, thereby discovering Vti1a and investigating its role in insulin-stimulated GLUT4 trafficking and Acrp30 secretion.	Differentiated 3T3-L1 adipocytes.	Stimulation: Insulin (1 nM, 100 nM for 30 min).<br>Genetic: Knockdown of Vti1a using siRNA (30 nmol) via electroporation.<br>Chemical: Brefeldin A (BFA, 10 µg/ml) to disrupt the Golgi apparatus.	Identification: Mass spectrometry.<br>Localization: Subcellular fractionation (sucrose gradient), Immunofluorescence microscopy with deconvolution.<br>Function: 2-Deoxyglucose uptake assay.<br>Expression: Western blotting.	Stimulation: 30 minutes.<br>Knockdown: 72 hours post-transfection.	State Comparison: Basal vs. Insulin.<br>Genetic Comparison: Scrambled siRNA (Control) vs. Vti1a siRNA.<br>Chemical Comparison: Vehicle vs. Brefeldin A.	The v-SNARE protein Vti1a has been identified as a structural component of insulin-sensitive GLUT4-containing membranes.<br>In its basal state, Vti1a is located in the same position as GLUT4 in the perinuclear region. Reduced Vti1a expression (knockdown) negatively impacts adipocyte function, specifically glucose transport and adiponectin secretion.	Vti1a	Stimulation	The study identifies Vti1a as a novel v-SNARE component of specialized insulin-sensitive GLUT4 vesicles. Vti1a regulates a critical common step in the trafficking of both GLUT4 and Acrp30, likely the budding or exit from intracellular storage compartments to travel to the plasma membrane. The presence of Vti1a in a BFA-resistant compartment supports the distinction of the GLUT4 storage compartment from the trans-Golgi network (TGN).	Fussion										Unclear risk (Not reported)	Unclear risk (Not reported)	Low risk	Unclear risk (Not reported)	Unclear risk (Not reported)	Unclear risk (Not reported)	
A.745	10.1074/jbc.M206936200	Luke H. Chamberlain, 2002	UK	To determine the localization of SNARE proteins (SNAP-23, syntaxin 4, VAMP2) involved in GLUT4 vesicle fusion within detergent-insoluble lipid rafts in 3T3-L1 adipocytes, clarifying the role of lipid rafts as platforms for fusion.	Differentiated 3T3-L1 adipocytes.	Stimulation: Insulin (1 µM for 30 min).<br>Chemical: Methyl-beta-cyclodextrin (MβCD) treatment for 45 min to deplete cholesterol.<br>Detergent: Triton X-100 (0.2%, 0.5%, 1.0%) for raft isolation.	Isolation: Triton X-100 solubilization and Sucrose Gradient Centrifugation.<br>Analysis: Subcellular Fractionation, Immunofluorescence Analysis of PM Lawns, Immunoblotting.	Stimulation: 30 minutes.<br>Depletion: 45 minutes post-MβCD.	State Comparison: Basal vs. Insulin.<br>Treatment Comparison: Control vs. MβCD (cholesterol depletion).<br>Fraction Comparison: Raft vs. Non-raft fractions.	The majority of SNAP-23 (>70%) and a significant proportion of Syntaxin 4 (~20%) are localized in detergent-insoluble lipid rafts. Approximately 25% of VAMP2 is also found in these lipid raft fragments.<br>The lipid rafts containing t-SNARE (SNAP-23/Syntaxin 4) and v-SNARE (VAMP2) are located on separate cell membranes, not the same membrane. Specifically, the VAMP2-containing lipid raft is located on the intracellular membrane, while the SNAP-23-containing lipid raft is located on the plasma membrane.<br>Disruption of the lipid raft structure by removing cholesterol (using methyl-β-cyclodextrin) inhibits insulin-stimulated glucose uptake.	SNAP-23, Syntaxin 4, VAMP2	Stimulation	The study proposes that lipid rafts serve as critical spatial platforms for GLUT4 vesicle fusion. The presence of t-SNAREs (on the plasma membrane) and v-SNAREs (on intracellular vesicles) both within lipid rafts suggests that SNARE interactions occur in this specialized lipid environment. This organization likely concentrates fusion proteins or facilitates membrane lipid mixing, potentially serving as a spatial integration point between insulin signaling (also raft-associated) and the membrane trafficking machinery.	Fusion										Unclear risk (Not reported)	Unclear risk (Not reported)	Low risk	Unclear risk (Not reported)	Unclear risk (Not reported)	Low risk	
A.748	10.3389/fendo.2021.653557	Man Mohan Shrestha, 2021	Singapore	To identify novel AMPK effectors that directly regulate GLUT4 translocation, specifically examining the role of Tmod3 phosphorylation by AMPK in GLUT4 insertion into the plasma membrane.	L6 myoblasts stably expressing myc-tagged GLUT4 (L6-GLUT4-myc).	Pharmacological: AICAR (2 mM, AMPK activator), Insulin (100 nM).<br>Genetic: Tmod3 knockdown (siRNA); Overexpression of Wild-type Tmod3 (WT) and mutants (S71A - non-phosphorylatable, S71D - phosphomimetic).<br>Chemical: Compound C (AMPK inhibitor), Latrunculin B (LatB - actin disruptor).	Proteomics: Quantitative phosphoproteomics.<br>Enzyme Activity: In vitro kinase assay.<br>Translocation: Colorimetric assay & Immunofluorescence (Surface GLUT4-myc).<br>Cytoskeleton: Actin polymerization assay (using pyrene).<br>Protein Analysis: Western blotting.	Translocation: 20 minutes (insulin) or 40 min – 2 hours (AICAR).<br>Phosphorylation: 30 minutes post-AICAR.	State Comparison: Basal/Vehicle vs. Stimulated (AICAR/Insulin).<br>Genetic Comparison: Control siRNA/Vector vs. Intervention (Tmod3 siRNA, Mutants).	Thyroid hormone (T3) significantly increases Akt phosphorylation, the translocation of VAMP2 and GLUT4 to the membrane, and insulin-induced glucose uptake.<br>Akt inhibitors (Akt inhibitor X) completely eliminate the agonistic effect of T3, demonstrating that T3 acts via the Akt pathway.<br>When VAMP2 is suppressed by siRNA, the effect of T3 on GLUT4 translocation and glucose uptake is lost. This identifies VAMP2 as a crucial mediator of T3's action.	AMPK	Stimulation	The study identifies a novel signaling pathway where AMPK activates Tmod3 via phosphorylation at Ser71. This event modulates actin cytoskeletal dynamics (enhancing actin polymerization), facilitating the insertion/fusion step of GLUT4 vesicles into the plasma membrane. This mechanism is specific to the AMPK pathway and independent of the Insulin pathway (which relies on Akt/Rac1).	Fusion										Unclear risk (Not reported)	Unclear risk (Not reported)	Low risk	Unclear risk (Not reported)	Unclear risk (Not reported)	Low risk	
A.749	10.1074/jbc.M304261200	Charlotte H. Widberg, 2003	Australia	To investigate the interaction between b-Tomosyn and the adipocyte SNARE complex (Syntaxin4/SNAP23/VAMP-2) and the SM protein Munc18c, and to determine the role of b-Tomosyn in insulin-stimulated GLUT4 translocation.	3T3-L1 adipocytes and CHO-T cells (CHO cells overexpressing human insulin receptor).	Stimulation: Insulin (100 nM or 1 µM).<br>Overexpression: Myc-tagged b-Tomosyn (b-Tom-Myc) via pCX4 retroviral vector.<br>Interaction: Use of recombinant GST-fusion proteins.	Translocation: Plasma membrane lawn assay combined with quantitative immunofluorescence (HA-GLUT4).<br>Interaction: Immunoprecipitation, GST pull-down assay.<br>Expression: Western blotting.	20 minutes post-insulin stimulation (for translocation assay).	Genetic Comparison: Mock/Control vector infected cells vs. b-Tomosyn expressing cells.<br>State Comparison: Basal vs. Insulin.	Studies confirm the presence of the β-Tomosyn isoform (130 kDa) in 3T3-L1 adipocytes.<br>β-Tomosyn interacts with Syntaxin 4 and SNAP23 (important t-SNAREs for GLUT4) but not with Munc18c.<br>Overexpression of β-Tomosyn significantly inhibits the translocation of GLUT4 to the plasma membrane under the influence of insulin. This suggests that β-Tomosyn plays a negative regulatory role in the exocytosis of GLUT4.	b-Tomosyn (Syntaxin-binding protein)	Inhibition	b-Tomosyn acts as a negative regulator of GLUT4 trafficking. The proposed mechanism is that b-Tomosyn (via its C-terminal VAMP-like domain) mimics VAMP2 to bind t-SNAREs (Syntaxin4/SNAP23), forming futile complexes. This prevents VAMP2 (on GLUT4 vesicles) from binding and fusing with the plasma membrane. Additionally, Tomosyn may regulate Munc18c function.	Fusion										Unclear risk (Not reported)	Unclear risk (Not reported)	Low risk	Unclear risk (Not reported)	Unclear risk (Not reported)	Low risk	
A.750	10.1016/j.biocel.2015.02.013	Koki Nagano, 2015	Japan	To identify the role of tomosyn as a novel Akt substrate in insulin-dependent GLUT4 exocytosis, specifically determining the phosphorylation site and the regulatory mechanism of SNARE interactions.	Differentiated 3T3-L1 adipocytes.	Stimulation: Insulin (1 nM, 10 nM, 100 nM for 5–20 min).<br>Inhibition: MK-2206 (Akt inhibitor, 10 µM).<br>Genetic: siRNA-knockdown of Tomosyn; Overexpression of Wild-type (WT) and Mutants (S724A - non-phosphorylatable, S724D - phosphomimetic).	Phosphorylation: Phos-tag SDS-PAGE, In vitro kinase assay.<br>Interaction: Immunoprecipitation, Western blotting.<br>Function: Immunofluorescence (GLUT4 externalization), 2-Deoxyglucose uptake assay.	5 to 20 minutes post-insulin.	State Comparison: Basal vs. Insulin.<br>Treatment Comparison: Control/DMSO vs. Inhibitor (MK-2206).<br>Genetic Comparison: WT vs. Mutants (S724A, S724D).	Studies have identified the protein Tomosyn (specifically β-tomosyn 130-kDa) as a novel substrate of Akt2 kinase in adipocytes. Insulin stimulates phosphorylation of Tomosyn at the Serine [illegible] site.<br>In its unphosphorylated state, Tomosyn binds tightly to Syntaxin-4 and prevents vesicle fusion. When phosphorylated by Akt (simulated by the [illegible] mutation), Tomosyn's affinity for Syntaxin-4 decreases, allowing Syntaxin 4 to participate in the SNARE complex for exocytosis.<br>Tomosyn acts as a brake preventing GLUT4 from escaping the membrane. Upon removal of Tomosyn (knockdown), the amount of GLUT4 on the cell surface increases even under basal conditions.	Tomosyn (STXBP5)	Inhibition	Tomosyn acts as a negative regulator (sequestering Syntaxin-4). Insulin activates Akt, which phosphorylates Tomosyn at Ser724. This event induces a conformational change that causes Tomosyn to dissociate from Syntaxin-4. The released Syntaxin-4 can then participate in the SNARE complex (with SNAP23 and VAMP2) to promote the fusion of GLUT4 vesicles with the plasma membrane.	Fusion<br>Docking										Unclear risk (Not reported)	Unclear risk (Not reported)	Low risk	Unclear risk (Not reported)	Unclear risk (Not reported)	Low risk	
A.754	10.1083/jcb.117.6.1181	Linda J. Robinson, 1992.	USA	To develop a permeabilized cell system to study the mechanism of insulin-stimulated GLUT4 translocation, specifically investigating the roles of ATP and GTP-binding proteins.	3T3-L1 adipocytes and 3T3-L1 fibroblast transfectants expressing GLUT4.	Stimulation: Insulin (10^{-7} M).<br>Permeabilization: Streptolysin-O (SLO, 0.8 IU/ml).<br>Chemicals: GTPγS (100 µM - non-hydrolyzable GTP analog), ATP (or regenerating system), Apyrase (to deplete ATP).	Imaging: PM Lawn Technique combined with immunofluorescence microscopy and electron microscopy (EM).<br>Labeling: Immunogold labeling.<br>Confocal Microscopy: Confocal laser immunofluorescence microscopy.	15 minutes post-incubation with stimuli.	State Comparison: Intact vs. Permeabilized cells; Basal vs. Insulin.<br>Treatment Comparison: +ATP vs. -ATP; Control vs. GTPγS.	In permeabilized fat cells, the removal of ATP or the addition of apyrase completely prevents the translocation of GLUT4 to the membrane under the influence of insulin. This demonstrates that the translocation is ATP energy-dependent.<br>GTPγS (a non-hydrolyzable homologue of GTP) is capable of self-stimulating GLUT4 translocation in the absence of insulin, but inhibits the effect of insulin when added.<br>At the plasma membrane, GLUT4 is mainly concentrated in clathrin lattices and clathrin-coated pits, suggesting a clathrin-mediated internalization mechanism.	ATP, GTP-binding protein, Clathrin	Stimulation	The study proposes that GLUT4 translocation requires ATP at an early signaling stage and one (or more) GTP-binding proteins at a later stage (fusion/translocation). The association of GLUT4 with clathrin lattices may be critical for maintaining intracellular retention in the absence of insulin. Insulin and GTPγS likely operate cooperatively.	Translocation<br>Fusion<br>Signaling										Unclear risk (Not reported)	Unclear risk (Not reported)	Low risk	Unclear risk (Not reported)	Unclear risk (Not reported)	Low risk	
A.757	10.1038/ncomms6951	Chun-Yan Lim, 2015	Singapore	To identify novel Akt2 effectors and elucidate the role of Tropomodulin 3 (Tmod3) in regulating GLUT4 exocytosis via cortical actin remodeling.	3T3-L1 adipocytes.	Stimulation: Insulin.<br>Genetic: Knockdown of Akt2, Tmod3; Expression of Tmod3 mutants (S71A, S71D).	Identification: Mass spectrometry.<br>Function: Glucose uptake assay, GLUT4 PM insertion assay.<br>Imaging: Actin remodeling assay.		State Comparison: Basal vs. Insulin.<br>Genetic Comparison: Wild-type vs. Knockdown/Mutant Tmod3 cells.	"Using mass spectrometry analysis, the study identified Tropomodulin 3 (Tmod3 – an actin filament capping protein) as the specific interacting partner of Akt2 in adipocytes.<br>Insulin stimulates Akt2 phosphorylation of Tmod3 at the Serine 71 site. Mutations at this site result in loss of insulin responsiveness.<br>Phosphorized Tmod3 (p-Tmod3) plays a vital role in cortical actin remodeling to allow GLUT4 vesicle fusion. The interaction between Tmod3 and Tropomyosin (Tm5NM1) is essential for this process.	Tmod3 (Tropomodulin 3)	Stimulation	Akt2 phosphorylates Tmod3 at Ser71, triggering cortical actin remodeling. This reorganization of the actin cytoskeleton creates the necessary conditions for the fusion of GLUT4-containing vesicles with the plasma membrane.	Fusion	Unclear risk (Not reported)	Low risk	Unclear risk (Not reported)	Unclear risk (Not reported)	Unclear risk (Not reported)	Unclear risk (Not reported)	Unclear risk (Not reported)	Unclear risk (Not reported)	Unclear risk (Not reported)	Unclear risk (Not reported)	Unclear risk (Not reported)	Low risk	Unclear risk (Not reported)	Unclear risk (Not reported)	Low risk	
A.758	10.1530/JME-19-0253	Pablo Pánico, 2020	Mexico	To determine if the protein TUG is a substrate of calpain-10 and the role of this cleavage in GLUT4 translocation in adipocytes.	3T3-L1 adipocytes.	Stimulation: Insulin.<br>Intervention: Inhibition or Knockdown of Calpain-10; Expression of TUG variants.	Proteomic analysis, Biochemical substrate assays.		State Comparison: Insulin vs. No Insulin.<br>Genetic Comparison: Normal vs. Calpain-10 inhibited/knockdown.	TUG (ASPL) contains a PEST sequence and is a substrate of calpain-10. Calpain-10 cleaves TUG at amino acid residue 166, generating characteristic fragments (18 kDa N-terminal; 42 kDa). This cleavage is stimulated by insulin. Calpain-10-mediated cleavage of TUG is a requisite step for releasing GLUT4 vesicles from intracellular retention, allowing them to translocate. Inhibition of calpain-10 prevents TUG processing and reduces GLUT4 translocation.	TUG and Calpain-10	Stimulation (via cleavage)	Calpain-10 cleaves TUG, a protein responsible for the intracellular retention of GLUT4. This proteolytic cleavage releases GLUT4-containing vesicles, enabling their translocation to the plasma membrane upon insulin stimulation.	Fusion										Unclear risk (Not reported)	Unclear risk (Not reported)	Low risk	Unclear risk (Not reported)	Unclear risk (Not reported)	Low risk	
A.761	10.1074/jbc.272.48.30208	Anna Krook, 1997	UK	To use naturally occurring insulin receptor mutants (Gln-1174, Leu-1178) to demonstrate that PI3K activation alone is insufficient to mediate the metabolic (GLUT4 translocation) and mitogenic effects of insulin.	CHO.K1 cells (Chinese Hamster Ovary fibroblasts, naturally lacking GLUT4).	Stimulation: Insulin (1–100 nM).<br>Genetic: Overexpression of mutant Insulin Receptors (Gln-1174, Leu-1178); Microinjection of GFP-GLUT4 plasmid.	Imaging: Fluorescence microscopy.<br>Assays: PI3K assay, Immunoprecipitation & Western blotting, Reporter gene assays (Elk-1/Luciferase).	60 minutes post-insulin (for GLUT4).	Receptor Comparison: Wild-type vs. Mutant Insulin Receptor expressing cells.<br>State Comparison: Basal vs. Insulin.	Studies on two natural insulin receptor mutants (Gln-1174 and Leu-1178) showed that while these receptors can robustly activate PI 3-kinase (comparable to wild-type at high insulin concentrations), they completely fail to stimulate glycogen synthesis and DNA synthesis. This proves that PI3K activation alone is insufficient to mediate insulin's metabolic and mitogenic effects. Both mutants showed severely impaired tyrosine autophosphorylation compared to wild-type.	PI3K	Stimulation (Necessary but not sufficient)	The study demonstrates that while PI3K activation (combined with IRS-1) is necessary, it is not sufficient to trigger GLUT4 translocation. Additional signals (potentially involving Shc or other unidentified pathways) are required to fully execute this metabolic function.	Signaling										Unclear risk (Not reported)	Unclear risk (Not reported)	Low risk	Unclear risk (Not reported)	Unclear risk (Not reported)	Low risk	
A.762	10.1210/en.142.7.2833	Diana L. Esposito, 2001	USA and Italy	To determine the role of specific YXXM motifs (Tyr612 and Tyr632) on IRS-1 in activating PI3K and mediating GLUT4 translocation in adipocytes.	Isolated rat adipose cells (Primary adipocytes).	Stimulation: Insulin (0-60 nM).<br>Genetic: Electroporation of plasmids encoding GLUT4-HA and IRS-1 mutants (IRS1-F6, IRS1-Y612, IRS1-Y632, IRS1-Y612/Y632).	Translocation: Assay for cell surface epitope-tagged GLUT4 (anti-HA and ^{125}I-labeled secondary antibody).<br>Interaction/Activity: Co-immunoprecipitation & PI3K assay.	Stimulation: 30 minutes.<br>Expression: 20 hours post-electroporation.	Genetic Comparison: Wild-type IRS-1 vs. Mutant IRS-1 expressing cells.<br>State Comparison: Basal vs. Insulin.	Tyrosine 612 and 632 on the human IRS-1 molecule were identified as the principal phosphorylation sites responsible for binding and activating PI 3-kinase. The double mutant replacing both Tyrosines with Phenylalanine (IRS-1 Y612F, Y632F) reduced IRS-1-associated PI 3-kinase activity by ~60-70% and correspondingly reduced insulin-stimulated GLUT4 translocation. This confirms a tight correlation between the level of PI3K activation via IRS-1 and the magnitude of GLUT4 translocation.	IRS-1 (Insulin receptor substrate-1)	Stimulation	The study pinpoints the YXXM pair at positions 612 and 632 on IRS-1 as sufficient and necessary for full activation of PI3K and the execution of GLUT4 translocation in adipocytes.	Signaling										Unclear risk (Not reported)	Unclear risk (Not reported)	Low risk	Unclear risk (Not reported)	Unclear risk (Not reported)	Low risk	
A.764	10.1210/en.2006-1549	Mitsuhiko Umahara, 2008.	Japan and USA	To define the molecular mechanism by which PDGF stimulates GLUT4 translocation and compare it with insulin, specifically focusing on the role of Munc18c phosphorylation.	Differentiated 3T3L1 adipocytes.	Stimulation: PDGF (5 nM), Insulin (100 nM).<br>Genetic: Overexpression of FLAG-Munc18c (Wild-type), FLAG-Y218A-Munc18c mutant, FLAG-Y521A-Munc18c mutant.	Protein Analysis: Immunoprecipitation, Immunoblotting.<br>Translocation: Colorimetric assay (Myc-GLUT4), Confocal fluorescence microscopy (eGFP-GLUT4).	0, 10, 20, 30 minutes post-stimulation.	State Comparison: Basal vs. PDGF vs. Insulin.<br>Genetic Comparison: Empty vector vs. Munc18c WT vs. Munc18c Mutants.	PDGF is less effective than insulin at stimulating GLUT4 translocation. This is because PDGF does not induce tyrosine phosphorylation of Munc18c and fails to dissociate Munc18c from Syntaxin 4, unlike insulin. Overexpression of wild-type Munc18c inhibited PDGF-stimulated GLUT4 translocation but had no effect on insulin's action. This suggests that Munc18c phosphorylation (which occurs with insulin) releases this inhibition.	Munc18c (Syntaxin 4 binding protein)	Inhibition (Relieved by phosphorylation)	The paper defines a distinct mechanism for PDGF: PDGF triggers Munc18c phosphorylation at Tyr521, which releases Syntaxin 4 from Munc18c inhibition, allowing GLUT4 translocation. In contrast, Insulin stimulates stronger GLUT4 translocation but does not primarily rely on Munc18c Tyr521 phosphorylation to release Syntaxin 4 (implying other mechanisms or sites are involved for insulin).	Fusion<br>Docking										Unclear risk (Not reported)	Unclear risk (Not reported)	Low risk	Unclear risk (Not reported)	Unclear risk (Not reported)	Low risk	
A.765	10.1128/MCB.24.12.5447-5458.2004	Avirup Bose, 2004.	USA	To determine the role of the atypical myosin motor Myo1c in the GLUT4 trafficking pathway and its relationship to PI3K-sensitive steps (specifically fusion).	Differentiated 3T3-L1 adipocytes.	Stimulation: Insulin (100 nM).<br>Chemicals: LY294002 (PI3K inhibitor), Latrunculin B (actin disruption), Colchicine (microtubule disruption).<br>Genetic: Overexpression of YFP-Myo1c (Wild-type) and Myo1c(T) (Dominant-negative / cargo-binding domain only).	Imaging: Ultrafast 3D microscopy, TIRF microscopy, Live-cell imaging.<br>Assay: Myc-GLUT4-CFP internalization assay.	30 minutes post-insulin; Time-lapse every 5–10 seconds for 10–15 minutes.	Treatment Comparison: Control vs. Insulin vs. LY294002 + Insulin.<br>Genetic Comparison: Myc-GLUT4 alone vs. Co-expression with Myo1c.<br>Region Comparison: Ruffling vs. Non-ruffling membrane areas.	When 3T3-L1 adipocytes are treated with the PI 3-kinase inhibitor LY294002, GLUT4 vesicles still translocate to the plasma membrane region (evidenced by sensitivity to extracellular proteases) but fail to fuse/expose the transporter. Expression of a dominant-negative Myo1c mutant (myosin I tail) causes a similar effect to PI 3-kinase inhibition: GLUT4 vesicles accumulate at the membrane but are inhibited from fusing. This identifies Myo1c as a promoter of the membrane fusion step.	Myo1c (Unconventional myosin)	Stimulation	Myo1c plays a dual role: (1) Tethering/Transporting GLUT4 vesicles to the cortical actin zone, and (2) Promoting membrane fusion via the generation of membrane ruffles and local actin remodeling. The mechanical activity of Myo1c at these hot spots facilitates fusion, acting downstream or in parallel with PI3K signaling.	Translocation/ tethering/ fusion										Unclear risk (Not reported)	Unclear risk (Not reported)	Low risk	Unclear risk (Not reported)	Low risk	Low risk	
A.768	10.1016/S0021-9258(18)38244-X	Shinobu Satoh,1993	USA and UK	To elucidate the subcellular trafficking kinetics of GLUT4 regulated by insulin in rat adipocytes using the bismannose photolabel (ATB-BMPA), specifically to determine whether insulin regulates the endocytosis or exocytosis step.	Isolated rat adipose cells.	Stimulation: Insulin (0.7 nM).<br>Chemicals: Collagenase (for reversal), KCN (to stop transport), ATB-BMPA (photolabel).	Labeling: Bismannose photolabeling combined with immunoprecipitation.<br>Analysis: Subcellular fractionation, 3-O-Methyl-D-glucose transport assay, Kinetic modeling (2-compartment model).	Stimulation: 30 minutes.<br>Reversal: Monitored over 60–120 minutes.	State Comparison: Basal vs. Insulin-stimulated vs. Collagenase-reversed.	Using the membrane-impermeant photolabel ATB-BMPA, the study demonstrated that the major effect of insulin on GLUT4 distribution is to stimulate the exocytosis rate of GLUT4 vesicles from the intracellular pool to the plasma membrane, rather than inhibiting endocytosis. The cycle time for a GLUT4 molecule was calculated to be ~47 minutes in the basal state, decreasing to 16 minutes under insulin stimulation.	GLUT4	Stimulation (of Exocytosis)	The study confirms that the primary mechanism of insulin action is to promote the exocytosis step (specifically docking/fusion) of GLUT4 rather than inhibiting endocytosis. The kinetic data also suggest the existence of intermediate states on the plasma membrane during GLUT4 trafficking.											Unclear risk (Not reported)	Unclear risk (Not reported)	Low risk	Unclear risk (Not reported)	Unclear risk (Not reported)	Low risk	
A.770	10.1074/jbc.M302094200	Takashi Katome, 2003.	Japan	To clarify the specific roles of individual Akt isoforms (Akt1, Akt2, Akt3) in insulin actions (GLUT4 translocation, glucose uptake, glycogen synthesis) using RNA interference (siRNA) and adenoviral overexpression.	3T3-L1-GLUT4myc adipocytes and CHO-GLUT4myc cells.	Stimulation: Insulin (100 nM).<br>Genetic: siRNA specific for Akt1, Akt2, Akt3; Adenovirus expressing Constitutively Active (CA-Akt) and Dominant Negative (DN-Akt).	Function: GLUT4 translocation assay (surface myc), 2-Deoxyglucose (2-DG) uptake assay, Glycogen synthesis assay.<br>Signaling: In vitro kinase assay, Western blotting.	Stimulation: 10 minutes.<br>Expression: 48 hours post-infection/transfection.	Control: GFP/siGFP vs. Mutants/siRNA.<br>State: Basal vs. Insulin.<br>Isoform: Akt1 vs. Akt2 vs. Akt3 silencing.	Specific gene silencing revealed that Akt2 deficiency severely reduces insulin-stimulated glucose uptake and GLUT4 translocation, whereas Akt1 deficiency has no significant effect on these processes. Akt1 plays a major role in activating p70 S6 kinase (related to protein synthesis/growth), while Akt2 has a minor role in this pathway. Although they have distinct endogenous roles, overexpression of Akt1 can compensate for Akt2 function in glucose transport, suggesting specificity depends on expression levels or localization rather than catalytic structure.	Akt1, Akt2, Akt3	Stimulation	The study defines non-redundant functional roles for Akt isoforms. Akt2 is more specialized for metabolic glucose effects (GLUT4 translocation), whereas Akt1 and Akt2 share roles in glycogen synthesis. This reinforces the central role of Akt in insulin signaling leading to GLUT4.	Signaling										Unclear risk (Not reported)	Unclear risk (Not reported)	Low risk	Unclear risk (Not reported)	Unclear risk (Not reported)	Low risk	

A.771	10.1074/jbc.RA118.003021	Estifanos N. Habtemichael, 2018	USA	To identify the protease responsible for TUG cleavage (Usp25m) and elucidate the mechanism by which Usp25m regulates ubiquitin-like processing of TUG to control GLUT4 translocation in adipocytes.	3T3-L1 adipocytes and Primary adipocytes.	Stimulation: Insulin (80-160 nM).<br>Diet: High Fat Diet (HFD) or High Fat High Sugar (HFS).<br>Genetic: Knockdown of Usp25m/KIF5B; Overexpression of Usp25m and TUG mutants (GGVV, GGYY).	Protein Analysis: Immunoblotting, Immunoprecipitation.<br>Function: Cell surface biotinylation (GLUT4/IRAP translocation).<br>Imaging: Confocal microscopy.<br>Expression: Quantitative RT-PCR.	Stimulation: 15–30 minutes.<br>Diet: 3 days or 3 weeks.	Genetic: Control shRNA vs. Usp25m shRNA.<br>State: Basal vs. Insulin.<br>Diet: Regular Chow vs. HFD.<br>Mutant: WT TUG vs. Mutant TUG.	The study identifies Usp25m (a deubiquitinating protease) as a specific interactor of TUG (the GLUT4 retention protein). Usp25m removes a ubiquitin-like modifier from TUG. This activity is a prerequisite for the subsequent proteolytic cleavage of TUG, which releases GLUT4 vesicles for translocation. Knockdown of Usp25m prevents TUG processing and inhibits insulin-stimulated GLUT4 translocation. Usp25m protein abundance is decreased in adipose tissue from HFD-fed mice, suggesting a role in insulin resistance.	Usp25m	Stimulation	Insulin activates Usp25m to process the retention protein TUG. This releases GLUT4 storage vesicles (GSVs) from the Golgi matrix. Concurrently, the generated TUGUL fragment binds to KIF5B, acting as a signal to load GSVs onto microtubules for transport to the plasma membrane. This is an Akt-independent pathway that is impaired in obesity.	Translocation	High risk	Low risk	Unclear risk (Not reported)	Unclear risk (Not reported)	High risk	High risk	High risk	Low risk	Unclear risk (Not reported)	Unclear risk (Not reported)	High risk	Low risk	High risk	High risk	Low risk	
A.772	10.1091/mbc.11.7.2403	Varinder K. Randhawa, 2000.	Canada	To determine the distinct roles of VAMP2 and VAMP3/cellubrevin in GLUT4 translocation in muscle cells using Tetanus toxin (TeTx) and toxin-resistant mutants.	L6-GLUT4myc myoblasts.	Stimulation: Insulin (100 nM).<br>Genetic: Transfection of Tetanus toxin (TeTx) light chain; Rescue with toxin-resistant mutants (VW-VAMP2, VW-VAMP3).	Function: Single-cell fluorescence assay (surface GLUT4).<br>Imaging: Immunofluorescence microscopy.<br>Isolation: Immunoadsorption of GLUT4 vesicles.	20 minutes post-insulin; 48 hours post-transfection.	State: Untransfected vs. TeTx transfected.<br>Rescue: TeTx alone vs. TeTx + VW-VAMP2 vs. TeTx + VW-VAMP3.	Although VAMP3 co-localizes with GLUT4, expression of toxin-resistant VAMP3 failed to rescue the inhibition of glucose transport caused by TeTx, confirming the functional specificity of VAMP2. Results indicate that despite structural homology, these two SNAREs have distinct roles: VAMP2 is specialized for insulin-stimulated GLUT4 fusion.	VAMP2 and VAMP3	Stimulation (VAMP2 specific)	The study confirms VAMP2 as the resident v-SNARE on insulin-responsive GLUT4 vesicles (GSVs) whose integrity is obligatory for the fusion of these vesicles with the plasma membrane in muscle cells. VAMP3, while present on some GLUT4 vesicles (likely recycling endosomes), does not participate in the primary fusion step of the insulin-sensitive pathway. Interaction with the cortical actin cytoskeleton is a distinguishing feature of VAMP2.	Fusion										Unclear risk (Not reported)	Unclear risk (Not reported)	Low risk	Unclear risk (Not reported)	Unclear risk (Not reported)	Low risk	
A.775	10.1074/jbc.273.3.1444	Laura B. Martin, 1998.	Australia and UK	To determine the specific role of VAMP2 in insulin-dependent GLUT4 trafficking and distinguish it from other v-SNAREs like VAMP1 and cellubrevin (VAMP3).	SLO-permeabilized 3T3-L1 adipocytes.	Stimulation: Insulin (1 μM), GTPγS (100 μM).<br>Introduction: GST-fusion proteins (VAMP1, VAMP2, Cellubrevin) or synthetic peptides (N-terminal VAMP2).<br>Ablation: Endosome ablation using Tf-HRP.	Assay: Plasma membrane lawn assay with immunofluorescence.<br>Fractionation: Differential centrifugation.<br>Protein Analysis: Western blotting.	15 minutes post-incubation.	Control: GST control vs. GST-VAMPs; Peptide control vs. VAMP2 peptide.<br>State: Ablated vs. Control.	Using Tetanus toxin (TeTx) to cleave both VAMP2 and Cellubrevin (VAMP3), insulin-stimulated GLUT4 translocation was inhibited by ~50%. Re-expression of resistant VAMP2 restored translocation, whereas resistant Cellubrevin (VAMP3) did not. This demonstrates VAMP2 has a specific role that VAMP3 cannot substitute. Immunofluorescence and fractionation showed VAMP2 is highly concentrated in the GLUT4-containing compartment (LMM) and translocates to the membrane with insulin.	VAMP2 and VAMP3	Stimulation (VAMP2 specific)	VAMP2 plays a specific and unique role in regulating the fusion of specialized GLUT4 vesicles (GSVs) with the plasma membrane upon insulin signaling. In contrast, Cellubrevin/VAMP3 is likely responsible for the constitutive endosomal recycling pathway of proteins like GLUT1. The N-terminus of VAMP2 is a critical functional domain conferring this specificity.	Fusion										Unclear risk (Not reported)	Unclear risk (Not reported)	Low risk	Unclear risk (Not reported)	Unclear risk (Not reported)	Low risk	
A.780	10.1371/journal.pone.0216236	Edward B. Arias, 2019	USA	To determine the whole-body glucose regulation phenotype and tissue-specific glucose uptake (muscle, adipose) in a novel AS160 knockout (AS160-KO) rat model.	AS160 knockout Wistar rats (AS160-KO rats) and Wild-type (WT) littermates. Isolated tissues: Soleus muscle, Epitrochlearis muscle, Epididymal adipose tissue.	Genetic: AS160 gene knockout.<br>Stimulation: Insulin (physiological and supraphysiological doses).	Metabolism: 2-Deoxyglucose uptake assay (ex vivo), Glucose tolerance test (GTT - in vivo).<br>Protein Analysis: Immunoblotting (Western blot).	Ex vivo: 30–60 minutes insulin incubation.<br>In vivo: Monitored over 120 minutes (GTT).	Genotype Comparison: Wild-type (WT) vs. Knockout (KO).<br>State Comparison: Basal vs. Insulin.<br>Tissue Comparison: Muscle vs. Adipose.	AS160-KO rats exhibit impaired glucose tolerance compared to WT rats, despite unchanged plasma insulin concentrations. In KO rats, insulin-stimulated glucose uptake is significantly reduced in various muscle types (epitrochlearis and soleus), accompanied by reduced GLUT4 protein abundance. Conversely, isolated adipocytes from KO rats show greater insulin-stimulated glucose uptake than WT, corresponding to a ~2-fold increase in GLUT4 protein abundance in adipose tissue.	AS160 (TBC1D4)	Inhibition (in muscle) / Stimulation (in adipose)	The study reinforces the essential role of AS160 in glucose uptake in skeletal muscle but suggests that in adipocytes, AS160 may not be the sole rate-limiting factor, or an effective compensatory mechanism exists (unlike in muscle). This highlights tissue-specific roles of AS160.	Signaling	Unclear risk (Not reported)	Low risk	Unclear risk (Not reported)	Unclear risk (Not reported)	Unclear risk (Not reported)	Unclear risk (Not reported)	Unclear risk (Not reported)	Unclear risk (Not reported)	Unclear risk (Not reported)	Unclear risk (Not reported)	Unclear risk (Not reported)	Low risk	Unclear risk (Not reported)	Unclear risk (Not reported)	Low risk	
A.781	10.1002/2211-5463.12528	Ji-Hee Kim, 2017.	Korea	To elucidate the physiological role of WNK1 in insulin-stimulated GLUT4 trafficking and glucose uptake in skeletal muscle.	L6 myotubes and Mouse skeletal muscle (ex vivo).	Stimulation: Insulin (100 nM).<br>Genetic: siRNA knockdown of WNK1.<br>Chemical: WNK463 (WNK1 inhibitor).	Function: 2-Deoxyglucose uptake assay.<br>Translocation: Surface biotinylation assay.<br>Signaling: Western blotting.	Stimulation: 20–30 minutes.<br>Knockdown: 48 hours.	Genetic: Control siRNA vs. WNK1 siRNA.<br>State: Vehicle vs. Insulin.<br>Chemical: Vehicle vs. WNK463.	Insulin stimulates WNK1 phosphorylation at T58 via the PI3K/Akt pathway in skeletal muscle cells. Knockdown of WNK1 reduces TBC1D4 phosphorylation and decreases cell surface GLUT4 abundance (both basal and insulin-stimulated). Loss-of-function WNK1 mutants cause similar effects. In type 2 diabetic (db/db) mice, WNK1 phosphorylation is significantly blunted, suggesting a role in pathogenesis..	WNK1 (With-No-Lysine [K] Kinase 1)	Stimulation	WNK1 is an essential component of the insulin signaling pathway in skeletal muscle. It acts downstream of Akt but independently of AS160 direct phosphorylation. WNK1 regulates the phosphorylation of TBC1D1 (an AS160-related Rab-GAP), thereby promoting GLUT4 translocation.	Signaling										Unclear risk (Not reported)	Unclear risk (Not reported)	Low risk	Unclear risk (Not reported)	Unclear risk (Not reported)	Low risk	
A.782	10.1074/jbc.M801750200	Ilana Talior-Volodarsky, 2008.	Canada	To determine the role of alpha-actinin-4 (ACTN4) in insulin-stimulated GLUT4 translocation, based on the prior observation of ACTN4 co-precipitating with GLUT4.	L6-GLUT4myc myoblasts.	Stimulation: Insulin (100 nM).<br>Genetic: siRNA knockdown of ACTN4.<br>Chemical: Latrunculin B (actin disruption).	Interaction: Mass spectrometry, Immunoprecipitation.<br>Translocation: Cell surface GLUT4myc detection (colorimetric/fluorescence), Confocal microscopy.	Stimulation: 20 minutes.<br>Knockdown: 48 hours.	Genetic: Control siRNA vs. ACTN4 siRNA.<br>State: Basal vs. Insulin.<br>Cargo: GLUT4 vs. Transferrin receptor (TfR).	Insulin stimulates a strong association between ACTN4 and GLUT4; this is specific to insulin (not PDGF) and PI3K-dependent. ACTN4 knockdown significantly inhibits insulin-stimulated glucose uptake and almost obliterates surface GLUT4 gain. This effect is selective for insulin; ACTN4 deficiency does not affect GLUT4 translocation induced by PDGF, K+ depolarization, or mitochondrial uncoupling. Without ACTN4, GLUT4 fails to co-localize with the insulin-triggered cortical actin mesh and remains trapped in the perinuclear region, unable to accumulate at the cell periphery.	ACTN4 (α-actinin-4)	Stimulation	ACTN4 serves as a linker between GLUT4 vesicles and the actin cytoskeleton. Insulin stimulates ACTN4 to bind GLUT4 vesicles, facilitating their positioning and anchoring at sites of cortical actin remodeling to prepare for fusion with the plasma membrane.	Translocation										Unclear risk (Not reported)	Unclear risk (Not reported)	Low risk	Unclear risk (Not reported)	Low risk	Low risk	
A.783	10.1074/jbc.M105364200	Takeshi Imamura, 2001.	USA	To determine the mechanism by which Endothelin-1 (ET-1) stimulates glucose transport in 3T3-L1 adipocytes, specifically the roles of beta-arrestin and Yes tyrosine kinase.	3T3-L1 adipocytes.	Stimulation: Endothelin-1 (100 nM), Insulin (100 nM).<br>Genetic: Overexpression of beta-arrestin 1 (WT and V53D dominant negative); Microinjection of anti-Yes, anti-Src antibodies; Expression of Yes kinase-dead (KR).<br>Chemical: Wortmannin (PI3K inhibitor), PP2 (Src inhibitor).	Function: 2-Deoxyglucose uptake assay.<br>Translocation: GLUT4 translocation assay.<br>Signaling: Immunoprecipitation & Western blotting.	30–45 minutes post-stimulation.	State: Basal vs. ET-1 vs. Insulin.<br>Injection: Control IgG vs. Anti-Yes.<br>Genetic: Vector vs. beta-arr1 V53D.	PP2 (Src inhibitor) blocks ET-1-induced glucose uptake and Gαq/11 phosphorylation but has no effect on insulin action, indicating distinct pathways. Among Src family kinases, only c-Yes is involved in ET-1-induced GLUT4 translocation (anti-c-Yes antibody blocks the effect, anti-c-Src/c-Fyn do not). Upon ET-1 binding to the ETA receptor, β-arrestin 1 is recruited to the receptor and acts as a scaffold to recruit c-Yes. Inhibiting β-arrestin 1 blocks ET-1 signaling but not insulin signaling.	Endothelin-1 (ET-1), Yes, beta-arrestin 1	Stimulation	Upon ET-1 binding to the ETA receptor, β-arrestin 1 is recruited to act as a scaffold, which in turn recruits and activates the kinase c-Yes. This initiates a PI3K/Akt signaling cascade that leads to GLUT4 translocation and glucose uptake, distinct from the canonical insulin receptor pathway.	Signaling										Unclear risk (Not reported)	Unclear risk (Not reported)	Low risk	Unclear risk (Not reported)	Low risk	Low risk	
A.785	10.1016/j.molmet.2020.101091	Stewart W.C. Masson, 2020.	New Zealand	To investigate the mechanism by which beta-catenin regulates glucose transport in skeletal muscle, specifically testing the hypothesis that beta-catenin acts downstream of Akt to regulate the actin remodeling required for GLUT4 translocation.	L6-GLUT4myc myoblasts; C57BL/6 mouse skeletal muscle (ex vivo).	Stimulation: Insulin (10–100 nM).<br>Genetic: siRNA knockdown of beta-catenin; Expression of stable beta-catenin mutant (S552A - Akt non-phosphorylatable); Knockdown of M-cadherin.<br>Chemical: BYL719 (PI3K/Akt inhibitor), Jasplakinolide (actin stabilizer).	Function: 2-Deoxyglucose uptake assay.<br>Translocation: Surface GLUT4myc assay.<br>Signaling/Interaction: Immunoblotting (Western blot), Immunoprecipitation.<br>Cytoskeleton: F-actin/G-actin ratio assay.	Stimulation: 20 minutes.<br>Knockdown: 48 hours post-transfection.	Genetic: Scrambled siRNA vs. beta-catenin siRNA; Wild-type (WT) vs. S552A mutant.<br>State: Basal vs. Insulin.<br>Chemical: Vehicle vs. Inhibitor.	Muscle-specific deletion of beta-catenin in mice (BCAT-mKO) significantly impaired insulin-stimulated glucose uptake in gastrocnemius and tibialis anterior muscles and compromised systemic blood glucose lowering. Insulin increases beta-catenin phosphorylation at Serine 552. This process is Akt- and PI3K-dependent but Rac1-independent. Phosphorylated beta-catenin (S552) binds to M-cadherin upon insulin stimulation. This interaction is essential for insulin to stimulate actin polymerization and glucose transport.	M-cadherin, Actin, Beta-catenin	Stimulation	Insulin activates Akt, which phosphorylates beta-catenin at Ser552. Phosphorylated beta-catenin enhances its binding to M-cadherin at the plasma membrane. This complex plays a critical role in promoting actin remodeling, creating the necessary cytoskeletal conditions for GLUT4 translocation and membrane insertion.	Signaling, Translocation	Unclear risk (Not reported)	Low risk	Unclear risk (Not reported)	Unclear risk (Not reported)	Unclear risk (Not reported)	Unclear risk (Not reported)	Unclear risk (Not reported)	Unclear risk (Not reported)	Unclear risk (Not reported)	Unclear risk (Not reported)	Unclear risk (Not reported)	Low risk	Unclear risk (Not reported)	Unclear risk (Not reported)	Low risk	
