# Supplementary material for: Molecular architecture of GLUT4 trafficking and exocytosis in adipocytes and skeletal muscle: A systematic review and integrated conceptual framework
Source: Front Endocrinol (Lausanne). 2026 Oct 19;17:1932455. doi: 10.3389/fendo.2026.1932455 (PMC13725575; doi:10.3389/fendo.2026.1932455)
Supplement: Supplementary file 2 [file Table2.pdf]

Table S2. Molecular mediators of GLUT4 exocytosis in adipocytes. Comprehensive list of molecules reported to participate in adipocyte GLUT4 trafficking, organized according to major trafficking phases including signaling, vesicle translocation, tethering, docking, and membrane fusion.

Phase	Primary molecule studied	Ref	Primary model system	Intervention class	Intervention tier
**Signaling**	**14-3-3 proteins**	**A.29**	• CHO IR/IRS-1 cells<br>• 3T3-L1 adipocytes	Genetic perturbation/rescue + Pharmacological perturbation + Physiological stimulus	Tier 1 - convergent mechanistic evidence
	**53BP2S**	**A.4**	• COS-7 cells (monkey kidney cells).<br>• 3T3-L1 adipocytes (mouse adipocytes).<br>• CHO-C400 cells (Chinese hamster ovary cells expressing the human insulin receptor).	Genetic perturbation/rescue + Pharmacological perturbation + Physiological stimulus	Tier 4 - descriptive/correlative evidence
	**Akt (PKB)**	**A.59**	• Mouse 3T3-L1 adipocytes	Genetic perturbation/rescue	Tier 2 - functional mechanistic evidence
		**A.74**	• Mouse 3T3-L1 adipocytes and HeLa cells.	Genetic perturbation/rescue + + Physiological stimulus	Tier 1 - convergent mechanistic evidence
		**A.148**	• 3T3-L1 adipocytes (differentiated from fibroblasts)	Genetic perturbation/rescue	Tier 2 - functional mechanistic evidence
		**A.256**	• Adipocytes differentiated from the mouse 3T3-L1 cell line.	Genetic perturbation/rescue	Tier 2 - functional mechanistic evidence
		**A.371**	3T3-L1 adipocytes	Genetic perturbation/rescue	Tier 2 - functional mechanistic evidence
		**A.501**	3T3-L1 adipocytes.	Disease/stress model	Tier 3 - pharmacological functional evidence
		**A.526**	Primary cultures of rat adipose cells.	Genetic perturbation/rescue	Tier 2 - functional mechanistic evidence
		**A.538**	Freshly isolated rat adipocytes and 3T3-L1 adipocytes.	Genetic perturbation/rescue	Tier 2 - functional mechanistic evidence

		A.543	3T3-L1 adipocytes.	Genetic perturbation/rescue	Tier 2 - functional mechanistic evidence
	Akt1	A.74	• Mouse 3T3-L1 adipocytes and HeLa cells.	Genetic perturbation/rescue + + Physiological stimulus	Tier 1 - convergent mechanistic evidence
		A.430	1. Primary adipocytes isolated from wild mice (WT) and Akt2-deficient mice.<br><br>2. Primary mouse embryonic fibroblasts (MEFs) and immortalized lines are differentiated into fat cells from genotypes: WT, Akt1 deficiency, Akt2 deficiency, and both.<br><br>3. Brown preadipocytes isolated from mice.	Genetic perturbation/rescue	Tier 2 - functional mechanistic evidence
		A.386	3T3-L1 adipocytes	Genetic perturbation/rescue	Tier 2 - functional mechanistic evidence
		A.361	3T3-L1 adipocytes	Genetic perturbation/rescue	Tier 2 - functional mechanistic evidence
		A.770	3T3-L1-GLUT4myc adipocytes and CHO-GLUT4myc cells.	Genetic perturbation/rescue	Tier 2 - functional mechanistic evidence
	Akt2	A.61	• Primary rat adipocytes	Pharmacological perturbation	Tier 3 - pharmacological functional evidence
		A.74	• Mouse 3T3-L1 adipocytes and HeLa cells.	Genetic perturbation/rescue + + Physiological stimulus	Tier 1 - convergent mechanistic evidence
		A.203	• 3T3-L1 adipocytes (differentiated from mouse fibroblasts).<br>• Mouse lung cancer cell line (KP1 lung cancer cells).	Genetic perturbation/rescue	Tier 2 - functional mechanistic evidence
		A.342	Adipocytes isolated from the testicular crestal fat cushion of male Sprague-Dawley rats.	Pharmacological perturbation	Tier 3 - pharmacological functional evidence
		A.386	3T3-L1 adipocytes	Genetic perturbation/rescue	Tier 2 - functional mechanistic evidence

		A.430	1. Primary adipocytes isolated from wild mice (WT) and Akt2-deficient mice.<br><br>2. Primary mouse embryonic fibroblasts (MEFs) and immortalized lines are differentiated into fat cells from genotypes: WT, Akt1 deficiency, Akt2 deficiency, and both.<br><br>3. Brown preadipocytes isolated from mice.	Genetic perturbation/rescue	Tier 2 - functional mechanistic evidence
		A.585	3T3-L1 adipocytes.	Pharmacological perturbation	Tier 3 - pharmacological functional evidence
		A.770	3T3-L1-GLUT4myc adipocytes and CHO-GLUT4myc cells.	Genetic perturbation/rescue	Tier 2 - functional mechanistic evidence
		A.361	3T3-L1 adipocytes	Genetic perturbation/rescue	Tier 2 - functional mechanistic evidence
	**Akt3**	A.770	3T3-L1-GLUT4myc adipocytes and CHO-GLUT4myc cells.	Genetic perturbation/rescue	Tier 2 - functional mechanistic evidence
	**AMPK**	A.1	3T3-L1 adipocytes	Pharmacological perturbation	Tier 3 - pharmacological functional evidence
	**CAP**	A.85	• Differentiated mouse 3T3-L1 adipocytes<br>• CHO cells overexpressing the human insulin receptor (CHO-IR)	Genetic perturbation/rescue	Tier 2 - functional mechanistic evidence
	**Crk**	A.85	• Differentiated mouse 3T3-L1 adipocytes<br>• CHO cells overexpressing the human insulin receptor (CHO-IR)	Genetic perturbation/rescue	Tier 2 - functional mechanistic evidence
	**Flotillin**	A.85	• Differentiated mouse 3T3-L1 adipocytes<br>• CHO cells overexpressing the human insulin receptor (CHO-IR)	Genetic perturbation/rescue	Tier 2 - functional mechanistic evidence
	**C3G**	A.85	• Differentiated mouse 3T3-L1 adipocytes<br>• CHO cells overexpressing the human insulin receptor (CHO-IR)	Genetic perturbation/rescue	Tier 2 - functional mechanistic evidence
	**APS**	A.85	• Differentiated mouse 3T3-L1 adipocytes<br>• CHO cells overexpressing the human insulin receptor (CHO-IR)	Genetic perturbation/rescue	Tier 2 - functional mechanistic evidence
	**ARF6**	A.290	Fat cells differentiate from the 3T3-L1 line (3T3-L1 adipocytes).	Genetic perturbation/rescue	Tier 2 - functional mechanistic evidence

		**A.114**	• Mouse 3T3-L1 adipocytes	Genetic perturbation/rescue	Tier 2 - functional mechanistic evidence
		**A.148**	• 3T3-L1 adipocytes (differentiated from fibroblasts)	Genetic perturbation/rescue	Tier 2 - functional mechanistic evidence
		**A.164**	• Cell-based reconstitution model: 3T3-L1 fibroblasts ectopically expressing Myc-GLUT4, HA-sortilin, and AS160/Tbc1d1.<br><br>• Differentiated 3T3-L1 adipocytes.	Genetic perturbation/rescue	Tier 2 - functional mechanistic evidence
		**A.176**	• AS160 whole-body knockout mice (AS160(-/-)) and primary tissues/cells isolated from them: white adipocytes (from epididymal/periuterine fat), soleus muscle, extensor digitorum longus (EDL), gastrocnemius muscle, and quadriceps muscle.	Genetic perturbation/rescue	Tier 2 - functional mechanistic evidence
		**A.254**	• Adipocytes differentiated from the 3T3-L1 cell line.	Genetic perturbation/rescue + Pharmacological perturbation + Physiological stimulus	Tier 1 - convergent mechanistic evidence
		**A.371**	3T3-L1 adipocytes	Genetic perturbation/rescue	Tier 2 - functional mechanistic evidence
	**AS160 (TBC1D4)**	**A.386**	3T3-L1 adipocytes	Genetic perturbation/rescue	Tier 2 - functional mechanistic evidence
		**A.390**	3T3-L1 cells (in the form of undifferentiated fibroblasts and differentiated fat cells).	Genetic perturbation/rescue	Tier 2 - functional mechanistic evidence
		**A.407**	Adipocytes of the 3T3-L1 lineage.	Genetic perturbation/rescue	Tier 2 - functional mechanistic evidence
		**A.448**	3T3-L1 fat cells express HA-Glut4/GFP protein	Genetic perturbation/rescue	Tier 2 - functional mechanistic evidence
		**A.589**	3T3-L1 adipocytes.	Genetic perturbation/rescue	Tier 2 - functional mechanistic evidence
		**A.625**	3T3-L1 adipocytes.	Genetic perturbation/rescue + Physiological stimulus	Tier 1 - convergent mechanistic evidence
		**A.722**	Differentiated 3T3-L1 adipocytes, HEK293E cells, CHO IR/IRS-1 cells.	Genetic perturbation/rescue	Tier 2 - functional mechanistic evidence

		A.780	AS160 knockout Wistar rats (AS160-KO rats) and Wild-type (WT) littermates. Isolated tissues: Soleus muscle, Epitrochlearis muscle, Epididymal adipose tissue.	Genetic perturbation/rescue	Tier 2 - functional mechanistic evidence
	**ASIP**	A.328	3T3-L1 fat cells	Disease/stress model	Tier 2 - functional mechanistic evidence
	**beta-arrestin 1**	A.783	3T3-L1 adipocytes.	Genetic perturbation/rescue + Pharmacological perturbation + Physiological stimulus	Tier 1 - convergent mechanistic evidence
	**c-Cbl**	A.83	• Fully differentiated mouse 3T3-L1 adipocytes	Genetic perturbation/rescue	Tier 2 - functional mechanistic evidence
	**c-Cbl**	A.502	Differentiated 3T3-L1 adipocytes.	Disease/stress model	Tier 2 - functional mechanistic evidence
	**c-Cbl**	A.732	Differentiated 3T3-L1 adipocytes and CHO-IR cells.	Genetic perturbation/rescue	Tier 2 - functional mechanistic evidence
	**Ca2+/calmodulin**	A.321	Mouse 3T3-F442A adipocytes (3T3-F442A murine adipocytes).	Pharmacological perturbation	Tier 3 - pharmacological functional evidence
	**CADTK (Pyk2)**	A.502	Differentiated 3T3-L1 adipocytes.	Disease/stress model	Tier 2 - functional mechanistic evidence
	**Cbl Protein**	A.605	3T3-L1 adipocytes.	Genetic perturbation/rescue	Tier 2 - functional mechanistic evidence
	**Cbl-b**	A.732	Differentiated 3T3-L1 adipocytes and CHO-IR cells.	Genetic perturbation/rescue	Tier 2 - functional mechanistic evidence
	**Cdc42**	A.127	• Differentiated 3T3-L1 adipocytes	Genetic perturbation/rescue	Tier 2 - functional mechanistic evidence
	**ClipR-59**	A.688	Mouse 3T3-L1 adipocytes and Human Embryonic Kidney (HEK293) cells.	Genetic perturbation/rescue + Pharmacological perturbation + Physiological stimulus	Tier 1 - convergent mechanistic evidence
	**DGK-ζ**	A.16	• 3T3-L1 adipocytes	Genetic perturbation/rescue + Physiological stimulus	Tier 1 - convergent mechanistic evidence
	**E4orf1**	A.220	3T3-L1 preadipocytes engineered for inducible expression of E4orf1 or empty vector control (Null Vector, NV).	Genetic perturbation/rescue	Tier 2 - functional mechanistic evidence

	**Endothelin-1 (ET-1)**	**A.783**	3T3-L1 adipocytes.	Genetic perturbation/rescue + Pharmacological perturbation + Physiological stimulus	Tier 1 - convergent mechanistic evidence
	**FLJ00068**	**A.709**	In vitro differentiated 3T3-L1 adipocytes and L1-GLUT4 cell line (3T3-L1 stably expressing myc7-GFP-GLUT4).	Genetic perturbation/rescue	Tier 2 - functional mechanistic evidence
	**Gapex-5**	**A.267**	Fat cells differentiate from 3T3-L1 (3T3-L1 adipocytes) and Cos-1 cells (used for protein interaction experiments).	Genetic perturbation/rescue + Physiological stimulus	Tier 1 - convergent mechanistic evidence
	**Glucose**	**A.271**	Rat adipocytes and Rat skeletal muscles: soleus muscles and extensor digitorum longus (EDL).	Genetic perturbation/rescue	Tier 2 - functional mechanistic evidence
		**A.295**	Primary cultured rat adipocytes.	Physiological stimulus	Tier 4 - descriptive/correlative evidence
	**GLUT4**	**A.241**	• Immortalized brown adipocyte cell lines isolated from brown adipose tissue of 3-day-old neonatal mice with three genotypes:<br>– Wild-type (IRS-2+/+)<br>– Heterozygous (IRS-2+/−)<br>– IRS-2–deficient (IRS-2−/−)<br>• Primary brown adipocytes were also used for validation.	Genetic perturbation/rescue + Physiological stimulus	Tier 1 - convergent mechanistic evidence
	**Gqi**	**A.264**	Fat cells differentiated from the 3T3-L1 lineage (3T3-L1 adipocytes) and Chinese hamster ovarian cells (CHO cells). Specifically, transgenic cell lines expressing c-MYC-tagged GLUT4 (GLUT4myc) and target receptors (PAFR, α1-AR, EP3, EP4).	Pharmacological perturbation	Tier 3 - pharmacological functional evidence
	**GRK2**	**A.287**	3T3-L1 fat cells (3T3-L1 adipocytes).	Genetic perturbation/rescue + Pharmacological perturbation + Physiological stimulus	Tier 1 - convergent mechanistic evidence
	**GTPγS**	**A.289**	Differentiated 3T3-L1 adipocytes are perforated by Streptolysin-O (SL-O permeabilized).	Pharmacological perturbation	Tier 3 - pharmacological functional evidence
	**Gα11**	**A.290**	Fat cells differentiate from the 3T3-L1 line (3T3-L1 adipocytes).	Genetic perturbation/rescue	Tier 2 - functional mechanistic evidence
		**A.742**	Differentiated 3T3-L1 adipocytes.	Genetic perturbation/rescue	Tier 2 - functional mechanistic evidence

	**Gαi2**	**A.291**	Transgenic mice (iQ205L) express the activated Gαi2(Q205L) mutation in adipose tissue, skeletal muscle and liver; along with wild-type littermates.	Genetic perturbation/rescue	Tier 2 - functional mechanistic evidence
	**Gαq**	**A.262**	3T3-L1 adipocytes	Genetic perturbation/rescue	Tier 2 - functional mechanistic evidence
		**A.742**	Differentiated 3T3-L1 adipocytes.	Genetic perturbation/rescue	Tier 2 - functional mechanistic evidence
	**Insulin Receptor Substrate-1 (IRS-1)**	**A.616**	Primary adipocytes.	Pharmacological perturbation	Tier 3 - pharmacological functional evidence
		**A.244**	• 3T3-L1 adipocytes.	Pharmacological perturbation	Tier 3 - pharmacological functional evidence
		**A.348**	Adipose cells isolated from epididymal fat pads of male rats, were primarily cultured and transient transfected with the epitope-attached GLUT4 encoding gene (GLUT4-HA)	Genetic perturbation/rescue	Tier 2 - functional mechanistic evidence
		**A.385**	1. 3T3-L1 adipocytes<br><br>2. Extra: rat adipocytes (Rat adipocytes)	Pharmacological perturbation	Tier 3 - pharmacological functional evidence
		**A.762**	Isolated rat adipose cells (Primary adipocytes).	Genetic perturbation/rescue	Tier 2 - functional mechanistic evidence
	**IRS-2**	**A.240**	• Brown adipocytes differentiated from preadipocytes isolated from neonatal wild-type (WT) mice and IRS-2 knockout (IRS-2 KO) mice	Genetic perturbation/rescue + Pharmacological perturbation + Physiological stimulus	Tier 1 - convergent mechanistic evidence
		**A.349**	1. Primary subject: Adipose cells isolated from epididymal fat pads of male rats, primary cultured and transient transfection with the epitope-binding GLUT4 encoding gene (GLUT4-HA)<br><br>2. Secondary subject: Freshly isolated rat adipose cells for precipitation immunoassay	Genetic perturbation/rescue	Tier 2 - functional mechanistic evidence

		A.241	• Immortalized brown adipocyte cell lines isolated from brown adipose tissue of 3-day-old neonatal mice with three genotypes:<br>– Wild-type (IRS-2+/+)<br>– Heterozygous (IRS-2+/−)<br>– IRS-2–deficient (IRS-2−/−)<br>• Primary brown adipocytes were also used for validation.	Genetic perturbation/rescue + Physiological stimulus	Tier 1 - convergent mechanistic evidence
	**LRP1**	A.390	3T3-L1 cells (in the form of undifferentiated fibroblasts and differentiated fat cells).	Genetic perturbation/rescue	Tier 2 - functional mechanistic evidence
	**miR-130a-3p**	A.292	FVB series mice (including WT wild mice, 130KO knockout mice, 130OE overexpress mice) and 3T3-L1 fat cell line.	Disease/stress model	Tier 2 - functional mechanistic evidence
	**MLCK**	A.321	Mouse 3T3-F442A adipocytes (3T3-F442A murine adipocytes).	Pharmacological perturbation	Tier 3 - pharmacological functional evidence
	**Myotubularin (MTM1)**	A.251	• Muscle cell lines: L6 myotubes, C2C12 myotubes.<br>• Adipocyte cell lines: 3T3-L1 adipocytes (specifically the CAR"delta"1 line).	Genetic perturbation/rescue	Tier 2 - functional mechanistic evidence
	**NCS-1**	A.490	Fully differentiated 3T3-L1 adipocytes	Genetic perturbation/rescue	Tier 2 - functional mechanistic evidence
	**Nexilin**	A.493	L6 rat skeletal muscle cells (myotubes) and 3T3-L1 mouse adipocytes.	Genetic perturbation/rescue	Tier 2 - functional mechanistic evidence
	**Norepinephrine**	A.264	Fat cells differentiated from the 3T3-L1 lineage (3T3-L1 adipocytes) and Chinese hamster ovarian cells (CHO cells). Specifically, transgenic cell lines expressing c-MYC-tagged GLUT4 (GLUT4myc) and target receptors (PAFR, α1-AR, EP3, EP4).	Pharmacological perturbation	Tier 3 - pharmacological functional evidence
	**P-Rex1**	A.308	3T3-L1 adipocytes	Genetic perturbation/rescue + Pharmacological perturbation + Physiological stimulus	Tier 1 - convergent mechanistic evidence
	**p130Cas**	A.502	Differentiated 3T3-L1 adipocytes.	Disease/stress model	Tier 2 - functional mechanistic evidence

	PAF	A.264	Fat cells differentiated from the 3T3-L1 lineage (3T3-L1 adipocytes) and Chinese hamster ovarian cells (CHO cells). Specifically, transgenic cell lines expressing c-MYC-tagged GLUT4 (GLUT4myc) and target receptors (PAFR, α1-AR, EP3, EP4).	Pharmacological perturbation	Tier 3 - pharmacological functional evidence
	PDK1	A.537	Freshly isolated rat adipocytes.	Genetic perturbation/rescue	Tier 2 - functional mechanistic evidence
	PFKFB3	A.436	1. HA-GLUT4-HeLa stable expression HeLa cells (HA-GLUT4-HeLa cells) used for large-area screening.<br><br>2. 3T3-L1 mouse adipocytes (3T3-L1 adipocytes) used to verify the mechanism.<br><br>3. HEK293 cells used for overexpression experiments.	Genetic perturbation/rescue	Tier 2 - functional mechanistic evidence
	PHLDB1	A.19	• Mouse 3T3-L1 adipocyte	Genetic perturbation/rescue	Tier 2 - functional mechanistic evidence
	Phospholipase C-γ	A.534	Primary brown adipocytes from rats and HeLa cells (expressing GLUT4-GFP).	Genetic perturbation/rescue	Tier 2 - functional mechanistic evidence
		A.101	• Differentiated 3T3-L1 adipocytes<br>• Primary mouse adipocytes	Pharmacological perturbation	Tier 3 - pharmacological functional evidence
	PI(3)K	A.405	Adipocytes of the 3T3-L1 lineage.	Genetic perturbation/rescue	Tier 2 - functional mechanistic evidence
		A.517	3T3-L1 adipocytes.	Pharmacological perturbation	Tier 3 - pharmacological functional evidence
		A.602	3T3-L1 adipocytes.	Genetic perturbation/rescue	Tier 2 - functional mechanistic evidence
		A.385	1. 3T3-L1 adipocytes<br><br>2. Extra: rat adipocytes (Rat adipocytes)	Pharmacological perturbation	Tier 3 - pharmacological functional evidence
		A.403	Adipocytes of the 3T3-L1 lineage.	Genetic perturbation/rescue	Tier 2 - functional mechanistic evidence
		A.621	Isolated rat adipose cells.	Genetic perturbation/rescue	Tier 2 - functional mechanistic evidence

		A.673	Differentiated 3T3-L1 adipocytes.	Genetic perturbation/rescue + Physiological stimulus	Tier 2 - functional mechanistic evidence
	**Phosphatidylinositol 3-kinase catalytic subunit p110α (p110α PI 3-kinase)**	A.503	3T3-L1 adipocytes.	Genetic perturbation/rescue	Tier 2 - functional mechanistic evidence
		A.508	3T3-L1 adipocytes differentiated from fibroblasts.	Genetic perturbation/rescue	Tier 2 - functional mechanistic evidence
	**PI3K p110β**	A.508	3T3-L1 adipocytes differentiated from fibroblasts.	Genetic perturbation/rescue	Tier 2 - functional mechanistic evidence
	**PI4K**	A.490	Fully differentiated 3T3-L1 adipocytes	Genetic perturbation/rescue	Tier 2 - functional mechanistic evidence
	**Pikfyve**	A.478	Animal: Mouse (C57BL/6J background). Specifically, MPIfKO (Muscle-specific PIKfyve Knockout) mice and control mice.<br>Tissue: Isolated rhabdomyoplasty (soleus muscle, long toe extensor muscle - EDL, lower leg twin muscle - gastrocnemius) and adipose tissue.	Disease/stress model	Tier 2 - functional mechanistic evidence
		A.88	• Mouse 3T3-L1 adipocytes	Disease/stress model	Tier 2 - functional mechanistic evidence
	**ArPIKfyve**	A.88	• Mouse 3T3-L1 adipocytes	Disease/stress model	Tier 2 - functional mechanistic evidence
	**PIP3**	A.528	3T3-L1 adipocytes.	Genetic perturbation/rescue	Tier 2 - functional mechanistic evidence
	**PKC-ζ**	A.227	• Primary adipocytes isolated from epididymal fat pads of male Sprague–Dawley rats.	Genetic perturbation/rescue	Tier 2 - functional mechanistic evidence
		A.150	Adipocytes and hepatocytes isolated from male Sprague–Dawley rats.	Physiological stimulus	Tier 4 - descriptive/correlative evidence
		A.335	3T3-L1 adipocytes	Genetic perturbation/rescue	Tier 2 - functional mechanistic evidence
		A.339	rat adipocytes and L6 myotubes	Genetic perturbation/rescue	Tier 2 - functional mechanistic evidence

		A.546	Freshly isolated rat adipocytes.	Genetic perturbation/rescue	Tier 2 - functional mechanistic evidence
		A.534	Primary brown adipocytes from rats and HeLa cells (expressing GLUT4-GFP).	Genetic perturbation/rescue	Tier 2 - functional mechanistic evidence
		A.241	• Immortalized brown adipocyte cell lines isolated from brown adipose tissue of 3-day-old neonatal mice with three genotypes:<br>– Wild-type (IRS-2+/+)<br>– Heterozygous (IRS-2+/−)<br>– IRS-2–deficient (IRS-2−/−)<br>• Primary brown adipocytes were also used for validation.	Genetic perturbation/rescue + Physiological stimulus	Tier 1 - convergent mechanistic evidence
	**PKCλ**	A.227	• Primary adipocytes isolated from epididymal fat pads of male Sprague–Dawley rats.	Genetic perturbation/rescue	Tier 2 - functional mechanistic evidence
		A.335	3T3-L1 adipocytes	Genetic perturbation/rescue	Tier 2 - functional mechanistic evidence
	**PLC-γ1**	A.334	3T3-L1 adipocytes	Pharmacological perturbation	Tier 3 - pharmacological functional evidence
	**pp60Src**	A.540	A431 human epidermoid carcinoma cells (expressing β2 AR-GFP or GLUT4-GFP), 3T3-L1, and 3T3-F442A mouse adipocytes.	Genetic perturbation/rescue	Tier 2 - functional mechanistic evidence
	**Protein Phosphatase-2C-α (PP2C-α)**	A.554	3T3-L1 adipocytes.	Genetic perturbation/rescue	Tier 2 - functional mechanistic evidence
	**PtdIns-3-P**	A.344	Skeletal muscle cells of the L6 line and fat cells of the 3T3-L1 line.	Genetic perturbation/rescue	Tier 2 - functional mechanistic evidence
		A.251	• Muscle cell lines: L6 myotubes, C2C12 myotubes.<br>• Adipocyte cell lines: 3T3-L1 adipocytes (specifically the CAR"delta"1 line).	Genetic perturbation/rescue	Tier 2 - functional mechanistic evidence
	**PTEN (Phosphatase and tensin homolog)**	A.743	Differentiated 3T3-L1 adipocytes.	Genetic perturbation/rescue	Tier 2 - functional mechanistic evidence
	**PTP1B (Protein-tyrosine phosphatase 1B)**	A.555	Primary cultured rat adipocytes.	Genetic perturbation/rescue	Tier 2 - functional mechanistic evidence
	**Rab31**	A.267	Fat cells differentiate from 3T3-L1 (3T3-L1 adipocytes) and Cos-1 cells (used for protein interaction experiments).	Genetic perturbation/rescue + Physiological stimulus	Tier 1 - convergent mechanistic evidence

	Rac1	A.8	• L1-GLUT4 cell line (a stable cell line expressing GLUT4myc7-GFP) differentiated from 3T3-L1 fibroblasts	Genetic perturbation/rescue + Pharmacological perturbation + Physiological stimulus	Tier 1 - convergent mechanistic evidence
		A.106	• Adipocyte-specific Rac1 knockout mice (adipo-rac1-KO) on a C57BL/6 genetic background<br>• Primary cultured mature white adipocytes isolated from subcutaneous white adipose tissue (sWAT)	Genetic perturbation/rescue + Pharmacological perturbation + Physiological stimulus	Tier 1 - convergent mechanistic evidence
	Ras	A.403	Adipocytes of the 3T3-L1 lineage.	Genetic perturbation/rescue	Tier 2 - functional mechanistic evidence
		A.621	Isolated rat adipose cells.	Genetic perturbation/rescue	Tier 2 - functional mechanistic evidence
	RhoA	A.290	Fat cells differentiate from the 3T3-L1 line (3T3-L1 adipocytes).	Genetic perturbation/rescue	Tier 2 - functional mechanistic evidence
	RIP140	A.12	• 3T3-L1 adipocytes (differentiated for 4 days and 8 days).<br>• G7 adipocytes (stably expressing myc-GLUT4-EGFP).<br>• Primary adipocytes from the epididymal fat pads of male mice (epididymal adipocytes).<br>• RIP140-null adipocytes.	Genetic perturbation/rescue + Disease/stress model + Physiological stimulus	Tier 1 - convergent mechanistic evidence
	RUVBL2	A.625	3T3-L1 adipocytes.	Genetic perturbation/rescue + Physiological stimulus	Tier 1 - convergent mechanistic evidence
	SHIP2	A.505	3T3-L1 adipocytes.	Genetic perturbation/rescue	Tier 2 - functional mechanistic evidence
	SNAREs	A.543	3T3-L1 adipocytes.	Genetic perturbation/rescue	Tier 2 - functional mechanistic evidence
	STEAP4	A.441	Human preadipocytes are cultured and differentiated into adult fat cells.	Genetic perturbation/rescue	Tier 2 - functional mechanistic evidence
	Tbc1d1	A.164	• Cell-based reconstitution model: 3T3-L1 fibroblasts ectopically expressing Myc-GLUT4, HA-sortilin, and AS160/Tbc1d1.<br><br>• Differentiated 3T3-L1 adipocytes.	Genetic perturbation/rescue	Tier 2 - functional mechanistic evidence
		A.327	3T3-L1 adipocytes, L6 muscle cells (L6 cells), and tissues from wild rats or AS160 knockout mice.	Genetic perturbation/rescue + Pharmacological perturbation + Physiological stimulus	Tier 1 - convergent mechanistic evidence

		A.408	1. C2C1 muscle cells.<br>2. 3T3-L1 fat cells.<br>3. Human muscle biopsy samples: Taken from healthy people and type 2 diabetics.	Disease/stress model	Tier 2 - functional mechanistic evidence
		A.654	3T3-L1 adipocytes.	Genetic perturbation/rescue + Pharmacological perturbation + Physiological stimulus	Tier 1 - convergent mechanistic evidence
	**TC10**	A.85	• Differentiated mouse 3T3-L1 adipocytes<br>• CHO cells overexpressing the human insulin receptor (CHO-IR)	Genetic perturbation/rescue	Tier 2 - functional mechanistic evidence
		A.128	• Differentiated 3T3-L1 adipocytes	Genetic perturbation/rescue + Pharmacological perturbation + Physiological stimulus	Tier 1 - convergent mechanistic evidence
		A.405	Adipocytes of the 3T3-L1 lineage.	Genetic perturbation/rescue	Tier 2 - functional mechanistic evidence
	**TC10a**	A.679	Differentiated 3T3L1 adipocytes.	Genetic perturbation/rescue	Tier 2 - functional mechanistic evidence
	**TCGAP (TC10/cdc42 GTPase activating protein)**	A.680	Mouse 3T3-L1 adipocytes, Cos-1, HEK293T, and CHO/IR cells.	Genetic perturbation/rescue	Tier 2 - functional mechanistic evidence
	**Tf receptor**	A.390	3T3-L1 cells (in the form of undifferentiated fibroblasts and differentiated fat cells).	Genetic perturbation/rescue	Tier 2 - functional mechanistic evidence
	**Yes**	A.783	3T3-L1 adipocytes.	Genetic perturbation/rescue + Pharmacological perturbation + Physiological stimulus	Tier 1 - convergent mechanistic evidence
**Translocation**	**Actin**	A.250	• Adipocytes differentiated from the 3T3-L1 fibroblast cell line	Genetic perturbation/rescue	Tier 2 - functional mechanistic evidence
		A.365	3T3-L1 adipocytes	Genetic perturbation/rescue	Tier 2 - functional mechanistic evidence
	**Adipsin**	A.57	• Mouse 3T3-L1 adipocytes	Pharmacological perturbation	Tier 3 - pharmacological functional evidence
	**Akt (PKB)**	A.250	• Adipocytes differentiated from the 3T3-L1 fibroblast cell line	Genetic perturbation/rescue	Tier 2 - functional mechanistic evidence

	**Amino acids**	**A.394**	1. 3T3-L1 cells (differentiated fibroblasts and fat cells)<br><br>2. Other: CHO-K1 cells, NIH 3T3 cells, AML12 liver cell line	Pharmacological perturbation	Tier 3 - pharmacological functional evidence
	**AS160 (TBC1D4)**	**A.96**	• 3T3-L1 adipocytes<br>• HEK-293F cells<br>• Escherichia coli (strains BL21, Rosetta BL21, XL-1 Blue)	Pharmacological perturbation	Tier 3 - pharmacological functional evidence
		**A.387**	3T3-L1 adipocytes	Genetic perturbation/rescue	Tier 2 - functional mechanistic evidence
		**A.389**	3T3-L1 adipocytes	Genetic perturbation/rescue + Physiological stimulus	Tier 1 - convergent mechanistic evidence
		**A.457**	_ AS160 Thr649Ala knockin mice<br>- epididymal fat pads<br>- Primary adipose cells and isolated soleus muscles	Physiological stimulus	Tier 4 - descriptive/correlative evidence
	**Axin**	**A.689**	Differentiated 3T3-L1 adipocytes and HEK293T cells.	Genetic perturbation/rescue + Pharmacological perturbation + Disease/stress model + Physiological stimulus	Tier 1 - convergent mechanistic evidence
	**Calpain-10 (CAPN10)**	**A.116**	• Differentiated mouse 3T3-L1 adipocytes	Genetic perturbation/rescue	Tier 2 - functional mechanistic evidence
	**CBP (Lysine acetyltransferases)**	**A.510**	Animals: Skeletal muscle-specific p300/CBP knockout mice (PCKO mice) and related compound heterozygous lines.<br><br>Cells: L6 myoblasts/myotubes, 3T3-L1 adipocytes, and primary human skeletal muscle cells.	Genetic perturbation/rescue	Tier 2 - functional mechanistic evidence
	**CD-MPR**	**A.351**	Adipocytes isolated from epididymal fat pads of male Ola (SD-like)	Physiological stimulus	Tier 4 - descriptive/correlative evidence
	**Sortilin**	**A.67**	• Mouse 3T3-L1 adipocytes and HeLa cells.	Genetic perturbation/rescue	Tier 2 - functional mechanistic evidence
	**Cellugyrin (Synaptogyrin-2)**	**A.67**	• Mouse 3T3-L1 adipocytes and HeLa cells.	Genetic perturbation/rescue	Tier 2 - functional mechanistic evidence
	**CLASP2**	**A.303**	3T3-L1 adipocytes	Genetic perturbation/rescue	Tier 2 - functional mechanistic evidence
	**Actin**	**A.250**	• Adipocytes differentiated from the 3T3-L1 fibroblast cell line	Genetic perturbation/rescue	Tier 2 - functional mechanistic evidence

		A.365	3T3-L1 adipocytes	Genetic perturbation/rescue	Tier 2 - functional mechanistic evidence
		A.31	Mouse 3T3-L1 adipocyte	Genetic perturbation/rescue + Pharmacological perturbation	Tier 2 - functional mechanistic evidence
		A.402	Adipocytes of the 3T3-L1 lineage.	Genetic perturbation/rescue	Tier 2 - functional mechanistic evidence
	**Dennd4C**	A.401	1. Adipocytes (adipocytes) of the 3T3-L1 line<br><br>2. HEK293 cells.	Genetic perturbation/rescue + Physiological stimulus	Tier 1 - convergent mechanistic evidence
	**EHD2**	A.230	The study aimed to identify previously uncharacterized regulatory proteins associated with insulin-responsive GLUT4 vesicles (IRGTVs) and to elucidate their role in insulin-stimulated recruitment of GLUT4 to the plasma membrane.	Genetic perturbation/rescue	Tier 2 - functional mechanistic evidence
	**EXOC6**	A.24	• Mouse 3T3-L1 adipocyte	Genetic perturbation/rescue + Physiological stimulus	Tier 1 - convergent mechanistic evidence
	**EXOC6B**	A.24	• Mouse 3T3-L1 adipocyte	Genetic perturbation/rescue + Physiological stimulus	Tier 1 - convergent mechanistic evidence
	**FAK (pY397)**	A.253	• Primary adipocytes isolated from subcutaneous or epididymal adipose tissue of male C57BL/6J mice.<br>• Primary adipocytes isolated from human abdominal subcutaneous adipose tissue.	Pharmacological perturbation	Tier 3 - pharmacological functional evidence
	**GDI-1**	A.268	Fat cells differentiate from 3T3-L1 adipocytes and human embryonic kidney cells (HEK-293 cells).	Genetic perturbation/rescue	Tier 2 - functional mechanistic evidence
	**GDI-2**	A.268	Fat cells differentiate from 3T3-L1 adipocytes and human embryonic kidney cells (HEK-293 cells).	Genetic perturbation/rescue	Tier 2 - functional mechanistic evidence
	**GDP dissociation inhibitor (GDI)**	A.99	• Differentiated 3T3-L1 adipocytes<br>• COS-1 cells<br>• CHO-T cells	Physiological stimulus	Tier 4 - descriptive/correlative evidence
		A.225	• Adipocytes differentiated from the 3T3-L1 fibroblast cell line.	Physiological stimulus	Tier 4 - descriptive/correlative evidence

GLUT4

		A.279	Fat cells differentiate from the 3T3-L1 lineage (3T3-L1 adipocytes), CHO-IR cells (human insulin receptor-expressing hamster ovarian cells), and HEK293 cells.	Pharmacological perturbation	Tier 3 - pharmacological functional evidence
		A.304	3T3-L1 adipocytes	Genetic perturbation/rescue	Tier 2 - functional mechanistic evidence
		A.306	Fat cells differentiated from the 3T3-L1 fibroblast line of mice.	Genetic perturbation/rescue	Tier 2 - functional mechanistic evidence
	**IRAP**	A.351	Adipocytes isolated from epididymal fat pads of male Ola (SD-like)	Physiological stimulus	Tier 4 - descriptive/correlative evidence
		A.389	3T3-L1 adipocytes	Genetic perturbation/rescue + Physiological stimulus	Tier 1 - convergent mechanistic evidence
		A.398	3T3-L1 adipocytes	Disease/stress model	Tier 2 - functional mechanistic evidence
	**IRS-3**	A.45	• Primary rat adipocytes	Genetic perturbation/rescue	Tier 2 - functional mechanistic evidence
	**IRS-4**	A.45	• Primary rat adipocytes	Genetic perturbation/rescue	Tier 2 - functional mechanistic evidence
	**Kif13A**	A.10	• Mouse 3T3-L1 adipocytes (differentiated 3T3-L1 adipocytes) and human SGBS adipocytes.<br>• In vitro model: adipocytes differentiated from fibroblasts	Genetic perturbation/rescue + Pharmacological perturbation + Disease/stress model + Physiological stimulus	Tier 1 - convergent mechanistic evidence
	**KIF3**	A.381	3T3-L1 adipocytes	Genetic perturbation/rescue + Pharmacological perturbation + Physiological stimulus	Tier 1 - convergent mechanistic evidence
	**KIF3A**	A.689	Differentiated 3T3-L1 adipocytes and HEK293T cells.	Genetic perturbation/rescue + Pharmacological perturbation + Disease/stress model + Physiological stimulus	Tier 1 - convergent mechanistic evidence
	**Microtubules**	A.31	Mouse 3T3-L1 adipocyte	Genetic perturbation/rescue + Pharmacological perturbation	Tier 2 - functional mechanistic evidence
		A.384	3T3-L1 adipocytes	Pharmacological perturbation	Tier 3 - pharmacological functional evidence

		A.25	Mouse 3T3-L1 adipocyte	Pharmacological perturbation	Tier 3 - pharmacological functional evidence
		A.350	Isolated adipocytes from epididymal adipose tissues of Sprague-Dawley rats	Pharmacological perturbation	Tier 3 - pharmacological functional evidence
		A.416	Adipocytes of the 3T3-L1 lineage.	Genetic perturbation/rescue	Tier 2 - functional mechanistic evidence
		A.197	Differentiated 3T3-L1 adipocytes.	Genetic perturbation/rescue	Tier 2 - functional mechanistic evidence
	**Myo1c (Myosin 1c)**	A.118	• 3T3-L1 adipocytes<br>• CHO cells expressing insulin receptor and IRS-1 (CHO/IR/IRS-1 cells)	Genetic perturbation/rescue + Pharmacological perturbation + Physiological stimulus	Tier 1 - convergent mechanistic evidence
	**Myosin 5a**	A.481	Differentiated 3T3-L1 adipocytes.	Genetic perturbation/rescue + Pharmacological perturbation + Physiological stimulus	Tier 1 - convergent mechanistic evidence
	**N4bp2l1**	A.487	• Animal: Male rat C57BL/6 (8 weeks old).<br>• Cells: 3T3-L1 fat cells (differentiated from pre-fat cells) and HEK293T cells.	Disease/stress model	Tier 2 - functional mechanistic evidence
	**NAALADase**	A.485	Adipocytes were isolated from male rats of the Sprague–Dawley strain.	Pharmacological perturbation	Tier 3 - pharmacological functional evidence
	**p115 N-terminal fragment overexpression**	A.509	3T3-L1 adipocytes and COS7 cells.	Genetic perturbation/rescue	Tier 2 - functional mechanistic evidence
	**p300**	A.510	Animals: Skeletal muscle-specific p300/CBP knockout mice (PCKO mice) and related compound heterozygous lines.<br><br>Cells: L6 myoblasts/myotubes, 3T3-L1 adipocytes, and primary human skeletal muscle cells.	Genetic perturbation/rescue	Tier 2 - functional mechanistic evidence
	**Phosphorylated Tbc1d1**	A.599	3T3-L1 fibroblasts.	Genetic perturbation/rescue	Tier 2 - functional mechanistic evidence
	**PI3K**	A.46	• Mouse 3T3-L1 adipocyte	Genetic perturbation/rescue	Tier 2 - functional mechanistic evidence
		A.53	• Mouse 3T3-L1 adipocytes	Pharmacological perturbation	Tier 3 - pharmacological functional evidence

	p38	A.53	• Mouse 3T3-L1 adipocytes	Pharmacological perturbation	Tier 3 - pharmacological functional evidence
	MAPK	A.53	• Mouse 3T3-L1 adipocytes	Pharmacological perturbation	Tier 3 - pharmacological functional evidence
	PID1	A.527	Primary brown adipocytes from mice and mouse models: Wild-type (WT), Whole-body PID1 knockout, and Adipocyte-specific PID1 knockout.	Disease/stress model	Tier 2 - functional mechanistic evidence
	PIKfyve	A.257	• Differentiated 3T3-L1 adipocytes (3T3L1 adipocytes).<br>• Human embryonic kidney cells (HEK293).<br>• Chinese hamster ovary cells (CHO-T).	Pharmacological perturbation	Tier 3 - pharmacological functional evidence
	Protein TUG (Tether containing a UBX domain for GLUT4)	A.234	• Differentiated 3T3-L1 adipocytes.	Genetic perturbation/rescue + Pharmacological perturbation + Physiological stimulus	Tier 1 - convergent mechanistic evidence
	PtdIns(3)P	A.518	3T3-L1 adipocytes.	Genetic perturbation/rescue	Tier 2 - functional mechanistic evidence
	PtdIns5P	A.257	• Differentiated 3T3-L1 adipocytes (3T3L1 adipocytes).<br>• Human embryonic kidney cells (HEK293).<br>• Chinese hamster ovary cells (CHO-T).	Pharmacological perturbation	Tier 3 - pharmacological functional evidence
	Rab10	A.24	• Mouse 3T3-L1 adipocyte	Genetic perturbation/rescue + Physiological stimulus	Tier 1 - convergent mechanistic evidence
		A.268	Fat cells differentiate from 3T3-L1 adipocytes and human embryonic kidney cells (HEK-293 cells).	Genetic perturbation/rescue	Tier 2 - functional mechanistic evidence
		A.387	3T3-L1 adipocytes	Genetic perturbation/rescue	Tier 2 - functional mechanistic evidence
		A.401	1. Adipocytes (adipocytes) of the 3T3-L1 line<br><br>2. HEK293 cells.	Genetic perturbation/rescue + Physiological stimulus	Tier 1 - convergent mechanistic evidence
		A.562	3T3-L1 adipocytes	Genetic perturbation/rescue + Physiological stimulus	Tier 1 - convergent mechanistic evidence
		A.564	3T3-L1 adipocytes.	Genetic perturbation/rescue	Tier 2 - functional mechanistic evidence

		A.196	• Primary adipocytes isolated from white adipose tissue (WAT).<br>• Female mice with adipose-specific Rab10 gene deletion (Rab10KO).	Genetic perturbation/rescue	Tier 2 - functional mechanistic evidence
	**Rab11**	A.280	Fat cells differentiate from 3T3-L1 adipocytes and 3T3-L1 fibroblasts.	Genetic perturbation/rescue	Tier 2 - functional mechanistic evidence
	**Rab35**	A.675	3T3-L1 adipocytes, HeLa cells, HEK293 cells.	Genetic perturbation/rescue + Physiological stimulus	Tier 1 - convergent mechanistic evidence
	**Rab4**	A.381	3T3-L1 adipocytes	Genetic perturbation/rescue + Pharmacological perturbation + Physiological stimulus	Tier 1 - convergent mechanistic evidence
		A.733	Differentiated 3T3-L1 adipocytes.	Pharmacological perturbation	Tier 3 - pharmacological functional evidence
	**Rab4 ΔCT**	A.248	• Adipocytes isolated from the epididymal fat pads of male Wistar rats.	Genetic perturbation/rescue	Tier 2 - functional mechanistic evidence
	**Rac**	A.637	3T3-L1 adipocytes.	Genetic perturbation/rescue	Tier 2 - functional mechanistic evidence
	**RalA**	A.51	• Mouse 3T3-L1 adipocytes<br>• Rat skeletal muscle cells (L6 myoblasts stably expressing Myc-Glut4).	Genetic perturbation/rescue + Pharmacological perturbation + Physiological stimulus	Tier 1 - convergent mechanistic evidence
	**Syntaxin 16**	A.535	3T3-L1 adipocytes	Genetic perturbation/rescue	Tier 2 - functional mechanistic evidence
	**Tankyrase**	A.398	3T3-L1 adipocytes	Disease/stress model	Tier 2 - functional mechanistic evidence
	**TBC1D13**	A.675	3T3-L1 adipocytes, HeLa cells, HEK293 cells.	Genetic perturbation/rescue + Physiological stimulus	Tier 1 - convergent mechanistic evidence
	**TC10**	A.637	3T3-L1 adipocytes.	Genetic perturbation/rescue	Tier 2 - functional mechanistic evidence
	**TNKS1 (Tankyrase 1)**	A.689	Differentiated 3T3-L1 adipocytes and HEK293T cells.	Genetic perturbation/rescue + Pharmacological perturbation + Disease/stress model + Physiological stimulus	Tier 1 - convergent mechanistic evidence
	**TNKS2 (Tankyrase 2)**	A.689	Differentiated 3T3-L1 adipocytes and HEK293T cells.	Genetic perturbation/rescue + Pharmacological perturbation + Disease/stress model + Physiological stimulus	Tier 1 - convergent mechanistic evidence

	**Usp25m**	**A.771**	3T3-L1 adipocytes and Primary adipocytes.	Genetic perturbation/rescue + Disease/stress model + Physiological stimulus	Tier 1 - convergent mechanistic evidence
	**VAMP2 (Synaptobrevin 2)**	**A.351**	Adipocytes isolated from epididymal fat pads of male Ola (SD-like)	Physiological stimulus	Tier 4 - descriptive/correlative evidence
	**Vti1a**	**A.744**	Differentiated 3T3-L1 adipocytes.	Genetic perturbation/rescue	Tier 2 - functional mechanistic evidence
	**α-tubulin**	**A.347**	3T3-L1 adipocytes	Pharmacological perturbation	Tier 3 - pharmacological functional evidence
**Tethering**	**EXO70**	**A.153**	• Adipocytes differentiated from the 3T3-L1 fibroblast cell line.	Genetic perturbation/rescue + Physiological stimulus	Tier 1 - convergent mechanistic evidence
		**A.697**	3T3L1 adipocytes and 293T cells.	Genetic perturbation/rescue	Tier 2 - functional mechanistic evidence
	**Exocyst 8-component protein complex**	**A.326**	Adipocytes differentiated from mouse preadipocytes and HeLa cells (for whole-genome CRISPR screening).	Genetic perturbation/rescue	Tier 2 - functional mechanistic evidence
		**A.153**	• Adipocytes differentiated from the 3T3-L1 fibroblast cell line.	Genetic perturbation/rescue + Physiological stimulus	Tier 1 - convergent mechanistic evidence
	**Glucose**	**A.336**	3T3-L1 adipocytes	Pharmacological perturbation	Tier 3 - pharmacological functional evidence
	**Insulin**	**A.285**	Tế bào mỡ 3T3 L1 (3T3 L1 adipocytes).	Genetic perturbation/rescue	Tier 2 - functional mechanistic evidence
	**Munc18c**	**A.336**	3T3-L1 adipocytes	Pharmacological perturbation	Tier 3 - pharmacological functional evidence
	**RalA (GTPase)**	**A.247**	• Differentiated 3T3-L1 adipocytes (used for GLUT4 studies).<br>• Cos-1 cells.<br>• MDCK cells.<br>• Zebrafish embryos.	Genetic perturbation/rescue + Pharmacological perturbation + Physiological stimulus	Tier 1 - convergent mechanistic evidence
	**RGC1**	**A.27**	• Cells: 3T3-L1 adipocytes (differentiated adipocytes)<br>• COS-1 cells<br>• 293T cells<br>• Mouse epididymal adipose tissue	Genetic perturbation/rescue + Pharmacological perturbation + Physiological stimulus	Tier 1 - convergent mechanistic evidence

	RGC2 (AS250)	A.27	• Cells: 3T3-L1 adipocytes (differentiated adipocytes)<br>• COS-1 cells<br>• 293T cells<br>• Mouse epididymal adipose tissue	Genetic perturbation/rescue + Pharmacological perturbation + Physiological stimulus	Tier 1 - convergent mechanistic evidence
	rsec8 (components of the exocyst complex)	A.243	• 3T3-L1 adipocytes.	Genetic perturbation/rescue	Tier 2 - functional mechanistic evidence
	Sec5 (exocyst subunit)	A.247	• Differentiated 3T3-L1 adipocytes (used for GLUT4 studies).<br>• Cos-1 cells.<br>• MDCK cells.<br>• Zebrafish embryos.	Genetic perturbation/rescue + Pharmacological perturbation + Physiological stimulus	Tier 1 - convergent mechanistic evidence
	SEC6	A.153	• Adipocytes differentiated from the 3T3-L1 fibroblast cell line.	Genetic perturbation/rescue + Physiological stimulus	Tier 1 - convergent mechanistic evidence
		A.243	• 3T3-L1 adipocytes.	Genetic perturbation/rescue	Tier 2 - functional mechanistic evidence
	SEC8	A.153	• Adipocytes differentiated from the 3T3-L1 fibroblast cell line.	Genetic perturbation/rescue + Physiological stimulus	Tier 1 - convergent mechanistic evidence
	TC10	A.697	3T3L1 adipocytes and 293T cells.	Genetic perturbation/rescue	Tier 2 - functional mechanistic evidence
	TUG (ASPSCR1)	A.42	• Cells: 3T3-L1 adipocytes, HEK293 cells.<br>• Animals: Male C57BL/6J mice (SIRT2 knockout and wild-type).	Genetic perturbation/rescue + Pharmacological perturbation + Physiological stimulus	Tier 1 - convergent mechanistic evidence
**Docking**	AS160 (TBC1D4)	A.191	• Differentiated 3T3-L1 adipocytes	Genetic perturbation/rescue	Tier 2 - functional mechanistic evidence
	CIP2	A.740	3T3-L1 adipocytes and COS-1 cells (for protein interaction).	Genetic perturbation/rescue	Tier 2 - functional mechanistic evidence
	CIP4	A.740	3T3-L1 adipocytes and COS-1 cells (for protein interaction).	Genetic perturbation/rescue	Tier 2 - functional mechanistic evidence
	Munc18c	A.194	• Differentiated 3T3-L1 adipocytes.	Genetic perturbation/rescue	Tier 2 - functional mechanistic evidence
		A.329	3T3-L1 adipocytes	Disease/stress model	Tier 2 - functional mechanistic evidence
		A.591	3T3-L1 adipocytes.	Genetic perturbation/rescue	Tier 2 - functional mechanistic evidence

	myosin II	A.482	Differentiated 3T3-L1 adipocytes.	Genetic perturbation/rescue	Tier 2 - functional mechanistic evidence
	Protein Kinase C-ζ (PKC-ζ)	A.552	3T3-L1 adipocytes and L6 muscle cells.	Pharmacological perturbation	Tier 3 - pharmacological functional evidence
	Rab10	A.567	3T3-L1 adipocytes.	Genetic perturbation/rescue + Physiological stimulus	Tier 1 - convergent mechanistic evidence
		A.647	3T3-L1 adipocytes.	Genetic perturbation/rescue	Tier 2 - functional mechanistic evidence
		A.26	• 3T3-L1 adipocytes<br>• COS-1 cells<br>• 293T cells	Genetic perturbation/rescue + Physiological stimulus	Tier 1 - convergent mechanistic evidence
	RalA	A.26	• 3T3-L1 adipocytes<br>• COS-1 cells<br>• 293T cells	Genetic perturbation/rescue + Physiological stimulus	Tier 1 - convergent mechanistic evidence
	Rab14	A.647	3T3-L1 adipocytes.	Genetic perturbation/rescue	Tier 2 - functional mechanistic evidence
	Rab4 (small GTPase)	A.190	• Primary adipocytes isolated from epididymal fat tissue of male rats (Rat adipocytes).	Genetic perturbation/rescue	Tier 2 - functional mechanistic evidence
	Synip (Syntaxin 4-interacting protein)	A.665	Differentiated 3T3-L1 adipocytes, CHO/IR cells, HEK293T cells, and mouse tissues.	Genetic perturbation/rescue	Tier 2 - functional mechanistic evidence
	Syntaxin 4 (t-SNARE)	A.190	• Primary adipocytes isolated from epididymal fat tissue of male rats (Rat adipocytes).	Genetic perturbation/rescue	Tier 2 - functional mechanistic evidence
	Tymosin	A.269	Mouse adipocytes and preadipocytes.	Genetic perturbation/rescue + Physiological stimulus	Tier 1 - convergent mechanistic evidence
Fusion	14-3-3 proteins (particularly the γ isoform)	A.91	• Adipocytes isolated from male Wistar rats<br>• HEK293T cell line	Genetic perturbation/rescue	Tier 2 - functional mechanistic evidence
	80K-H	A.298	Cell lines: CHO cells (Cho-K1, Cho-mycGLUT4), 3T3-L1 fat cells, L6 muscle tubes (L6 myotubes), and COS-1 cells.	Genetic perturbation/rescue	Tier 2 - functional mechanistic evidence
	Actin Shell	A.299	3T3-L1 adipocytes	Genetic perturbation/rescue	Tier 2 - functional mechanistic evidence
	Akt (PKB)	A.358	3T3-L1 adipocytes	Genetic perturbation/rescue	Tier 2 - functional mechanistic evidence

	**Akt2**	**A.64**	• Cells: CHO-IR cells (CHO cells overexpressing the human insulin receptor) and 3T3-L1 adipocytes.	Genetic perturbation/rescue + Pharmacological perturbation + Physiological stimulus	Tier 1 - convergent mechanistic evidence
	**ATP**	**A.754**	3T3-L1 adipocytes and 3T3-L1 fibroblast transfectants expressing GLUT4.	Pharmacological perturbation	Tier 3 - pharmacological functional evidence
	**b-Tomosyn (Syntaxin-binding protein)**	**A.749**	3T3-L1 adipocytes and CHO-T cells (CHO cells overexpressing human insulin receptor).	Genetic perturbation/rescue + Physiological stimulus	Tier 1 - convergent mechanistic evidence
	**Calpain-10**	**A.758**	3T3-L1 adipocytes.	Genetic perturbation/rescue	Tier 2 - functional mechanistic evidence
	**CDP138**	**A.112**	• 3T3-L1 adipocytes<br>• HEK293T cells	Genetic perturbation/rescue + Physiological stimulus	Tier 1 - convergent mechanistic evidence
	**Clathrin**	**A.754**	3T3-L1 adipocytes and 3T3-L1 fibroblast transfectants expressing GLUT4.	Pharmacological perturbation	Tier 3 - pharmacological functional evidence
	**Csp1 (Cysteine-string protein 1)**	**A.736**	Differentiated 3T3-L1 adipocytes and L6 skeletal muscle cells.	Genetic perturbation/rescue	Tier 2 - functional mechanistic evidence
	**DOC2b (Double C2 domain protein beta)**	**A.13**	• Mouse 3T3-L1 adipocyte	Genetic perturbation/rescue + Pharmacological perturbation + Physiological stimulus	Tier 1 - convergent mechanistic evidence
	**Phosphatidylinositol 4-kinase type IIIα (PI4K-IIIα)**	**A.229**	• Differentiated 3T3-L1 adipocytes derived from murine fibroblasts.	Genetic perturbation/rescue	Tier 2 - functional mechanistic evidence
	**EFR3 (primarily EFR3A isoform)**	**A.229**	• Differentiated 3T3-L1 adipocytes derived from murine fibroblasts.	Genetic perturbation/rescue	Tier 2 - functional mechanistic evidence
	**Exo70**	**A.352**	Primary isolated adipose cells from male CD rats	Genetic perturbation/rescue	Tier 2 - functional mechanistic evidence
		**A.640**	Mouse 3T3-L1 adipocytes.	Genetic perturbation/rescue	Tier 2 - functional mechanistic evidence
	**GLUT4**	**A.278**	3T3-L1 adipocytes	Genetic perturbation/rescue	Tier 2 - functional mechanistic evidence
		**A.768**	Isolated rat adipose cells.	Pharmacological perturbation	Tier 3 - pharmacological functional evidence

	GTP-binding protein	A.754	3T3-L1 adipocytes and 3T3-L1 fibroblast transfectants expressing GLUT4.	Pharmacological perturbation	Tier 3 - pharmacological functional evidence
	insulin	A.369	White adipose cells were isolated from the epididymal fat pads of male CD mice.	Genetic perturbation/rescue	Tier 2 - functional mechanistic evidence
	Microtubules	A.635	3T3-L1 adipocytes.	Genetic perturbation/rescue	Tier 2 - functional mechanistic evidence
	Munc18-1	A.151	• Recombinant liposome model (Proteoliposomes) containing purified SNARE proteins.<br>• 3T3-L1 adipocytes (used for physiological validation).	Genetic perturbation/rescue	Tier 2 - functional mechanistic evidence
	Munc18c	A.54	• Animals: Mice (C57BL/6J) (Munc18c+/+, +/−, −/−).<br>• Cells: Adipocytes differentiated from mesenchymal embryonic fibroblasts (MEFs) obtained from mouse embryos at day 13.5.	Genetic perturbation/rescue + Pharmacological perturbation + Physiological stimulus	Tier 1 - convergent mechanistic evidence
		A.151	• Recombinant liposome model (Proteoliposomes) containing purified SNARE proteins.<br>• 3T3-L1 adipocytes (used for physiological validation).	Genetic perturbation/rescue	Tier 2 - functional mechanistic evidence
		A.413	1. Adipocytes (adipocytes) of the 3T3-L1 line<br>2. Chinese hamster ovarian cells (CHO)	Genetic perturbation/rescue	Tier 2 - functional mechanistic evidence
		A.471	Differentiated 3T3-L1 adipocytes.	Genetic perturbation/rescue	Tier 2 - functional mechanistic evidence
		A.472	Cells: Differentiated 3T3-L1 fat cells (3T3L1 adipocytes) and HEK293-S3 cells.	Genetic perturbation/rescue	Tier 2 - functional mechanistic evidence
		A.473	Cells: Differentiated 3T3-L1 adipocytes (3T3-L1 adipocytes) and CHO cells express insulin receptor stability (CHO/IR).<br><br>Animal tissue: Mouse hind-limb skeletal muscle.<br><br>Recombinant proteins: Recombinant His-Munc18c, GST-Munc18c.	Genetic perturbation/rescue + Pharmacological perturbation + Physiological stimulus	Tier 1 - convergent mechanistic evidence
		A.474	Animals: Transgenic mice express GLUT4-EGFP under the control of human rhabdomyocardial actin promoter (HSA). C57Bl/6 mouse background.	Genetic perturbation/rescue	Tier 2 - functional mechanistic evidence

			Tissues: Rhabdomyocardia (quadriceps, twin muscles - gastrocnemius), heart, adipose tissue.		
		**A.764**	Differentiated 3T3L1 adipocytes.	Genetic perturbation/rescue	Tier 2 - functional mechanistic evidence
	**Myo1c (Unconventional myosin)**	**A.765**	Differentiated 3T3-L1 adipocytes.	Genetic perturbation/rescue + Physiological stimulus	Tier 1 - convergent mechanistic evidence
	**Myosin IIA**	**A.20**	• Mouse 3T3-L1 adipocyte	Pharmacological perturbation	Tier 3 - pharmacological functional evidence
		**A.215**	Differentiated 3T3-L1 adipocytes.	Pharmacological perturbation	Tier 3 - pharmacological functional evidence
		**A.593**	3T3-L1 adipocytes.	Pharmacological perturbation	Tier 3 - pharmacological functional evidence
	**Myosin IIB**	**A.20**	• Mouse 3T3-L1 adipocyte	Pharmacological perturbation	Tier 3 - pharmacological functional evidence
	**Myosin Light Chain Kinase (MLCK)**	**A.215**	Differentiated 3T3-L1 adipocytes.	Pharmacological perturbation	Tier 3 - pharmacological functional evidence
		**A.594**	3T3-L1 adipocytes.	Genetic perturbation/rescue	Tier 2 - functional mechanistic evidence
	**NSF SNAPs**	**A.100**	• Primary mouse adipocytes	Physiological stimulus	Tier 4 - descriptive/correlative evidence
	**other Rab3 isoforms**	**A.354**	1. Primary rat adipocytes: From the epididymal tissue of Wistar mice (used for most GTP and Rab3B mutant binding experiments).<br><br>2. 3T3-L1 fat cells: Used for siRNA knockdown experiments of Rab3 isoforms.	Genetic perturbation/rescue + Pharmacological perturbation + Physiological stimulus	Tier 1 - convergent mechanistic evidence
	**Phosphatidic acid (PA)**	**A.410**	Adipocytes of the 3T3-L1 lineage.	Genetic perturbation/rescue	Tier 2 - functional mechanistic evidence
		**A.81**	• Mouse 3T3-L1 adipocytes<br>• L6 myoblasts	Genetic perturbation/rescue	Tier 2 - functional mechanistic evidence
	**Phospholipase D1 (PLD1)**	**A.410**	Adipocytes of the 3T3-L1 lineage.	Genetic perturbation/rescue	Tier 2 - functional mechanistic evidence

	Phosphorylated Syntaxin 4	A.519	HeLa cells (expressing GLUT4) and 3T3-L1 adipocytes.	Genetic perturbation/rescue	Tier 2 - functional mechanistic evidence
	PI3K	A.358	3T3-L1 adipocytes	Genetic perturbation/rescue	Tier 2 - functional mechanistic evidence
		A.399	Adipocytes of the 3T3-L1 lineage.	Pharmacological perturbation	Tier 3 - pharmacological functional evidence
	PKCζ	A.298	Cell lines: CHO cells (Cho-K1, Cho-mycGLUT4), 3T3-L1 fat cells, L6 muscle tubes (L6 myotubes), and COS-1 cells.	Genetic perturbation/rescue	Tier 2 - functional mechanistic evidence
	Rab3B	A.354	1. Primary rat adipocytes: From the epididymal tissue of Wistar mice (used for most GTP and Rab3B mutant binding experiments).<br><br>2. 3T3-L1 fat cells: Used for siRNA knockdown experiments of Rab3 isoforms.	Genetic perturbation/rescue + Pharmacological perturbation + Physiological stimulus	Tier 1 - convergent mechanistic evidence
	Rip11	A.608	3T3-L1 adipocytes.	Genetic perturbation/rescue + Pharmacological perturbation + Physiological stimulus	Tier 1 - convergent mechanistic evidence
	SNAP25	A.396	human skeletal muscle tissue and adipose tissue; mouse 3T3-L1 fat cell line; PC12 cells (positive control).	Physiological stimulus	Tier 4 - descriptive/correlative evidence
		A.260	• Differentiated 3T3-L1 adipocytes.	Physiological stimulus	Tier 4 - descriptive/correlative evidence
	SNAP23	A.618	Differentiated 3T3-L1 adipocytes (for functional assays) and COS cells (for protein interaction assays).	Genetic perturbation/rescue	Tier 2 - functional mechanistic evidence
		A.737	Differentiated 3T3-L1 adipocytes.	Genetic perturbation/rescue	Tier 2 - functional mechanistic evidence
		A.660	Mouse 3T3-L1 adipocytes.	Pharmacological perturbation	Tier 3 - pharmacological functional evidence
		A.745	Differentiated 3T3-L1 adipocytes.	Pharmacological perturbation	Tier 3 - pharmacological functional evidence
	Snapin	A.640	Mouse 3T3-L1 adipocytes.	Genetic perturbation/rescue	Tier 2 - functional mechanistic evidence

	**Synaptobrevin 1**	**A.396**	human skeletal muscle tissue and adipose tissue; mouse 3T3-L1 fat cell line; PC12 cells (positive control).	Physiological stimulus	Tier 4 - descriptive/correlative evidence
	**Synip (Syntaxin 4 interacting protein)**	**A.672**	Differentiated 3T3-L1 adipocytes and CHO/IR cells.	Genetic perturbation/rescue + Pharmacological perturbation + Physiological stimulus	Tier 1 - convergent mechanistic evidence
	**Syntaxin 4**	**A.260**	• Differentiated 3T3-L1 adipocytes.	Physiological stimulus	Tier 4 - descriptive/correlative evidence
		**A.396**	human skeletal muscle tissue and adipose tissue; mouse 3T3-L1 fat cell line; PC12 cells (positive control).	Physiological stimulus	Tier 4 - descriptive/correlative evidence
		**A.411**	Adipocytes of the 3T3-L1 line are permeated with Streptolysin O	Pharmacological perturbation	Tier 3 - pharmacological functional evidence
		**A.413**	1. Adipocytes (adipocytes) of the 3T3-L1 line<br>2. Chinese hamster ovarian cells (CHO)	Genetic perturbation/rescue	Tier 2 - functional mechanistic evidence
		**A.442**	Adipocytes of the 3T3-L1 lineage.	Genetic perturbation/rescue	Tier 2 - functional mechanistic evidence
		**A.471**	Differentiated 3T3-L1 adipocytes.	Genetic perturbation/rescue	Tier 2 - functional mechanistic evidence
		**A.472**	Cells: Differentiated 3T3-L1 fat cells (3T3L1 adipocytes) and HEK293-S3 cells.	Genetic perturbation/rescue	Tier 2 - functional mechanistic evidence
		**A.660**	Mouse 3T3-L1 adipocytes.	Pharmacological perturbation	Tier 3 - pharmacological functional evidence
		**A.668**	Differentiated 3T3-L1 adipocytes.	Physiological stimulus	Tier 3 - pharmacological functional evidence
		**A.669**	Transgenic mice overexpressing Syntaxin 4 on a C57BL/6J background (both sexes, 4-6 months old).	Genetic perturbation/rescue	Tier 2 - functional mechanistic evidence
		**A.670**	Differentiated 3T3-L1 adipocytes and rat epididymal fat pads (for mRNA).	Genetic perturbation/rescue + Physiological stimulus	Tier 1 - convergent mechanistic evidence
		**A.737**	Differentiated 3T3-L1 adipocytes.	Genetic perturbation/rescue	Tier 2 - functional mechanistic evidence

		A.745	Differentiated 3T3-L1 adipocytes.	Pharmacological perturbation	Tier 3 - pharmacological functional evidence
		A.368	3T3-L1 adipocytes	Physiological stimulus	Tier 4 - descriptive/correlative evidence
	**TBK1**	A.520	3T3-L1 adipocytes and Adipocyte-specific TBK1 knockout mice (TBK1 AKO)	Genetic perturbation/rescue + Pharmacological perturbation + Physiological stimulus	Tier 1 - convergent mechanistic evidence
	**Tctex1d2 (Tctex1 domain containing 2)**	A.681	Mouse 3T3-L1 adipocytes.	Genetic perturbation/rescue + Physiological stimulus	Tier 1 - convergent mechanistic evidence
	**Tmod3 (Tropomodulin 3)**	A.757	3T3-L1 adipocytes.	Genetic perturbation/rescue + Physiological stimulus	Tier 1 - convergent mechanistic evidence
	**Tomosyn (STXBP5)**	A.750	Differentiated 3T3-L1 adipocytes.	Genetic perturbation/rescue + Pharmacological perturbation + Physiological stimulus	Tier 1 - convergent mechanistic evidence
	**Tropomyosin Tpm3.1**	A.75	• Differentiated 3T3-L1 adipocytes<br>• Mice (C57BL/6 and FVB/N strains), including transgenic mice overexpressing Tpm3.1 (Tg) and Tpm3.1 knockout (KO) mice<br>• Mouse white adipose tissue (WAT)<br>• Mouse skeletal muscle	Genetic perturbation/rescue + Pharmacological perturbation + Physiological stimulus	Tier 1 - convergent mechanistic evidence
	**TUG**	A.758	3T3-L1 adipocytes.	Genetic perturbation/rescue	Tier 2 - functional mechanistic evidence
	**VAMP2 (Synaptobrevin 2)**	A.139	• 3T3-L1 adipocytes differentiated from fibroblasts	Physiological stimulus	Tier 4 - descriptive/correlative evidence
		A.260	• Differentiated 3T3-L1 adipocytes.	Physiological stimulus	Tier 4 - descriptive/correlative evidence
		A.315	White adipose cells and brown adipose cells isolated from male Sprague-Dawley rats.	Physiological stimulus	Tier 4 - descriptive/correlative evidence
		A.396	human skeletal muscle tissue and adipose tissue; mouse 3T3-L1 fat cell line; PC12 cells (positive control).	Physiological stimulus	Tier 4 - descriptive/correlative evidence
		A.411	Adipocytes of the 3T3-L1 line are permeated with Streptolysin O	Pharmacological perturbation	Tier 3 - pharmacological functional evidence

		A.737	Differentiated 3T3-L1 adipocytes.	Genetic perturbation/rescue	Tier 2 - functional mechanistic evidence
		A.745	Differentiated 3T3-L1 adipocytes.	Pharmacological perturbation	Tier 3 - pharmacological functional evidence
		A.775	SLO-permeabilized 3T3-L1 adipocytes.	Physiological stimulus	Tier 4 - descriptive/correlative evidence
		A.452	3T3L1 adipocytes	Genetic perturbation/rescue	Tier 2 - functional mechanistic evidence
	VAMP3 (Cellubrevin)	A.139	• 3T3-L1 adipocytes differentiated from fibroblasts	Physiological stimulus	Tier 4 - descriptive/correlative evidence
		A.146	• 3T3-L1 adipocytes (differentiated from fibroblasts)<br>• Cells permeabilized with Streptolysin-O (SLO)	Pharmacological perturbation	Tier 3 - pharmacological functional evidence
		A.411	Adipocytes of the 3T3-L1 line are permeated with Streptolysin O	Pharmacological perturbation	Tier 3 - pharmacological functional evidence
		A.775	SLO-permeabilized 3T3-L1 adipocytes.	Physiological stimulus	Tier 4 - descriptive/correlative evidence
	VAMP4	A.139	• 3T3-L1 adipocytes differentiated from fibroblasts	Physiological stimulus	Tier 4 - descriptive/correlative evidence
	VAMP5	A.139	• 3T3-L1 adipocytes differentiated from fibroblasts	Physiological stimulus	Tier 4 - descriptive/correlative evidence
	VAMP7	A.139	• 3T3-L1 adipocytes differentiated from fibroblasts	Physiological stimulus	Tier 4 - descriptive/correlative evidence
		A.452	3T3L1 adipocytes	Genetic perturbation/rescue	Tier 2 - functional mechanistic evidence
	VAMP8	A.139	• 3T3-L1 adipocytes differentiated from fibroblasts	Physiological stimulus	Tier 4 - descriptive/correlative evidence
	VAP-33	A.9	• Rat skeletal muscle cells (L6 skeletal myoblasts) stably expressing myc-tagged GLUT4 (L6-GLUT4myc).<br>• Mouse adipose cells (3T3-L1 adipocytes).	Genetic perturbation/rescue + Physiological stimulus	Tier 1 - convergent mechanistic evidence
