# Supplementary material for: Molecular architecture of GLUT4 trafficking and exocytosis in adipocytes and skeletal muscle: A systematic review and integrated conceptual framework
Source: Front Endocrinol (Lausanne). 2026 Oct 19;17:1932455. doi: 10.3389/fendo.2026.1932455 (PMC13725575; doi:10.3389/fendo.2026.1932455)
Supplement: Supplementary file 3 [file Table3.pdf]

Table S3. Molecular mediators of GLUT4 exocytosis in skeletal muscle cells. Comprehensive summary of signaling molecules, Rab GTPases, cytoskeletal regulators, tethering factors, docking proteins, and SNARE-associated components involved in skeletal muscle GLUT4 trafficking.

Phase	Primary molecule studied	Ref	Primary model system	Intervention class	Intervention tier
**Signaling**	**4-HPA**	**A.273**	L6-line mouse skeletal musculoskeletal cells express myc-tagged GLUT4 (L6-GLUT4myc myotubes).	Pharmacological perturbation	Tier 3 - pharmacological functional evidence
	**Akt (PKB)**	**A.223**	• Male Sprague–Dawley rats with type 2 diabetes induced by a high-fat diet (HFD) combined with low-dose streptozotocin (STZ).<br>• Tissue examined: soleus muscle.	Disease/stress model	Tier 4 - descriptive/correlative evidence
		**A.242**	• Intact soleus muscle isolated from male Wistar rats (body weight 55–65 g)	Pharmacological perturbation	Tier 3 - pharmacological functional evidence
		**A.324**	Skeletal muscle (epitrochlearis muscle) isolated from male rats of the Wistar lineage.	Pharmacological perturbation	Tier 3 - pharmacological functional evidence
	**Akt1**	**A.223**	• Male Sprague–Dawley rats with type 2 diabetes induced by a high-fat diet (HFD) combined with low-dose streptozotocin (STZ).<br>• Tissue examined: soleus muscle.	Disease/stress model	Tier 4 - descriptive/correlative evidence
		**A.544**	L6 myoblasts stably transfected with GLUT4 cDNA carrying an exofacial myc-epitope tag (L6-GLUT4myc myoblasts).	Genetic perturbation/rescue	Tier 2 - functional mechanistic evidence
	**Akt2**	**A.7**	• Mouse skeletal muscle L6 cells with a C57BL/6 genetic background	Genetic perturbation/rescue + Physiological stimulus	Tier 1 - convergent mechanistic evidence
		**A.65**	• Mouse skeletal muscle cells: C2C12 cell line	Genetic perturbation/rescue	Tier 2 - functional mechanistic evidence
		**A.223**	• Male Sprague–Dawley rats with type 2 diabetes induced by a high-fat diet (HFD) combined with low-dose streptozotocin (STZ).<br>• Tissue examined: soleus muscle.	Disease/stress model	Tier 4 - descriptive/correlative evidence
		**A.427**	Musculoskeletal fibers from the gastrocnemius muscle of male C57BL/6 mice (conventional wild-type mice)	Genetic perturbation/rescue	Tier 2 - functional mechanistic evidence

	AMPK	A.2	Male Sprague-Dawley rats	Pharmacological perturbation	Tier 3 - pharmacological functional evidence
		A.135	• Mouse H-2K^b skeletal muscle cell line	Disease/stress model	Tier 2 - functional mechanistic evidence
		A.223	• Male Sprague–Dawley rats with type 2 diabetes induced by a high-fat diet (HFD) combined with low-dose streptozotocin (STZ).<br>• Tissue examined: soleus muscle.	Disease/stress model	Tier 4 - descriptive/correlative evidence
		A.276	The human skeletal muscle cell line (SKM) is differentiated into myotubes.	Genetic perturbation/rescue	Tier 2 - functional mechanistic evidence
		A.541	Female mice strains: Wild-type (WT) C57BL/6J, Muscle-specific Kinase Dead α2 (AMPK KD), Muscle-specific Double Knockout α1/α2 (AMPK mdKO), Whole-body Knockout (AMPK γ3 KO). Isolated Soleus and Extensor Digitorum Longus (EDL) muscles.	Genetic perturbation/rescue	Tier 2 - functional mechanistic evidence
	aPKC	A.223	• Male Sprague–Dawley rats with type 2 diabetes induced by a high-fat diet (HFD) combined with low-dose streptozotocin (STZ).<br>• Tissue examined: soleus muscle.	Disease/stress model	Tier 4 - descriptive/correlative evidence
	APPL2	A.685	C2C12 and L6 myotubes, isolated skeletal muscles (EDL, soleus), and HEK293 cells.	Genetic perturbation/rescue + Pharmacological perturbation + Physiological stimulus	Tier 1 - convergent mechanistic evidence
	AS160 (TBC1D4)	A.93	• Tibialis anterior muscles of female ICR mice, 8 weeks old	Genetic perturbation/rescue	Tier 2 - functional mechanistic evidence
		A.115	• Female ICR mice (tibialis anterior muscles)	Genetic perturbation/rescue	Tier 2 - functional mechanistic evidence
		A.176	• AS160 whole-body knockout mice (AS160(-/-)) and primary tissues/cells isolated from them: white adipocytes (from epididymal/periuterine fat), soleus muscle, extensor digitorum longus (EDL), gastrocnemius muscle, and quadriceps muscle.	Genetic perturbation/rescue	Tier 2 - functional mechanistic evidence

		A.206	• Mouse skeletal muscle, including extensor digitorum longus (EDL), gastrocnemius, and tibialis anterior muscles.<br>• Mouse models used: female ICR mice, Akt2 knockout (KO) mice, and AMPKα2-inactive (α2i) transgenic mice.	Genetic perturbation/rescue	Tier 2 - functional mechanistic evidence
		A.324	Skeletal muscle (epitrochlearis muscle) isolated from male rats of the Wistar lineage.	Pharmacological perturbation	Tier 3 - pharmacological functional evidence
		A.476	Tế bào cơ L6 (L6 muscle cells) biểu hiện GLUT4 gắn thẻ myc (L6-GLUT4myc), bao gồm cả giai đoạn nguyên bào cơ (myoblasts) and ống cơ (myotubes).	Genetic perturbation/rescue + Physiological stimulus	Tier 1 - convergent mechanistic evidence
		A.677	Whole-body Tbc1d4 knockout mice (Whole-body D4KO) and muscle-specific Tbc1d4 knockout mice (Muscle-specific mD4KO), along with Wild-type (WT) littermates. Isolated Extensor Digitorum Longus (EDL) and Tibialis anterior muscles.	Genetic perturbation/rescue + Pharmacological perturbation + Physiological stimulus	Tier 1 - convergent mechanistic evidence
		A.721	L6 GLUT4myc myoblasts and CHO-IR cells.	Genetic perturbation/rescue	Tier 2 - functional mechanistic evidence
		A.780	AS160 knockout Wistar rats (AS160-KO rats) and Wild-type (WT) littermates. Isolated tissues: Soleus muscle, Epitrochlearis muscle, Epididymal adipose tissue.	Genetic perturbation/rescue	Tier 2 - functional mechanistic evidence
	**ATM protein kinase**	A.104	• Male Wistar rats (insulin resistance induced by a high-fat diet)<br>• Mouse embryonic fibroblast (MEF) cell lines A29 (ATM+/+) and A38 (ATM−/−)<br>• Rat L6 myoblast cell line	Disease/stress model	Tier 2 - functional mechanistic evidence
	**Ca2+**	A.341	L6-line skeletal muscle cells express stable GLUT4 tagged extracellular Myc (L6-GLUT4myc myotubes).<br><br>Primary skeletal muscle cells isolated from Sprague-Dawley neonatal rats	Genetic perturbation/rescue	Tier 2 - functional mechanistic evidence
		A.475	L6 rhabdomyocytes (L6 myotubes) stably express myc-tagged GLUT4 (L6-GLUT4myc).	Genetic perturbation/rescue	Tier 2 - functional mechanistic evidence
	**CaMKII (particularly the δ isoform)**	A.117	• Mouse C2C12 skeletal muscle cells (C2C12 myoblasts) differentiated into myotubes	Genetic perturbation/rescue + Pharmacological perturbation + Physiological stimulus	Tier 1 - convergent mechanistic evidence

	**CaMKII (α, β, γ isoforms)**	**A.113**	• Rat L6 skeletal muscle cells differentiated into myotubes	Pharmacological perturbation	Tier 3 - pharmacological functional evidence
	**Caveolin-3**	**A.646**	Conditionally immortalized skeletal muscle cell lines (normal endogenous caveolin-3 or caveolin-3 deficient).	Genetic perturbation/rescue	Tier 2 - functional mechanistic evidence
	**FLJ00068**	**A.578**	Skeletal muscle of adult male C57BL/6 mice and L6-GLUT4 rat myoblasts.	Genetic perturbation/rescue + Physiological stimulus	Tier 1 - convergent mechanistic evidence
		**A.619**	L6-GLUT4 myocytes derived from adult male rat and mouse myoblasts, and muscle-specific rac1 knockout mice.	Genetic perturbation/rescue + Physiological stimulus	Tier 1 - convergent mechanistic evidence
	**Flotillin-1**	**A.646**	Conditionally immortalized skeletal muscle cell lines (normal endogenous caveolin-3 or caveolin-3 deficient).	Genetic perturbation/rescue	Tier 2 - functional mechanistic evidence
	**GIV**	**A.270**	Line cells: HeLa cells, L6 rat skeletal myotubes, and Cos7 cells.	Genetic perturbation/rescue + Pharmacological perturbation + Physiological stimulus	Tier 1 - convergent mechanistic evidence
	**Glucose**	**A.271**	Rat adipocytes and Rat skeletal muscles: soleus muscles and extensor digitorum longus (EDL).	Genetic perturbation/rescue	Tier 2 - functional mechanistic evidence
		**A.322**	L6-line skeletal muscle cells expressing the human insulin receptor (L6hIR)	Genetic perturbation/rescue	Tier 2 - functional mechanistic evidence
	**Gαi2**	**A.291**	Transgenic mice (iQ205L) express the activated Gαi2(Q205L) mutation in adipose tissue, skeletal muscle and liver; along with wild-type littermates.	Genetic perturbation/rescue	Tier 2 - functional mechanistic evidence
	**H2O2**	**A.341**	L6-line skeletal muscle cells express stable GLUT4 tagged extracellular Myc (L6-GLUT4myc myotubes).<br><br>Primary skeletal muscle cells isolated from Sprague-Dawley neonatal rats	Genetic perturbation/rescue	Tier 2 - functional mechanistic evidence
	**HIF-1 alpha**	**A.451**	C2C12 myoblast	Genetic perturbation/rescue + Disease/stress model	Tier 1 - convergent mechanistic evidence
	**Irisin**	**A.429**	1. L6 rat myoblast cells have differentiated into myotubes.<br><br>2. Primary myoblasts isolated from the front and hind legs of mouse pups.	Genetic perturbation/rescue	Tier 2 - functional mechanistic evidence

	**Isoleucine**	A.76	• Male Sprague–Dawley rats, approximately 9 weeks old	Physiological stimulus	Tier 4 - descriptive/correlative evidence
	**K+**	A.475	L6 rhabdomyocytes (L6 myotubes) stably express myc-tagged GLUT4 (L6-GLUT4myc).	Genetic perturbation/rescue	Tier 2 - functional mechanistic evidence
	**Leucine**	A.76	• Male Sprague–Dawley rats, approximately 9 weeks old	Physiological stimulus	Tier 4 - descriptive/correlative evidence
	**MARCKS (Myristoylated alanine-rich C-kinase substrate)**	A.258	• L6 rat skeletal muscle cells (L6 myotubes).	Genetic perturbation/rescue + Pharmacological perturbation + Physiological stimulus	Tier 1 - convergent mechanistic evidence
	**Myotubularin (MTM1)**	A.251	• Muscle cell lines: L6 myotubes, C2C12 myotubes.<br>• Adipocyte cell lines: 3T3-L1 adipocytes (specifically the CAR"delta"1 line).	Genetic perturbation/rescue	Tier 2 - functional mechanistic evidence
	**Nexilin**	A.493	L6 rat skeletal muscle cells (myotubes) and 3T3-L1 mouse adipocytes.	Genetic perturbation/rescue	Tier 2 - functional mechanistic evidence
	**NYGGF4 (gen)**	A.439	C2C12 differentiated mouse musculoskeletal cells	Genetic perturbation/rescue	Tier 2 - functional mechanistic evidence
	**p38 MAPK**	A.187	• Differentiated L6 muscle cells (L6 myotubes) stably expressing Myc-tagged GLUT4 (L6-GLUT4myc).	Genetic perturbation/rescue	Tier 2 - functional mechanistic evidence
		A.283	L6 series myotubes express stable GLUT4 tagged Myc in the extracellular (L6-GLUT4myc).	Pharmacological perturbation	Tier 3 - pharmacological functional evidence
	**PDGF-BB (A Type of Growth Factor)**	A.340	L6 myoblasts express GLUT4 stably tagged with Myc (GLUT4myc).	Pharmacological perturbation	Tier 3 - pharmacological functional evidence
	**PI3K**	A.187	• Differentiated L6 muscle cells (L6 myotubes) stably expressing Myc-tagged GLUT4 (L6-GLUT4myc).	Genetic perturbation/rescue	Tier 2 - functional mechanistic evidence
	**PI3K-C2α (Phosphoinositide 3-kinase class II alpha)**	A.727	L6 muscle cells.	Genetic perturbation/rescue	Tier 2 - functional mechanistic evidence
	**PI5P**	A.425	Myotubes differentiated from the L6 myoblast cell line	Genetic perturbation/rescue	Tier 2 - functional mechanistic evidence

	**Pikfyve**	**A.478**	Animal: Mouse (C57BL/6J background). Specifically, MPIfKO (Muscle-specific PIKfyve Knockout) mice and control mice. Tissue: Isolated rhabdomyoplasty (soleus muscle, long toe extensor muscle - EDL, lower leg twin muscle - gastrocnemius) and adipose tissue.	Disease/stress model	Tier 2 - functional mechanistic evidence
	**PIP4Kα**	**A.425**	Myotubes differentiated from the L6 myoblast cell line	Genetic perturbation/rescue	Tier 2 - functional mechanistic evidence
	**PKC**	**A.530**	L6 myoblast muscle cells stably expressing myc-tagged GLUT4 (GLUT4myc).	Genetic perturbation/rescue	Tier 2 - functional mechanistic evidence
	**PKCα**	**A.322**	L6-line skeletal muscle cells expressing the human insulin receptor (L6hIR)	Genetic perturbation/rescue	Tier 2 - functional mechanistic evidence
	**PKCε**	**A.531**	C2C12 myotubes stably expressing myc-tagged GLUT4 (GLUT4myc)	Genetic perturbation/rescue	Tier 2 - functional mechanistic evidence
	**Protein Kinase C-betaII (PKCβII)**	**A.258**	• L6 rat skeletal muscle cells (L6 myotubes).	Genetic perturbation/rescue + Pharmacological perturbation + Physiological stimulus	Tier 1 - convergent mechanistic evidence
	**Protein Kinase C-δ (PKC-δ)**	**A.549**	Rat skeletal muscle cells.	Genetic perturbation/rescue	Tier 2 - functional mechanistic evidence
	**PKCζ**	**A.339**	rat adipocytes and L6 myotubes	Genetic perturbation/rescue	Tier 2 - functional mechanistic evidence
	**PKCζ**	**A.550**	Differentiated L6 rat myotubes expressing myc-tagged GLUT4 (L6myc cells) or overexpressing PKC-ζ.	Genetic perturbation/rescue	Tier 2 - functional mechanistic evidence
	**Protein Kinase C-λ (PKC-λ)**	**A.222**	L6 myotubes (fully differentiated rat skeletal muscle cells derived from L6 myoblasts).	Genetic perturbation/rescue	Tier 2 - functional mechanistic evidence
	**Protein Kinase D3 (PKD3)**	**A.545**	L6 myotubes - a rat skeletal muscle cell line.	Genetic perturbation/rescue + Pharmacological perturbation + Physiological stimulus	Tier 1 - convergent mechanistic evidence
	**PtdIns-3-P**	**A.344**	Skeletal muscle cells of the L6 line and fat cells of the 3T3-L1 line.	Genetic perturbation/rescue	Tier 2 - functional mechanistic evidence
	**PtdIns-3-P**	**A.251**	• Muscle cell lines: L6 myotubes, C2C12 myotubes.<br>• Adipocyte cell lines: 3T3-L1 adipocytes (specifically the CAR"delta"1 line).	Genetic perturbation/rescue	Tier 2 - functional mechanistic evidence

	**Rab13**	**A.232**	• Differentiated C2C12 myotubes stably expressing HA-tagged GLUT4 (C2C12-GLUT4HA)	Genetic perturbation/rescue	Tier 2 - functional mechanistic evidence
		**A.530**	L6 myoblast muscle cells stably expressing myc-tagged GLUT4 (GLUT4myc).	Genetic perturbation/rescue	Tier 2 - functional mechanistic evidence
	**Rab14**	**A.232**	• Differentiated C2C12 myotubes stably expressing HA-tagged GLUT4 (C2C12-GLUT4HA)	Genetic perturbation/rescue	Tier 2 - functional mechanistic evidence
	**Rab8A**	**A.232**	• Differentiated C2C12 myotubes stably expressing HA-tagged GLUT4 (C2C12-GLUT4HA)	Genetic perturbation/rescue	Tier 2 - functional mechanistic evidence
	**Rac1**	**A.7**	• Mouse skeletal muscle L6 cells with a C57BL/6 genetic background	Genetic perturbation/rescue + Physiological stimulus	Tier 1 - convergent mechanistic evidence
		**A.427**	Musculoskeletal fibers from the gastrocnemius muscle of male C57BL/6 mice (conventional wild-type mice)	Genetic perturbation/rescue	Tier 2 - functional mechanistic evidence
		**A.610**	L6 myoblasts.	Genetic perturbation/rescue	Tier 2 - functional mechanistic evidence
	**TBC1D1**	**A.38**	• Animals: Mice.<br>• Mouse models: Tbc1d1 Ser231Ala knock-in mice, Ampkα1/α2-mKO (skeletal muscle–specific knockout) mice, and wild-type (WT) mice.<br>• Tissue samples: Isolated soleus and EDL (extensor digitorum longus) muscles	Genetic perturbation/rescue + Pharmacological perturbation + Physiological stimulus	Tier 1 - convergent mechanistic evidence
		**A.327**	3T3-L1 adipocytes, L6 muscle cells (L6 cells), and tissues from wild rats or AS160 knockout mice.	Genetic perturbation/rescue + Pharmacological perturbation + Physiological stimulus	Tier 1 - convergent mechanistic evidence
		**A.408**	1. C2C1 muscle cells.<br>2. 3T3-L1 fat cells.<br>3. Human muscle biopsy samples: Taken from healthy people and type 2 diabetics.	Disease/stress model	Tier 2 - functional mechanistic evidence
		**A.476**	Tế bào cơ L6 (L6 muscle cells) biểu hiện GLUT4 gắn thẻ myc (L6-GLUT4myc), bao gồm cả giai đoạn nguyên bào cơ (myoblasts) and ống cơ (myotubes).	Genetic perturbation/rescue + Physiological stimulus	Tier 1 - convergent mechanistic evidence
	**TBC1D1 (phosphorylated)**	**A.193**	• Male ICR mice, 8–10 weeks of age.<br>• Skeletal muscles examined included soleus, tibialis anterior (TA), and extensor digitorum longus (EDL).	Pharmacological perturbation	Tier 3 - pharmacological functional evidence

		A.154	• Human embryonic kidney cells (HEK-293).<br>• Muscle cells (L6 myotubes) differentiated from rat myoblasts.	Pharmacological perturbation	Tier 3 - pharmacological functional evidence
	Unc119b	A.331	Mouse: C57BL/6 series (including WT wild mice, Unc119b KO gene-deleting mice, Unc119a KO gene deleting mice) and db/db leptin receptor mutant mice.<br>+4<br><br>Cells: C2C12 muscle cell lines, HBG4 reporter cell lines (C2C12 expressing HiBiT-GLUT4), and HEK293T cells	Disease/stress model	Tier 2 - functional mechanistic evidence
	WNK1 (With-No-Lysine [K] Kinase 1)	A.781	L6 myotubes and Mouse skeletal muscle (ex vivo).	Genetic perturbation/rescue	Tier 2 - functional mechanistic evidence
Translocation	Actin	A.418	Tế bào cơ xương (myotubes) dòng L6 biểu hiện GLUT4myc.<br>Tế bào cơ xương (myotubes) dòng L6 biểu hiện GLUT4myc.<br>L6 myotubes express GLUT4myc.	Genetic perturbation/rescue	Tier 2 - functional mechanistic evidence
		A.460	L6 skeletal muscle cells express GLUT4 tagged with c-myc (L6myc cells), which are differentiated into myotubes.	Pharmacological perturbation	Tier 3 - pharmacological functional evidence
		A.785	L6-GLUT4myc myoblasts; C57BL/6 mouse skeletal muscle (ex vivo).	Genetic perturbation/rescue + Pharmacological perturbation + Physiological stimulus	Tier 1 - convergent mechanistic evidence
		A.192	Differentiated mouse skeletal muscle cells (L6 myotubes).	Pharmacological perturbation	Tier 3 - pharmacological functional evidence
	ACTN4 (α-actinin-4)	A.782	L6-GLUT4myc myoblasts.	Genetic perturbation/rescue + Pharmacological perturbation + Physiological stimulus	Tier 1 - convergent mechanistic evidence
	AICAR	A.434	1. Male rats Imprinting Control Region (ICR) (7-10 weeks old).<br><br>2. Muscle-specific AMPKα2 inactivated transgenic mice on FVB and control mice, respectively.<br><br>3. Quadriceps muscle fibers of live mice.	Genetic perturbation/rescue	Tier 2 - functional mechanistic evidence

	**AMPKα2**	**A.434**	1. Male rats Imprinting Control Region (ICR) (7-10 weeks old).<br><br>2. Muscle-specific AMPKα2 inactivated transgenic mice on FVB and control mice, respectively.<br><br>3. Quadriceps muscle fibers of live mice.	Genetic perturbation/rescue	Tier 2 - functional mechanistic evidence
	**ARP2/3 complex**	**A.87**	• Rat L6 muscle cells stably expressing myc-tagged GLUT4	Genetic perturbation/rescue + Pharmacological perturbation + Physiological stimulus	Tier 1 - convergent mechanistic evidence
	**ARP3**	**A.87**	• Rat L6 muscle cells stably expressing myc-tagged GLUT4	Genetic perturbation/rescue + Pharmacological perturbation + Physiological stimulus	Tier 1 - convergent mechanistic evidence
	**ARPC2 (p34)**	**A.87**	• Rat L6 muscle cells stably expressing myc-tagged GLUT4	Genetic perturbation/rescue + Pharmacological perturbation + Physiological stimulus	Tier 1 - convergent mechanistic evidence
	**AS160 (TBC1D4)**	**A.457**	_ AS160 Thr649Ala knockin mice<br>- epididymal fat pads<br>- Primary adipose cells and isolated soleus muscles	Physiological stimulus	Tier 4 - descriptive/correlative evidence
	**Beta-catenin**	**A.785**	L6-GLUT4myc myoblasts; C57BL/6 mouse skeletal muscle (ex vivo).	Genetic perturbation/rescue + Pharmacological perturbation + Physiological stimulus	Tier 1 - convergent mechanistic evidence
	**CBP (Lysine acetyltransferases)**	**A.510**	Animals: Skeletal muscle-specific p300/CBP knockout mice (PCKO mice) and related compound heterozygous lines.<br><br>Cells: L6 myoblasts/myotubes, 3T3-L1 adipocytes, and primary human skeletal muscle cells.	Genetic perturbation/rescue	Tier 2 - functional mechanistic evidence
	**CHIP**	**A.449**	Mouse lacks CHIP (CHIP -/- mice)<br><br>- Mouse skeletal muscle cell C2C12 (rabid type and CHIP deficient)<br>- Mouse embryonic fibroblasts (rabid and CHIP-deficient)<br>- Muscle fibers isolated from rabies-type or CHIP-deficient mice after fasting or glucose injections	Physiological stimulus	Tier 4 - descriptive/correlative evidence
	**Conventional kinesin**	**A.380**	1. Myoblasts of the C2C12 line<br><br>2. Other: CHO-IR cells (yellow guinea pig ovarian cells overexpressing insulin receptors), 293 cells (human embryonic kidney cells)	Genetic perturbation/rescue	Tier 2 - functional mechanistic evidence

	**FAK**	**A.252**	• C2C12 skeletal muscle cells differentiated into myotubes, studied under two conditions: insulin-sensitive (MF) and insulin-resistant (MFI).	Genetic perturbation/rescue + Pharmacological perturbation + Physiological stimulus	Tier 1 - convergent mechanistic evidence
	**GLUT1**	**A.372**	Skeletal muscles of transgenic mice overexpress Glut1 or Glut4	Physiological stimulus	Tier 4 - descriptive/correlative evidence
	**GLUT4**	**A.372**	Skeletal muscles of transgenic mice overexpress Glut1 or Glut4	Physiological stimulus	Tier 4 - descriptive/correlative evidence
		**A.738**	Male Wistar rats (250-300g); white portions of gastrocnemius and quadriceps muscles.	Physiological stimulus	Tier 4 - descriptive/correlative evidence
	**GLUT4 framework proteins**	**A.375**	The L6 series myotubes stably express the GLUT4myc protein.	Pharmacological perturbation	Tier 3 - pharmacological functional evidence
	**Insulin receptor**	**A.738**	Male Wistar rats (250-300g); white portions of gastrocnemius and quadriceps muscles.	Physiological stimulus	Tier 4 - descriptive/correlative evidence
	**Low molecular weight GTP-binding proteins**	**A.373**	Lean Zucker rats, 14 weeks old.	Physiological stimulus	Tier 4 - descriptive/correlative evidence
	**M-cadherin**	**A.785**	L6-GLUT4myc myoblasts; C57BL/6 mouse skeletal muscle (ex vivo).	Genetic perturbation/rescue + Pharmacological perturbation + Physiological stimulus	Tier 1 - convergent mechanistic evidence
	**metabolic enzymes**	**A.375**	The L6 series myotubes stably express the GLUT4myc protein.	Pharmacological perturbation	Tier 3 - pharmacological functional evidence
	**Microtubule**	**A.461**	Adult mouse muscle fibers: Flexor digitorum brevis (FDB) muscle (for observation of living cells), Soleus muscle, and Extensor digitorum longus (EDL) (for measuring glucose uptake).<br><br>Human muscle: Vastus lateralis muscle from healthy men.<br><br>Cell line: L6 myoblasts/myotubes.	Disease/stress model	Tier 2 - functional mechanistic evidence
		**A.460**	L6 skeletal muscle cells express GLUT4 tagged with c-myc (L6myc cells), which are differentiated into myotubes.	Pharmacological perturbation	Tier 3 - pharmacological functional evidence

	**Myosin Va**	**A.484**	Cells: L6 muscle cells express GLUT4 tagged myc (L6-GLUT4myc), including myoblasts and myotubes.<br><br>Other Cells: CHO cells express insulin receptors (CHO-IR) for lysates.	Genetic perturbation/rescue	Tier 2 - functional mechanistic evidence
	**p300**	**A.510**	Animals: Skeletal muscle-specific p300/CBP knockout mice (PCKO mice) and related compound heterozygous lines.<br><br>Cells: L6 myoblasts/myotubes, 3T3-L1 adipocytes, and primary human skeletal muscle cells.	Genetic perturbation/rescue	Tier 2 - functional mechanistic evidence
	**PAK1**	**A.631**	L6 skeletal muscle cells.	Genetic perturbation/rescue	Tier 2 - functional mechanistic evidence
	**PI(3,4,5)P_3**	**A.418**	Tế bào cơ xương (myotubes) dòng L6 biểu hiện GLUT4myc.<br>Tế bào cơ xương (myotubes) dòng L6 biểu hiện GLUT4myc.<br>L6 myotubes express GLUT4myc.	Genetic perturbation/rescue	Tier 2 - functional mechanistic evidence
	**PI3K**	**A.418**	Tế bào cơ xương (myotubes) dòng L6 biểu hiện GLUT4myc.<br>Tế bào cơ xương (myotubes) dòng L6 biểu hiện GLUT4myc.<br>L6 myotubes express GLUT4myc.	Genetic perturbation/rescue	Tier 2 - functional mechanistic evidence
	**PYK2**	**A.52**	• Animals: C57BL/6J mice (vastus lateralis muscle), Sprague-Dawley rats (extensor digitorum longus – EDL muscle).<br>• Cells: L6 myotubes (rat skeletal muscle cell line).	Genetic perturbation/rescue	Tier 2 - functional mechanistic evidence
	**Rab14**	**A.576**	L6 myoblasts.	Genetic perturbation/rescue	Tier 2 - functional mechanistic evidence
	**Rab4**	**A.186**	• Male Sprague–Dawley rats, weighing 150–175 g.<br>• Studied tissue: hind limb skeletal muscles.	Physiological stimulus	Tier 4 - descriptive/correlative evidence
	**Rab8A**	**A.484**	Cells: L6 muscle cells express GLUT4 tagged myc (L6-GLUT4myc), including myoblasts and myotubes.<br><br>Other Cells: CHO cells express insulin receptors (CHO-IR) for lysates.	Genetic perturbation/rescue	Tier 2 - functional mechanistic evidence

		A.576	L6 myoblasts.	Genetic perturbation/rescue	Tier 2 - functional mechanistic evidence
	Rac1 (Rho family GTPase)	A.166	• Mouse skeletal muscle (gastrocnemius) from wild-type mice and muscle-specific rac1 knockout mice (m-rac1-KO).	Genetic perturbation/rescue	Tier 2 - functional mechanistic evidence
	RalA	A.51	• Mouse 3T3-L1 adipocytes<br>• Rat skeletal muscle cells (L6 myoblasts stably expressing Myc-Glut4).	Genetic perturbation/rescue + Pharmacological perturbation + Physiological stimulus	Tier 1 - convergent mechanistic evidence
	SCAMP37	A.738	Male Wistar rats (250-300g); white portions of gastrocnemius and quadriceps muscles.	Physiological stimulus	Tier 4 - descriptive/correlative evidence
	SCAMP39	A.738	Male Wistar rats (250-300g); white portions of gastrocnemius and quadriceps muscles.	Physiological stimulus	Tier 4 - descriptive/correlative evidence
	Small-molecular-mass GTP-binding proteins	A.382	Lean male Zucker rats	Physiological stimulus	Tier 4 - descriptive/correlative evidence
	TBC1D1	A.41	• Animals: Sprague-Dawley rats.<br>• Models: TBC1D1 knockout (TBC1D1 KO) rats and wild-type (WT) rats.<br>• Tissues: Red gastrocnemius (RG) and white gastrocnemius (WG), extensor digitorum longus (EDL).	Genetic perturbation/rescue + Disease/stress model	Tier 2 - functional mechanistic evidence
	Ubiquitin-degrading system proteins	A.375	The L6 series myotubes stably express the GLUT4myc protein.	Pharmacological perturbation	Tier 3 - pharmacological functional evidence
Tethering	EXOC1	A.698	Rat L6 myoblasts and differentiated myotubes.	Genetic perturbation/rescue	Tier 2 - functional mechanistic evidence
	EXOC5	A.698	Rat L6 myoblasts and differentiated myotubes.	Genetic perturbation/rescue	Tier 2 - functional mechanistic evidence
	EXOC7	A.698	Rat L6 myoblasts and differentiated myotubes.	Genetic perturbation/rescue	Tier 2 - functional mechanistic evidence
	Exocyst complex	A.698	Rat L6 myoblasts and differentiated myotubes.	Genetic perturbation/rescue	Tier 2 - functional mechanistic evidence
	Myo1c	A.480	Cells: L6 myoblasts express myc-tagged GLUT4 (L6-GLUT4myc) or fluorescent-tagged GLUT4 (GLUT4-RFP/GFP).<br>Tissue: Muscle fibers separated from the short toe flexor muscles	Genetic perturbation/rescue + Pharmacological perturbation + Physiological stimulus	Tier 1 - convergent mechanistic evidence

			(FDB fibers) of male rats (12 weeks of age).		
**Docking**	**EXOC5 (a subunit of the exocyst protein trafficking complex)**	**A.195**	• Skeletal muscle–specific Exoc5 knockout mice (Exoc5-SMKO) and littermate control mice.<br>• Mice were generated by crossing Exoc5 floxed mice with HSA-MCM (Human Skeletal Actin Mer-Cre-Mer) mice.	Disease/stress model	Tier 2 - functional mechanistic evidence
	**MICAL-L2**	**A.6**	• Mouse skeletal muscle L6 cells<br>• CHO-IR cells	Genetic perturbation/rescue + Physiological stimulus	Tier 1 - convergent mechanistic evidence
	**Protein Kinase C-ζ (PKC-ζ)**	**A.552**	3T3-L1 adipocytes and L6 muscle cells.	Pharmacological perturbation	Tier 3 - pharmacological functional evidence
	**Rab13**	**A.572**	L6 rat skeletal muscle cells.	Genetic perturbation/rescue	Tier 2 - functional mechanistic evidence
	**Rab8A**	**A.572**	L6 rat skeletal muscle cells.	Genetic perturbation/rescue	Tier 2 - functional mechanistic evidence
**Fusion**	**80K-H**	**A.298**	Cell lines: CHO cells (Cho-K1, Cho-mycGLUT4), 3T3-L1 fat cells, L6 muscle tubes (L6 myotubes), and COS-1 cells.	Genetic perturbation/rescue	Tier 2 - functional mechanistic evidence
	**AMPK**	**A.748**	L6 myoblasts stably expressing myc-tagged GLUT4 (L6-GLUT4-myc).	Genetic perturbation/rescue + Pharmacological perturbation + Physiological stimulus	Tier 1 - convergent mechanistic evidence
	**AS160 (TBC1D4)**	**A.286**	Myoblasts and myotubes of the L6 line stably express Myc-tagged GLUT4 (L6-GLUT4myc).	Genetic perturbation/rescue	Tier 2 - functional mechanistic evidence
	**Complexin-2**	**A.155**	• L6 rat skeletal muscle cells, including both myoblasts and myotube	Genetic perturbation/rescue	Tier 2 - functional mechanistic evidence
	**Csp1 (Cysteine-string protein 1)**	**A.736**	Differentiated 3T3-L1 adipocytes and L6 skeletal muscle cells.	Genetic perturbation/rescue	Tier 2 - functional mechanistic evidence
	**insulin**	**A.366**	Skeletal muscle fibers from transgenic mice express HA-GLUT4-GFP muscle specificity	Physiological stimulus	Tier 4 - descriptive/correlative evidence

	Munc18c	A.473	Cells: Differentiated 3T3-L1 adipocytes (3T3-L1 adipocytes) and CHO cells express insulin receptor stability (CHO/IR).<br><br>Animal tissue: Mouse hind-limb skeletal muscle.<br><br>Recombinant proteins: Recombinant His-Munc18c, GST-Munc18c.	Genetic perturbation/rescue + Pharmacological perturbation + Physiological stimulus	Tier 1 - convergent mechanistic evidence
		A.474	Animals: Transgenic mice express GLUT4-EGFP under the control of human rhabdomyocardial actin promoter (HSA). C57Bl/6 mouse background.<br><br>Tissues: Rhabdomyocardia (quadriceps, twin muscles - gastrocnemius), heart, adipose tissue.	Genetic perturbation/rescue	Tier 2 - functional mechanistic evidence
	Phosphatidic acid (PtdOH / phosphatidic acid)	A.81	• Mouse 3T3-L1 adipocytes<br>• L6 myoblasts	Genetic perturbation/rescue	Tier 2 - functional mechanistic evidence
	PI(3,4,5)P3	A.355	GLUT4myc cells: Mouse muscle cells differentiate into myotubes with stable expression of GLUT4myc fusion protein	Pharmacological perturbation	Tier 3 - pharmacological functional evidence
	PI3K class IA	A.355	GLUT4myc cells: Mouse muscle cells differentiate into myotubes with stable expression of GLUT4myc fusion protein	Pharmacological perturbation	Tier 3 - pharmacological functional evidence
	PI3P	A.355	GLUT4myc cells: Mouse muscle cells differentiate into myotubes with stable expression of GLUT4myc fusion protein	Pharmacological perturbation	Tier 3 - pharmacological functional evidence
	PKCζ	A.50	• Primary rat skeletal muscle cells.	Genetic perturbation/rescue	Tier 2 - functional mechanistic evidence
		A.298	Cell lines: CHO cells (Cho-K1, Cho-mycGLUT4), 3T3-L1 fat cells, L6 muscle tubes (L6 myotubes), and COS-1 cells.	Genetic perturbation/rescue	Tier 2 - functional mechanistic evidence
	Rab8A	A.286	Myoblasts and myotubes of the L6 line stably express Myc-tagged GLUT4 (L6-GLUT4myc).	Genetic perturbation/rescue	Tier 2 - functional mechanistic evidence
		A.573	C2C12 mouse myoblasts.	Genetic perturbation/rescue + Physiological stimulus	Tier 1 - convergent mechanistic evidence
	Rac	A.286	Myoblasts and myotubes of the L6 line stably express Myc-tagged GLUT4 (L6-GLUT4myc).	Genetic perturbation/rescue	Tier 2 - functional mechanistic evidence

	SNAP25	A.396	human skeletal muscle tissue and adipose tissue; mouse 3T3-L1 fat cell line; PC12 cells (positive control).	Physiological stimulus	Tier 4 - descriptive/correlative evidence
	Synaptobrevin 1	A.396	human skeletal muscle tissue and adipose tissue; mouse 3T3-L1 fat cell line; PC12 cells (positive control).	Physiological stimulus	Tier 4 - descriptive/correlative evidence
	Syntaxin 4	A.396	human skeletal muscle tissue and adipose tissue; mouse 3T3-L1 fat cell line; PC12 cells (positive control).	Physiological stimulus	Tier 4 - descriptive/correlative evidence
		A.669	Transgenic mice overexpressing Syntaxin 4 on a C57BL/6J background (both sexes, 4-6 months old).	Genetic perturbation/rescue	Tier 2 - functional mechanistic evidence
	Tropomyosin Tpm3.1	A.75	• Differentiated 3T3-L1 adipocytes<br>• Mice (C57BL/6 and FVB/N strains), including transgenic mice overexpressing Tpm3.1 (Tg) and Tpm3.1 knockout (KO) mice<br>• Mouse white adipose tissue (WAT)<br>• Mouse skeletal muscle	Genetic perturbation/rescue + Pharmacological perturbation + Physiological stimulus	Tier 1 - convergent mechanistic evidence
	VAMP2 (Synaptobrevin 2)	A.396	human skeletal muscle tissue and adipose tissue; mouse 3T3-L1 fat cell line; PC12 cells (positive control).	Physiological stimulus	Tier 4 - descriptive/correlative evidence
		A.772	L6-GLUT4myc myoblasts.	Genetic perturbation/rescue	Tier 2 - functional mechanistic evidence
	VAMP3 (Cellubrevin)	A.772	L6-GLUT4myc myoblasts.	Genetic perturbation/rescue	Tier 2 - functional mechanistic evidence
	VAP-33	A.9	• Rat skeletal muscle cells (L6 skeletal myoblasts) stably expressing myc-tagged GLUT4 (L6-GLUT4myc).<br>• Mouse adipose cells (3T3-L1 adipocytes).	Genetic perturbation/rescue + Physiological stimulus	Tier 1 - convergent mechanistic evidence
