# Supplementary material for: Molecular architecture of GLUT4 trafficking and exocytosis in adipocytes and skeletal muscle: A systematic review and integrated conceptual framework
Source: Front Endocrinol (Lausanne). 2026 Oct 19;17:1932455. doi: 10.3389/fendo.2026.1932455 (PMC13725575; doi:10.3389/fendo.2026.1932455)
Supplement: Supplementary file 4 [file SupplementaryFile1.docx]

**Supplementary File 1: Detailed Search Strategy**

**Table of Contents**

1. Introduction
2. Databases and Other Information Sources
3. Search Strategies by Database
    3.1. PubMed (MEDLINE)
    3.2. Embase (via Embase.com)
    3.3. Scopus (via Scopus.com)
    3.4. Additional Sources (Grey Literature, Hand Searching)
4. Search Results Management

**1. Introduction**

This document provides a comprehensive overview of the electronic search strategies applied to identify relevant literature for our systematic review. The strategy was designed using the PECO (Population, Exposure, Comparator, Outcome) framework and refined in consultation with an experienced health sciences librarian. It adheres to the PRISMA-S extension for systematic review search reporting.

We aimed to ensure reproducibility, transparency, and comprehensiveness by including both controlled vocabulary (e.g., MeSH, Emtree) and free-text terms, combined using Boolean operators. The search was designed to retrieve peer-reviewed studies and grey literature where relevant.

**PECO Framework:**

- **Population (P):** In vitro adipocyte and skeletal muscle cell models, as well as ex vivo and in vivo animal models expressing endogenous or recombinant GLUT4.
- **Exposure/Intervention (E):** Experimental manipulations designed to activate, inhibit, or modulate GLUT4 exocytosis (e.g., insulin stimulation, contraction or AICAR treatment, pharmacological inhibition, genetic manipulation).
- **Comparator (C):** No specific comparator required.
- **Outcome (O):** Molecular components, functional roles, and regulatory mechanisms involved in the exocytotic trafficking of GLUT4 (vesicle mobilization, tethering, docking, membrane fusion).

**Study Types Included:** Eligible primary experimental studies.

**Date Range:** From inception to August 10, 2025.
**Language Restrictions:** Publications available in English
**Protocol Registration:** PROSPERO CRD420251105528

**2. Databases and Information Sources**

We systematically searched the following databases:

- **PubMed (MEDLINE)**
- **Embase (via Embase.com)**
- **Scopus (via Scopus.com)**

Academic Search Engines:

- Google Scholar: We will screen the first 200 results per search string in Google Scholar, consistent with the methodology suggested by Haddaway et al. (2015) for grey literature saturation.

Biological Preprint Servers: Focused searches on biology-specific preprint repositories such as bioRxiv, where molecular mechanism studies are often published prior to peer review.

Dissertations and Theses: Searching the ProQuest Dissertations & Theses Global database, which contains highly detailed original data.

**3. Search Strategies by Database**

**3.1. PubMed (MEDLINE)**

**Search Date:** Aug 10,2025

| **Component** | **Search Terms** |
| --- | --- |
| **Population** | ( "Glucose Transporter Type 4"[Mesh] OR GLUT4[tiab] OR SLC2A4[tiab] OR "GLUT4 storage vesicle"[tiab] OR "GSV"[tiab]) |
| **Exposure** | None |
| **Comparator** | None |
| **Outcome** | ( "Protein Transport"[Mesh] OR "Exocytosis"[Mesh] OR "Biological Transport"[Mesh] OR trafficking[tiab] OR translocation[tiab] OR translocate[tiab] OR recruitment[tiab] OR mobilization[tiab] OR exocytosis[tiab] OR docking[tiab] OR fusion[tiab] OR tethering[tiab]) |
| **Study Type** | NOT( "review"[Publication Type] ) |
| **Limits** | ( english[lang] ) |
| **Combined** | P AND O S AND Limits |
| **Results** | 3689 |

**Syntax:**

( "Glucose Transporter Type 4"[Mesh] OR GLUT4[tiab] OR SLC2A4[tiab] OR "GLUT4 storage vesicle"[tiab] OR "GSV"[tiab])

AND

( "Protein Transport"[Mesh] OR "Exocytosis"[Mesh] OR "Biological Transport"[Mesh] OR trafficking[tiab] OR translocation[tiab] OR translocate[tiab] OR recruitment[tiab] OR mobilization[tiab] OR exocytosis[tiab] OR docking[tiab] OR fusion[tiab] OR tethering[tiab])

AND

( english[lang] )

NOT

("Review"[Publication Type] )

**3.2. Embase (via Ovid)**

**Search Date:** Aug 10, 2025

| **Component** | **Search Terms** |
| --- | --- |
| **Population** | ('glucose transporter type 4'/exp OR glut4:ti,ab OR slc2a4:ti,ab OR "glut4 storage vesicle":ti,ab OR gsv:ti,ab) |
| **Exposure** | None |
| **Comparator** | None |
| **Outcome** | ('protein transport'/exp OR 'exocytosis'/exp OR 'biological transport'/exp OR trafficking:ti,ab OR translocation:ti,ab OR translocate:ti,ab OR recruitment:ti,ab OR mobilization:ti,ab OR exocytosis:ti,ab OR docking:ti,ab OR fusion:ti,ab OR tethering:ti,ab) |
| **Study Type** | NOT  'review'/it |
| **Limits** | ([english]/lim) |
| **Results** | 8100 |

**Example Syntax:**

('glucose transporter type 4'/exp OR glut4:ti,ab OR slc2a4:ti,ab OR "glut4 storage vesicle":ti,ab OR gsv:ti,ab)

AND

('protein transport'/exp OR 'exocytosis'/exp OR 'biological transport'/exp OR trafficking:ti,ab OR translocation:ti,ab OR translocate:ti,ab OR recruitment:ti,ab OR mobilization:ti,ab OR exocytosis:ti,ab OR docking:ti,ab OR fusion:ti,ab OR tethering:ti,ab)

AND

([english]/lim)

NOT

'review'/it

**3.3. Scopus**

**Search Date:** Aug 10, 2025

| **Component** | **Search Terms** |
| --- | --- |
| **Population** | ( TITLE-ABS-KEY("Glucose Transporter Type 4")  OR TITLE-ABS-KEY(GLUT4)  OR TITLE-ABS-KEY(SLC2A4)  OR TITLE-ABS-KEY("GLUT4 storage vesicle")  OR TITLE-ABS-KEY(GSV)  ) |
| **Exposure** | NONE |
| **Comparator** | NONE |
| **Outcome** | ( TITLE-ABS-KEY("Protein Transport")  OR TITLE-ABS-KEY("Exocytosis")  OR TITLE-ABS-KEY("Biological Transport")  OR TITLE-ABS-KEY(trafficking)  OR TITLE-ABS-KEY(translocation)  OR TITLE-ABS-KEY(translocate)  OR TITLE-ABS-KEY(recruitment)  OR TITLE-ABS-KEY(mobilization)  OR TITLE-ABS-KEY(exocytosis)  OR TITLE-ABS-KEY(docking)  OR TITLE-ABS-KEY(fusion)  OR TITLE-ABS-KEY(tethering)  ) |
| **Study Type** | NOT  (  DOCTYPE("re")  ) |
| **Filters** | ( LANGUAGE(English) ) |
| **Results** | 3723 |

( TITLE-ABS-KEY("Glucose Transporter Type 4")

OR TITLE-ABS-KEY(GLUT4)

OR TITLE-ABS-KEY(SLC2A4)

OR TITLE-ABS-KEY("GLUT4 storage vesicle")

OR TITLE-ABS-KEY(GSV)

)

AND

( TITLE-ABS-KEY("Protein Transport")

OR TITLE-ABS-KEY("Exocytosis")

OR TITLE-ABS-KEY("Biological Transport")

OR TITLE-ABS-KEY(trafficking)

OR TITLE-ABS-KEY(translocation)

OR TITLE-ABS-KEY(translocate)

OR TITLE-ABS-KEY(recruitment)

OR TITLE-ABS-KEY(mobilization)

OR TITLE-ABS-KEY(exocytosis)

OR TITLE-ABS-KEY(docking)

OR TITLE-ABS-KEY(fusion)

OR TITLE-ABS-KEY(tethering)

)

AND

( LANGUAGE(English) )

AND NOT DOCTYPE(re)

**3.4. Google Scholar**

**Search Date:** Aug 10, 2025

| **Component** | **Search Terms** |
| --- | --- |
| **Population** | (("Glucose Transporter Type 4" OR GLUT4 OR SLC2A4 OR "GLUT4 storage vesicle" OR GSV) |
| **Exposure** | NONE |
| **Comparator** | NONE |
| **Outcome** | ("Protein Transport" OR "Exocytosis" OR "Biological Transport" OR trafficking OR translocation OR translocate OR recruitment OR mobilization OR exocytosis OR docking OR fusion OR tethering)) |
| **Study Type** | NONE |
| **Filters** | NONE |
| **Results** | 200 |

allintitle: (("Glucose Transporter Type 4" OR GLUT4 OR SLC2A4 OR "GLUT4 storage vesicle" OR GSV) AND ("Protein Transport" OR "Exocytosis" OR "Biological Transport" OR trafficking OR translocation OR translocate OR recruitment OR mobilization OR exocytosis OR docking OR fusion OR tethering))

**3.5. Additional Sources**

| **Source** | **Date** | **Terms Used / Notes** | **Results** |
| --- | --- | --- | --- |
| **BioRxiv** | 10/8/2025 | ("Glucose Transporter Type 4" OR GLUT4 OR SLC2A4 OR "GLUT4 storage vesicle" OR GSV)  AND  ("Protein Transport" OR Exocytosis OR "Biological Transport" OR trafficking OR translocation OR translocate OR recruitment OR mobilization OR exocytosis OR docking OR fusion OR tethering)  NOT Review | 120 |
| **ProQuest Dissertations & Theses** | 10/8/2025 | ti,ab("Glucose Transporter Type 4" OR GLUT4 OR SLC2A4 OR "GLUT4 storage vesicle" OR GSV)  AND  ti,ab("Protein Transport" OR "Exocytosis" OR "Biological Transport" OR trafficking OR translocation OR translocate OR recruitment OR mobilization OR exocytosis OR docking OR fusion OR tethering) | 3 |

**4. Search Results Management**

All retrieved references were imported into EndNote then uploaded to Rayyan. Automatic and manual deduplication was performed. The deduplicated dataset was then uploaded to Rayyan for title/abstract and full-text screening by two independent reviewers, with a third reviewer resolving disagreements.

The number of records identified, screened, excluded, and included is reported in the main manuscript and illustrated in the PRISMA 2020 flow diagram.
