# Supplementary material for: Molecular architecture of GLUT4 trafficking and exocytosis in adipocytes and skeletal muscle: A systematic review and integrated conceptual framework
Source: Front Endocrinol (Lausanne). 2026 Oct 19;17:1932455. doi: 10.3389/fendo.2026.1932455 (PMC13725575; doi:10.3389/fendo.2026.1932455)
Supplement: Supplementary file 5 [file SupplementaryFile2.docx]

**Supplementary File 2:** Full-text reports excluded from the final evidence synthesis

| **CODE** | **TITLE** | **DOI** | **Primary reason for the exclusion** | **Categories** |
| --- | --- | --- | --- | --- |
| A.3 | 5′-AMP-activated protein kinase increases glucose uptake independent of GLUT4 translocation in cardiac myocytes | 10.1139/cjpp-2013-0107 | Investigated a cardiac/cardiomyocyte or heart model; the final review scope was restricted to adipocyte and skeletal-muscle models. | **Wrong population/model** |
| A.5 | A carboxy-terminal deletion mutant of protein kinase CβII inhibits insulin-stimulated 2-deoxyglucose uptake in L6 rat skeletal muscle cells | 10.1210/me.10.10.1273 | Did not directly assess a GLUT4 trafficking/exocytosis endpoint (vesicle mobilization, surface insertion, tethering, docking, fusion, or subcellular redistribution); it evaluated glucose uptake, signalling, expression, or a systemic phenotype only. | **Wrong outcome** |
| A.11 | A myosin-derived peptide C109 binds to GLUT4-vesicles and inhibits the insulin-induced glucose transport stimulation and GLUT4 recruitment in rat adipocytes | 10.1006/bbrc.1997.7671 | Did not provide a qualifying functional link between a molecular regulator and a defined GLUT4 exocytosis/trafficking step in the prespecified model scope. | **Wrong outcome** |
| A.14 | A novel akt substrate containing C2 domain regulates GLUT4 insertion into the plasma membrane | 10.2337/db11-1-378 | Conference abstract or meeting record without sufficiently detailed, full primary experimental methods and results for eligibility assessment. | **Ineligible publication type / insufficient primary data** |
| A.15 | A novel anchor protein tug mediates GLUT4 translocation by amp-activated protein kinase in the ischemic heart |  | Investigated a cardiac/cardiomyocyte or heart model; the final review scope was restricted to adipocyte and skeletal-muscle models. | **Wrong population/model** |
| A.17 | A novel method to monitor insulin-stimulated GTP-loading of Rab11a in cardiomyocytes | 10.1016/j.cellsig.2006.10.008 | Population: Mouse cardiomyocyte cell line H9c2-hIR stably expressing the human insulin receptor. | **Wrong population** |
| A.21 | A novel target for combating insulin resistance |  | Did not provide a qualifying functional link between a molecular regulator and a defined GLUT4 exocytosis/trafficking step in the prespecified model scope. | **Wrong outcome** |
| A.22 | A peroxovanadium compound stimulates muscle glucose transport as powerfully as insulin and contractions combined | 10.2337/diabetes.52.8.1918 | Did not provide a qualifying functional link between a molecular regulator and a defined GLUT4 exocytosis/trafficking step in the prespecified model scope. | **Wrong outcome** |
| A.23 | A phosphotyrosyl mimetic peptide reverses impairment of insulin- stimulated translocation of GLUT4 caused by overexpression of PTP1B in rat adipose cells | 10.1021/bi9816103 | Did not provide a qualifying functional link between a molecular regulator and a defined GLUT4 exocytosis/trafficking step in the prespecified model scope. | **Wrong outcome** |
| A.28 | A robust assay measuring GLUT4 translocation in rat myoblasts overexpressing GLUT4-myc and AS160_v2 | 10.1016/j.ab.2009.10.036 | Methods/assay report that did not test a molecular regulator or regulatory mechanism of GLUT4 trafficking in an eligible primary experimental study. | **Wrong study design** |
| A.30 | A role for EFR3 in insulin action? | 10.1111/dme.5_13048 | Conference abstract or meeting record without sufficiently detailed, full primary experimental methods and results for eligibility assessment. | **Ineligible publication type / insufficient primary data** |
| A.32 | A role for phospholipase D in GLUT4 glucose transporter translocation | 10.1074/jbc.275.10.7144 | Reported a GLUT4 trafficking observation but did not test an eligible molecular regulator or regulatory mechanism with a defined trafficking endpoint. | **Wrong outcome** |
| A.33 | A role for protein kinase Bβ/Akt2 in insulin-stimulated GLUT4 translocation in adipocytes | 10.1128/mcb.19.11.7771 | Did not provide a qualifying functional link between a molecular regulator and a defined GLUT4 exocytosis/trafficking step in the prespecified model scope. | **Wrong outcome** |
| A.34 | A role for Raf-1 in the divergent signaling pathways mediating insulin-stimulated glucose transport | 10.1016/s0021-9258(17)36999-5 | Did not provide a qualifying functional link between a molecular regulator and a defined GLUT4 exocytosis/trafficking step in the prespecified model scope. | **Wrong outcome** |
| A.35 | A SNARE protein P34 regulates the intracellular sequestration of glut4 vesicles in adipocytes | 10.2337/db12-1578-1720 | Conference abstract or meeting record without sufficiently detailed, full primary experimental methods and results for eligibility assessment. | **Ineligible publication type / insufficient primary data** |
| A.36 | A specific dileucine motif is required for the GGA-dependent entry of newly synthesized insulin-responsive aminopeptidase into the insulin-responsive compartment | 10.1074/jbc.M601583200 | Did not directly assess a GLUT4 trafficking/exocytosis endpoint (vesicle mobilization, surface insertion, tethering, docking, fusion, or subcellular redistribution); it evaluated glucose uptake, signalling, expression, or a systemic phenotype only. | **Wrong outcome** |
| A.37 | A steady state analysis indicates that negative feedback regulation of PTP1B by Akt elicits bistability in insulin-stimulated GLUT4 translocation | 10.1186/1742-4682-1-2 | Computational, structural, or mathematical analysis without experimental validation in an eligible GLUT4-trafficking model. | **Wrong study design** |
| A.39 | A Tether Containing a UBX Domain (TUG) Mediates Glucose Transporter Translocation in the Ischemic Heart | 10.1161/res.121.suppl_1.158 | Investigated a cardiac/cardiomyocyte or heart model; the final review scope was restricted to adipocyte and skeletal-muscle models. | **Wrong population/model** |
| A.40 | A type IV P-type ATPase affects insulin-mediated glucose uptake in adipose tissue and skeletal muscle in mice | 10.1016/j.jnutbio.2006.01.002 | Did not directly assess a GLUT4 trafficking/exocytosis endpoint (vesicle mobilization, surface insertion, tethering, docking, fusion, or subcellular redistribution); it evaluated glucose uptake, signalling, expression, or a systemic phenotype only. | **Wrong outcome** |
| A.43 | Actin cytoskeletal remodeling in the regulation of glucose homeostasis | 10.1364/PIBM.2017.T1B.2 | Conference abstract or meeting record without sufficiently detailed, full primary experimental methods and results for eligibility assessment. | **Ineligible publication type / insufficient primary data** |
| A.44 | Actin filaments participate in the relocalization of phosphatidylinositol 3-kinase to glucose transporter-containing compartments and in the stimulation of glucose uptake in 3T3-L1 adipocytes | 10.1042/bj3310917 | Did not provide a qualifying functional link between a molecular regulator and a defined GLUT4 exocytosis/trafficking step in the prespecified model scope. | **Wrong outcome** |
| A.47 | Activation of foxo1 induces akt phosphorylation and mimics the metabolic functions of insulin without proliferative effects on 3T3-L1 adipocytes | 10.2337/db21-121-OR | Conference abstract or meeting record without sufficiently detailed, full primary experimental methods and results for eligibility assessment. | **Ineligible publication type / insufficient primary data** |
| A.48 | Activation of glucose transport during simulated ischemia in H9c2 cardiac myoblasts is mediated by protein kinase C isoforms | 10.1016/j.lfs.2005.04.039 | Investigated a cardiac/cardiomyocyte or heart model; the final review scope was restricted to adipocyte and skeletal-muscle models. | **Wrong population/model** |
| A.49 | Activation of phosphatidylinositol-3 kinase, AMP-activated kinase and Akt substrate-160 kDa by trans-10, cis-12 conjugated linoleic acid mediates skeletal muscle glucose uptake | 10.1016/j.jnutbio.2012.01.006 | Did not directly assess a GLUT4 trafficking/exocytosis endpoint (vesicle mobilization, surface insertion, tethering, docking, fusion, or subcellular redistribution); it evaluated glucose uptake, signalling, expression, or a systemic phenotype only. | **Wrong outcome** |
| A.55 | ADIPOSE CLC-3 DEFICIENCY PROTECTS AGAINST DIET-INDUCED OBESITY AND INSULIN RESISTANCE BY REGULATING GLUT4 THROUGH THE PI3K/AKT PATHWAY | 10.1016/j.fertnstert.2021.07.229 | Did not directly assess a GLUT4 trafficking/exocytosis endpoint (vesicle mobilization, surface insertion, tethering, docking, fusion, or subcellular redistribution); it evaluated glucose uptake, signalling, expression, or a systemic phenotype only. | **Wrong outcome** |
| A.56 | Adipose RAB10 knockout induces insulin intolerance | 10.2337/db1518471928 | Did not provide a qualifying functional link between a molecular regulator and a defined GLUT4 exocytosis/trafficking step in the prespecified model scope. | **Wrong outcome** |
| A.58 | Aerobic glycolysis is a major regulator of Akt activity | 10.1007/s00125-012-2688-9 | Did not provide a qualifying functional link between a molecular regulator and a defined GLUT4 exocytosis/trafficking step in the prespecified model scope. | **Wrong outcome** |
| A.63 | Akt1 and Akt2 exhibit isoform-specific functions in HL-1 cardiomyocytes |  | Investigated a cardiac/cardiomyocyte or heart model; the final review scope was restricted to adipocyte and skeletal-muscle models. | **Wrong population/model** |
| A.68 | Amino Acids Potentiate Insulin Signaling in CHO-K1 at High Glucose Conditions | 10.1016/j.arcmed.2012.04.008 | Did not directly assess a GLUT4 trafficking/exocytosis endpoint (vesicle mobilization, surface insertion, tethering, docking, fusion, or subcellular redistribution); it evaluated glucose uptake, signalling, expression, or a systemic phenotype only. | **Wrong outcome** |
| A.69 | AMP kinase activation and glut4 translocation in isolated cardiomyocytes |  | Population: Isolated adult ventricular cardiomyocytes. | **Wrong population** |
| A.70 | AMPK activation by prolonged stimulation with interleukin-1β contributes to the promotion of GLUT4 translocation in skeletal muscle cells | 10.1002/cbin.10673 | Did not provide a qualifying functional link between a molecular regulator and a defined GLUT4 exocytosis/trafficking step in the prespecified model scope. | **Wrong outcome** |
| A.71 | AMPK and the RabGAPs TBC1D1 and TBC1D4 are necessary but not sufficient for contraction-mediated glucose metabolism in skeletal muscle | 10.1007/s00125-020-05221-5 | Did not provide a qualifying functional link between a molecular regulator and a defined GLUT4 exocytosis/trafficking step in the prespecified model scope. | **Wrong outcome** |
| A.72 | AMPK enhances insulin and GLUT4 action in L6 myotubes via lowering plasma membrane cholesterol |  | Did not provide a qualifying functional link between a molecular regulator and a defined GLUT4 exocytosis/trafficking step in the prespecified model scope. | **Wrong outcome** |
| A.73 | AMPK/AS 160 mediates tiliroside derivatives-stimulated GLUT4 translocation in muscle cells | 10.2147/DDDT.S164441 | Did not provide a qualifying functional link between a molecular regulator and a defined GLUT4 exocytosis/trafficking step in the prespecified model scope. | **Wrong outcome** |
| A.77 | An efficient co-expression and purification system for the complex of Stx4 and C-terminal domain of Synip | 10.1016/j.bbrc.2008.04.067 | Did not directly assess a GLUT4 trafficking/exocytosis endpoint (vesicle mobilization, surface insertion, tethering, docking, fusion, or subcellular redistribution); it evaluated glucose uptake, signalling, expression, or a systemic phenotype only. | **Wrong outcome** |
| A.78 | An inhibitor of p38 mitogen-activated protein kinase prevents insulin- stimulated glucose transport but not glucose transporter translocation in 3T3-L1 adipocytes and L6 myotubes | 10.1074/jbc.274.15.10071 | Did not provide a qualifying functional link between a molecular regulator and a defined GLUT4 exocytosis/trafficking step in the prespecified model scope. | **Wrong outcome** |
| A.79 | An inositol 1,4,5-triphosphate (IP3)-IP3 receptor pathway is required for insulin-stimulated glucose transporter 4 translocation and glucose uptake in cardiomyocytes | 10.1210/en.2010-0116 | Population: Primary neonatal rat cardiomyocytes | **Wrong population** |
| A.80 | An insulin-related peptide expressed in 3T3L1 adipocytes is localized in GLUT4 vesicles and secreted in response to exogenous insulin, which augments the insulin-stimulated glucose uptake | 10.1242/jcs.00178 | Reported a GLUT4 trafficking observation but did not test an eligible molecular regulator or regulatory mechanism with a defined trafficking endpoint. | **Wrong outcome** |
| A.82 | Analysis of GLUT4 distribution in whole skeletal muscle fibers: Identification of distinct storage compartments that are recruited by insulin and muscle contractions | 10.1083/jcb.142.6.1429 | Reported a GLUT4 trafficking observation but did not test an eligible molecular regulator or regulatory mechanism with a defined trafficking endpoint. | **Wrong outcome** |
| A.84 | APPL1 affects on glucose uptake induced by uniaxial stretch in C2C12 myotubes | 10.1007/s00125-014-3355-0 | Did not provide a qualifying functional link between a molecular regulator and a defined GLUT4 exocytosis/trafficking step in the prespecified model scope. | **Wrong outcome** |
| A.86 | Are tyrosine kinases involved in mediating contraction-stimulated muscle glucose transport? | 10.1152/ajpendo.00280.2005 | Did not directly assess a GLUT4 trafficking/exocytosis endpoint (vesicle mobilization, surface insertion, tethering, docking, fusion, or subcellular redistribution); it evaluated glucose uptake, signalling, expression, or a systemic phenotype only. | **Wrong outcome** |
| A.89 | Arrest of endosome acidification by bafilomycin A1 mimics insulin action on GLUT4 translocation in 3T3-L1 adipocytes | 10.1042/0264-6021:3390599 | Did not provide a qualifying functional link between a molecular regulator and a defined GLUT4 exocytosis/trafficking step in the prespecified model scope. | **Wrong outcome** |
| A.90 | AS160 is required for sequestration of Glut4 in non-cycling storage compartments while the rate constant for Glut4 exocytosis is regulated by an additional unidentified Akt substrate |  | Did not provide a qualifying functional link between a molecular regulator and a defined GLUT4 exocytosis/trafficking step in the prespecified model scope. | **Wrong outcome** |
| A.92 | AS160 regulates glucose uptake in the heart | 10.2337/db12-1-377 | Investigated a cardiac/cardiomyocyte or heart model; the final review scope was restricted to adipocyte and skeletal-muscle models. | **Wrong population/model** |
| A.94 | AS160 Ser711 phosphorylation is impaired in skeletal muscle from insulin resistant subjects |  | Human-only, non-target tissue, or non-eligible biological model; no qualifying mechanistic validation in adipocytes or skeletal muscle was reported. | **Wrong population/model** |
| A.95 | AS160 vs Tbc1d1: Cooperative determination of insulin-responsive GLUT4 trafficking activity after exercise-mimetic stimuli | 10.1007/s00125-015-3687-4 | Did not provide a qualifying functional link between a molecular regulator and a defined GLUT4 exocytosis/trafficking step in the prespecified model scope. | **Wrong outcome** |
| A.97 | Assay of insulin-stimulated signaling by flow cytometry: Key points of regulation |  | Did not provide a qualifying functional link between a molecular regulator and a defined GLUT4 exocytosis/trafficking step in the prespecified model scope. | **Wrong outcome** |
| A.98 | Assembly-promoting protein Munc18c stimulates SNARE-dependent membrane fusion through its SNARE-like peptide | 10.1016/j.jbc.2022.102470 | Did not directly assess a GLUT4 trafficking/exocytosis endpoint (vesicle mobilization, surface insertion, tethering, docking, fusion, or subcellular redistribution); it evaluated glucose uptake, signalling, expression, or a systemic phenotype only. | **Wrong outcome** |
| A.103 | Atbp, adipocyte-specific tubulin-binding protein, can control cytoskeletal structure and insulin stimulated glucose uptake | 10.2337/db1518471928 | Did not provide a qualifying functional link between a molecular regulator and a defined GLUT4 exocytosis/trafficking step in the prespecified model scope. | **Wrong outcome** |
| A.105 | ATP-sensitive binding of a 70-kDa cytosolic protein to the glucose transporter in rat adipocytes | 10.1074/jbc.270.14.7869 | Did not provide a qualifying functional link between a molecular regulator and a defined GLUT4 exocytosis/trafficking step in the prespecified model scope. | **Wrong outcome** |
| A.107 | BAG6 contributes to glucose uptake by supporting the cell surface translocation of the glucose transporter GLUT4 | 10.1242/BIO.047324 | Did not provide a qualifying functional link between a molecular regulator and a defined GLUT4 exocytosis/trafficking step in the prespecified model scope. | **Wrong outcome** |
| A.108 | Beta-catenin is a key player in insulin-stimulated skeletal muscle glucose transport | 10.2337/db19-1773-P | Conference abstract or meeting record without sufficiently detailed, full primary experimental methods and results for eligibility assessment. | **Ineligible publication type / insufficient primary data** |
| A.109 | Biphasic effects of chronic ethanol exposure on insulin-stimulated glucose uptake in primary cultured rat skeletal muscle cells: Role of the Akt pathway and GLUT4 | 10.1002/dmrr.1152 | Did not directly assess a GLUT4 trafficking/exocytosis endpoint (vesicle mobilization, surface insertion, tethering, docking, fusion, or subcellular redistribution); it evaluated glucose uptake, signalling, expression, or a systemic phenotype only. | **Wrong outcome** |
| A.110 | Botulinum neurotoxin B inhibits insulin-stimulated glucose uptake into 3T3-L1 adipocytes and cleaves cellubrevin unlike type A toxin which failed to proteolyze the SNAP-23 present | 10.1021/bi962331n | Did not provide a qualifying functional link between a molecular regulator and a defined GLUT4 exocytosis/trafficking step in the prespecified model scope. | **Wrong outcome** |
| A.111 | Bradykinin directly GLUT4 translocation via an insulin-independent pathway | 10.2337/diabetes.47.4.550 | Did not provide a qualifying functional link between a molecular regulator and a defined GLUT4 exocytosis/trafficking step in the prespecified model scope. | **Wrong outcome** |
| A.119 | CAP defines a second signalling pathway required for insulin-stimulated glucose transport | 10.1038/35025089 | Did not directly assess a GLUT4 trafficking/exocytosis endpoint (vesicle mobilization, surface insertion, tethering, docking, fusion, or subcellular redistribution); it evaluated glucose uptake, signalling, expression, or a systemic phenotype only. | **Wrong outcome** |
| A.120 | Carbohydrate response element binding protein (ChREBP) Regulates insulin-stimulated GLUT4 exocytosis in adipocytes independent of AKT | K có bài báo này | No citable primary full report could be verified from the submitted title/identifier; the record could not be matched to an eligible experimental article. | **Ineligible publication type / insufficient primary data** |
| A.121 | Cardiac PI3K-Akt impairs insulin-stimulated glucose uptake independent of mTORC1 and GLUT4 translocation | 10.1210/me.2012-1210 | Investigated a cardiac/cardiomyocyte or heart model; the final review scope was restricted to adipocyte and skeletal-muscle models. | **Wrong population/model** |
| A.122 | Cardiac SNARE Expression in Health and Disease | 10.3389/fendo.2019.00881 | Investigated a cardiac/cardiomyocyte or heart model; the final review scope was restricted to adipocyte and skeletal-muscle models. | **Wrong population/model** |
| A.123 | Carrier-mediated trans-membrane delivery of PIP3 overcomes cellular insulin resistance induced by proximal, but not distal signaling defect | 10.1007/s00125-010-1872-z | Did not provide a qualifying functional link between a molecular regulator and a defined GLUT4 exocytosis/trafficking step in the prespecified model scope. | **Wrong outcome** |
| A.124 | Catestatin Induces Glucose Uptake and GLUT4 Trafficking in Adult Rat Cardiomyocytes | 10.1155/2018/2086109 | Investigated a cardiac/cardiomyocyte or heart model; the final review scope was restricted to adipocyte and skeletal-muscle models. | **Wrong population/model** |
| A.125 | Caveolin-associated filamentous actin (Cav-actin) defines a novel F-actin structure in adipocytes | 10.1074/jbc.C200292200 | Did not provide a qualifying functional link between a molecular regulator and a defined GLUT4 exocytosis/trafficking step in the prespecified model scope. | **Wrong outcome** |
| A.126 | CCAAT/enhancer binding protein α maintains the ability of insulin-stimulated GLUT4 translocation in 3T3-C2 fibroblastic cells | 10.1016/j.bbamcr.2004.12.007 | Did not provide a qualifying functional link between a molecular regulator and a defined GLUT4 exocytosis/trafficking step in the prespecified model scope. | **Wrong outcome** |
| A.129 | Cellular depletion of atypical PKCλ is associated with enhanced insulin sensitivity and glucose uptake in L6 rat skeletal muscle cells | 10.1152/ajpendo.00171.2010 | Did not directly assess a GLUT4 trafficking/exocytosis endpoint (vesicle mobilization, surface insertion, tethering, docking, fusion, or subcellular redistribution); it evaluated glucose uptake, signalling, expression, or a systemic phenotype only. | **Wrong outcome** |
| A.130 | Changes in Skeletal Muscle PAK1 Levels Regulate Tissue Crosstalk to Impact Whole Body Glucose Homeostasis | 10.3389/fendo.2022.821849 | Did not provide a qualifying functional link between a molecular regulator and a defined GLUT4 exocytosis/trafficking step in the prespecified model scope. | **Wrong outcome** |
| A.131 | Characterising the role of Rab3 in adipose tissue insulin resistance and its link to chronic inflammation | 10.1111/dme.14245 | Did not provide a qualifying functional link between a molecular regulator and a defined GLUT4 exocytosis/trafficking step in the prespecified model scope. | **Wrong outcome** |
| A.132 | Characterization of a major protein in GLUT4 vesicles. Concentration in the vesicles and insulin-stimulated translocation to the plasma membrane |  | Reported a GLUT4 trafficking observation but did not test an eligible molecular regulator or regulatory mechanism with a defined trafficking endpoint. | **Wrong outcome** |
| A.133 | Characterization of GLUT4-containing vesicles in 3T3-L1 adipocytes by total internal reflection fluorescence microscopy | 10.1007/s11427-009-0081-9 | Did not provide a qualifying functional link between a molecular regulator and a defined GLUT4 exocytosis/trafficking step in the prespecified model scope. | **Wrong outcome** |
| A.134 | Characterization of the insulin-regulated membrane aminopeptidase in 3T3- L1 adipocytes | 10.1074/jbc.271.6.3328 | Did not provide a qualifying functional link between a molecular regulator and a defined GLUT4 exocytosis/trafficking step in the prespecified model scope. | **Wrong outcome** |
| A.136 | Characterization of the role of the Rab GTPase-activating protein AS160 in insulin-regulated GLUT4 trafficking | 10.1074/jbc.M503897200 | Did not provide a qualifying functional link between a molecular regulator and a defined GLUT4 exocytosis/trafficking step in the prespecified model scope. | **Wrong outcome** |
| A.137 | Characterization of two distinct binding modes between syntaxin 4 and Munc18c | 10.1042/BJ20082293 | Did not directly assess a GLUT4 trafficking/exocytosis endpoint (vesicle mobilization, surface insertion, tethering, docking, fusion, or subcellular redistribution); it evaluated glucose uptake, signalling, expression, or a systemic phenotype only. | **Wrong outcome** |
| A.138 | Characterization of unique regulatory mechanism of Tbc1d1 in GLUT4 trafficking by single molecule analysis of GLUT4 behavior | 10.2337/db12-1-377 | Conference abstract or meeting record without sufficiently detailed, full primary experimental methods and results for eligibility assessment. | **Ineligible publication type / insufficient primary data** |
| A.140 | Chemical hypoxia-induced glucose transporter-4 translocation in neonatal rat cardiomyocytes | 10.1016/j.arcmed.2007.06.022 | Population: Primary cultured neonatal rat cardiomyocytes | **Wrong population** |
| A.141 | Chloroquine Increases Glucose Uptake via Enhancing GLUT4 Translocation and Fusion with the Plasma Membrane in L6 Cells | 10.1159/000445562 | Reported a GLUT4 trafficking observation but did not test an eligible molecular regulator or regulatory mechanism with a defined trafficking endpoint. | **Wrong outcome** |
| A.142 | Chronic activation of 5'-AMP-activated protein kinase increases GLUT-4, hexokinase, and glycogen in muscle | 10.1152/jappl.1999.87.5.1990 | Did not directly assess a GLUT4 trafficking/exocytosis endpoint (vesicle mobilization, surface insertion, tethering, docking, fusion, or subcellular redistribution); it evaluated glucose uptake, signalling, expression, or a systemic phenotype only. | **Wrong outcome** |
| A.143 | Chronic activation of AMPK attenuates contraction-stimulated GLUT4 translocation in mouse skeletal muscle | 10.2337/db11-379-477 | Conference abstract or meeting record without sufficiently detailed, full primary experimental methods and results for eligibility assessment. | **Ineligible publication type / insufficient primary data** |
| A.144 | Chronic treatment with insulin selectively down-regulates cell-surface GLUT4 glucose transporters in 3T3-L1 adipocytes |  | Did not provide a qualifying functional link between a molecular regulator and a defined GLUT4 exocytosis/trafficking step in the prespecified model scope. | **Wrong outcome** |
| A.145 | Clathrin heavy chain 22 controls biogenesis of the human glucose transporter 4 storage compartment from the early secretory pathway | 10.1111/dme.13883 | Did not provide a qualifying functional link between a molecular regulator and a defined GLUT4 exocytosis/trafficking step in the prespecified model scope. | **Wrong outcome** |
| A.147 | Cloning of a Rab3 isotype predominately expressed in adipocytes | 10.1073/pnas.89.11.5049 | Did not provide a qualifying functional link between a molecular regulator and a defined GLUT4 exocytosis/trafficking step in the prespecified model scope. | **Wrong outcome** |
| A.149 | Comparative effects of GTPγS and insulin on the activation of Rho, phosphatidylinositol 3-kinase, and protein kinase N in Rat adipocytes. Relationship to glucose transport | 10.1074/jbc.273.13.7470 | Did not provide a qualifying functional link between a molecular regulator and a defined GLUT4 exocytosis/trafficking step in the prespecified model scope. | **Wrong outcome** |
| A.152 | Compartment ablation analysis of the insulin-responsive glucose transporter (GLUT4) in 3T3-L1 adipocytes | 10.1042/bj3150487 | Did not provide a qualifying functional link between a molecular regulator and a defined GLUT4 exocytosis/trafficking step in the prespecified model scope. | **Wrong outcome** |
| A.156 | Confocal imaging of the subcellular distribution of phosphatidylinositol 3,4,5-trisphosphate in insulin- and PDGF-stimulated 3T3-L1 adipocytes | 10.1042/0264-6021:3440511 | Did not provide a qualifying functional link between a molecular regulator and a defined GLUT4 exocytosis/trafficking step in the prespecified model scope. | **Wrong outcome** |
| A.157 | Consequences of tyrosine phosphorylation of Sx4 and Munc18c on GLUT4 trafficking | 10.1111/dme.5_13048 | Conference abstract or meeting record without sufficiently detailed, full primary experimental methods and results for eligibility assessment. | **Ineligible publication type / insufficient primary data** |
| A.158 | Constitutive activation of protein kinase Bα by membrane targeting promotes glucose and system A amino acid transport, protein synthesis, and inactivation of glycogen synthase kinase 3 in L6 muscle cells | 10.2337/diabetes.47.7.1006 | Did not provide a qualifying functional link between a molecular regulator and a defined GLUT4 exocytosis/trafficking step in the prespecified model scope. | **Wrong outcome** |
| A.159 | Construction and characterization of a conditionally active version of the serine/threonine kinase Akt | 10.1074/jbc.273.19.11937 | Did not provide a qualifying functional link between a molecular regulator and a defined GLUT4 exocytosis/trafficking step in the prespecified model scope. | **Wrong outcome** |
| A.160 | Contraction stimulates translocation of glucose transporter GLUT4 in skeletal muscle through a mechanism distinct from that of insulin | 10.1073/pnas.92.13.5817 | Did not provide a qualifying functional link between a molecular regulator and a defined GLUT4 exocytosis/trafficking step in the prespecified model scope. | **Wrong outcome** |
| A.161 | Contraction-Mediated Glucose Transport in Skeletal Muscle Is Regulated by a Framework of AMPK, TBC1D1/4, and Rac1 | 10.2337/db21-0587 | Conference abstract or meeting record without sufficiently detailed, full primary experimental methods and results for eligibility assessment. | **Ineligible publication type / insufficient primary data** |
| A.162 | Contraction-related stimuli regulate GLUT4 traffic in C2C 12-GLUT4myc skeletal muscle cells | 10.1152/ajpendo.00773.2009 | Did not provide a qualifying functional link between a molecular regulator and a defined GLUT4 exocytosis/trafficking step in the prespecified model scope. | **Wrong outcome** |
| A.163 | Conventional kinesin KIF5B mediates insulin-stimulated GLUT4 movements on microtubules | 10.1093/emboj/cdg237 | Did not provide a qualifying functional link between a molecular regulator and a defined GLUT4 exocytosis/trafficking step in the prespecified model scope. | **Wrong outcome** |
| A.165 | Cortactin, an actin binding protein, regulates GLUT4 translocation via actin filament remodeling | 10.1134/S0006297911110083 | Did not provide a qualifying functional link between a molecular regulator and a defined GLUT4 exocytosis/trafficking step in the prespecified model scope. | **Wrong outcome** |
| A.167 | Crystal structure of human Myosin 1c - The motor in GLUT4 exocytosis: Implications for Ca2 + regulation and 14-3-3 binding | 10.1016/j.jmb.2014.03.004 | Computational, structural, or mathematical analysis without experimental validation in an eligible GLUT4-trafficking model. | **Wrong study design** |
| A.168 | Cysteine string protein 1 (CSP1) modulates insulin sensitivity by attenuating glucose transporter 4 (GLUT4) vesicle docking with the plasma membrane | 10.2152/jmi.60.197 | Did not provide a qualifying functional link between a molecular regulator and a defined GLUT4 exocytosis/trafficking step in the prespecified model scope. | **Wrong outcome** |
| A.169 | Cysteine string protein 1 involved in insulin resistance by attenuating Glut4 vesicle docking with plasma membrane |  | Did not provide a qualifying functional link between a molecular regulator and a defined GLUT4 exocytosis/trafficking step in the prespecified model scope. | **Wrong outcome** |
| A.170 | De novo lipogenesis in skeletal muscle promotes insulin resistance | 10.2337/db12-1-377 | Conference abstract or meeting record without sufficiently detailed, full primary experimental methods and results for eligibility assessment. | **Ineligible publication type / insufficient primary data** |
| A.171 | Deciphering the mechanisms regulating GLUT4 translocation in skeletal muscle by spatial proteomics | 10.1016/j.mcpro.2022.100351 | Abstract-level/provisional proteomics report; it did not provide a completed primary experimental dataset with validated trafficking-regulator results. | **Ineligible publication type / insufficient primary data** |
| A.172 | Defective insulin-induced GLUT4 translocation in skeletal muscle of high fat-fed rats is associated with alterations in both Akt/protein kinase B and atypical protein kinase C (zeta/lambda) activities | 10.2337/diabetes.50.8.1901 | Reported a GLUT4 trafficking observation but did not test an eligible molecular regulator or regulatory mechanism with a defined trafficking endpoint. | **Wrong outcome** |
| A.173 | Defective insulin-stimulated GLUT4 translocation in brown adipocytes induces systemic glucose homeostasis dysregulation independent of thermogenesis in female mice | 10.1016/j.molmet.2021.101305 | Did not provide a qualifying functional link between a molecular regulator and a defined GLUT4 exocytosis/trafficking step in the prespecified model scope. | **Wrong outcome** |
| A.174 | Degradation of IRS1 leads to impaired glucose uptake in adipose tissue of the type 2 diabetes mouse model TALLYHO/Jng | 10.1677/JOE-09-0026 | Did not directly assess a GLUT4 trafficking/exocytosis endpoint (vesicle mobilization, surface insertion, tethering, docking, fusion, or subcellular redistribution); it evaluated glucose uptake, signalling, expression, or a systemic phenotype only. | **Wrong outcome** |
| A.175 | Deletion of both Rab-GTPase activating proteins TBC1D1 and TBC1D4 in mice eliminates insulin- and AICAR-stimulated glucose transport | 10.1007/s00125-013-3012-z | Did not provide a qualifying functional link between a molecular regulator and a defined GLUT4 exocytosis/trafficking step in the prespecified model scope. | **Wrong outcome** |
| A.177 | Demonstration of differential quantitative requirements for NSF among multiple vesicle fusion pathways of GLUT4 using a dominant-negative ATPase-deficient NSF | 10.1016/j.bbrc.2005.05.075 | Did not provide a qualifying functional link between a molecular regulator and a defined GLUT4 exocytosis/trafficking step in the prespecified model scope. | **Wrong outcome** |
| A.178 | Dependence of insulin-stimulated glucose transporter 4 translocation on 3-phosphoinositide-dependent protein kinase-1 and its target threonine-410 in the activation loop of protein kinase C-ζ | 10.1210/mend.13.10.0364 | Did not provide a qualifying functional link between a molecular regulator and a defined GLUT4 exocytosis/trafficking step in the prespecified model scope. | **Wrong outcome** |
| A.179 | Detection of insulin-regulated GLUT4-translocation by the insertion of a protein C epitope in L6 myoblasts | 10.1016/s0165-022x(02)00007-6 | Methods/assay report that did not test a molecular regulator or regulatory mechanism of GLUT4 trafficking in an eligible primary experimental study. | **Wrong study design** |
| A.180 | Development of a new model system to dissect isoform specific Akt signalling in adipocytes | 10.1042/BJ20150191 | Did not directly assess a GLUT4 trafficking/exocytosis endpoint (vesicle mobilization, surface insertion, tethering, docking, fusion, or subcellular redistribution); it evaluated glucose uptake, signalling, expression, or a systemic phenotype only. | **Wrong outcome** |
| A.181 | Development of an in vitro reconstitution assay for glucose transporter 4 translocation | 10.1073/pnas.96.26.14919 | Did not directly assess a GLUT4 trafficking/exocytosis endpoint (vesicle mobilization, surface insertion, tethering, docking, fusion, or subcellular redistribution); it evaluated glucose uptake, signalling, expression, or a systemic phenotype only. | **Wrong outcome** |
| A.182 | Developmentally spliced PKCβII provides a possible link between mTORC2 and Akt kinase to regulate 3T3-L1 adipocyte insulin-stimulated glucose transport | 10.1016/j.bbrc.2009.08.063 | Did not provide a qualifying functional link between a molecular regulator and a defined GLUT4 exocytosis/trafficking step in the prespecified model scope. | **Wrong outcome** |
| A.183 | DHHC7 Palmitoylates Glucose Transporter 4 (Glut4) and Regulates Glut4 Membrane Translocation | 10.1074/jbc.M116.747139 | Did not provide a qualifying functional link between a molecular regulator and a defined GLUT4 exocytosis/trafficking step in the prespecified model scope. | **Wrong outcome** |
| A.184 | Diacylglycerol kinase z, a novel binding partner of insulin receptor substrate, negatively modulated GLUT4 translocation in 3T3-L1 adipocytes |  | Did not provide a qualifying functional link between a molecular regulator and a defined GLUT4 exocytosis/trafficking step in the prespecified model scope. | **Wrong outcome** |
| A.185 | Diacylglycerol promotes GLUT4 translocation to the cell surface in a PKCε-dependent and PKCλ/ι and -ζ-independent manner | 10.1016/j.lfs.2013.06.014 | Did not provide a qualifying functional link between a molecular regulator and a defined GLUT4 exocytosis/trafficking step in the prespecified model scope. | **Wrong outcome** |
| A.188 | Differential regulation of cardiac glucose and fatty acid uptake by endosomal pH and actin filaments | 10.1152/ajpcell.00334.2009 | Investigated a cardiac/cardiomyocyte or heart model; the final review scope was restricted to adipocyte and skeletal-muscle models. | **Wrong population/model** |
| A.189 | Differential Strain-dependent Ovarian and Metabolic Responses in a Mouse Model of PCOS | 10.1210/endocr/bqad024 | Review, commentary, or other non-primary report rather than an eligible primary experimental study. | **Ineligible publication type / insufficient primary data** |
| A.198 | Disruption of microtubules in rat skeletal muscle does not inhibit insulin- or contraction-stimulated glucose transport | 10.1152/ajpendo.00238.2002 | Did not directly assess a GLUT4 trafficking/exocytosis endpoint (vesicle mobilization, surface insertion, tethering, docking, fusion, or subcellular redistribution); it evaluated glucose uptake, signalling, expression, or a systemic phenotype only. | **Wrong outcome** |
| A.199 | Disruption of the exocyst trafficking complex results in impaired glucose uptake in skeletal muscle | 10.2337/db19-1940-P | Conference abstract or meeting record without sufficiently detailed, full primary experimental methods and results for eligibility assessment. | **Ineligible publication type / insufficient primary data** |
| A.200 | Dissecting multiple steps of GLUT4 trafficking and identifying the sites of insulin action | 10.1016/j.cmet.2006.11.013 | Reported a GLUT4 trafficking observation but did not test an eligible molecular regulator or regulatory mechanism with a defined trafficking endpoint. | **Wrong outcome** |
| A.201 | Dissection of GLUT4 recycling pathway into exocytosis and endocytosis in rat adipocytes. Evidence that GTP-binding proteins are involved in both processes | 10.1074/jbc.270.19.11489 | Did not provide a qualifying functional link between a molecular regulator and a defined GLUT4 exocytosis/trafficking step in the prespecified model scope. | **Wrong outcome** |
| A.202 | Dissociation of 5′ AMP-activated protein kinase activation and glucose uptake stimulation by mitochondrial uncoupling and hyperosmolar stress: Differential sensitivities to intracellular Ca2+ and protein kinase C inhibition | 10.1006/bbrc.2001.5275 | Did not provide a qualifying functional link between a molecular regulator and a defined GLUT4 exocytosis/trafficking step in the prespecified model scope. | **Wrong outcome** |
| A.204 | Distinct roles of two Rab GAPs, AS160/Tbc1d4 and Tbc1d1, in intracellular GLUT4 trafficking analyzed by single molecule imaging of GLUT4 behavior | 10.1091/mbc.E11-10-0886 | Did not provide a qualifying functional link between a molecular regulator and a defined GLUT4 exocytosis/trafficking step in the prespecified model scope. | **Wrong outcome** |
| A.205 | Distinct signals in the GLUT4 glucose transporter for internalization and for targeting to an insulin-responsive compartment | 10.1083/jcb.130.5.1071 | Reported a GLUT4 trafficking observation but did not test an eligible molecular regulator or regulatory mechanism with a defined trafficking endpoint. | **Wrong outcome** |
| A.207 | DOC2B enhanced muscle insulin sensitivity by interacting with cytoskeletal proteins | 10.1096/fasebj.2018.32.1_supplement.719.10 | Did not provide a qualifying functional link between a molecular regulator and a defined GLUT4 exocytosis/trafficking step in the prespecified model scope. | **Wrong outcome** |
| A.208 | Doc2b is a key effector of insulin secretion and skeletal muscle insulin sensitivity | 10.2337/db11-1525 | Conference abstract or meeting record without sufficiently detailed, full primary experimental methods and results for eligibility assessment. | **Ineligible publication type / insufficient primary data** |
| A.209 | Doc2b promotes GLUT4 exocytosis by activating the SNARE-mediated fusion reaction in a calcium- and membrane bending-dependent manner | 10.1091/mbc.E12-11-0810 | Isolated SNARE-fusion/reconstitution evidence was not validated as GLUT4 trafficking in a prespecified intact adipocyte or skeletal-muscle model. | **Wrong population/model** |
| A.210 | DOC2B: A novel syntaxin-4 binding protein mediating insulin-regulated GLUT4 vesicle fusion in adipocytes | 10.2337/db08-0303 | Conference abstract or meeting record without sufficiently detailed, full primary experimental methods and results for eligibility assessment. | **Ineligible publication type / insufficient primary data** |
| A.211 | Doc2β is a novel Munc18c-interacting partner and positive effector of syntaxin 4-mediated exocytosis | 10.1074/jbc.M701661200 | Did not provide a qualifying functional link between a molecular regulator and a defined GLUT4 exocytosis/trafficking step in the prespecified model scope. | **Wrong outcome** |
| A.212 | DOC2β: Part of the insulin-stimulated glut4 vesicle trafficking machinery in adipocytes |  | Did not provide a qualifying functional link between a molecular regulator and a defined GLUT4 exocytosis/trafficking step in the prespecified model scope. | **Wrong outcome** |
| A.213 | Dual Function of PI(4,5)P2 in Insulin-Regulated Exocytic Trafficking of GLUT4 in Adipocytes | 10.1016/j.jmb.2020.06.019 | Did not provide a qualifying functional link between a molecular regulator and a defined GLUT4 exocytosis/trafficking step in the prespecified model scope. | **Wrong outcome** |
| A.214 | Dual regulation of Rho and Rac by p120 catenin controls adipocyte plasma membrane trafficking | 10.1074/jbc.M603127200 | Did not provide a qualifying functional link between a molecular regulator and a defined GLUT4 exocytosis/trafficking step in the prespecified model scope. | **Wrong outcome** |
| A.216 | Dual-mode of insulin action controls GLUT4 vesicle exocytosis | 10.1083/jcb.201008135 | Reported a GLUT4 trafficking observation but did not test an eligible molecular regulator or regulatory mechanism with a defined trafficking endpoint. | **Wrong outcome** |
| A.217 | Dynamic Metabolomics Reveals that Insulin Primes the Adipocyte for Glucose Metabolism | 10.1016/j.celrep.2017.11.085 | Did not directly assess a GLUT4 trafficking/exocytosis endpoint (vesicle mobilization, surface insertion, tethering, docking, fusion, or subcellular redistribution); it evaluated glucose uptake, signalling, expression, or a systemic phenotype only. | **Wrong outcome** |
| A.218 | Dynamics of signaling during insulin-stimulated endocytosis of its receptor in adipocytes | 10.1074/jbc.270.1.59 | Did not provide a qualifying functional link between a molecular regulator and a defined GLUT4 exocytosis/trafficking step in the prespecified model scope. | **Wrong outcome** |
| A.219 | E4orf1 protein requires the distal but not proximal insulin signaling to enhance Glut4 translocation | Chỉ có abst | Abstract-only record without a citable full primary experimental report containing sufficient methods and results. | **Ineligible publication type / insufficient primary data** |
| A.221 | Effect of insulin on GLUT4 cell surface content and turnover rate in human skeletal muscle as measured by the exofacial bis-mannose photolabeling technique | 10.2337/diab.46.12.1965 | Human-only, non-target tissue, or non-eligible biological model; no qualifying mechanistic validation in adipocytes or skeletal muscle was reported. | **Wrong population/model** |
| A.224 | Effects of general receptor for phosphoinositides 1 on insulin and insulin-like growth factor I-induced cytoskeletal rearrangement, glucose transporter-4 translocation, and deoxyribonucleic acid synthesis | 10.1210/en.139.12.4984 | Did not provide a qualifying functional link between a molecular regulator and a defined GLUT4 exocytosis/trafficking step in the prespecified model scope. | **Wrong outcome** |
| A.228 | Effects of transiently expressed atypical (ζ, λ), conventional (α, β) and novel (δ, ɛ) protein kinase C isoforms on insulin-stimulated translocation of epitope-tagged GLUT4 glucose transporters in rat adipocytes: specific interchangeable effects of protein kinases C-ζ and C-λ |  | Reported a GLUT4 trafficking observation but did not test an eligible molecular regulator or regulatory mechanism with a defined trafficking endpoint. | **Wrong outcome** |
| A.231 | Electrical pulse stimulation induces GLUT4 translocation in a Rac-Akt-dependent manner in C2C12 myotubes | 10.1002/1873-3468.12982 | Did not provide a qualifying functional link between a molecular regulator and a defined GLUT4 exocytosis/trafficking step in the prespecified model scope. | **Wrong outcome** |
| A.233 | Endocytosis of the glucose transporter GLUT8 is mediated by interaction of a dileucine motif with the β2-adaptin subunit of the AP-2 adaptor complex | 10.1242/jcs.02943 | Artificial or heterologous experimental system was used without mechanistic validation in an eligible GLUT4-expressing adipocyte or skeletal-muscle model. | **Wrong population/model** |
| A.235 | Endothelin-1 suppresses insulin-stimulated Akt phosphorylation and glucose uptake via GPCR kinase 2 in skeletal muscle cells | 10.1111/bph.13406 | Did not directly assess a GLUT4 trafficking/exocytosis endpoint (vesicle mobilization, surface insertion, tethering, docking, fusion, or subcellular redistribution); it evaluated glucose uptake, signalling, expression, or a systemic phenotype only. | **Wrong outcome** |
| A.236 | Enigma interacts with adaptor protein with PH and SH2 domains to control insulin-induced actin cytoskeleton remodeling and glucose transporter 4 translocation | 10.1210/me.2005-0455 | Did not directly assess a GLUT4 trafficking/exocytosis endpoint (vesicle mobilization, surface insertion, tethering, docking, fusion, or subcellular redistribution); it evaluated glucose uptake, signalling, expression, or a systemic phenotype only. | **Wrong outcome** |
| A.237 | Entry of newly synthesized GLUT4 into the insulin-responsive storage compartment is dependent upon both the amino terminus and the large cytoplasmic loop | 10.1074/jbc.M405694200 | Did not provide a qualifying functional link between a molecular regulator and a defined GLUT4 exocytosis/trafficking step in the prespecified model scope. | **Wrong outcome** |
| A.238 | Entry of newly synthesized GLUT4 into the insulin-responsive storage compartment is GGA dependent | 10.1038/sj.emboj.7600159 | Did not provide a qualifying functional link between a molecular regulator and a defined GLUT4 exocytosis/trafficking step in the prespecified model scope. | **Wrong outcome** |
| A.239 | Erythrocyte dematin regulates glucose transport via Akt phosphorylation and 14-3-3zeta association |  | Did not provide a qualifying functional link between a molecular regulator and a defined GLUT4 exocytosis/trafficking step in the prespecified model scope. | **Wrong outcome** |
| A.245 | Evidence for involvement of protein kinase C (PKC)-ζ and noninvolvement of diacylglycerol-sensitive PKCs in insulin-stimulated glucose transport in L6 myotubes | 10.1210/en.138.11.4721 | Did not provide a qualifying functional link between a molecular regulator and a defined GLUT4 exocytosis/trafficking step in the prespecified model scope. | **Wrong outcome** |
| A.246 | Exercise causes muscle GLUT4 translocation in an insulin-independent manner | 10.4172/0974-8369.1000S3007 | Did not provide a qualifying functional link between a molecular regulator and a defined GLUT4 exocytosis/trafficking step in the prespecified model scope. | **Wrong outcome** |
| A.249 | Expression of a synapsinsIIb site 1 phosphorylation mutant in 3t3-L1 adipocytes inhibits basal intracellular retention of Glut4 | 10.1242/jcs.03413 | Did not provide a qualifying functional link between a molecular regulator and a defined GLUT4 exocytosis/trafficking step in the prespecified model scope. | **Wrong outcome** |
| A.255 | Functional cloning of TUG as a regulator of GLUT4 glucose transporter trafficking | 10.1038/nature01989 | Did not provide a qualifying functional link between a molecular regulator and a defined GLUT4 exocytosis/trafficking step in the prespecified model scope. | **Wrong outcome** |
| A.259 | Functional role of Rab11 in GLUT4 trafficking in cardiomyocytes | 10.1016/j.mce.2005.02.004 | Investigated a cardiac/cardiomyocyte or heart model; the final review scope was restricted to adipocyte and skeletal-muscle models. | **Wrong population/model** |
| A.261 | Fyn and CDK5 negatively regulate insulin-stimulated GLUT4 translocation through cortical F-actin rearrangement |  | Did not provide a qualifying functional link between a molecular regulator and a defined GLUT4 exocytosis/trafficking step in the prespecified model scope. | **Wrong outcome** |
| A.263 | G(i)-mediated translocation of GLUT4 is independent of p85/p110α and p110γ phosphoinositide 3-kinases but might involve the activation of Akt kinase | 10.1042/0264-6021:3450543 | Did not provide a qualifying functional link between a molecular regulator and a defined GLUT4 exocytosis/trafficking step in the prespecified model scope. | **Wrong outcome** |
| A.265 | Gain-of-Function Dynamin-2 Mutations Linked to Centronuclear Myopathy Impair Ca2+-Induced Exocytosis in Human Myoblasts | 10.3390/ijms231810363 | Did not provide a qualifying functional link between a molecular regulator and a defined GLUT4 exocytosis/trafficking step in the prespecified model scope. | **Wrong outcome** |
| A.266 | GAPDH binds GLUT4 reciprocally to hexokinase-II and regulates glucose transport activity | 10.1042/BJ20081319 | Did not directly assess a GLUT4 trafficking/exocytosis endpoint (vesicle mobilization, surface insertion, tethering, docking, fusion, or subcellular redistribution); it evaluated glucose uptake, signalling, expression, or a systemic phenotype only. | **Wrong outcome** |
| A.272 | Glucose transport in L6 myoblasts overexpressing GLUT1 and GLUT4 |  | Did not provide a qualifying functional link between a molecular regulator and a defined GLUT4 exocytosis/trafficking step in the prespecified model scope. | **Wrong outcome** |
| A.274 | Glucose uptake through translocation and activation of GLUT4 in PI3K/Akt signaling pathway by asiatic acid in diabetic rats | 10.1177/0960327114561663 | Did not provide a qualifying functional link between a molecular regulator and a defined GLUT4 exocytosis/trafficking step in the prespecified model scope. | **Wrong outcome** |
| A.275 | Glut-4 is translocated to both caveolae and non-caveolar lipid rafts, but is partially internalized through caveolae in insulin-stimulated adipocytes | 10.1038/cr.2007.73 | Did not provide a qualifying functional link between a molecular regulator and a defined GLUT4 exocytosis/trafficking step in the prespecified model scope. | **Wrong outcome** |
| A.277 | GLUT4 endosomal trafficking, but not signaling is altered during insulin resistance in 3T3-L1 cells | 10.2337/db12-1-377 | Conference abstract or meeting record without sufficiently detailed, full primary experimental methods and results for eligibility assessment. | **Ineligible publication type / insufficient primary data** |
| A.281 | Glut4 subcellular trafficking: molecular components and vesicular pathways | 10.1042/bst025460sb | Reported a GLUT4 trafficking observation but did not test an eligible molecular regulator or regulatory mechanism with a defined trafficking endpoint. | **Wrong outcome** |
| A.282 | GLUT4 trafficking in insulin-stimulated rat adipose cells: Evidence that heterotrimeric GTP-binding proteins regulate the fusion of docked GLUT4-containing vesicles | 10.1042/0264-6021:3430571 | Did not provide a qualifying functional link between a molecular regulator and a defined GLUT4 exocytosis/trafficking step in the prespecified model scope. | **Wrong outcome** |
| A.284 | GLUT4 translocation with insulin: revisiting the case for dose-dependent quantal release | 10.1101/2025.04.11.648479 | Did not provide a qualifying functional link between a molecular regulator and a defined GLUT4 exocytosis/trafficking step in the prespecified model scope. | **Wrong outcome** |
| A.293 | High glucose and insulin in combination cause insulin receptor substrate-1 and -2 depletion and protein kinase B desensitisation in primary cultured rat adipocytes: Possible implications for insulin resistance in type 2 diabetes | 10.1530/eje.0.1480157 | Did not directly assess a GLUT4 trafficking/exocytosis endpoint (vesicle mobilization, surface insertion, tethering, docking, fusion, or subcellular redistribution); it evaluated glucose uptake, signalling, expression, or a systemic phenotype only. | **Wrong outcome** |
| A.294 | Hydrogen Peroxide Generated during Cellular Insulin Stimulation Is Integral to Activation of the Distal Insulin Signaling Cascade in 3T3-L1 Adipocytes | 10.1074/jbc.M105061200 | Did not provide a qualifying functional link between a molecular regulator and a defined GLUT4 exocytosis/trafficking step in the prespecified model scope. | **Wrong outcome** |
| A.296 | Identification and characterization of new players in insulin-controlled GLUT4 trafficking | 10.2337/db20-296-OR | Conference abstract or meeting record without sufficiently detailed, full primary experimental methods and results for eligibility assessment. | **Ineligible publication type / insufficient primary data** |
| A.300 | Identification of a novel AS160 splice variant that regulates GLUT4 translocation and glucose-uptake in rat muscle cells | 10.1016/j.cellsig.2008.08.010 | Did not provide a qualifying functional link between a molecular regulator and a defined GLUT4 exocytosis/trafficking step in the prespecified model scope. | **Wrong outcome** |
| A.301 | Identification of a novel modulator of GLUT4 from virtual screening study using GLUT4 homology model | 10.1091/mbc.E15-09-0674 | Computational, structural, or mathematical analysis without experimental validation in an eligible GLUT4-trafficking model. | **Wrong study design** |
| A.302 | Identification of a novel phosphorylation site on TBC1D4 regulated by AMP-activated protein kinase in skeletal muscle | 10.1152/ajpcell.00297.2009 | Did not provide a qualifying functional link between a molecular regulator and a defined GLUT4 exocytosis/trafficking step in the prespecified model scope. | **Wrong outcome** |
| A.305 | Identification of discrete classes of endosome-derived small vesicles as a major cellular pool for recycling membrane proteins | 10.1091/mbc.12.4.981 | Did not provide a qualifying functional link between a molecular regulator and a defined GLUT4 exocytosis/trafficking step in the prespecified model scope. | **Wrong outcome** |
| A.307 | Identification of new molecular components in the GLUT4 clusters in the plasma membrane of adipose cells by mass spectrometry-based proteomics |  | Did not provide a qualifying functional link between a molecular regulator and a defined GLUT4 exocytosis/trafficking step in the prespecified model scope. | **Wrong outcome** |
| A.309 | Identification of Rab proteins as regulated intermediates in insulin and energy status stimulated GLUT4 trafficking | 10.1111/j.1464-5491.2011.03232_18.x | Did not provide a qualifying functional link between a molecular regulator and a defined GLUT4 exocytosis/trafficking step in the prespecified model scope. | **Wrong outcome** |
| A.310 | Identification of regulatory nodes in Glut4 trafficking | 10.2337/db13-1743-1824 | Conference abstract or meeting record without sufficiently detailed, full primary experimental methods and results for eligibility assessment. | **Ineligible publication type / insufficient primary data** |
| A.311 | Identification of three distinct functional sites of insulin-mediated GLUT4 trafficking in adipocytes using quantitative single molecule imaging | 10.1091/mbc.e10-01-0029 | Reported a GLUT4 trafficking observation but did not test an eligible molecular regulator or regulatory mechanism with a defined trafficking endpoint. | **Wrong outcome** |
| A.312 | Identification of tomosyn as a new downstream target of Akt |  | Did not provide a qualifying functional link between a molecular regulator and a defined GLUT4 exocytosis/trafficking step in the prespecified model scope. | **Wrong outcome** |
| A.313 | Identifying new regulators of insulin-stimulated GLUT4 exocytosis |  | Did not provide a qualifying functional link between a molecular regulator and a defined GLUT4 exocytosis/trafficking step in the prespecified model scope. | **Wrong outcome** |
| A.314 | Identifying new sites of insulin action in GLUT4 vesicle exocytosis | 10.1091/mbc.E11-10-0886 | Did not provide a qualifying functional link between a molecular regulator and a defined GLUT4 exocytosis/trafficking step in the prespecified model scope. | **Wrong outcome** |
| A.316 | Immunoelectron microscopic evidence that GLUT4 translocation explains the stimulation of glucose transport in isolated rat white adipose cells | 10.1242/jcs.113.23.4203 | Reported a GLUT4 trafficking observation but did not test an eligible molecular regulator or regulatory mechanism with a defined trafficking endpoint. | **Wrong outcome** |
| A.317 | Impaired glucose tolerance, insulin sensitivity and biphasic insulin release in Doc2b knockout mice | 10.2337/db11-1929-2041 | Conference abstract or meeting record without sufficiently detailed, full primary experimental methods and results for eligibility assessment. | **Ineligible publication type / insufficient primary data** |
| A.318 | Impaired insulin-regulated membrane aminopeptidase translocation to the plasma membrane in adipocytes of Otsuka Long Evans Tokushima Fatty rats |  | Did not directly assess a GLUT4 trafficking/exocytosis endpoint (vesicle mobilization, surface insertion, tethering, docking, fusion, or subcellular redistribution); it evaluated glucose uptake, signalling, expression, or a systemic phenotype only. | **Wrong outcome** |
| A.319 | Impaired tethering and fusion of GLUT4 vesicles in insulin-resistant human adipose cells | 10.2337/db12-1741 | Conference abstract or meeting record without sufficiently detailed, full primary experimental methods and results for eligibility assessment. | **Ineligible publication type / insufficient primary data** |
| A.320 | Impaired translocation of GLUT4 results in insulin resistance of atrophic soleus muscle | 10.1155/2015/291987 | Reported a GLUT4 trafficking observation but did not test an eligible molecular regulator or regulatory mechanism with a defined trafficking endpoint. | **Wrong outcome** |
| A.323 | In situ detection of the activation of Rac1 and RalA small GTPases in mouse adipocytes by immunofluorescent microscopy following in vivo and ex vivo insulin stimulation | 10.1016/j.cellsig.2017.08.004 | Did not provide a qualifying functional link between a molecular regulator and a defined GLUT4 exocytosis/trafficking step in the prespecified model scope. | **Wrong outcome** |
| A.325 | INCREMENT of COMPLEXIN-2 in INSULIN RESISTANCE-LIKE STATES. POSSIBLE INVOLVEMENT in DEFICIENT GLUT4-TRANSLOCATION |  | Did not provide a qualifying functional link between a molecular regulator and a defined GLUT4 exocytosis/trafficking step in the prespecified model scope. | **Wrong outcome** |
| A.330 | Inhibition of insulin/PI3K/AKT signaling decreases adipose Sortilin 1 in mice and 3 T3-L1 adipocytes | 10.1016/j.bbadis.2017.08.012 | Did not provide a qualifying functional link between a molecular regulator and a defined GLUT4 exocytosis/trafficking step in the prespecified model scope. | **Wrong outcome** |
| A.332 | Initial entry of IRAP into the insulin-responsive storage compartment occurs prior to basal or insulin-stimulated plasma membrane recycling | 10.1152/ajpendo.00175.2005 | Did not directly assess a GLUT4 trafficking/exocytosis endpoint (vesicle mobilization, surface insertion, tethering, docking, fusion, or subcellular redistribution); it evaluated glucose uptake, signalling, expression, or a systemic phenotype only. | **Wrong outcome** |
| A.333 | Insulin accelerates inter-endosomal GLUT4 traffic via phosphatidylinositol 3-kinase and protein kinase B | 10.1074/jbc.M102964200 | Did not directly assess a GLUT4 trafficking/exocytosis endpoint (vesicle mobilization, surface insertion, tethering, docking, fusion, or subcellular redistribution); it evaluated glucose uptake, signalling, expression, or a systemic phenotype only. | **Wrong outcome** |
| A.337 | Insulin and muscle contraction regulate TBC1D1 through phosphorylation and interaction with the cytosolic tail of insulin-regulated aminopeptidase | 10.1007/s00125-018-4693-0 | Did not provide a qualifying functional link between a molecular regulator and a defined GLUT4 exocytosis/trafficking step in the prespecified model scope. | **Wrong outcome** |
| A.338 | Insulin and muscle contraction-induced GLUT4 traffic: Integration within specific phosphorylation-patterns of the downstream target TBC1D4 | 10.1007/s00125-020-05221-5 | Did not provide a qualifying functional link between a molecular regulator and a defined GLUT4 exocytosis/trafficking step in the prespecified model scope. | **Wrong outcome** |
| A.343 | Insulin induces a change in Rab5 subcellular localization in adipocytes independently of phosphatidylinositol 3-kinase activation | 10.1210/en.137.8.3408 | Did not provide a qualifying functional link between a molecular regulator and a defined GLUT4 exocytosis/trafficking step in the prespecified model scope. | **Wrong outcome** |
| A.345 | Insulin induces the exocytic traffic of LRP1 from GSV-like structural vesicles |  | Did not provide a qualifying functional link between a molecular regulator and a defined GLUT4 exocytosis/trafficking step in the prespecified model scope. | **Wrong outcome** |
| A.346 | Insulin induces translocation of glucose transporter GLUT4 to plasma membrane caveolae in adipocytes | 10.1096/fj.01-0646fje | Reported a GLUT4 trafficking observation but did not test an eligible molecular regulator or regulatory mechanism with a defined trafficking endpoint. | **Wrong outcome** |
| A.353 | Insulin Regulates Glut4 Confinement in Plasma Membrane Clusters in Adipose Cells | 10.1371/journal.pone.0057559 | Did not provide a qualifying functional link between a molecular regulator and a defined GLUT4 exocytosis/trafficking step in the prespecified model scope. | **Wrong outcome** |
| A.356 | Insulin resistance and muscle insulin receptor substrate-1 serine hyperphosphorylation | 10.14814/phy2.12236 | Did not directly assess a GLUT4 trafficking/exocytosis endpoint (vesicle mobilization, surface insertion, tethering, docking, fusion, or subcellular redistribution); it evaluated glucose uptake, signalling, expression, or a systemic phenotype only. | **Wrong outcome** |
| A.357 | Insulin responsiveness of endosomal GLUT4 vesicles revealed by a novel GLUT4 probe | 10.1091/mbc.E13-10-0584 | Did not provide a qualifying functional link between a molecular regulator and a defined GLUT4 exocytosis/trafficking step in the prespecified model scope. | **Wrong outcome** |
| A.359 | Insulin signaling regulating the trafficking and plasma membrane fusion of GLUT4-containing intracellular vesicles | 10.1006/excr.1999.4675 | Did not provide a qualifying functional link between a molecular regulator and a defined GLUT4 exocytosis/trafficking step in the prespecified model scope. | **Wrong outcome** |
| A.360 | Insulin signaling regulation of sortilin 1 and Glut4 storage vesicle formation in NIH 3T3-L1 adipocytes and mouse adipose tissue |  | Did not provide a qualifying functional link between a molecular regulator and a defined GLUT4 exocytosis/trafficking step in the prespecified model scope. | **Wrong outcome** |
| A.362 | Insulin signaling via the PI3-kinase/Akt pathway regulates airway glucose uptake and barrier function in a CFTR-dependent manner | 10.1152/ajplung.00364.2016 | Did not directly assess a GLUT4 trafficking/exocytosis endpoint (vesicle mobilization, surface insertion, tethering, docking, fusion, or subcellular redistribution); it evaluated glucose uptake, signalling, expression, or a systemic phenotype only. | **Wrong outcome** |
| A.363 | Insulin signaling: GLUT4 vesicles exit via the exocyst | 10.1016/S0960-9822(03)00478-0 | Did not provide a qualifying functional link between a molecular regulator and a defined GLUT4 exocytosis/trafficking step in the prespecified model scope. | **Wrong outcome** |
| A.364 | Insulin signals governing translocation of glucose transporter glut4 in muscle cells |  | Did not provide a qualifying functional link between a molecular regulator and a defined GLUT4 exocytosis/trafficking step in the prespecified model scope. | **Wrong outcome** |
| A.367 | Insulin stimulates membrane fusion and GLUT4 accumulation in clathrin coats on adipocyte plasma membranes | 10.1128/MCB.01719-06 | Reported a GLUT4 trafficking observation but did not test an eligible molecular regulator or regulatory mechanism with a defined trafficking endpoint. | **Wrong outcome** |
| A.370 | Insulin stimulates the phosphorylation of the exocyst protein Sec8 in adipocytes | 10.1042/BSR20080162 | Did not directly assess a GLUT4 trafficking/exocytosis endpoint (vesicle mobilization, surface insertion, tethering, docking, fusion, or subcellular redistribution); it evaluated glucose uptake, signalling, expression, or a systemic phenotype only. | **Wrong outcome** |
| A.374 | Insulin-Activated Signaling Pathway and GLUT4 Membrane Translocation in hiPSC-Derived Cardiomyocytes | 10.3390/ijms25158197 | Investigated a cardiac/cardiomyocyte or heart model; the final review scope was restricted to adipocyte and skeletal-muscle models. | **Wrong population/model** |
| A.376 | Insulin-dependent protein trafficking in skeletal muscle cells | 10.1152/ajpendo.1998.275.2.e187 | Did not provide a qualifying functional link between a molecular regulator and a defined GLUT4 exocytosis/trafficking step in the prespecified model scope. | **Wrong outcome** |
| A.377 | Insulin-independent glucose uptake in skeletal muscle following B2-adrenoceptor activation is mediated by mTORC2 and GLUT4 |  | Did not provide a qualifying functional link between a molecular regulator and a defined GLUT4 exocytosis/trafficking step in the prespecified model scope. | **Wrong outcome** |
| A.378 | INSULIN-INDEPENDENT GLUT4 TRAFFICKING IN SKELETAL MUSCLE: NEW MECHANISMS AND ADVANCES TO FACE METABOLIC DISEASES |  | Did not provide a qualifying functional link between a molecular regulator and a defined GLUT4 exocytosis/trafficking step in the prespecified model scope. | **Wrong outcome** |
| A.379 | Insulin-induced actin filament remodeling colocalizes actin with phosphatidylinositol 3-kinase and GLUT4 in L6 myotubes | 10.1242/jcs.113.2.279 | Reported a GLUT4 trafficking observation but did not test an eligible molecular regulator or regulatory mechanism with a defined trafficking endpoint. | **Wrong outcome** |
| A.383 | Insulin-induced βcyto-actin dynamics in live skeletal muscle | 10.2337/db16-1695-1770 | Conference abstract or meeting record without sufficiently detailed, full primary experimental methods and results for eligibility assessment. | **Ineligible publication type / insufficient primary data** |
| A.391 | Insulin-regulated trafficking of dual-labeled glucose transporter 4 in primary rat adipose cells | 10.1006/bbrc.2001.5620 | Did not directly assess a GLUT4 trafficking/exocytosis endpoint (vesicle mobilization, surface insertion, tethering, docking, fusion, or subcellular redistribution); it evaluated glucose uptake, signalling, expression, or a systemic phenotype only. | **Wrong outcome** |
| A.392 | Insulin-regulated trafficking of GLUT4 requires ubiquitination | 10.1111/j.1600-0854.2010.01113.x | Did not provide a qualifying functional link between a molecular regulator and a defined GLUT4 exocytosis/trafficking step in the prespecified model scope. | **Wrong outcome** |
| A.393 | Insulin-responsive aminopeptidase trafficking in 3T3-L1 adipocytes | 10.1074/jbc.275.4.2560 | Did not provide a qualifying functional link between a molecular regulator and a defined GLUT4 exocytosis/trafficking step in the prespecified model scope. | **Wrong outcome** |
| A.395 | Insulin-responsive human adipocytes express two glucose transporter isoforms and target them to different vesicles | 10.1210/jc.77.1.286 | Did not provide a qualifying functional link between a molecular regulator and a defined GLUT4 exocytosis/trafficking step in the prespecified model scope. | **Wrong outcome** |
| A.397 | Insulin-stimulated endoproteolytic TUG cleavage links energy expenditure with glucose uptake | 10.1038/s42255-021-00359-x | Did not provide a qualifying functional link between a molecular regulator and a defined GLUT4 exocytosis/trafficking step in the prespecified model scope. | **Wrong outcome** |
| A.400 | Insulin-stimulated glucose uptake partly relies on p21-activated kinase (PAK)2, but not PAK1, in mouse skeletal muscle | 10.1113/JP280294 | Did not directly assess a GLUT4 trafficking/exocytosis endpoint (vesicle mobilization, surface insertion, tethering, docking, fusion, or subcellular redistribution); it evaluated glucose uptake, signalling, expression, or a systemic phenotype only. | **Wrong outcome** |
| A.406 | Insulin-stimulated PAK1 activation is required for glucose uptake in skeletal muscle cells |  | Did not provide a qualifying functional link between a molecular regulator and a defined GLUT4 exocytosis/trafficking step in the prespecified model scope. | **Wrong outcome** |
| A.409 | Insulin-stimulated plasma membrane association and activation of Akt2, aPKC zeta and aPKC lambda in high fat fed rodent skeletal muscle | 10.1113/jphysiol.2005.086694 | Did not directly assess a GLUT4 trafficking/exocytosis endpoint (vesicle mobilization, surface insertion, tethering, docking, fusion, or subcellular redistribution); it evaluated glucose uptake, signalling, expression, or a systemic phenotype only. | **Wrong outcome** |
| A.412 | Insulin-stimulated translocation of the fatty acid transporter CD36 to the plasma membrane is mediated by the small GTPase Rac1 in adipocytes | 10.1016/j.cellsig.2024.111102 | Did not provide a qualifying functional link between a molecular regulator and a defined GLUT4 exocytosis/trafficking step in the prespecified model scope. | **Wrong outcome** |
| A.414 | Insulin, but not contraction, activates Akt/PKB in isolated rat skeletal muscle | 10.1074/jbc.273.24.14679 | Did not provide a qualifying functional link between a molecular regulator and a defined GLUT4 exocytosis/trafficking step in the prespecified model scope. | **Wrong outcome** |
| A.415 | Integration of multiple downstream signals determines the net effect of insulin on MAP kinase vs. PI 3′-kinase activation: Potential role of insulin-stimulated H2O2 | 10.1016/j.cellsig.2003.08.002 | Did not provide a qualifying functional link between a molecular regulator and a defined GLUT4 exocytosis/trafficking step in the prespecified model scope. | **Wrong outcome** |
| A.417 | Intracellular retention and insulin-stimulated mobilization of GLUT4 glucose transporters. | 10.1016/S0083-6729(08)00607-9 | Artificial or heterologous experimental system was used without mechanistic validation in an eligible GLUT4-expressing adipocyte or skeletal-muscle model. | **Wrong population/model** |
| A.419 | Intracellular targeting of the insuin-regulatable glucose transporter (GLUT4) is isoform specific and independent of cell type |  | Did not provide a qualifying functional link between a molecular regulator and a defined GLUT4 exocytosis/trafficking step in the prespecified model scope. | **Wrong outcome** |
| A.420 | Intracellular trafficking and secretion of adiponectin is dependent on GGA-coated vesicles | 10.1074/jbc.M511313200 | Did not directly assess a GLUT4 trafficking/exocytosis endpoint (vesicle mobilization, surface insertion, tethering, docking, fusion, or subcellular redistribution); it evaluated glucose uptake, signalling, expression, or a systemic phenotype only. | **Wrong outcome** |
| A.421 | Inverse relationship between GLUT-4 phosphorylation and its intrinsic activity |  | Did not provide a qualifying functional link between a molecular regulator and a defined GLUT4 exocytosis/trafficking step in the prespecified model scope. | **Wrong outcome** |
| A.422 | Investigating the role of palmitoylation in insulin action | 10.1111/dme.12091_1 | Conference abstract or meeting record without sufficiently detailed, full primary experimental methods and results for eligibility assessment. | **Ineligible publication type / insufficient primary data** |
| A.423 | Investigation of the expression and localization of glucose transporter 4 and fatty acid translocase/CD36 in equine skeletal muscle | 10.2460/ajvr.2004.65.951 | Human-only, non-target tissue, or non-eligible biological model; no qualifying mechanistic validation in adipocytes or skeletal muscle was reported. | **Wrong population/model** |
| A.424 | Investigations of intracellular Ca2+ concentrations in direct vicinity of GLUT4-storagevesicles in adipocytes | 10.1055/s-0044-1785408 | Did not provide a qualifying functional link between a molecular regulator and a defined GLUT4 exocytosis/trafficking step in the prespecified model scope. | **Wrong outcome** |
| A.426 | Involvement of the actin network in insulin signalling. |  | Did not directly assess a GLUT4 trafficking/exocytosis endpoint (vesicle mobilization, surface insertion, tethering, docking, fusion, or subcellular redistribution); it evaluated glucose uptake, signalling, expression, or a systemic phenotype only. | **Wrong outcome** |
| A.428 | IRAP is a key regulator of insulin-dependant GLUT4 trafficking by controlling the sorting of GLUT4 from endosomes to GLUT4-specialized compartments | 10.1091/mbc.E10-10-0839 | Did not provide a qualifying functional link between a molecular regulator and a defined GLUT4 exocytosis/trafficking step in the prespecified model scope. | **Wrong outcome** |
| A.431 | Isolation of GLUT4 storage vesicles. |  | Methods/assay report that did not test a molecular regulator or regulatory mechanism of GLUT4 trafficking in an eligible primary experimental study. | **Wrong study design** |
| A.432 | Key Facilitator Proteins that Mediate Sarcolemma Membrane Repair have Potential as Therapeutics for Muscle Disease and Injury | 10.1096/fasebj.2019.33.1_supplement.701.1 | Did not provide a qualifying functional link between a molecular regulator and a defined GLUT4 exocytosis/trafficking step in the prespecified model scope. | **Wrong outcome** |
| A.433 | Kinetic analysis of glucose transporter trafficking in fibroblasts and adipocytes | 10.1021/bi00047a018 | Computational, structural, or mathematical analysis without experimental validation in an eligible GLUT4-trafficking model. | **Wrong study design** |
| A.435 | Kinetics of GLUT4 trafficking in rat and human skeletal muscle | 10.2337/db08-1539 | Conference abstract or meeting record without sufficiently detailed, full primary experimental methods and results for eligibility assessment. | **Ineligible publication type / insufficient primary data** |
| A.437 | Knockdown of exocyst Sec10 in mouse skeletal muscle cells and analysis of insulin stimulated GLUT4 exocytosis |  | Did not provide a qualifying functional link between a molecular regulator and a defined GLUT4 exocytosis/trafficking step in the prespecified model scope. | **Wrong outcome** |
| A.438 | Knockdown of HIF-1alpha abrogates insulin-stimulated glucose uptake in the skeletal muscle cells | 10.2337/db11-379-477 | Conference abstract or meeting record without sufficiently detailed, full primary experimental methods and results for eligibility assessment. | **Ineligible publication type / insufficient primary data** |
| A.440 | Knockdown of protein phosphatase 1 regulatory subunit negatively affects insulin action | 10.2337/db13-1743-1824 | Conference abstract or meeting record without sufficiently detailed, full primary experimental methods and results for eligibility assessment. | **Ineligible publication type / insufficient primary data** |
| A.443 | Lack of CD2AP Disrupts Glucose Transporter 4 Trafficking and Attenuates Glucose Uptake |  | Did not provide a qualifying functional link between a molecular regulator and a defined GLUT4 exocytosis/trafficking step in the prespecified model scope. | **Wrong outcome** |
| A.444 | Large GLUT4 vesicles are stationary while locally and reversibly depleted during transient insulin stimulation of skeletal muscle of living mice imaging analysis of GLUT4-enhanced green fluorescent protein vesicle dynamics | 10.2337/db06-1578 | Conference abstract or meeting record without sufficiently detailed, full primary experimental methods and results for eligibility assessment. | **Ineligible publication type / insufficient primary data** |
| A.445 | LNK Participates in Insulin Resistance in Polycystic Ovary Syndrome by Regulating Adipose Glucose Transport |  | Human-only, non-target tissue, or non-eligible biological model; no qualifying mechanistic validation in adipocytes or skeletal muscle was reported. | **Wrong population/model** |
| A.446 | Long-term insulin treatment of 3T3-L1 adipocytes results mis-targeting of GLUT4: Implications for insulin-stimulated glucose transport | 10.1007/s001250051523 | Did not provide a qualifying functional link between a molecular regulator and a defined GLUT4 exocytosis/trafficking step in the prespecified model scope. | **Wrong outcome** |
| A.447 | Long-term treatment with TNF-a represses GKAP42 binding to IRS-1 causing impairment of insulin signaling through IRS-1 and insulin-dependent glucose uptake in 3T3-L1 adipocytes |  | Did not provide a qualifying functional link between a molecular regulator and a defined GLUT4 exocytosis/trafficking step in the prespecified model scope. | **Wrong outcome** |
| A.450 | Loss of cortical actin filaments in insulin-resistant skeletal muscle cells impairs GLUT4 vesicle trafficking and glucose transport | 10.1152/ajpcell.00107.2006 | Did not provide a qualifying functional link between a molecular regulator and a defined GLUT4 exocytosis/trafficking step in the prespecified model scope. | **Wrong outcome** |
| A.453 | Mapping sites of insulin regulation in Glut4 trafficking: Insulin regulates fusion, sequestration into and release from GSVS | 10.2337/db14-2574-2600 | Conference abstract or meeting record without sufficiently detailed, full primary experimental methods and results for eligibility assessment. | **Ineligible publication type / insufficient primary data** |
| A.454 | Measuring GLUT4 vesicle exocytosis using intracellular intravital microscopy | 10.1091/mbc.E15-09-0674 | Methods/assay report that did not test a molecular regulator or regulatory mechanism of GLUT4 trafficking in an eligible primary experimental study. | **Wrong study design** |
| A.455 | Mechanisms and roles of Foxo1-Akt negative feedback pathway in adipocytes | 10.2337/db20-1699-P | Conference abstract or meeting record without sufficiently detailed, full primary experimental methods and results for eligibility assessment. | **Ineligible publication type / insufficient primary data** |
| A.456 | Metformin attenuates hepatic insulin resistance in type-2 diabetic rats through PI3K/Akt/GLUT-4 signalling independent to bicuculline-sensitive GABAA receptor stimulation | 10.1080/13880209.2016.1268635 | Human-only, non-target tissue, or non-eligible biological model; no qualifying mechanistic validation in adipocytes or skeletal muscle was reported. | **Wrong population/model** |
| A.459 | Microtubule network is required for insulin signaling through activation of Akt/protein kinase B: evidence that insulin stimulates vesicle docking/fusion but not intracellular mobility | 10.1074/jbc.M607101200 | Did not directly assess a GLUT4 trafficking/exocytosis endpoint (vesicle mobilization, surface insertion, tethering, docking, fusion, or subcellular redistribution); it evaluated glucose uptake, signalling, expression, or a systemic phenotype only. | **Wrong outcome** |
| A.462 | Minimal hypothesis of insulin action consistent with a mathematical description of the spatial and temporal dynamics of Glut4 trafficking |  | Computational, structural, or mathematical analysis without experimental validation in an eligible GLUT4-trafficking model. | **Wrong study design** |
| A.463 | Molecular analysis of insulin resistance in isolated ventricular cardiomyocytes of obese Zucker rats | 10.1152/ajpendo.1997.273.1.e59 | Investigated a cardiac/cardiomyocyte or heart model; the final review scope was restricted to adipocyte and skeletal-muscle models. | **Wrong population/model** |
| A.464 | Molecular Basis of Insulin-Responsive GLUT4 Trafficking Systems Revealed by Single Molecule Imaging | 10.1111/j.1600-0854.2011.01279.x | Reported a GLUT4 trafficking observation but did not test an eligible molecular regulator or regulatory mechanism with a defined trafficking endpoint. | **Wrong outcome** |
| A.465 | Molecular basis of TBC1D1 for acquiring temporal insulin-responsive ability triggering glut4 translocation | 10.2337/db13-1-387 | Conference abstract or meeting record without sufficiently detailed, full primary experimental methods and results for eligibility assessment. | **Ineligible publication type / insufficient primary data** |
| A.466 | Molecular mechanisms controlling GLUT4 intracellular retention | 10.1091/mbc.E08-03-0236 | Did not provide a qualifying functional link between a molecular regulator and a defined GLUT4 exocytosis/trafficking step in the prespecified model scope. | **Wrong outcome** |
| A.467 | Molecular mechanisms of contraction-induced translocation of GLUT4 in isolated cardiomyocytes | 10.1016/s0002-9149(97)00461-x | Population: Isolated adult rat ventricular cardiomyocytes. | **Wrong population** |
| A.468 | Molecular processes regulating glucose transport by skeletal muscle in response to conjugated linoleic acid isomers |  | Did not provide a qualifying functional link between a molecular regulator and a defined GLUT4 exocytosis/trafficking step in the prespecified model scope. | **Wrong outcome** |
| A.477 | Muscle glycogen content affects insulin-stimulated glucose transport and protein kinase B activity | 10.1152/ajpendo.2000.279.5.e947 | Did not provide a qualifying functional link between a molecular regulator and a defined GLUT4 exocytosis/trafficking step in the prespecified model scope. | **Wrong outcome** |
| A.479 | mVps45 knockdown selectively modulates VAMP expression in 3T3-L1 adipocytes | 10.1080/19420889.2015.1026494 | Did not directly assess a GLUT4 trafficking/exocytosis endpoint (vesicle mobilization, surface insertion, tethering, docking, fusion, or subcellular redistribution); it evaluated glucose uptake, signalling, expression, or a systemic phenotype only. | **Wrong outcome** |
| A.483 | Myosin IIA promotes docking of GLUT4-containing vesicles, but not recycling endosomes, to the plasma membrane |  | Did not provide a qualifying functional link between a molecular regulator and a defined GLUT4 exocytosis/trafficking step in the prespecified model scope. | **Wrong outcome** |
| A.486 | N-wasp-cortactin signaling promotes skeletal muscle GLUT4 vesicle translocation | 10.2337/db16-1695-1770 | Conference abstract or meeting record without sufficiently detailed, full primary experimental methods and results for eligibility assessment. | **Ineligible publication type / insufficient primary data** |
| A.488 | NAD(P)H oxidase-dpendent H2O2 production and RyR-IP3R activation are required for insulin induced GLUT4 translocation and glucose uptake in skeletal muscle cells |  | Did not provide a qualifying functional link between a molecular regulator and a defined GLUT4 exocytosis/trafficking step in the prespecified model scope. | **Wrong outcome** |
| A.489 | Naturally occurring compensated insulin resistance selectively alters glucose transporters in visceral and subcutaneous adipose tissues without change in AS160 activation | 10.1016/j.bbadis.2011.02.007 | Did not directly assess a GLUT4 trafficking/exocytosis endpoint (vesicle mobilization, surface insertion, tethering, docking, fusion, or subcellular redistribution); it evaluated glucose uptake, signalling, expression, or a systemic phenotype only. | **Wrong outcome** |
| A.491 | Negative regulation of syntaxin4/SNAP-23/VAMP2-mediated membrane fusion by Munc18c In Vitro | 10.1371/journal.pone.0004074 | Artificial or heterologous experimental system was used without mechanistic validation in an eligible GLUT4-expressing adipocyte or skeletal-muscle model. | **Wrong population/model** |
| A.492 | New regulators of insulin-stimulated glut4 exocytosis | 10.2337/db19-191-OR | Conference abstract or meeting record without sufficiently detailed, full primary experimental methods and results for eligibility assessment. | **Ineligible publication type / insufficient primary data** |
| A.494 | NFAT-133 increases glucose uptake in L6 myotubes by activating AMPK pathway | 10.1016/j.ejphar.2015.11.006 | Did not directly assess a GLUT4 trafficking/exocytosis endpoint (vesicle mobilization, surface insertion, tethering, docking, fusion, or subcellular redistribution); it evaluated glucose uptake, signalling, expression, or a systemic phenotype only. | **Wrong outcome** |
| A.495 | Nocodazole Inhibits Insulin-stimulated Glucose Transport in 3T3-L1 Adipocytes via a Microtubule-independent Mechanism | 10.1074/jbc.M105452200 | Did not provide a qualifying functional link between a molecular regulator and a defined GLUT4 exocytosis/trafficking step in the prespecified model scope. | **Wrong outcome** |
| A.496 | Non-conventional myosin 1c (Myo1c), but Not CaMKII, is required for insulin stimulated GLUT4 translocation to the plasma membrane (PM) in myoblasts | 10.1091/mbc.E10-10-0839 | Did not provide a qualifying functional link between a molecular regulator and a defined GLUT4 exocytosis/trafficking step in the prespecified model scope. | **Wrong outcome** |
| A.497 | Novel mechanisms to ATP-dependent glucose uptake in skeletal muscle cells | 10.1096/fasebj.26.1_supplement.lb715 | Did not provide a qualifying functional link between a molecular regulator and a defined GLUT4 exocytosis/trafficking step in the prespecified model scope. | **Wrong outcome** |
| A.498 | Novel roles of the Akt substrate, AS47 in GH-induced inhibition of insulin-stimulated glucose uptake in adipocytes without affecting GLUT4 translocation to plasma membranes |  | Did not provide a qualifying functional link between a molecular regulator and a defined GLUT4 exocytosis/trafficking step in the prespecified model scope. | **Wrong outcome** |
| A.499 | Novel systems for dynamically assessing insulin action in live cells reveals heterogeneity in the insulin response | 10.1111/tra.12035 | Did not provide a qualifying functional link between a molecular regulator and a defined GLUT4 exocytosis/trafficking step in the prespecified model scope. | **Wrong outcome** |
| A.500 | Opposite effect of JAK2 on insulin-dependent activation of mitogen-activated protein kinases and Akt in muscle cells: Possible target to ameliorate insulin resistance | 10.2337/diabetes.55.04.06.db05-1265 | Did not provide a qualifying functional link between a molecular regulator and a defined GLUT4 exocytosis/trafficking step in the prespecified model scope. | **Wrong outcome** |
| A.504 | Overexpression of protein tyrosine phosphatase-α (PTP-α) but not PTP-κ inhibits translocation of GLUT4 in rat adipose cells | 10.1006/bbrc.1999.0183 | Did not provide a qualifying functional link between a molecular regulator and a defined GLUT4 exocytosis/trafficking step in the prespecified model scope. | **Wrong outcome** |
| A.506 | Overexpression of vesicle-associated membrane protein (VAMP) 3, but not VAMP2, protects glucose transporter (GLUT) 4 protein translocation in an in vitro model of cardiac insulin resistance | 10.1074/jbc.M112.363630 | Investigated a cardiac/cardiomyocyte or heart model; the final review scope was restricted to adipocyte and skeletal-muscle models. | **Wrong population/model** |
| A.507 | Overexpression of wild-type Akt1 promoted insulin-stimulated p70S6 kinase (p70S6K) activity and affected GSK3 beta regulation, but did not promote insulin-stimulated GLUT4 translocation or glucose transport in L6 myotubes |  | Did not provide a qualifying functional link between a molecular regulator and a defined GLUT4 exocytosis/trafficking step in the prespecified model scope. | **Wrong outcome** |
| A.511 | p38γ MAPK regulation of glucose transporter expression and glucose uptake in L6 myotubes and mouse skeletal muscle | 10.1152/ajpregu.00563.2003 | Did not directly assess a GLUT4 trafficking/exocytosis endpoint (vesicle mobilization, surface insertion, tethering, docking, fusion, or subcellular redistribution); it evaluated glucose uptake, signalling, expression, or a systemic phenotype only. | **Wrong outcome** |
| A.512 | Pak1 signals to p41-ARC and N-WASP in response to insulin to evoke actin remodeling and glucose uptake in skeletal muscle cells |  | Did not provide a qualifying functional link between a molecular regulator and a defined GLUT4 exocytosis/trafficking step in the prespecified model scope. | **Wrong outcome** |
| A.513 | Pantophysin is a phosphoprotein component of adipocyte transport vesicles and associates with GLUT4-containing vesicles | 10.1074/jbc.275.3.2029 | Did not provide a qualifying functional link between a molecular regulator and a defined GLUT4 exocytosis/trafficking step in the prespecified model scope. | **Wrong outcome** |
| A.514 | Passive stretching produces Akt- and MAPK-dependent augmentations of GLUT4 translocation and glucose uptake in skeletal muscles of mice | 10.1007/s00424-005-1512-5 | Did not provide a qualifying functional link between a molecular regulator and a defined GLUT4 exocytosis/trafficking step in the prespecified model scope. | **Wrong outcome** |
| A.515 | Perinuclear localization and insulin responsiveness of GLUT4 requires cytoskeletal integrity in 3T3-L1 adipocytes | 10.1074/jbc.M003432200 | Reported a GLUT4 trafficking observation but did not test an eligible molecular regulator or regulatory mechanism with a defined trafficking endpoint. | **Wrong outcome** |
| A.516 | Permissive action of protein kinase C-ζ in insulin-induced CD36- and GLUT4 translocation in cardiac myocytes | 10.1677/JOE-09-0046 | Investigated a cardiac/cardiomyocyte or heart model; the final review scope was restricted to adipocyte and skeletal-muscle models. | **Wrong population/model** |
| A.521 | Phosphorylation of the N-terminus of Syntaxin-16 controls interaction with mVps45 and GLUT4 trafficking in adipocytes | 10.7717/peerj.15630 | Did not provide a qualifying functional link between a molecular regulator and a defined GLUT4 exocytosis/trafficking step in the prespecified model scope. | **Wrong outcome** |
| A.522 | Phosphorylation of Tmod3 by Akt2 regulates GLUT4 translocation by remodeling cortical actin | 10.2337/db12-1-377 | Conference abstract or meeting record without sufficiently detailed, full primary experimental methods and results for eligibility assessment. | **Ineligible publication type / insufficient primary data** |
| A.523 | Phosphorylation of Tmod3 by AMPK Regulates GLUT4 translocation and glucose transport in l6 myoblasts |  | Did not provide a qualifying functional link between a molecular regulator and a defined GLUT4 exocytosis/trafficking step in the prespecified model scope. | **Wrong outcome** |
| A.524 | Phosphorylation pattern and biological activity of TBC1D4, the RabGAP regulating GLUT4 translocation | 10.1055/s-0041-1727357 | Did not provide a qualifying functional link between a molecular regulator and a defined GLUT4 exocytosis/trafficking step in the prespecified model scope. | **Wrong outcome** |
| A.525 | Photoactivation of GLUT4 translocation promotes glucose uptake via PI3-K/Akt2 signaling in 3T3-L1 adipocytes | 10.1142/S1793545813500673 | Did not provide a qualifying functional link between a molecular regulator and a defined GLUT4 exocytosis/trafficking step in the prespecified model scope. | **Wrong outcome** |
| A.529 | PIP3 but not PIP2 increases insulin stimulated glucose uptake and glucose utilization Mediated by the Activation of PKCξ/λ/GLUT4 signaling pathway in type 2 diabetic rat liver and 3T3L1 adipocytes | 10.2337/db12-1578-1720 | Conference abstract or meeting record without sufficiently detailed, full primary experimental methods and results for eligibility assessment. | **Ineligible publication type / insufficient primary data** |
| A.532 | Plasticity in v-snare requirements for GLUT4 trafficking in adipocytes |  | Did not provide a qualifying functional link between a molecular regulator and a defined GLUT4 exocytosis/trafficking step in the prespecified model scope. | **Wrong outcome** |
| A.533 | Platelet-derived growth factor inhibits insulin stimulation of insulin receptor substrate-1-associated phosphatidylinositol 3-kinase in 3T3-L1 adipocytes without affecting glucose transport | 10.1074/jbc.273.39.25139 | Did not provide a qualifying functional link between a molecular regulator and a defined GLUT4 exocytosis/trafficking step in the prespecified model scope. | **Wrong outcome** |
| A.536 | Possible role for gp160 in constitutive but not insulin-stimulated GLUT4 trafficking: dissociation of gp160 and GLUT4 localization | 10.1042/bj3300405 | Reported a GLUT4 trafficking observation but did not test an eligible molecular regulator or regulatory mechanism with a defined trafficking endpoint. | **Wrong outcome** |
| A.539 | Potential role of Rab4 in the regulation of subcellular localization of Glut4 in adipocytes | 10.1128/MCB.16.12.6879 | Did not provide a qualifying functional link between a molecular regulator and a defined GLUT4 exocytosis/trafficking step in the prespecified model scope. | **Wrong outcome** |
| A.542 | Protein kinase B phosphorylation of PIKfyve regulates the trafficking of GLUT4 vesicles | 10.1242/jcs.01517 | Did not provide a qualifying functional link between a molecular regulator and a defined GLUT4 exocytosis/trafficking step in the prespecified model scope. | **Wrong outcome** |
| A.547 | Protein kinase C-ζ phosphorylates insulin-responsive aminopeptidase in vitro at Ser-80 and Ser-91 | 10.1016/S0003-9861(02)00261-8 | Did not provide a qualifying functional link between a molecular regulator and a defined GLUT4 exocytosis/trafficking step in the prespecified model scope. | **Wrong outcome** |
| A.551 | Protein kinase D1 is essential for contraction-induced glucose uptake but is not involved in fatty acid uptake into cardiomyocytes | 10.1074/jbc.M111.281881 | Investigated a cardiac/cardiomyocyte or heart model; the final review scope was restricted to adipocyte and skeletal-muscle models. | **Wrong population/model** |
| A.553 | Protein phosphatase 2A negatively regulates insulin's metabolic signaling pathway by inhibiting Akt (protein kinase B) activity in 3T3-L1 adipocytes | 10.1128/MCB.24.19.8778-8789.2004 | Did not provide a qualifying functional link between a molecular regulator and a defined GLUT4 exocytosis/trafficking step in the prespecified model scope. | **Wrong outcome** |
| A.556 | Proteolytic cleavage of cellubrevin and vesicle-associated membrane protein (VAMP) by tetanus toxin does not impair insulin-stimulated glucose transport or GLUT4 translocation in rat adipocytes | 10.1042/bj3210233 | Did not provide a qualifying functional link between a molecular regulator and a defined GLUT4 exocytosis/trafficking step in the prespecified model scope. | **Wrong outcome** |
| A.557 | Proteomic analysis of GLUT4 storage vesicles reveals LRP1 to be an important vesicle component and target of insulin signaling | 10.1074/jbc.M109.040428 | Reported a GLUT4 trafficking observation but did not test an eligible molecular regulator or regulatory mechanism with a defined trafficking endpoint. | **Wrong outcome** |
| A.558 | Proteomic analysis of GLUT4 storage vesicles reveals tumor suppressor candidate 5 (TUSC5) as a novel regulator of insulin action in adipocytes | 10.1074/jbc.M115.657361 | Did not provide a qualifying functional link between a molecular regulator and a defined GLUT4 exocytosis/trafficking step in the prespecified model scope. | **Wrong outcome** |
| A.559 | Proteomic analysis of protein palmitoylation in adipocytes | 10.4161/adip.22117 | Did not provide a qualifying functional link between a molecular regulator and a defined GLUT4 exocytosis/trafficking step in the prespecified model scope. | **Wrong outcome** |
| A.560 | Proximity ligation assay to study the GLUT4 membrane trafficking machinery | 10.1007/978-1-4939-7507-5_16 | Methods/assay report that did not test a molecular regulator or regulatory mechanism of GLUT4 trafficking in an eligible primary experimental study. | **Wrong study design** |
| A.561 | PYK2 as a Mediator of Endothelin-l/Gα11 Signaling to GLUT4 Glucose Transporters | 10.1074/jbc.c100524200 | Did not provide a qualifying functional link between a molecular regulator and a defined GLUT4 exocytosis/trafficking step in the prespecified model scope. | **Wrong outcome** |
| A.563 | Rab10 and Rab14 associate with insulin-responsive glut4 storage vesicles in adipocytes | 10.1091/mbc.E10-10-0839 | Did not provide a qualifying functional link between a molecular regulator and a defined GLUT4 exocytosis/trafficking step in the prespecified model scope. | **Wrong outcome** |
| A.565 | Rab10 in insulin-stimulated GLUT4 translocation | 10.1042/BJ20071318 | Did not provide a qualifying functional link between a molecular regulator and a defined GLUT4 exocytosis/trafficking step in the prespecified model scope. | **Wrong outcome** |
| A.566 | Rab10 regulates Glut4 cycling through endosomes and release from static retention, but not fusion to the plasma membrane | 10.2337/db11-379-477 | Conference abstract or meeting record without sufficiently detailed, full primary experimental methods and results for eligibility assessment. | **Ineligible publication type / insufficient primary data** |
| A.568 | Rab11 is associated with GLUT4-containing vesicles and redistributes in response to insulin | 10.1007/s001250051563 | Did not provide a qualifying functional link between a molecular regulator and a defined GLUT4 exocytosis/trafficking step in the prespecified model scope. | **Wrong outcome** |
| A.569 | Rab14 limits the sorting of Glut4 from endosomes into insulin-sensitive regulated secretory compartments in adipocytes | 10.1042/BCJ20160020 | Did not directly assess a GLUT4 trafficking/exocytosis endpoint (vesicle mobilization, surface insertion, tethering, docking, fusion, or subcellular redistribution); it evaluated glucose uptake, signalling, expression, or a systemic phenotype only. | **Wrong outcome** |
| A.570 | Rab4, but not the transferrin receptor, is colocalized with GLUT4 in an insulin-sensitive intracellular compartment in rat skeletal muscle | 10.1006/bbrc.1995.2469 | Reported a GLUT4 trafficking observation but did not test an eligible molecular regulator or regulatory mechanism with a defined trafficking endpoint. | **Wrong outcome** |
| A.571 | Rab4b is a small GTPase involved in the control of the glucose transporter GLUT4 localization in adipocyte | 10.1371/journal.pone.0005257 | Reported a GLUT4 trafficking observation but did not test an eligible molecular regulator or regulatory mechanism with a defined trafficking endpoint. | **Wrong outcome** |
| A.574 | RABIF/MSS4 is a Rab-stabilizing holdase chaperone required for GLUT4 exocytosis | 10.1073/pnas.1712176114 | Reported a GLUT4 trafficking observation but did not test an eligible molecular regulator or regulatory mechanism with a defined trafficking endpoint. | **Wrong outcome** |
| A.575 | Rabs 10, 14, and 8a/b are required for Glut4 translocation in adipocytes, but affect different trafficking steps | 10.2337/db12-1-377 | Conference abstract or meeting record without sufficiently detailed, full primary experimental methods and results for eligibility assessment. | **Ineligible publication type / insufficient primary data** |
| A.577 | Rac-1 superactivation triggers insulin-independent glucose transporter 4 (GLUT4) translocation that bypasses signaling defects exerted by c-Jun N-terminal kinase (JNK)- and ceramide-induced insulin resistance | 10.1074/jbc.M113.467647 | Did not provide a qualifying functional link between a molecular regulator and a defined GLUT4 exocytosis/trafficking step in the prespecified model scope. | **Wrong outcome** |
| A.579 | Rac1 activation is a major requirement for Glut4 translocation and glucose uptake in both muscle cells and mouse skeletal muscle |  | Did not provide a qualifying functional link between a molecular regulator and a defined GLUT4 exocytosis/trafficking step in the prespecified model scope. | **Wrong outcome** |
| A.580 | Rac1 governs exercise-stimulated glucose uptake in skeletal muscle through regulation of GLUT4 translocation in mice | 10.1113/JP272039 | Did not provide a qualifying functional link between a molecular regulator and a defined GLUT4 exocytosis/trafficking step in the prespecified model scope. | **Wrong outcome** |
| A.581 | Rac1 is a novel regulator of contraction-stimulated glucose uptake in skeletal muscle | 10.2337/db12-0491 | Conference abstract or meeting record without sufficiently detailed, full primary experimental methods and results for eligibility assessment. | **Ineligible publication type / insufficient primary data** |
| A.582 | Rac1 signaling is required for insulin-stimulated glucose uptake and is dysregulated in insulin-resistant murine and human skeletal muscle | 10.2337/db12-1148 | Conference abstract or meeting record without sufficiently detailed, full primary experimental methods and results for eligibility assessment. | **Ineligible publication type / insufficient primary data** |
| A.583 | Rac1-activation is a novel convergence-point between insulin and exercise-signaling in human skeletal muscle |  | Did not provide a qualifying functional link between a molecular regulator and a defined GLUT4 exocytosis/trafficking step in the prespecified model scope. | **Wrong outcome** |
| A.584 | RalA controls glucose homeostasis by regulating glucose uptake in brown fat | 10.1073/pnas.1801050115 | Did not provide a qualifying functional link between a molecular regulator and a defined GLUT4 exocytosis/trafficking step in the prespecified model scope. | **Wrong outcome** |
| A.586 | Rapid preparation of a plasma membrane fraction from adipocytes and muscle cells: Application to detection of translocated glucose transporter 4 on the plasma membrane | 10.1271/bbb.70342 | Methods/assay report that did not test a molecular regulator or mechanism of GLUT4 trafficking in an eligible primary experimental study. | **Wrong study design** |
| A.587 | Regulated dynamic subcellular GLUT4 localization revealed by proximal proteome mapping in human muscle cells | 10.1242/jcs.261454 | Did not provide a qualifying functional link between a molecular regulator and a defined GLUT4 exocytosis/trafficking step in the prespecified model scope. | **Wrong outcome** |
| A.588 | Regulation of cell surface GLUT1, GLUT3, and GLUT4 by insulin and IGF-I in L6 myotubes | 10.1016/0014-5793(95)00589-2 | Did not provide a qualifying functional link between a molecular regulator and a defined GLUT4 exocytosis/trafficking step in the prespecified model scope. | **Wrong outcome** |
| A.590 | Regulation of insulin-stimulated glucose transport by AMP-activated protein kinase in 3T3-L1 adipocytes | 10.1111/j.1464-5491.2011.03554_7.x | Did not provide a qualifying functional link between a molecular regulator and a defined GLUT4 exocytosis/trafficking step in the prespecified model scope. | **Wrong outcome** |
| A.592 | Regulation of multisite phosphorylation and 14-3-3 binding of AS160 in response to IGF-1, EGF, PMA and AICAR | 10.1042/BJ20070649 | Did not directly assess a GLUT4 trafficking/exocytosis endpoint (vesicle mobilization, surface insertion, tethering, docking, fusion, or subcellular redistribution); it evaluated glucose uptake, signalling, expression, or a systemic phenotype only. | **Wrong outcome** |
| A.595 | Regulation of recombinant TBC1D1, the RabGAP involved in GLUT4 translocation | 10.1055/s-0039-1688318 | Did not provide a qualifying functional link between a molecular regulator and a defined GLUT4 exocytosis/trafficking step in the prespecified model scope. | **Wrong outcome** |
| A.596 | Regulation of subcellular distribution of GLUT4 in cardiomyocytes: Rab4A reduces basal glucose transport and augments insulin responsiveness | 10.1055/s-0032-1329212 | Investigated a cardiac/cardiomyocyte or heart model; the final review scope was restricted to adipocyte and skeletal-muscle models. | **Wrong population/model** |
| A.597 | Regulation of the phosphorylation of FERM domain containing proteins moesin, radixin and ezrin by insulin | 10.1111/j.1464-5491.2009.02662.x | Did not provide a qualifying functional link between a molecular regulator and a defined GLUT4 exocytosis/trafficking step in the prespecified model scope. | **Wrong outcome** |
| A.598 | Regulation of the RalGAP complex by Akt-catalyzed phosphorylation | 10.1091/mbc.E11-10-0886 | Did not provide a qualifying functional link between a molecular regulator and a defined GLUT4 exocytosis/trafficking step in the prespecified model scope. | **Wrong outcome** |
| A.600 | Relationship between phosphorylation of Akt and AS160 on key regulatory sites and insulin-stimulated glucose uptake in rat skeletal muscle |  | Did not provide a qualifying functional link between a molecular regulator and a defined GLUT4 exocytosis/trafficking step in the prespecified model scope. | **Wrong outcome** |
| A.601 | Requirement for distinct vesicle-associated membrane proteins in insulin- and AMP-activated protein kinase (AMPK)-induced translocation of GLUT4 and CD36 in cultured cardiomyocytes | 10.1007/s00125-010-1832-7 | Investigated a cardiac/cardiomyocyte or heart model; the final review scope was restricted to adipocyte and skeletal-muscle models. | **Wrong population/model** |
| A.603 | Requirement for PIKfyve enzymatic activity in acute and long-term insulin cellular effects | 10.1210/en.2002-220615 | Did not directly assess a GLUT4 trafficking/exocytosis endpoint (vesicle mobilization, surface insertion, tethering, docking, fusion, or subcellular redistribution); it evaluated glucose uptake, signalling, expression, or a systemic phenotype only. | **Wrong outcome** |
| A.604 | Requirement of atypical protein kinase Cλ for insulin stimulation of glucose uptake but not for Akt activation in 3T3-L1 adipocytes | 10.1128/mcb.18.12.6971 | Did not provide a qualifying functional link between a molecular regulator and a defined GLUT4 exocytosis/trafficking step in the prespecified model scope. | **Wrong outcome** |
| A.606 | Retromer's role in insulin-stimulated glut4 trafficking in adipocytes | 10.1091/mbc.E12-10-0757 | Did not provide a qualifying functional link between a molecular regulator and a defined GLUT4 exocytosis/trafficking step in the prespecified model scope. | **Wrong outcome** |
| A.607 | RhoGDIα in the regulation of insulin-stimulated rac1 activity and glucose metabolism in skeletal muscle |  | Did not provide a qualifying functional link between a molecular regulator and a defined GLUT4 exocytosis/trafficking step in the prespecified model scope. | **Wrong outcome** |
| A.609 | RNAi-based analysis of CAP, Cbl, and CrkII function in the regulation of GLUT4 by insulin | 10.1074/jbc.C400180200 | Did not provide a qualifying functional link between a molecular regulator and a defined GLUT4 exocytosis/trafficking step in the prespecified model scope. | **Wrong outcome** |
| A.611 | Role for the microtubule cytoskeleton in GLUT4 vesicle trafficking and in the regulation of insulin-stimulated glucose uptake |  | Did not provide a qualifying functional link between a molecular regulator and a defined GLUT4 exocytosis/trafficking step in the prespecified model scope. | **Wrong outcome** |
| A.612 | Role for the microtubule cytoskeleton in GLUT4 vesicle trafficking and in the regulation of insulin-stimulated glucose uptake | Trùng bài A611 | Duplicate bibliographic record of an already-screened report; retained only once in the screening database. | **Duplicate record** |
| A.613 | Role of Akt substrate of 160 kDa in insulin-stimulated and contraction-stimulated glucose transport | 10.1139/H07-026 | Conference abstract or meeting record without sufficiently detailed, full primary experimental methods and results for eligibility assessment. | **Ineligible publication type / insufficient primary data** |
| A.614 | Role of clusters in insulin-regulated GLUT4 trafficking in adipose cells: a new paradigm? | 10.7150/ijbs.6.716 | Did not provide a qualifying functional link between a molecular regulator and a defined GLUT4 exocytosis/trafficking step in the prespecified model scope. | **Wrong outcome** |
| A.615 | Role of EHD1 and EHBP1 in perinuclear sorting and insulin-regulated GLUT4 recycling in 3T3-L1 adipocytes | 10.1074/jbc.M401918200 | Did not provide a qualifying functional link between a molecular regulator and a defined GLUT4 exocytosis/trafficking step in the prespecified model scope. | **Wrong outcome** |
| A.617 | Role of protein kinase C in the regulation of glucose transport in the rat adipose cell. Translocation of glucose transporters without stimulation of glucose transport activity |  | Did not directly assess a GLUT4 trafficking/exocytosis endpoint (vesicle mobilization, surface insertion, tethering, docking, fusion, or subcellular redistribution); it evaluated glucose uptake, signalling, expression, or a systemic phenotype only. | **Wrong outcome** |
| A.620 | Role of transverse tubules (T-tubules) in muscle glucose transport | 10.1007/978-1-4899-1928-1_3 | Reported a GLUT4 trafficking observation but did not test an eligible molecular regulator or regulatory mechanism with a defined trafficking endpoint. | **Wrong outcome** |
| A.622 | Roles of insulin, guanosine 5'-[γ-thio]triphosphate and phorbol 12-myristate 13-acetate in signalling pathways of GLUT4 translocation |  | Did not provide a qualifying functional link between a molecular regulator and a defined GLUT4 exocytosis/trafficking step in the prespecified model scope. | **Wrong outcome** |
| A.623 | Roles of PI 3-kinase and Ras on insulin-stimulated glucose transport in 3T3-L1 adipocytes | 10.1152/ajpendo.1997.272.2.e326 | Did not provide a qualifying functional link between a molecular regulator and a defined GLUT4 exocytosis/trafficking step in the prespecified model scope. | **Wrong outcome** |
| A.624 | Roles of sortilin-mediated retrograde trafficking in insulin-responsive Glut4 trafficking systems analyzed with single molecule imaging of Glut4 behavior | 10.1091/mbc.E10-10-0839 | Did not provide a qualifying functional link between a molecular regulator and a defined GLUT4 exocytosis/trafficking step in the prespecified model scope. | **Wrong outcome** |
| A.626 | SEC16A is a RAB10 effector required for insulin-stimulated GLUT4 trafficking in adipocytes | 10.1083/jcb.201509052 | Reported a GLUT4 trafficking observation but did not test an eligible molecular regulator or regulatory mechanism with a defined trafficking endpoint. | **Wrong outcome** |
| A.627 | Separation of insulin signaling into distinct GLUT4 translocation and activation steps | 10.1128/MCB.24.17.7567-7577.2004 | Did not provide a qualifying functional link between a molecular regulator and a defined GLUT4 exocytosis/trafficking step in the prespecified model scope. | **Wrong outcome** |
| A.628 | Septin 7 reduces nonmuscle myosin IIA activity in the SNAP23 complex and hinders GLUT4 storage vesicle docking and fusion | 10.1016/j.yexcr.2016.12.010 | Reported a GLUT4 trafficking observation but did not test an eligible molecular regulator or regulatory mechanism with a defined trafficking endpoint. | **Wrong outcome** |
| A.629 | Sequestosome 1/P62, a novel IRS-1 interacting protein, regulates insulin stimulated glucose uptake | 10.2337/db12-1578-1720 | Conference abstract or meeting record without sufficiently detailed, full primary experimental methods and results for eligibility assessment. | **Ineligible publication type / insufficient primary data** |
| A.630 | Serine 474 phosphorylation is essential for maximal Akt2 kinase activity in adipocytes | 10.1074/jbc.RA119.010036 | Did not provide a qualifying functional link between a molecular regulator and a defined GLUT4 exocytosis/trafficking step in the prespecified model scope. | **Wrong outcome** |
| A.632 | Signals and subcellular steps in GLUT4 translocation affected in insulin resistance |  | Did not provide a qualifying functional link between a molecular regulator and a defined GLUT4 exocytosis/trafficking step in the prespecified model scope. | **Wrong outcome** |
| A.633 | Single Point Mutations Result in the Miss-Sorting of Glut4 to a Novel Membrane Compartment Associated with Stress Granule Proteins | 10.1371/journal.pone.0068516 | Did not provide a qualifying functional link between a molecular regulator and a defined GLUT4 exocytosis/trafficking step in the prespecified model scope. | **Wrong outcome** |
| A.634 | SIRT6 as a potential target for treating insulin resistance | 10.1016/j.lfs.2019.116558 | Did not directly assess a GLUT4 trafficking/exocytosis endpoint (vesicle mobilization, surface insertion, tethering, docking, fusion, or subcellular redistribution); it evaluated glucose uptake, signalling, expression, or a systemic phenotype only. | **Wrong outcome** |
| A.636 | Skeletal muscle cells and adipocytes differ in their reliance on TC10 and Rac for insulin-induced actin remodeling | 10.1210/me.2003-0294 | Did not directly assess a GLUT4 trafficking/exocytosis endpoint (vesicle mobilization, surface insertion, tethering, docking, fusion, or subcellular redistribution); it evaluated glucose uptake, signalling, expression, or a systemic phenotype only. | **Wrong outcome** |
| A.638 | SNAP-23 participates in SNARE complex assembly in rat adipose cells |  | Did not directly assess a GLUT4 trafficking/exocytosis endpoint (vesicle mobilization, surface insertion, tethering, docking, fusion, or subcellular redistribution); it evaluated glucose uptake, signalling, expression, or a systemic phenotype only. | **Wrong outcome** |
| A.639 | SNAP23 promotes insulin-dependent glucose uptake in 3T3-L1 adipocytes: Possible interaction with cytoskeleton | 10.1152/ajpcell.1999.276.5.c1108 | Did not provide a qualifying functional link between a molecular regulator and a defined GLUT4 exocytosis/trafficking step in the prespecified model scope. | **Wrong outcome** |
| A.641 | SNARE expression and distribution during 3T3-L1 adipocyte differentiation | 10.1016/S0014-5793(02)02278-0 | Did not provide a qualifying functional link between a molecular regulator and a defined GLUT4 exocytosis/trafficking step in the prespecified model scope. | **Wrong outcome** |
| A.642 | SNARE-binding protein synaptosomal-associated protein of 29 kDa (SNAP29) regulates the intracellular sequestration of glucose transporter 4 (GLUT4) vesicles in adipocytes | 10.1111/jdi.13912 | Did not provide a qualifying functional link between a molecular regulator and a defined GLUT4 exocytosis/trafficking step in the prespecified model scope. | **Wrong outcome** |
| A.643 | Sorbitol activates atypical protein kinase C and GLUT4 glucose transporter translocation/glucose transport through proline-rich tyrosine kinase-2, the extracellular signal-regulated kinase pathway and phospholipase D | 10.1042/0264-6021:3620665 | Did not provide a qualifying functional link between a molecular regulator and a defined GLUT4 exocytosis/trafficking step in the prespecified model scope. | **Wrong outcome** |
| A.644 | Sortilin is a major protein component of Glut4-containing vesicles | 10.1074/jbc.272.39.24145 | Did not provide a qualifying functional link between a molecular regulator and a defined GLUT4 exocytosis/trafficking step in the prespecified model scope. | **Wrong outcome** |
| A.645 | Sorting of GLUT4 into its insulin-sensitive store requires the Sec1/Munc18 protein mVps45 | 10.1091/mbc.E13-01-0011 | Did not provide a qualifying functional link between a molecular regulator and a defined GLUT4 exocytosis/trafficking step in the prespecified model scope. | **Wrong outcome** |
| A.648 | ß-catenin facilitates skeletal muscle insulin-stimulated glucose transport through the regulation of actin remodeling | 10.2337/db20-297-OR | Conference abstract or meeting record without sufficiently detailed, full primary experimental methods and results for eligibility assessment. | **Ineligible publication type / insufficient primary data** |
| A.649 | Stimulation of GLUT4 (glucose transporter isoform 4) storage vesicle formation by sphingolipid depletion | 10.1042/BJ20091529 | Did not provide a qualifying functional link between a molecular regulator and a defined GLUT4 exocytosis/trafficking step in the prespecified model scope. | **Wrong outcome** |
| A.650 | Stimulation of GLUT4 (glucose transporter isoform 4) storage vesicle formation by sphingolipid depletion | 10.1042/BJ20091529 | Duplicate bibliographic record of an already-screened report; retained only once in the screening database. | **Duplicate record** |
| A.651 | Stimulatory effect of paeoniflorin on adenosine A-1 receptors to increase the translocation of protein kinase C (PKC) and glucose transporter (GLUT 4) in isolated rat white adipocytes | 10.1016/S0024-3205(98)00112-X | Did not provide a qualifying functional link between a molecular regulator and a defined GLUT4 exocytosis/trafficking step in the prespecified model scope. | **Wrong outcome** |
| A.652 | Subcellular trafficking of GLUT4 in insulin target cells | 10.1006/scdb.1996.0034 | Did not provide a qualifying functional link between a molecular regulator and a defined GLUT4 exocytosis/trafficking step in the prespecified model scope. | **Wrong outcome** |
| A.653 | Submissive role of AS160 in Tbc1d1-mediated glut4 trafficking activation in response to Ca2+ and insulin | 10.2337/db14-1-388 | Conference abstract or meeting record without sufficiently detailed, full primary experimental methods and results for eligibility assessment. | **Ineligible publication type / insufficient primary data** |
| A.655 | Substrate trapping proteomics reveals novel mechanism for regulation of mTORC1 signaling by βTrCP-FNIP1/2-FLCN axis | 10.1158/1538-7445.AM2016-4543 | Did not provide a qualifying functional link between a molecular regulator and a defined GLUT4 exocytosis/trafficking step in the prespecified model scope. | **Wrong outcome** |
| A.656 | Substrates of semicarbazide-sensitive amine oxidase co-operate with vanadate to stimulate tyrosine phosphorylation of insulin-receptor-substrate proteins, phosphoinositide 3-kinase activity and GLUT4 translocation in adipose cells | 10.1042/0264-6021:3500171 | Did not provide a qualifying functional link between a molecular regulator and a defined GLUT4 exocytosis/trafficking step in the prespecified model scope. | **Wrong outcome** |
| A.657 | Super-resolution microscopy reveals the insulin-resistanceregulated reorganization of GLUT4 on plasma membranes | 10.1242/jcs.192450 | Did not provide a qualifying functional link between a molecular regulator and a defined GLUT4 exocytosis/trafficking step in the prespecified model scope. | **Wrong outcome** |
| A.658 | Suppression of PPAR-γ attenuates insulin-stimulated glucose uptake by affecting both GLUT1 and GLUT4 in 3T3-L1 adipocytes | 10.1152/ajpendo.00695.2006 | Did not directly assess a GLUT4 trafficking/exocytosis endpoint (vesicle mobilization, surface insertion, tethering, docking, fusion, or subcellular redistribution); it evaluated glucose uptake, signalling, expression, or a systemic phenotype only. | **Wrong outcome** |
| A.659 | Sustained AS160 and TBC1D1 phosphorylations in human skeletal muscle 30 min after a single bout of exercise | 10.1152/japplphysiol.00044.2014 | Human-only, non-target tissue, or non-eligible biological model; no qualifying mechanistic validation in adipocytes or skeletal muscle was reported. | **Wrong population/model** |
| A.662 | Synip phosphorylation does not regulate insulin-stimulated GLUT4 translocation | 10.1016/j.bbrc.2005.05.027 | Did not provide a qualifying functional link between a molecular regulator and a defined GLUT4 exocytosis/trafficking step in the prespecified model scope. | **Wrong outcome** |
| A.663 | Synip phosphorylation is required for insulin-stimulated Glut4 translocation | 10.1016/j.bbrc.2007.02.095 | Did not provide a qualifying functional link between a molecular regulator and a defined GLUT4 exocytosis/trafficking step in the prespecified model scope. | **Wrong outcome** |
| A.664 | Synip phosphorylation is required for insulin-stimulated Glut4 translocation and glucose uptake in podocyte | 10.1507/endocrj.EJ14-0099 | Did not provide a qualifying functional link between a molecular regulator and a defined GLUT4 exocytosis/trafficking step in the prespecified model scope. | **Wrong outcome** |
| A.666 | Syntaxin 16 controls the intracellular sequestration of GLUT4 in 3T3-L1 adipocytes | 10.1016/j.bbrc.2006.06.135 | Did not provide a qualifying functional link between a molecular regulator and a defined GLUT4 exocytosis/trafficking step in the prespecified model scope. | **Wrong outcome** |
| A.667 | Syntaxin 16 phosphorylation and its role in GLUT4 trafficking | 10.1111/dme.5_13048 | Conference abstract or meeting record without sufficiently detailed, full primary experimental methods and results for eligibility assessment. | **Ineligible publication type / insufficient primary data** |
| A.671 | Syntaxin 6 regulates Glut4 trafficking in 3T3-L1 adipocytes | 10.1091/mbc.E02-11-0722 | Did not provide a qualifying functional link between a molecular regulator and a defined GLUT4 exocytosis/trafficking step in the prespecified model scope. | **Wrong outcome** |
| A.674 | TBC1D1, a Rab-GTPase activating protein, is critical for maintaining βcell mass and glucose homeostasis |  | Did not provide a qualifying functional link between a molecular regulator and a defined GLUT4 exocytosis/trafficking step in the prespecified model scope. | **Wrong outcome** |
| A.676 | TBC1D15 affects glucose uptake by regulating GLUT4 translocation | 10.1016/j.gene.2018.10.025 | Did not provide a qualifying functional link between a molecular regulator and a defined GLUT4 exocytosis/trafficking step in the prespecified model scope. | **Wrong outcome** |
| A.678 | TC10 and insulin-stimulated glucose transport | 10.1016/S0076-6879(06)06055-1 | Did not provide a qualifying functional link between a molecular regulator and a defined GLUT4 exocytosis/trafficking step in the prespecified model scope. | **Wrong outcome** |
| A.682 | Temporal separation of insulin-stimulated GLUT4/IRAP vesicle plasma membrane docking and fusion in 3T3L1 adipocytes | 10.1074/jbc.274.52.37357 | Reported a GLUT4 trafficking observation but did not test an eligible molecular regulator or regulatory mechanism with a defined trafficking endpoint. | **Wrong outcome** |
| A.683 | The 5' AMP-activated protein kinase (AMPK)/Axin1-Rac1 signaling pathway mediates contraction-regulated glucose uptake in skeletal muscle cells | 10.1002/dmrr.3222 | Did not provide a qualifying functional link between a molecular regulator and a defined GLUT4 exocytosis/trafficking step in the prespecified model scope. | **Wrong outcome** |
| A.684 | The actin regulatory protein Tropomyosin 5NM1 regulates glucose uptake and GLUT4 trafficking in the mouse | 10.1091/mbc.E12-10-0757 | Did not provide a qualifying functional link between a molecular regulator and a defined GLUT4 exocytosis/trafficking step in the prespecified model scope. | **Wrong outcome** |
| A.686 | The amino terminus of insulin-responsive aminopeptidase causes Glut4 translocation in 3T3-L1 adipocytes | 10.1074/jbc.272.37.23323 | Did not provide a qualifying functional link between a molecular regulator and a defined GLUT4 exocytosis/trafficking step in the prespecified model scope. | **Wrong outcome** |
| A.687 | The antihyperglycemic drug α-lipoic acid stimulates glucose uptake via both GLUT4 translocation and GLUT4 activation: Potential role of p38 mitogen-activated protein kinase in GLUT4 activation | 10.2337/diabetes.50.6.1464 | Did not provide a qualifying functional link between a molecular regulator and a defined GLUT4 exocytosis/trafficking step in the prespecified model scope. | **Wrong outcome** |
| A.690 | The carboxy-terminal region of the TBC1D4 (AS160) RabGAP mediates protein homodimerization | 10.1016/j.ijbiomac.2017.05.119 | Did not directly assess a GLUT4 trafficking/exocytosis endpoint (vesicle mobilization, surface insertion, tethering, docking, fusion, or subcellular redistribution); it evaluated glucose uptake, signalling, expression, or a systemic phenotype only. | **Wrong outcome** |
| A.691 | The cytosolic C-terminus of the glucose transporter GLUT4 contains an acidic cluster endosomal targeting motif distal to the dileucine signal | 10.1042/0264-6021:3500099 | Did not provide a qualifying functional link between a molecular regulator and a defined GLUT4 exocytosis/trafficking step in the prespecified model scope. | **Wrong outcome** |
| A.692 | The deubiquitinating enzyme USP25 binds tankyrase and regulates trafficking of the facilitative glucose transporter GLUT4 in adipocytes | 10.1038/s41598-019-40596-5 | Did not provide a qualifying functional link between a molecular regulator and a defined GLUT4 exocytosis/trafficking step in the prespecified model scope. | **Wrong outcome** |
| A.693 | The effect of PKC on the mechanism underlying calcium signal-regulated GLUT4 translocation in skeletal muscle cells | 10.1002/dmrr.2604 | Did not provide a qualifying functional link between a molecular regulator and a defined GLUT4 exocytosis/trafficking step in the prespecified model scope. | **Wrong outcome** |
| A.694 | The endosomal compartment is an insulin-sensitive recruitment site for GLUT4 and GLUT1 glucose transporters in cardiac myocytes | 10.1210/endo.142.12.8555 | Investigated a cardiac/cardiomyocyte or heart model; the final review scope was restricted to adipocyte and skeletal-muscle models. | **Wrong population/model** |
| A.695 | The Endosomal pH Regulator NHE6 is Required for Insulin-Stimulated Glucose Uptake in Adipocytes | 10.1101/2023.07.30.551163 | Did not provide a qualifying functional link between a molecular regulator and a defined GLUT4 exocytosis/trafficking step in the prespecified model scope. | **Wrong outcome** |
| A.696 | The exocyst complex in insulin-stimulated glucose uptake in skeletal muscle cells |  | Did not provide a qualifying functional link between a molecular regulator and a defined GLUT4 exocytosis/trafficking step in the prespecified model scope. | **Wrong outcome** |
| A.699 | The exocytotic trafficking of TC10 occurs through both classical and nonclassical secretory transport pathways in 3T3L1 adipocytes | 10.1128/MCB.23.3.961-974.2003 | Did not directly assess a GLUT4 trafficking/exocytosis endpoint (vesicle mobilization, surface insertion, tethering, docking, fusion, or subcellular redistribution); it evaluated glucose uptake, signalling, expression, or a systemic phenotype only. | **Wrong outcome** |
| A.700 | The F5QQI and T498ELEY motifs function in sorting GLUT4 to Its storage pool in adipocytes |  | Did not provide a qualifying functional link between a molecular regulator and a defined GLUT4 exocytosis/trafficking step in the prespecified model scope. | **Wrong outcome** |
| A.701 | The formin family protein, formin homolog overexpressed in spleen, interacts with the insulin-responsive aminopeptidase and profilin IIa | 10.1210/me.2003-0056 | Did not provide a qualifying functional link between a molecular regulator and a defined GLUT4 exocytosis/trafficking step in the prespecified model scope. | **Wrong outcome** |
| A.702 | The function of the exocyst is regulated by effector phosphorylation | 10.1091/mbc.E10-10-0839 | Did not provide a qualifying functional link between a molecular regulator and a defined GLUT4 exocytosis/trafficking step in the prespecified model scope. | **Wrong outcome** |
| A.703 | The glucose transporter (GLUT-4) and vesicle-associated membrane protein-2 (VAMP-2) are segregated from recycling endosomes in insulin- sensitive cells | 10.1083/jcb.134.3.625 | Reported a GLUT4 trafficking observation but did not test an eligible molecular regulator or regulatory mechanism with a defined trafficking endpoint. | **Wrong outcome** |
| A.704 | The glucose transporter 4 FQQI motif is necessary for akt substrate of 160-kilodalton-dependent plasma membrane translocation but not golgi-localized γ-ear-containing arf-binding protein-dependent entry into the insulin-responsive storage compartment | 10.1210/me.2006-0476 | Did not provide a qualifying functional link between a molecular regulator and a defined GLUT4 exocytosis/trafficking step in the prespecified model scope. | **Wrong outcome** |
| A.705 | The glucose transporter GluT4 and secretory carrier membrane proteins (SCAMPs) colocalize in rat adipocytes and partially segregate during insulin stimulation |  | Reported a GLUT4 trafficking observation but did not test an eligible molecular regulator or regulatory mechanism with a defined trafficking endpoint. | **Wrong outcome** |
| A.706 | The glucose transporter GLUT4 and the aminopeptidase vp165 colocalise in tubulo-vesicular elements in adipocytes and cardiomyocytes | 10.1242/jcs.110.18.2281 | Investigated a cardiac/cardiomyocyte or heart model; the final review scope was restricted to adipocyte and skeletal-muscle models. | **Wrong population/model** |
| A.707 | The Gly→Arg972 amino acid polymorphism in insulin receptor substrate-1 affects glucose metabolism in skeletal muscle cells | 10.1210/jc.85.5.2004 | Did not provide a qualifying functional link between a molecular regulator and a defined GLUT4 exocytosis/trafficking step in the prespecified model scope. | **Wrong outcome** |
| A.708 | The GTP-binding protein Rac does not couple PI 3-kinase to insulin-stimulated glucose transport in adipocytes | 10.1016/S0960-9822(95)00256-9 | Did not directly assess a GLUT4 trafficking/exocytosis endpoint (vesicle mobilization, surface insertion, tethering, docking, fusion, or subcellular redistribution); it evaluated glucose uptake, signalling, expression, or a systemic phenotype only. | **Wrong outcome** |
| A.710 | The inability of phosphatidylinositol 3-kinase activation to stimulate GLUT4 translocation indicates additional signaling pathways are required for insulin-stimulated glucose uptake | 10.1073/pnas.92.22.10247 | Did not provide a qualifying functional link between a molecular regulator and a defined GLUT4 exocytosis/trafficking step in the prespecified model scope. | **Wrong outcome** |
| A.711 | The interaction of Akt with APPL1 is required for insulin-stimulated Glut4 translocation | 10.1074/jbc.M704150200 | Did not provide a qualifying functional link between a molecular regulator and a defined GLUT4 exocytosis/trafficking step in the prespecified model scope. | **Wrong outcome** |
| A.712 | The luminal Vps10p domain of sortilin plays the predominant role in targeting to insulin-responsive glut4-containing vesicles | 10.1074/jbc.M608971200 | Did not provide a qualifying functional link between a molecular regulator and a defined GLUT4 exocytosis/trafficking step in the prespecified model scope. | **Wrong outcome** |
| A.713 | The mechanism of activation of the actin binding protein EHBP1 by Rab8 family members | 10.1038/s41467-020-17792-3 | Did not directly assess a GLUT4 trafficking/exocytosis endpoint (vesicle mobilization, surface insertion, tethering, docking, fusion, or subcellular redistribution); it evaluated glucose uptake, signalling, expression, or a systemic phenotype only. | **Wrong outcome** |
| A.714 | The mechanism of Ca2+ signal-regulated intracellular trafficking of GLUT4 in skeletal muscle cells | 10.1002/dmrr.2727 | Did not provide a qualifying functional link between a molecular regulator and a defined GLUT4 exocytosis/trafficking step in the prespecified model scope. | **Wrong outcome** |
| A.715 | The osmotic shock-induced glucose transpolar pathway in 3T3-L1 adipocytes is mediated by Gab-1 and requires Gab-1-associated phosphatidylinositol 3-kinase activity for full activation | 10.1016/s0021-9258(19)61455-9 | Did not provide a qualifying functional link between a molecular regulator and a defined GLUT4 exocytosis/trafficking step in the prespecified model scope. | **Wrong outcome** |
| A.716 | The participation of microtubule in insulin-mediated glucose transport |  | Did not provide a qualifying functional link between a molecular regulator and a defined GLUT4 exocytosis/trafficking step in the prespecified model scope. | **Wrong outcome** |
| A.717 | The persistent effect of prior exercise on insulin-stimulated glucose transport in rat skeletal muscle is accompanied by sustained and simultaneously increased phosphorylation of AS160 on two key regulatory sites |  | Did not provide a qualifying functional link between a molecular regulator and a defined GLUT4 exocytosis/trafficking step in the prespecified model scope. | **Wrong outcome** |
| A.718 | The pivotal role of protein kinase C zeta (PKCzeta) in insulin- and AMP-activated protein kinase (AMPK)-mediated glucose uptake in muscle cells | 10.1016/j.cellsig.2010.05.020 | Did not directly assess a GLUT4 trafficking/exocytosis endpoint (vesicle mobilization, surface insertion, tethering, docking, fusion, or subcellular redistribution); it evaluated glucose uptake, signalling, expression, or a systemic phenotype only. | **Wrong outcome** |
| A.719 | The potential role of protein kinaseB/Akt substrate 160 in the process of aerobic exercises improving glucose transporter 4 trafficking | 10.3969/j.issn.1001-1242.2010.06.003 | Full report was not published in English, whereas the prespecified eligibility criteria included English-language reports only. | **Ineligible publication language** |
| A.720 | The Rab GTPase-activating protein AS160 as a common regulator of insulin- and Gαq-mediated intracellular GLUT4 vesicle distribution | 10.1507/endocrj.K08E-216 | Did not provide a qualifying functional link between a molecular regulator and a defined GLUT4 exocytosis/trafficking step in the prespecified model scope. | **Wrong outcome** |
| A.723 | The RAB10 effector protein SEC16A regulates GLUT4 trafficking in adipocytes | 10.2337/db1518471928 | Did not provide a qualifying functional link between a molecular regulator and a defined GLUT4 exocytosis/trafficking step in the prespecified model scope. | **Wrong outcome** |
| A.724 | The RalGap complex regulates rala activity and Glut4 vesicle trafficking in adipocytes | 10.1091/mbc.E10-10-0839 | Did not provide a qualifying functional link between a molecular regulator and a defined GLUT4 exocytosis/trafficking step in the prespecified model scope. | **Wrong outcome** |
| A.725 | The Ras signaling pathway mimics insulin action on glucose transporter translocation | 10.1073/pnas.90.10.4460 | Did not provide a qualifying functional link between a molecular regulator and a defined GLUT4 exocytosis/trafficking step in the prespecified model scope. | **Wrong outcome** |
| A.726 | The role for the retromer complex in GLUT4 trafficking and degradation in adipocytes |  | Did not provide a qualifying functional link between a molecular regulator and a defined GLUT4 exocytosis/trafficking step in the prespecified model scope. | **Wrong outcome** |
| A.728 | The role of the Insulin regulated aminopeptidase and the Rab-GAP AS160 in Glut4 trafficking |  | Did not provide a qualifying functional link between a molecular regulator and a defined GLUT4 exocytosis/trafficking step in the prespecified model scope. | **Wrong outcome** |
| A.729 | The role of the small GTPase Rab14 in GLUT4 trafficking in adipocytes | 10.1111/j.1464-5491.2011.03233_1.x | Did not provide a qualifying functional link between a molecular regulator and a defined GLUT4 exocytosis/trafficking step in the prespecified model scope. | **Wrong outcome** |
| A.730 | The role of vesicle associated SNAREs (vSNAREs) in sorting of glucose transporter 4 (GLUT4) into the insulin responsive compartment | 10.1111/dme.12091_1 | Conference abstract or meeting record without sufficiently detailed, full primary experimental methods and results for eligibility assessment. | **Ineligible publication type / insufficient primary data** |
| A.731 | The role of β-and γ-actin in insulin-stimulated glucose transport | 10.2337/db16-1695-1770 | Conference abstract or meeting record without sufficiently detailed, full primary experimental methods and results for eligibility assessment. | **Ineligible publication type / insufficient primary data** |
| A.734 | The snare protein SNAP23 and the snare interacting protein Munc18c in human skeletal muscle are implicated in insulin sensitivity |  | Did not provide a qualifying functional link between a molecular regulator and a defined GLUT4 exocytosis/trafficking step in the prespecified model scope. | **Wrong outcome** |
| A.735 | The SUMO conjugating enzyme Ubc9 is a regulator of GLUT4 turnover and targeting to the insulin-responsive storage compartment in 3T3-L1 adipocytes | 10.2337/db06-1100 | Conference abstract or meeting record without sufficiently detailed, full primary experimental methods and results for eligibility assessment. | **Ineligible publication type / insufficient primary data** |
| A.739 | The TBC1D1 interactome: Integrated role for the two phosphotyrosine binding (PTB) domains in protein and phosphoinositide binding | 10.1111/dme.5_13048 | Conference abstract or meeting record without sufficiently detailed, full primary experimental methods and results for eligibility assessment. | **Ineligible publication type / insufficient primary data** |
| A.741 | The translocation of the glucose transporter sub-types GLUT1 and GLUT4 in isolated fat cells is differently regulated by phorbol esters | 10.1042/bj2750597 | Did not provide a qualifying functional link between a molecular regulator and a defined GLUT4 exocytosis/trafficking step in the prespecified model scope. | **Wrong outcome** |
| A.746 | Thyroid hormone promotes insulin-induced glucose uptake by enhancing Akt phosphorylation and VAMP2 translocation in 3T3-L1 adipocytes | 10.1002/jcp.22613 | Did not provide a qualifying functional link between a molecular regulator and a defined GLUT4 exocytosis/trafficking step in the prespecified model scope. | **Wrong outcome** |
| A.747 | TIRFing out Studies on Glut4 Trafficking | 10.1016/j.devcel.2006.12.008 | Reported a GLUT4 trafficking observation but did not test an eligible molecular regulator or regulatory mechanism with a defined trafficking endpoint. | **Wrong outcome** |
| A.751 | Trafficking kinetics of the insulin-regulated membrane aminopeptidase in 3T3-L1 adipocytes | 10.1006/bbrc.1997.7459 | Did not directly assess a GLUT4 trafficking/exocytosis endpoint (vesicle mobilization, surface insertion, tethering, docking, fusion, or subcellular redistribution); it evaluated glucose uptake, signalling, expression, or a systemic phenotype only. | **Wrong outcome** |
| A.752 | Trafficking of Glut4-green fluorescent protein chimaeras in 3T3-L1 adipocytes suggests distinct internalization mechanisms regulating cell surface glut4 levels |  | Did not provide a qualifying functional link between a molecular regulator and a defined GLUT4 exocytosis/trafficking step in the prespecified model scope. | **Wrong outcome** |
| A.753 | Transient changes in four GLUT4 compartments in rat adipocytes during the transition, insulin-stimulated to basal: Implications for the GLUT4 trafficking pathway | 10.1021/bi026474n | Reported a GLUT4 trafficking observation but did not test an eligible molecular regulator or regulatory mechanism with a defined trafficking endpoint. | **Wrong outcome** |
| A.755 | Translocation of the glucose transporter GLUT4 in cardiac myocytes of the rat | 10.1073/pnas.88.17.7815 | Investigated a cardiac/cardiomyocyte or heart model; the final review scope was restricted to adipocyte and skeletal-muscle models. | **Wrong population/model** |
| A.756 | Tropomodulin3 as the link between insulin-activated AKT2 and cortical actin remodeling in preparation of GLUT4 exocytosis | 10.1080/19490992.2015.1031949 | Did not provide a qualifying functional link between a molecular regulator and a defined GLUT4 exocytosis/trafficking step in the prespecified model scope. | **Wrong outcome** |
| A.759 | TUG mediates GLUT4 translocation by AMP activated protein kinase in the heart |  | Investigated a cardiac/cardiomyocyte or heart model; the final review scope was restricted to adipocyte and skeletal-muscle models. | **Wrong population/model** |
| A.760 | Two compartments for insulin-stimulated exocytosis in 3T3-L1 adipocytes defined by endogenous ACRP30 and GLUT4 | 10.1083/jcb.146.3.609 | Reported a GLUT4 trafficking observation but did not test an eligible molecular regulator or regulatory mechanism with a defined trafficking endpoint. | **Wrong outcome** |
| A.763 | Tyrosine phosphorylation of Munc18c on residue 521 abrogates binding to Syntaxin 4 | 10.1186/1471-2091-12-19 | Did not directly assess a GLUT4 trafficking/exocytosis endpoint (vesicle mobilization, surface insertion, tethering, docking, fusion, or subcellular redistribution); it evaluated glucose uptake, signalling, expression, or a systemic phenotype only. | **Wrong outcome** |
| A.766 | Unique regulatory mechanism of TBC1D1 RabGap in glut4 trafficking revealed by single molecule imaging of glut4 behavior | 10.1091/mbc.E12-10-0757 | Did not provide a qualifying functional link between a molecular regulator and a defined GLUT4 exocytosis/trafficking step in the prespecified model scope. | **Wrong outcome** |
| A.767 | Upstream regulators of myosin ii during GLUT4-Mediated glucose uptake in 3T3-L1 adipocytes | 10.1091/mbc.E10-10-0839 | Did not provide a qualifying functional link between a molecular regulator and a defined GLUT4 exocytosis/trafficking step in the prespecified model scope. | **Wrong outcome** |
| A.769 | Use of rat myoblasts overexpressing AS160-v2 protein for the characterization of GLUT4 translocation activators |  | Did not provide a qualifying functional link between a molecular regulator and a defined GLUT4 exocytosis/trafficking step in the prespecified model scope. | **Wrong outcome** |
| A.773 | VAMP3 null mice display normal constitutive, insulin- and exercise-regulated vesicle trafficking | 10.1128/MCB.21.5.1573-1580.2001 | Did not directly assess a GLUT4 trafficking/exocytosis endpoint (vesicle mobilization, surface insertion, tethering, docking, fusion, or subcellular redistribution); it evaluated glucose uptake, signalling, expression, or a systemic phenotype only. | **Wrong outcome** |
| A.774 | Variations in the requirement for v-SNAREs in GLUT4 trafficking in adipocytes | 10.1242/jcs.047449 | Did not provide a qualifying functional link between a molecular regulator and a defined GLUT4 exocytosis/trafficking step in the prespecified model scope. | **Wrong outcome** |
| A.776 | Vimentin binds IRAP and is involved in GLUT4 vesicle trafficking | 10.1016/j.bbrc.2010.12.134 | Did not provide a qualifying functional link between a molecular regulator and a defined GLUT4 exocytosis/trafficking step in the prespecified model scope. | **Wrong outcome** |
| A.777 | Visualization and quantitation of GLUT4 translocation in human skeletal muscle following glucose ingestion and exercise | 10.14814/phy2.12375 | Human-only, non-target tissue, or non-eligible biological model; no qualifying mechanistic validation in adipocytes or skeletal muscle was reported. | **Wrong population/model** |
| A.778 | Voltage-gated potassium channel Kv1.3 regulates GLUT4 trafficking to the plasma membrane via a Ca2+-dependent mechanism | 10.1152/ajpcell.00091.2005 | Did not provide a qualifying functional link between a molecular regulator and a defined GLUT4 exocytosis/trafficking step in the prespecified model scope. | **Wrong outcome** |
| A.779 | Where does insulin signalling intersect with the cellular trafficking machinery? | 10.1111/dme.14810 | Did not provide a qualifying functional link between a molecular regulator and a defined GLUT4 exocytosis/trafficking step in the prespecified model scope. | **Wrong outcome** |
| A.784 | β-catenin is important for the development of an insulin responsive pool of GLUT4 glucose transporters in 3T3-L1 adipocytes | 10.1016/j.yexcr.2018.03.011 | Did not provide a qualifying functional link between a molecular regulator and a defined GLUT4 exocytosis/trafficking step in the prespecified model scope. | **Wrong outcome** |
