# Supplementary material for: Molecular architecture of GLUT4 trafficking and exocytosis in adipocytes and skeletal muscle: A systematic review and integrated conceptual framework
Source: Front Endocrinol (Lausanne). 2026 Oct 19;17:1932455. doi: 10.3389/fendo.2026.1932455 (PMC13725575; doi:10.3389/fendo.2026.1932455)
Supplement: Supplementary file 6 [file Table4.docx]

**Table S4.** Evolution of experimental approaches in GLUT4 trafficking research. Summary of key methodological advances that have shaped the understanding of GLUT4 trafficking biology, including pioneering discoveries, experimental techniques, advantages, limitations, and emerging technologies applied across different trafficking components.

| **No.** | **Functional Group / Key Molecule** | **Year** | **Pioneering Author** | **Technique & Original Discovery Event** | **Advantages** | **Disadvantages** | **Subsequent Discovery/Measurement Techniques Used** | **Current direction** |
| --- | --- | --- | --- | --- | --- | --- | --- | --- |
| I. METABOLITES, CHEMICALS & FUNDAMENTAL MOLECULES | | | | | | | | |
| 1 | insulin | 1921 | F. Banting, C. Best | Pancreatic extract from dogs injected into pancreatectomized dog models, demonstrating the existence of a hypoglycemic hormone. | Direct demonstration of in vivo physiological function, possessing immediate clinical application value. | Crude extract, low purity, difficult to accurately quantify the initial molecular mechanism. | Plasma membrane lawn assay (1), Hyperinsulinemic-euglycemic clamp (2), Immunoblotting (3). | Utilization of super-resolution microscopy and single-molecule tracking in vivo. |
| 2 | Amino acid | 1806 | Vauquelin LN | Analytical chemistry: Isolation of Asparagine from asparagus (the first amino acid discovered). | Direct and precise for determining fundamental chemical properties. | Time-consuming, requires large sample volumes, fails to reflect dynamic biological functions. | Hindlimb perfusion technique, 2-DG uptake measurement, plasma membrane fractionation (4). | High-resolution mass spectrometry-based metabolomics combined with AI for structural prediction. |
| 3 | ATP | 1929 | Lohmann K | Biochemical analysis: First extraction and purification of the ATP nucleotide from muscle tissue. | Precise identification of the chemical structure of the energy-carrying molecule. | Extraction techniques may cause partial degradation of the substrate; complex procedures. | Firefly luciferase assay (bioluminescence measurement) (5). | Real-time FRET fluorescent biosensors (e.g., ATeam) in living cells. |
| 4 | PAF (Platelet-Activating Factor) | 1972 | J. Benveniste | Isolated from basophils, discovered as a specific soluble lipid factor capable of inducing platelet aggregation. | Identification of the specific cellular physiological origin. | Low yield, difficult to synthesize and preserve. | Surface Anti-c-MYC antibody binding assay, intracellular Ca2+ measurement using Fura-2 AM, cAMP measurement (6). | Mass spectrometry-based lipidomics for measuring ultra-trace level PAF fluctuations. |
| 5 | Ca2+/calmodulin | 1970 | Cheung WY | Protein purification: Extraction of the calcium-dependent phosphodiesterase activating factor. | Direct isolation of the intact functional protein. | Requires large target tissue volumes, difficult to observe intracellular kinetic changes. | Subcellular fractionation, RLC phosphorylation measurement using 32P (7), Biotinylated calmodulin binding assay (8). | Genetically encoded calcium indicators (GCaMPs) in live animal models. |
| 6 | GTPγS | 1971 | Eckstein F | Chemical synthesis: Creation of a non-hydrolyzable GTP analogue in the laboratory. | Provides a powerful tool to "lock" G-proteins in a constitutively active state. | An artificial compound, does not fully reflect the natural physiological cycle. | Plasma membrane sheet assay, in vitro AKT kinase activity measurement (9). | Design of optogenetic allosteric agonists/antagonists to control GTPases. |
| 7 | PIP2 (Diphosphoinositide) | 1949 | J. Folch | Applied solvent fractional extraction method on animal brain tissue, discovering an inositol lipid group containing two phosphate radicals. | Qualitative identification of a novel membrane lipid family. | Low sensitivity, unable to clearly distinguish positional isomers of the inositol ring. | Lipid fluorescence mixing assay, membrane permeability measurement (10). | Lipid-specific fluorescent probes (e.g., PH/FYVE domains) for in vivo observation. |
| 8 | PIP3 | 1988 | Traynor-Kaplan | In vivo lipid labeling: First detection of intracellularly generated PIP3 using TLC. | Accurately reflects lipid concentrations in live cell conditions. | Involves hazardous radioactivity, low cellular spatial resolution. | 6-NBDG uptake measurement, Flow cytometry (11), Enzyme activity/autophosphorylation assay (12). | Fluorescent PIP3 sensors and single-cell resolution lipidomics. |
| 9 | PI3P | 1988 | M. Whitman, L. Cantley | Thin-layer chromatography analysis of PI3-kinase enzyme reaction products, first discovery of inositol phospholipids phosphorylated at the D-3 position. | Gold standard method for separating lipid isomers. | Low sensitivity, sample preparation procedures easily lead to lipid loss. | TLC, Confocal microscopy with GFP-FYVE probe (13), HPLC (14). | Lipid-protein interaction analysis using in situ click chemistry technology. |
| 10 | PI5P | 1997 | L.E. Rameh | High-resolution HPLC analysis of lipid extracts, identifying a novel naturally occurring inositol lipid isomer within cells. | Extremely high resolution, capable of separating rare isomers. | High equipment cost, unable to map intracellular spatial localization. | In vitro lipid kinase assay, high-resolution HPLC, F-actin staining (15). | MALDI-Imaging mass spectrometry for mapping lipid distribution directly on tissues. |
| 11 | ATB-BMPA | 1990 | Clark AE | Chemical synthesis: Creation of a specific photolabeling agent for sugar transporters. | Specific and permanent binding to active transporters on the membrane. | Potential interference due to non-specific cross-linking. | Photolabeling combined with Immunoprecipitation, Kinetic modeling (16). | Use of photoactivatable fluorophores. |
| 12 | IP3 | 1983 | Streb, H., et al. | Bioinformatics screening to identify pleckstrin homology (PH) domain-containing proteins, followed by Northern blot to confirm expression. | Rapid discovery based on homologous regions. | Bioinformatics predictions require functional experimental validation. | Intracellular Ca2+ measurement using Fluo3-AM, Perforated patch-clamp technique (17). | Optical biosensors for real-time measurement of intracellular IP3 concentration (e.g., FIRE-1). |
| 13 | PDGF-BB | 1974 | Ross, R., et al. | Centrifugation and purification from blood platelets, combined with cell culture assays to measure proliferation levels. | Direct demonstration of the ability to stimulate proliferation in cultured cells. | Purity was low at the time, contaminated with other growth factors. | Colorimetric assay, Confocal microscopy (18). | Cryo-EM structural study of the ligand-receptor complex. |
| 14 | Irisin | 2012 | P. Boström | Mass spectrometry analysis: Discovery of a novel myokine cleaved from FNDC5 and secreted into the blood. | Objective discovery, precise peptide sequence identification. | Initial controversy regarding the existence of Irisin in humans due to poorly specific antibodies. | Intracellular ROS measurement (DCF-DA), Intracellular Ca2+ measurement (Fluo-3 AM) (19). | Utilization of Tandem Mass Spectrometry (Tandem MS) with precise isotope labeling. |
| 15 | AICAR | 1957 | J.G. Flaks, J.M. Buchanan | Purine biosynthesis pathway research: Identification of AICAR (ZMP) as a highly conserved natural intermediate metabolite. | Established a solid fundamental biochemical foundation. | Its role as an AMPK activator was not yet recognized. | Radioactive 2-DG uptake assay, Surface photolabeling (20), Intravital imaging in mice (21). | Metabolic impact assessment using PET/MRI imaging combined with radioisotopes. |
| II. RECEPTOR, CARGO & TRANSPORTERS | | | | | | | | |
| 1 | β-adrenergic receptor (GPCR) | 1981 | R.G. Shorr, R.J. Lefkowitz | Solubilization and affinity purification from frog erythrocyte membranes, first successful isolation of an intact G-protein coupled receptor. | First to maintain the coupling between the receptor and the G-protein. | Membrane structure disrupted, receptor integrity easily compromised. | The use of microinjection techniques for antibodies/mutant proteins (such as microinjection of anti-Gαq/11 antibodies or RGS2 protein) and PI3-kinase activity measurement is entirely accurate according to study (22). | Cryo-EM structural elucidation in the fully activated state. |
| 2 | CD-MPR | 1985 | B. Hoflack, S. Kornfeld | Affinity purification from macrophages and liver, discovery of a completely novel, cation-dependent mannose 6-phosphate recognizing receptor. | High specificity due to reliance on ligand affinity. | Difficult to preserve transient protein-protein interactions. | The use of Whole Mount Transmission Electron Microscopy combined with 3D Immuno-gold labeling technique to observe intracellular vesicles is confirmed in study (23). | Protein interaction network analysis (Interactomics) using BioID. |
| 3 | Adipsin | 1978 | P.H. Lesavre, H.J. Müller-Eberhard | Utilizing chromatography and purification from serum, isolation of a serine protease enzyme (later redefined as adipsin in adipose tissue). | Direct discovery of a circulating enzyme. | The enzyme's function was not immediately linked to adipose tissue metabolism. | Adipsin secretion measurement assay, Subcellular fractionation, Phospholipase D activity (24). | Tracking the secretion of extracellular vesicles (exosomes) containing the enzyme using NanoFCM. |
| 4 | Endothelin-1 (ET-1) | 1988 | Yanagisawa M | Biochemical purification: Isolation of a vasoconstricting peptide from endothelial cells. | Immediate identification of strong physiological properties (vasoconstriction). | Complex extraction process, extremely low peptide yield. | 2-Deoxyglucose uptake measurement, Immunoprecipitation & Western blotting (25). | AI-driven drug discovery for receptor antagonists. |
| 5 | LRP1 | 1988 | Herz J | Expression cloning: Discovery of an LDL receptor-related membrane protein. | Opened up possibilities for genetic functional and structural research. | Information regarding intracellular trafficking is limited. | Flow Cytometry, Kinetic Assays (Transferrin Efflux, α2-M Uptake), Mathematical modeling (26). | Tracking receptor recycling kinetics using super-resolution dSTORM. |
| 6 | GLUT4 | 1989 | James DE | cDNA cloning / 1F8 Antibody: Discovery of the insulin-sensitive glucose transporter. | Precise identification of the GLUT4-encoding gene and a pivotal antibody tool (1F8). | Inability to directly observe the real-time kinetics of GLUT4 vesicles. | TIRF microscopy, High-content imaging (27), Single-molecule tracking with Quantum dots (27-29), Pulse-chase (31) (30). | Single-molecule translocation measurement using highly automated multi-color TIRF. |
| 7 | GLUT1 | 1977 | M. Kasahara, P.C. Hinkle | Extraction and purification via dialysis filtration from human erythrocytes, first isolation of the D-glucose transport protein. | Successful isolation of the first fundamental transporter. | Artificial membrane structure differs from physiological membranes. | Immunogold labeling under electron microscopy, 3-O-methylglucose transport measurement (31), Photolabeling with ATB-BMPA (16). | Crystallographic conformational structure research. |
| 8 | IRAP (vp165) | 1995 | Keller SR | Microsomal purification: Isolation from GLUT4-containing vesicles (initially named vp165). | Discovered a perfect marker co-translocating with GLUT4. | Prolonged gradient centrifugation process, low yield. | Single-cell immunofluorescence translocation assay, HRP compartment ablation, Permeabilized cell assay (32). | Tracking pHluorin-IRAP to quantify membrane fusion events. |
| 9 | SORT1 | 1997 | Petersen CM | Affinity purification: Discovery of an intracellular receptor containing the Vps10p domain. | Identification of a novel transport receptor. | Its cargo was not immediately clearly identified. | In situ Proximity Ligation Assay (PLA), Fluorescence microscopy (33). | Creation of knock-in/knockout mouse models to analyze the TGN vesicle sorting system. |
| 10 | CDH15 (M-cadherin) | 1991 | M. Donalies | cDNA cloning, discovery of a specific adhesion molecule in muscle cells. | Muscle tissue-specific mapping. | Only demonstrated correlation, signaling mechanism unclear. | Surface GLUT4myc assay, F-actin/G-actin ratio measurement, Immunoprecipitation (34). | Observation of cytoskeletal structural binding using atomic force microscopy. |
| III. KINASE & PHOSPHATASE | | | | | | | | |
| 1 | PKC (Atypical PKC: ζ, λ) | 1977 | Nishizuka Y | Protein purification: Discovery of Diacylglycerol-dependent kinase enzyme. | Successful isolation of the pure enzyme and discovery of its dependence on factors such as Diacylglycerol for activation. | Time-consuming, low yield, and inability to observe the kinetic function of the enzyme in living cells or intact tissues. | Gene transfection/Adenoviral vectors for gene expression or suppression (35-37), Knockdown using siRNA (38), Subcellular fractionation combined with Western blot (35-37, 39), Confocal scanning microscopy (39, 40), In vitro kinase activity measurement/Immunoprecipitation kinase assay (35, 36, 41-43). | Combining genetic intervention (siRNA/viral vectors) with confocal fluorescence microscopy to map spatial localization and actin remodeling directly in living cells. |
| 2 | MLCK | 1974 | Pires E, Perry SV, Thomas MA | Enzyme purification (Gel filtration chromatography and electrophoresis from skeletal muscle tissue). | Direct purification from skeletal muscle tissue using gel filtration chromatography, revealing the fundamental biochemical activity of the enzyme. | Inability to observe the kinetic mechanism and regulation of this enzyme during intracellular vesicle trafficking. | Utilization of pharmacological inhibitors (ML-7, ML-9) (44), Knockdown using siRNA (45), Radioactive isotope labeling for glucose measurement combined with Western blot to measure RLC and MLCK phosphorylation levels (44-46), Confocal microscopy (44, 45). | Utilizing selective inhibitors (ML-7) and siRNA intervention combined with phosphorylation level assessment to demonstrate MLCK's role in activating MYH9 (non-muscle myosin IIA), facilitating the GLUT4 vesicle fusion process. |
| 3 | SRC (pp60SRC) | 1979 | Hunter T | Kinase Activity: Discovery of the first Tyrosine Kinase from the Rous sarcoma virus. | Provides the first evidence of an enzyme with Tyrosine Kinase activity, opening up the field of tyrosine phosphorylation research. | Primarily conducted in vitro or from viruses, failing to reflect how this molecule integrates into the intracellular signaling network of mammalian cells. | Gene depletion using Antisense morpholinos, expression of constitutively active mutants (CA-Src), observation via Epifluorescence and Confocal microscopy, activity measurement using SRC kinase assay with peptide substrates and lipid TLC (47). | Focusing on deciphering the mediating role of SRC in linking membrane receptors (e.g., β2-adrenergic receptors) with the PI3K signaling network and vesicle trafficking through real-time imaging observation. |
| 4 | c-YES (Src-family kinase) | 1980 | S. Kawai et al | Utilization of nucleic acid hybridization techniques and protein electrophoresis analysis from the avian Y73 sarcoma virus. | Viral nucleic acid hybridization techniques help identify transforming genes with kinase activity. | Viral studies fail to reflect specific physiological functions within the signal transduction networks of animal cells. | Microinjection of anti-YES antibodies (25), Expression of loss-of-function mutants (YES kinase-dead KR) (25), Measurement of 2-Deoxyglucose uptake and GLUT4 translocation (25). | Microinjection of specific antibodies and expression of inactivating mutants to isolate the role of c-YES, demonstrating it as an essential intermediate station in Endothelin-1 (ET-1) signaling leading to GLUT4 translocation. |
| 5 | PI3K (p110α, p110β, p85) | 1985 | Whitman M | Immunoprecipitation & TLC: Discovery of PI kinase activity associated with v-Src. | High specificity due to the use of antibodies, allowing direct detection of lipid kinase products. | Thin-layer chromatography (TLC) has limited resolution and sensitivity, making it difficult to quantify rapid dynamic lipid changes. | Microinjection of plasmids/antibodies (48, 49), Activity measurement using In vitro kinase assay/PI3K Activity Assay (49-51), Immunoelectron Microscopy and Confocal Fluorescence (52), Photoaffinity labeling combined with Kinetic Analysis (16). | Application of fluorescent biosensors to track the dynamics of phosphoinositide formation (such as PIP3) at membrane lipid rafts in living cells. |
| 6 | AMPK | 1987 | Carling D | Enzyme purification: AMPK was initially known as HMG-CoA reductase kinase. | Biochemical purification provides the fundamental characteristics of the enzyme (initially known as HMG-CoA reductase kinase). | Fails to reflect the "energy sensor" properties of the enzyme under physiological stress (muscle contraction, osmotic shock) in living models. | Glucose uptake measurement using 2-deoxy-D-[³H]glucose combined with the activator AICAR (1, 20, 53, 54), Confocal microscopy (55), Flow cytometry (56), Cell-surface photoaffinity labeling (20, 57), Activity assessment via measurement of ³²P incorporation into the SAMS peptide (54, 57). | Evaluation of AMPK's role in vivo and ex vivo in skeletal muscle using exercise-mimicking activators (AICAR, ZMP) combined with multiplexed glucose uptake measurements. |
| 7 | p38 MAPK | 1994 | J. Han | Macrophage stimulation with endotoxin (LPS) or osmotic shock, followed by isolation and identification of a novel 38 kDa protein kinase (named p38) with sharply increased tyrosine phosphorylation levels. | Identification of a novel protein that responds robustly to endotoxins or osmotic shock (physiological stress). | Labor-intensive isolation; difficult to identify the direct downstream targets of this kinase within the same experiment. | Antibody-coupled optical assay to quantify surface GLUT4, combined with In vitro kinase assays to measure the activity of α, β isoforms (58). | Utilizing selective inhibitors (e.g., SB203580) combined with kinetic assays to distinguish the role of p38 MAPK in activating the molecule's intrinsic activity versus the translocation process. |
| 8 | PLC-γ (PLC-γ1), PLD1 | 1988 | Suh PG | Brain library screening: Cloning of the first specific phospholipase. | Recombinant DNA technology allowed the decoding of the entire amino acid sequence of the molecule. | Merely possessing the gene sequence does not explain the function or interaction of the enzyme with cell membrane receptors. | Microinjection of fusion proteins (59), PLC activity assay on artificial liposomes via hydrolysis of [³H]-PI-(4,5)P2 (60), Measurement of DAG mass (60), Use of the U73122 inhibitor (59-61). | Measuring second messengers (DAG, IP3, Phosphatidic acid - PA) generated from PLC activity to map the signaling network connecting receptors with effector molecules (such as PKCζ). |
| 9 | PTP1B | 1988 | Tonks NK | Protein purification: Purification of the first human Protein-tyrosine phosphatase. | Structural determination and successful isolation of the first human phosphatase enzyme family. | Difficulties in identifying the true physiological target substrates of the enzyme within a complex cellular environment. | Transient transfection of catalytically inactive mutants (C/S mutants) acting as substrate traps (62), PTPase activity measurement via pNPP hydrolysis (62). | Creation of "substrate-trapping" mutants to isolate and identify target proteins, helping to distinguish the negative or positive regulatory roles of each phosphatase type. |
| 10 | GRK2 (β-ARK) | 1986 | Benovic JL | Biochemical isolation and purification of an enzyme from mutant lymphocytes. First identification of a novel protein kinase capable of specifically phosphorylating the β-adrenergic receptor only when it is bound to an agonist. | First successful isolation of an enzyme capable of specifically phosphorylating receptors already bound to agonists. | Purified biochemical assessment fails to describe the feedback interaction of the enzyme with membrane G-proteins in living cells. | Microinjection of anti-GRK2 antibodies, siRNA intervention, Adenoviral expression of kinase-dead mutants (K220R) or RGS domain-deleted mutants (D110A/K220R) (63), Immunofluorescence and Co-Immunoprecipitation (Co-IP) (63). | Expression of mutants lacking functional domains (e.g., the RGS domain) to identify GRK2 as an endogenous inhibitor of GLUT4 translocation through interference with Gαq/11 signaling. |
| 11 | PP2C-α | 1989 | Cohen P | Enzyme purification: Classification of Mg2+/Mn2+-dependent Protein Phosphatases. | Successful purification and classification of an enzyme belonging to the Mg2+/Mn2+-dependent Protein Phosphatase group. | Direct physiological targets of the enzyme in insulin-sensitive tissues were not yet identified. | Use of Adenoviral vectors to overexpress the wild-type (WT) or dominant-negative mutant (D174G-PP2C-α) (64), In vitro PI3K kinase activity measurement and Co-Immunoprecipitation (Co-IP) (64), Western blot (64). | Expression of phosphatase-dead mutants combined with Co-IP to demonstrate that PP2C-α directly interacts with and activates PI3K through dephosphorylation of the p85 subunit. |
| 12 | AKT (AKT1, AKT2, AKT3 / PKB) | 1991 | Bellacosa A | Sequencing + biochemical characterization of v-AKT as a serine/threonine kinase; concurrently, other groups cloned and expressed cellular PKB/RAC kinase via cDNA screening, in vitro translation, antisera, and kinase assays. | Gene sequencing provided the initial foundation for understanding this serine/threonine kinase family. | Lack of distinction in specific physiological functions between isoforms (AKT1 vs. AKT2) in specific tissues | Isoform-selective knockdown using siRNA/shRNA (38, 65-68), Microarray (38), TIRF microscopy (69, 70), Hierarchical cluster analysis (90) (69), CA/DN mutant expression using viral vectors (68, 71, 72), Heterodimerizer/Rapalog cross-activation system (73). | Specific gene silencing/activation (siRNA/CRISPR) and application of computational algorithms (TIRF tracking, Hierarchical clustering) to decouple the functions of individual isoforms (e.g., AKT2 is dominant in glucose metabolism). |
| 13 | FAK (pY397) | 1992 | Schaller MD | Anti-phosphotyrosine immunoblot: Identification of Focal Adhesion Kinase. | Phospho-tyrosine specific antibodies enable rapid detection of molecular activation. | Provides a static snapshot at a single point in time, failing to capture multidimensional spatial changes of the cytoskeleton. | Confocal microscopy tracking actin structures (74), siRNA intervention (74), RAC1 activity assay using G-LISA (75), Affinity purification combined with mass spectrometry (IP-MS) for protein interaction mapping (75). | Integration of Proteomics (mass spectrometry) systems to identify the interacting protein network (interactome) to understand how FAK coordinates actin remodeling and RAC1 signaling. |
| 14 | CADTK (Pyk2) | 1995 | S. Lev | Screening of a brain cDNA library to clone the gene encoding a novel tyrosine kinase homologous to FAK but activated by calcium signaling. | cDNA screening identified a novel tyrosine kinase activated by calcium signals. | Initial gene-level analysis lacked linkage to multiplexed signaling cascades such as exercise. | Cotransfection of dominant-negative/kinase-dead mutants (76, 77), Immune complex kinase activity measurement and PLD activity measurement (76, 77), testing with GTPγS stimulants combined with Western blot identifying tyrosine phosphorylation (9). | Application of kinase-inactive mutants and PLD activity measurements to establish the mediating role of PYK2/CADTK in linking non-insulin signals (e.g., glucose/muscle contraction) with the ERK/PLD pathway. |
| 15 | PFKFB3 | 1996 | Sakai A | cDNA cloning: Discovery of a glycolysis-regulating PFK-2 isozyme. | Successful cDNA cloning discovered the glycolysis-regulating isozyme at the molecular level. | Inability to observe the reverse regulatory relationship with the cell surface signaling network. | High-throughput screening using an siRNA library (78), Pharmacological inhibition using 3PO (78), ECAR measurement (Seahorse) and image analysis via IN Cell Analyzer (78), Western Blot assessing AKT/TBC1D4 phosphorylation (78). | Using a large-scale siRNA library and 3PO inhibition to demonstrate that PFKFB3-regulated glycolytic flux has a positive feedback effect on AKT activation and GLUT4 translocation. |
| 16 | TBK1 | 1999 | Pomerantz JL | Utilization of the yeast two-hybrid system and biochemical pull-down assays to identify a novel kinase (named TBK1) capable of interacting with the TANK protein, thereby activating the NF-κB signaling pathway. | The yeast two-hybrid system aided in identifying novel protein interactions with TANK. | In vitro assays failed to reflect the regulatory function on the exocytosis of the storage vesicle system. | Gene knockout using Cre-loxP (AKO) and gene knockdown using siRNA (79), Expression of inactive (K38A) and EXOC8 (Exo84) mutants (S8E/S8A) (79), Use of the Amlexanox inhibitor (79), LC-MS/MS analysis (79). | Utilizing knockout mouse models and targeted EXOC8 (Exo84) mutations to prove that TBK1 acts as a "gatekeeper", phosphorylating the exocyst complex to release GLUT4 vesicles for membrane fusion. |
| 17 | PDPK1 (PDK1) | 1997 | Alessi DR | Biochemical purification: Discovery of the kinase responsible for phosphorylating AKT. | Biochemical purification provided the intact enzyme for activity testing. | Difficult to investigate the binding of this molecule to membrane lipids (like PIP3) in vivo. | Cotransfection of PH domain-deleted mutant constructs (vPH-PDK1-myc) (80), PKB/AKT activity measurement via reaction with Crosstide substrate (80). | Utilizing mutant constructs with functional domain deletions (e.g., the PH domain) to demonstrate the necessity of lipid-dependent membrane localization in signal transduction. |
| 18 | PTEN | 1997 | Li J | Positional cloning: Discovery of a tumor suppressor gene (lipid phosphatase) on Chromosome 10. | Positional cloning discovered the gene locus and identified it as an important tumor suppressor gene. | Genetic analysis failed to demonstrate the enzyme's catalytic mechanism regarding lipid metabolism. | Use of Adenoviruses carrying the PTEN gene or microinjection of anti-PTEN antibodies (81), Membrane ruffling assay (rhodamine-phalloidin staining) (81), Quantification of PIP3 depletion levels (81). | Modulating PTEN expression levels using viral vectors to measure the negative feedback tuning capacity of the PI3K/AKT network and assess impacts on insulin sensitivity. |
| 19 | INPPL1 (SHIP2) | 1997 | Pesesse X | Identification and isolation based on the characteristics of an SH2 domain-containing protein with inositol phosphatase function. | Identification based on SH2 domain characteristics and in vitro inositol phosphatase function. | The isolated structure did not explain the kinetic impact of lipid degradation on insulin signaling. | Knockdown via electroporation-mediated siRNA (38), Adenoviral expression of phosphatase-deficient mutants (ΔIP-SHIP2) (82), Lipid analysis via HPLC (82), Glycogen synthesis measurement using radioisotopes (82). | Expression of loss-of-function mutants combined with HPLC analysis to directly measure the decrease in PI(3,4,5)P3, establishing the negative inhibitory role of SHIP2 in metabolic signaling. |
| 20 | PI4KA (PI4KIIIα), PIKFYVE, VAC14 (ArPIKfyve) | 1999 | Shisheva A | cDNA screening: Cloning of lipid metabolism kinases (homologs of yeast Fab1p). | cDNA screening facilitated the successful cloning of lipid metabolism kinases (Fab1p homologs). | Lack of an in vivo animal model to assess the overall physiological impact on tissue glucose metabolism. | Use of the selective inhibitor YM201636, lipid labeling with [γ-³²P]ATP and HPLC/TLC analysis (15, 83), Muscle-specific gene knockout (Cre-loxP MCK) combined with body composition measurement via EchoMRI and GTT/ITT (84). | Creation of a muscle-specific knockout mouse model (MPIfKO) combined with HPLC liquid chromatography to demonstrate that PIKFYVE and its PtdIns5P product are essential for maintaining prolonged AKT signaling in vivo. |
| 21 | DGK-ζ | 1996 | Bunting, M., et al. | cDNA library screening and protein detection using specific antibodies. | cDNA library screening helped discover a novel isoform of this lipid kinase. | Inability to investigate the kinetic linkages with other signaling proteins. | Yeast two-hybrid screening to find direct interactions with IRS1 (85), In vitro DGK and PIP5K enzyme activity measurements (85), Co-Immunoprecipitation (Co-IP). | Protein-protein interaction analysis (Yeast two-hybrid/Co-IP) to identify DGK-ζ as a signal-linking bottleneck (e.g., IRS1 binding) and a regulator of PIP2 synthesis. |
| 22 | CaMKII | 1983 | Kennedy, M. B., et al | Biochemical purification from rat brain homogenates, followed by Ca2+/Calmodulin-dependent enzyme activity assays. | Enzyme isolation demonstrated kinase activity directly dependent on Ca2+ and Calmodulin. | Static purification techniques failed to reflect the continuously fluctuating Calcium signaling in tissues like skeletal muscle. | Use of electrical pulse stimulation (EPS) models to simulate muscle contraction (86), 2-NBDG uptake assay (86), Use of KN-93 inhibitor or antCaNtide peptide (86, 87), DNA synthesis measurement ([³H]thymidine) (87). | Combining physiological stimulation models (electrical muscle contraction) with selective inhibitors and Calcium-luminescent agents to track real-time signaling. |
| 23 | MTM1 (myotubularin) | 1983 | Kennedy, M. B., et al. | Biochemical purification from rat brain homogenates, followed by Ca2+/Calmodulin-dependent enzyme activity assays. | Gene cloning identified a novel, highly conserved phosphatase family. | Encountered major limitations in identifying natural physiological lipid substrates in cells. | Radiolabeling with [³²P]orthophosphate and lipid composition analysis via HPLC (14), Adenoviral expression of "substrate-trapping" mutants (D278A) (14), Tracking via Confocal microscopy (14). | Applying substrate-trapping mutation technology combined with HPLC to accurately measure the depletion/accumulation of specific phosphoinositides (like PI3P). |
| 24 | PKD3 (PKC ν) | 1999 | A. Hayashi | Isolation and functional characterization of a novel human serine/threonine kinase isozyme. | Isolation of the novel isozyme provided the initial structural foundation for the serine/threonine kinase family. | Yet to distinguish basal state activity from activity under extracellular stimulation. | Use of Adenoviruses expressing wild-type PKD3 or dominant-negative mutants (DN-PKD3), Knockdown using siRNA (88), Activity measurement via In vitro kinase assay with peptide substrates (88), Confocal microscopy (88). | Applying Adenoviruses carrying dominant-negative mutant genes and siRNA to demonstrate that PKD3 maintains basal glucose uptake, operating completely independently of insulin signaling. |
| 25 | PAK1 | 1994 | E. Manser | Kinase purification from the brain: Discovery of PAK1 as the first serine/threonine kinase acting as a direct effector of RAC1 and CDC42. | Identification of PAK1 as a direct effector enzyme of small GTPases (RAC1, CDC42). | In vitro kinase assessment failed to demonstrate the complex cytoskeletal remodeling at the intact cellular scale. | Tracking actin structures in living cells (Live-cell imaging) using the LifeAct-GFP biosensor (89), Inhibition using the IPA3 molecule or autoinhibitory domain (PID) (89), Colorimetric/fluorescence measurement of GLUT4myc (89). | Utilizing biosensors in living cells to map the formation and remodeling of the cortical actin network in real-time. |
| 26 | WNK1 | 2000 | B.E. Xu | Sequencing and cDNA cloning from a gene expression library: Discovery of a completely novel serine/threonine kinase family (WNK) lacking the typical catalytic lysine in domain II. | Sequencing and cDNA cloning discovered a completely novel kinase family lacking the catalytic lysine in domain II. | The position of this enzyme within the complex intracellular signal transduction cascades was not yet determined. | Knockdown using siRNA (90), Use of pharmacological inhibitor WNK463 (90), Translocation measurement using Surface biotinylation assay (90), Western blotting to measure TBC1D4 phosphorylation (90). | Integrating a selective inhibitor (WNK463) and siRNA to map the signaling pathway, identifying WNK1 as an intermediary connecting signals from AKT to TBC1D4 (AS160) in skeletal muscle cells. |
| IV. GTPASES & EXCHANGE FACTORS (GEF/GAP) | | | | | | | | |
| 1 | p21ras (Ras) | 1979 | Shih TY | Utilized immunoprecipitation techniques to isolate for the first time a specific 21 kDa intracellular phosphoprotein (p21) from sarcoma virus-transformed cells. | First isolation of an oncogene-related small G-protein. | Required extremely sensitive antibodies; weak signal. | DNA synthesis measurement using BrdUrd, Microinjection of mutants (91). | KRAS G12C inhibitors, Nuclear Magnetic Resonance (NMR) structural analysis. |
| 2 | RAC1, RHOA, CDC42, TC10 (TC10α, TC10β), RALA | 1989 | Didsbury J | cDNA screening: Discovered a series of Rho/Ras family GTPases that control the cell membrane. | Established the entire subgroup of actin/membrane-regulating GTPases. | Lacked information regarding the physiological intracellular GTP/GDP-bound state. | GST-Pak1 PBD pull-down (92), In situ activation assay (polypeptide probe) (93), FRET imaging (Raichu-TC10) (94). | Utilizing multi-color FRET Biosensors to simultaneously observe multiple GTPases. |
| 3 | RAB GTPases (RAB3A, RAB3B, RAB3D, RAB4A, RAB4B, RAB8A, RAB10, RAB11, RAB14, RAB31, RAB35) | 1990 | Chavrier P | Library screening: Discovery of the Rab GTPase superfamily that controls intracellular vesicles. | Categorized the "zip codes" that guide intracellular vesicles. | Overwhelming number (>60 Rabs), difficult to determine the function of each type. | Bio-ATB-GTP photoaffinity analysis combined with MALDI-TOF MS (95), Dual-color TIRF, IRAP-pHluorin fusion assay (96). | Mapping the dynamic interactome using Proximity Labeling methods (BioID/APEX). |
| 4 | Gαq, Gα11 | 1991 | A Shenker | Used an antibody against the common C-terminus of αq/α11, identifying a 42-kDa protein in platelet membranes. | Identification of the Calcium-signaling G-protein family. | Difficult to isolate the complex with the receptor. | Single cell microinjection, Plasma membrane lawn assay (97). | Applying BRET to track G-protein dissociation in real-time. |
| 5 | GDI1, GDI2 | 1990 | Sasaki T | Protein purification: Discovery of the GTPase nucleotide dissociation inhibitor factor. | Discovered the regulatory factor for the Rab recycling cycle. | Biochemical purification required a large amount of bovine brains; low yield. | Acceptor photobleaching FRET (in vivo FRET) assessing dynamic interactions (98). | Cellular physiology analysis using fluorescent CRISPR-Cas9 knock-in. |
| 6 | TBC1D4 (AS160) | 2002 | Kane S | Used PAS antibodies to purify a 160 kDa protein that is a substrate phosphorylated by AKT in adipocytes. | Pivotal discovery: the bridge between AKT signaling and vesicle trafficking. | Required the use of antibodies recognizing phosphorylation motifs. | Exocytosis kinetics assay, HRP ablation assay (99), In vivo [³H]-2-DG uptake assay in mice (100). | Applying quantitative phosphoproteomics to decipher novel phosphorylation clusters. |
| 7 | TBC1D1 | 2007 | Roach WG | Screening for TBC1D4-homologous proteins, identified this as a RabGAP and a substrate of AKT/AMPK. | Discovered the intersection of AMPK and insulin signaling. | Dependent on initial bioinformatics analysis. | Single-molecule nanometry (quantum dots) (29), In vitro GAP Assay measurement (101). | Developing multi-point mutant knock-in mice to study metabolic physiology. |
| 8 | TBC1D13 | 2012 | Davey JR | Screened the TBC domain-containing protein group to identify this as a specific GAP for RAB35. | Accurately paired an orphan GAP with its target Rab (RAB35). | Could not initially assess multi-interactions in living cells. | In vivo GAP assay (RBD35 pull-down), in vitro GAP assay (102). | Establishing a 3D structural interaction model of TBC-Rab using AlphaFold. |
| 9 | PREX1 (P-Rex1) | 2002 | Welch HC | Biochemical purification and sequence analysis identified P-Rex1 as a PIP3- and Gβγ-regulated guanine-nucleotide exchange factor for RAC. | First discovery of a GEF co-regulated by PIP3 and G-proteins. | Complexity in reconstituting the in vitro lipid environment for testing. | Texas Red-phalloidin staining observing membrane ruffling structures (103). | Using allosteric activators/inhibitors to control intracellular P-Rex1. |
| 10 | DENND4C | 2011 | H. Sano | Initial identification via immunoprecipitation and in vitro assays demonstrating this is a specific nucleotide exchange factor (GEF) for RAB10. | Identified the key GEF activating RAB10 in adipocytes. | Demonstrating in vitro GEF activity was challenging. | RAB10 GEF Assay (32P labeling and TLC), SILAC phosphoproteomic (104). | Studying interaction crosstalk at membrane contact sites (MCS). |
| 11 | Gapex-5 | 2007 | I. J. Lodhi | Yeast two-hybrid system screening using the RAB31 protein as bait to isolate interacting molecules from a cDNA library. | Discovered the GEF regulating basal GLUT4 retention. | False positives from yeast required extensive experimental confirmation. | Rab activation assay (EEA1 pull-down), GST pull-down (105). | Creating the co-crystal structure of GAPVD1 and RAB31. |
| 12 | FLJ00068 | 2004 | T. Ota | Isolation and direct sequencing from clone libraries within the framework of the large-scale Full-Length Japan (FLJ) human gene sequencing project. | Discovered through large-scale sequencing, abundant raw data. | Lacked biochemical functional analysis upon initial discovery. | In situ RAC1 Activation Assay using polypeptide probes (106). | Conditional knockout to assess physiological role in skeletal muscle. |
| 13 | ARHGAP33 (TCGAP) | 2003 | Chiang SH | Yeast two-hybrid screening: Discovery of GAPs regulating TC10/CDC42 and Ral. | Linked CAP/CBL signaling with GTPase function. | Two-hybrid signal was weak, requiring confirmation via co-immunoprecipitation. | In vitro GAP assay, Protein-lipid overlay assay (107). | Super-resolution analysis of lipid raft microdomain structures. |
| 14 | RGC1, RGC2/AS250 | 2009 | Haipeng Cheng | Affinity purification from bovine brain extracts using an immobilized RALA protein column, followed by mass spectrometry analysis and gene cloning to identify a heterodimeric complex with GAP activity. | Resolved the mystery regarding the GAP regulating RALA. | Difficult to isolate large multi-subunit protein complexes. | Pull-down assay measuring GTP-RALA, In vitro GAP/kinase assay (108). | Evaluating interactions with the exocyst complex via mass spectrometry. |
| 15 | GIV (CCDC88A) | 2005 | Enomoto, A., et al. | Yeast two-hybrid screen using the AKT protein as bait. | Discovered a direct receptor signaling "enhancer" factor. | Complex structure, difficult to express in an intact recombinant form. | In situ Proximity Ligation Assay (PLA), Immunoisolation (109). | Developing drugs targeting the GIV-G protein interaction motif. |
| 16 | ARF | 1984 | Kahn, R. A., & Gilman, A. G. | Biochemical purification from rabbit liver as a necessary cofactor for cholera toxin activity. | Marked the birth of the GTPase family regulating vesicle budding. | Only known as a cofactor, cytological function unclear. | Quantitative fluorescence analysis, Surface-to-total ratio measurement (110). | Tracking ARF dynamics using bioluminescent sensors. |
| V. CYTOSKELETON & MOTOR PROTEINS | | | | | | | | |
| 1 | Tropomyosin TPM3.1, TMOD3 | 1948 | Bailey K | Chemical extraction: Purification of actin-regulating protein crystals from muscle. | Purified large quantities of fundamental regulatory proteins. | Destroyed tissue structure, providing no spatial information. | Illumina BeadArray, In vitro motility assays (111), Actin polymerization assay using pyrene (112). | Observing myofibril structures using multi-dimensional Cryo-EM. |
| 2 | Microtubules (α-tubulin, Tubulin) | 1967 | Borisy GG | Radioactive Colchicine binding: First isolation of pure microtubule proteins. | Discovered the main structural component of microtubules. | Required the use of hazardous radioactive materials. | Structured Illumination Microscopy (SIM), Muscle-on-a-chip model (113), 3D Parallax Microscopy (114) | Visualizing Nanoscale dynamics (elongation/shortening) optically. |
| 3 | actin | 1942 | Brúnó Ferenc Straub | Acetone-dried muscle powder extraction: This was Straub's breakthrough technique. He took rabbit muscle from which all myosin had been extracted (using a high-concentration salt solution), then treated the remaining muscle residue with acetone to dry it and remove lipids. Water extraction: When water was added to this dried muscle powder, a novel protein dissolved. Straub named it actin (from "activate") because it possessed the ability to activate myosin to generate muscle contraction. | Breakthrough in separating myosin and actin. | Crude, losing associated auxiliary proteins. | Actin-based Motility Assay in oocyte extracts, Time-lapse confocal YFP-actin (115, 116) | Studying membrane mechanical tension using FRET sensors. |
| 4 | Myosin II (MYH9, MYH10), MYO1C, MYO5A | 1989 | Shohet RV | cDNA cloning: Discovery of the Non-muscle Myosin motor superfamily. | Detailed classification of non-skeletal muscle motors. | Sequence data did not immediately prove motility. | In vitro ATPase assay (117), Cosedimentation assay (F-actin binding) (118), Actin free barbed-end assay (119). | Single-molecule motor tracking. |
| 5 | Kinesin | 1985 | Ronald Vale | Video-enhanced Differential Interference Contrast (AVEC-DIC) technique: This is an improved optical microscopy technique helping to observe small transport vesicles and microtubules that regular microscopes cannot see. | Direct visualization of vesicle transport on microtubules. | Difficult to immediately determine the biochemical properties of the motor. | GST-pull down (120), In vitro microtubule capture assay (121). | Designing Kinesin-based magnetically controlled nanoparticles. |
| 6 | KIF3A | 1994 | S. Kondo | Purification of protein complexes from neural tissue using specific antibodies and electron microscopy: successfully isolated KIF3A as a transport microtubule motor (kinesin). | Provided visual images of the motor structure. | Highly labor-intensive to purify from neural tissue. | Yeast two-hybrid screening, In vitro pull-down (122). | Optogenetic screening controlling kinesin directionality. |
| 7 | ACTN4 (α-actinin-4) | 1998 | K. Honda | Using molecular cloning and immunofluorescence techniques: successfully isolated the non-muscle α-actinin-4 isoform, which plays an important role in cell motility. | Clearly distinguished the skeletal muscle form and the cell motility form. | Easily confused with other actinin isoforms if the antibody is poor. | SILAC isotope labeling combined with quantitative LC-MS/MS (123), Immunoprecipitation (124). | Analyzing point mutations related to renal/metabolic pathology. |
| VI. SNARES, EXOCYST & VESICLE FUSION MACHINERY | | | | | | | | |
| 1 | clathrin | 1975 | Pearse BM | Centrifugation & Electrophoresis: Identification of the protein that forms the network coating intracellular vesicles. | Discovery of the first characteristic coat structure. | Required high-resolution electron microscopy. | Immunogold labeling under electron microscopy, Confocal laser immunofluorescence microscopy (125) | In vitro reconstitution of the clathrin lattice combined with Cryo-ET. |
| 2 | Exocyst complex: EXOC1 (Sec3), EXOC2 (Sec5), EXOC3 (Sec6), EXOC4 (Sec8), EXOC5 (Sec10), EXOC6/EXOC6B (Sec15 paralogs), EXOC7 (Exo70), EXOC8 (Exo84) | 1980 | Novick P | Screening for secretory mutants (sec mutants): Discovery in yeast of the membrane tethering complex. | An extremely powerful genetic method to find the entire pathway. | Yeast has distinct characteristics compared to higher animal cells. | Proximity ligation assay (PLA), Seahorse technology (metabolic measurement) (126), Nile red staining (127). | Resolving the structure of the 8-subunit holocomplex using Cryo-EM. |
| 3 | VAMP proteins: VAMP2, VAMP3 (cellubrevin), VAMP4, VAMP5, VAMP7, and VAMP8. | 1988 | Trimble WS | Expression library cloning: Discovery of v-SNAREs on secretory vesicles. | Discovery of the key synaptic vesicle membrane component. | Did not immediately identify the membrane fusion function. | Single-cell fluorescence assay (surface measurement) (128). | Real-time fusion pore recording using electrophysiology. |
| 4 | SNAP25 | 1989 | Oyler GA | Antibody screening: Membrane protein forming the ternary SNARE complex. | Generated powerful antibodies for histological diagnosis. | Not easy to purify the intact membrane protein. | PCR & Southern hybridization, Flow cytometry binding analysis (129). | Designing artificial SNAREs to direct membrane fusion. |
| 5 | CSP1 (cysteine-string protein 1) | 1990 | Zinsmaier KE | Antibody screening: Discovery of auxiliary proteins interacting with SNAREs. | Accidental discovery from Drosophila genetic research. | Required translation to mammalian models. | Subcellular fractionation (sucrose/iodixanol gradients), Plasma membrane lawn assay, Protein binding measurement (130). | Studying the chaperone (folding) role of Csp with the SNARE family. |
| 6 | VAP-33 | 1995 | P A Skehel | The combination of biochemical purification techniques (centrifugation, extraction), electron microscopy (ultrastructural observation), and molecular/genetic interaction screening (such as yeast two-hybrid systems or yeast mutants) to precisely identify the structural and regulatory "links" in the cellular machinery. | Identified the protein directly binding to v-SNARE. | Loose interactions are easily disrupted during extraction. | Single-cell GLUT4myc translocation assay technique (85). | Decoding the structure of the lipid-binding MSP domain. |
| 7 | STX4, STX16 | 1992 | Bennett MK | Gene library screening: Discovery of t-SNAREs acting as vesicle docking sites. | Identified t-SNAREs directing the fusion site. | Difficult to distinguish specificity between syntaxin isoforms. | In situ Proximity Ligation Assay (PLA), In vitro SNARE assembly assay (131), Lactate-Glo Assay (132). | Using pHluorin sensors to track the frequency and pattern of fusion (kiss-and-run). |
| 8 | p115 | 1995 | Sapperstein S | Functional screening: Protein acting as a tether at the Golgi apparatus. | Identified the tethering factor for Golgi system membranes. | Complex coiled molecular structure, difficult to study in vitro. | IRAP-CBD column affinity chromatography, Mass Spectrometry Analysis (133). | Creating synthetic membrane vesicles (liposomes) bearing p115. |
| 9 | tomosyn (including the b-tomosyn isoform) | 1998 | Fujita Y | SNARE complex purification: Protein that displaces Munc18 from Syntaxin. | Discovered a functional competitor to v-SNARE. | Difficult to separate splice variants. | Trans-SNARE disassembly assay (134), Phos-tag SDS-PAGE analysis (135). | Applying Cryo-EM to resolve the inhibitory binding structure. |
| 10 | SYNIP | 1999 | Min J | Yeast two-hybrid screening: Protein specifically binding to STX4. | Discovered an insulin-regulated inhibitory factor specific to STX4. | False positives from plasma membrane baits in yeast. | Nanodisc-liposome content mixing assay (136), Lipid-protein binding assay (137). | Evaluating SYNIP-membrane lipid interactions using spectroscopic tools. |
| 11 | Synaptobrevin | 1989 | W.S. Trimble, M. Baumert | Isolated from neural synaptic vesicles: discovery of an integral membrane protein playing a core role in the adhesion and fusion of secretory vesicles. | Discovery of the core integral membrane component. | Tightly bound to the membrane, difficult to solubilize. | Unified with VAMP measurement techniques (2). | Nanodisc technology containing single SNAREs. |
| VII. ADAPTORS, SCAFFOLDS & OTHER FACTORS | | | | | | | | |
| 1 | 14-3-3 (isoform γ) | 1967 | Moore BW | DEAE-cellulose chromatography: First discovered as acidic proteins in the brain. | Simple chromatography method detecting an abundant protein group. | Ambiguous name (14-3-3), took decades to clarify function. | Cell-free in vitro vesicle fusion assay, Live-cell fluorogenic assay (57), LC-MS/MS (48) | Measuring interaction with phosphoserine/threonine motifs via fluorescence polarization. |
| 2 | CBL, CBLB, APS (SH2B2), CAP (SORBS1), CRK, BCAR1 (p130Cas), flotillin | 1988 | Mayer BJ | Viral oncogene cloning: Discovery of SH2/SH3 domain-containing adaptor proteins. | Discovery of classical SH2/SH3 interaction domains. | Metabolic roles were not immediately identified. | Detergent-resistant membrane (DRM) fractionation via sucrose gradient centrifugation (138). | Analysis of ubiquitination and targeted protein degradation systems (PROTAC). |
| 3 | β-arrestin-1 | 1990 | Lohse MJ | Protein purification: Discovery of the protein blocking GPCR signaling. | Decoded the principle of receptor internalization. | Required a large amount of purified receptors in vitro. | Immunoprecipitation & Western blot (25). | Designing "Biased agonism" drugs prioritizing arrestin signaling. |
| 4 | IRS1, IRS2, IRS3, IRS4 | 1991 | Sun XJ | Expression cloning: First successful cloning of a direct substrate of the Insulin receptor. | Identification of the component directly receiving signals from the receptor. | Homologous regions made it difficult to separate the function of each IRS. | Radioimmunoassay measuring surface epitope-tagged GLUT4 (139). | Assessing serine phosphorylation levels (insulin resistance) using phosphoproteomics. |
| 5 | STEAP4, SAP97, PRKCSH (80K-H), TP53BP2 (53BP2S short isoform), ASIP | 1991 | Hirai M | cDNA library screening: Successively cloned scaffold proteins interacting with the cell surface. | Rapidly created a database of membrane scaffold proteins. | Static cDNA did not reveal the dynamic arrangement of complexes. | FRET (Fluorescence Resonance Energy Transfer) technique (140), Yeast two-hybrid screening (141). | Proximity Labeling technology for neighborhood mapping. |
| 6 | N4BP2L1, NAALADase, NCS1, Nexilin, Noc2 | 1993 | Israeli RS | cDNA cloning: (e.g., NAALADase cloned under the name Prostate-specific membrane antigen). | Discovered tissue-specific genes/proteins (e.g., prostate). | Functions were often confused until tested in other cell types. | ChIP, EMSA, Luciferase reporter, GLUT4-HiBiT luminescence assay, Glucose Uptake-Glo (142), MALDI-TOF MS (143). | Utilizing ultra-sensitive chemiluminescence systems (HiBiT) for kinetic measurements. |
| 7 | C3G | 1994 | Tanaka S | Library screening: Identified via interaction with the CRK protein. | Identified the connecting link for SH3 domains. | Dependent on the quality of the initial gene library. | In vitro Guanine nucleotide exchange assay (92). | Studying the role of PI3K-independent signal activation in insulin resistance. |
| 8 | Axin, Tankyrase, NRIP1 (RIP140), RAB11FIP5 (RIP11), RUVBL2 | 1995 | Cavaillès V | Positional cloning: Discovery of scaffold molecules and cofactors. | Discovered important transcriptional regulatory / scaffold proteins. | Difficult to preserve large complexes during purification. | Mammalian Tandem Affinity Purification (TAP) + MS (144) | Molecular pharmacology for specific inhibition of Tankyrase PARP activity. |
| 9 | CIP4/2, CLASP2, CLIPR-59, CDP138, EFR3, EHD2, DOC2B | 1997 | Aspenström P | Yeast two-hybrid screening: Protein scaffolds connecting lipids to membranes. | Connecting GTPases with the microtubule/actin cytoskeleton. | False bait phenomena could occur. | Super-resolution dSTORM analysis (145), HPLC–ESI–MS/MSⁿ mass spectrometry (146), Dual-color TIRFM (30) | Visualizing microtubule growth (microtubule plus-end tracking). |
| 10 | USP25M, CAPN10 (calpain-10) | 1999 | Valero R | cDNA cloning / GWAS: Discovery of Ubiquitin-cleaving enzymes and intracellular proteases. | Discovery of post-translational modification enzymes (ubiquitin/protein cleavage). | GWAS data required costly biochemical verification. | Biochemical substrate assays (147), Cell surface biotinylation (148) | Deubiquitinase inhibitors/activators restoring vesicle flux. |
| 11 | TUG (ASPSCR1) | 2001 | Ladanyi M | Chromosomal translocation analysis: Initially discovered as a gene related to Sarcoma tumors. | Identified from sarcoma pathology, applied to metabolism. | Initial metabolic role was overshadowed by cancer pathology. | Pulse-chase experiments analyzing degradation (30) | Study of USP25M-dependent TUG processing and GLUT4 vesicle mobilization in adipocytes. |
| 12 | miR-130a-3p | 2001 | Lagos-Quintana | Direct RNA cloning: Discovered during the first wave of miRNA publications. | Opened the era of micro-regulatory RNA research. | Extremely difficult to extract due to its very small size; easily degraded. | Wound healing assay, Tube formation, TUNEL staining, Immunohistochemistry (149) | Intravenous treatment with miRNA-containing exosomes. |
| 13 | β-catenin | 1989 | M. Ozawa, R. Kemler | Immunoprecipitation, identifying the intracellular protein acting as a scaffold for E-cadherin. | Discovered the core adhesion and Wnt signaling component. | Often overshadowed by its role at the cell adhesion membrane. | F-actin/G-actin Ratio measurement, Surface GLUT4myc assay (34) | Studying the crosstalk between Wnt and Insulin signaling. |
| 14 | MICAL | 2002 | Jonathan R Terman | The researchers identified MICAL by using the Yeast Two-Hybrid (Y2H) system to screen for proteins interacting with the Plexin A receptor, subsequently validating these findings through Co-immunoprecipitation (Co-IP) and Genetic Mutation analysis in Drosophila. | This multi-layered approach creates a robust validation pipeline that bridges the gap between large-scale molecular "hits" and definitive biological function in vivo. | The workflow requires significant time and resources to filter out experimental artifacts and remains limited in pinpointing the exact physical "directness" of the molecular links. | Spinning-disk confocal fluorescence microscopy, Structured illumination microscopy (SIM) (27) | Using super-resolution SIM and TIRF to observe the cytoskeleton. |
| 15 | PID1 (NYGGF4) | 2006 | Wang B | Using microarray techniques on human adipose tissue: discovered a novel gene with highly upregulated expression directly related to obesity and insulin resistance. | Mass quantitative discovery of pathology-related genes. | Large data analysis is prone to statistical noise. | Real-time PCR (RT-qPCR) technique for gene quantification (150) | Gene therapy via targeted in vivo RNAi intervention. |
| 16 | CHIP (STUB1) | 1999 | C.A. Ballinger | Gene library screening using the yeast two-hybrid technique: discovery of a TPR domain-containing protein acting as a co-chaperone and E3 ubiquitin ligase. | Discovered the intersection between the protein folding and degradation systems. | Required complex in vitro reactions to prove E3 ligase activity. | 2D-DIGE proteomic analysis, Hyperinsulinemic-euglycemic clamp (151) | Developing targeted protein degradation drugs. |
| 17 | HIF-1α | 1995 | G.L. Semenza, G.L. Wang | Purification by DNA affinity chromatography: identified the α subunit of the transcription factor that is sensitive to and directly responds to hypoxic conditions. | Precise identification of the oxygen-responsive factor. | Extremely sensitive to oxygen, easily degraded during sample processing. | Immunoprecipitation assays, Real-time PCR techniques (152) | Pharmacological stabilization of HIF-1α to mimic the effects of physical exercise. |
| 18 | EP300 (p300)/CREBBP (CBP) | 1993 | J.C. Chrivia | Protein interaction analysis: discovery of massive transcriptional co-activators directly binding to CREB and the E1A protein. | Identified oversized co-activators. | Extremely large mass made cloning and expression difficult. | Microarray analysis, Flow cytometry measuring lysine acetylation levels (153) | Studying post-translational modifications (Acetylation) that independently regulate transcription. |
| 19 | PDLIM7 (Enigma) | 1996 | S. Kuroda | Protein-interaction analysis demonstrated binding of LIM-domain proteins, including Enigma, to PKC. | Found an important scaffold for PKC. | Two-hybrid signals are easily lost if the proper 3D fold structure is lacking. | Rapid Amplification of cDNA Ends (RACE) technique; Northern blot to measure mRNA expression in tissues (Immunoprecipitation) | Discovery of interdomain structural interactions (LIM domain). |
| 20 | CAV3 (caveolin-3) | 1996 | Z. Tang | Applied Northern blot hybridization and cDNA cloning techniques: discovered Caveolin-3 as a structural scaffold protein forming caveolae, specifically expressed in muscle tissue. | Discovered the muscle tissue specificity of caveolae. | Radioactive Northern blot signals have zero spatial resolution. | Detergent-resistant membrane (DRM) fractionation via sucrose gradient centrifugation (43) | Single-molecule microscopy techniques clarifying the structural dynamics of caveolae. |
| 21 | APPL2 | 1999 | Y. Mitsuuchi | Through protein interaction analysis and sequence homology comparison with APPL1: identified APPL2 as an adaptor protein containing functional domains that directly interact with AKT and RAB5. | Rapidly found the functional homolog of the adaptor protein. | Its opposing function to APPL1 took years to clarify. | Antibody-coupled densitometric assay for GLUT4 measurement, Mass Spectrometry (73) | Optimizing medicinal chemistry targeting the intracellular BAR domain. |

**REFERENCES:**

1.	Salt IP, Connell JM, Gould GW. 5-Aminoimidazole-4-Carboxamide Ribonucleoside (Aicar) Inhibits Insulin-Stimulated Glucose Transport in 3t3-L1 Adipocytes. *Diabetes* (2000) 49(10):1649-56. doi: 10.2337/diabetes.49.10.1649.

2.	Fujimoto BA, Young M, Nakamura N, Ha H, Carter L, Pitts MW, et al. Disrupted Glucose Homeostasis and Skeletal-Muscle-Specific Glucose Uptake in an Exocyst Knockout Mouse Model. *J Biol Chem* (2021) 296:100482. Epub 20210227. doi: 10.1016/j.jbc.2021.100482.

3.	Kurth-Kraczek EJ, Hirshman MF, Goodyear LJ, Winder WW. 5' Amp-Activated Protein Kinase Activation Causes Glut4 Translocation in Skeletal Muscle. *Diabetes* (1999) 48(8):1667-71. doi: 10.2337/diabetes.48.8.1667.

4.	Bernard JR, Liao Y-H, Doerner PG, Ding Z, Hsieh M, Wang W, et al. An Amino Acid Mixture Is Essential to Optimize Insulin-Stimulated Glucose Uptake and Glut4 Translocation in Perfused Rodent Hindlimb Muscle. *J Appl Physiol* (2012) 113(1):97-104. doi: 10.1152/japplphysiol.01484.2011.

5.	Till M, Kolter T, Eckel J. Molecular Mechanisms of Contraction-Induced Translocation of Glut4 in Isolated Cardiomyocytes. *The American Journal of Cardiology* (1997) 80(3, Supplement 1):85A-9A. doi: <https://doi.org/10.1016/S0002-9149(97)00461-X>.

6.	Kishi K, Hayashi H, Wang L, Kamohara S, Tamaoka K, Shimizu T, et al. Gq-Coupled Receptors Transmit the Signal for Glut4 Translocation Via an Insulin-Independent Pathway. *J Biol Chem* (1996) 271(43):26561-8. doi: 10.1074/jbc.271.43.26561.

7.	Choi YO, Ryu HJ, Kim HR, Song YS, Kim C, Lee W, et al. Implication of Phosphorylation of the Myosin Ii Regulatory Light Chain in Insulin-Stimulated Glut4 Translocation in 3t3-F442a Adipocytes. *Exp Mol Med* (2006) 38(2):180-9. doi: 10.1038/emm.2006.22.

8.	Kane S, Lienhard GE. Calmodulin Binds to the Rab Gtpase Activating Protein Required for Insulin-Stimulated Glut4 Translocation. *Biochem Biophys Res Commun* (2005) 335(1):175-80. doi: 10.1016/j.bbrc.2005.07.056.

9.	Elmendorf JS, Chen D, Pessin JE. Guanosine 5′-O-(3-Thiotriphosphate) (Gtpγs) Stimulation of Glut4 Translocation Is Tyrosine Kinase-Dependent*. *Journal of Biological Chemistry* (1998) 273(21):13289-96. doi: 10.1074/jbc.273.21.13289.

10.	Vicogne J, Vollenweider D, Smith JR, Huang P, Frohman MA, Pessin JE. Asymmetric Phospholipid Distribution Drives <I>in Vitro</I> Reconstituted Snare-Dependent Membrane Fusion. *Proceedings of the National Academy of Sciences* (2006) 103(40):14761-6. doi: 10.1073/pnas.0606881103.

11.	Manna P, Jain SK. Pip3 but Not Pip2 Increases Glut4 Surface Expression and Glucose Metabolism Mediated by Akt/Pkcζ/Λ Phosphorylation in 3t3l1 Adipocytes. *Mol Cell Biochem* (2013) 381(1):291-9. doi: 10.1007/s11010-013-1714-7.

12.	Standaert ML, Bandyopadhyay G, Kanoh Y, Sajan MP, Farese RV. Insulin and Pip3 Activate Pkc-Ζ by Mechanisms That Are Both Dependent and Independent of Phosphorylation of Activation Loop (T410) and Autophosphorylation (T560) Sites. *Biochemistry* (2001) 40(1):249-55. doi: 10.1021/bi0018234.

13.	Kong AM, Horan KA, Sriratana A, Bailey CG, Collyer LJ, Nandurkar HH, et al. Phosphatidylinositol 3-Phosphate [Ptdins3p] Is Generated at the Plasma Membrane by an Inositol Polyphosphate 5-Phosphatase: Endogenous Ptdins3p Can Promote Glut4 Translocation to the Plasma Membrane. *Mol Cell Biol* (2006) 26(16):6065-81. doi: 10.1128/mcb.00203-06.

14.	Chaussade C, Pirola L, Bonnafous S, Blondeau F, Brenz-Verca S, Tronchère H, et al. Expression of Myotubularin by an Adenoviral Vector Demonstrates Its Function as a Phosphatidylinositol 3-Phosphate [Ptdins(3) P ] Phosphatase in Muscle Cell Lines: Involvement of Ptdins(3) P in Insulin-Stimulated Glucose Transport. *Molecular endocrinology (Baltimore, Md)* (2004) 17:2448-60. doi: 10.1210/me.2003-0261.

15.	Sbrissa D, Ikonomov OC, Filios C, Delvecchio K, Shisheva A. Functional Dissociation between Pikfyve-Synthesized Ptdins5p and Ptdins(3,5)P2 by Means of the Pikfyve Inhibitor Ym201636. *Am J Physiol Cell Physiol* (2012) 303(4):C436-46. Epub 20120523. doi: 10.1152/ajpcell.00105.2012.

16.	Yang J, Clarke JF, Ester CJ, Young PW, Kasuga M, Holman GD. Phosphatidylinositol 3-Kinase Acts at an Intracellular Membrane Site to Enhance Glut4 Exocytosis in 3t3-L1 Cells. *Biochem J* (1996) 313 ( Pt 1)(Pt 1):125-31. doi: 10.1042/bj3130125.

17.	Contreras-Ferrat AE, Toro B, Bravo R, Parra V, Vásquez C, Ibarra C, et al. An Inositol 1,4,5-Triphosphate (Ip3)-Ip3 Receptor Pathway Is Required for Insulin-Stimulated Glucose Transporter 4 Translocation and Glucose Uptake in Cardiomyocytes. *Endocrinology* (2010) 151(10):4665-77. Epub 20100804. doi: 10.1210/en.2010-0116.

18.	Török Dr, Patel N, JeBailey L, Thong FSL, Randhawa VK, Klip A, et al. Insulin but Not Pdgf Relies on Actin Remodeling and on Vamp2 for Glut4 Translocation in Myoblasts. *J Cell Sci* (2004) 117(22):5447-55. doi: 10.1242/jcs.01421.

19.	Lee HJ, Lee JO, Kim N, Kim JK, Kim HI, Lee YW, et al. Irisin, a Novel Myokine, Regulates Glucose Uptake in Skeletal Muscle Cells Via Ampk. *Mol Endocrinol* (2015) 29(6):873-81. Epub 20150331. doi: 10.1210/me.2014-1353.

20.	Chen Q, Xie B, Zhu S, Rong P, Sheng Y, Ducommun S, et al. A Tbc1d1 (Ser231ala)-Knockin Mutation Partially Impairs Aicar- but Not Exercise-Induced Muscle Glucose Uptake in Mice. *Diabetologia* (2017) 60(2):336-45. Epub 20161108. doi: 10.1007/s00125-016-4151-9.

21.	Lauritzen HP, Galbo H, Toyoda T, Goodyear LJ. Kinetics of Contraction-Induced Glut4 Translocation in Skeletal Muscle Fibers from Living Mice. *Diabetes* (2010) 59(9):2134-44. Epub 20100709. doi: 10.2337/db10-0233.

22.	Imamura T, Vollenweider P, Egawa K, Clodi M, Ishibashi K, Nakashima N, et al. G Alpha-Q/11 Protein Plays a Key Role in Insulin-Induced Glucose Transport in 3t3-L1 Adipocytes. *Mol Cell Biol* (1999) 19(10):6765-74. doi: 10.1128/mcb.19.10.6765.

23.	Ramm G, Slot JW, James DE, Stoorvogel W. Insulin Recruits Glut4 from Specialized Vamp2-Carrying Vesicles as Well as from the Dynamic Endosomal/Trans-Golgi Network in Rat Adipocytes. *Mol Biol Cell* (2000) 11(12):4079-91. doi: 10.1091/mbc.11.12.4079.

24.	Millar CA, Meerloo T, Martin S, Hickson GRX, Shimwell NJ, Wakelam MJO, et al. Adipsin and the Glucose Transporter Glut4 Traffic to the Cell Surface Via Independent Pathways in Adipocytes. *Traffic* (2000) 1(2):141-51. doi: 10.1034/j.1600-0854.2000.010206.x.

25.	Imamura T, Huang J, Dalle S, Ugi S, Usui I, Luttrell LM, et al. Β-Arrestin-Mediated Recruitment of the Src Family Kinase Yes Mediates Endothelin-1-Stimulated Glucose Transport*. *Journal of Biological Chemistry* (2001) 276(47):43663-7. doi: 10.1074/jbc.M105364200.

26.	Brewer PD, Habtemichael EN, Romenskaia I, Mastick CC, Coster AC. Insulin-Regulated Glut4 Translocation: Membrane Protein Trafficking with Six Distinctive Steps. *J Biol Chem* (2014) 289(25):17280-98. Epub 20140428. doi: 10.1074/jbc.M114.555714.

27.	Diaz-Vegas A, Norris DM, Jall-Rogg S, Cooke KC, Conway OJ, Shun-Shion AS, et al. A High-Content Endogenous Glut4 Trafficking Assay Reveals New Aspects of Adipocyte Biology. *Life Science Alliance* (2023) 6(1):e202201585. doi: 10.26508/lsa.202201585.

28.	Hatakeyama H, Morino T, Ishii T, Kanzaki M. Cooperative Actions of Tbc1d1 and As160/Tbc1d4 in Glut4-Trafficking Activities. *J Biol Chem* (2019) 294(4):1161-72. doi: 10.1074/jbc.RA118.004614.

29.	Hatakeyama H, Kanzaki M. Regulatory Mode Shift of Tbc1d1 Is Required for Acquisition of Insulin-Responsive Glut4-Trafficking Activity. *Mol Biol Cell* (2013) 24(6):809-17. Epub 20130116. doi: 10.1091/mbc.E12-10-0725.

30.	Bogan JS, Rubin BR, Yu C, Löffler MG, Orme CM, Belman JP, et al. Endoproteolytic Cleavage of Tug Protein Regulates Glut4 Glucose Transporter Translocation. *J Biol Chem* (2012) 287(28):23932-47. Epub 20120518. doi: 10.1074/jbc.M112.339457.

31.	Wang W, Hansen PA, Marshall BA, Holloszy JO, Mueckler M. Insulin Unmasks a Cooh-Terminal Glut4 Epitope and Increases Glucose Transport across T-Tubules in Skeletal Muscle. *J Cell Biol* (1996) 135(2):415-30. doi: 10.1083/jcb.135.2.415.

32.	Jordens I, Molle D, Xiong W, Keller SR, McGraw TE. Insulin-Regulated Aminopeptidase Is a Key Regulator of Glut4 Trafficking by Controlling the Sorting of Glut4 from Endosomes to Specialized Insulin-Regulated Vesicles. *Mol Biol Cell* (2010) 21(12):2034-44. Epub 20100421. doi: 10.1091/mbc.e10-02-0158.

33.	Kioumourtzoglou D, Pryor PR, Gould GW, Bryant NJ. Alternative Routes to the Cell Surface Underpin Insulin-Regulated Membrane Trafficking of Glut4. *J Cell Sci* (2015) 128(14):2423-9. Epub 20150612. doi: 10.1242/jcs.166561.

34.	Masson SWC, Sorrenson B, Shepherd PR, Merry TL. Β-Catenin Regulates Muscle Glucose Transport Via Actin Remodelling and M-Cadherin Binding. *Mol Metab* (2020) 42:101091. Epub 20201001. doi: 10.1016/j.molmet.2020.101091.

35.	Braiman L, Alt A, Kuroki T, Ohba M, Bak A, Tennenbaum T, et al. Activation of Protein Kinase C Zeta Induces Serine Phosphorylation of Vamp2 in the Glut4 Compartment and Increases Glucose Transport in Skeletal Muscle. *Mol Cell Biol* (2001) 21(22):7852-61. doi: 10.1128/mcb.21.22.7852-7861.2001.

36.	Bandyopadhyay G, Kanoh Y, Sajan MP, Standaert ML, Farese RV. Effects of Adenoviral Gene Transfer of Wild-Type, Constitutively Active, and Kinase-Defective Protein Kinase C-Lambda on Insulin-Stimulated Glucose Transport in L6 Myotubes. *Endocrinology* (2000) 141(11):4120-7. doi: 10.1210/endo.141.11.7766.

37.	Kotani K, Ogawa W, Hashiramoto M, Onishi T, Ohno S, Kasuga M. Inhibition of Insulin-Induced Glucose Uptake by Atypical Protein Kinase C Isotype-Specific Interacting Protein in 3t3-L1 Adipocytes*. *Journal of Biological Chemistry* (2000) 275(34):26390-5. doi: 10.1074/jbc.M002537200.

38.	Zhou QL, Park JG, Jiang ZY, Holik JJ, Mitra P, Semiz S, et al. Analysis of Insulin Signalling by Rnai-Based Gene Silencing. *Biochem Soc Trans* (2004) 32(Pt 5):817-21. doi: 10.1042/bst0320817.

39.	Liu X-J, Yang C, Gupta N, Zuo J, Chang Y-S, Fang F-D. Protein Kinase C-Ζ Regulation of Glut4 Translocation through Actin Remodeling in Cho Cells. *J Mol Med* (2007) 85(8):851-61. doi: 10.1007/s00109-007-0232-z.

40.	Liu LZ, Zhao HL, Zuo J, Ho SK, Chan JC, Meng Y, et al. Protein Kinase Czeta Mediates Insulin-Induced Glucose Transport through Actin Remodeling in L6 Muscle Cells. *Mol Biol Cell* (2006) 17(5):2322-30. Epub 20060308. doi: 10.1091/mbc.e05-10-0969.

41.	Standaert ML, Bandyopadhyay G, Perez L, Price D, Galloway L, Poklepovic A, et al. Insulin Activates Protein Kinases C-&#X3b6; and C-&#X3bb; by an Autophosphorylation-Dependent Mechanism and Stimulates Their Translocation to Glut4 Vesicles and Other Membrane Fractions in Rat Adipocytes *. *Journal of Biological Chemistry* (1999) 274(36):25308-16. doi: 10.1074/jbc.274.36.25308.

42.	Luiken JJ, Ouwens DM, Habets DD, van der Zon GC, Coumans WA, Schwenk RW, et al. Permissive Action of Protein Kinase C-Zeta in Insulin-Induced Cd36- and Glut4 Translocation in Cardiac Myocytes. *J Endocrinol* (2009) 201(2):199-209. Epub 20090309. doi: 10.1677/joe-09-0046.

43.	Standaert ML, Galloway L, Karnam P, Bandyopadhyay G, Moscat J, Farese RV. Protein Kinase C-Ζ as a Downstream Effector of Phosphatidylinositol 3-Kinase During Insulin Stimulation in Rat Adipocytes: Potential Role in Glucose Transport*. *Journal of Biological Chemistry* (1997) 272(48):30075-82. doi: 10.1074/jbc.272.48.30075.

44.	Fulcher FK, Smith BT, Russ M, Patel YM. Dual Role for Myosin Ii in Glut4-Mediated Glucose Uptake in 3t3-L1 Adipocytes. *Exp Cell Res* (2008) 314(17):3264-74. Epub 20080822. doi: 10.1016/j.yexcr.2008.08.007.

45.	Woody S, Stall R, Ramos J, Patel YM. Regulation of Myosin Light Chain Kinase During Insulin-Stimulated Glucose Uptake in 3t3-L1 Adipocytes. *PLoS One* (2013) 8(10):e77248. Epub 20131008. doi: 10.1371/journal.pone.0077248.

46.	Stall R, Ramos J, Kent Fulcher F, Patel YM. Regulation of Myosin Iia and Filamentous Actin During Insulin-Stimulated Glucose Uptake in 3t3-L1 Adipocytes. *Exp Cell Res* (2014) 322(1):81-8. Epub 20131225. doi: 10.1016/j.yexcr.2013.12.011.

47.	Shumay E, Song X, Wang HY, Malbon CC. Pp60src Mediates Insulin-Stimulated Sequestration of the Beta(2)-Adrenergic Receptor: Insulin Stimulates Pp60src Phosphorylation and Activation. *Mol Biol Cell* (2002) 13(11):3943-54. doi: 10.1091/mbc.e02-03-0174.

48.	Martin SS, Haruta T, Morris AJ, Klippel A, Williams LT, Olefsky JM. Activated Phosphatidylinositol 3-Kinase Is Sufficient to Mediate Actin Rearrangement and Glut4 Translocation in 3t3-L1 Adipocytes*. *Journal of Biological Chemistry* (1996) 271(30):17605-8. doi: 10.1074/jbc.271.30.17605.

49.	Asano T, Kanda A, Katagiri H, Nawano M, Ogihara T, Inukai K, et al. P110β Is up-Regulated During Differentiation of 3t3-L1 Cells and Contributes to the Highly Insulin-Responsive Glucose Transport Activity*. *Journal of Biological Chemistry* (2000) 275(23):17671-6. doi: 10.1074/jbc.M910391199.

50.	Somwar R, Niu W, Kim DY, Sweeney G, Randhawa VK, Huang C, et al. Differential Effects of Phosphatidylinositol 3-Kinase Inhibition on Intracellular Signals Regulating Glut4 Translocation and Glucose Transport*. *Journal of Biological Chemistry* (2001) 276(49):46079-87. doi: 10.1074/jbc.M109093200.

51.	Katagiri H, Asano T, Ishihara H, Inukai K, Shibasaki Y, Kikuchi M, et al. Overexpression of Catalytic Subunit P110&#X3b1; of Phosphatidylinositol 3-Kinase Increases Glucose Transport Activity with Translocation of Glucose Transporters in 3t3-L1 Adipocytes *. *Journal of Biological Chemistry* (1996) 271(29):16987-90. doi: 10.1074/jbc.271.29.16987.

52.	Ishiki M, Randhawa VK, Poon V, JeBailey L, Klip A. Insulin Regulates the Membrane Arrival, Fusion, and C-Terminal Unmasking of Glucose Transporter-4 Via Distinct Phosphoinositides*. *Journal of Biological Chemistry* (2005) 280(31):28792-802. doi: 10.1074/jbc.M500501200.

53.	Guan F, Yu B, Qi G-x, Hu J, Zeng D-y, Luo J. Chemical Hypoxia-Induced Glucose Transporter-4 Translocation in Neonatal Rat Cardiomyocytes. *Arch Med Res* (2008) 39(1):52-60. doi: 10.1016/j.arcmed.2007.06.022.

54.	Kjøbsted R, Treebak JT, Fentz J, Lantier L, Viollet B, Birk JB, et al. Prior Aicar Stimulation Increases Insulin Sensitivity in Mouse Skeletal Muscle in an Ampk-Dependent Manner. *Diabetes* (2014) 64(6):2042-55. doi: 10.2337/db14-1402.

55.	Yamaguchi S, Katahira H, Ozawa S, Nakamichi Y, Tanaka T, Shimoyama T, et al. Activators of Amp-Activated Protein Kinase Enhance Glut4 Translocation and Its Glucose Transport Activity in 3t3-L1 Adipocytes. *Am J Physiol Endocrinol Metab* (2005) 289(4):E643-9. Epub 20050531. doi: 10.1152/ajpendo.00456.2004.

56.	Webster I, Friedrich SO, Lochner A, Huisamen B. Amp Kinase Activation and Glut4 Translocation in Isolated Cardiomyocytes. *Cardiovasc J Afr* (2010) 21(2):72-8.

57.	Fryer LG, Foufelle F, Barnes K, Baldwin SA, Woods A, Carling D. Characterization of the Role of the Amp-Activated Protein Kinase in the Stimulation of Glucose Transport in Skeletal Muscle Cells. *Biochem J* (2002) 363(Pt 1):167-74. doi: 10.1042/0264-6021:3630167.

58.	Somwar R, Kim DY, Sweeney G, Huang C, Niu W, Lador C, et al. Glut4 Translocation Precedes the Stimulation of Glucose Uptake by Insulin in Muscle Cells: Potential Activation of Glut4 Via P38 Mitogen-Activated Protein Kinase. *Biochem J* (2001) 359(Pt 3):639-49. doi: 10.1042/0264-6021:3590639.

59.	Kayali AG, Eichhorn J, Haruta T, Morris AJ, Nelson JG, Vollenweider P, et al. Association of the Insulin Receptor with Phospholipase C-Γ (Plcγ) in 3t3-L1 Adipocytes Suggests a Role for Plcγ in Metabolic Signaling by Insulin*. *Journal of Biological Chemistry* (1998) 273(22):13808-18. doi: 10.1074/jbc.273.22.13808.

60.	Eichhorn J, Kayali AG, Austin DA, Webster NJG. Insulin Activates Phospholipase C-Γ1 Via a Pi-3 Kinase Dependent Mechanism in 3t3-L1 Adipocytes. *Biochem Biophys Res Commun* (2001) 282(2):615-20. doi: 10.1006/bbrc.2001.4616.

61.	Lorenzo M, Teruel T, Hernandez R, Kayali AG, Webster NJG. Plcγ Participates in Insulin Stimulation of Glucose Uptake through Activation of Pkcζ in Brown Adipocytes. *Exp Cell Res* (2002) 278(2):146-57. doi: 10.1006/excr.2002.5570.

62.	Chen H, Wertheimer SJ, Lin CH, Katz SL, Amrein KE, Burn P, et al. Protein-Tyrosine Phosphatases Ptp1b and Syp Are Modulators of Insulin-Stimulated Translocation of Glut4 in Transfected Rat Adipose Cells *. *Journal of Biological Chemistry* (1997) 272(12):8026-31. doi: 10.1074/jbc.272.12.8026.

63.	Usui I, Imamura T, Satoh H, Huang J, Babendure JL, Hupfeld CJ, et al. Grk2 Is an Endogenous Protein Inhibitor of the Insulin Signaling Pathway for Glucose Transport Stimulation. *EMBO J* (2004) 23(14):2821-9. Epub 20040708. doi: 10.1038/sj.emboj.7600297.

64.	Yoshizaki T, Maegawa H, Egawa K, Ugi S, Nishio Y, Imamura T, et al. Protein Phosphatase-2cα as a Positive Regulator of Insulin Sensitivity through Direct Activation of Phosphatidylinositol 3-Kinase in 3t3-L1 Adipocytes*. *Journal of Biological Chemistry* (2004) 279(21):22715-26. doi: 10.1074/jbc.M313745200.

65.	Takenaka N, Izawa R, Wu J, Kitagawa K, Nihata Y, Hosooka T, et al. A Critical Role of the Small Gtpase Rac1 in Akt2-Mediated Glut4 Translocation in Mouse Skeletal Muscle. *The FEBS Journal* (2014) 281(5):1493-504. doi: 10.1111/febs.12719.

66.	Beg M, Abdullah N, Thowfeik FS, Altorki NK, McGraw TE. Distinct Akt Phosphorylation States Are Required for Insulin Regulated Glut4 and Glut1-Mediated Glucose Uptake. *eLife* (2017) 6:e26896. doi: 10.7554/eLife.26896.

67.	Jiang ZY, Zhou QL, Coleman KA, Chouinard M, Boese Q, Czech MP. Insulin Signaling through Akt/Protein Kinase B Analyzed by Small Interfering Rna-Mediated Gene Silencing. *Proceedings of the National Academy of Sciences* (2003) 100(13):7569-74. doi: 10.1073/pnas.1332633100.

68.	Katome T, Obata T, Matsushima R, Masuyama N, Cantley LC, Gotoh Y, et al. Use of Rna Interference-Mediated Gene Silencing and Adenoviral Overexpression to Elucidate the Roles of Akt/Protein Kinase B Isoforms in Insulin Actions. *J Biol Chem* (2003) 278(30):28312-23. Epub 20030506. doi: 10.1074/jbc.M302094200.

69.	Ng Y, Ramm G, Burchfield JG, Coster ACF, Stöckli J, James DE. Cluster Analysis of Insulin Action in Adipocytes Reveals a Key Role for Akt at the Plasma Membrane *<Sup></Sup>. *J Biol Chem* (2010) 285(4):2245-57. doi: 10.1074/jbc.M109.060236.

70.	Ducluzeau PH, Fletcher LM, Welsh GI, Tavaré JM. Functional Consequence of Targeting Protein Kinase B/Akt to Glut4 Vesicles. *J Cell Sci* (2002) 115(Pt 14):2857-66. doi: 10.1242/jcs.115.14.2857.

71.	Bae SS, Cho H, Mu J, Birnbaum MJ. Isoform-Specific Regulation of Insulin-Dependent Glucose Uptake by Akt/Protein Kinase B. *J Biol Chem* (2003) 278(49):49530-6. Epub 20030930. doi: 10.1074/jbc.M306782200.

72.	Cong L-N, Chen H, Li Y, Zhou L, McGibbon MA, Taylor SI, et al. Physiological Role of Akt in Insulin-Stimulated Translocation of Glut4 in Transfected Rat Adipose Cells. *Mol Endocrinol* (1997) 11(13):1881-90. doi: 10.1210/mend.11.13.0027.

73.	Cheng KKY, Zhu W, Chen B, Wang Y, Wu D, Sweeney G, et al. The Adaptor Protein Appl2 Inhibits Insulin-Stimulated Glucose Uptake by Interacting with Tbc1d1 in Skeletal Muscle. *Diabetes* (2014) 63(11):3748-58. doi: 10.2337/db14-0337.

74.	Bisht B, Dey CS. Focal Adhesion Kinase Contributes to Insulin-Induced Actin Reorganization into a Mesh Harboring Glucose Transporter-4 in Insulin Resistant Skeletal Muscle Cells. *BMC Cell Biol* (2008) 9:48. Epub 20080904. doi: 10.1186/1471-2121-9-48.

75.	Kopietz F, Neuhaus M, Borreguero-Muñoz A, Kryvokhyzha D, Stenkula KG. Focal Adhesion Kinase Orchestrates Glut4 Translocation and Glucose Uptake Via Cytoskeletal Turnover in Primary Adipocytes. *The FASEB Journal* (2025) 39(10):e70660. doi: 10.1096/fj.202402764RR.

76.	Paul S, Yang L, Mattingly H, Goyal Y, Shvartsman SY, Veraksa A. Activation-Induced Substrate Engagement in Erk Signaling. *Mol Biol Cell* (2020) 31(4):235-43. Epub 20200108. doi: 10.1091/mbc.E19-07-0355.

77.	Bandyopadhyay G, Sajan MP, Kanoh Y, Standaert ML, Quon MJ, Reed BC, et al. Glucose Activates Protein Kinase C-Zeta /Lambda through Proline-Rich Tyrosine Kinase-2, Extracellular Signal-Regulated Kinase, and Phospholipase D: A Novel Mechanism for Activating Glucose Transporter Translocation. *J Biol Chem* (2001) 276(38):35537-45. Epub 20010719. doi: 10.1074/jbc.M106042200.

78.	Trefely S, Khoo PS, Krycer JR, Chaudhuri R, Fazakerley DJ, Parker BL, et al. Kinome Screen Identifies Pfkfb3 and Glucose Metabolism as Important Regulators of the Insulin/Insulin-Like Growth Factor (Igf)-1 Signaling Pathway. *J Biol Chem* (2015) 290(43):25834-46. Epub 20150904. doi: 10.1074/jbc.M115.658815.

79.	Uhm M, Bazuine M, Zhao P, Chiang S-H, Xiong T, Karunanithi S, et al. Phosphorylation of the Exocyst Protein Exo84 by Tbk1 Promotes Insulin-Stimulated Glut4 Trafficking. *Science Signaling* (2017) 10(471):eaah5085. doi: 10.1126/scisignal.aah5085.

80.	Grillo S, Grémeaux T, Le Marchand-Brustel Y, Tanti J. Potential Role of 3-Phosphoinositide-Dependent Protein Kinase 1 (Pdk1) in Insulin-Stimulated Glucose Transporter 4 Translocation in Adipocytes. *FEBS Lett* (1999) 461(3):277-9. doi: 10.1016/s0014-5793(99)01472-6.

81.	Nakashima N, Sharma PM, Imamura T, Bookstein R, Olefsky JM. The Tumor Suppressor Pten Negatively Regulates Insulin Signaling in 3t3-L1 Adipocytes *. *Journal of Biological Chemistry* (2000) 275(17):12889-95. doi: 10.1074/jbc.275.17.12889.

82.	Wada T, Sasaoka T, Funaki M, Hori H, Murakami S, Ishiki M, et al. Overexpression of Sh2-Containing Inositol Phosphatase 2 Results in Negative Regulation of Insulin-Induced Metabolic Actions in 3t3-L1 Adipocytes Via Its 5'-Phosphatase Catalytic Activity. *Mol Cell Biol* (2001) 21(5):1633-46. doi: 10.1128/mcb.21.5.1633-1646.2001.

83.	Ikonomov OC, Sbrissa D, Dondapati R, Shisheva A. Arpikfyve–Pikfyve Interaction and Role in Insulin-Regulated Glut4 Translocation and Glucose Transport in 3t3-L1 Adipocytes. *Exp Cell Res* (2007) 313(11):2404-16. doi: 10.1016/j.yexcr.2007.03.024.

84.	Ikonomov OC, Sbrissa D, Delvecchio K, Feng HZ, Cartee GD, Jin JP, et al. Muscle-Specific Pikfyve Gene Disruption Causes Glucose Intolerance, Insulin Resistance, Adiposity, and Hyperinsulinemia but Not Muscle Fiber-Type Switching. *Am J Physiol Endocrinol Metab* (2013) 305(1):E119-31. Epub 20130514. doi: 10.1152/ajpendo.00030.2013.

85.	Liu T, Yu B, Kakino M, Fujimoto H, Ando Y, Hakuno F, et al. A Novel Irs-1-Associated Protein, Dgkzeta Regulates Glut4 Translocation in 3t3-L1 Adipocytes. *Sci Rep* (2016) 6:35438. Epub 20161014. doi: 10.1038/srep35438.

86.	Liu S, Zhang J, Qi R, Deng B, Ni Y, Zhang C, et al. Camkii and Kalirin, a Rac1-Gef, Regulate Akt Phosphorylation Involved in Contraction-Induced Glucose Uptake in Skeletal Muscle Cells. *Biochem Biophys Res Commun* (2022) 610:170-5. doi: 10.1016/j.bbrc.2022.03.152.

87.	Illario M, Monaco S, Cavallo AL, Esposito I, Formisano P, D'Andrea L, et al. Calcium-Calmodulin-Dependent Kinase Ii (Camkii) Mediates Insulin-Stimulated Proliferation and Glucose Uptake. *Cell Signal* (2009) 21(5):786-92. Epub 20090109. doi: 10.1016/j.cellsig.2009.01.022.

88.	Chen J, Lu G, Wang QJ. Protein Kinase C-Independent Effects of Protein Kinase D3 in Glucose Transport in L6 Myotubes. *Mol Pharmacol* (2005) 67(1):152-62. doi: 10.1124/mol.104.004200.

89.	Tunduguru R, Chiu TT, Ramalingam L, Elmendorf JS, Klip A, Thurmond DC. Signaling of the P21-Activated Kinase (Pak1) Coordinates Insulin-Stimulated Actin Remodeling and Glucose Uptake in Skeletal Muscle Cells. *Biochem Pharmacol* (2014) 92(2):380-8. Epub 20140906. doi: 10.1016/j.bcp.2014.08.033.

90.	Kim JH, Kim H, Hwang KH, Chang JS, Park KS, Cha SK, et al. Wnk1 Kinase Is Essential for Insulin-Stimulated Glut4 Trafficking in Skeletal Muscle. *FEBS Open Bio* (2018) 8(11):1866-74. Epub 20181005. doi: 10.1002/2211-5463.12528.

91.	Haruta T, Morris AJ, Rose DW, Nelson JG, Mueckler M, Olefsky JM. Insulin-Stimulated Glut4 Translocation Is Mediated by a Divergent Intracellular Signaling Pathway(*). *J Biol Chem* (1995) 270(47):27991-4. doi: 10.1074/jbc.270.47.27991.

92.	Chiang S-H, Baumann CA, Kanzaki M, Thurmond DC, Watson RT, Neudauer CL, et al. Insulin-Stimulated Glut4 Translocation Requires the Cap-Dependent Activation of Tc10. *Nature* (2001) 410(6831):944-8. doi: 10.1038/35073608.

93.	Takenaka N, Araki N, Satoh T. Involvement of the Protein Kinase Akt2 in Insulin-Stimulated Rac1 Activation Leading to Glucose Uptake in Mouse Skeletal Muscle. *PLoS One* (2019) 14(2):e0212219. Epub 20190208. doi: 10.1371/journal.pone.0212219.

94.	Kawase K, Nakamura T, Takaya A, Aoki K, Namikawa K, Kiyama H, et al. Gtp Hydrolysis by the Rho Family Gtpase Tc10 Promotes Exocytic Vesicle Fusion. *Dev Cell* (2006) 11(3):411-21. doi: 10.1016/j.devcel.2006.07.008.

95.	Koumanov F, Pereira VJ, Richardson JD, Sargent SL, Fazakerley DJ, Holman GD. Insulin Regulates Rab3-Noc2 Complex Dissociation to Promote Glut4 Translocation in Rat Adipocytes. *Diabetologia* (2015) 58(8):1877-86. Epub 20150530. doi: 10.1007/s00125-015-3627-3.

96.	Chen Y, Wang Y, Zhang J, Deng Y, Jiang L, Song E, et al. Rab10 and Myosin-Va Mediate Insulin-Stimulated Glut4 Storage Vesicle Translocation in Adipocytes. *J Cell Biol* (2012) 198(4):545-60. doi: 10.1083/jcb.201111091.

97.	Kanzaki M, Watson RT, Artemyev NO, Pessin JE. The Trimeric Gtp-Binding Protein (G<Sub>Q</Sub>/G<Sub>11</Sub>) &#X3b1; Subunit Is Required for Insulin-Stimulated Glut4 Translocation in 3t3l1 Adipocytes *. *Journal of Biological Chemistry* (2000) 275(10):7167-75. doi: 10.1074/jbc.275.10.7167.

98.	Chen Y, Deng Y, Zhang J, Yang L, Xie X, Xu T. Gdi-1 Preferably Interacts with Rab10 in Insulin-Stimulated Glut4 Translocation. *Biochem J* (2009) 422(2):229-35. Epub 20090813. doi: 10.1042/bj20090624.

99.	Eguez L, Lee A, Chavez JA, Miinea CP, Kane S, Lienhard GE, et al. Full Intracellular Retention of Glut4 Requires As160 Rab Gtpase Activating Protein. *Cell Metab* (2005) 2(4):263-72. doi: 10.1016/j.cmet.2005.09.005.

100.	Kramer HF, Witczak CA, Taylor EB, Fujii N, Hirshman MF, Goodyear LJ. As160 Regulates Insulin- and Contraction-Stimulated Glucose Uptake in Mouse Skeletal Muscle *<Sup> </Sup>. *J Biol Chem* (2006) 281(42):31478-85. doi: 10.1016/S0021-9258(19)84060-7.

101.	Chavez JA, Roach WG, Keller SR, Lane WS, Lienhard GE. Inhibition of Glut4 Translocation by Tbc1d1, a Rab Gtpase-Activating Protein Abundant in Skeletal Muscle, Is Partially Relieved by Amp-Activated Protein Kinase Activation. *J Biol Chem* (2008) 283(14):9187-95. Epub 20080207. doi: 10.1074/jbc.M708934200.

102.	Davey JR, Humphrey SJ, Junutula JR, Mishra AK, Lambright DG, James DE, et al. <Scp>Tbc1d13</Scp> Is a <Scp>Rab35</Scp> Specific <Scp>Gap</Scp> That Plays an Important Role in <Scp>Glut4</Scp> Trafficking in Adipocytes. *Traffic* (2012) 13(10):1429-41. doi: 10.1111/j.1600-0854.2012.01397.x.

103.	Balamatsias D, Kong AM, Waters JE, Sriratana A, Gurung R, Bailey CG, et al. Identification of P-Rex1 as a Novel Rac1-Guanine Nucleotide Exchange Factor (Gef) That Promotes Actin Remodeling and Glut4 Protein Trafficking in Adipocytes. *J Biol Chem* (2011) 286(50):43229-40. Epub 20111015. doi: 10.1074/jbc.M111.306621.

104.	Sano H, Peck GR, Kettenbach AN, Gerber SA, Lienhard GE. Insulin-Stimulated Glut4 Protein Translocation in Adipocytes Requires the Rab10 Guanine Nucleotide Exchange Factor Dennd4c. *J Biol Chem* (2011) 286(19):16541-5. Epub 20110322. doi: 10.1074/jbc.C111.228908.

105.	Lodhi IJ, Chiang SH, Chang L, Vollenweider D, Watson RT, Inoue M, et al. Gapex-5, a Rab31 Guanine Nucleotide Exchange Factor That Regulates Glut4 Trafficking in Adipocytes. *Cell Metab* (2007) 5(1):59-72. doi: 10.1016/j.cmet.2006.12.006.

106.	Takenaka N, Nihata Y, Satoh T. Rac1 Activation Caused by Membrane Translocation of a Guanine Nucleotide Exchange Factor in Akt2-Mediated Insulin Signaling in Mouse Skeletal Muscle. *PLoS One* (2016) 11(5):e0155292. Epub 20160510. doi: 10.1371/journal.pone.0155292.

107.	Chiang SH, Hwang J, Legendre M, Zhang M, Kimura A, Saltiel AR. Tcgap, a Multidomain Rho Gtpase-Activating Protein Involved in Insulin-Stimulated Glucose Transport. *EMBO J* (2003) 22(11):2679-91. doi: 10.1093/emboj/cdg262.

108.	Chen XW, Leto D, Xiong T, Yu G, Cheng A, Decker S, et al. A Ral Gap Complex Links Pi 3-Kinase/Akt Signaling to Rala Activation in Insulin Action. *Mol Biol Cell* (2011) 22(1):141-52. Epub 20101209. doi: 10.1091/mbc.E10-08-0665.

109.	Lopez-Sanchez I, Ma GS, Pedram S, Kalogriopoulos N, Ghosh P. Giv/Girdin Binds Exocyst Subunit-Exo70 and Regulates Exocytosis of Glut4 Storage Vesicles. *Biochem Biophys Res Commun* (2015) 468(1-2):287-93. doi: 10.1016/j.bbrc.2015.10.111.

110.	Bose A, Cherniack AD, Langille SE, Nicoloro SM, Buxton JM, Park JG, et al. G(Alpha)11 Signaling through Arf6 Regulates F-Actin Mobilization and Glut4 Glucose Transporter Translocation to the Plasma Membrane. *Mol Cell Biol* (2001) 21(15):5262-75. doi: 10.1128/mcb.21.15.5262-5275.2001.

111.	Kee AJ, Yang L, Lucas CA, Greenberg MJ, Martel N, Leong GM, et al. An Actin Filament Population Defined by the Tropomyosin Tpm3.1 Regulates Glucose Uptake. *Traffic* (2015) 16(7):691-711. Epub 20150429. doi: 10.1111/tra.12282.

112.	Shrestha MM, Lim C-Y, Bi X, Robinson RC, Han W. Tmod3 Phosphorylation Mediates Ampk-Dependent Glut4 Plasma Membrane Insertion in Myoblasts. *Front Endocrinol (Lausanne)* (2021) Volume 12 - 2021. doi: 10.3389/fendo.2021.653557.

113.	Knudsen JR, Persson KW, Henriquez-Olguin C, Li Z, Di Leo N, Hesselager SA, et al. Microtubule-Mediated Glut4 Trafficking Is Disrupted in Insulin-Resistant Skeletal Muscle. *Elife* (2023) 12. Epub 20230419. doi: 10.7554/eLife.83338.

114.	Dawicki-McKenna JM, Goldman YE, Ostap EM. Sites of Glucose Transporter-4 Vesicle Fusion with the Plasma Membrane Correlate Spatially with Microtubules. *PLoS One* (2012) 7(8):e43662. Epub 20120820. doi: 10.1371/journal.pone.0043662.

115.	Kanzaki M, Pessin JE. Insulin-Stimulated Glut4 Translocation in Adipocytes Is Dependent Upon Cortical Actin Remodeling*210. *J Biol Chem* (2001) 276(45):42436-44. doi: 10.1074/jbc.M108297200.

116.	Kanzaki M, Watson RT, Khan AH, Pessin JE. Insulin Stimulates Actin Comet Tails on Intracellular Glut4-Containing Compartments in Differentiated 3t3l1 Adipocytes*210. *J Biol Chem* (2001) 276(52):49331-6. doi: 10.1074/jbc.M109657200.

117.	Yip MF, Ramm G, Larance M, Hoehn KL, Wagner MC, Guilhaus M, et al. Camkii-Mediated Phosphorylation of the Myosin Motor Myo1c Is Required for Insulin-Stimulated Glut4 Translocation in Adipocytes. *Cell Metab* (2008) 8(5):384-98. doi: 10.1016/j.cmet.2008.09.011.

118.	Yoshizaki T, Imamura T, Babendure JL, Lu JC, Sonoda N, Olefsky JM. Myosin 5a Is an Insulin-Stimulated Akt2 (Protein Kinase Bbeta) Substrate Modulating Glut4 Vesicle Translocation. *Mol Cell Biol* (2007) 27(14):5172-83. Epub 20070521. doi: 10.1128/mcb.02298-06.

119.	Boguslavsky S, Chiu T, Foley KP, Osorio-Fuentealba C, Antonescu CN, Bayer KU, et al. Myo1c Binding to Submembrane Actin Mediates Insulin-Induced Tethering of Glut4 Vesicles. *Mol Biol Cell* (2012) 23(20):4065-78. Epub 20120823. doi: 10.1091/mbc.E12-04-0263.

120.	Takazawa K, Noguchi T, Hosooka T, Yoshioka T, Tobimatsu K, Kasuga M. Insulin-Induced Glut4 Movements in C2c12 Myoblasts: Evidence against a Role of Conventional Kinesin Motor Proteins. *Kobe J Med Sci* (2008) 54(1):E14-22. Epub 20080523.

121.	Imamura T, Huang J, Usui I, Satoh H, Bever J, Olefsky JM. Insulin-Induced Glut4 Translocation Involves Protein Kinase C-Lambda-Mediated Functional Coupling between Rab4 and the Motor Protein Kinesin. *Mol Cell Biol* (2003) 23(14):4892-900. doi: 10.1128/mcb.23.14.4892-4900.2003.

122.	Guo H-L, Zhang C, Liu Q, Li Q, Lian G, Wu D, et al. The Axin/Tnks Complex Interacts with Kif3a and Is Required for Insulin-Stimulated Glut4 Translocation. *Cell Res* (2012) 22(8):1246-57. doi: 10.1038/cr.2012.52.

123.	Foster LJ, Rudich A, Talior I, Patel N, Huang X, Furtado LM, et al. Insulin-Dependent Interactions of Proteins with Glut4 Revealed through Stable Isotope Labeling by Amino Acids in Cell Culture (Silac). *J Proteome Res* (2006) 5(1):64-75. doi: 10.1021/pr0502626.

124.	Talior-Volodarsky I, Randhawa VK, Zaid H, Klip A. Α-Actinin-4 Is Selectively Required for Insulin-Induced Glut4 Translocation*. *Journal of Biological Chemistry* (2008) 283(37):25115-23. doi: 10.1074/jbc.M801750200.

125.	Robinson L, Pang S, Harris D, Heuser J, James D. Translocation of the Glucose Transporter (Glut4) to the Cell Surface in Permeabilized 3t3-L1 Adipocytes: Effects of Atp, Insulin, and Gtpγs and Localization of Glut4 to Clathrin Lattices. *The Journal of cell biology* (1992) 117:1181-96.

126.	Fujimoto BA, Young M, Carter L, Pang APS, Corley MJ, Fogelgren B, et al. The Exocyst Complex Regulates Insulin-Stimulated Glucose Uptake of Skeletal Muscle Cells. *Am J Physiol Endocrinol Metab* (2019) 317(6):E957-e72. Epub 20191008. doi: 10.1152/ajpendo.00109.2019.

127.	Wang S, Crisman L, Miller J, Datta I, Gulbranson DR, Tian Y, et al. Inducible Exoc7/Exo70 Knockout Reveals a Critical Role of the Exocyst in Insulin-Regulated Glut4 Exocytosis. *J Biol Chem* (2019) 294(52):19988-96. Epub 20191118. doi: 10.1074/jbc.RA119.010821.

128.	Randhawa VK, Bilan PJ, Khayat ZA, Daneman N, Liu Z, Ramlal T, et al. Vamp2, but Not Vamp3/Cellubrevin, Mediates Insulin-Dependent Incorporation of Glut4 into the Plasma Membrane of L6 Myoblasts. *Mol Biol Cell* (2000) 11:2403-17.

129.	Jagadish MN, Fernandez CS, Hewish DR, Macaulay SL, Gough KH, Grusovin J, et al. Insulin-Responsive Tissues Contain the Core Complex Protein Snap-25 (Synaptosomal-Associated Protein 25) a and B Isoforms in Addition to Syntaxin 4 and Synaptobrevins 1 and 2. *Biochem J* (1996) 317 ( Pt 3)(Pt 3):945-54. doi: 10.1042/bj3170945.

130.	Chamberlain L, Graham M, Kane S, Jackson J, Maier V, Burgoyne R, et al. The Synaptic Vesicle Protein, Cysteine-String Protein, Is Associated with the Plasma Membrane in 3t3-L1 Adipocytes and Interacts with Syntaxin 4. *J Cell Sci* (2001) 114:445-55. doi: 10.1242/jcs.114.2.445.

131.	Kioumourtzoglou D, Gould GW, Bryant NJ. Insulin Stimulates Syntaxin4 Snare Complex Assembly Via a Novel Regulatory Mechanism. *Mol Cell Biol* (2014) 34(7):1271-9. Epub 20140127. doi: 10.1128/mcb.01203-13.

132.	Bremner SK, Al Shammari WS, Milligan RS, Hudson BD, Sutherland C, Bryant NJ, et al. Pleiotropic Effects of Syntaxin16 Identified by Gene Editing in Cultured Adipocytes. *Front Cell Dev Biol* (2022) 10:1033501. Epub 20221118. doi: 10.3389/fcell.2022.1033501.

133.	Hosaka T, Brooks CC, Presman E, Kim SK, Zhang Z, Breen M, et al. P115 Interacts with the Glut4 Vesicle Protein, Irap, and Plays a Critical Role in Insulin-Stimulated Glut4 Translocation. *Mol Biol Cell* (2005) 16(6):2882-90. Epub 20050330. doi: 10.1091/mbc.e05-01-0072.

134.	Wang S, Liu Y, Crisman L, Wan C, Miller J, Yu H, et al. Genetic Evidence for an Inhibitory Role of Tomosyn in Insulin-Stimulated Glut4 Exocytosis. *Traffic* (2020) 21(10):636-46. doi: 10.1111/tra.12760.

135.	Nagano K, Takeuchi H, Gao J, Mori Y, Otani T, Wang D, et al. Tomosyn Is a Novel Akt Substrate Mediating Insulin-Dependent Glut4 Exocytosis. *The International Journal of Biochemistry & Cell Biology* (2015) 62:62-71. doi: 10.1016/j.biocel.2015.02.013.

136.	Zhang Y, Hu Y, Xie H, Liu M, Yang J, Ma C. Structural Mechanism of Soluble N-Ethylmaleimide-Sensitive Factor Attachment Protein Receptor Complex Assembly in Lipid Bilayers Revealed by Solid-State Nmr. *J Am Chem Soc* (2023) 145(19):10641-50. Epub 20230509. doi: 10.1021/jacs.3c00764.

137.	Saito T, Okada S, Nohara A, Tagaya Y, Osaki A, Oh IS, et al. Syntaxin4 Interacting Protein (Synip) Binds Phosphatidylinositol (3,4,5) Triphosphate. *PLoS One* (2012) 7(8):e42782. Epub 20120803. doi: 10.1371/journal.pone.0042782.

138.	Fecchi K, Volonte D, Hezel MP, Schmeck K, Galbiati F. Spatial and Temporal Regulation of Glut4 Translocation by Flotillin-1 and Caveolin-3 in Skeletal Muscle Cells. *FASEB J* (2006) 20(6):705-7. Epub 20060202. doi: 10.1096/fj.05-4661fje.

139.	Zhou L, Chen H, Lin CH, Cong L-N, McGibbon MA, Sciacchitano S, et al. Insulin Receptor Substrate-2 (Irs-2) Can Mediate the Action of Insulin to Stimulate Translocation of Glut4 to the Cell Surface in Rat Adipose Cells *. *Journal of Biological Chemistry* (1997) 272(47):29829-33. doi: 10.1074/jbc.272.47.29829.

140.	Cheng R, Qiu J, Zhou X-Y, Chen X-H, Zhu C, Qin D-N, et al. Knockdown of Steap4 Inhibits Insulin-Stimulated Glucose Transport and Glut4 Translocation Via Attenuated Phosphorylation of Akt, Independent of the Effects of Eea1. *Mol Med Report* (2011) 4:519-23. doi: 10.3892/mmr.2011.443.

141.	Hodgkinson CP, Mander A, Sale GJ. Identification of 80k-H as a Protein Involved in Glut4 Vesicle Trafficking. *Biochem J* (2005) 388(Pt 3):785-93. doi: 10.1042/bj20041845.

142.	Watanabe K, Matsumoto A, Tsuda H, Iwamoto S. N4bp2l1 Interacts with Dynactin and Contributes to Glut4 Trafficking and Glucose Uptake in Adipocytes. *J Diabetes Investig* (2021) 12(11):1958-66. Epub 20210717. doi: 10.1111/jdi.13623.

143.	Park SY, Ha BG, Choi GH, Lee W. N-Acetylated Α-Linked Acidic Dipeptidase Expressed in Rat Adipocytes Is Localized in the Insulin-Responsive Glucose Transporter (Glut4) Intracellular Compartments and Involved in the Insulin-Stimulated Glut4 Recruitment. *Arch Biochem Biophys* (2004) 424(1):11-22. doi: 10.1016/j.abb.2004.01.011.

144.	Xie X, Chen Y, Xue P, Fan Y, Deng Y, Peng G, et al. Ruvbl2, a Novel As160-Binding Protein, Regulates Insulin-Stimulated Glut4 Translocation. *Cell Res* (2009) 19(9):1090-7. doi: 10.1038/cr.2009.68.

145.	Koester AM, Geiser A, Laidlaw KME, Morris S, Cutiongco MFA, Stirrat L, et al. Efr3 and Phosphatidylinositol 4-Kinase Iiiα Regulate Insulin-Stimulated Glucose Transport and Glut4 Dispersal in 3t3-L1 Adipocytes. *Biosci Rep* (2022) 42(7). doi: 10.1042/bsr20221181.

146.	Liu L-Z, Cheung SCK, Lan L-L, Ho SKS, Chan JCN, Tong PCY. Microtubule Network Is Required for Insulin-Induced Signal Transduction and Actin Remodeling. *Mol Cell Endocrinol* (2013) 365(1):64-74. doi: 10.1016/j.mce.2012.09.005.

147.	Panico P, Hiriart M, Ostrosky-Wegman P, Salazar A. Tug Is a Calpain-10 Substrate Involved in the Translocation of Glut4 in Adipocytes. *J Mol Endocrinol* (2020) 65. doi: 10.1530/JME-19-0253.

148.	Habtemichael EN, Li DT, Alcázar-Román A, Westergaard XO, Li M, Petersen MC, et al. Usp25m Protease Regulates Ubiquitin-Like Processing of Tug Proteins to Control Glut4 Glucose Transporter Translocation in Adipocytes. *J Biol Chem* (2018) 293(27):10466-86. Epub 20180517. doi: 10.1074/jbc.RA118.003021.

149.	Wu J, Dong T, Chen T, Sun J, Luo J, He J, et al. Hepatic Exosome-Derived Mir-130a-3p Attenuates Glucose Intolerance Via Suppressing Phlpp2 Gene in Adipocyte. *Metabolism - Clinical and Experimental* (2020) 103. doi: 10.1016/j.metabol.2019.154006.

150.	Zeng X-Q, Zhang C-M, Tong M-L, Chi X, Li X-L, Ji C-B, et al. Knockdown of Nyggf4 Increases Glucose Transport in C2c12 Mice Skeletal Myocytes by Activation Irs-1/Pi3k/Akt Insulin Pathway. *J Bioenerg Biomembr* (2012) 44(3):351-5. doi: 10.1007/s10863-012-9438-z.

151.	McDonough H, Lenhart KC, Ronnebaum SM, Zhang C, An J, Portbury A, et al. Loss of Chip Expression Perturbs Glucose Homeostasis and Leads to Type Ii Diabetes through Defects in Microtubule Polymerization and Glucose Transporter Localization. *bioRxiv* (2017):166389. doi: 10.1101/166389.

152.	Sakagami H, Makino Y, Mizumoto K, Isoe T, Takeda Y, Watanabe J, et al. Loss of Hif-1α Impairs Glut4 Translocation and Glucose Uptake by the Skeletal Muscle Cells. *Am J Physiol Endocrinol Metab* (2014) 306(9):E1065-76. Epub 20140311. doi: 10.1152/ajpendo.00597.2012.

153.	Martins VF, LaBarge SA, Stanley A, Svensson K, Hung CW, Keinan O, et al. P300 or Cbp Is Required for Insulin-Stimulated Glucose Uptake in Skeletal Muscle and Adipocytes. *JCI Insight* (2022) 7(1). Epub 20220111. doi: 10.1172/jci.insight.141344.
