# Supplementary material for: Molecular architecture of GLUT4 trafficking and exocytosis in adipocytes and skeletal muscle: A systematic review and integrated conceptual framework
Source: Front Endocrinol (Lausanne). 2026 Oct 19;17:1932455. doi: 10.3389/fendo.2026.1932455 (PMC13725575; doi:10.3389/fendo.2026.1932455)
Supplement: Supplementary file 7 [file Table5.docx]

**Table S5.** Preclinical observations relevant to GLUT4-associated outcomes. Overview of pharmacological, exercise-related metabolic stimuli, and trafficking-directed therapeutic approaches derived from GLUT4 trafficking biology, including proposed molecular targets, mechanisms of action, and potential metabolic applications.

| **Preclinical observations relevant to GLUT4-associated outcomes** | **Therapeutic applications** | **Applications in diabetes prevention** |
| --- | --- | --- |
| Resveratrol (plant polyphenol) | [Pleiotropic, preclinical, model-specific, non-target-selective; association with GLUT4-related endpoints, not proof of direct trafficking modulation]. Associated with enhanced GLUT4 expression and metabolic improvements in preclinical models, potentially involving broad PI3K-AKT and AMPK signaling networks rather than acting as a direct modulator of the exocytic machinery (1, 2). | Acts as an antioxidant and improves insulin sensitivity, helping to prevent metabolic complications associated with obesity and diabetes (1, 2). |
| STX4 (syntaxin-4) (t-SNARE protein) | Supplementing STX4 in the pancreatic islets of diabetic preclinical models helps restore insulin secretion ability (3). | Transgenic mice overexpressing STX4 are protected against streptozotocin-induced β-cell mass loss and glucose intolerance, while also extending lifespan and metabolic health span (3, 4). |
| Berberine & Vanillic acid | [Pleiotropic, preclinical, model-specific, non-target-selective; association with GLUT4-related endpoints, not proof of direct trafficking modulation]. Observed to improve GLUT4 translocation endpoints through broader AMPK-dependent metabolic pathways, acting independently of canonical insulin signaling (1, 4). | Are considered potential natural compounds in controlling blood glucose and preventing the development of insulin resistance through the AMPK activation mechanism (1). |
| TBC1D1 (Rab-GAP protein) | TBC1D1 removal impairs GLUT4 redistribution to the cell membrane induced by muscle contraction (5). Inhibiting TBC1D1 via phosphorylation (by AMPK) helps promote glucose transport (6). | Plays a critical role in exercise tolerance, a key factor in maintaining metabolic health and preventing disease through physical activity (5). |
| TUG (ASPSCR1) (tether containing UBX domain for GLUT4) | Insulin stimulates the cleavage of the TUG protein by the enzyme USP25M, releasing the GLUT4 storage vesicles (GSVs) for transport to the membrane; this is a potential mechanism for regulating glucose uptake (7, 8). Reducing TUG mimics the effect of insulin, increasing the amount of GLUT4 on the cell surface (9). | Acetylation of TUG and deacetylation by SIRT2 regulate insulin sensitivity; this links the energy/redox state to blood glucose control ability, suggesting a role in preventing nutrition-related metabolic disorders (9). |
| AICAR (AMPK activator) | Stimulates glucose transport and GLUT4 translocation in skeletal muscle cells through AMPK activation, acting as an exercise-related metabolic stimulus (10). | It is used as a model to develop exercise-related metabolic stimuli to stimulate insulin-independent glucose uptake, useful for prevention in subjects with insulin resistance (10). |
| irisin (myokine) | Stimulates glucose uptake and GLUT4 translocation in skeletal muscle cells through a signaling pathway involving ROS, Ca2+, AMPK, and p38 MAPK (11). | It is a hormone secreted during exercise, playing a protective role against metabolic disorders by enhancing energy expenditure and insulin sensitivity (11). |
| PGC-1α (transcriptional coactivator) | Gene therapy using adenovirus to overexpress PGC-1α in skeletal muscle helps restore GLUT4 expression and increases glucose transport by 3-fold in animal models with diabetes (12). | Improves mitochondrial function and insulin sensitivity by enhancing GLUT4 expression, helping to prevent the progression of type 2 diabetes (12). |
| Mangiferin (extract of Salacia oblonga) | [Pleiotropic, preclinical, model-specific, non-target-selective; association with GLUT4-related endpoints, not proof of direct trafficking modulation]. Reported to enhance GLUT4 protein expression and surface presentation in specific L6 and 3T3-L1 cell models, though the precise targeted node within the trafficking network remains undefined (1). | Potential for use as a natural supplement to maintain stable blood glucose and prevent insulin resistance (1). |
| TBC1D4 (AS160) (Rab-GAP protein) | It is an important signaling node; phosphorylation of TBC1D4 by AKT (due to insulin) or AMPK (due to exercise) inactivates it, allowing Rab proteins (like RAB10) to activate GLUT4 translocation (13). | Exercise increases TBC1D4 phosphorylation, helping to enhance post-exercise insulin sensitivity, playing a critical role in diabetes prevention through physical activity (14). |

**REFERENCES**:

1.	Shanak S, Bassalat N, Barghash A, Kadan S, Ardah M, Zaid H. Drug Discovery of Plausible Lead Natural Compounds That Target the Insulin Signaling Pathway: Bioinformatics Approaches. *Evid Based Complement Alternat Med* (2022) 2022:2832889. Epub 20220320. doi: 10.1155/2022/2832889.

2.	Rezaei Farimani A, Goodarzi MT, Saidijam M, Yadegarazari R, Zarei S, Asadi S. Effect of Resveratrol on Snare Proteins Expression and Insulin Resistance in Skeletal Muscle of Diabetic Rats. *Iran J Basic Med Sci* (2019) 22(12):1408-14. doi: 10.22038/ijbms.2019.13988.

3.	Oh E, Miller RA, Thurmond DC. Syntaxin 4 Overexpression Ameliorates Effects of Aging and High-Fat Diet on Glucose Control and Extends Lifespan. *Cell Metab* (2015) 22(3):499-507. doi: 10.1016/j.cmet.2015.07.023.

4.	Sutandar V, Saleh M, Maritska Z. Glut4 as a Protein Target for T2dm Therapy with Natural Compounds. *Biology Medicine & Natural Product Chemistry* (2023) 12:289-293. doi: 10.14421/biomedich.2023.121.289-293.

5.	Whitfield J, Paglialunga S, Smith BK, Miotto PM, Simnett G, Robson HL, et al. Ablating the Protein Tbc1d1 Impairs Contraction-Induced Sarcolemmal Glucose Transporter 4 Redistribution but Not Insulin-Mediated Responses in Rats. *J Biol Chem* (2017) 292(40):16653-64. Epub 20170814. doi: 10.1074/jbc.M117.806786.

6.	Chavez JA, Roach WG, Keller SR, Lane WS, Lienhard GE. Inhibition of Glut4 Translocation by Tbc1d1, a Rab Gtpase-Activating Protein Abundant in Skeletal Muscle, Is Partially Relieved by Amp-Activated Protein Kinase Activation. *J Biol Chem* (2008) 283(14):9187-95. Epub 20080207. doi: 10.1074/jbc.M708934200.

7.	Bogan JS, Rubin BR, Yu C, Löffler MG, Orme CM, Belman JP, et al. Endoproteolytic Cleavage of Tug Protein Regulates Glut4 Glucose Transporter Translocation. *J Biol Chem* (2012) 287(28):23932-47. Epub 20120518. doi: 10.1074/jbc.M112.339457.

8.	Habtemichael EN, Li DT, Alcázar-Román A, Westergaard XO, Li M, Petersen MC, et al. Usp25m Protease Regulates Ubiquitin-Like Processing of Tug Proteins to Control Glut4 Glucose Transporter Translocation in Adipocytes. *J Biol Chem* (2018) 293(27):10466-86. Epub 20180517. doi: 10.1074/jbc.RA118.003021.

9.	Belman JP, Bian RR, Habtemichael EN, Li DT, Jurczak MJ, Alcázar-Román A, et al. Acetylation of Tug Protein Promotes the Accumulation of Glut4 Glucose Transporters in an Insulin-Responsive Intracellular Compartment. *J Biol Chem* (2015) 290(7):4447-63. Epub 20150105. doi: 10.1074/jbc.M114.603977.

10.	Merrill GF, Kurth EJ, Hardie DG, Winder WW. Aica Riboside Increases Amp-Activated Protein Kinase, Fatty Acid Oxidation, and Glucose Uptake in Rat Muscle. *Am J Physiol* (1997) 273(6):E1107-12. doi: 10.1152/ajpendo.1997.273.6.E1107.

11.	Lee HJ, Lee JO, Kim N, Kim JK, Kim HI, Lee YW, et al. Irisin, a Novel Myokine, Regulates Glucose Uptake in Skeletal Muscle Cells Via Ampk. *Mol Endocrinol* (2015) 29(6):873-81. Epub 20150331. doi: 10.1210/me.2014-1353.

12.	Khan MT, Al-Dhaleai RE, Alayadhi SM, Alhalwachi Z, Butler AE. The Role of Gene Therapy and Rna-Based Therapeutic Strategies in Diabetes. *Int J Mol Sci* (2025) 26(21):10264. 10.3390/ijms26211026.

13.	Sano H, Kane S, Sano E, Mı̂inea CP, Asara JM, Lane WS, et al. Insulin-Stimulated Phosphorylation of a Rab Gtpase-Activating Protein Regulates Glut4 Translocation *. *Journal of Biological Chemistry* (2003) 278(17):14599-602. doi: 10.1074/jbc.C300063200.

14.	Sakamoto K, Holman GD. Emerging Role for As160/Tbc1d4 and Tbc1d1 in the Regulation of Glut4 Traffic. *Am J Physiol Endocrinol Metab* (2008) 295(1):E29-37. Epub 20080513. doi: 10.1152/ajpendo.90331.2008.
